# Using open data and open-source software to develop spatial indicators of urban design and transport features for achieving healthy and sustainable cities

**DOI:** 10.1016/S2214-109X(22)00072-9

**Published:** 2022-06

**Authors:** Geoff Boeing, Carl Higgs, Shiqin Liu, Billie Giles-Corti, James F Sallis, Ester Cerin, Melanie Lowe, Deepti Adlakha, Erica Hinckson, Anne Vernez Moudon, Deborah Salvo, Marc A Adams, Ligia V Barrozo, Tamara Bozovic, Xavier Delclòs-Alió, Jan Dygrýn, Sara Ferguson, Klaus Gebel, Thanh Phuong Ho, Poh-Chin Lai, Joan C Martori, Kornsupha Nitvimol, Ana Queralt, Jennifer D Roberts, Garba H Sambo, Jasper Schipperijn, David Vale, Nico Van de Weghe, Guillem Vich, Jonathan Arundel

**Affiliations:** Department of Urban Planning and Spatial Analysis, Sol Price School of Public Policy, University of Southern California, Los Angeles, CA, USA (G Boeing PhD); Healthy Liveable Cities Lab, RMIT University, Melbourne, VIC, Australia (C Higgs MPH, Prof B Giles-Corti PhD, J Arundel PhD); School of Public Policy and Urban Affairs, Northeastern University, Boston, MA, USA (S Liu MS); Telethon Kids Institute, The University of Western Australia, Perth, WA, Australia (Prof B Giles-Corti); Mary MacKillop Institute for Health Research, Australian Catholic University, Melbourne, VIC, Australia (Prof J F Sallis PhD, Prof E Cerin PhD); Herbert Wertheim School of Public Health and Human Longevity Science, University of California, San Diego, CA, USA (Prof J F Sallis); School of Public Health (Prof E Cerin) and Department of Geography (Prof P-C Lai PhD), The University of Hong Kong, Hong Kong Special Administrative Region, China; Melbourne Centre for Cities (M Lowe PhD) and Transport, Health and Urban Design Research Lab, Melbourne School of Design (T P Ho MSc), University of Melbourne, Melbourne, VIC, Australia; Department of Landscape Architecture and Environmental Planning, Natural Learning Initiative, College of Design, North Carolina State University, Raleigh, NC, USA (D Adlakha PhD); Human Potential Centre, School of Sport and Recreation, Auckland University of Technology, Auckland, New Zealand (Prof E Hinckson PhD, T Bozovic PhD); Department of Urban Design and Planning, Urban Form Lab, University of Washington, Seattle, WA, USA (Prof A Vernez Moudon DSc); Prevention Research Center, Brown School, Washington University in St Louis, St Louis, MO, USA (D Salvo PhD); College of Health Solutions, Julie Ann Wrigley Global Futures Laboratory, Arizona State University, Phoenix, AZ, USA (M A Adams PhD); Department of Geography, School of Philosophy, Literature, and Human Sciences (L V Barrozo PhD) and Institute of Advanced Studies (L V Barrozo), University of São Paulo, São Paulo, Brazil; ISGlobal, Barcelona’s Institute for Global Health, Barcelona, Spain (G Vich PhD); Department of Geography, Rovira i Virgili University, Vila-seca, Spain (G Vich, X Delclòs-Alió PhD); Faculty of Physical Culture, Palacký University Olomouc, Olomouc, Czech Republic (J Dygrýn PhD); School of Natural and Built Environment, Queen’s University Belfast, Belfast, UK (S Ferguson PhD); Australian Centre for Public and Population Health Research, School of Public Health, University of Technology Sydney, Sydney, NSW, Australia (K Gebel PhD); Prevention Research Collaboration, School of Public Health, Faculty of Medicine and Health, University of Sydney, Sydney, NSW, Australia (K Gebel); Department of Economics and Business, University of Vic—Central University of Catalonia, Vic, Spain (J C Martori PhD); Office of the Permanent Secretary for the Bangkok Metropolitan Administration, Bangkok, Thailand (K Nitvimol MA); AFIPS Research Group, Department of Nursing, University of Valencia, Valencia, Spain (A Queralt PhD); Department of Kinesiology, School of Public Health, University of Maryland, College Park, MD, USA (J D Roberts DrPH); Department of Geography, University of Maiduguri, Maiduguri, Nigeria (G H Sambo PhD); Department of Sport Science and Clinical Biomechanics, University of Southern Denmark, Odense, Denmark (J Schipperijn PhD); Research Centre for Architecture, Urbanism and Design, Lisbon School of Architecture, University of Lisbon, Lisbon, Portugal (D Vale PhD); Department of Geography, Ghent University, Ghent, Belgium (N Van de Weghe PhD)

## Abstract

Benchmarking and monitoring of urban design and transport features is crucial to achieving local and international health and sustainability goals. However, most urban indicator frameworks use coarse spatial scales that either only allow between-city comparisons, or require expensive, technical, local spatial analyses for within-city comparisons. This study developed a reusable, open-source urban indicator computational framework using open data to enable consistent local and global comparative analyses. We show this framework by calculating spatial indicators—for 25 diverse cities in 19 countries—of urban design and transport features that support health and sustainability. We link these indicators to cities’ policy contexts, and identify populations living above and below critical thresholds for physical activity through walking. Efforts to broaden participation in crowdsourcing data and to calculate globally consistent indicators are essential for planning evidence-informed urban interventions, monitoring policy effects, and learning lessons from peer cities to achieve health, equity, and sustainability goals.

## Introduction

Policies that determine cities’ urban form, land use patterns, and transport opportunities also determine health and sustainability. Creating healthier and more sustainable cities is a global priority integral to achieving the UN’s Sustainable Development Goals (SDGs) and WHO’s health equity goals.^1^ Various indicator frameworks have been proposed to monitor progress towards these goals. However, most existing frameworks rely on citywide measures and focus on comparisons between cities. Although such comparisons are useful for determining priorities and interventions at the international and national levels, within-city (ie, neighbourhood-level) comparisons are key to unlocking the full potential of city planning to unmask and attenuate local urban health inequities.^2,3^

### Within-city versus between-city spatial indicators

Maps can help local and regional planners to reveal the spatial distribution of health-promoting infrastructure and amenities within cities (eg, walkable streets, public transport, daily living needs, and green spaces), and identify inequities in access. Mapped neighbourhood-level spatial indicators facilitate comparisons within cities, highlight resource distribution and areas needing interventions, encourage accountability, and empower communities to advocate for improvements.4 Growing access to big data and high-powered computing enables neighbourhood-level spatial indicators to be developed and disseminated more readily.

Urban policy targets are often set at a citywide level (eg, percentage of city population with access to amenities).^3^ Neighbourhood-level spatial indicators help planners to identify differences in access to urban design and transport features that support healthy and sustainable lifestyles, to better target local interventions. However, planners also need a means of aggregating consistently measured neighbourhood-level spatial indicators to the city scale, to compare between cities (benchmarking) and over time (monitoring). These are crucial first steps towards achieving urban health and sustainability goals. Nevertheless, many prominent indicator guidelines do not address measurement standards, indicator targets, or data acquisition (panel 1).

### Creating globally applicable city planning spatial indicators

Creating high-quality, fine-grained spatial indicators to measure progress towards healthy and sustainable cities worldwide presents technical challenges for both between-city and within-city comparisons.^7–9^ Although some cities collect and maintain high-quality,^10^ fine-grained data on land use, transport infrastructure, and socioeconomic characteristics, many do not. Even when such data exist, they might not be publicly available to researchers and practitioners. Researchers doing comparative analyses—particularly international analyses—must account for region-specific and dataset-specific inconsistencies in assumptions, standards, scales, and timelines. Data quality varies widely, as do digitisation standards and encodings, collection dates, local meanings of transport infrastructure or land use classifications, and spatial scales (eg, defining the city as an incorporated municipality, urbanised area, or metropolitan area).^11^

Lack of access to software, training, and resources also constrains indicator creation. Closed-source, proprietary geographic information systems are often expensive, do not lend themselves to open science or reproducibility, and fit poorly with modern data science practices.^12^ Advanced spatial analysis requires extensive training, but such expertise can be uncommon in government agencies and can be difficult or expensive to procure from the private sector. Resource constraints pose a particular challenge in low-income and middle-income countries, where data availability, data quality, and local technical capacity might be scarce. These limitations thwart efforts to develop actionable indicators to track the creation of healthy and sustainable urban environments in our planet’s most rapidly developing cities, and constrain governments’ capacity to develop evidence-based policies and monitor their effects.^13^

### A 21st century approach to calculating spatial indicators

Given the importance of urban spatial indicators to benchmark and monitor cities and inform interventions, a better model for creating indicators would leverage emerging open-source software and open-data commons to build high-quality, accessible, free tools for calculating and visualising such indicators. Open-data sources with global scope offer opportunities to measure and analyse urban health and sustainability indicators in diverse geographical contexts.^14–18^ OpenStreetMap (OSM) is a crowdsourced mapping project that provides open access to regularly updated spatial data worldwide, coded according to consistent and community-led guidelines.^19^

This third paper in the Series on urban design, transport, and health addresses the need to better measure, map, and compare urban design and transport features important for creating healthy and sustainable cities. We present an open-source software framework that uses open data to calculate spatial indicators within and between cities around the world, including in understudied and under-resourced countries. We show the feasibility and utility of our approach by creating a cross-sectional snapshot of priority indicators recommended in the first *Lancet* Series (Series 1) on urban design, transport, and health, showing between-city comparisons, and mapping within-city spatial inequities.^20^ We link these indicators to the local policy contexts identified by Lowe and colleagues in the first paper in this Series (Series 2),^21^ and identify populations living above and below the critical thresholds identified by Cerin and colleagues in the second paper in this Series.^22^ We discuss the practical value of this tool and empirical findings for policy making. The paper concludes with a call for action: to build healthy and sustainable cities, we must better measure city building and we must build healthy and sustainable cities for all—not just for some—by reducing within-city inequities.

### Measuring spatial indicators of urban design and transport features for healthy and sustainable cities

#### International collaboration network

The Global Healthy and Sustainable City Indicators Collaboration comprises a network of built environment and health researchers, formed to develop a framework for assessing the progress of urban design and transport features that support healthy and sustainable cities. The network comprises a core team of multidisciplinary researchers working with local experts, including academics and city planners, across 25 cities in 19 lower-middle-income to high-income countries across six world regions (appendix p 4). Lowe and colleagues^21^ describe the characteristics and sampling methods for these cities. Investigators were contacted through international networks and at conferences to volunteer to lead participation of cities.

#### Open-source framework

We developed an open-source software framework to calculate spatial indicators using open data at both fine-grained and aggregated levels, supporting within-city and between-city comparisons, as described by Liu and colleagues.^23^ Detailed descriptions of these methods, which were created in conjunction with collaborators, appear in the appendix (pp 2–23), including urban study region boundary definitions, source data to support comparable analyses of what we define as local neighbourhood features across the cities, and reproducible workflows for indicator estimation. We use the term neighbourhood here in a technical sense, referring to a walkable catchment within some distance threshold of a residential reference point, rather than the colloquial sense of social or political boundaries. We generally defined urban study regions using city administrative boundaries and the Global Human Settlements 2015 urban centres layer.^24^ We derived pedestrian-accessible street networks and built environment features from OSM, with validation conducted with local collaborators. To support between-city analyses of urban neighbourhoods, we generated a 250 m grid associated with 2015 population estimates^25^ to summarise the indicators’ distribution at high resolution for mapping with regard to population.

For each city, we calculated spatial indicators of urban design and transport features that support healthy and sustainable cities.^20,26^ The following methods and definitions are detailed in the appendix (pp 2–23). Indicators were calculated for sample points generated at regular 30 m intervals along the pedestrian street network for populated areas in each city’s urban region. These sample points represent an assumed spatial distribution of dwellings in each city to facilitate the measurement of local neighbourhood characteristics. The 1000 m (approximately 13 min walking time^27^) extent of the pedestrian network reachable from each sample point was intersected with the 250 m urban neighbourhood grid to represent a local walkable catchment area: a computationally tractable approximation of the sausage buffer method for walkable catchments^28,29^ as described by Liu and colleagues.^23^ We estimated population and street intersection densities in each sample point’s local walkable catchment. The nearest distance to several features—healthy food markets, convenience stores, public transport stops, and public open space entry points—was estimated for each sample point and evaluated against an accessibility threshold^5^ of 500 m using a binary access score (equal to 1 if the estimated access distance was within 500 m, and 0 otherwise). Access to public transport stops was evaluated against three criteria: (1) any, and where transport schedule data were retrievable; (2) average weekday daytime service every 30 min or less; and (3) serviced every 20 min or less. Public transport schedule data were obtained from transport agencies in general transit feed specification (GTFS) format. GTFS is an established standard for representing public transportation schedules used for urban transport research in diverse contexts. Two kinds of public open spaces were measured: any, and larger than 1·5 hectares. These public transport and open space typologies are associated with active transport behaviours, following those measured by Arundel and colleagues.^4^ We summarised each sample point’s local walkable environment using two composite indicators: a daily living score^4,30,31^ for local access to a mix of amenities (summing equal-weighted binary access scores to a healthy food market, a convenience store, and a public transport stop within 500 m); and a local walkability index^4,31^ that sums equal-weighted standardised scores of population density, street intersection density, and daily living score. These measures of well serviced and walkable neighbourhoods are standard in the built environment and health literature,^32,33^ with well established associations with physical activity and walking for transport.^4,30,31^

Residential point measures were aggregated and averaged to 250 m hexagonal cells serving as empirically derived neighbourhoods. These urban neighbourhoods were the spatial units used to characterise the within-city and between-city distribution of indicators in absolute terms relative to the city (within-city estimates), and relative to all cities via Z scores (between-city estimates). We conducted a spatial analysis of population and intersection density indicators for two physical activity scenarios identified by Cerin and colleagues.^22^ Scenario A meets the threshold for 80% probability of walking for transport, and scenario B meets the threshold for reaching WHO’s target of more or equal to a 15% relative reduction in insufficient physical activity through walking.

### How cities performed against the indicators

#### Population access to amenities

Table 1 presents estimates of the population percentage within a 500 m walk to amenities, alongside citywide estimates of 2015 transport-sector particulate matter (PM_2·5_) emissions, where available.^24^ Broad variation exists between cities, but cities in middle-income countries tended to have lower estimates of population access to amenities than cities in high-income countries. In contrast, transport-sector PM_2·5_ emissions were higher in cities of middle-income countries (1621·8 tonnes per year on average) than high-income countries (333·1 tonnes per year).

The population percentage within a 500 m walk to a healthy food market varied from 6% (Phoenix, AZ, USA) to 70% (Bern, Switzerland). European cities had the highest estimates (53% on average), whereas all three US cities were in the lowest quartile, each less than 20%. The three Australian cities, along with Maiduguri (Nigeria), Bangkok (Thailand), and Chennai (India), also exhibited low access (less than a quarter of the population had such access). On average, access to convenience stores within 500 m (40%) was slightly higher than access to healthy food markets (36%). This difference was particularly the case for cities with low access to healthy food. For example, 21% of the population of Phoenix had a convenience store within 500 m, more than three times greater than the population percentage that had access to healthy food. An exception to this pattern was Chennai, with an estimate of 16% for convenience stores, compared with 20% for healthy food. It is likely that not all existing healthy food market locations were available through OSM, particularly in cities like Bangkok, Chennai, Hanoi (Vietnam), and Maiduguri, where informal stalls might be important sources of healthy food.

Access to any public transport stop (eg, bus, ferry, train, or tram) within 500 m was achieved for more than 60% of the population in most cities, with three cities in middle-income countries as exceptions: Maiduguri (10%), Mexico City (Mexico; 36%) and Chennai (39%). However, in these cities the local transport context must be considered, as informal collective transport options play an important role, but spatial data to track them is scarce. Nevertheless, the disparity between estimated access to formal public transport infrastructure in middle-income and high-income countries was notable, and might be a factor in the observed trend of approximately five-fold higher PM_2·5_ emissions in middle-income versus high-income country cities, notwithstanding considerable between-city variation for both groups (table 1).

Accounting for public transport service frequency for cities where such data were available substantially reduced estimates of accessibility. The average population percentage with access to any stops with service every 30 min was 70% (SD 27). For service every 20 min the average city estimate fell to 45% (SD 23). For example, 87% of the population of Melbourne, VIC, Australia, had access to any public transport, but only 67% had access to stops with weekday service every 30 min, and only 49% with service every 20 min—less than the average for cities in high-income countries (55% [SD 15]). In other cities there were modest reductions in population access when adjusting for service frequency, indicating broad consistency in proximity and service frequency. For example, São Paulo (Brazil), Bern, and Lisbon (Portugal) had greater than 90% population access to public transport with average weekday service frequency of 20 min or less.

For most cities, the average population percentage with access to public open space within 500 m walking distance was relatively high at 76% (SD 22). Some policy settings mandate universal access, and any estimates less than 100% suggest equity gaps that need addressing. Some cities had low estimates (eg, Maiduguri and Bangkok), although this might reflect limitations of measuring public open space in different cities using OSM data. Once access was restricted to public open spaces larger than 1·5 hectares, population access dropped by approximately 10 percentage points on average, to 66% (SD 26). However, in the European cities of Bern, Vic (Spain), and Odense (Denmark), 70% or more of the population had access within 500 m to such larger public open space. Substantial inequities in access to public open space within 500 m were apparent between cities in middle-income and high-income countries: only 42% of the population in cities of middle-income countries had access, compared with 75% in cities of high-income countries.

#### Percentage of population meeting thresholds for urban design and transport features to support active lifestyles

Cerin and colleagues^22^ identified thresholds to support active lifestyles and achieve WHO physical activity targets. On average, less than half of the population in cities of high-income countries lived in neighbourhoods reaching the population density threshold for 80% probability of walking for transport (scenario A, 49%) or meeting WHO’s target for reducing insufficient physical activity through walking (scenario B, 38%), compared with 98% of the population for both targets in cities of middle-income countries (table 2). Cities with the highest estimated population percentages living in neighbourhoods with population densities that support higher transport walking (table 2) were in Africa (Maiduguri), Asia (Bangkok, Chennai, Hanoi, and Hong Kong), and Latin America (Mexico City and São Paulo). These cities all exceeded 90% of the population for both scenarios A and B, as did Lisbon, Barcelona (Spain), and Valencia (Spain). Belfast (UK), Graz (Austria), and Sydney (NSW, Australia) exceeded 50% for at least one of the two scenarios. However, Ghent (Belgium), Odense, Olomouc (Czech Republic), and Adelaide (SA, Australia) had notably low population density estimates that met neither population threshold. In Baltimore (MD, USA), Phoenix, Seattle (WA, USA), Auckland (New Zealand), Melbourne, Cologne (Germany), and Vic, less than 50% of the population met density thresholds. For scenario B, on average 97% (SD 3) of the population in cities of middle-income countries lived in neighbourhoods supporting densities meeting or exceeding the lower threshold for reaching WHO’s target of more than or equal to a 15% relative reduction in insufficient physical activity through combined transport and recreational walking, compared with 38% (SD 35) for cities in high-income countries, across which population density was more variable.

Estimates of the population percentage living in neighbourhoods with intersection density meeting thresholds to support higher physical activity displayed a distinct pattern for many cities when compared with the marginal population density characteristics summarised previously. There were no clear differences in intersection density estimates by country income classification (as observed with population density), with broad variation between cities regardless of country income grouping. Cities with high population estimates (>70% for both scenarios) of meeting or exceeding thresholds for population density and intersection density included Mexico City, São Paulo, Hong Kong, Chennai, Lisbon, Barcelona, and Valencia. Cities with moderate estimates for neighbourhood population density (>40%) but high for intersection density (>70%) included Bern, Belfast, and Graz. Cities with lower population density estimates, but moderate to high population exposure (>50%) to intersection density, included Baltimore, Phoenix, Ghent, Olomouc, Odense, Cologne, and Vic. Seattle had lower population percentage exposure estimates for both population density and intersection density than the other two US cities in this study. Maiduguri, Bangkok, and Hanoi each had high neighbourhood population density exposures but moderate to low intersection density exposure. Sydney had moderate neighbourhood population density exposure but lower exposure for intersection density, whereas Auckland and Melbourne had lower population densities and lower intersection density population exposure estimates, and Adelaide had both low population density and intersection density (<40%).

#### Spatial distribution of walkable neighbourhoods and access to public open space

**Figure 1:**
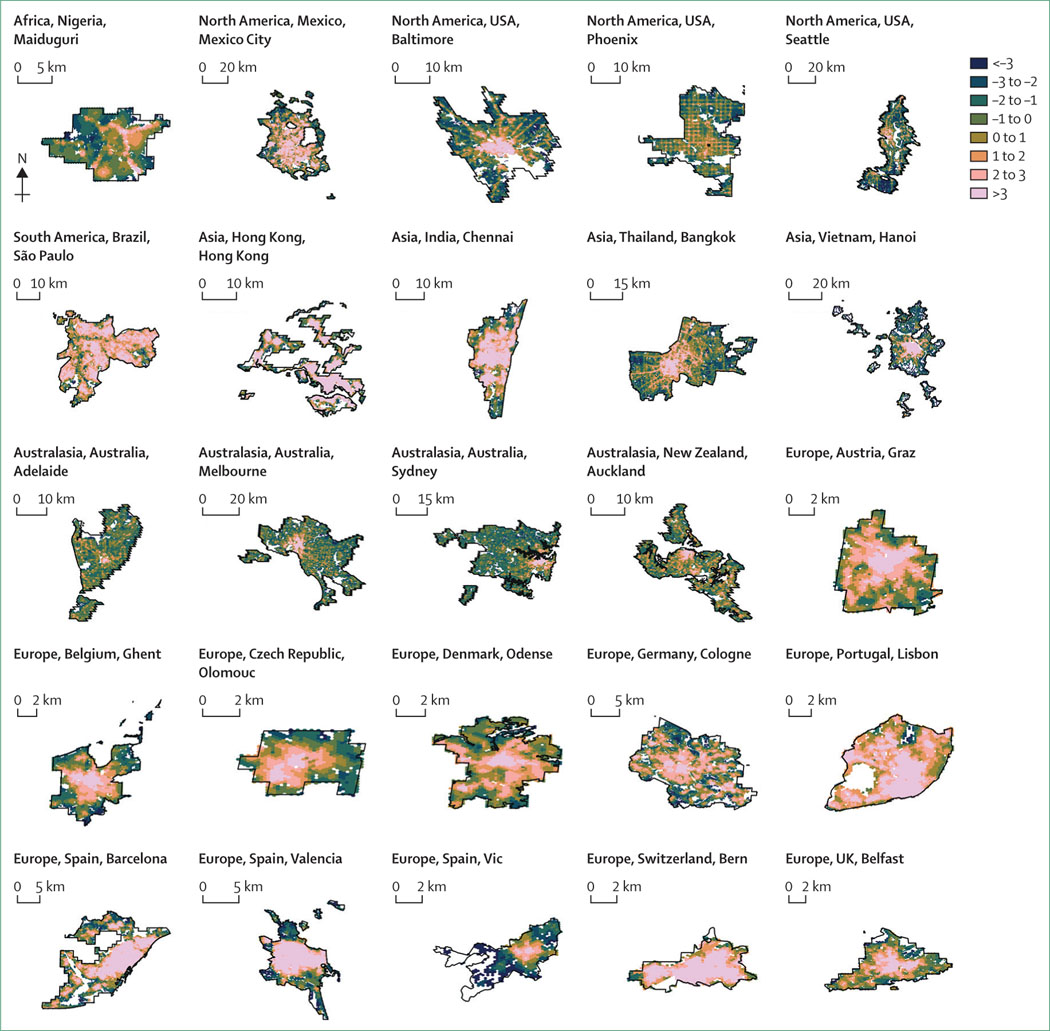
Relative walkability in 25 cities Zero indicates global average. Scale indicates SD.

**Figure 2:**
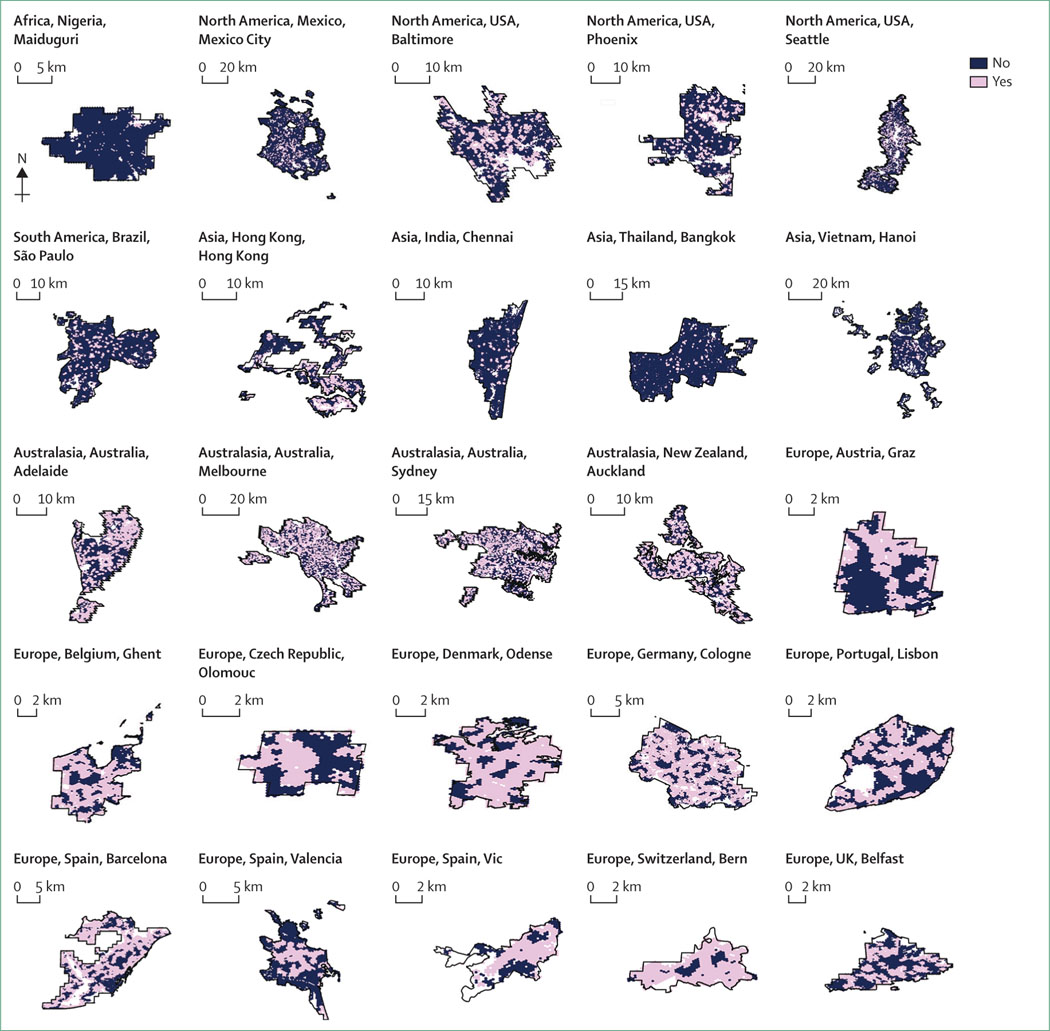
Access to large public open space within 500 m across 25 global cities Access for urban neighbourhoods was considered achieved when at least half of the sampled walkable area was estimated to be located within 500 m of areas identified as public open space 1·5 hectares or larger.

Neighbourhood-level results show spatial patterning and inequities within cities. Figure 1 presents the spatial distribution of urban walkability for the 25 cities. Access to a large public open space within 500 m (figure 2) offers a different conceptualisation of spaces that provide opportunities for physical activity—in addition to health benefits from nature, social connectedness, and heat-island mitigation—and shows different spatial patterns than walkability. Achieving policy goals of access to both walkable neighbourhoods with local destinations and public open space requires evidence-driven city planning to reach a balance and prevent unintended negative consequences for the health and wellbeing of residents.^34–37^

Additional maps and visualisations of the population percentages meeting thresholds to support active and sustainable lifestyles for each city are included in the appendix (pp 24–407). To summarise, although we identified walkable neighbourhoods across all cities, Australasian and US cities in particular exhibited sprawl outside their cores. Most of the cities in middle-income countries and the USA were poorly served by public open space compared with the European and Australasian cities. The spatial distribution data from the project have been made publicly available and provide an opportunity for researchers to conduct their own analyses, which could be supplemented with city-specific covariate data (eg, sociodemographic characteristics or air pollutant distributions) that might not be publicly available globally.

### An indicator framework for better city planning

This study developed an open-source urban indicator computational framework using open-data sources supporting international comparative analyses. We showed applications of this framework by calculating spatial indicators of built environment features for 25 diverse cities. These data and this analytical workflow enabled comparisons of within-city and between-city performance on the indicators. In general, the data were available and useful in all but the lowest-income cities, so broadening participation in crowdsourced data is essential for worldwide efforts to monitor urban indicators.

Many people do not have access to the urban design and transport features needed for healthy and sustainable cities. Our results show that older, compact cities had better walkability, irrespective of economic development status. The worst performing cities for walkability were in high-income countries including the USA, Australia, and New Zealand. These cities were developed primarily in the 20th century under a car-centric planning model.^38^ Lowe and colleagues^21^ similarly found that Australian and US cities were the most likely to have contemporary urban design and transport policies that favour car use over active transport. However, investment in public transport has made it a viable alternative to the car in some Australasian cities,^4^ whereas US cities have fallen behind by global standards. In less-resourced cities, the economic necessity of providing mobility might take precedence over intersectoral planning that protects and promotes healthy lifestyles. Although walking for transport is important, access to public open space is also essential for health and wellbeing,^39^ particularly with a changing climate. Four of the five worst-performing cities for access to public open space were in middle-income countries, joined by the car-dominated city of Phoenix. Inadequate policy frameworks and gaps between policy and implementation probably contributed to unequal access to health-supporting urban design and transport features.^21^ Access inequities revealed by these spatial analyses point to areas requiring policy interventions to reduce health inequities between and within cities. Creating high-quality, fine-grained spatial indicators that incorporate evidence-based targets facilitates comparison not just between cities, but within cities. This evidence in turn provides a foundation for planning future interventions, monitoring policy effects, and harnessing lessons from comparable countries.

For example, São Paulo and Bangkok have similar populations and large proportions of residents in informal settlements. Yet more than 95% of São Paulo’s residents were estimated to have access to frequent public transport, compared with 62% of Bangkok’s residents. More generally, and across all indicators reported, São Paulo outperformed Bangkok. These divergences are best understood by looking not just at the between-city results, but the within-city results. Relative urban walkability for São Paulo was high across most of the urban area, whereas Bangkok achieved high walkability in the central city only (figure 1). In São Paulo, the areas where walkability was lower followed the paths of the two major rivers that pass through the city, adjoined by highways and industrially zoned land. In Bangkok, outside the central city, walkability was achieved only in the areas near the highways that radiate outwards like spokes from the central hub. Between these spokes were the areas with the least walkable access to local amenities. Consistent with our findings, Lowe and colleagues^21^ found that Bangkok had the greatest policy gaps and limitations, whereas São Paulo’s policy framework performed better than those of many cities in high-income countries. The crucial links between transport, land use, and health equity need to be recognised in future iterations and implementations of Bangkok’s city plans, developed under the holistic approach advocated for by Peraphan and Sittha.^40^ This reasoning applies similarly in other countries—including in low-density, car-centric cities in high-income countries, such as the USA and Australia.

#### Call for action

This study showed the feasibility of producing comparative spatial indicators to benchmark cities on urban design and transport features important for public health and sustainability. The workflow for creating the indicators has inherent value, but is most useful if the urban policy and research community uses the open-source framework to continue monitoring cities’ progress towards health and sustainability goals with periodic indicator scorecards. Regional, national, and global agencies can play important roles in incentivising such work, particularly when data collection is required to fill open data gaps. Our open data and open-source methods allow anyone to freely replicate this study. The open-source philosophy posits that communities of research and practice should collectively build and share tools rather than develop individual ad hoc scripts that produce incomparable indicators. A potential benefit of using the methods presented here would be that consistent measures could be created and compared at different points in time with few barriers to participation.

Open data and open-source tools together create an opportunity that, for the first time, enables built environment, health, and policy researchers to quantify and monitor the progress of their city and compare local results across cities globally—if the data and tools are of sufficient quality. Data availability and quality vary both between and within cities, and this is a particular concern when using open data.^41,42^ We therefore developed methods to identify and overcome data limitations through extensive consultation and validation with local collaborators throughout the process.^23^ Data and tools will improve if researchers and practitioners contribute to common methods. We recommend that all cities participate in the open commons, using emerging standard open platforms like OSM to collect and contribute data, and adopt existing, standard open-data platforms with easy access and consistent digitisation standards for local data collection. If the goal is the public good, then open source should be the default for government data and analytics. But governments need not be solely responsible. Open data can be created through three mechanisms: government investment, commercial investment, and crowdsourcing. We encourage collaborations between academia and industry, alongside multilateral efforts to set standards and foster participation in the development and application of indicators. It is imperative that we actively encourage community engagement through crowd-sourcing, and that these indicators are used for planning and advocacy to achieve health and sustainability goals while reducing inequities.^2^

In this article, we described a tool created by an international team and presented results for 25 international cities. We also described a process that starts and finishes with local knowledge: gather data locally; calculate, analyse, and compare indicators globally; and interpret the results locally using local context and knowledge to derive insights, plan interventions, and advocate for reform. As an international team with local collaborators, we identified study areas, developed tools, collected data, ran analyses, and validated results. This collaborative approach lowered the barriers—technical constraints, resource limitations, and costs—to conducting this kind of analysis. Better city planning around the world requires better monitoring by local governments, with an emphasis on local participation, local data, and local use. We have developed this framework, shown its utility, and provided open-source tools to stimulate adoption and creation of common indicators that can be benchmarked and monitored to support healthy and sustainable cities.

To create an international system for monitoring spatial indicators of health, sustainability, and equity, cities should promote the crowdsourcing of data using the current indicators and thresholds as a foundation for global comparisons (panel 2). Cities should provide technical assistance in data collection, analysis, and application. A growing number of data observatories focus on urban SDG indicators, but they tend to ignore spatial and population distributions that enable evidence-based planning for targeted local interventions. As our results show, beyond just a city-level focus, these indicators require a within-city focus to unpack heterogeneity. Maps allow such variations and inequities to be easily seen and understood. Organisations such as the UN and WHO are well positioned to support the expansion of this work into a global observatory of within-city and between-city indicators to promote better city planning that can be used by local decision makers to benchmark and monitor progress.

## Supplementary Material

Supplementary Material

## Figures and Tables

**Table 1:** Mean population percentage estimates for proximal access to amenities

	Estimated population percentage with access within 500 m walking distance to a:							Total emission of PM_2·5_ from the transport sector in 2015 (tonnes per year)
		
	Healthy food market	Convenience store	Public transport stop (OSM or GTFS)	Public transport stop with regular service (30 min)	Public transport stop with regular service (20 min)	Public open space	Public open space >1·5 hectares	

Africa								
Nigeria								
Maiduguri	17·4%	27·4%	9·6%	..	..	1·9%	0·5%	7·5
North America								
Mexico								
Mexico City	26·4%	22·7%	35·8%	24·7%	19·7%	49·6%	19·7%	532·0
USA								
Baltimore	14·3%	29·1%	63·1%	51·3%	42·8%	62·5%	39·2%	324·8
Phoenix	5·6%	21·0%	66·0%	61·6%	24·1%	36·5%	24·6%	268·3
Seattle	15·5%	26·1%	60·3%	36·3%	26·6%	59·2%	35·0%	316·3
South America								
Brazil								
São Paulo	35·2%	36·7%	96·1%	95·7%	94·2%	71·7%	15·5%	2306·5
Asia								
Hong Kong								
Hong Kong	48·9%	52·2%	89·5%	86·9%	83·6%	86·9%	54·1%	1903·7
India								
Chennai	19·7%	15·6%	39·1%	3·2%	3·2%	41·1%	11·3%	657·9
Thailand								
Bangkok	15·3%	33·4%	63·0%	62·1%	43·2%	14·1%	6·5%	4163·9
Vietnam								
Hanoi	38·0%	46·4%	65·5%	21·9%	11·2%	26·7%	14·1%	2062·8
Australasia								
Australia								
Adelaide	18·8%	19·9%	89·2%	81·9%	53·7%	87·3%	58·0%	147·4
Melbourne	20·7%	29·6%	86·7%	67·2%	49·4%	88·2%	63·3%	1364·0
Sydney	22·3%	28·7%	94·7%	78·4%	57·7%	90·1%	60·3%	564·8
New Zealand								
Auckland	31·2%	47·9%	91·0%	81·4%	55·7%	90·6%	64·9%	340·3
Europe								
Austria								
Graz	62·6%	56·4%	92·2%	..	..	84·9%	39·5%	14·4
Belgium								
Ghent	49·5%	44·1%	86·5%	..	..	92·7%	62·7%	47·4
Czech Republic								
Olomouc	37·2%	43·7%	88·8%	..	..	90·4%	46·0%	2·6
Denmark								
Odense	43·7%	36·1%	84·4%	66·1%	59·0%	92·9%	73·4%	3·9
Germany								
Cologne	51·1%	56·9%	79·0%	71·7%	60·2%	89·6%	65·8%	158·9
Portugal								
Lisbon	64·2%	60·7%	97·0%	95·7%	92·8%	90·1%	51·3%	208·1
Spain								
Barcelona	63·8%	61·6%	91·4%	78·3%	75·8%	88·2%	62·8%	186·1
Valencia	59·7%	48·4%	81·6%	78·3%	77·2%	78·4%	43·8%	105·0
Vic	50·7%	40·1%	57·7%	..	..	81·4%	74·8%	..
Switzerland								
Bern	69·3%	73·5%	94·8%	93·6%	91·8%	98·9%	80·0%	10·3
UK								
Belfast	29·0%	47·8%	92·9%	82·9%	72·6%	65·0%	46·8%	29·4
Middle-income, mean (SD)	25·3% (9·5)	30·4% (10·9)	34·3% (36·7)	34·2% (25·3)	11·3% (6·9)	51·5% (29·9)	41·5% (37·0)	1621·8 (1539·4)
High-income, mean (SD)	39·9% (19·9)	43·3% (15·0)	61·5% (21·0)	81·8% (15·4)	55·1% (14·7)	83·5% (12·4)	74·1% (15·8)	333·1 (496·9)
Total, mean (SD)	36·4% (18·9)	40·2% (15·0)	54·7% (27·5)	70·4% (27·2)	44·6% (23·2)	75·8% (22·3)	66·0% (26·1)	655·3 (1014·3)

OSM=OpenStreetMap. GTFS=general transit feed specification.

**Table2:** Proportion of population living in neighbourhoods reaching or exceeding thresholds for spatial indicators that support physical activity goals

	Population density threshold		Street intersection density threshold	
	Scenario A (95% CI 4790–6750)	Scenario B (95% CI 5677–7823)	Scenario A (95% CI 90–110)	Scenario B (95% CI 106–156)

Africa				
Nigeria				
Maiduguri	98·0%	95·9%	45·6%	28·5%
North America				
Mexico				
Mexico City	98·9%	98·1%	89·6%	78·6%
USA				
Baltimore	39·6%	28·0%	64·8%	51·7%
Phoenix	30·1%	15·7%	74·4%	51·0%
Seattle	10·9%	6·4%	61·3%	43·2%
South America				
Brazil				
São Paulo	99·6%	99·4%	88·0%	70·4%
Asia				
Hong Kong				
Hong Kong	98·3%	97·7%	95·7%	91·5%
India				
Chennai	99·8%	99·7%	90·4%	79·3%
Thailand				
Bangkok	98·2%	97·0%	61·5%	39·7%
Vietnam				
Hanoi	95·7%	93·0%	67·6%	56·3%
Australasia				
Australia				
Adelaide	3·7%	0·0%	38·4%	12·6%
Melbourne	33·4%	17·8%	37·8%	20·8%
Sydney	67·5%	51·0%	24·9%	13·4%
New Zealand				
Auckland	47·9%	22·3%	26·6%	14·5%
Europe				
Austria				
Graz	64·0%	44·1%	92·5%	81·3%
Belgium				
Ghent	0·0%	0·0%	67·5%	54·8%
Czech Republic				
Olomouc	0·0%	0·0%	69·0%	54·2%
Denmark				
Odense	6·0%	0·0%	94·8%	85·3%
Germany				
Cologne	47·5%	21·6%	83·9%	71·6%
Portugal				
Lisbon	98·1%	96·8%	99·7%	98·6%
Spain				
Barcelona	95·5%	92·3%	82·6%	74·9%
Valencia	97·8%	95·8%	78·6%	72·3%
Vic	47·1%	24·3%	65·3%	56·4%
Switzerland				
Bern	82·1%	58·3%	99·3%	98·2%
UK				
Belfast	59·6%	40·1%	91·1%	74·0%
Middle-income, mean (SD)	98·4% (1·5)	97·2% (2·5)	73·8% (18·5)	58·8% (21·1)
High-income, mean (SD)	48·9% (35·0)	37·5% (35·3)	71·0% (24·1)	59·0% (28·1)
Total, mean (SD)	60·8% (37–2)	51·8% (40·2)	71·6% (22·6)	58·9% (26·2)

Scenario A is defined as meeting the threshold for 80% probability of walking for transport, and scenario B is defined as meeting the threshold for reaching WHO’s target of more than or equal to a 15% relative reduction in insufficient physical activity through walking.

## Panel 1: Limitations of current guidelines for developing consistent spatial indicators

There are various guidelines for developing health and sustainability indicators, but each has limitations. The International Organisation for Standardisation (ISO) is an independent, non-governmental organisation established in 1946 to develop consensus-based, market-relevant international standards to help solve global challenges. The ISO37120 standard proposes indicators for city services and quality of life that can track and monitor city performance over time.^5^ ISO advocates for standardised indicators to provide a uniform approach to what is measured and how that measurement is done. However, ISO’s framework stops short of recommending targets, and it does not deal with the difficulties involved in sourcing necessary (and consistent) data. Further, the indicators tend to be citywide rather than neighbourhood-scale, limiting capacity for within-city comparisons.

For example, the ISO indicator for public transport access (19·6—percentage of population living within 0·5 km of public transit running at least every 20 min during peak periods) suggests a single citywide measure and does not stipulate potential data sources or a target for the percentage of the population served at this level. The indicator makes no reference to the spatial boundary used to determine the population catchment, nor does it stipulate whether the distance threshold for evaluating access should be measured using Euclidean distance or transport network traversal. Depending on the network’s connectivity, Euclidean distance often overestimates access. Thus, two analysts independently measuring the same city at the same time might generate substantially different results.

The UN Habitat’s New Urban Agenda Monitoring Framework is more comprehensive and addresses barriers to consistently measure and compare cities.^6^ Taking a similar public transport indicator as an example (proportion of the population that has convenient access to public transport disaggregated by age group, sex, and people with disabilities), UN Habitat provides seven pages of guidance on how the indicator should be constructed and possible data sources. However, it only suggests a citywide measure, and does not provide targets.

## Panel 2: Global call for action

We urge the UN and WHO to:

- Incentivise and promote open data and open-source tools in the pursuit of meeting health, sustainability, and equity goals
- Support expansion of our indicators into a global observatory of within-city and between-city indicators
- Provide practical guidance on identifying barriers to walking and wheelchair access

We urge local, regional, and national governments to:

- Use the open-source framework we presented to create consistent measures that can inform policy and monitor cities’ progress towards health and sustainability goals, using indicator scorecards to provide regular feedback and prompt targeted interventions
- Involve local populations in crowdsourcing data about their own communities
- Provide resources to fill gaps of missing data
- Map barriers to walking access to refine indicators
- Work together with academics and share best practices
- Use open commons and emerging standard open platforms such as OpenStreetMap to collect and contribute data

We urge scientific societies to:

- Host city indicators for continual monitoring and comparison
- Provide essential resources, expertise, and tools to ensure longevity of the hosting platform
- Train students and involve local populations as citizen scientists to collect crowdsourced data about their own communities
