# Supplementary material for: Using open data and open-source software to develop spatial indicators of urban design and transport features for achieving healthy and sustainable cities
Source: Lancet Glob Health. Author manuscript; Available in PMC 2023 Mar 7. (PMC9902524; doi:10.1016/S2214-109X(22)00072-9)
Supplement: Supplementary Material [file NIHMS1859903-supplement-Supplementary_Material.pdf]

THE LANCET
Global Health

Supplementary appendix

This appendix formed part of the original submission and has been peer reviewed. We post it as supplied by the authors.

Supplement to: Boeing G, Higgs C, Liu S, et al. Using open data and open-source software to develop spatial indicators of urban design and transport features for achieving healthy and sustainable cities. *Lancet Glob Health* 2022; **10:** e907–18.

Using open data and open-source software to develop spatial indicators of urban design and transport features for achieving healthy and sustainable cities: a 25-city study

Technical and Map Appendices

Global Healthy & Sustainable City-Indicators Collaboration, 2022

Appendix A: Technical documentation

Contents

APPENDIX A 2
A1. Preliminaries 3
A1.1. Spatial data collection 3
A1.2. Defining study regions 6
A1.3. Processing administrative boundaries 6
A1.3.1. General methods 6
A1.3.2. City-specific methods 6
A2. Reproducible analytic workflow for estimating indicators 9
A2.1. Data acquisition 9
A2.2. Setup configuration 14
A2.3. Pre-processing input data 14
A2.3.1. Points of interest 14
A2.3.2. Public transport stops with regular daytime weekday service 16
A2.3.3. Public open space 19
A2.4. Processing sampling estimates, and aggregating indicators 21
A3. Mapping 23
References 23

A1. Preliminaries

A1.1. Spatial data collection

Collaborators from 19 countries (Table A) in the International Physical Activity and Environment Network (IPEN, https://www.ipenproject.org/) and other collaborators expressed interest in the Global Healthy and Sustainable City-Indicators Collaboration. They nominated local representatives with spatial expertise to complete a Google Forms survey (Table B) detailing available resources for representing their region in the study.

Seventeen cities had available street network data, with datasets publicly available in 15 cities. However, only six cities had publicly available data on urban design and transport features of interest, with commercially licensed data being more commonplace. Some datasets were provided by local collaborators during the survey, but they were inconsistent in terms of availability, formats, comparability, and coverage. Thus, for consistency we decided to use open data with global coverage in the first instance.

This ‘open data first’ approach meant that we were able to expand our analysis to include the full set of indicators for all 25 cities. For sources of open data for each studied city, see Appendix A2·1. The limitations of open data were identified through consultation with collaborators throughout the indicator estimation workflow, and we developed methods to mitigate these limitations where required by incorporating custom data and methods to improve the validity of our analysis. Details on the data validation process were published in the software framework method paper.[1]

Due to the lack of detailed information on street networks and walkability barriers, we recognise two important assumptions when measuring the considered walkable distances: 1) streets do not present barriers to walking or wheelchair access; and 2) people’s walking ranges are higher.

Table A. Study region characteristics

	City	Urban study region area (km²)	Population estimate (2015)	Population per km²	GNI per capita classification (2021)	Sample points analysed	Sample points analysed per km²
**Africa**							
Nigeria (NGA)	Maiduguri	125	1,092,447	8,722	Lower middle	62,358	498
**America, North**							
Mexico (MEX)	Mexico City	2,312	20,216,501	8,744	Upper middle	1,516,235	656
United States (USA)	Baltimore	741	1,381,445	1,865	High	429,005	579
	Phoenix	772	1,320,016	1,710		466,658	605
	Seattle	1,885	2,199,327	1,167		1,038,975	551
**America, South**							
Brazil (BRA)	São Paulo	1,018	11,718,034	11,512	Upper middle	676,239	664
**Asia**							
Hong Kong SAR (HKG)	Hong Kong	373	7,325,576	19,665	High	269,800	724
India (IND)	Chennai	425	6,602,769	15,549	Lower middle	276,913	652
Thailand (THA)	Bangkok	1,190	9,337,076	7,844	Upper middle	695,113	584
Vietnam (VNM)	Hanoi	1,220	5,938,818	4,866	Lower middle	375,743	308
**Australasia**							
Australia (AUS)	Adelaide	541	985,647	1,822	High	307,819	569
	Melbourne	1,657	3,741,467	2,258		929,061	561
	Sydney	1,334	4,082,229	3,061		676,664	507
New Zealand (NZL)	Auckland	468	1,234,554	2,638	High	232,260	496
**Europe**							
Austria (AUT)	Graz	69	283,101	4,121	High	72,894	1,061
Belgium (BEL)	Ghent	75	174,411	2,339	High	50,852	682
Czech Republic (CZE)	Olomouc	27	88,044	3,275	High	26,775	996
Denmark (DNM)	Odense	56	157,018	2,791	High	49,196	874
Germany (DEU)	Cologne	348	1,118,442	3,218	High	225,412	648
Portugal (PRT)	Lisbon	85	583,347	6,867	High	79,941	941
Spain (ESP)	Barcelona	359	3,259,527	9,068	High	262,708	731
	Valencia	86	682,752	7,937		76,410	888
	Vic	31	43,813	1,433		11,499	376
Switzerland (CHE)	Bern	32	158,179	4,898	High	46,801	1,449
Northern Ireland (GBR)	Belfast	98	400,731	4,084	High	67,244	685
**Summary**							
	**min**	27	43,813	1,167		11,499	308
	**25th percentile**	85	400,731	2,339		67,244	561
	**median**	373	1,234,554	4,084		262,708	652
	**75th percentile**	1,018	4,082,229	7,937		466,658	731
	**max**	2,312	20,216,501	19,665		1,516,235	1,449
	**interquartile range**	933	3,681,497	5,599		399,414	170
	**mean**	613	3,365,011	5,658		356,903	691
	**sd**	654	4,736,826	4,643		379,168	240

Table B. Spatial data survey items

#	Item
1	Timestamp
2	Email address
3	Name
4	What is the city for which you are uploading data?
5	Country
6	City boundary data (study region)
7	Is an alternative routable street network data set to OpenStreetMap preferred for indicator calculation in your city? If yes: file upload; Data source; URL or citation; Reference date; Spatial reference EPSG code; Licence type (e.g. "CC BY 4.0", "none specified", etc).; Please detail any restrictions, limitations or other important points to note on the use of this data in this project
8	*Do you have access to General Transit Feed Specification (GTFS) data for your city? If yes: file upload; Data source(s); URL(s) or citation(s); Reference date(s); License type (e.g. "CC BY 4.0", "none specified", etc).; Please detail any restrictions, limitations or other important points to note on the use of this data in this project*
9	Are alternative or supplementary Points of Interest (POIs) / destinations / activity centre data preferred for indicator calculation in your city, instead of or in addition to those sourced from OpenStreetMap?
10	*Population per city area table. If available: file upload; Data source; URL or citation; Reference date; License type (e.g. "CC BY 4.0", "none specified", etc).; Please detail any restrictions, limitations or other important points to note on the use of this data*
11	Area boundaries. If available: file upload; Attribute column used for linkage with the population per area CSV; Data source; URL or citation; Reference date; Spatial reference EPSG code; License type (e.g. "CC BY 4.0", "none specified", etc).; Please detail any restrictions, limitations or other important points to note on the use of this data in this project
12	*Please upload a CSV or Excel file with the area ID (or name) and count of dwellings. If available: file upload; Data source; URL or citation; Reference date; License type (e.g. "CC BY 4.0", "none specified", etc).; Please detail any restrictions, limitations or other important points to note on the use of this data*
13	Are the area boundaries associated with dwelling counts the same as those previously uploaded for population? If yes: file upload; Area boundaries; Attribute column used for linkage with the dwellings per area CSV; Data source; URL or citation; Reference date; Spatial reference EPSG code; License type (e.g. "CC BY 4.0", "none specified", etc).; Please detail any restrictions, limitations or other important points to note on the use of this data in this project
14	*Please upload a CSV or Excel file with the area ID (or name) of usual residence and usual place of work and joint counts. If available: file upload; Data source; URL or citation; Reference date; License type (e.g. "CC BY 4.0", "none specified", etc).; Please detail any restrictions, limitations or other important points to note on the use of this data*
15	Are the area boundaries associated with journey to work counts the same as those previously uploaded for population or dwellings? If no: file upload; Attribute column used for linkage with the journey to work CSV; Data source; URL or citation; Reference date; Spatial reference EPSG code; License type (e.g. "CC BY 4.0", "none specified", etc).; Please detail any restrictions, limitations or other important points to note on the use of this data in this project; Can you provide Digital Elevation Model (DEM) data for your study region superior to that which will be made available under NASADEM as described at https://earthdata.nasa.gov/community/community-data-system-programs/measures-projects/nasadem ?
16	*Can you provide Digital Elevation Model (DEM) data for your study region superior to that which will be made available under NASADEM as described at https://earthdata.nasa.gov/community/community-data-system-programs/measures-projects/nasadem? If yes: file upload; Data source; URL or citation; Reference date; Spatial reference EPSG code; License type (e.g. "CC BY 4.0", "none specified", etc).; Please detail any restrictions, limitations or other important points to note on the use of this data in this project; Do you have an open source residential address data set available for your city which is appropriate for use in this project?; Residential address points*
17	Do you have an open source residential address data set available for your city which is appropriate for use in this project? If yes: file upload; Data source; URL or citation; Reference date; Spatial reference EPSG code; License type (e.g. "CC BY 4.0", "none specified", etc).; Please detail any restrictions, limitations or other important points to note on the use of this data in this project; Do you have a source of open space / green space data to be preferred over use of OpenStreetMap data?; Open Space data
18	*Do you have a source of open space / green space data to be preferred over use of OpenStreetMap data? If yes: file upload; Definition of open space; Data source; URL or citation; Reference date; Spatial reference EPSG code; License type (e.g. "CC BY 4.0", "none specified", etc).; Please detail any restrictions, limitations or other important points to note on the use of this data in this project*

A1.2. Defining study regions

The analysis area for each city included in our study was conceptually framed as the urban portion of a city's broad administrative boundaries. Administrative boundaries were either supplied by collaborators via the preliminary spatial data survey or acquired by the researchers independently. The urban study region was defined as the intersection of this boundary with that of corresponding urban centres identified by the Global Human Settlements project (UCDB R2019A v1·2).[2] The use of this global data source for urban centres helps to ensure that the analysis focuses on exposure for urban populations across all cities, but not for lower-density rural settings on the urban fringe, which may otherwise fall within an administrative boundary.

A1.3. Processing administrative boundaries

A curated dataset of study region administrative boundaries was manually prepared. Administrative boundaries provided by collaborators for their city were used in the first instance, with alternative open-data sources for boundaries identified for the remaining cities. Boundaries for some cities were revised through consultation with collaborators following review of the preliminary data validation reports generated in the pre-processing phase. The final city-specific boundary methods are detailed below.

A1.3.1 General methods

Where city boundaries were acquired as a single valid feature, these were output in the relevant projected CRS for that region to a geopackage file. City boundaries supplied as a series of administrative polygons (e.g. Belfast) were dissolved using QGIS 3·8·1-Zanzibar.[3] Any gaps that incorrectly remained were removed using the "delete holes" tool, and saved to the geopackage file. For cities where boundaries were either not supplied (e.g., Auckland) or not supplied in a usable format (Hanoi, as lines and points—not areas) alternative boundaries were sourced. For cities with geometry type of PolygonZ (Ghent, Odense), these were exported as a regular polygon geometry, discarding Z-dimension data, which may cause difficulties with subsequent processing of multipolygons.

A1.3.2 City-specific methods

GHS urban boundaries: Baltimore, Seattle (United States); Maiduguri (Nigeria)

It was decided that for these cities the best solution for study region boundaries was to use the urban region as defined in the Global Human Settlements dataset (UCDB R2019A v1·2). It was decided not to do this for all regions, as a number of collaborators indicated that the predicted urban centre as defined in the GHS dataset did not reliably include urban-fringe growth areas, which were considered important (e.g. Australian cities, Mexico City). As such, where boundaries were approved by collaborators, these existing ones were used. Elsewhere, on approval from collaborators, the GHS urban centre was used.

Auckland, New Zealand

New Zealand administrative boundaries (statsnzmeshblock-higher-geographies-2019-generalised-SHP.zip) were retrieved from the Stats NZ geographic data portal (https://datafinder.stats.govt.nz/data/category/annual-boundaries/2019/). The Auckland city boundary was processed by selecting the Mesh Blocks with UR_2019_V_1 value of 'Auckland' and with IUR2019__1 classed as major urban. The boundaries were dissolved and saved to the boundaries geopackage.

Barcelona, Spain

The initial identified boundary was revised using a collaborator-supplied shape file following preliminary data validation. Boundaries were dissolved and exported following the general method.

Bern, Switzerland

A boundary zip file was retrieved from the Geoportal of the Canton of Bern (https://www.geo.apps.be.ch/index.php?option=com_easysdi_shop&task=download.direct&id=195). Using the shapefile "GENGRZ5_GEN2G5", the feature of the name "Bern" was exported as Bern to the boundaries geopackage.

Chennai, India
An administrative boundary for Chennai from OpenStreetMap was retrieved in geojson format (https://nominatim.openstreetmap.org/search.php?q=Chennai+India&polygon_geojson=1&format=geojson). The QGIS delete holes processing tool was used to include a gap identified as representing the St Thomas Mount cantonment (http://www.cbstm.org.in:9080/Cantonment/web/Home.html) in the Chennai urban study region. The derived Chennai boundary was then exported to the boundaries geopackage.

Cologne, Germany
The boundary was retrieved from the Köln (Cologne) open-data portal (https://offenedaten-koeln.de/dataset/stadtteile). The 'Fix geometries'' tool was run, then the Dissolve algorithm, the ''Delete holes'' algorithm, and finally was exported as Cologne to the boundaries geopackage.

Ghent, Belgium
A boundary for the city of Ghent omitting the North Sea port industrial area was supplied by collaborators, following the advice received in the preliminary data survey that this portion of the city should be excluded from consideration as 'urban'. Boundaries were dissolved and exported following the general method.

Graz, Austria
Boundaries were retrieved from the Austrian government open-data portal (https://www.data.gv.at/katalog/dataset/land-stmk_bezirksgrenzen/resource/086b453d-c474-4458-ba31-2b5408f5b999). The field BEZNAM with value 'Graz Stadt' was exported as Graz to the boundaries geopackage.

Hanoi, Vietnam
Administrative boundaries for Vietnam (vnm_admbnda_2018_shp.zip) were retrieved from Humanitarian Data Exchange (https://data.humdata.org/dataset/viet-nam-administrative-boundaries-polygon-polylinev), and the level 1 boundary layer (vnm_admbnda_adm1_2018_v2) feature corresponding to ADM1_EN value of 'Ha Noi' was saved to the boundaries geopackage.

Hong Kong SAR, China
A boundary was retrieved from ESRI China (http://opendata.esrichina.hk/datasets/hong-kong-18-districts/data). The 'fix geometries' algorithm was used to resolve validity issues, boundaries were dissolved, and 'delete holes' was run prior to saving to the curated geopackage.

Mexico City, Mexico
Administrative boundary data for Mexico City were retrieved from https://data.humdata.org/dataset/mexican-administrative-level-0-country-1-estado-and-2-municipio-boundary-polygons. Following advice from collaborators, the Mexico City boundary was constructed to reflect Greater Mexico City, in the sense of Zona Metropolitana del Valle de México (https://en.wikipedia.org/wiki/Greater_Mexico_City#Metropolitan_Area_of_the_Valley_of_Mexico). This involved the combination of: 16 municipalities (alcaldías) of Ciudad de México (Azcapotzalco, Álvaro Obregón, Benito Juárez, Coyoacán, Cuajimalpa de Morelos, Cuauhtémoc, Gustavo A. Madero, Iztacalco, Iztapalapa, Magdalena Contreras, Miguel Hidalgo, Milpa Alta, Tláhuac, Tlalpan, Venustiano Carranza, Xochimilco); the 40 municipalities of the State of Mexico (Acolman, Atenco, Atizapán de Zaragoza, Chalco, Chiautla, Chicoloapan, Chiconcuac, Chimalhuacán, Coacalco de Berriozábal, Cocotitlán, Coyotepec, Cuautitlán, Cuautitlán Izcalli, Ecatepec de Morelos, Huehuetoca, Huixquilucan, Ixtapaluca, Jaltenco, La Paz, Melchor Ocampo, Naucalpan de Juárez, Nextlalplan, Nezahualcoyotl, Nicolás Romero, Papalotla, San Martín de las Pirámides, Tecámac, Temamatla, Teoloyucán, Teotihuacán, Tepetlaoxtoc, Tepotzotlán, Texcoco, Tezoyuca, Tlamanalco, Tlalnepantla de Baz, Tultepec, Tultitlán, Valle de Chalco Solidaridad, Zumpango); one conurbation municipality of the State of Hidalgo (Tizayuca); and additional municipalities (Amecameca, Apaxco, Atlautla, Axapusco, Ayapango, Ecatzingo, Hueypoxtla, Isidro Fabela, Jilotzingo, Juchitepec, Nopaltepec, Otumba, Ozumba, Temascalapa, Tenango del Aire, Tepetlixpa, Tequixquiac, Villa del Carbón), and Tonanitla.

Noting that some of the ADM2_ES names in the source data were not unique within the country of Mexico, the ADM1_ES state names were limited to ''Distrito Federal'', ''Hidalgo'' and ''Mexico''. The required zones were

identified using the query, which involved manually amending names to match their recorded representation in the source data (i.e. some missing characters, truncation, and switched characters). Matched provinces were verified to be in their correct location using a subsequent audit.

ADM2_ES IN (‘Azcapotzalco’, ‘Álvaro Obregón’, ‘Benito Julrez’, ‘Coyoacán’, ‘Cuajimalpa de Morelos’, ‘Cuauhtémoc’, ‘Gustavo A. Madero’, ‘Iztacalco’, ‘Iztapalapa’, ‘La Magdalena Contreras’, ‘Miguel Hidalgo’, ‘Milpa Alta’, ‘Tláhuac’, ‘Tlalpan’, ‘Venustiano Carranza’, ‘Xochimilco’, ‘Acolman’, ‘Atenco’, ‘Atizapán de Zaragoza’, ‘Chalco’, ‘Chiautla’, ‘Chicoloapan’, ‘Chiconcuac’, ‘Chimalhuacán’, ‘Coacalco de Berrioz’, ‘Cocotitlán’, ‘Coyotepec’, ‘Cuautitlán’, ‘Cuautitlán Izcalli’, ‘Ecatepec de Morelos’, ‘Huehuetoca’, ‘Huixquilucan’, ‘Ixtapaluca’, ‘Jaltenco’, ‘La Paz’, ‘Melchor Ocampo’, ‘Naucalpan de JuIrez’, ‘Nextlalpan’, ‘Nezahualcoyotl’, ‘Nicolts Romero’, ‘Papalotla’, ‘San Martín de las Pirámides’, ‘Tec’, ‘Temamatla’, ‘Teoloyucan’, ‘Teotihuacan’, ‘Tepetlaoxtoc’, ‘Tepotzotlán’, ‘Texcoco’, ‘Tezoyuca’, ‘Tlalmanalco’, ‘Tlalnepantla de Baz’, ‘Tultepec’, ‘Tultitlán’, ‘Valle de Chalco Solidaridad’, ‘Zumpango’, ‘Tizayuca’, ‘Amecameca’, ‘Apaxco’, ‘Atlautla’, ‘Axapusco’, ‘Ayapango’, ‘Ecatzingo’, ‘Hueypoxtla’, ‘Isidro Fabela’, ‘Jilotzingo’, ‘Juchitepec’, ‘Nopaltepec’, ‘Otumba’, ‘Ozumba’, ‘Temascalapa’, ‘Tenango del Aire’, ‘Tepetlixpa’, ‘Tequixquiac’, ‘Villa del Carb’, ‘Tonanitla’) AND ADM1_ES IN (‘DISTRITO FEDERAL’, ‘HIDALGO’, ‘MEXICO’)

The final query returned 76 municipalities correctly representing the de facto extent of Greater Mexico City. The retrieved polygons’ boundaries were dissolved, reprojected to EPSG 32614 (UTM14N), and saved to the boundaries.gpkg file as the layer ‘Mexico City’.

Phoenix, United States

A boundary shapefile was retrieved from a US open-data portal (https://catalog.data.gov/dataset/tiger-line-shapefile-2015-state-arizona-current-place-state-based), and the feature with the name of ‘Phoenix City’ was exported as Phoenix to boundaries geopackage.

São Paulo, Brazil

Administrative boundaries for Brazil were retrieved from Humanitarian Data Exchange (https://data.humdata.org/dataset/brazil-administrative-level-0-boundaries), and the feature with ‘name_2’ value of ‘Sao Paolo’ was saved to the boundaries geopackage.

A2. Reproducible analytic workflow for estimating indicators

Following the preliminaries stage, we developed a generalised workflow for calculating within- and between-city indicator estimates. The following supplementary materials elaborate on how our open-source framework was applied to the analysis of the 25 cities presented in this study. This material is intended to provide guidance for researchers and practitioners seeking to reproduce our results or extend them for different study regions and time points.

Figure A below illustrates the general workflow of the framework, which comprises a four-step process from data acquisition to indicator aggregation. Researchers and practitioners seeking to reproduce the indicator results for their cities are advised to look at the open-source framework repository at https://github.com/global-healthy-liveable-cities/global-indicators.git, and then consult the following details for calculating indicators.

Figure A. Indicator calculation workflow

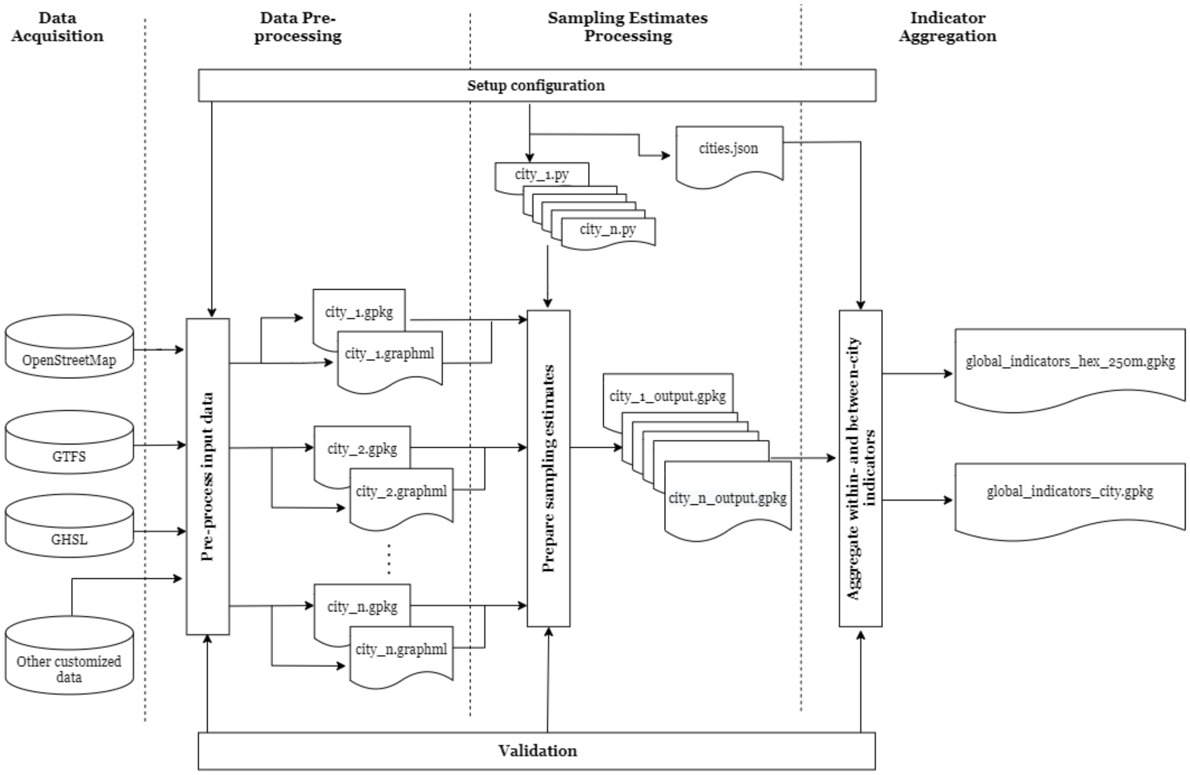

A2.1. Data acquisition

Other than the study region boundaries described in the preliminaries, most data were sourced from open datasets with global scope. These global open-data sources are summarised in Table C. The other exceptions were a set of custom destinations developed by collaborators for Maiduguri, and public transport schedule datasets in General Transit Feed Specification (GTFS) format developed by specific agencies, many of which were hosted on OpenMobilityData. Data sources of GTFS feeds available for the studied cities are summarised in Table D.

Table C. Input data sources for the 25 study regions

Input data	Source	URL	Comments
Study region boundaries	Global Human Settlements Layer Urban Centers Database, UCDB R2019A	https://jeodpp.jrc.ec.europa.eu/ftp/jrc-opendata/GHSL/GHS_STAT_UCDB2015MT_GLOBE_R2019A/	Data were directly downloaded from the GHSL database.
Population	Global Human Settlements Layer, population distribution raster data, GHS-POP R2019A	https://cidportal.jrc.ec.europa.eu/ftp/jrc-opendata/GHSL/GHS_POP_MT_GLOBE_R2019A/	Data were directly downloaded from the GHSL database.
Transport-sector PM2.5 emissions (tonnes per annum)	Global Human Settlements Layer, GHS UCDB 2015	https://cidportal.jrc.ec.europa.eu/ftp/jrc-opendata/GHSL/GHS_POP_MT_GLOBE_R2019A/	Data were directly downloaded from the GHSL database. Note that "transport-sector" encompasses road transport as well as aviation and shipping.
Pedestrian street network	OpenStreetMap planet archive file dated 3 August 2020	https://planet.osm.org/pbf/planet-200803.osm.pbf	Data were retrieved from OpenStreetMap via the Overpass API for buffered boundaries of study regions using the Python package OSMnx.
Healthy food outlets, supermarkets, and convenience stores	OpenStreetMap	https://taginfo.openstreetmap.org/	These spatial features were obtained following the OpenStreetMap tagging guidelines and collaborator feedback, along with the OpenStreetMap Taginfo for the appropriate key-value pair tag, detailed in Table G.
Public transportation	OpenStreetMap	https://transitfeeds.com/; https://taginfo.openstreetmap.org/	Data were obtained from OpenStreetMap following the tagging guidelines and collaborator feedback detailed in Table G, and from various data sources for GTFS format. A more comprehensive list of GTFS data sources is shown in Table D.
Public open space	OpenStreetMap	https://taginfo.openstreetmap.org/	Data were obtained from OpenStreetMap following the tagging guidelines and collaborator feedback detailed in Appendix A 2.3.3.

Table D. General Transit Feed Specification (GTFS) data sources

Country	City	Feed #	Agency / Provider	Year	Analysis period (yyyymmdd)	
					Start	End
Mexico	Mexico City	1	FederalDistrictGovernment*	2019	20190405	20190605
United States	Baltimore	1	MarylandMTA*	2019	20190405	20190605
United States	Phoenix	1	Valleymetro*	2019	20190405	20190605
United States	Seattle	1	KingCountyMetro*	2019	20190405	20190605
Brazil	São Paulo	1	SPTrans*	2019	20191008	20191205
Hong Kong SAR	Hong Kong	1	data.gov.hk	2019	20190405	20190605
India	Chennai	1	MTC	2010	20100405	20100605
India	Chennai	2	J Kishore Kumar	2016	20161008	20161205
Thailand	Bangkok	1	Namtang Open Data	2021	20210405	20210605
Vietnam	Hanoi	1,2,3	World Bank	2018	20180405	20180605
Australia	Adelaide	1	AdelaideMetro*	2019	20191008	20191205
Australia	Melbourne	1	PublicTransportVictoria*	2019	20191008	20191205
Australia	Sydney	1	Transport for NSW	2019	20191008	20191205
New Zealand	Auckland	1	AucklandTransport*	2019	20191008	20191205
Denmark	Odense	1	Rejseplanen*	2019	20190405	20190605
Germany	Cologne	1	VRS*	2018	20180405	20180605
Portugal	Lisbon	1	Carris*	2019	20190405	20190605
Portugal	Lisbon	2	Metro de lisboa*	2019	20190405	20190605
Portugal	Lisbon	3	Fertagus*	2019	20190405	20190605
Portugal	Lisbon	4	MTS*	2019	20190405	20190605
Portugal	Lisbon	5	Soflusa*	2019	20190405	20190605
Portugal	Lisbon	6	Transtejo*	2019	20190405	20190605
Portugal	Lisbon	7	CP*	2019	20190405	20190605
Spain	Barcelona	1	AMB*	2019	20190405	20190605
Spain	Barcelona	2	TMB*	2019	20190405	20190605
Spain	Barcelona	3	TMB*	2019	20190405	20190605
Spain	Valencia	1	MetroValencia*	2019	20190405	20190605
Spain	Valencia	2	EMT*	2019	20190405	20190605
Switzerland	Bern	1	opentransportdata.swiss	2019	20190405	20190605
UK	Belfast	1	Translink	2017	20170405	20170605

* Data sourced from GTFS feed aggregator OpenMobilityData (https://transitfeeds.com)

Table E. Project parameters

Parameters		Description	Value
Project settings	folderPath	The folder where data resources for the project are located	/home/jovyan/work/process/data
	units	Units used by the coordinate reference system	m
	units_full	Full name for the units	metres
	study_buffer	Study region buffer, to account for edge effects	1600
	buffered_study_region_name	Buffered study region's name for map display purposes	1600 m study region buffer
	hex_diag	Hexagon diagonal length and buffer distance (metres)	250
	hex_buffer	Hexagon buffer distance, to account for edge effects	250
	multiprocessing	Number of processors to use in multiprocessing scripts	6
	no_forward_edge_issues	Used to flag and mitigate potential geometry discrepancies	0
	year	The year being targeted by this analysis	2020
SQL	admin_db	SQL settings to connect to Postgresql+Postgis Docker container	postgres
	db_host	as above	127.0.0.1
	db_port	as above	5433
OpenStreetMap	osm_data	Downloaded OpenStreetMap (OSM) data	planet-latest_2020-08-13.osm.pbf
	osm_date	Date at which OSM download was current	20200813
	osmnx_retain_all	If false, only retain main connected network when retrieving OSM roads	FALSE
Sample points	points_id	sampling points unique id	location_id
	points	name of point data locations used for sampling	sampling_points
	point_sampling_interval	interval in metres along which network is to be sampled	30
Roads	intersection_tolerance	tolerance in metres for cleaning intersections	12
	network_edges	as above	edges
	network_junctions	as above	nodes
Network analysis	distance	sausage buffer network size -- in units specified above	1600
	tolerance	search tolerance (in units specified above)	500
	line_buffer	buffer distance for network lines as sausage buffer	50
	limit	distance is a limit beyond which not to search for destinations	1600
	aos_threshold	Distance within which all Areas of Open Space are sought	1600
Population	population_data	population raster dataset (defined in datasets worksheet)	population
	population_target	target year for population data	2015
	pop_min_threshold	hex grid cell estimated population inclusion threshold	5

Table F. Study region parameters used for pre-processing

City	EPSG code	Region boundary data	Additional region-specific parameter notes
Maiduguri	32633	GHS:UC_NM_MN='Maiduguri'	1
Mexico City	32614	./data/boundaries.gpkg:Mexico City	
Baltimore	32618	GHS:UC_NM_MN='Baltimore'	
Phoenix	32612	./data/boundaries.gpkg:Phoenix	
Seattle	32610	GHS:UC_NM_MN='Seattle'	
São Paulo	32723	./data/boundaries.gpkg:Sao Paulo	
Hong Kong	32650	./data/boundaries.gpkg:Hong Kong	2,3,4
Chennai	32644	./data/boundaries.gpkg:Chennai	
Bangkok	32647	./data/boundaries.gpkg:Bangkok	
Hanoi	32648	./data/boundaries.gpkg:Hanoi	
Adelaide	7845	./data/boundaries.gpkg:Adelaide	
Melbourne	7845	./data/boundaries.gpkg:Melbourne	
Sydney	7845	./data/boundaries.gpkg:Sydney	
Auckland	2193	./data/boundaries.gpkg:Auckland	
Graz	32633	./data/boundaries.gpkg:Graz	
Ghent	32631	./data/boundaries.gpkg:Ghent	
Olomouc	32633	./data/boundaries.gpkg:Olomouc	
Odense	32632	./data/boundaries.gpkg:Odense	
Cologne	32631	./data/boundaries.gpkg:Cologne	
Lisbon	3763	./data/boundaries.gpkg:Lisbon	
Barcelona	25831	./data/boundaries.gpkg:Barcelona	
Valencia	25830	./data/boundaries.gpkg:Valencia	
Vic	25831	./data/boundaries.gpkg:Vic	5
Bern	32633	./data/boundaries.gpkg:Bern	
Belfast	29902	./data/boundaries.gpkg:Belfast	

Notes on parameters accounting for study region exceptions to generic processing workflow

1. *Custom destinations:* Custom destination data to use, in addition to those identified using OSM. Use a comma-separated list specifying file name (located in study region folder, once generated), category plain name field, category full name field, Y coordinate, X coordinate, EPSG number. Specifically for Maiduguri: *Maiduguri_shops_convenience_complete_2020-07-13_categorised_final.csv,dest_name,dest_name_full,Latitude,Longitude,4326*

2. *No buffered study region:* Instead of using buffered study region, use regular study region for excerpting network from OSM. This may allow for looping over true islands to extract individual networks (e.g. Hong Kong), which may not be possible with the buffered region (which results in only retaining larger network segment). For Hong Kong, this is set to 'TRUE', for other cities it has been left blank.

3. *Network polygon iteration:* Iterate over polygons for network retrieval, and then combine. This is useful for cities spanning multiple islands, in conjunction with the '*No buffered study region*' parameter. For Hong Kong, this is set to 'TRUE', for other cities it has been left blank.

4. *Network connection threshold:* Minimum threshold distance in units as per project parameters for disconnected networks (e.g. road networks for cities spanning multiple islands) to be included in the combined network for a city. For Hong Kong, this is set to 200, a value determined through manual inspection of the geographic context; for other cities, it has been left blank.

5. *Not GHS urban intersection*: For cities where intersection with the GHS urban area is not valid. For Vic which does not have a corresponding urban area in the GHS dataset), this variable was set to 'TRUE', indicating that the provided boundary is to be used.

A2.2. Setup configuration

Project and study region configuration parameters were defined using configuration files and were used to facilitate our software framework's semi-automated workflow to calculate spatial indicators of the 25 study regions. Tables E and F summarise project parameters and study region–specific parameters used during pre-processing. Other parameters used for sampling estimate processing and indicator calculation were compiled into a series of configuration files within the project's Github repository, which can be constructed with a single command (https://github.com/global-healthy-liveable-cities/global-indicators/blob/7852424e4852c99b5b0f7b65982d289db28e2ed0/process/setup_config.py). Researchers can draw upon them as templates and adapt for a new project as required.

A2.3. Pre-processing input data

Data for each study region was pre-processed with a series of Python scripts, drawing upon the configuration parameters in Tables E and F for analyses, with output to city-specific PostgreSQL (PostGIS) databases, before final output in geopackage format as used in the main analyses. Generalised data pre-processing for urban boundaries, pedestrian street network, population, spatial features, and sample points has been described in our methods paper.[1] Specific details on deriving points of interest, regularly serviced public transport locations, and public open space access points are provided below.

A2.3.1 Points of interest

Destination categories were coded using a conceptual mapping of key-value pair terms (Table G) from OpenStreetMap point, line, and polygon features, which were identified by reviewing coding guidelines (general, and community/country specific) and following a preliminary dataset validation survey of local collaborators, as shown in Table B). This approach helped us to ensure that the definition of built environment features and points of interest was consistent across study regions and matched the understandings of local experts. Where OpenStreetMap representation was considered inadequate based on the validation findings, we asked local collaborators to nominate usage of custom data for the study regions. In our study, this was only required for Maiduguri, Nigeria, because we found that, among the 25 study regions, Maiduguri is the only notable outlier regarding the representation of daily living amenities on OpenStreetMap (Table H). The custom destination data for Maiduguri were collected directly from the field by trained field assistants using handheld GPS, and the locations recorded in Microsoft Excel.

Table G. OpenStreetMap tags (key-value pairs) used to identify urban features

Variable	Description	OpenStreetMap tag	
		Key	***Value***
healthy_food_market	Healthy Food / Market		
		shop	supermarket
		supermarket	supermarket
		amenity	supermarket
		building	supermarket
		shop	grocery
		shop	bakery
		shop	pastry
		name	Tortillería
		shop	butcher
		shop	seafood
		shop	fishmonger
		shop	greengrocer
		shop	fruit
		shop	fruits
		shop	vegetables
		shop	deli
		shop	cheese
		amenity	marketplace
		amenity	market
		amenity	market_place
		amenity	public_market
		shop	marketplace
		shop	market
convenience	Convenience		
		shop	convenience
		amenity	fuel
		shop	kiosk
		shop	newsagent
		shop	newsagency
		amenity	newsagency
pt_any	Public transport stop (any)		
		public_transport	platform
		public_transport	stop_position
		highway	bus_stop
		highway	platform
		railway	platform
		public_transport	station
		amenity	ferry_terminal
		railway	tram_stop
		railway	stop

Table H. Preliminary OpenStreetMap destination data audit results

City	Population estimate[1]	Urban area (km^2)	Population per km^2	OpenStreetMap destination count		
				Healthy food	Convenience	Public transport
Maiduguri[2]	1,077,912	125	8,606	23	2	2
Mexico City	20,217,799	2,312	8,745	1,491	1,684	1,765
Baltimore	621,588	229	2,709	78	3,577	292
Phoenix	1,335,215	772	1,730	101	4,274	711
Seattle	922,474	551	1,675	371	5,984	695
São Paulo	11,770,758	1,018	11,564	1,562	5,104	1,819
Hong Kong	7,287,172	373	19,562	748	9,366	935
Chennai	6,502,693	425	15,314	209	853	151
Bangkok	9,301,270	1,190	7,814	654	1,247	1,667
Hanoi	5,936,947	1,220	4,865	537	915	1,127
Adelaide	995,195	541	1,840	352	5,985	297
Melbourne	3,753,083	1,657	2,265	1,530	8,968	1,707
Sydney	4,091,396	1,334	3,068	922	7,693	1,037
Auckland	1,247,659	468	2,666	659	5,028	776
Graz	280,642	69	4,085	285	2,122	187
Ghent	242,180	107	2,265	221	1,316	162
Olomouc	86,237	27	3208	60	340	64
Odense	151,224	56	2,688	97	244	65
Cologne	1,126,218	348	3,240	874	3,020	889
Lisbon	585,346	85	6,891	368	3,211	264
Barcelona	3,253,794	359	9,052	1,949	7,527	887
Valencia	729,856	86	8,485	395	1,537	152
Vic[3]	43,920	31	1,436	60	91	14
Bern	144,427	32	4,472	149	611	139
Belfast	385,650	98	3,931	95	298	138
**Percentile summary**						
0	43,920	27	1,436	23	2	2
25	385,650	86	2,666	101	853	151
(median) 50	1,077,912	359	3,931	368	2,122	297
75	4,091,396	772	8,485	748	5,104	935
100	20,217,799	2,312	19,562	1,949	9,366	1,819

[1] Population estimate was derived from the Global Human Settlement 2015 modelled population layer. The value corresponded to the population estimate within the identified urban portion of the city, identified using the Global Human Settlement dataset (with exception of Maiduguri and Vic). This was used as a common reference point for population data for all cities, as it was available at both a recent time point and high resolution (approximately 250sqm grid). The population data was used spatially in the project to indicate relative population density. Values in absolute terms may differ from other estimates, including those officially available, or more recent for the greater city areas which extend beyond urban centres.

[2] It was found that Maiduguri had relatively lower OSM destination counts than other cities of comparable urban size and population in the study. This observation led to the development of methods to include custom destinations, and an independent data-collection effort for Maiduguri.

[3] Vic appeared to be well represented with OSM destinations for its size and population. However, unlike all other cities it did not intersect a 2015 'urban area' identified by the Global Human Settlements dataset. In this regard, it should be noted that Vic is exceptional compared with other cities, whose urban study regions were defined using, or in conjunction with, the GHS urban layer; Vic's study region used in the above is its own administrative boundary (supplied by collaborators).

A2.3.2 Public transport stops with regular daytime weekday service

The usual daytime weekday frequency of public transport service is a marker of its utility, beyond proximity: if a bus stop is close, but rarely served, its usefulness is more limited than a regularly served stop. There are many factors that could be considered as markers of utility for public transport which were beyond the scope of our study—for example, the cost or time taken to reach a particular destination. For this study, we sought to estimate the percentage of population with access to a public transport stop served frequently enough that an average wait during weekday daytime hours (7am to 7pm) would be approximately 10 minutes; that is, a headway of 20 minutes, with average wait time conceptualised as half of the headway between departures.[4]

Public transport schedule data were obtained from transport agencies in General Transit Feed Specification (GTFS) format. GTFS (https://gtfs.org/, https://developers.google.com/transit/gtfs/reference) provides a format that has been broadly adopted by public transport agencies around the world for publishing and disseminating regularly updated transport schedules since 2005 (https://beyondtransparency.org/chapters/part-2/pioneering-open-data-standards-the-

gtfs-story/). It is an established standard for representing public transportation schedules used for urban transport research in diverse contexts.[5-7] The GTFS dataset is a zipped folder comprising a series of text files that contain comma-delimited data for routes, stop times, dates of service, and agency metadata, which can be merged to provide granular detail of a transport agency's services at each of its stop locations for a particular period.

We identified cities with available GTFS data targeting 2019 for public transport stop location headway analysis. We developed a headway analysis method broadly following Arundel et al.,[8] except that we used headway instead of median inter-arrival time. Methodological details of the analysis are presented as open-source code available at the repository. For fair comparisons, we sought to include cities in the analysis with complete GTFS coverage for the buffered study regions, and with feeds schedules for a time in Spring 2019 when most schools were not on holidays (5 April to 5 June for Northern Hemisphere cities; 8 October to 5 December for those in the Southern Hemisphere). Not all schedules neatly met this criterion. Specifically, we approximated coverage for Chennai using a feed dated to 2010 to capture rail service representation in particular; this compromise was preferable to exclusion, but we recognised it as an important caveat on presenting the results of this city.

Public transport stop locations were analysed to identify those with normal weekday operation time from 7am to 7pm during the time period of interest for that city. The analysis time period was specified for each study region in Table D. Average headways were calculated for each stop location based on the number of daily departures during the analysis time period. Subsequently, three sets of public transport locations were exported to support the accessibility analysis of sample points: 1) any public transport stops (combining GTFS and OSM data); 2) stops with a headway of 20 minutes; and 3) stops with a headway of 30 minutes (for use as a sensitivity analysis for the effect of threshold choice). The modes of public transport in the GTFS specification include tram, metro, rail, bus, ferry, cable tram, aerial lift, funicular, trolleybus, and monorail. Due to some inconsistencies in the GTFS specification by different transport agencies, some cities required custom parameterisation to match the specific implementation used by their transport agencies. For example, Bern and Sydney each required custom specification of route numbers in the configuration of feeds for these cities, to accurately represent their transport modes.
A summary of the transport stop headway analysis is shown in Table I.

Table I. GTFS stop count within 500m of urban study region boundaries (all modes), where data was identified and retrievable

City	GTFS stops within 500m of urban study region boundary				
	N	Headway			
		n ≤ 30 mins	n ≤ 20 mins	% ≤ 30 mins	% ≤ 20 mins
Maiduguri	–	–	–	–	–
Mexico City	5,575	3,758	2,707	67.4	48.6
Baltimore	4,145	2,863	2,171	69.1	52.4
Phoenix	4,285	3,932	1,053	91.8	24.6
Seattle	7,225	4,531	2,837	62.7	39.3
São Paulo	20,063	19,550	17,712	97.4	88.3
Hong Kong	4,308	4,102	3,141	95.2	72.9
Chennai	58	35	35	60.3	60.3
Bangkok	6,218	6,111	3,759	98.3	60.5
Hanoi	6,871	451	152	6.6	2.2
Adelaide	6,064	4,865	2,611	80.2	43.1
Melbourne	17,085	11,289	6,858	66.1	40.1
Sydney	21,679	12,297	6,982	56.7	32.2
Auckland	10,044	8,252	4,416	82.2	44.0
Graz	–	–	–	–	–
Ghent	–	–	–	–	–
Olomouc	–	–	–	–	–
Odense	545	334	231	61.3	42.4
Cologne	973	881	649	90.5	66.7
Lisbon	2,119	1,878	1,481	88.6	69.9
Barcelona	3,138	2,828	2,485	90.1	79.2
Valencia	1,175	1,155	1,127	98.3	95.9
Vic	–	–	–	–	–
Bern	410	372	321	90.7	78.3
Belfast	1,550	1,159	875	74.8	56.5

A2.3.3 Public open space

Parks, nature reserves, plazas, and squares could all be considered areas of public open space: open areas where people may gather for leisure. The identification of public open space using OpenStreetMap is a distinct challenge to other kinds of destinations, which are usually localised as discrete 'points'. Public open spaces are areas, not points, and so more complex topological relationships must be considered in addition to mapping of classification tags. In terms of representation on OpenStreetMap, open spaces may be located next to each other; in effect, a jointly larger area than contiguous polygonal boundaries taken individually would suggest. They may also be nested within larger areas of open space, which may or may not be publicly accessible.

A series of logical queries were used to first identify areas of open space; meeting any one of these was grounds for consideration as a potential public open space. These are summarised in Table J.

A layer of areas that were categorically not to be considered as public open space was created, and any portions of potential areas of open space that overlap areas marked for exclusion were also excluded. For example, if there was an area coded to suggest it could be a natural area that might potentially be an open space (e.g. 'boundary=nature_reserve'), but was located entirely within an area with a military or industrial land use, or was otherwise tagged to indicate that access was not public (e.g. for employees or staff only, private, or otherwise inaccessible), this was not considered an area of public open space and was excluded. In addition, areas of public open space that were not larger than a minimum size threshold (10 m^2) were excluded to ensure incidental slivers of public land like small nature strips did not contribute spuriously to estimates of access to public open space.

Once areas of public open space had been identified, proxy locations for possible entry points were created at regular intervals (every 20 metres) on the sections of the boundaries of those areas of public open space which are within 30 metres of the road network.[9] These pseudo-entry points can be used to evaluate distance to the nearest public open space, regardless of the fact that our data cannot be used to infer where the true entry locations are (and in many cases, there may be no formal entry points). Sets of pseudo-entry points were respectively exported for areas of public open space of any size, and those with a public area of greater than 1.5 hectares. The final sets of access points were used in origin–destination network analyses evaluating network distance to near public open space.

Table J. Processing parameters for areas of open space

Description	Values
Required tags	
These tag keys are assumed to be present on OSM features in order to evaluate values (null or otherwise); if they don't exist, they are created with null values when setting up OSM data.	beach,river,water,waterway,wetland,access,leisure,natural,sport,landuse,playground,boundary,recreation_ground,golf,military,agricultural,forestry,tourism,shop,supermarket,amenity,building,community_centre,place_of_worship,tourism,cuisine,gambling,place,highway,swimming_pool,garden:type
Specific inclusion criteria	
These are specific inclusion criteria which are to be joined as an OR query, using a specific table alias.	leisure IS NOT NULL,beach IS NOT NULL,place = 'square',highway = 'pedestrian'
Land use considerations	
These tags are to be joined in a comma-separated list, once they have been enclosed in single quotation marks.	common,conservation,forest,garden,leisure,park,recreation_ground,sport,trees,village_green,winter_sports,wood,dog_park,nature_reserve,off_leash,sports_centre,riverbank,beach
Boundary considerations	
These tags are to be joined in a comma-separated list, once they have been enclosed in single quotation marks.	national_park,nature_reserve,forest,state_forest,state_park,regional_park,park,county_park
Exclusion on keys	
Tags are joined using OR logic, and are used to define exclusion criteria where values are not null.	military,agricultural,forestry
Exclusion on values	
Where the keys in this json snippet are found to have values in their associated lists, these are grounds for exclusion. This snippet is used to format exclusion criteria using OR logic.	
Water features	
These tags are to be joined in a comma-separated list, once they have been enclosed in single quotation marks. These value tags indicate areas of blue space, which will be excluded when determining both access to and size of parks.	atoll,awash_rock,bay,coastal,coastline,coastline_old,glacier,high-water,hot_spring,island,islet,lake,marsh,oasis,old_coastline_import,peninsula,pond,river,river_terrace,riverbank,riverbed,shoal,spring,strait,stream,swamp,swimming_pool,underwater_rock,unprotected_spring,unprotected_well,water,water_park,waterfall,waterhole,waterway,wetland
Water sports	
These tags are to be joined in a comma-separated list, once they have been enclosed in single quotation marks. These tags are indicative of water features, which will be excluded from consideration.	swimming,surfing,canoe,scuba_diving,rowing,sailing,fishing,water_ski,water_sports,diving,windsurfing,canoeing,kayak

Table J. (cont.) Processing parameters for areas of open space

Description	Criteria
Linear features	
These tags are to be joined in a comma-separated list, once they have been enclosed in single quotation marks. These are linear features which may or may not be public open space, but need to be treated with care so they do not link together to form large areas of open space.	river,riverbank,riverbed,strait,waterway,stream,ditch,river,drain,canal,rapids,drystream,brook,derelict_canal,fairway
Linear feature criteria	
This is an SQL expression used to define a linear feature based on morphological or attribute criteria.	(area_ha > 0.5 AND roundness < 0.25) OR (waterway IS NOT NULL OR river IS NOT NULL)
Tags to exclude	
These tags are to be joined in a comma-separated list, once they have been enclosed in single quotation marks.	addr:city,addr:full,addr:place,addr:postcode,addr:province,addr:street,website,wikipedia,description,addr:housenumber,addr:interpolation,designation,email,phone,ref:capad2014_osm,nswlpi:cadid,wikidata,url
Tags to retain	
Keys to be joined in a comma-separated list (already in double quotation marks).	"os_id","area_ha","beach","river","public_access","within_public","amenity","access","boundary","golf","landuse","leisure","natural","playground","recreation_ground","sport","tourism","water","wetland","waterway","wood","water_feature","min_bounding_circle_area","min_bounding_circle_diameter","roundness","linear_feature","acceptable_linear_feature","highway","place"
Exclusion criteria for public spaces	
Where the keys in this json snippet are found to have values in their associated lists, these are used to indicate areas that are not to be flagged as public themselves, although they may be located inside larger areas of public open space.	{"amenity":["aged_care","animal_boarding","allotments","animal_boarding","bank","bar","biergarten","boatyard","carpark","childcare","casino","church","club","club_house","college","conference_centre","embassy","fast_food","garden_centre","grave_yard","hospital","gym","kindergarten","monastery","motel","nursing_home","parking","parking_space","prison","retirement","retirement_home","retirement_village","school","scout_hut","university"],"leisure":["garden","golf_course","horse_riding","pitch","racetrack","summer_camp","sports_club","stadium","sports_centre"],"building":["yes"],"area":["school"],"natural":["fell","bay","bog","cliff","geyser","reef","scrub","sinkhole","strait","volcano","wetland","wood","water"],"recreation_ground":["showground","school_playing_field","horse_racing","show_grounds","school_playing_fields"],"sport":["archery","badminton","bocce","boules","bowls","croquet","dog_racing","equestrian","futsal","gokarts","golf","greyhound_racing","horse_racing","karting","lacross","lacrosse","lawn_bowls","motocross","motor","motorcycle","polo","shooting","snooker","trugo"],"access":["customers","private","no"],"tourism":["alpine_hut","apartment","aquarium","bed_and_breakfast","caravan_site","chalet","gallery","guest_house","hostelsd","hotel","information","motel","museum","theme_park","zoo"],"garden:type":["residential","residental","private","commercial","pub","school","roof_garden"]}

A2.4. Processing sampling estimates, and aggregating indicators

Sample points estimates, urban neighbourhood grid, and city summaries of spatial indicators were processed following our open-source software framework. Specific methods applied to the case study cities are described in the current manuscript, and more details for the software framework are presented in our method paper.
A completed list of descriptive variables and spatial indicators is shown in Table K.

Table K. Descriptive variables and spatial indicators of urban design and transport features that promote health and sustainability

Indicator	Units	Comments	Data notes
**Descriptive variables**			
Urban study region area	km²	Urban study region boundaries	1 2
Urban study region population estimate	population counts	Overall urban population estimate for the study region	1 2 3
GNI per capita classification (2021)	Lower<br>Lower-middle<br>Upper-middle<br>High	The World Bank 2021 fiscal year GNI per capita classification (Atlas method)	7
Population per square kilometre		Urban population density estimate	1 2 3
Sample point count	points	Sample points generated every 30m along pedestrian network for 250m diagonal urban hexagonal grid regions with population estimates greater than 5	1 2 3 4
**Percentage of population with access to a …**		Pedestrian network distance indicators of access within 500m, evaluated for hexagonal grid cells and weighted for population percentage estimate	
Healthy food market or supermarket	%	Including supermarket, fresh food grocers, and food markets that sell fresh food ingredients that support options for healthier choices	1 2 3 4 5
Convenience store	%	Including newsagencies and convenience stores where basic staples and prepared food items may be purchased	1 2 3 4 5
Public transport stop			
any (OSM or GTFS)	%	The best estimate of access using either OpenStreetMap or GTFS sourced data (noting that both are imperfect)	1 2 3 4 5 6
with regular service (≤30 mins)	%	Accounting for 30 minute or less average weekday daytime service frequency	1 2 3 4 5
with regular service (≤20 mins)	%	Accounting for 20 minute or less average weekday daytime service frequency	1 2 3 4 5
Public open space			
any	%	Includes squares, parks, and other publicly accessible areas of natural land for leisure and recreation purposes.	1 2 3 4 5
1.5 hectares or larger	%	As above, restricted to areas with large publicly accessible areas	1 2 3 4 5
**Local walkability indicators**		Unweighted (spatial) and population-weighted city summary measures, both within city (in absolute units), and relative to all cities (as Z-scores)	
Population per square kilometre	density	Average of estimated population density within 1,000m local walkable catchments	1 2 3 4
Intersections per square kilometre	density	Average of estimated intersection density within 1,000m local walkable catchments	1 2 3 4
Daily living score	Sum of binary scores	Sum of binary access indicator scores to supermarket, convenience store, and public transport (OSM or GTFS) servicing, as a proxy of land use mix	1 2 3 4 5 6
Walkability index	sum of z-scores	Sum of Z-scores (within city, and between city) of population density, intersection density, and daily living score	1 2 3 4 5 6

1 GHS UCDB 2015 (See Table C)
2 Custom boundaries (see Appendix A1.3)
3 GHS POP 2015 (See Table C)
4 OpenStreetMap derived pedestrian network, using OSMnx (See Table C)
5 OpenStreetMap derived points of interest (see Appendix A2.3.1)
6 GTFS (mixed sources, targeting 2019; see Appendix A2.3.2)
7 World Bank list of economies (June 2020), http://databank.worldbank.org/data/download/site-content/CLASS.xls , last accessed 21 April 2021

A3. Mapping

In addition to some automated mapping using Python with Geopandas and Matplotlib, we also prepared bespoke maps for our publication using Giulio Fattori's QGIS multi-map plug in .[10] Our maps made use of Fabio Crameri's Scientific Colour Maps Batlow colour scale .[11] Please see our extensive supplementary map document in Appendix B for detailed maps for each city.

References

1. Liu S, Higgs C, Arundel J, et al. A generalized framework for measuring pedestrian accessibility around the world using open data. *Geogr Anal* 2021.
2. Florczyk A, Corbane C, Schiavina M, et al. GHS-UCDB R2019A - GHS Urban Centre Database 2015, multitemporal and multidimensional attributes. European Commission, Joint Research Centre (JRC); 2019.
3. QGIS.org. QGIS Geographic Information System. 2021 (accessed December 8 2021).
4. Nielsen G, Lange T. Network design for public transport success–theory and examples. International Conference on Competition and Ownership in Land Passenger Transport; 2007.
5. Kujala R, Weckström C, Darst RK, Mladenović MN, Saramäki J. A collection of public transport network data sets for 25 cities. *Scientific Data* 2018; **5**(1): 180089.
6. Hadas Y. Assessing public transport systems connectivity based on Google Transit data. *J Transp Geogr* 2013; **33**: 105-16.
7. Farber S, Morang MZ, Widener MJ. Temporal variability in transit-based accessibility to supermarkets. *Appl Geogr* 2014; **53**: 149-59.
8. Arundel J, Lowe M, Hooper P, et al. Creating liveable cities in Australia: Mapping urban policy implementation and evidence-based national liveability indicators. Melbourne: RMIT University Centre for Urban Research, 2017.
9. Higgs C, Badland H, Simons K, Knibbs LD, Giles-Corti B. The Urban Liveability Index: developing a policy-relevant urban liveability composite measure and evaluating associations with transport mode choice. *Int J Health Geogr* 2019; **18**(1): 14.
10. Fattori G. Multi Maps. (QGIS plugin). 2021. https://github.com/Korto19/Multiple-Maps (accessed 7 December 2021).
11. Crameri F. Scientific colour maps. 2021. https://zenodo.org/record/5501399#.YbMrXdDMIQ8 (accessed December 7 2021).

Appendix B: Map reports

(City map report sections are identified in the PDF bookmarks)

Contents

APPENDIX B 24

Global comparisons

Relative urban neighbourhood walkability across 25 global cities 25

Access to large public open space within 500m across 25 global cities 26

Access to a fresh food outlet (fruit and vegetable grocer, market, or supermarket) within 500m across 25 global cities 27

Access to any public transport within 500m across 25 global cities 28

Access to public transport with day time weekday service frequency of 20 minutes or better within 500m across 25 global cities, where GTFS data were available 29

City specific maps

Africa

Maiduguri, Nigeria 30

America, North

Mexico City, Mexico 45

Baltimore, USA 60

Phoenix, USA 76

Seattle, USA 91

America, South

Sao Paulo, Brazil 106

Asia

Hong Kong, China 123

Chennai, India 137

Bangkok, Thailand 154

Hanoi, Vietnam 168

Australasia

Adelaide, Australia 183

Melbourne, Australia 198

Sydney, Australia 213

Auckland, New Zealand 228

Europe

Graz, Austria 243

Ghent, Belgium 258

Olomouc, Czechia 273

Odense, Denmark 288

Cologne, Germany 303

Lisbon, Portugal 318

Barcelona, Spain 333

Valencia, Spain 348

Vic, Spain 363

Bern, Switzerland 378

Belfast, United Kingdom 393

Relative urban neighbourhood walkability across 25 global cities *(click a city to view map report)*

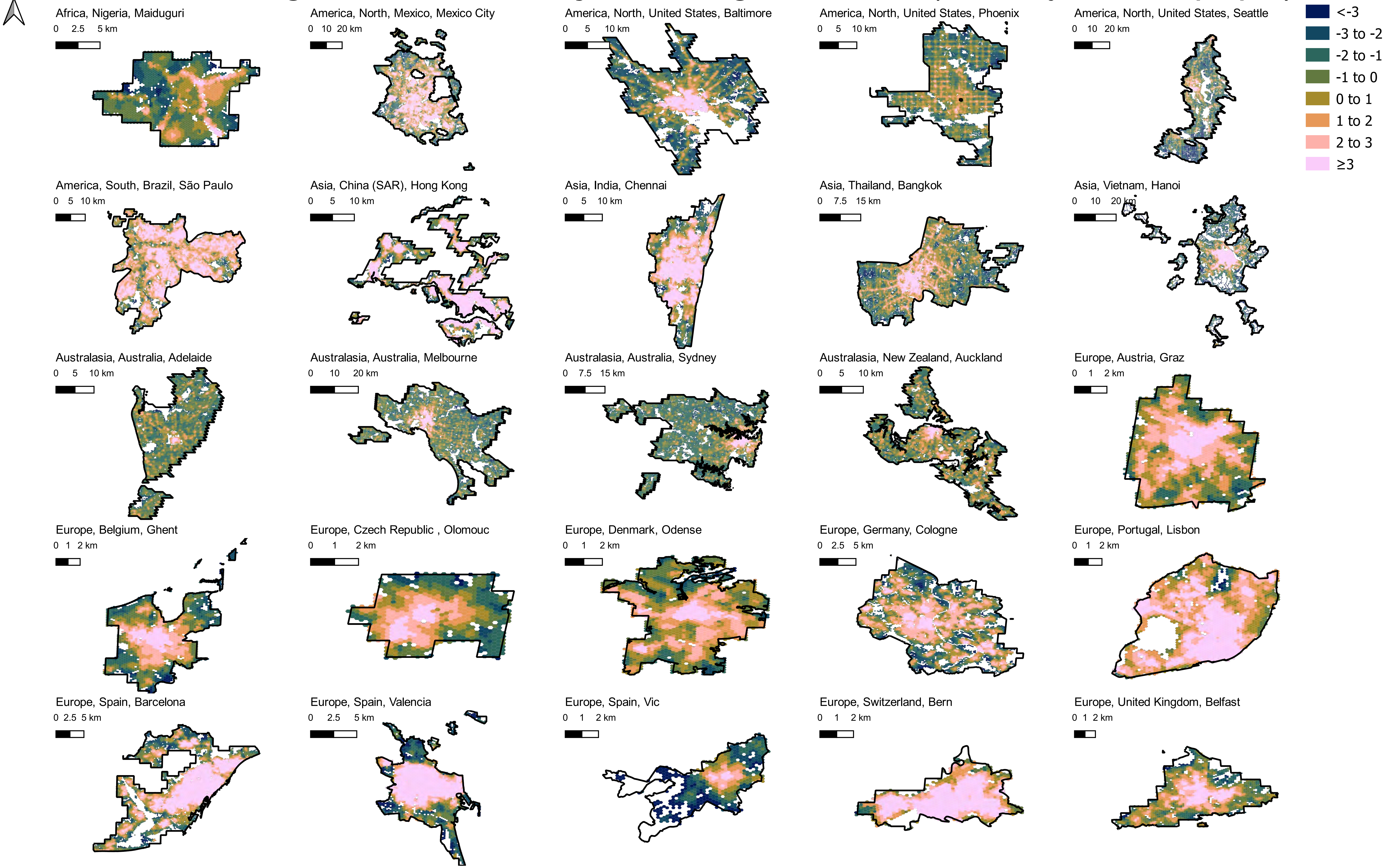

Access to large public open space within 500m across 25 global cities*

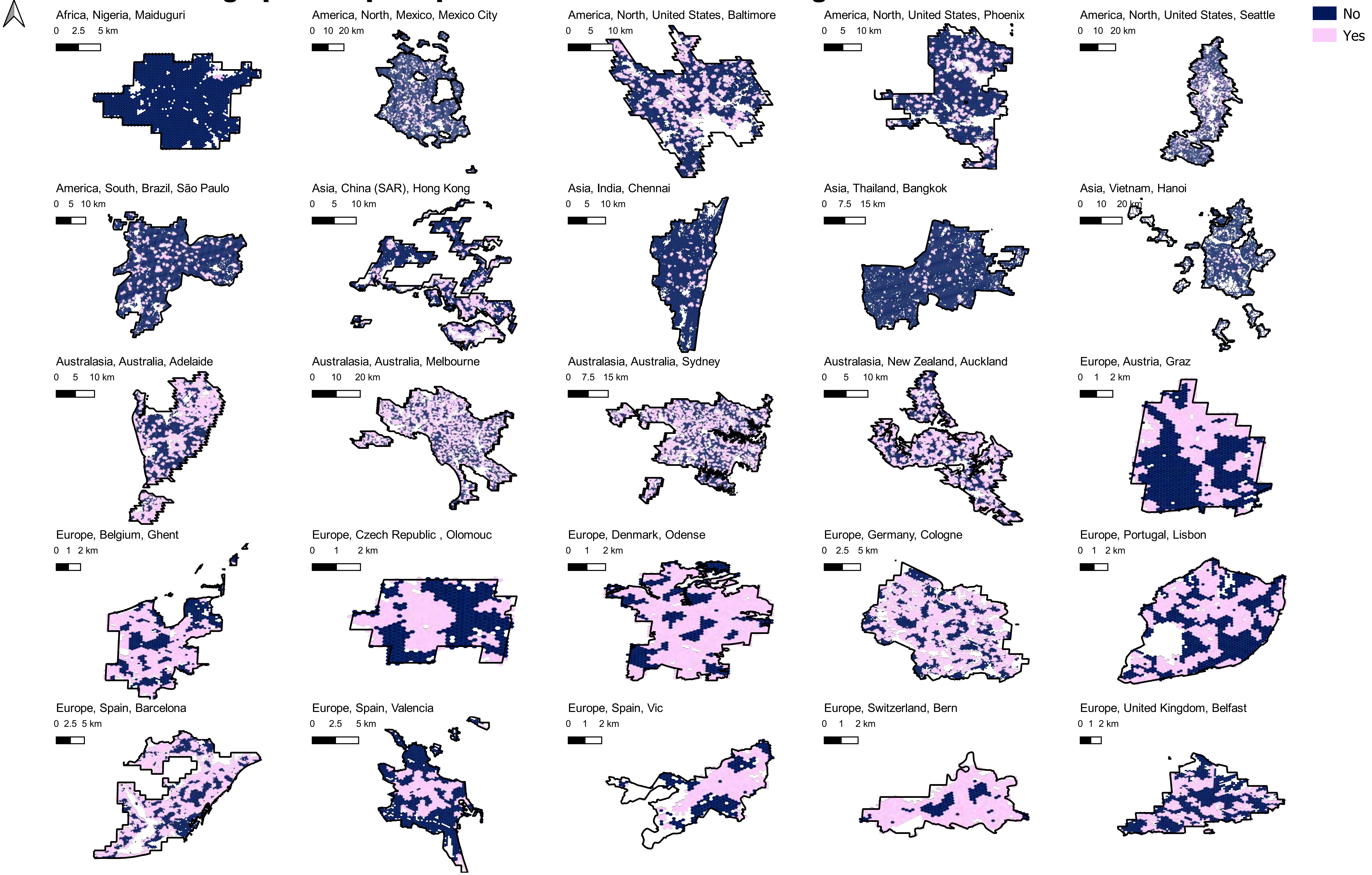

* In this map access for urban neighbourhoods was considered achieved when at least half of the sampled walkable area was estimated to within be located within 500m of areas identified as public open space of size 1.5 hectares or larger.

Access to a fresh food outlet (fruit and vegetable grocer, market, or supermarket) within 500m across 25 global cities*

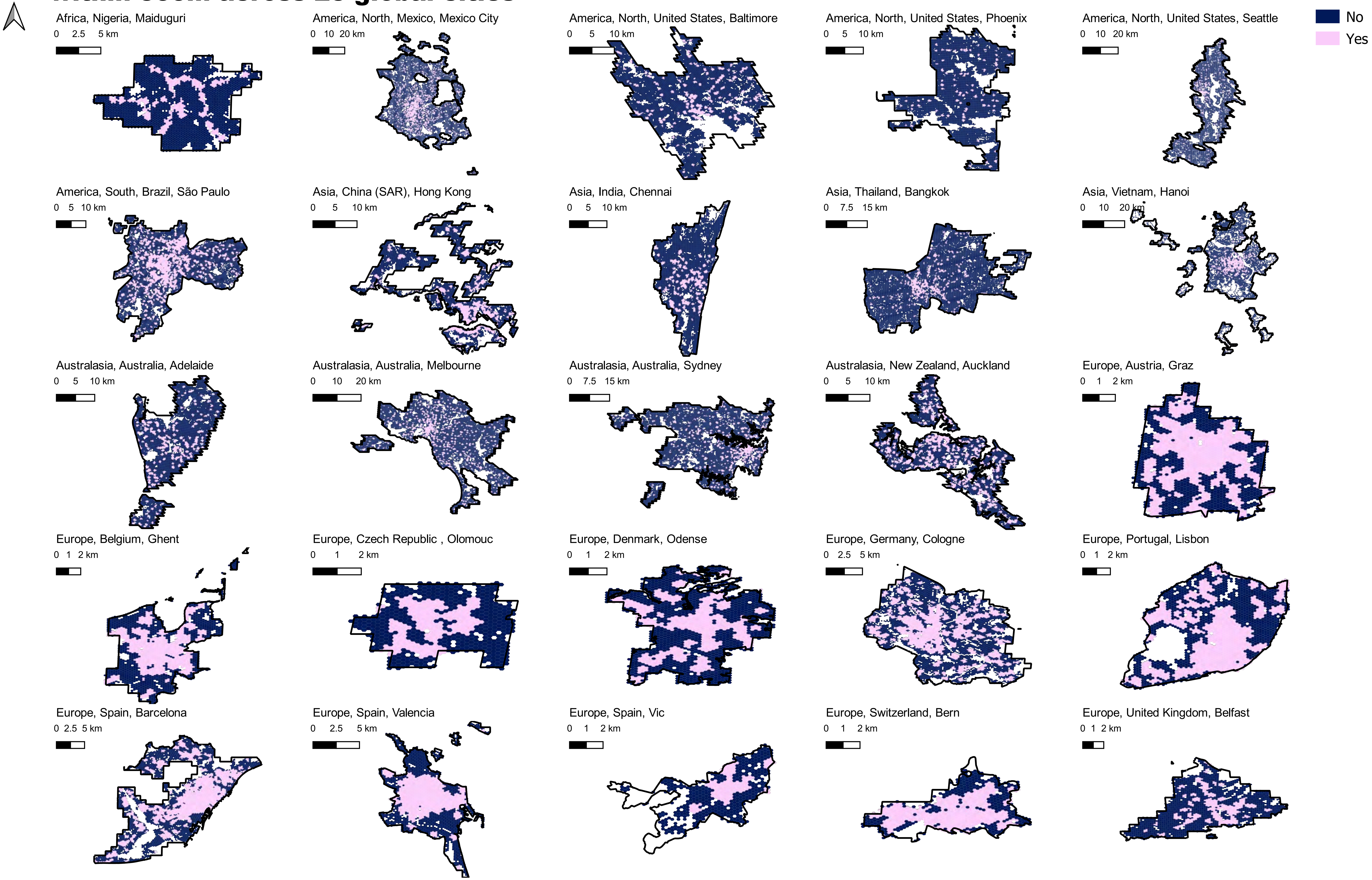

* In this map access for urban neighbourhoods was considered achieved when at least half of the sampled walkable area was estimated to within be located within 500m of identified features of interest.

Access to any public transport within 500m across 25 global cities*

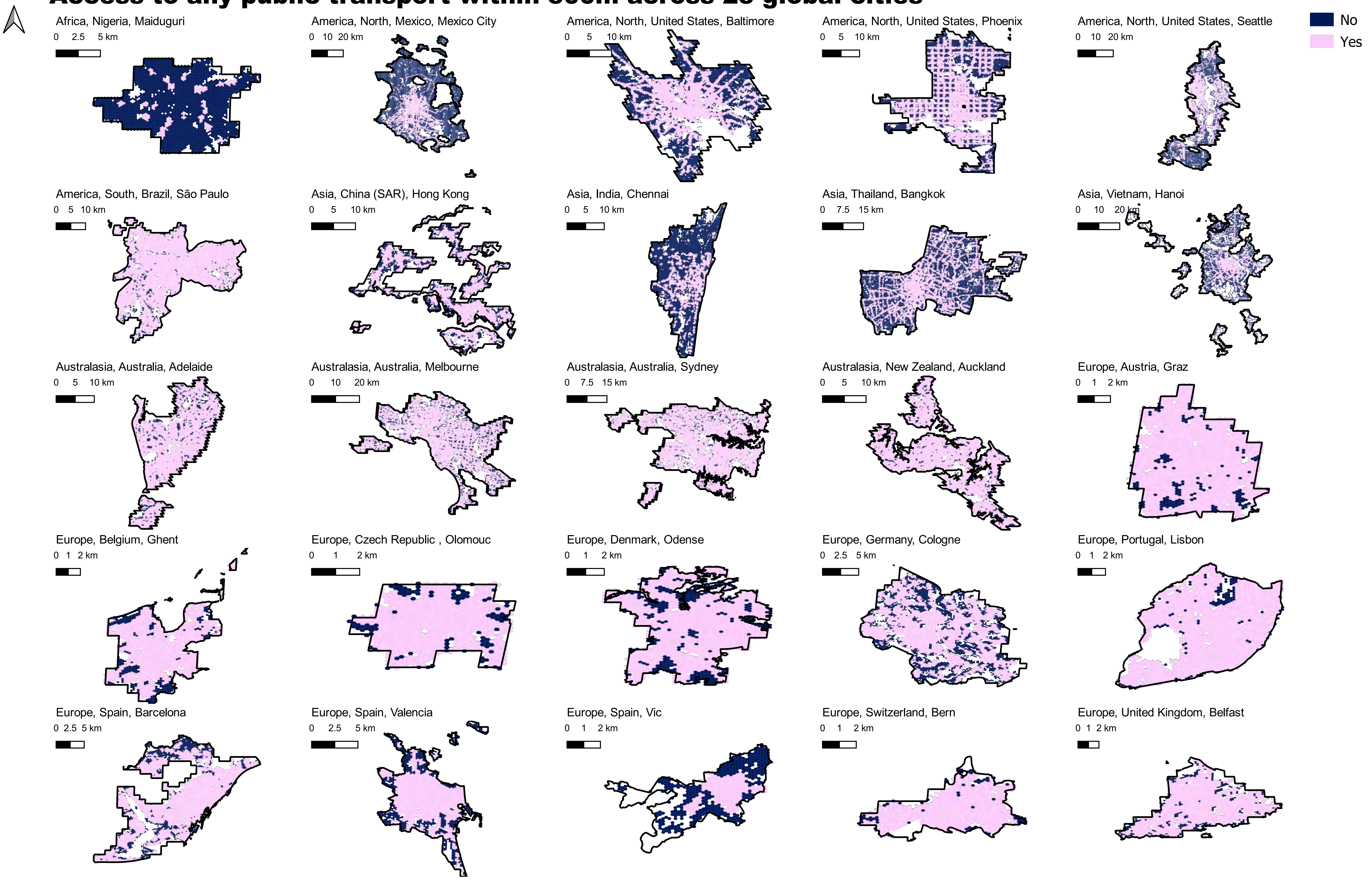

* In this map access for urban neighbourhoods was considered achieved when at least half of the sampled walkable area was estimated to within be located within 500m of identified features of interest.

Access to public transport with day time weekday service frequency of 20 minutes or better within 500m across 25 global cities, where GTFS data were available*

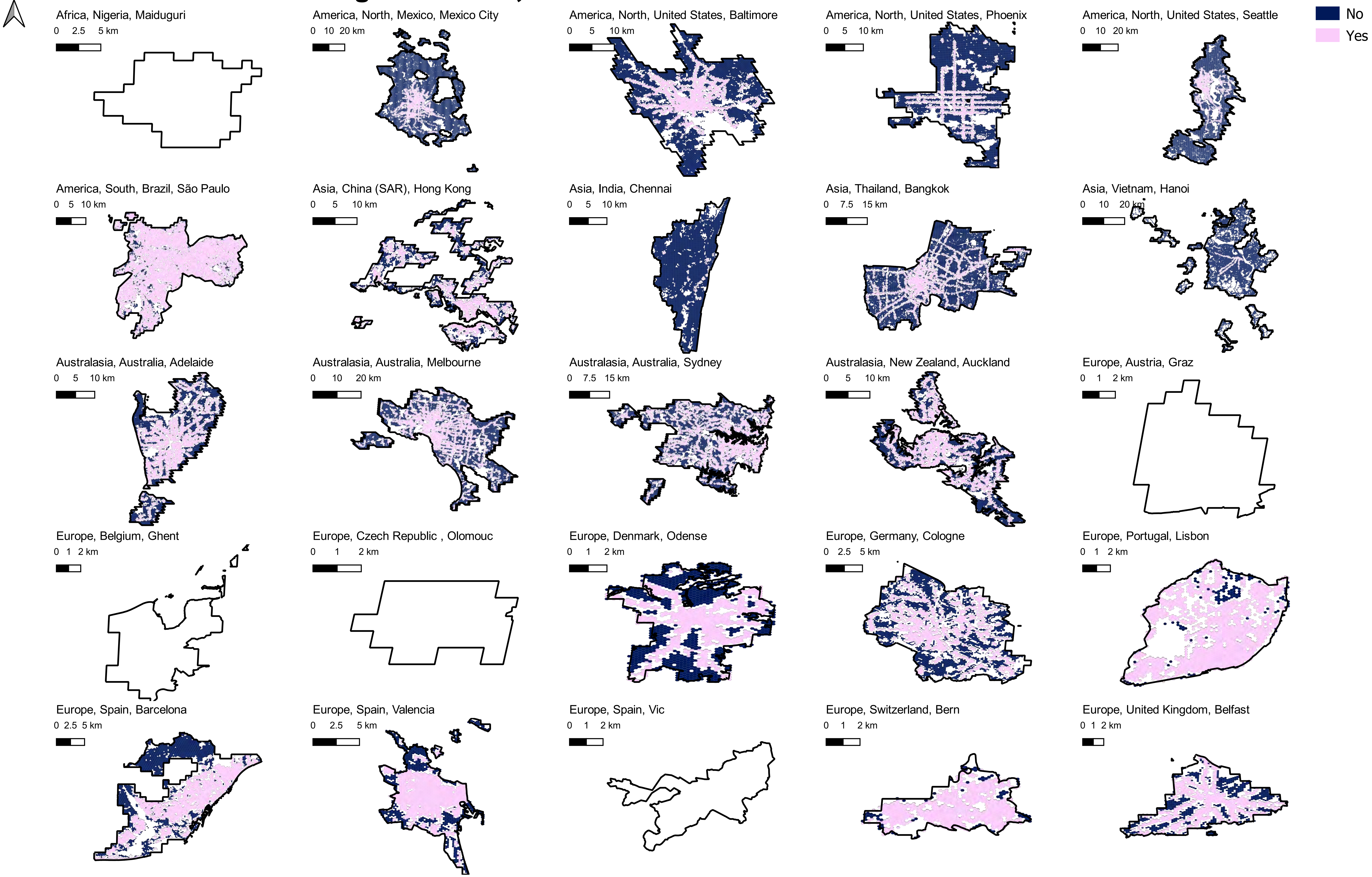

* In this map access for urban neighbourhoods was considered achieved when at least half of the sampled walkable area was estimated to within be located within 500m of identified features of interest.

Africa, Nigeria, Maiduguri

Satellite imagery of urban study region (Bing) | Walkability, relative to city | Walkability, relative to 25 global cities

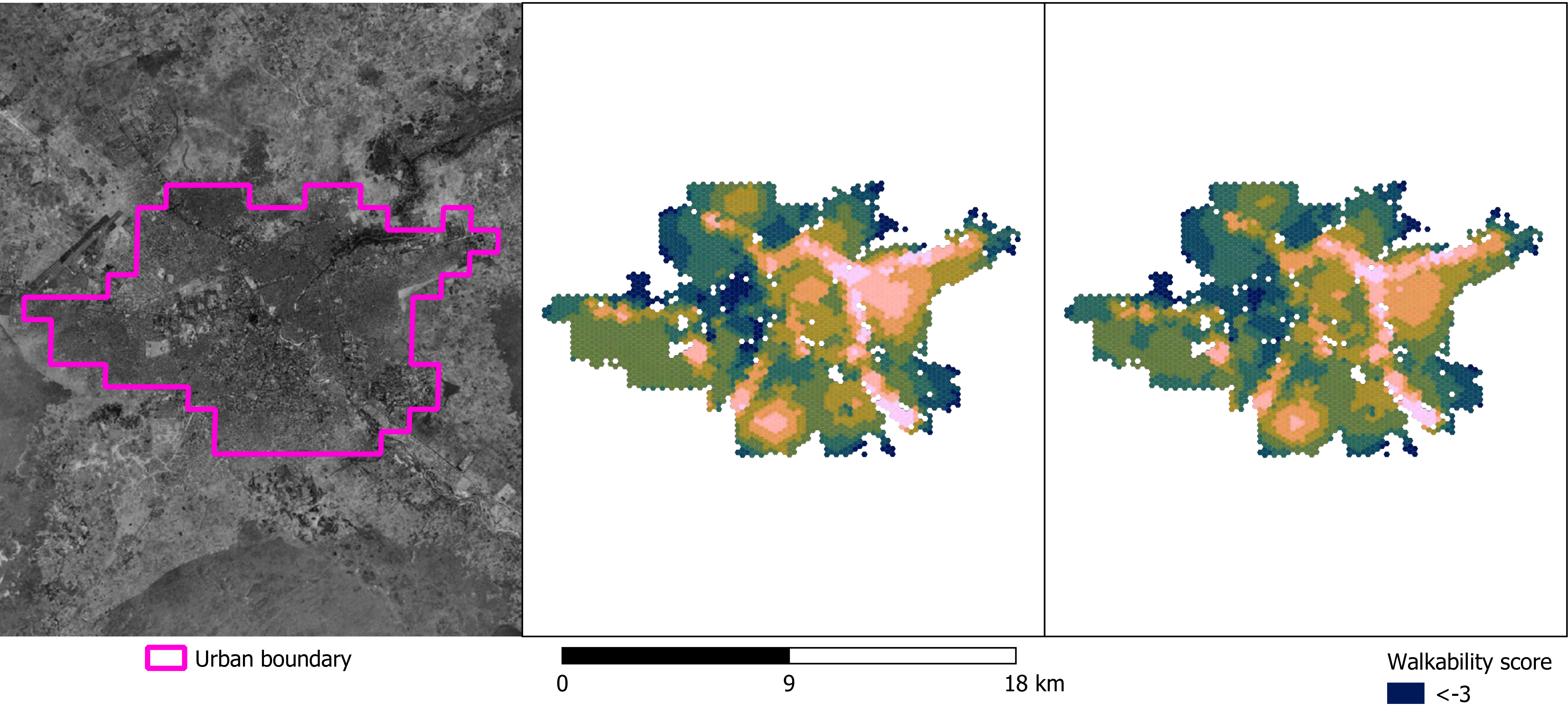

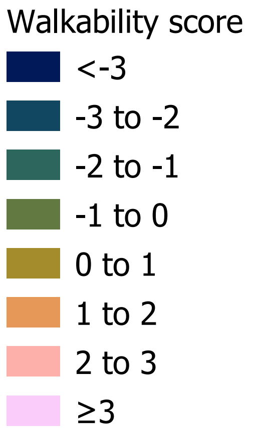

Walkability relative to all cities by component variables (2D histograms), and overall (histogram)

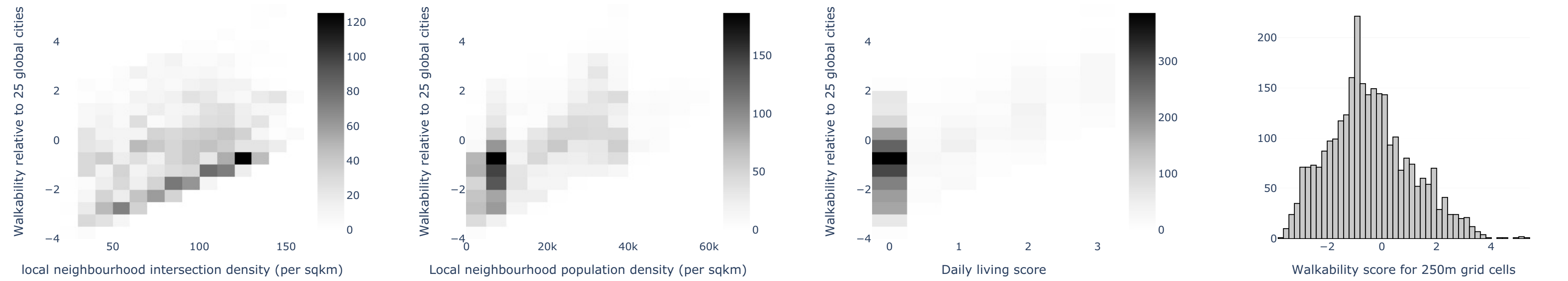

Mean 1000 m neighbourhood population per km²

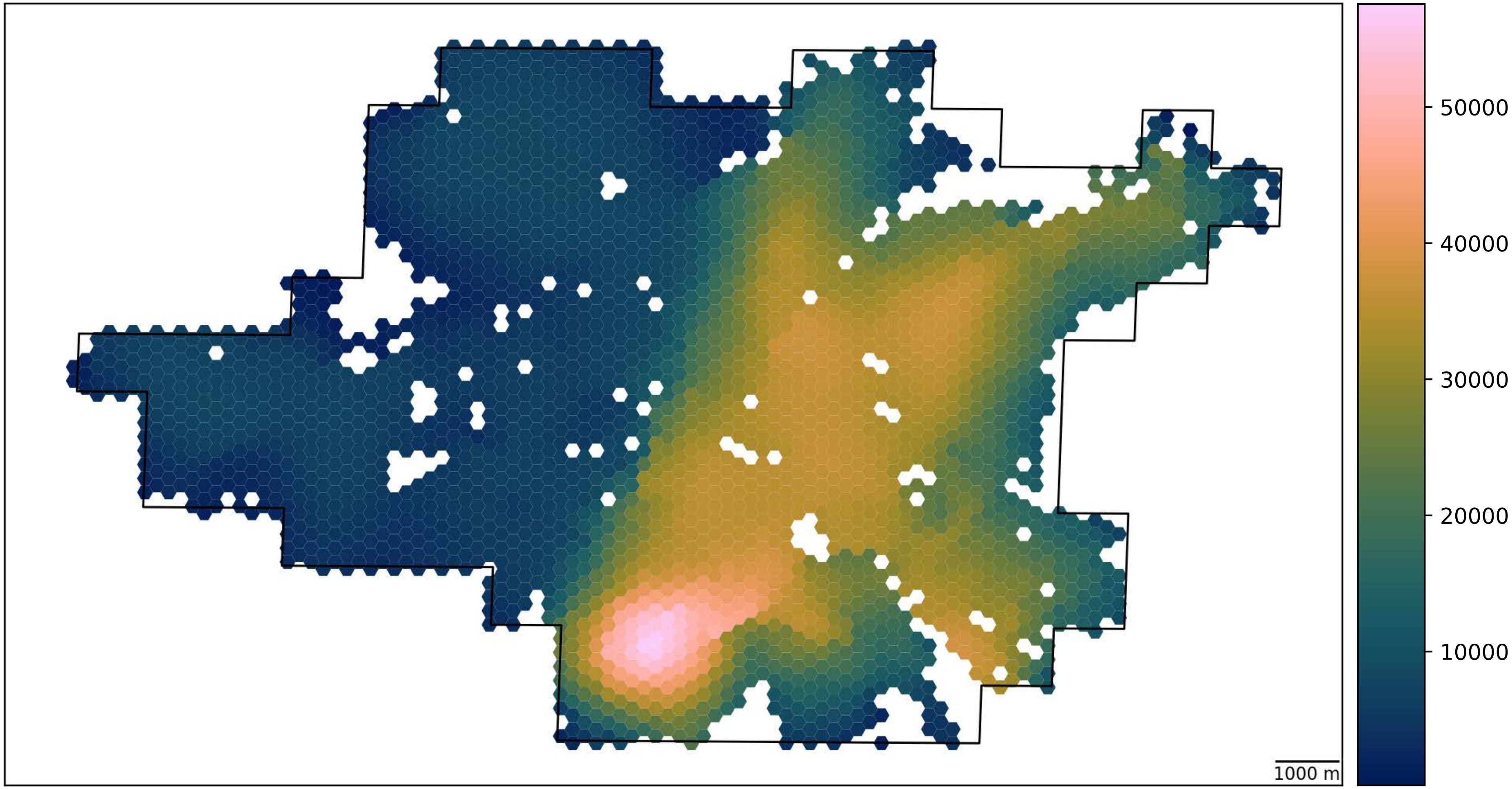

A: Estimated Mean 1000 m neighbourhood population per km² requirement for ≥80% probability of engaging in walking for transport

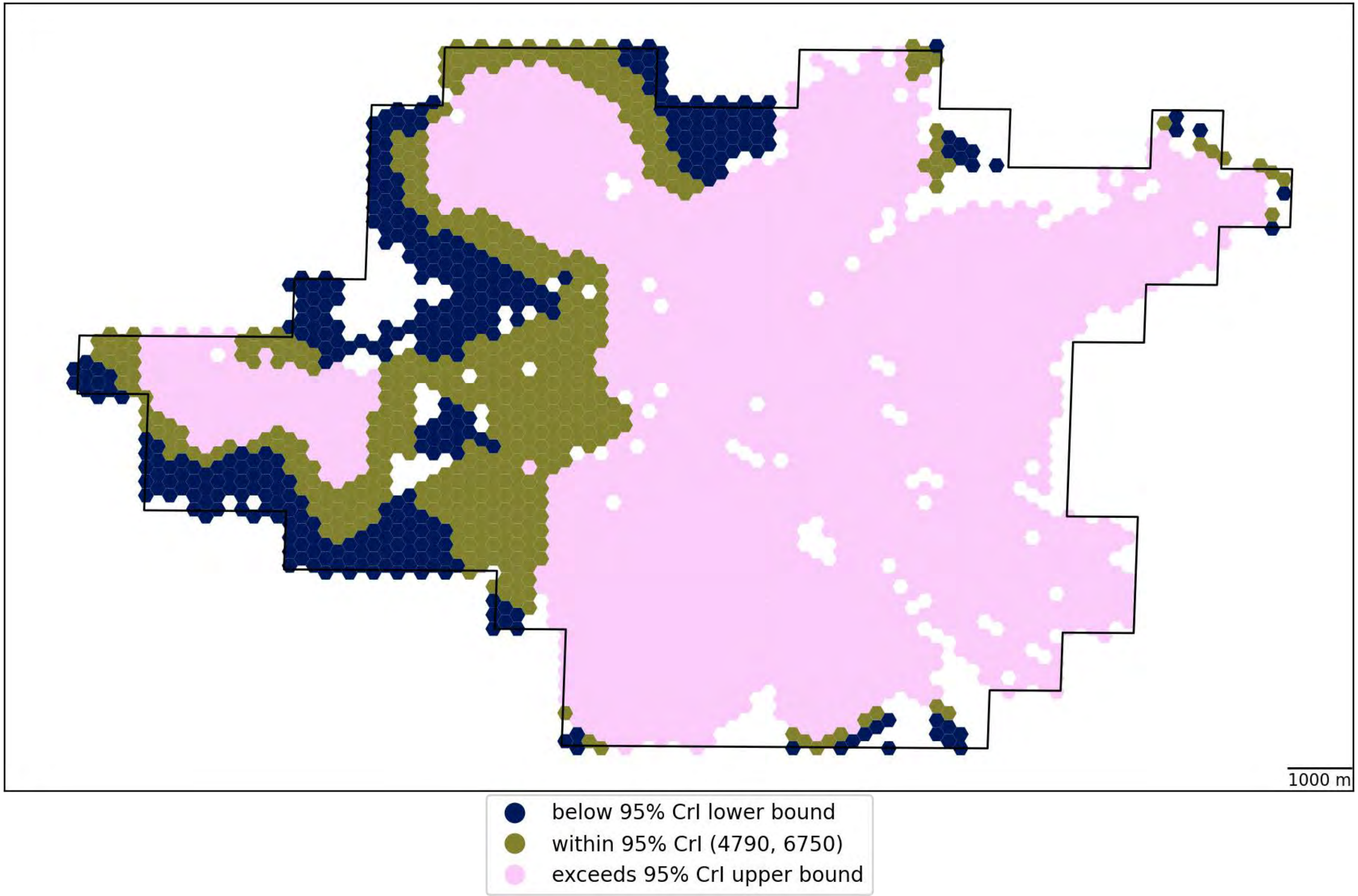

B: Estimated Mean 1000 m neighbourhood population per km² requirement for reaching the WHO's target of a ≥15% relative reduction in insufficient physical activity through walking

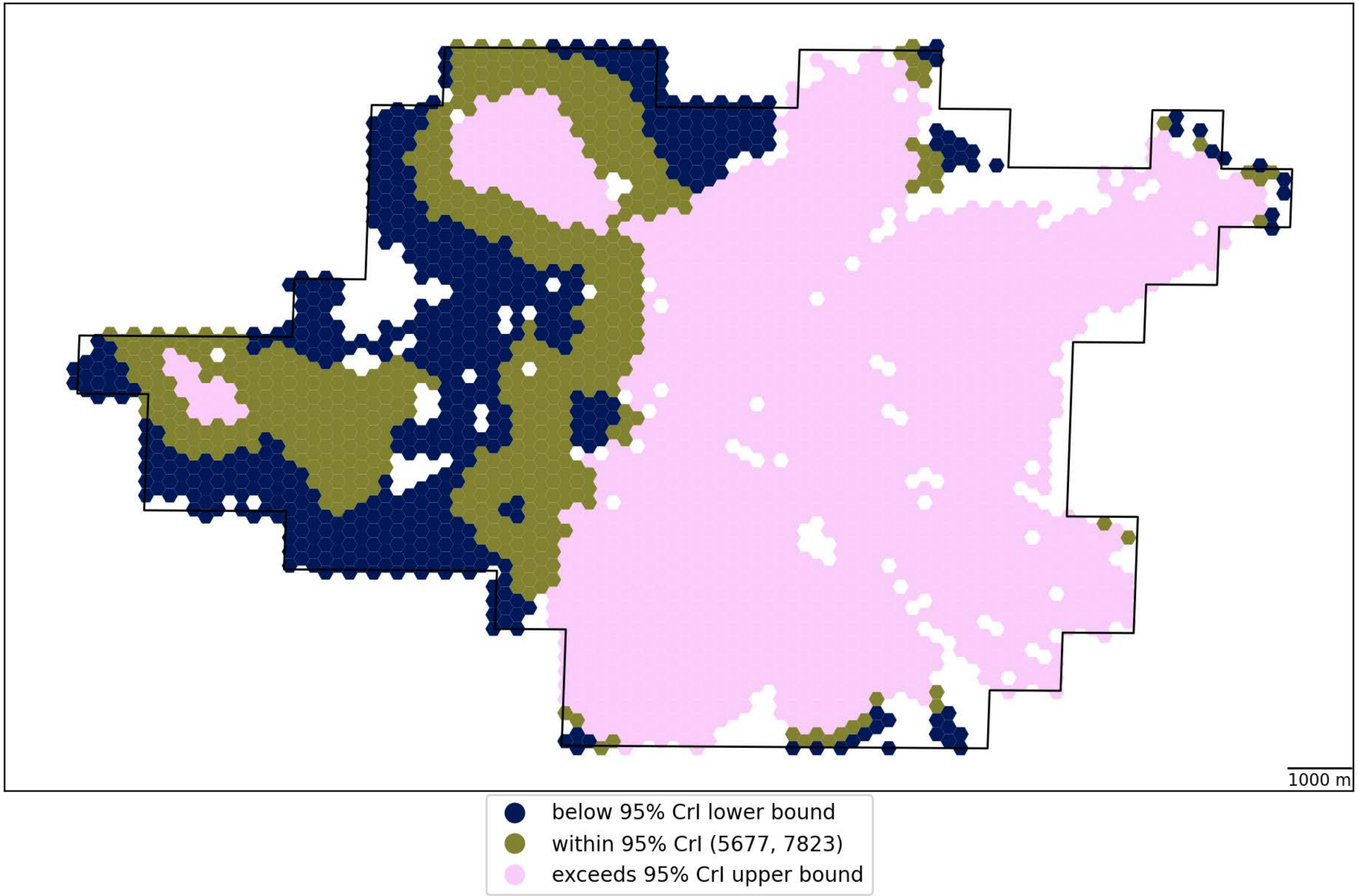

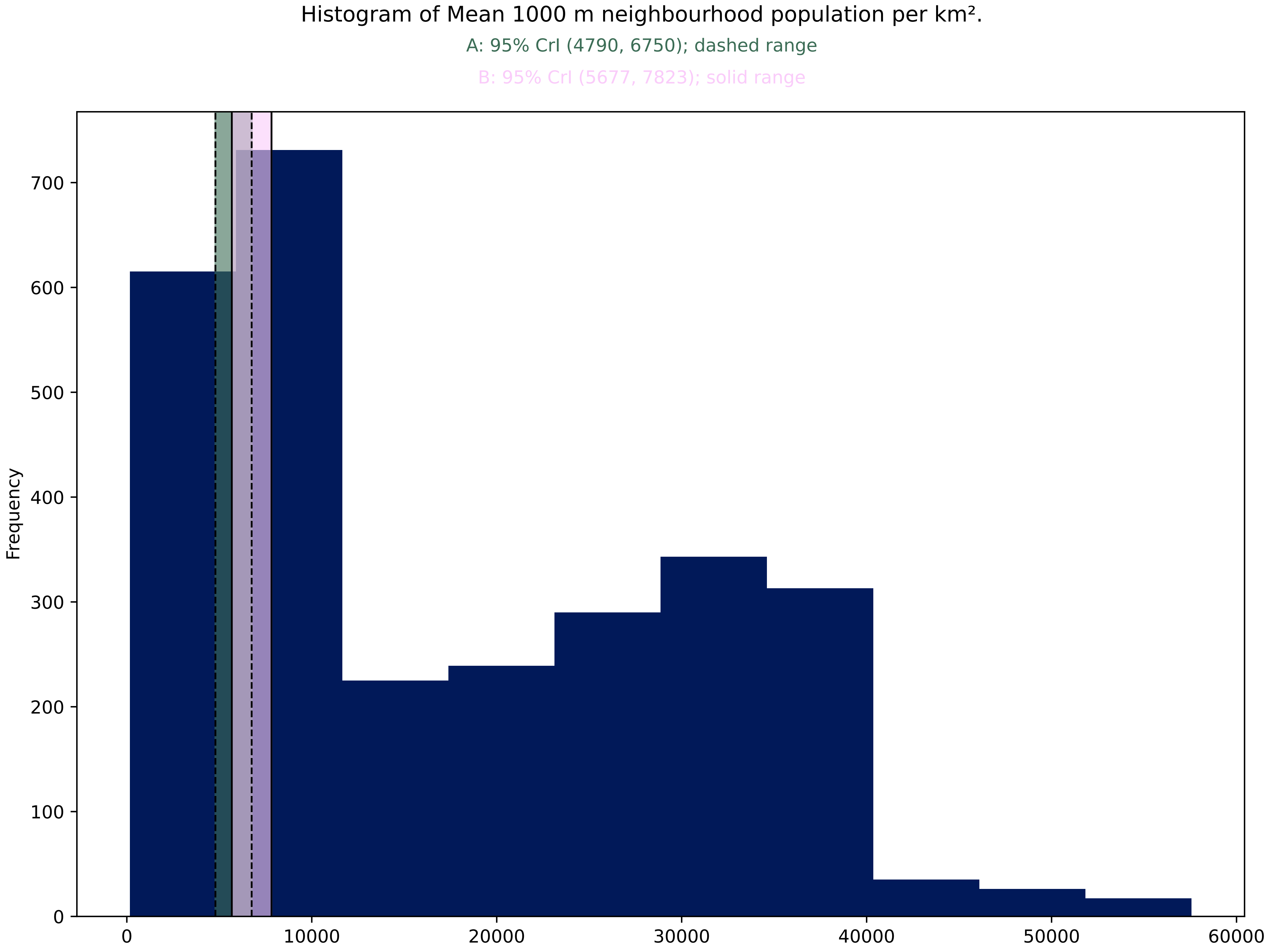
Histogram of Mean 1000 m neighbourhood population per km².
A: 95% CrI (4790, 6750); dashed range
B: 95% CrI (5677, 7823); solid range
Frequency
0
100
200
300
400
500
600
700
0
10000
20000
30000
40000
50000
60000

Mean 1000 m neighbourhood street intersections per km²

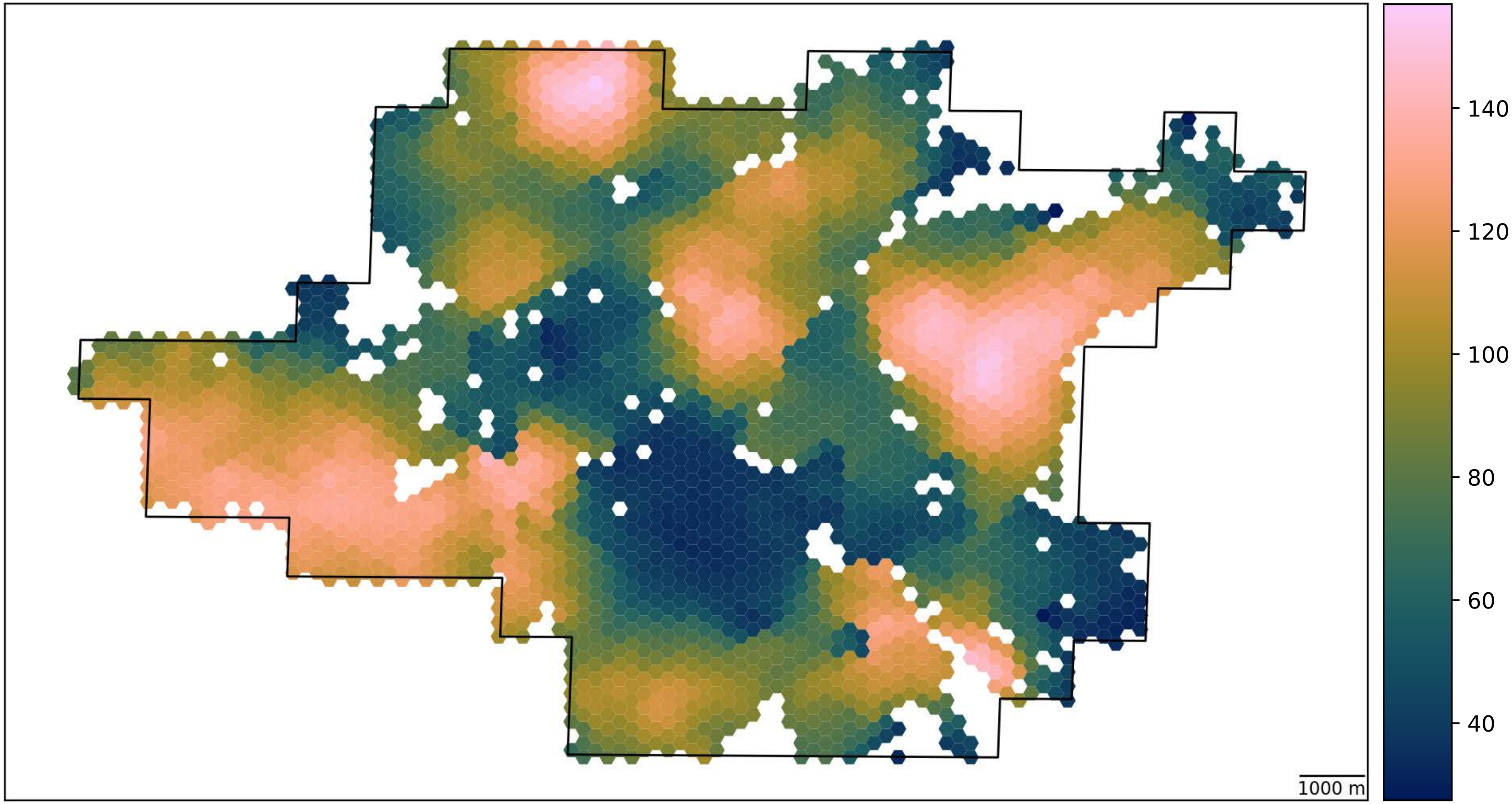

A: Estimated Mean 1000 m neighbourhood street intersections per km² requirement for ≥80% probability of engaging in walking for transport

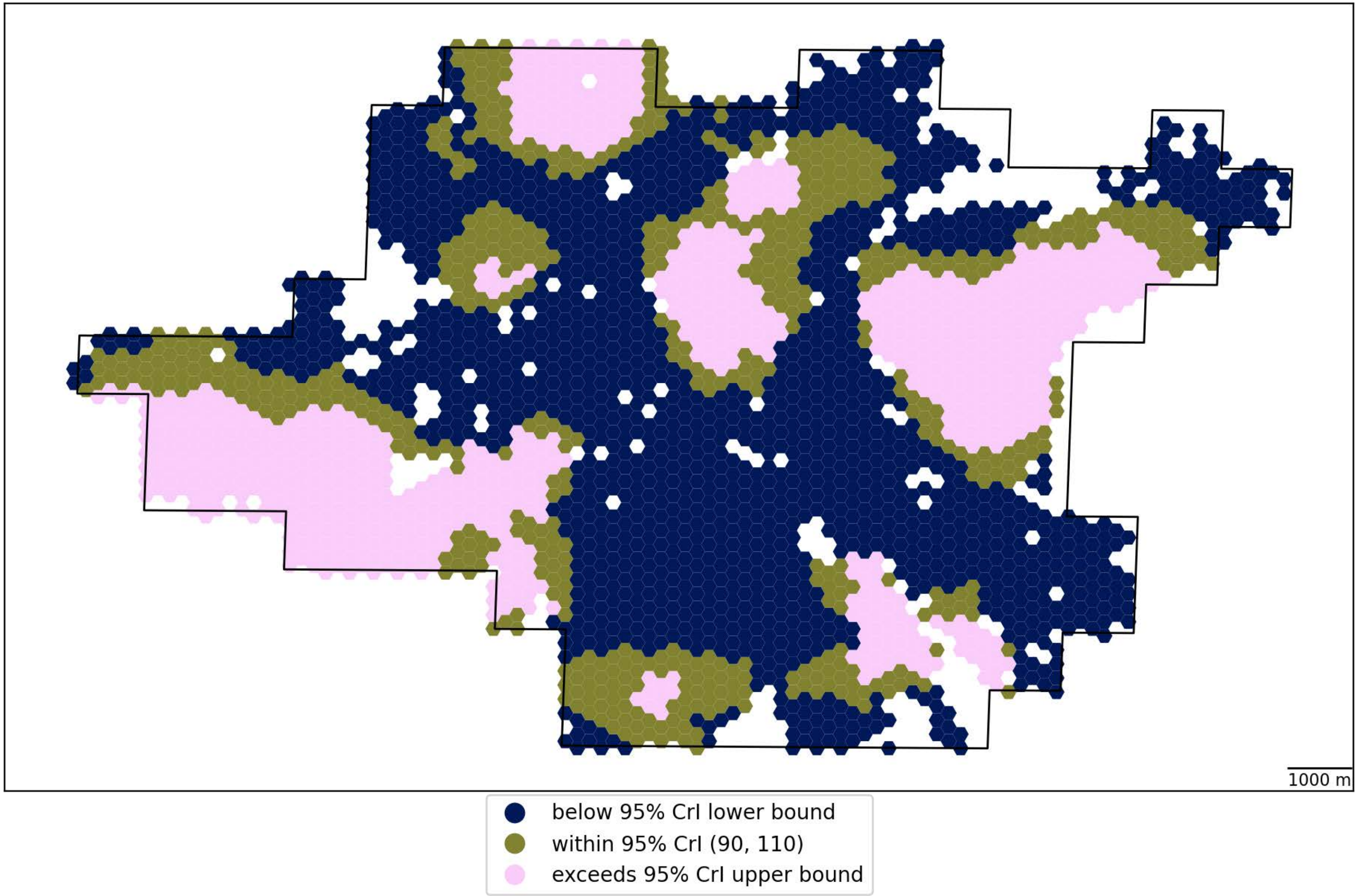

B: Estimated Mean 1000 m neighbourhood street intersections per km² requirement for reaching the WHO's target of a ≥15% relative reduction in insufficient physical activity through walking

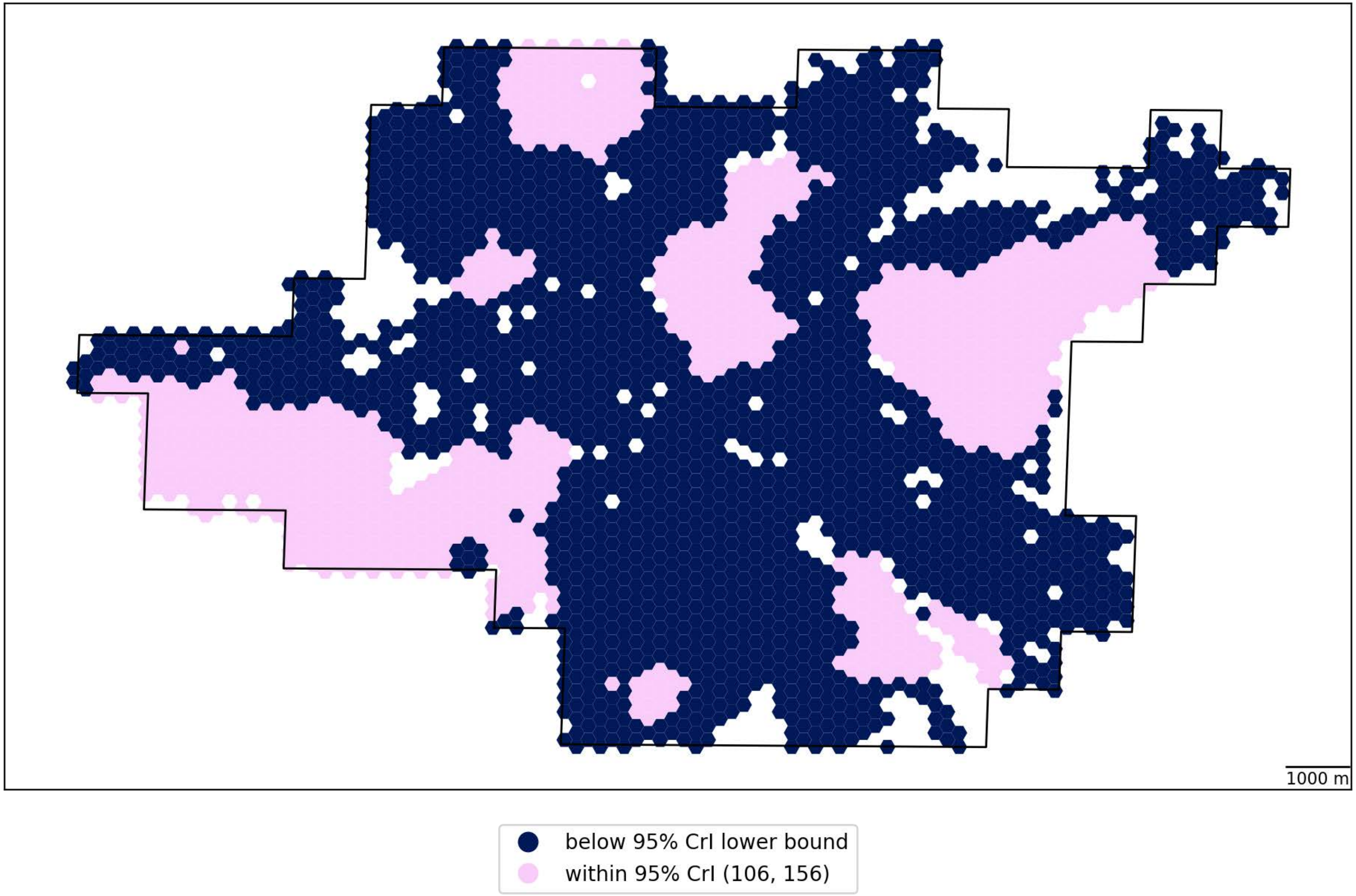

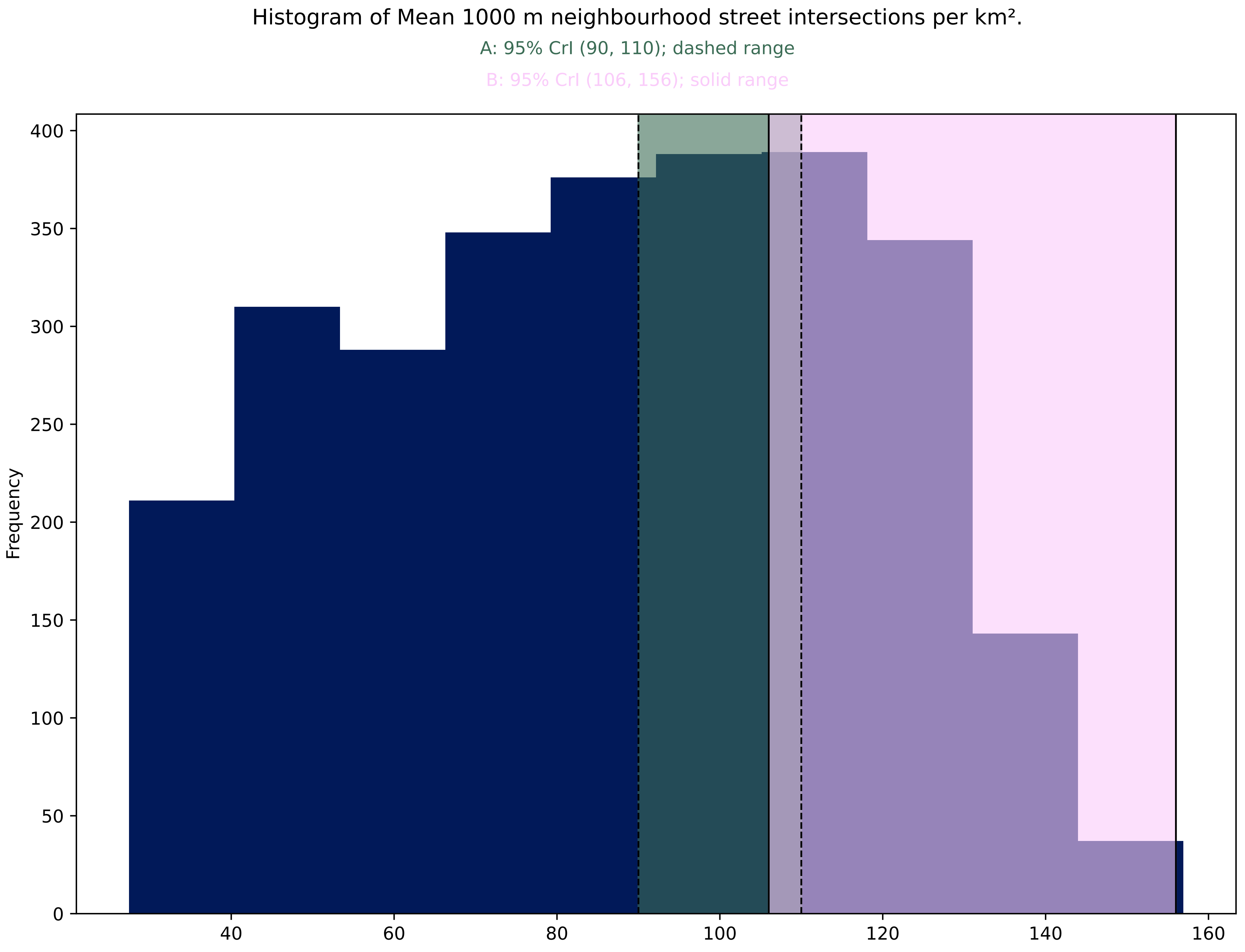
Histogram of Mean 1000 m neighbourhood street intersections per km².
A: 95% CrI (90, 110); dashed range
B: 95% CrI (106, 156); solid range
Frequency
0
50
100
150
200
250
300
350
400
40
60
80
100
120
140
160

Distance to nearest public transport stops (m; up to 500m)

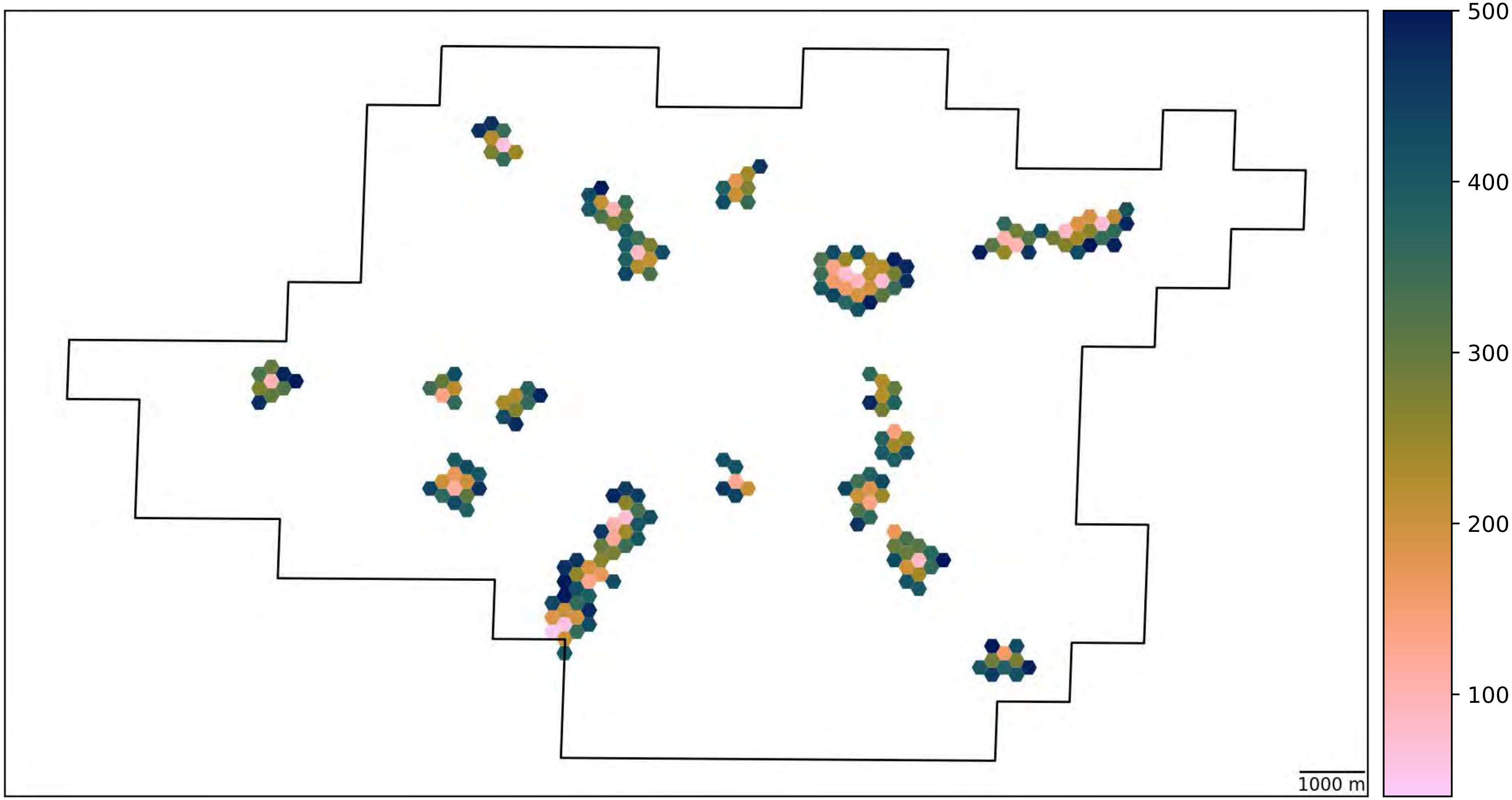

distances: Estimated Distance to nearest public transport stops (m; up to 500m) requirement for distances to destinations, measured up to a maximum distance target threshold of 500 metres

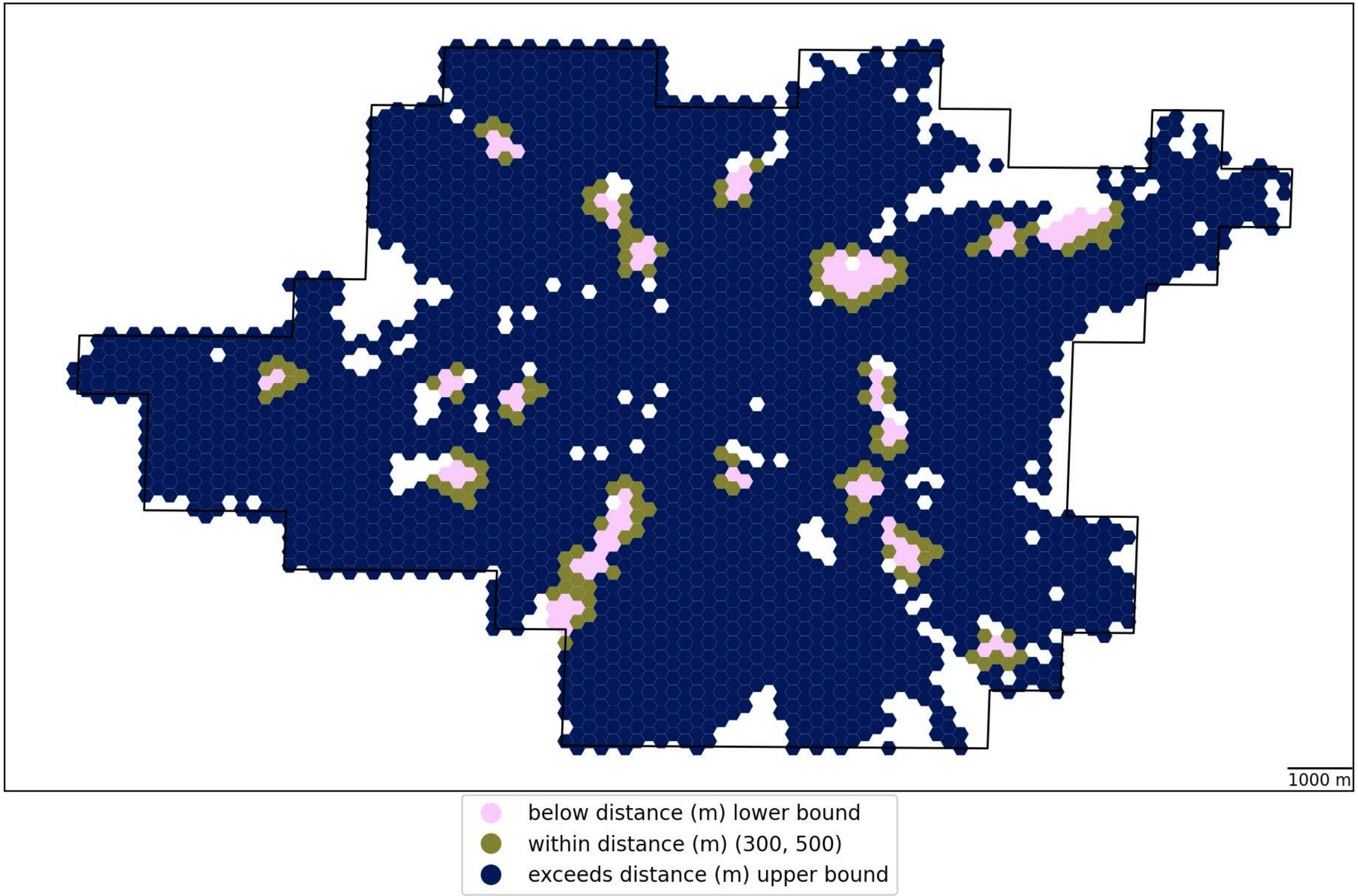

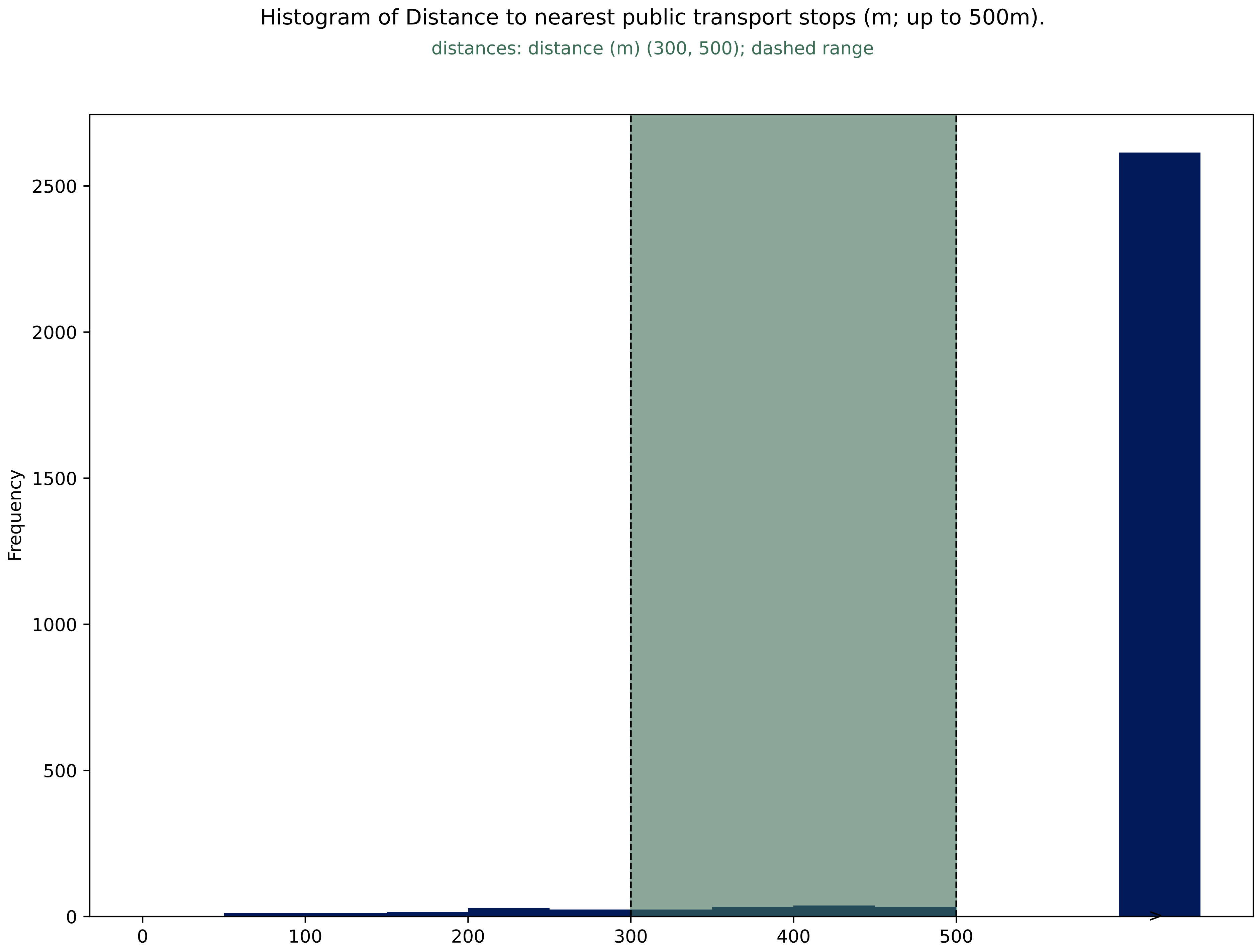

Histogram of Distance to nearest public transport stops (m; up to 500m).
distances: distance (m) (300, 500); dashed range
Frequency
2500
2000
1500
1000
500
0
0
100
200
300
400
500
>

Distance to nearest park (m; up to 500m)

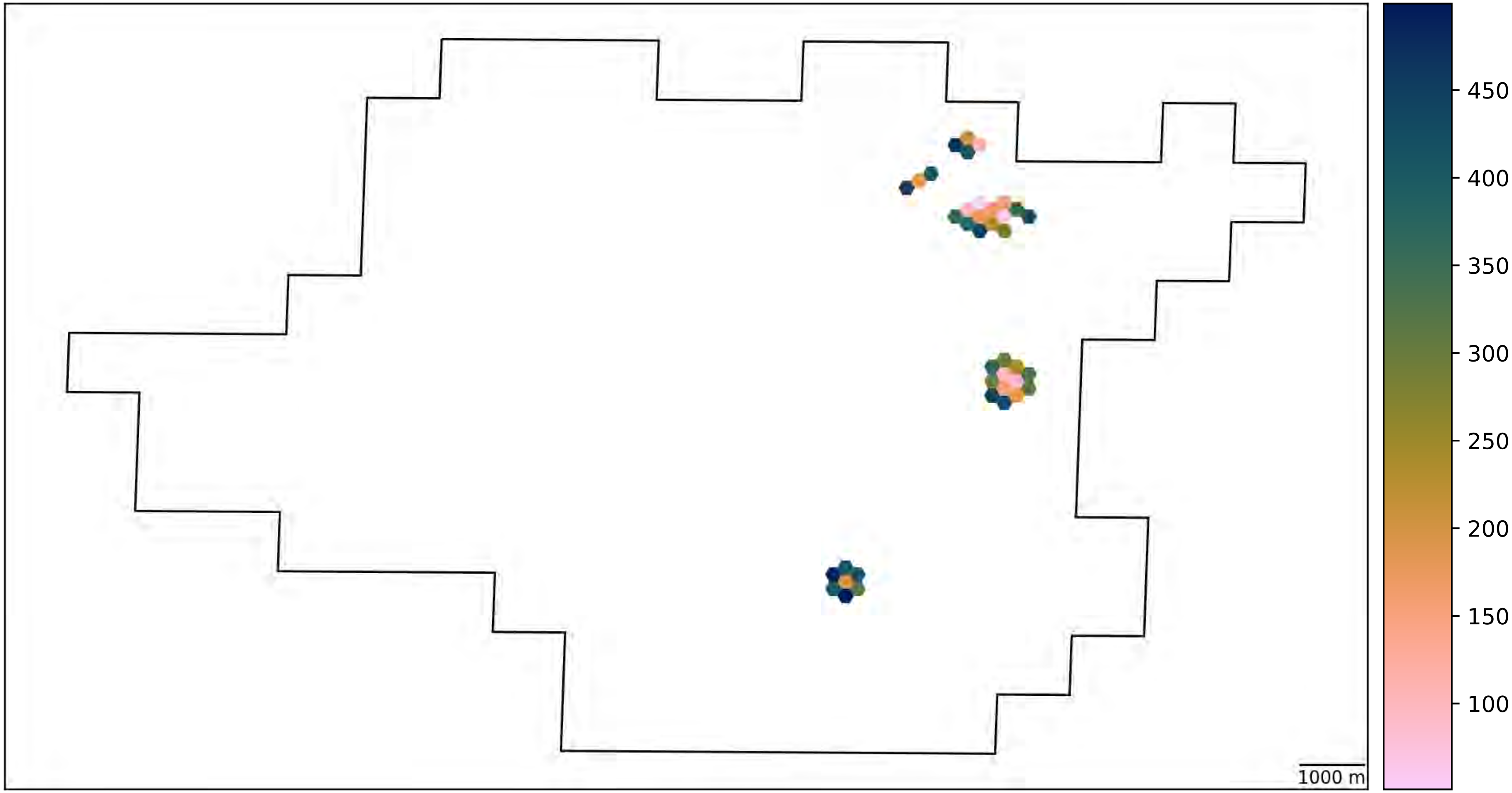

distances: Estimated Distance to nearest park (m; up to 500m) requirement for distances to destinations, measured up to a maximum distance target threshold of 500 metres

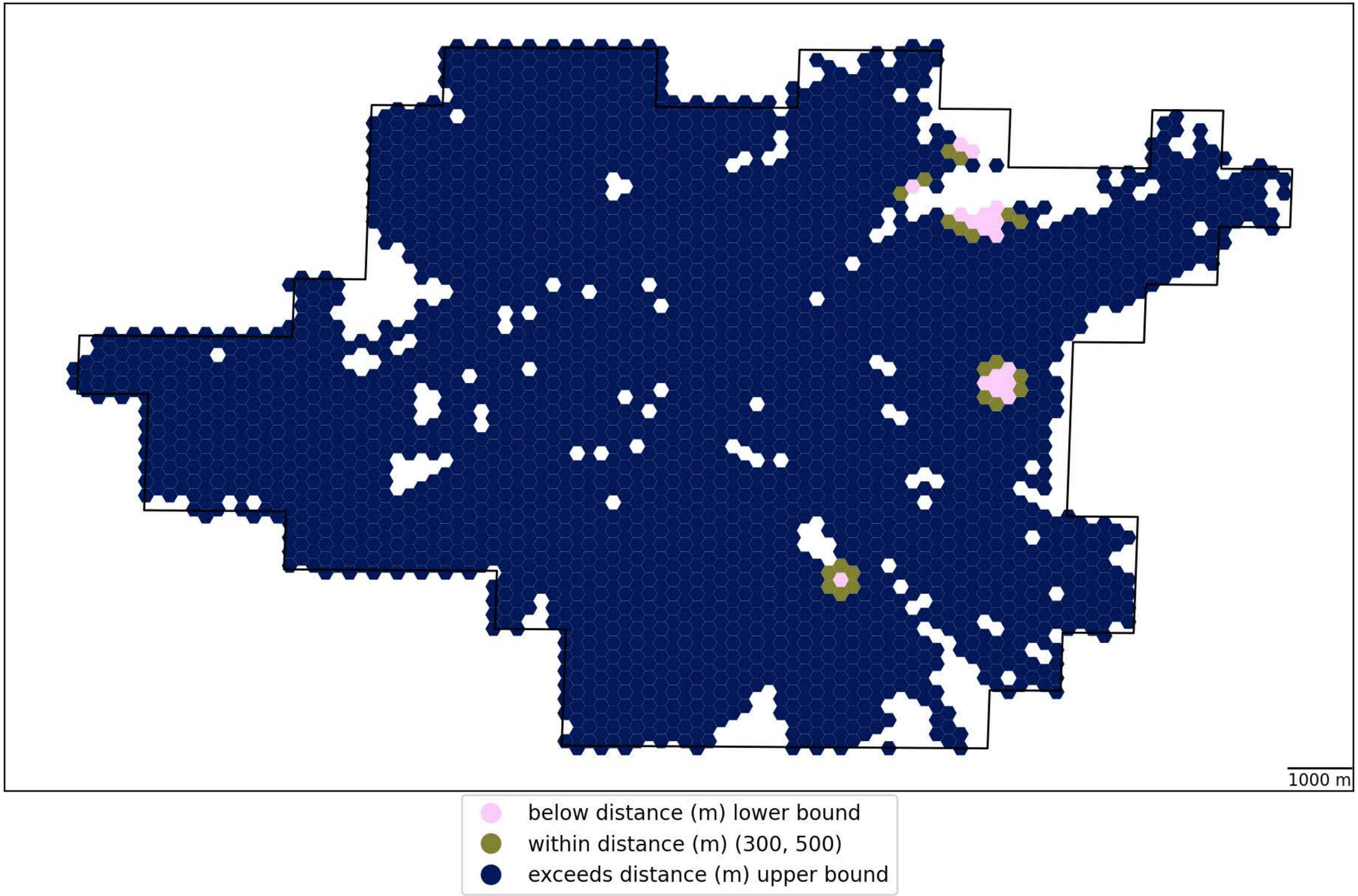

Histogram of Distance to nearest park (m; up to 500m).

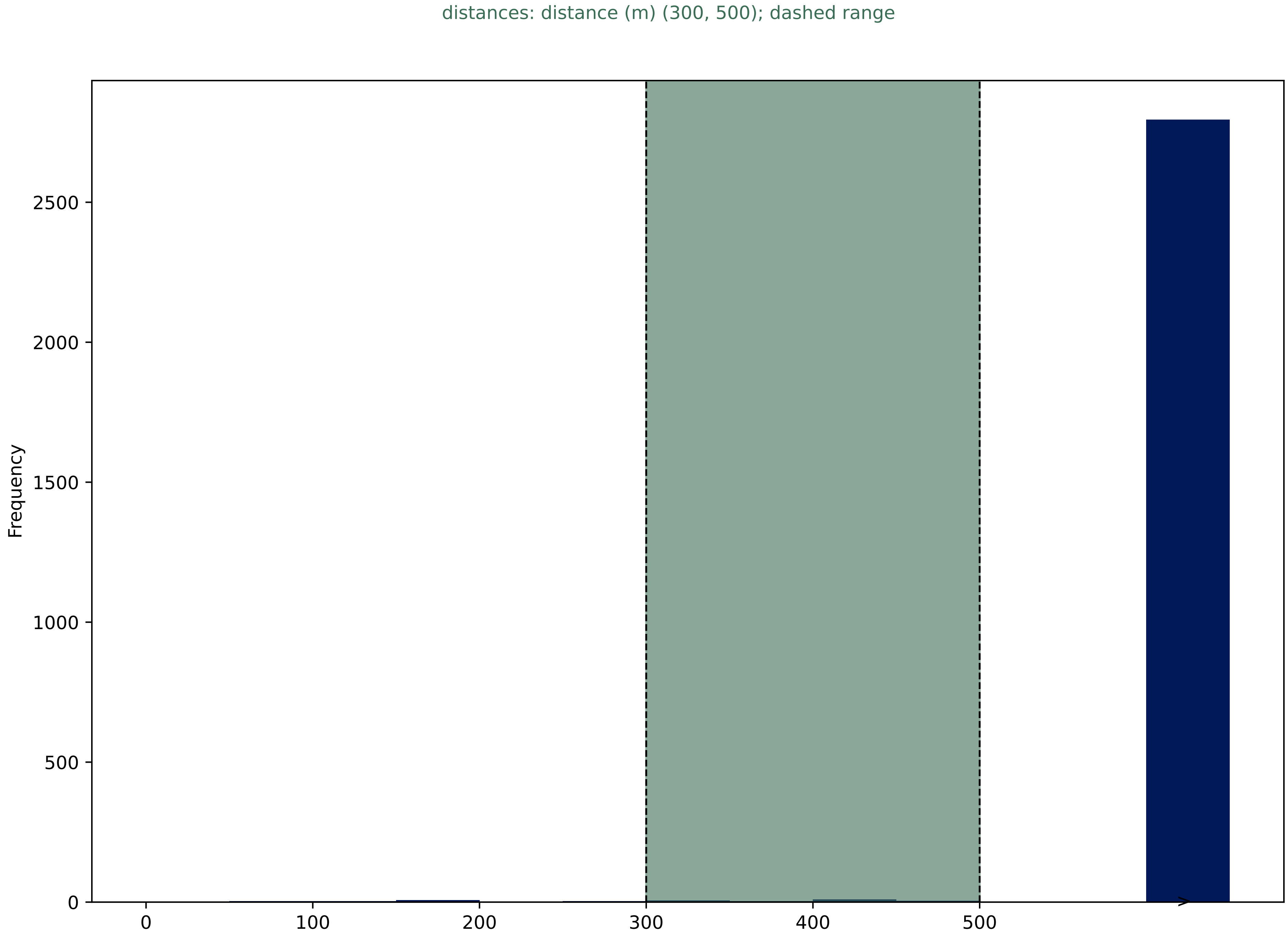

America, North, Mexico, Mexico City

Satellite imagery of urban study region (Bing)

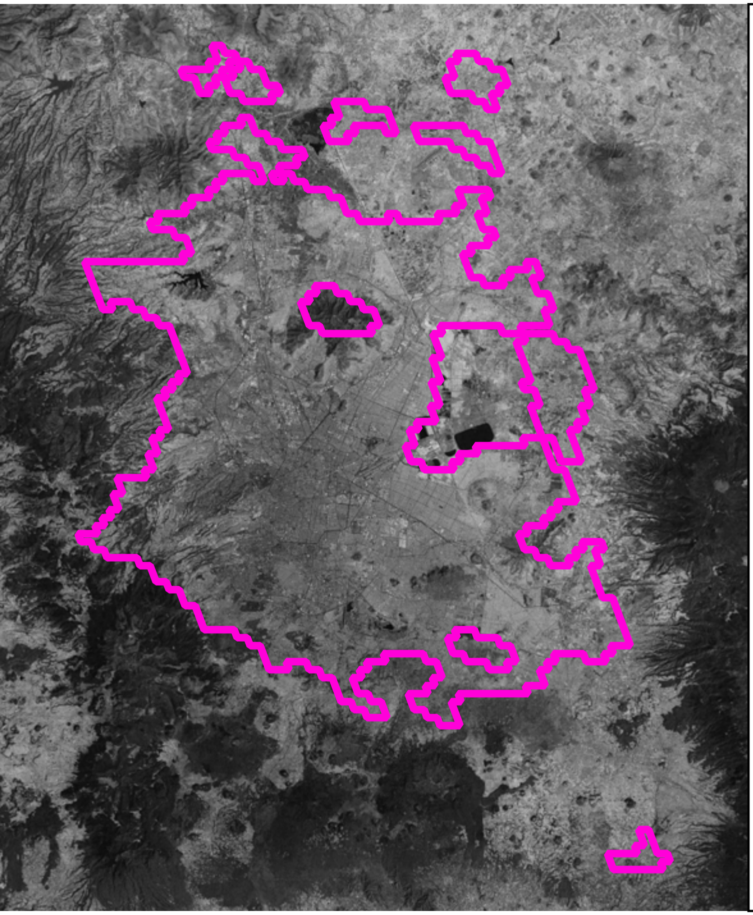

Walkability, relative to city

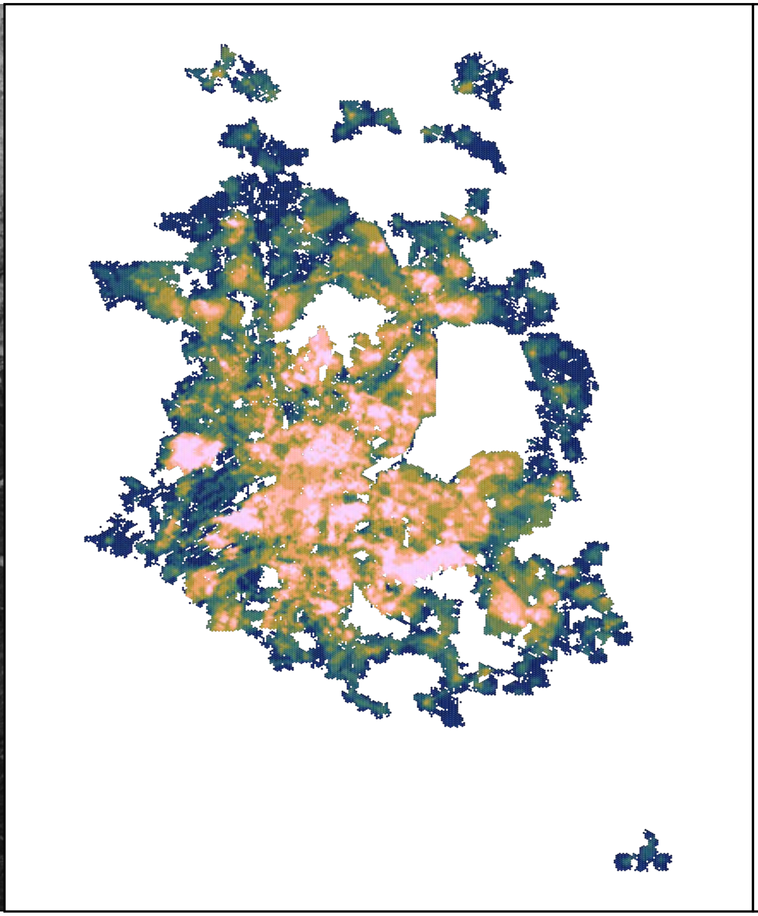

Walkability, relative to 25 global cities

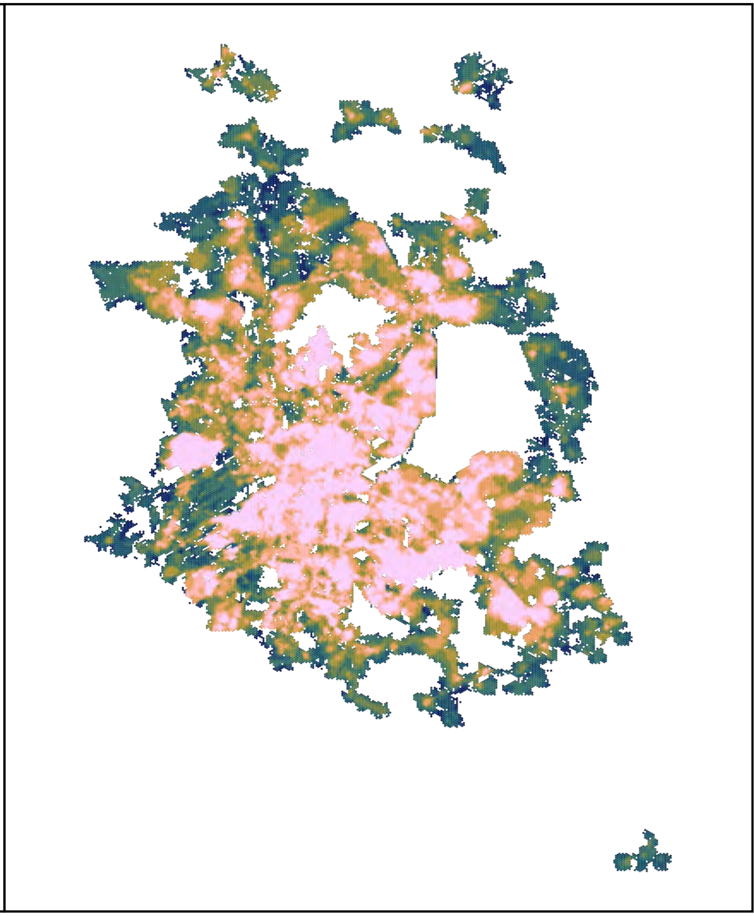

Urban boundary

0 30 60 km

Walkability score

- <-3
- -3 to -2
- -2 to -1
- -1 to 0
- 0 to 1
- 1 to 2
- 2 to 3
- ≥3

Walkability relative to all cities by component variables (2D histograms), and overall (histogram)

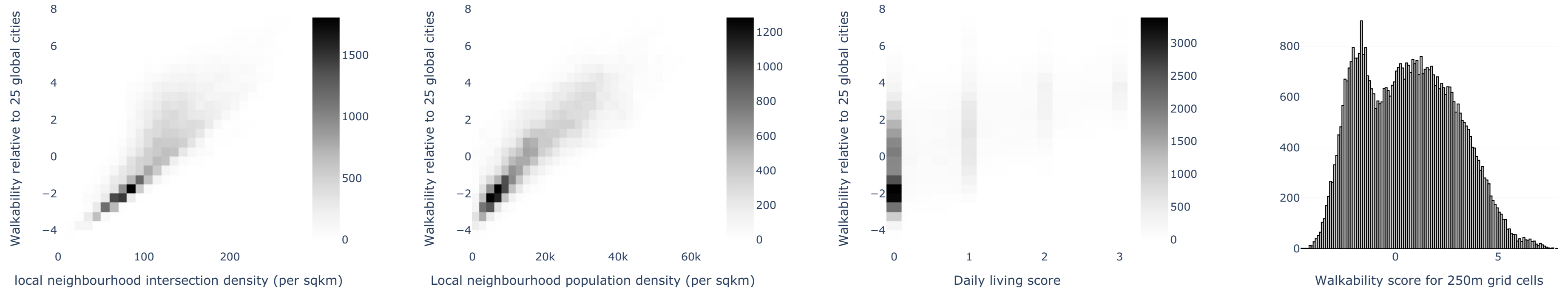

Mean 1000 m neighbourhood population per km²

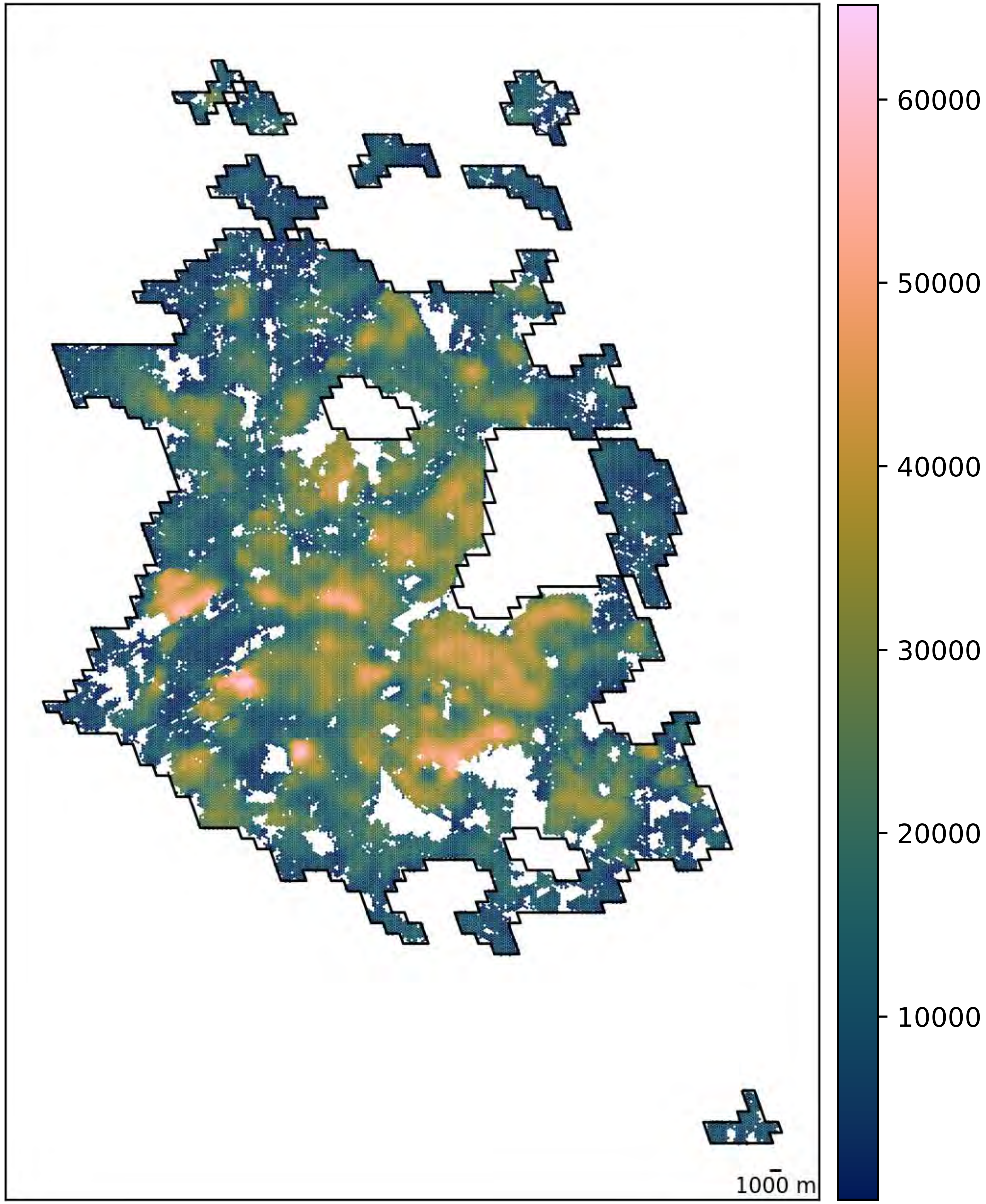

A: Estimated Mean 1000 m neighbourhood population per km² requirement for ≥80% probability of engaging in walking for transport

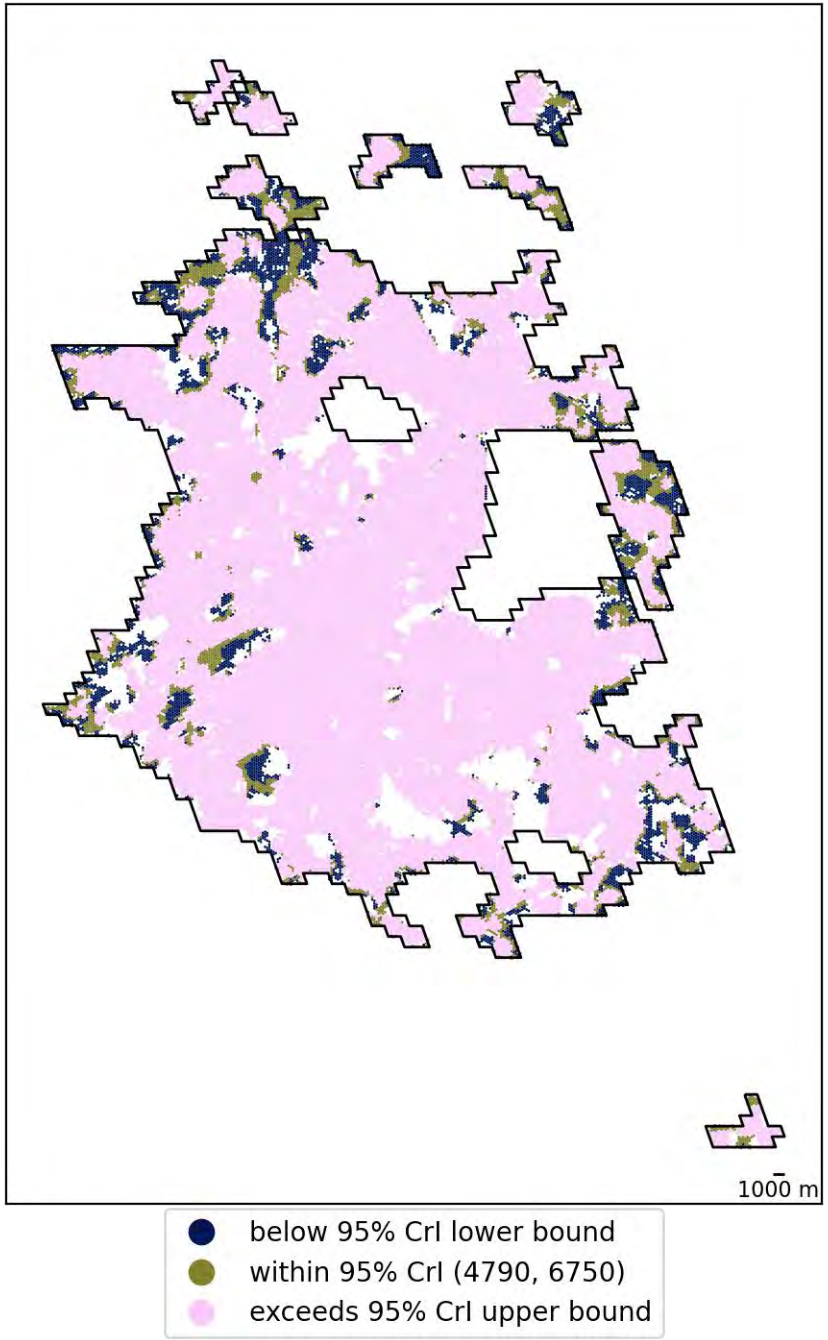

B: Estimated Mean 1000 m neighbourhood population per km² requirement for reaching the WHO's target of a ≥15% relative reduction in insufficient physical activity through walking

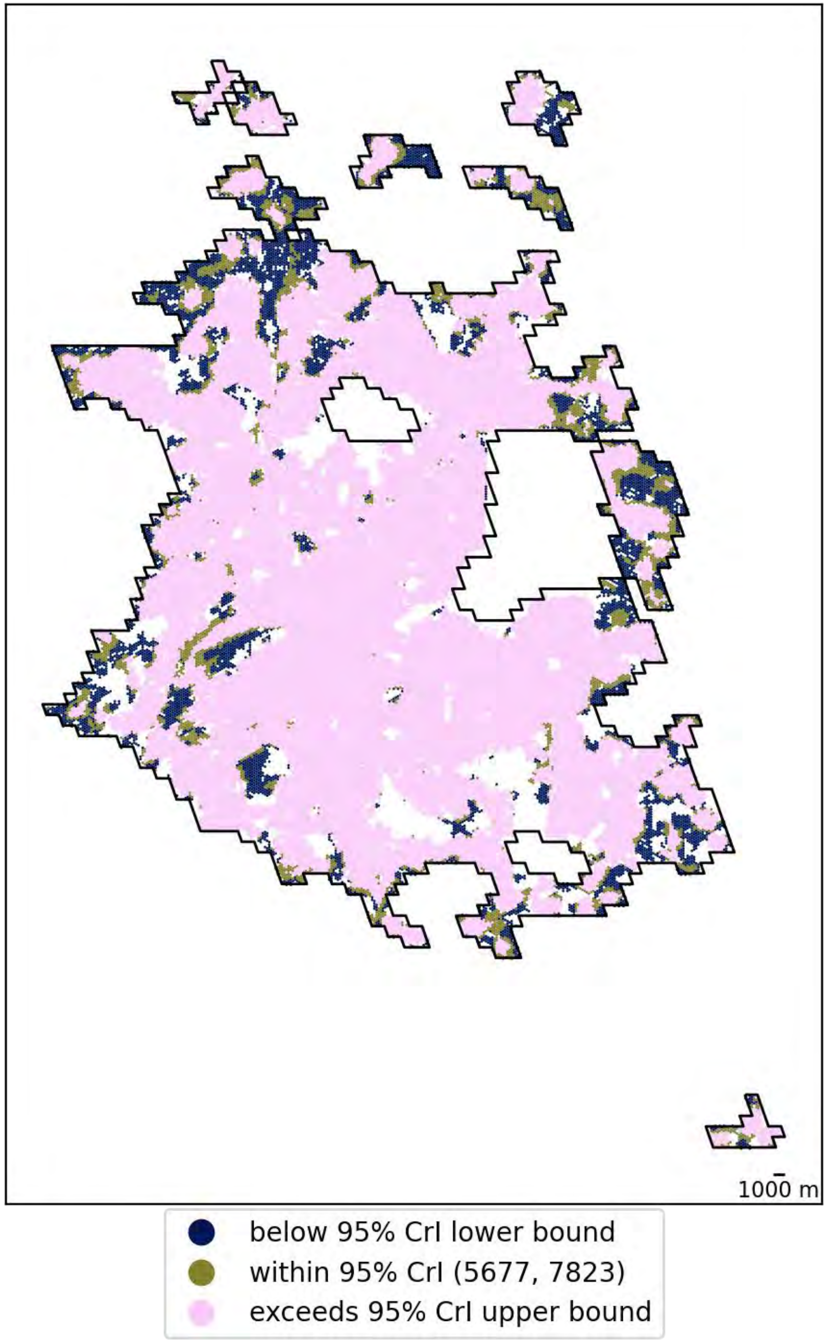

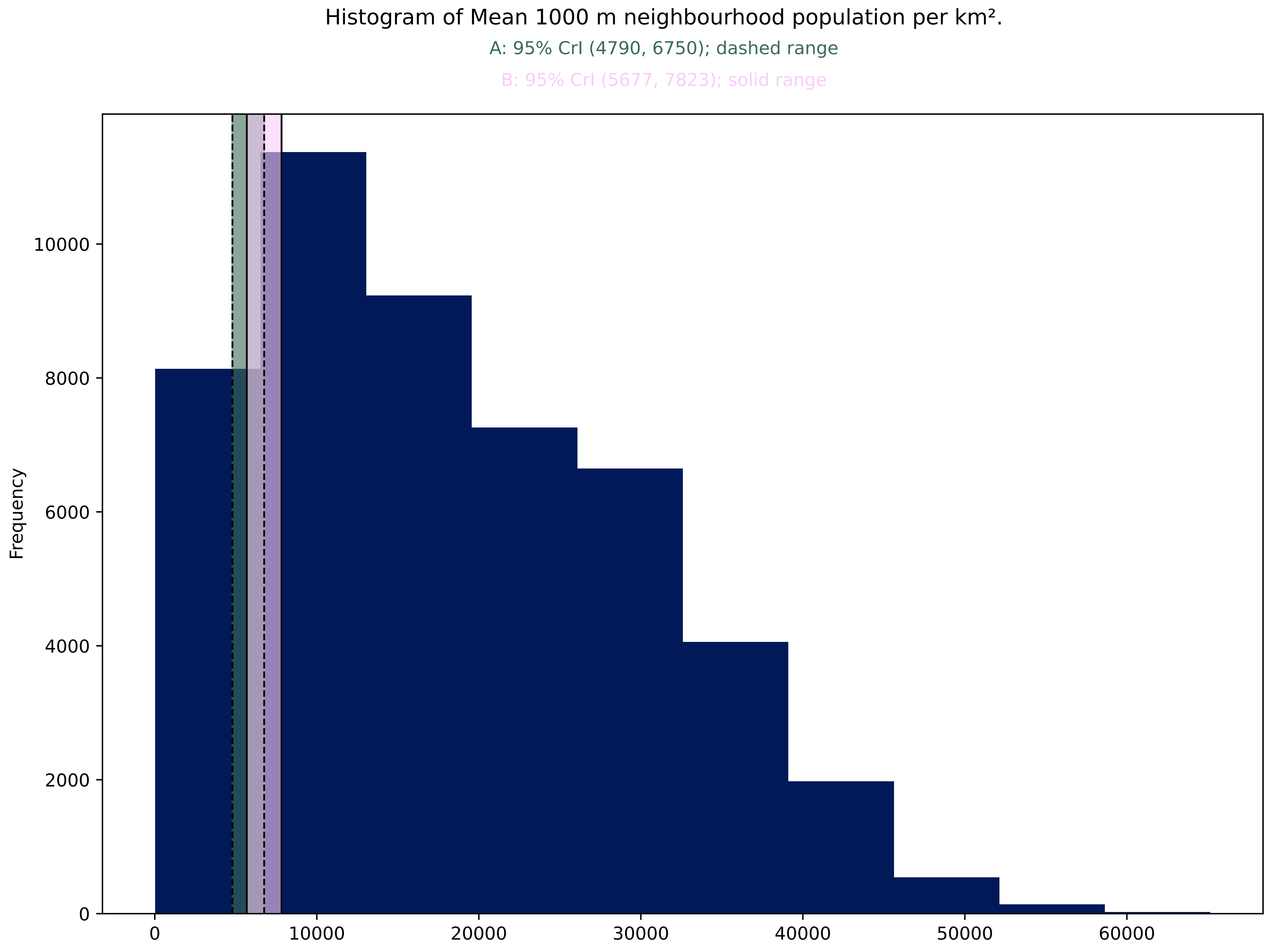
Histogram of Mean 1000 m neighbourhood population per km².
A: 95% CrI (4790, 6750); dashed range
B: 95% CrI (5677, 7823); solid range
Frequency
0
2000
4000
6000
8000
10000
0
10000
20000
30000
40000
50000
60000

Mean 1000 m neighbourhood street intersections per km²

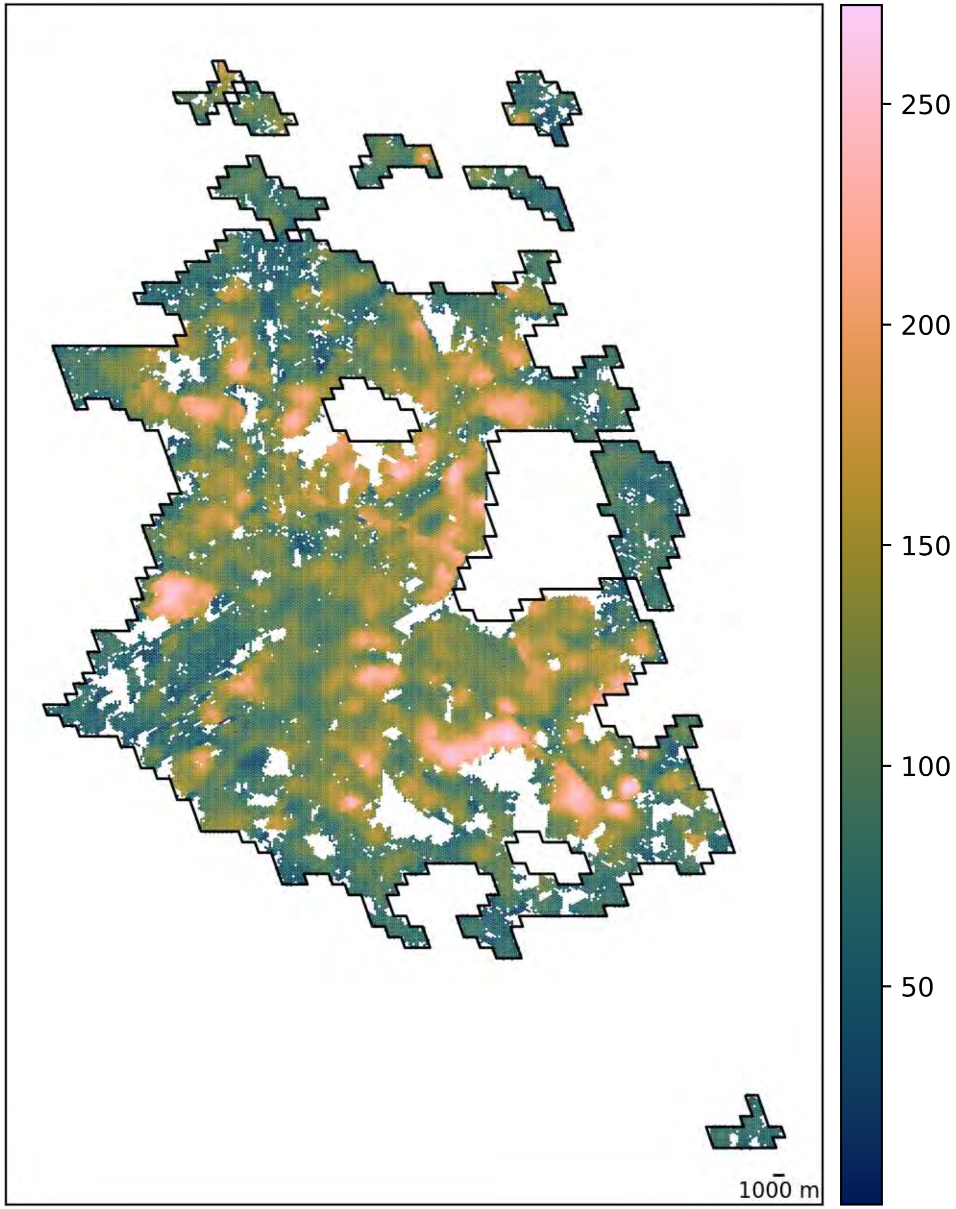

A: Estimated Mean 1000 m neighbourhood street intersections per km² requirement for ≥80% probability of engaging in walking for transport

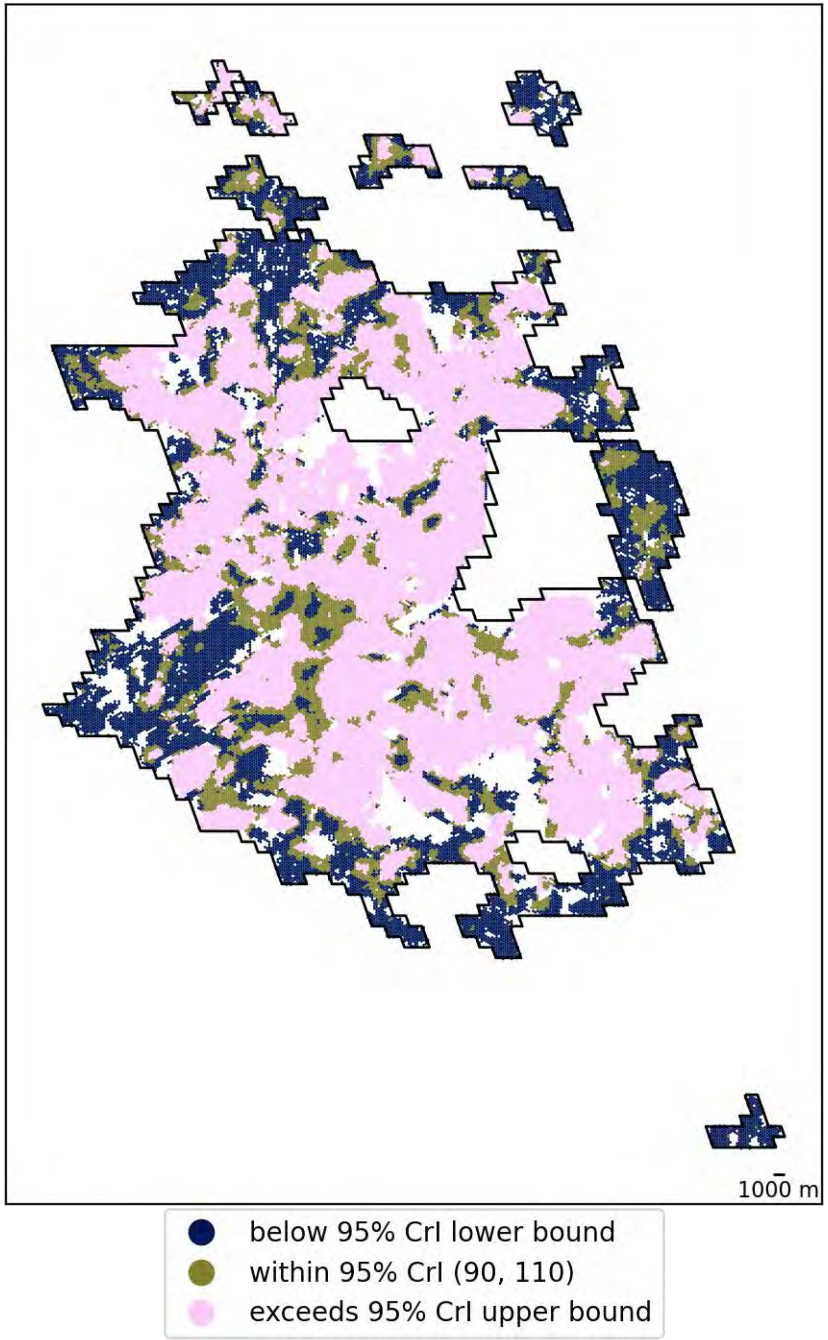

B: Estimated Mean 1000 m neighbourhood street intersections per km² requirement for reaching the WHO's target of a ≥15% relative reduction in insufficient physical activity through walking

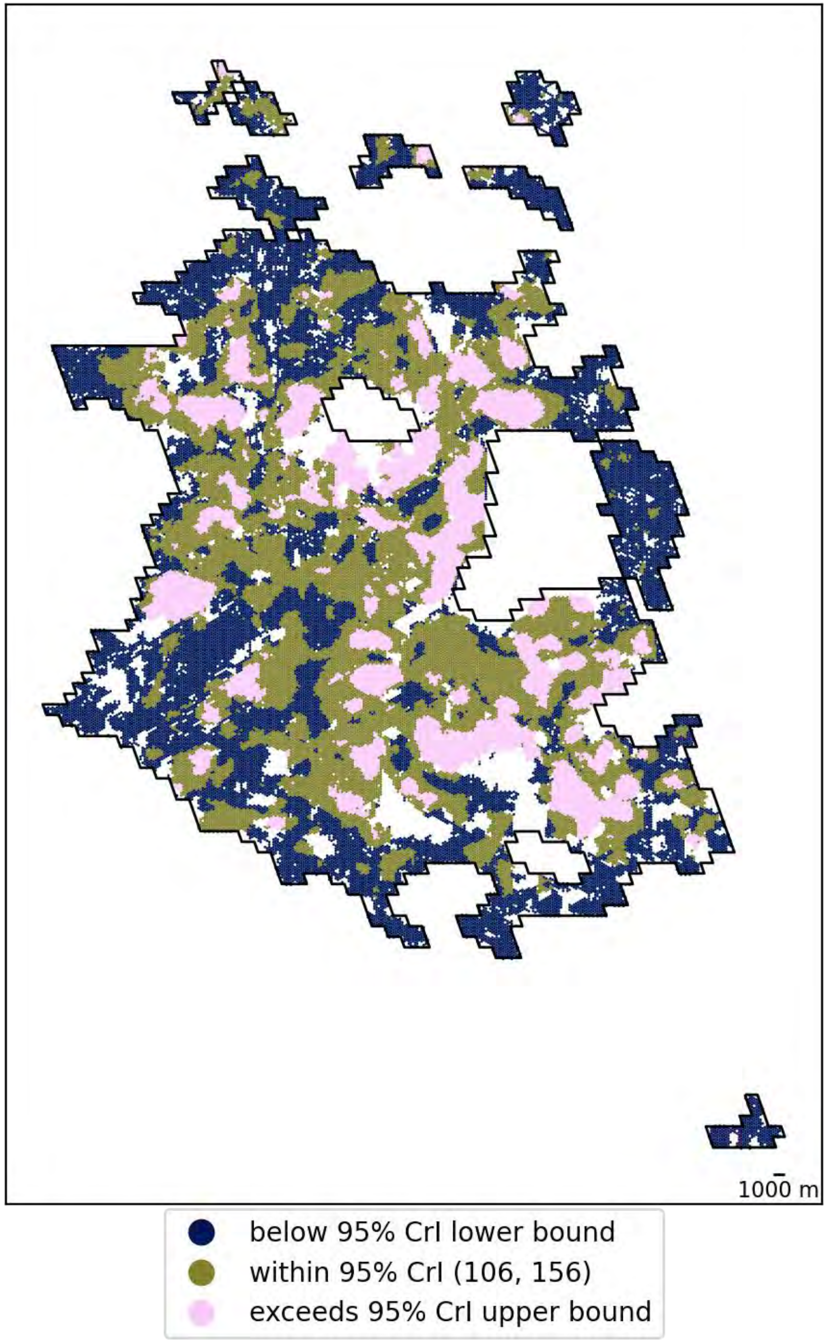

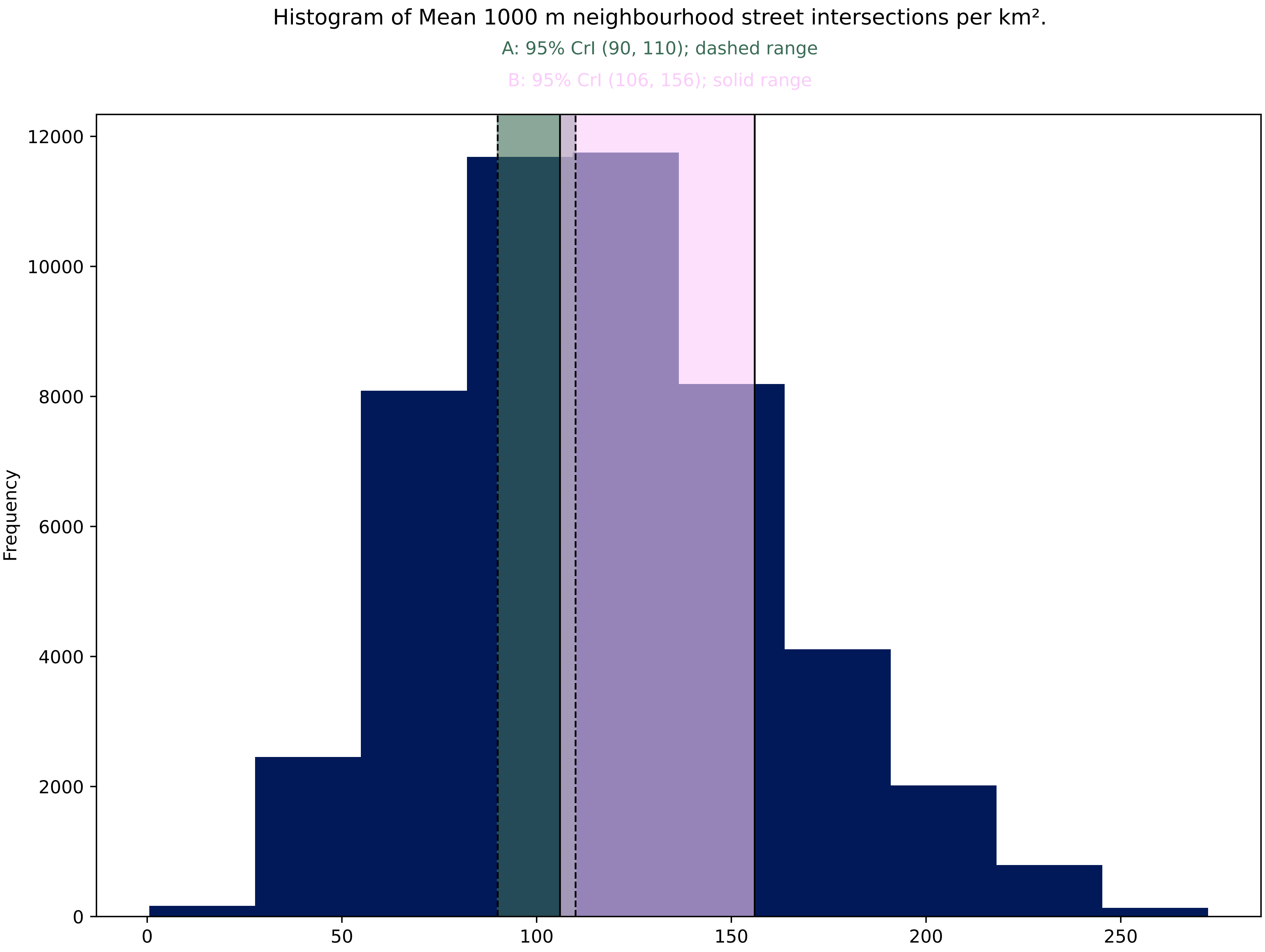
Histogram of Mean 1000 m neighbourhood street intersections per km².
A: 95% CrI (90, 110); dashed range
B: 95% CrI (106, 156); solid range
Frequency
0
2000
4000
6000
8000
10000
12000
0
50
100
150
200
250

Distance to nearest public transport stops (m; up to 500m)

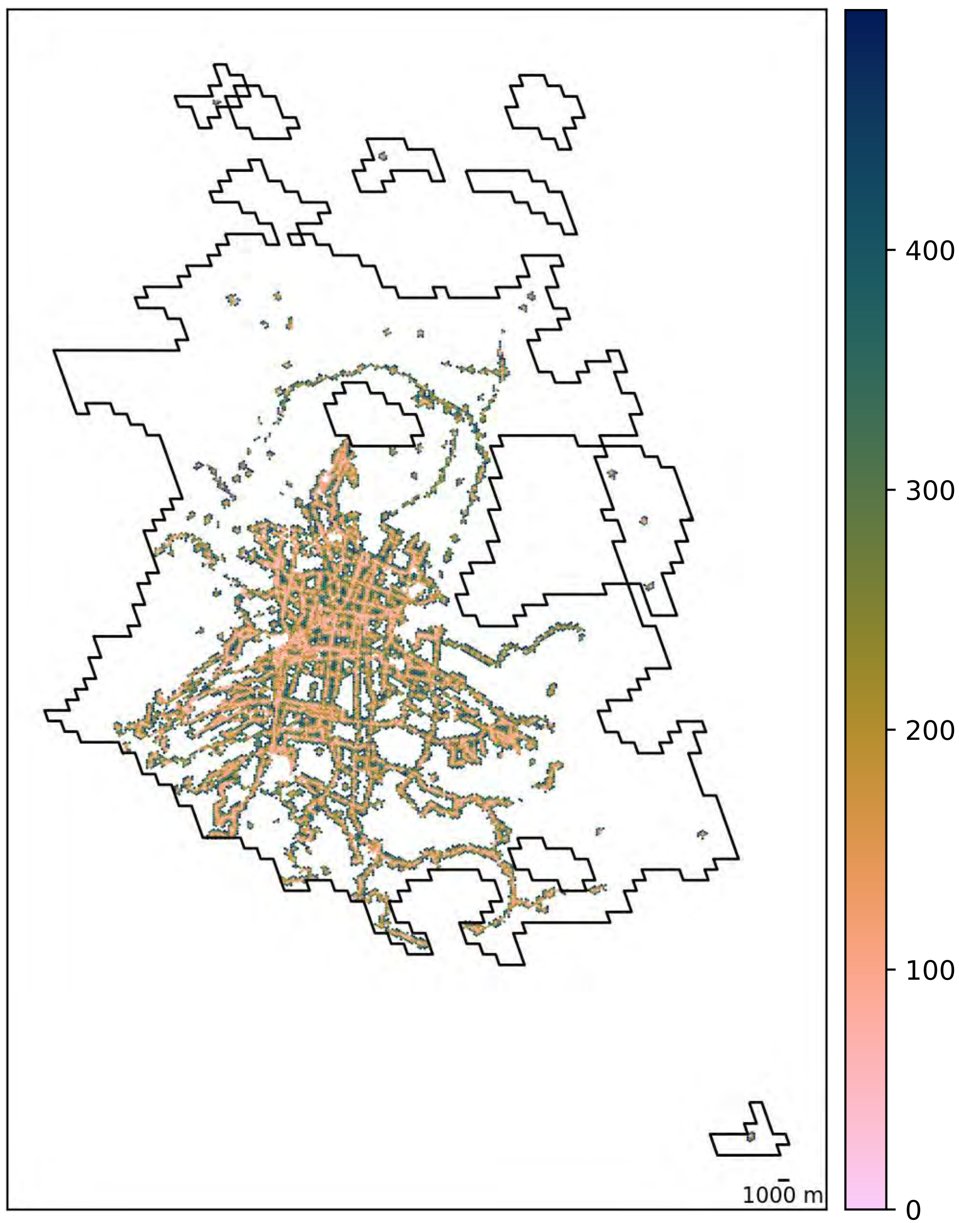

distances: Estimated Distance to nearest public transport stops (m; up to 500m) requirement for distances to destinations, measured up to a maximum distance target threshold of 500 metres

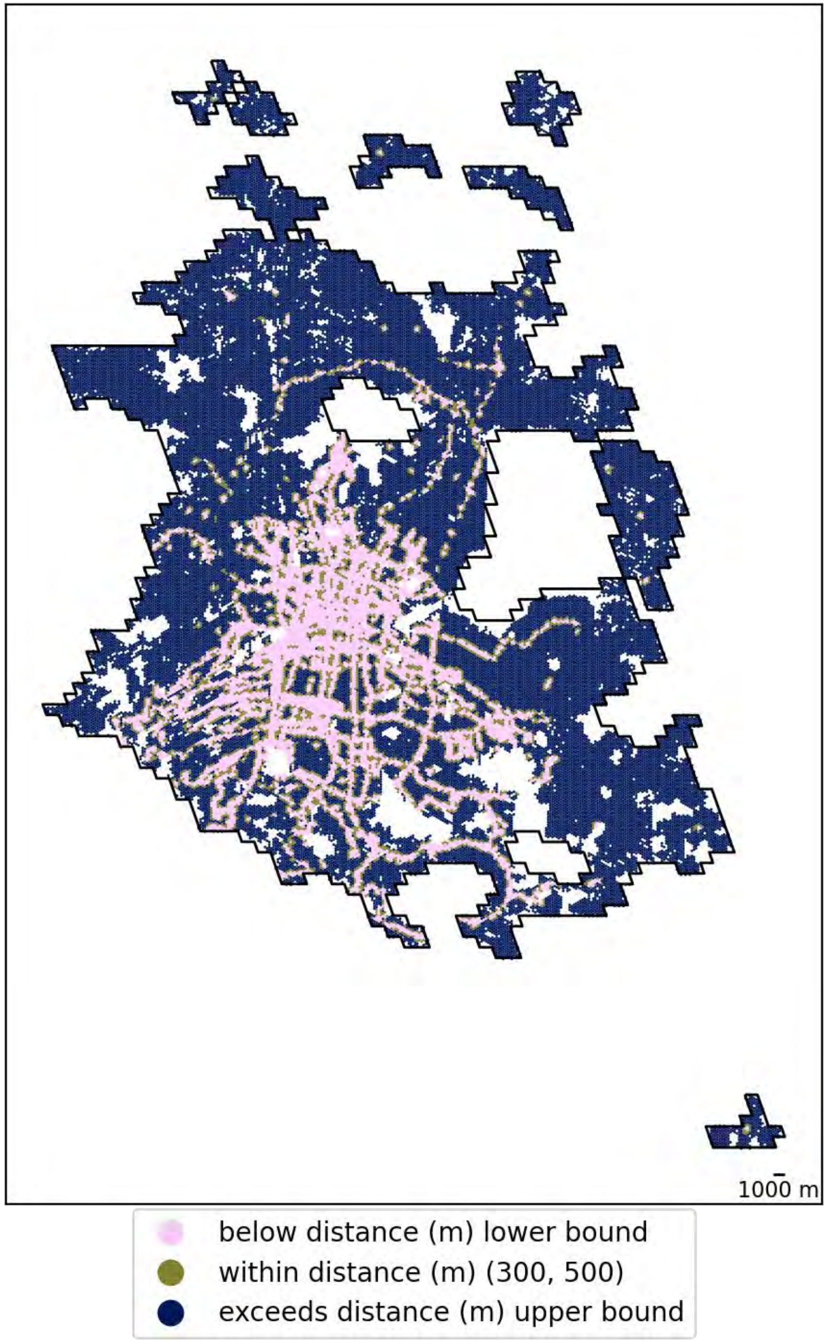

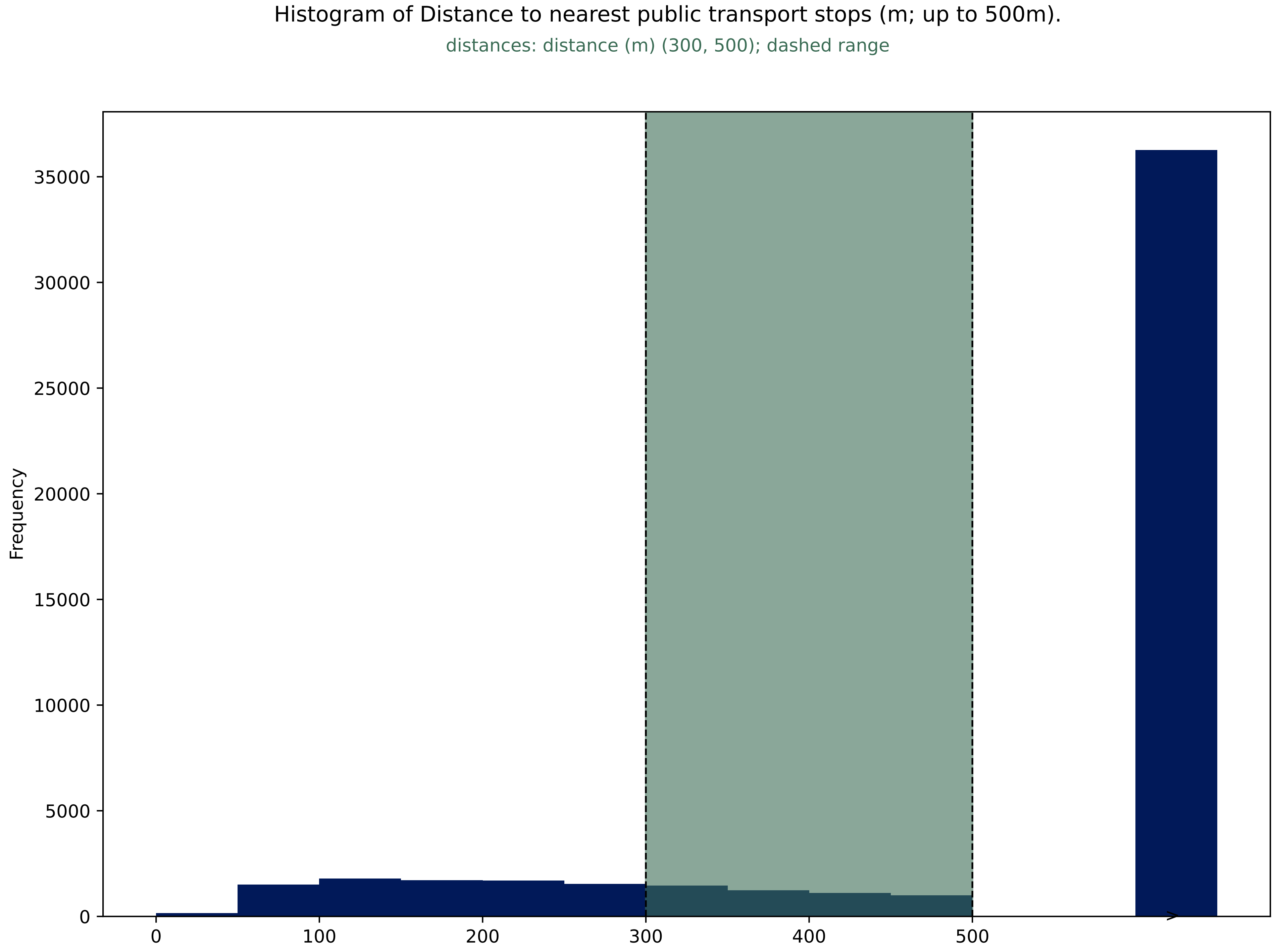
Histogram of Distance to nearest public transport stops (m; up to 500m).
distances: distance (m) (300, 500); dashed range
Frequency
35000
30000
25000
20000
15000
10000
5000
0
0
100
200
300
400
500
>

Distance to nearest park (m; up to 500m)

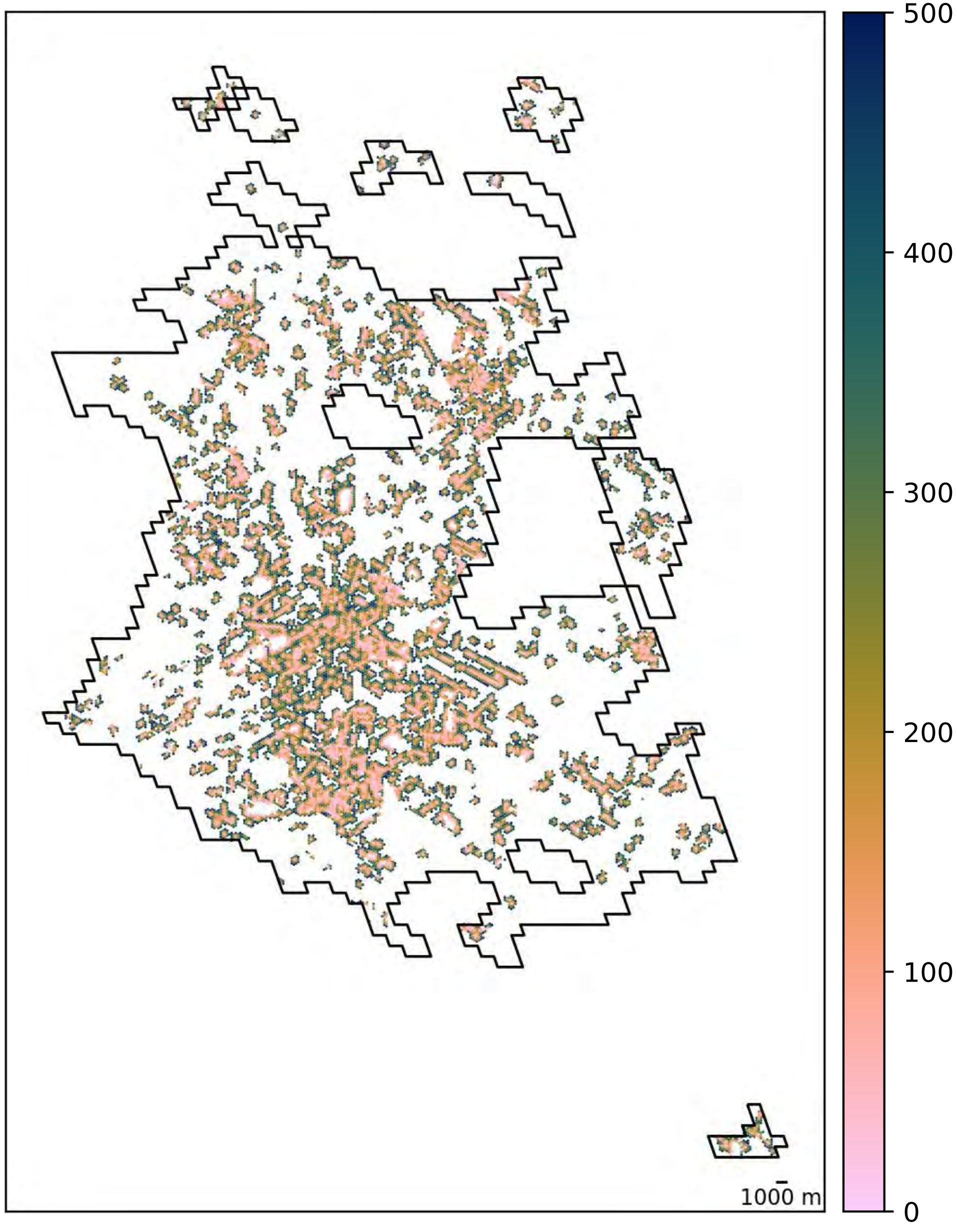

distances: Estimated Distance to nearest park (m; up to 500m) requirement for distances to destinations, measured up to a maximum distance target threshold of 500 metres

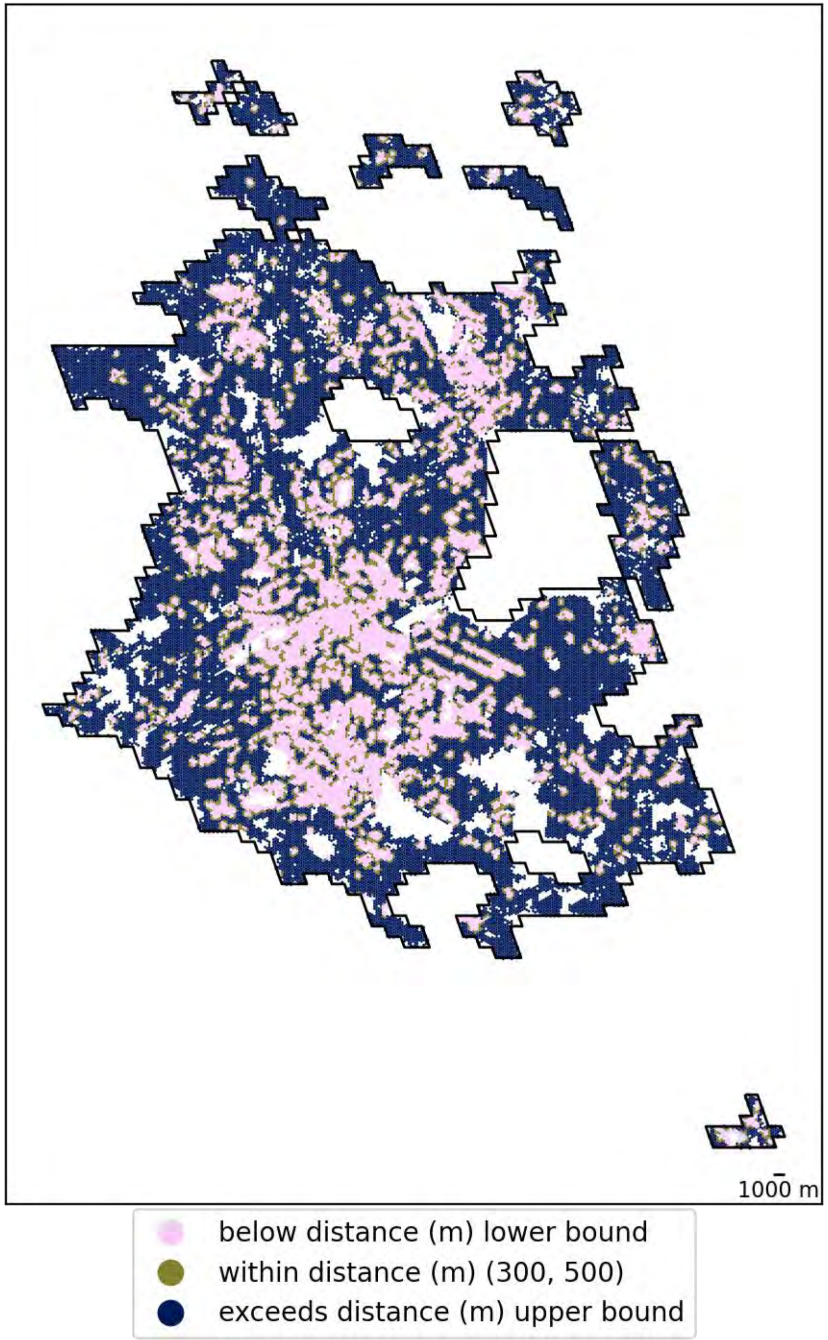

Histogram of Distance to nearest park (m; up to 500m).

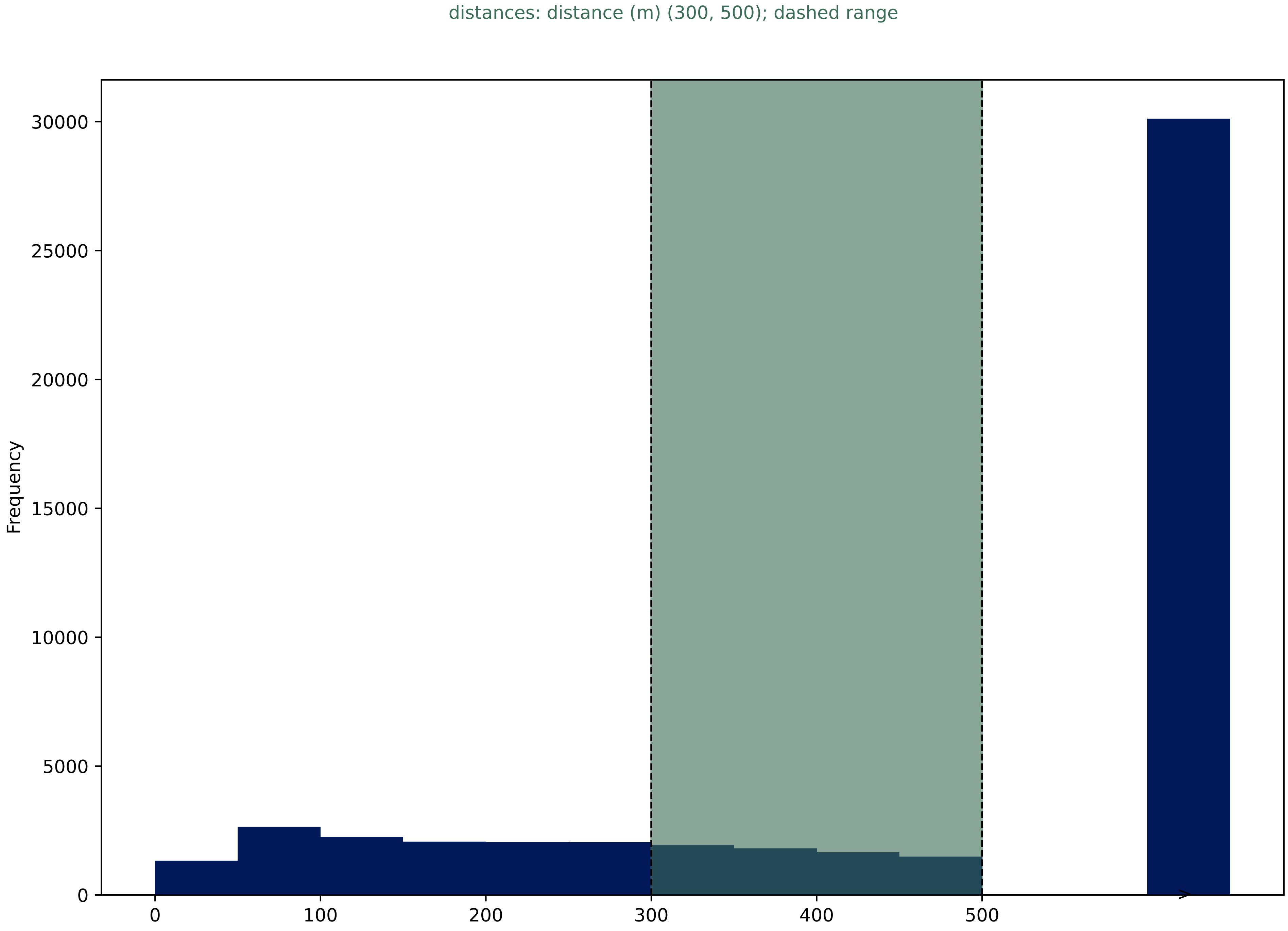

America, North, United States, Baltimore

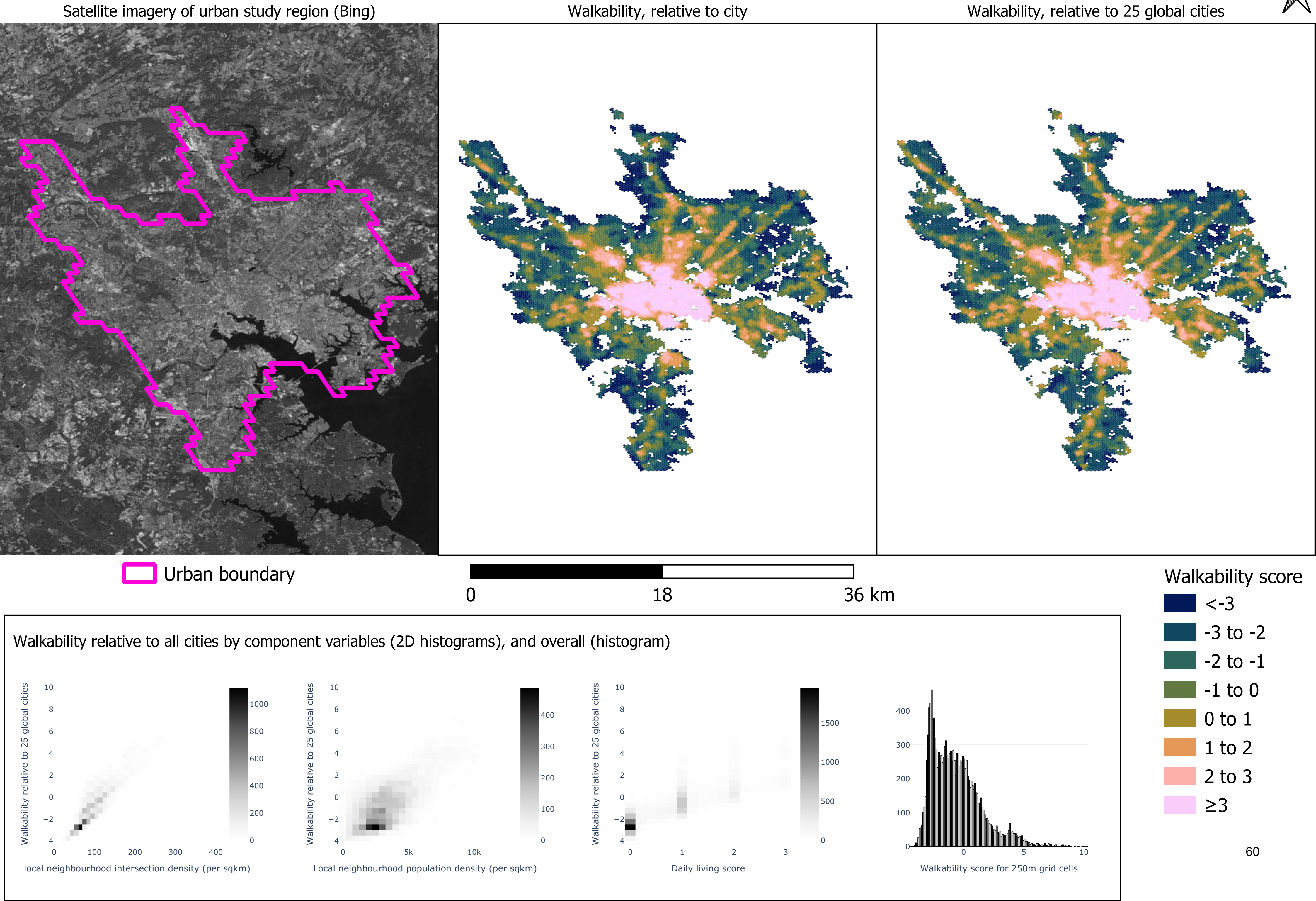

Mean 1000 m neighbourhood population per km²

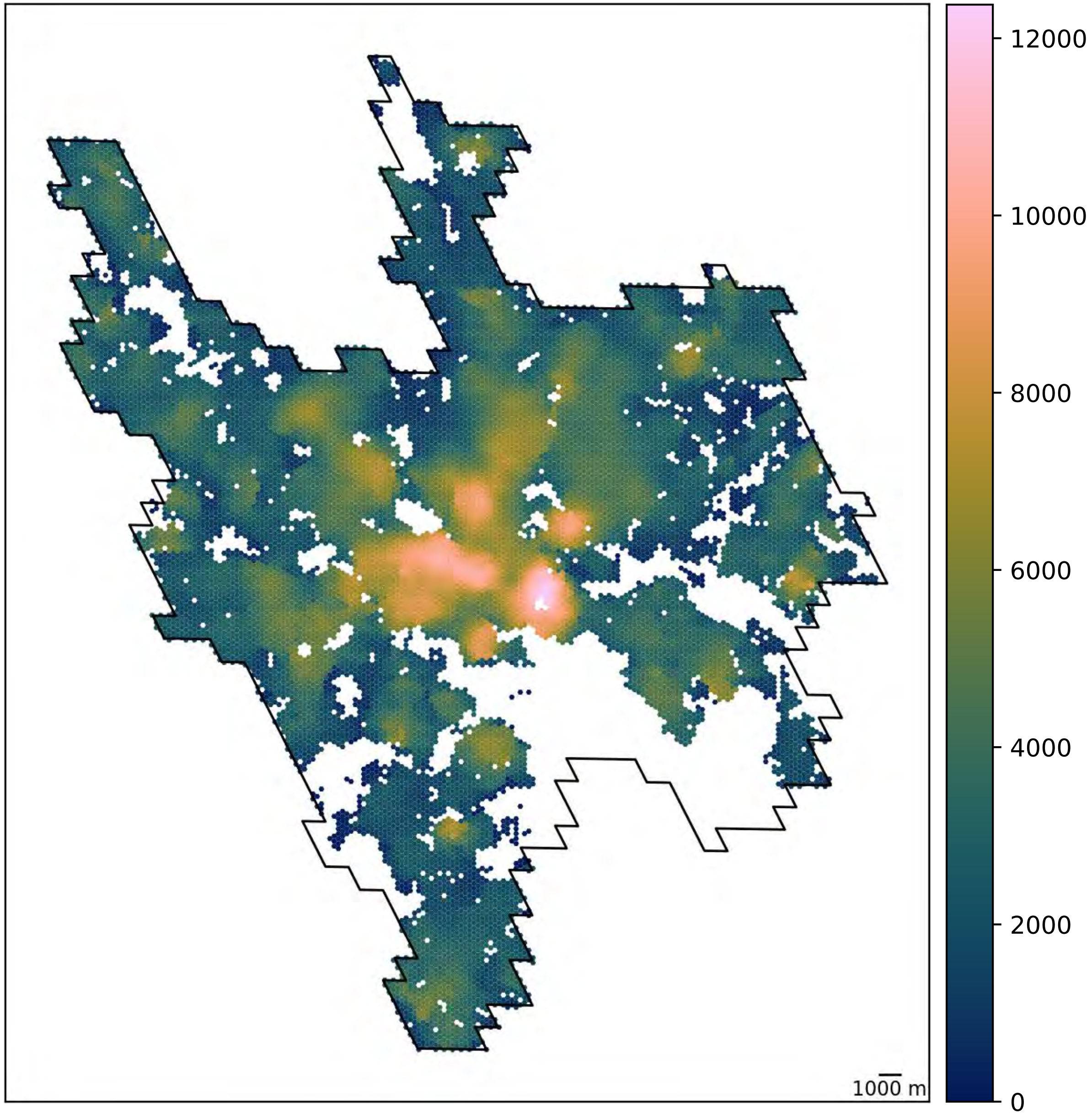

A: Estimated Mean 1000 m neighbourhood population per km² requirement for ≥80% probability of engaging in walking for transport

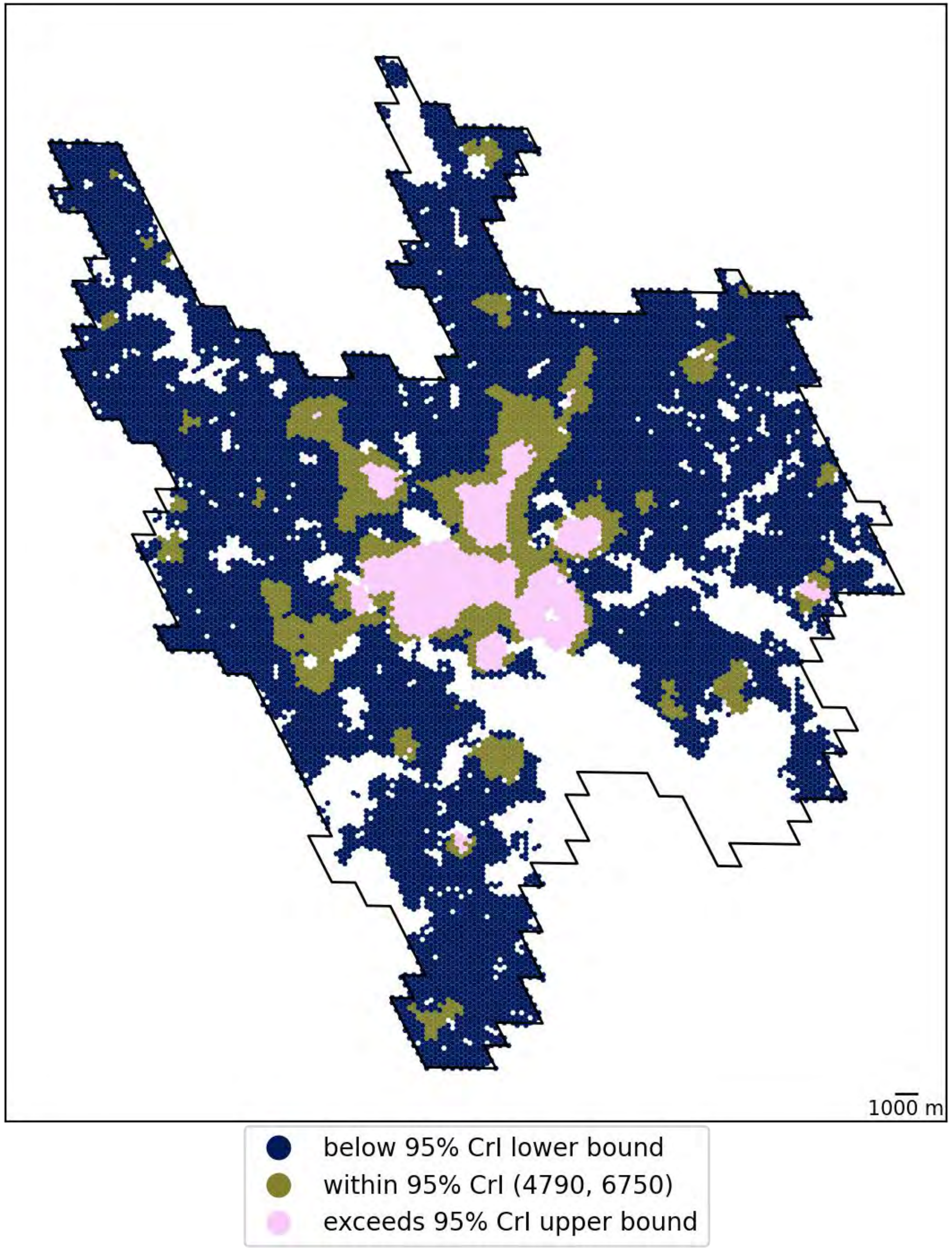

B: Estimated Mean 1000 m neighbourhood population per km² requirement for reaching the WHO's target of a ≥15% relative reduction in insufficient physical activity through walking

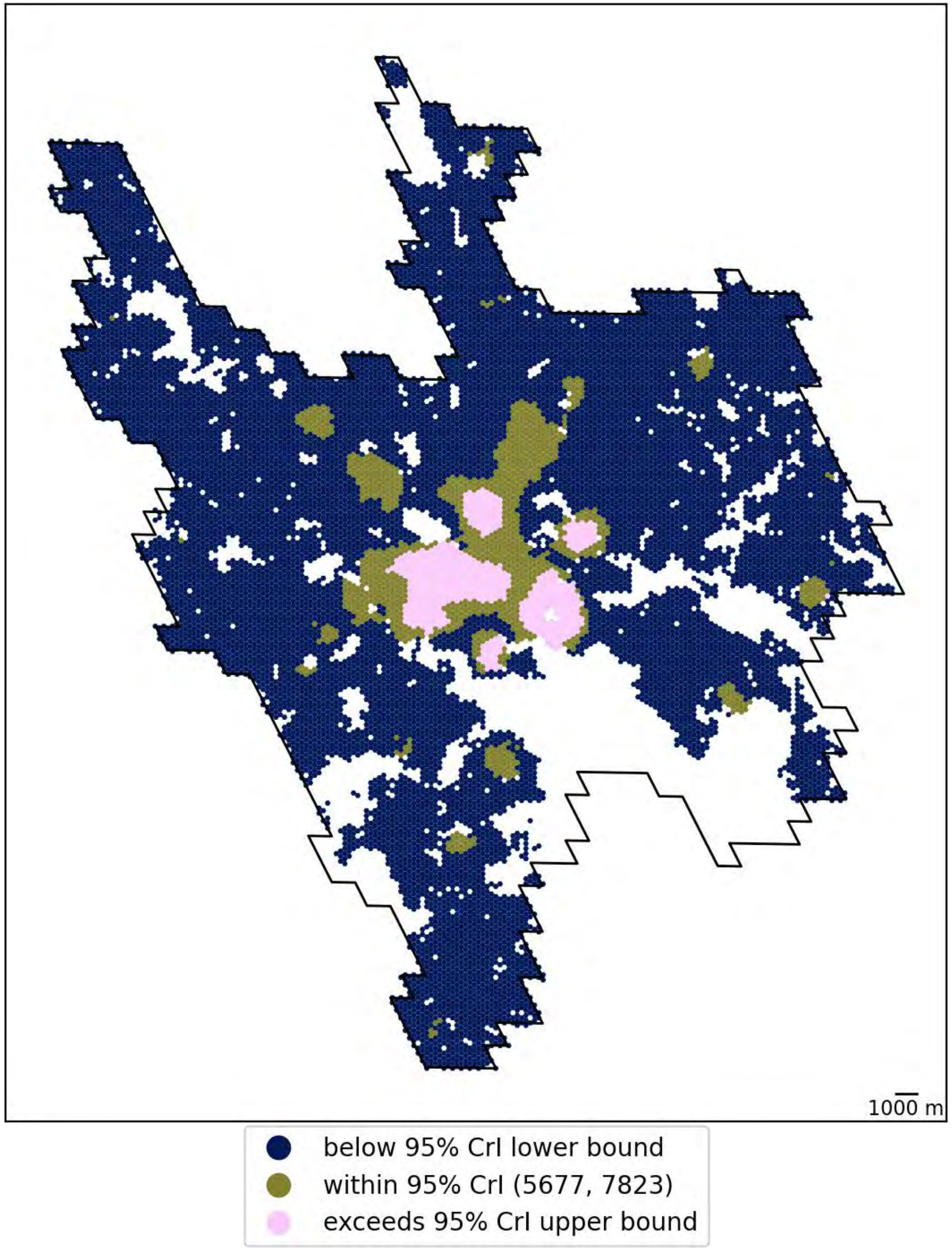

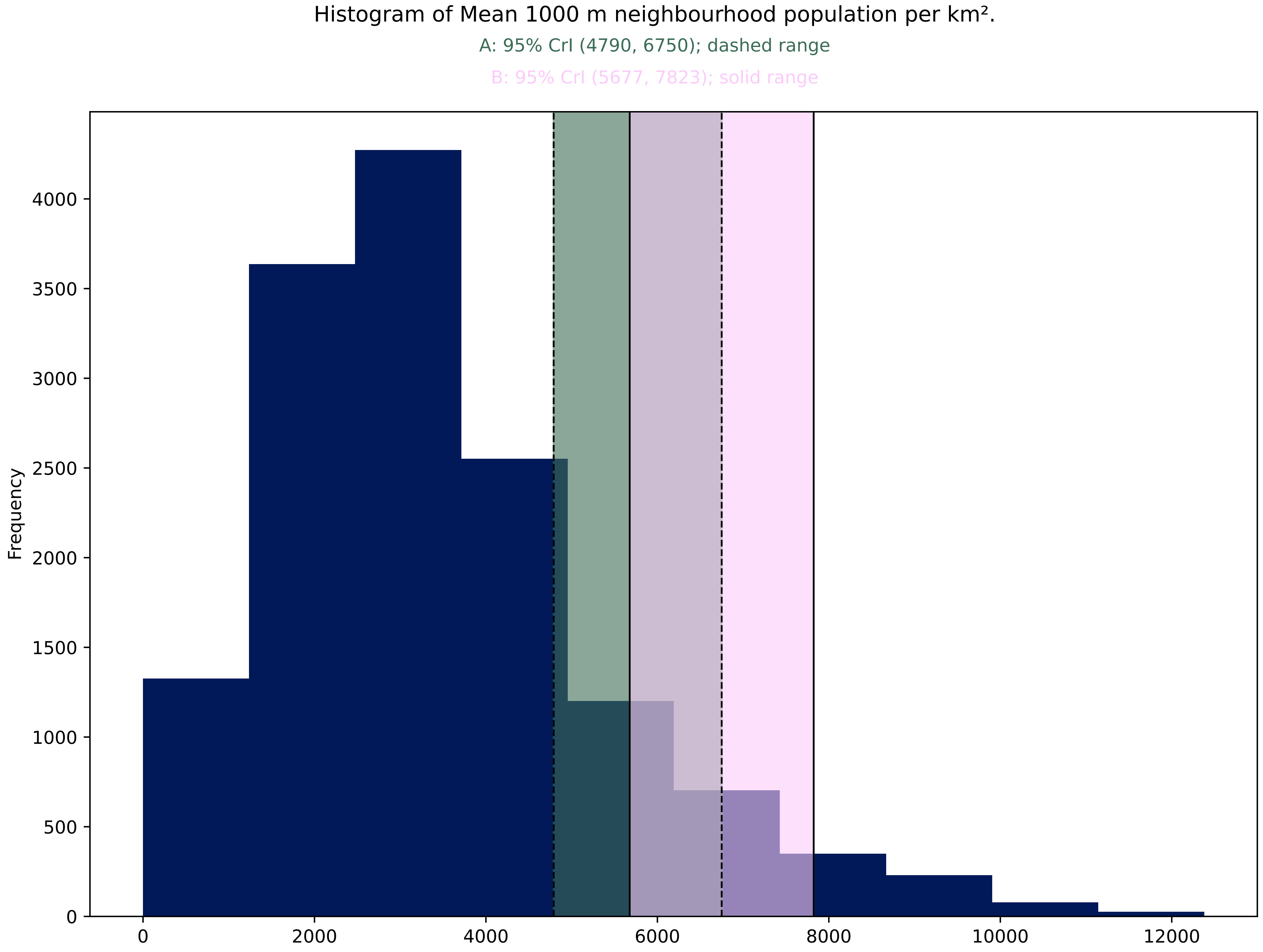
Histogram of Mean 1000 m neighbourhood population per km².
A: 95% CrI (4790, 6750); dashed range
B: 95% CrI (5677, 7823); solid range
Frequency
0
500
1000
1500
2000
2500
3000
3500
4000
0
2000
4000
6000
8000
10000
12000

Mean 1000 m neighbourhood street intersections per km²

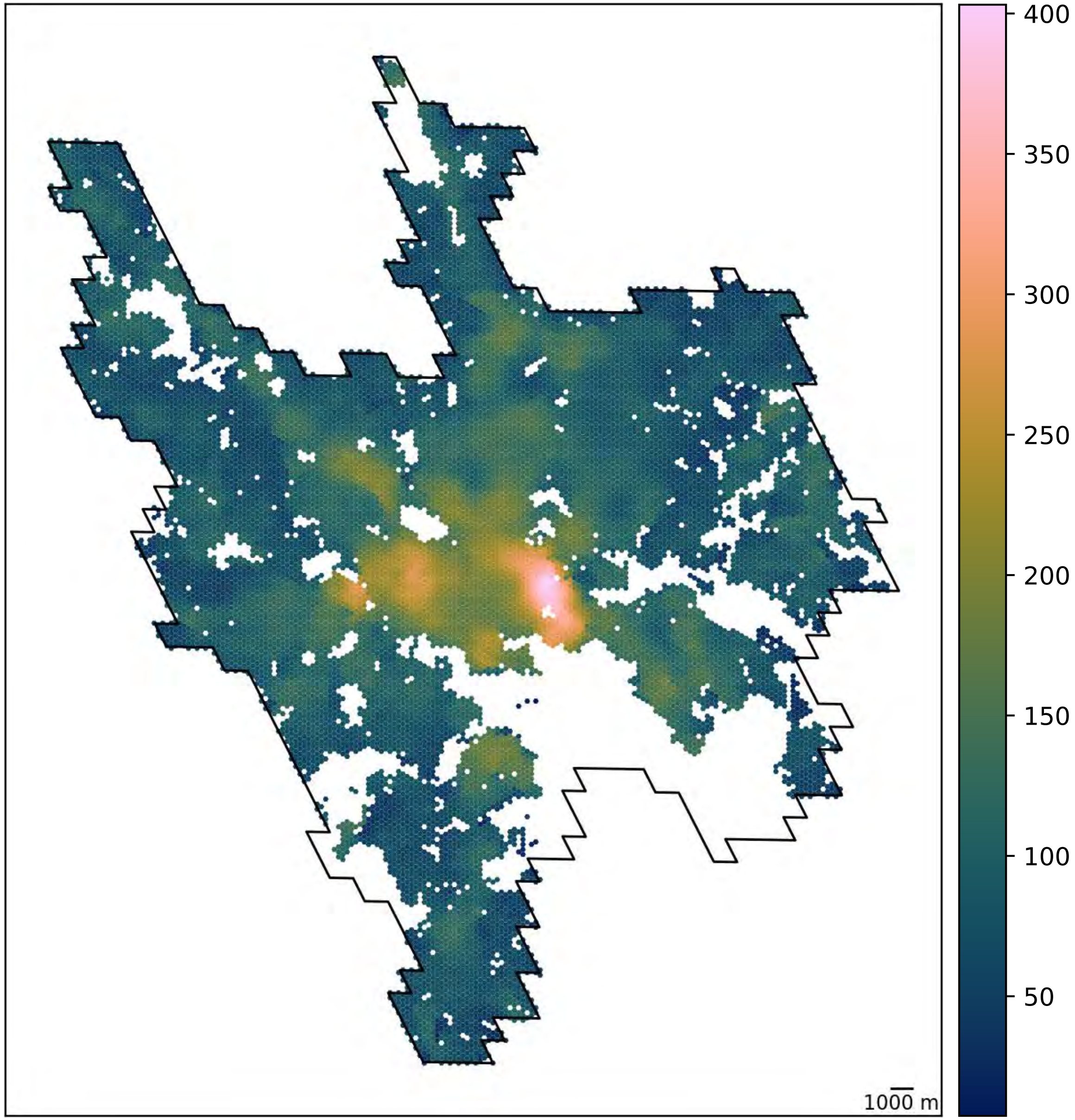

A: Estimated Mean 1000 m neighbourhood street intersections per km² requirement for ≥80% probability of engaging in walking for transport

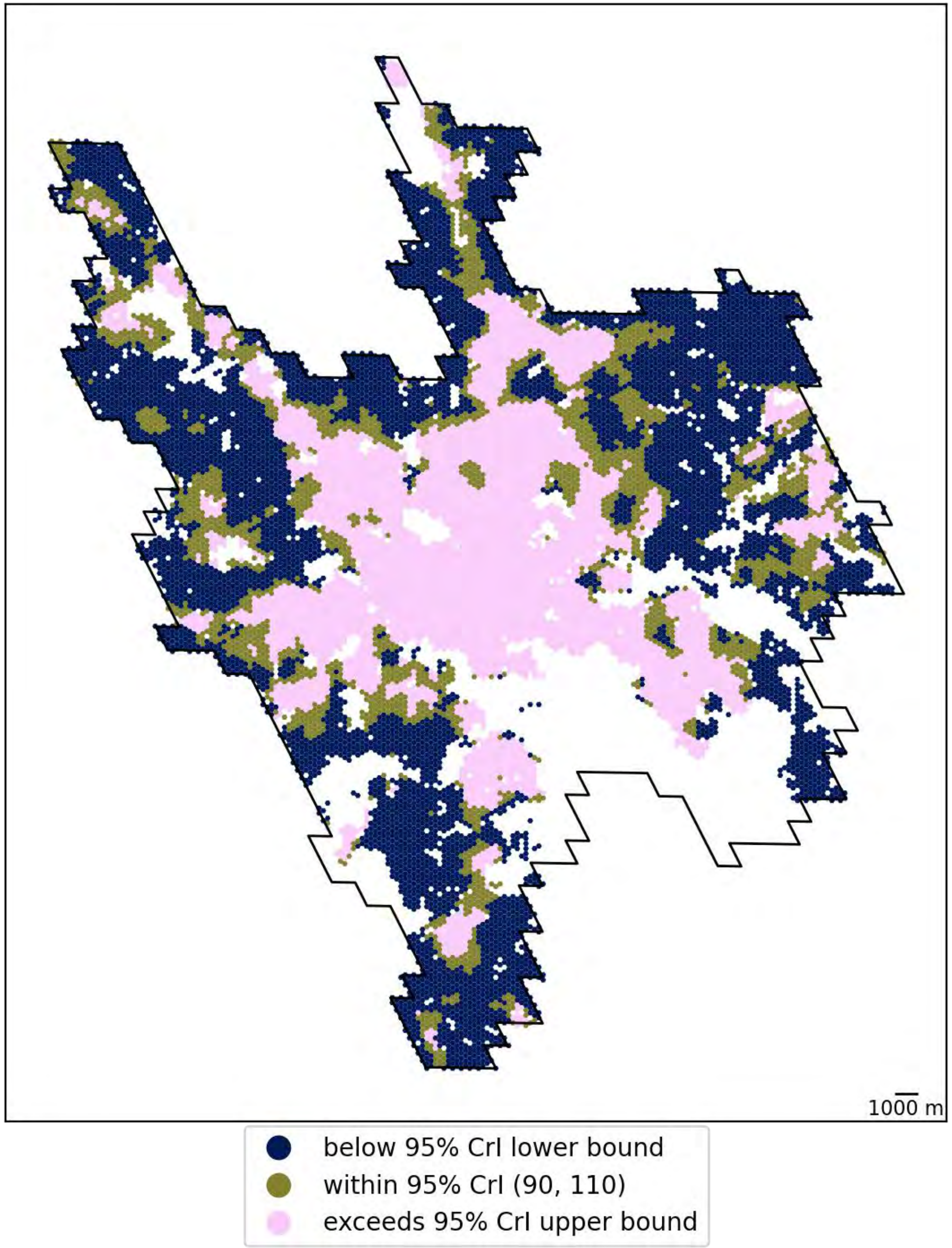

B: Estimated Mean 1000 m neighbourhood street intersections per km² requirement for reaching the WHO's target of a ≥15% relative reduction in insufficient physical activity through walking

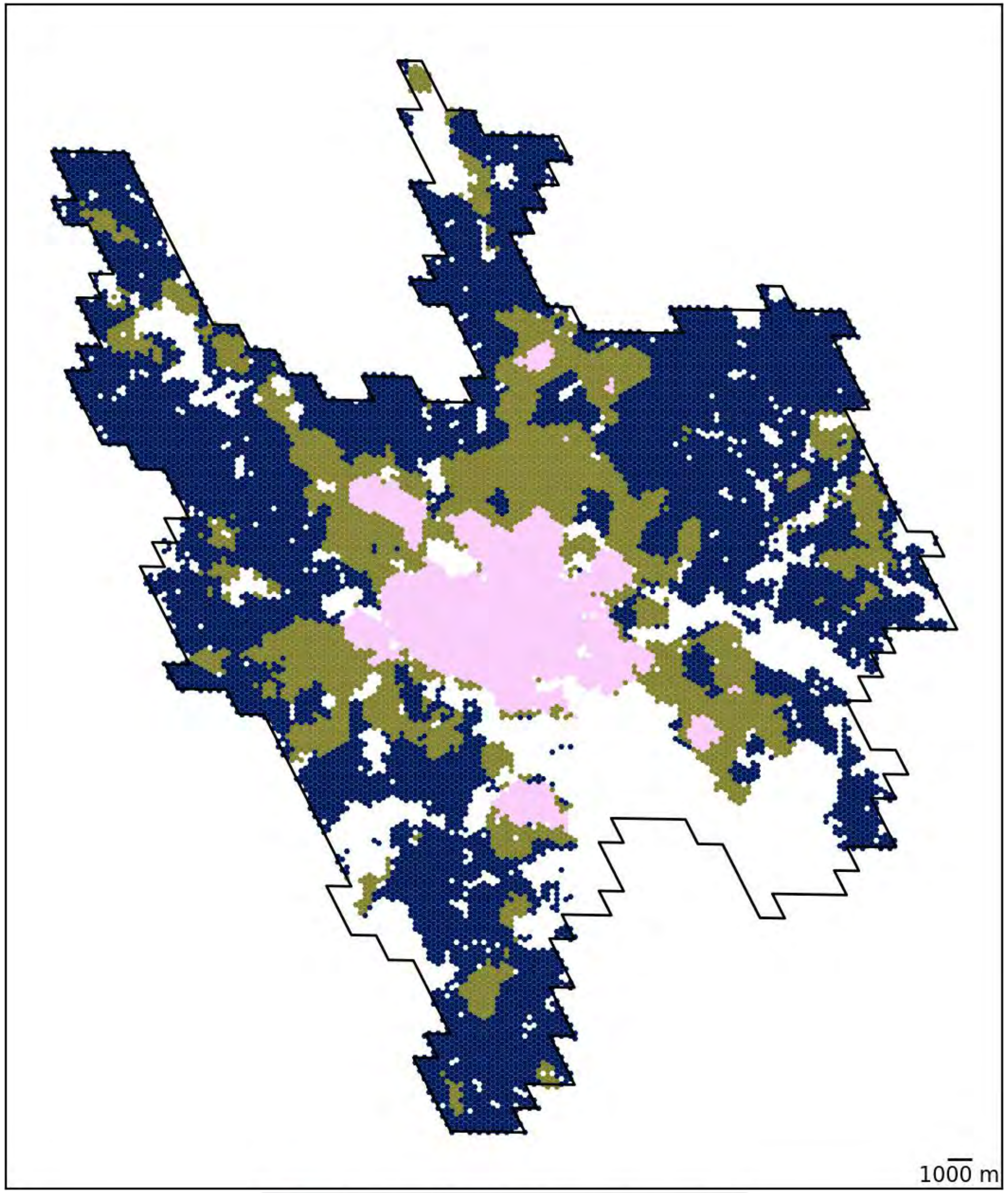

- below 95% CrI lower bound
- within 95% CrI (106, 156)
- exceeds 95% CrI upper bound

Histogram of Mean 1000 m neighbourhood street intersections per km².

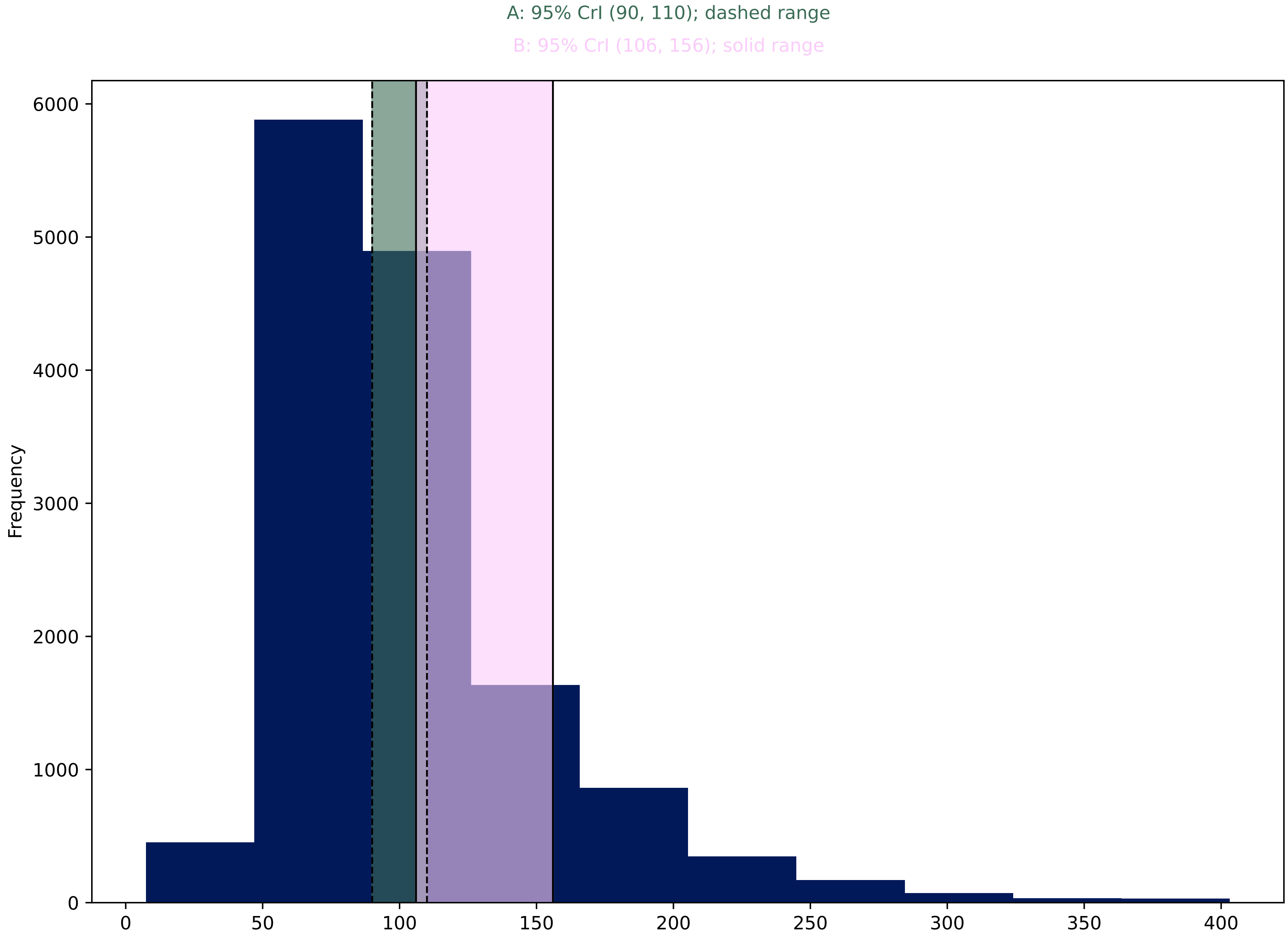

Distance to nearest public transport stops (m; up to 500m)

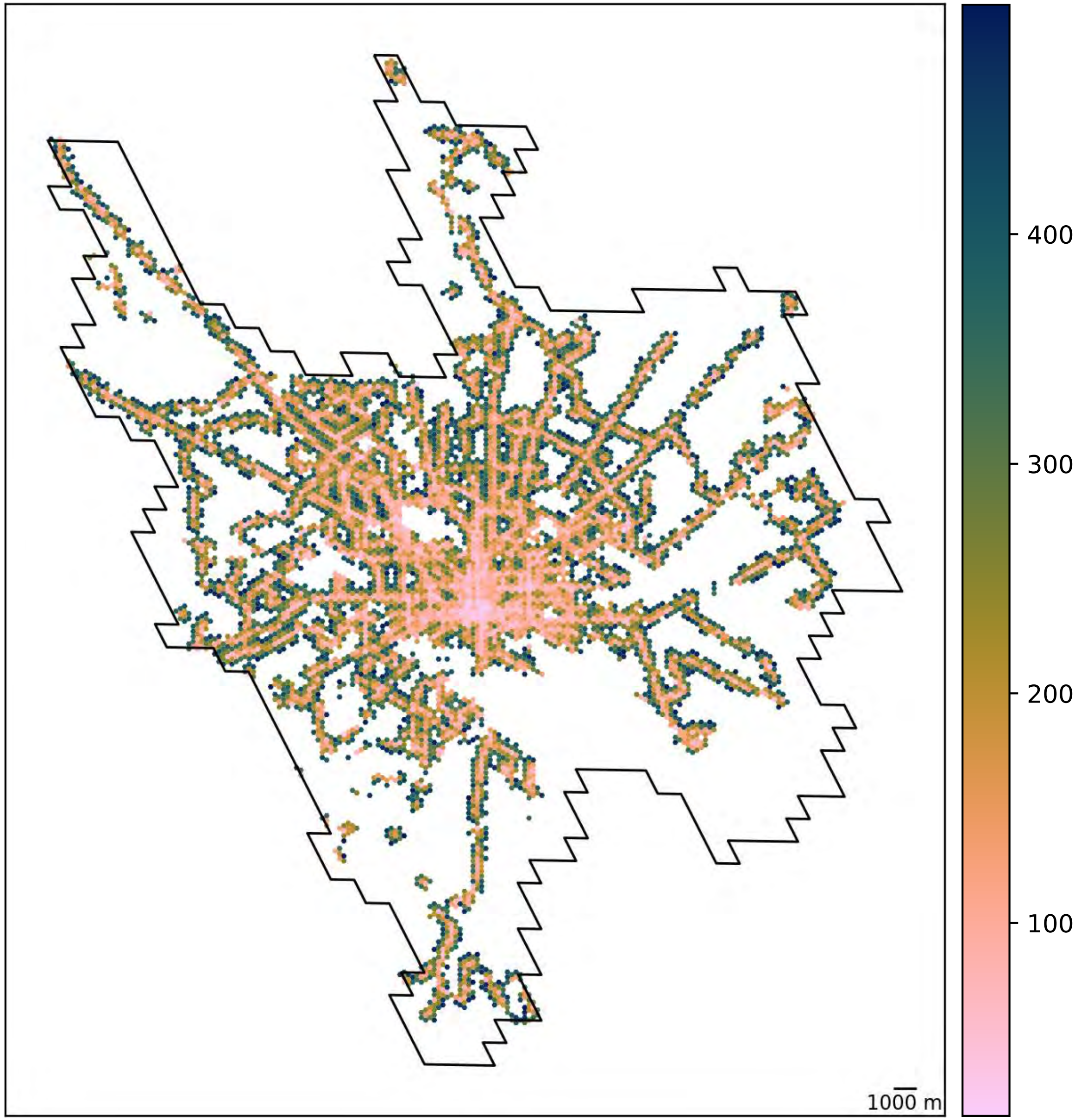

distances: Estimated Distance to nearest public transport stops (m; up to 500m) requirement for distances to destinations, measured up to a maximum distance target threshold of 500 metres

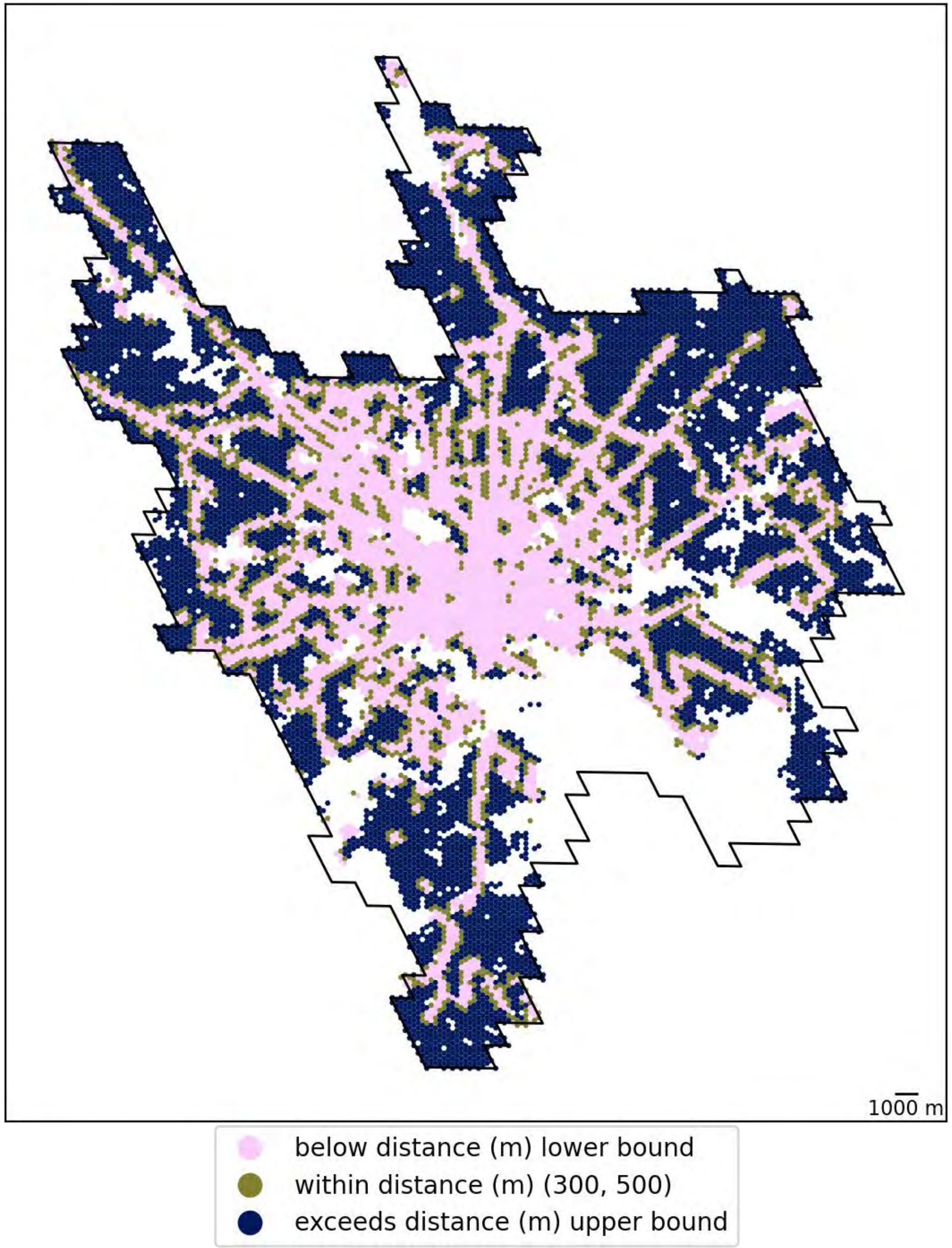

Histogram of Distance to nearest public transport stops (m; up to 500m).

distances: distance (m) (300, 500); dashed range

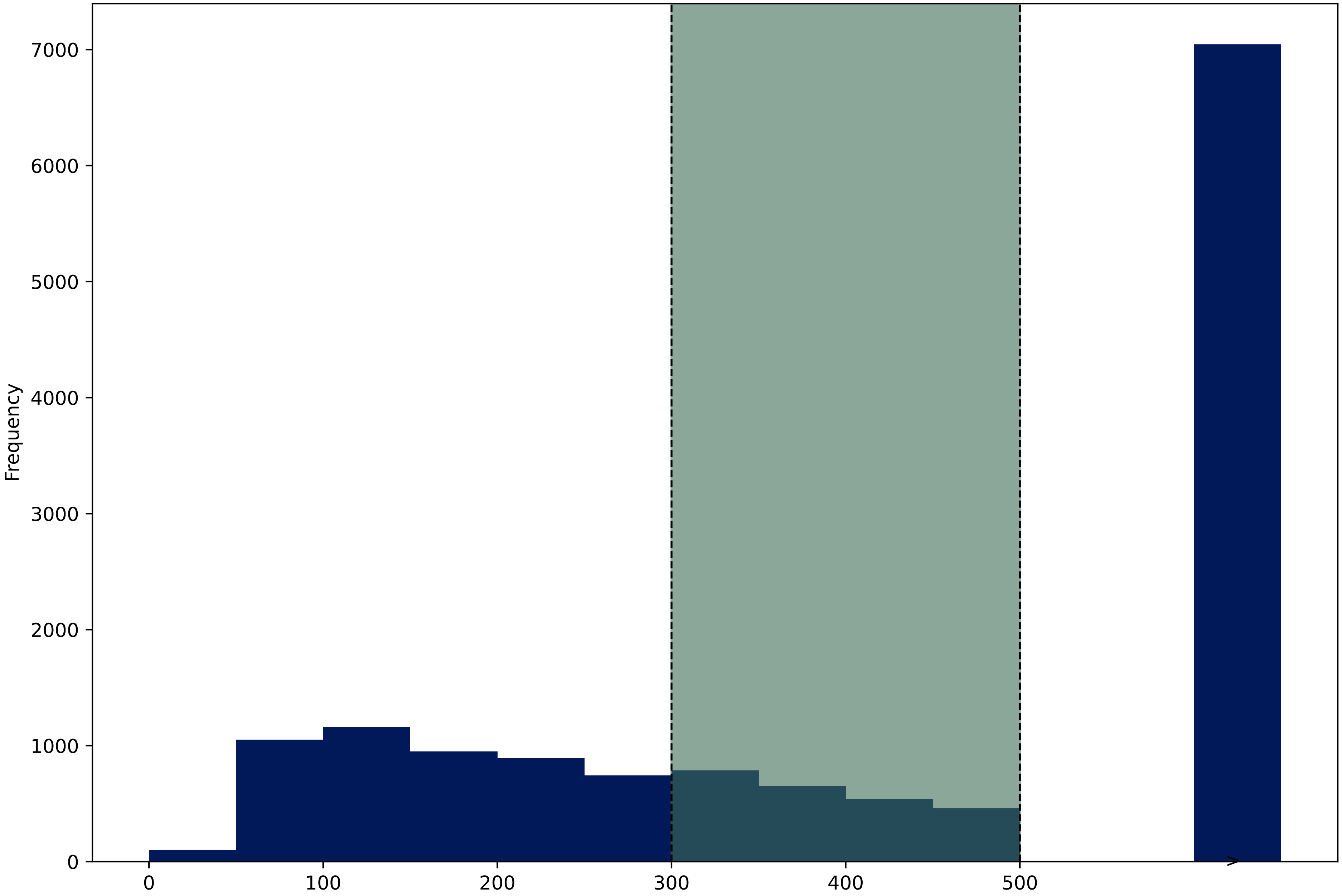

America, North, United States, Phoenix

Satellite imagery of urban study region (Bing) | Walkability, relative to city | Walkability, relative to 25 global cities

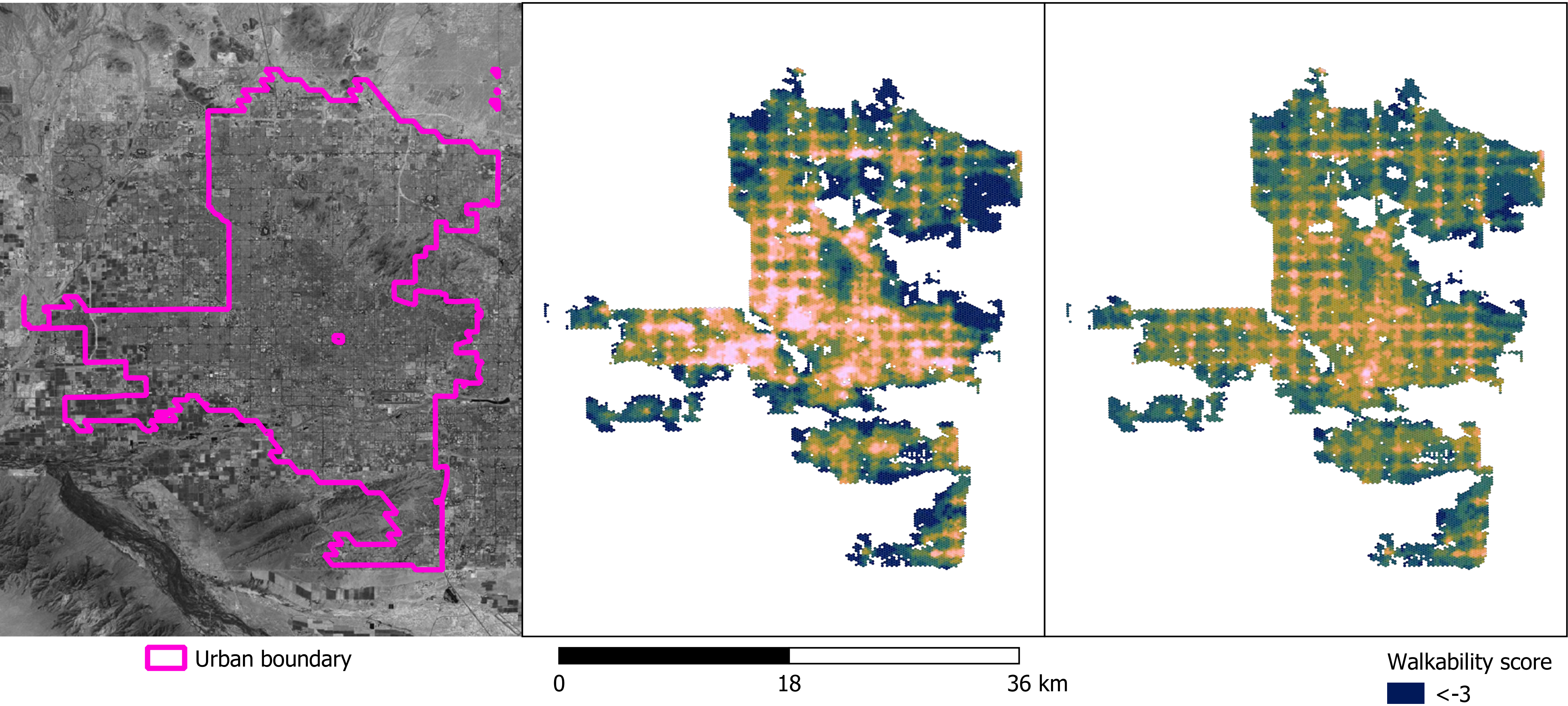

Walkability relative to all cities by component variables (2D histograms), and overall (histogram)

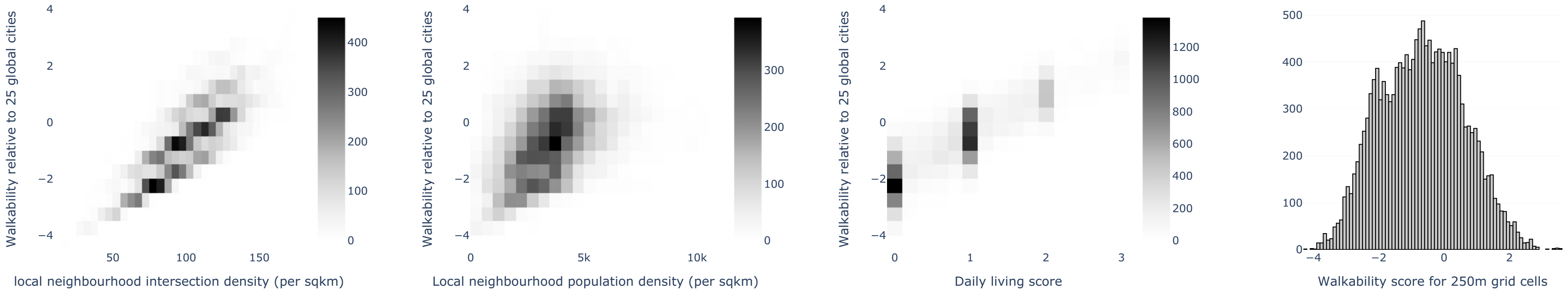

Distance to nearest park (m; up to 500m)

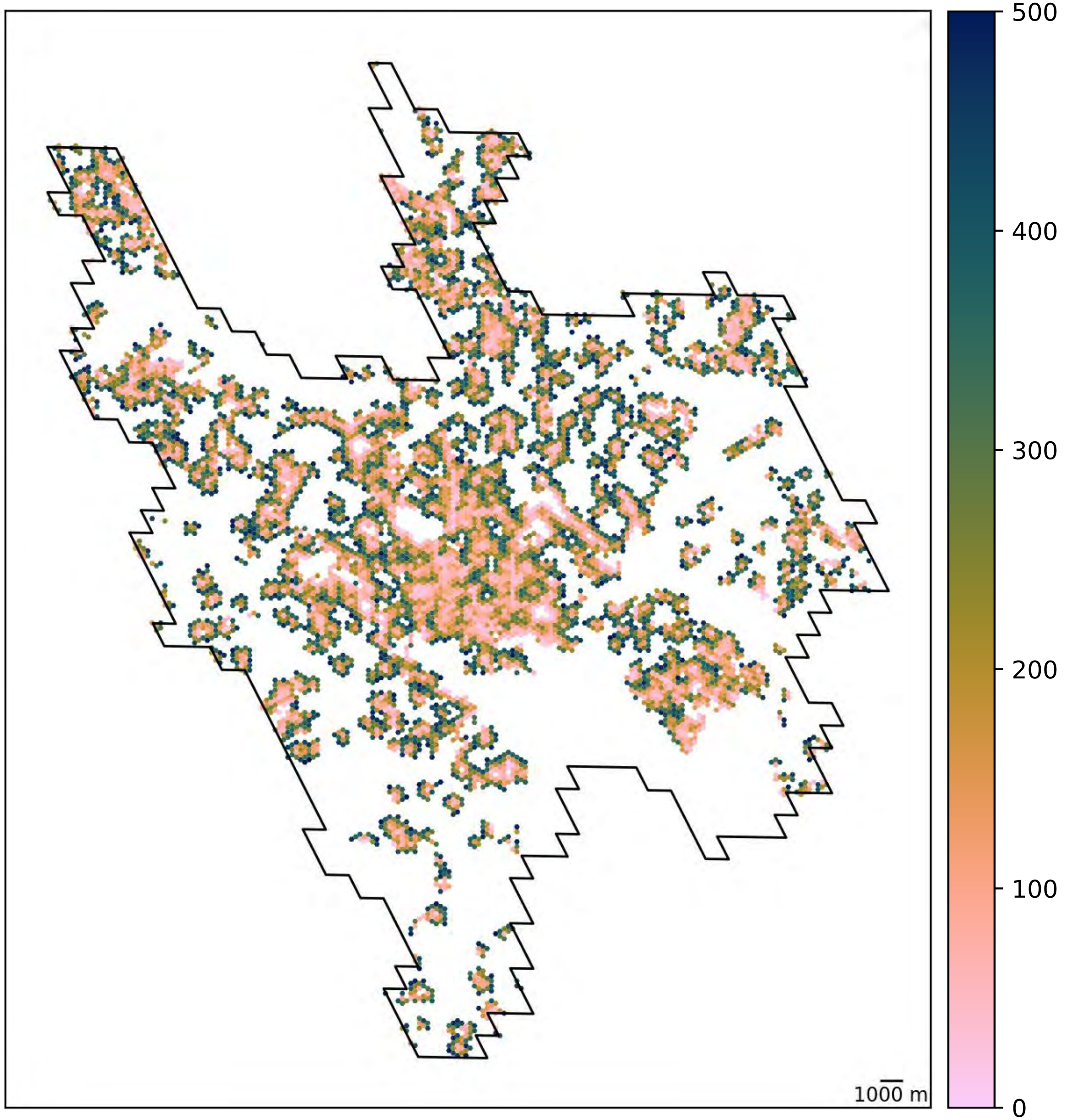

distances: Estimated Distance to nearest park (m; up to 500m) requirement for distances to destinations, measured up to a maximum distance target threshold of 500 metres

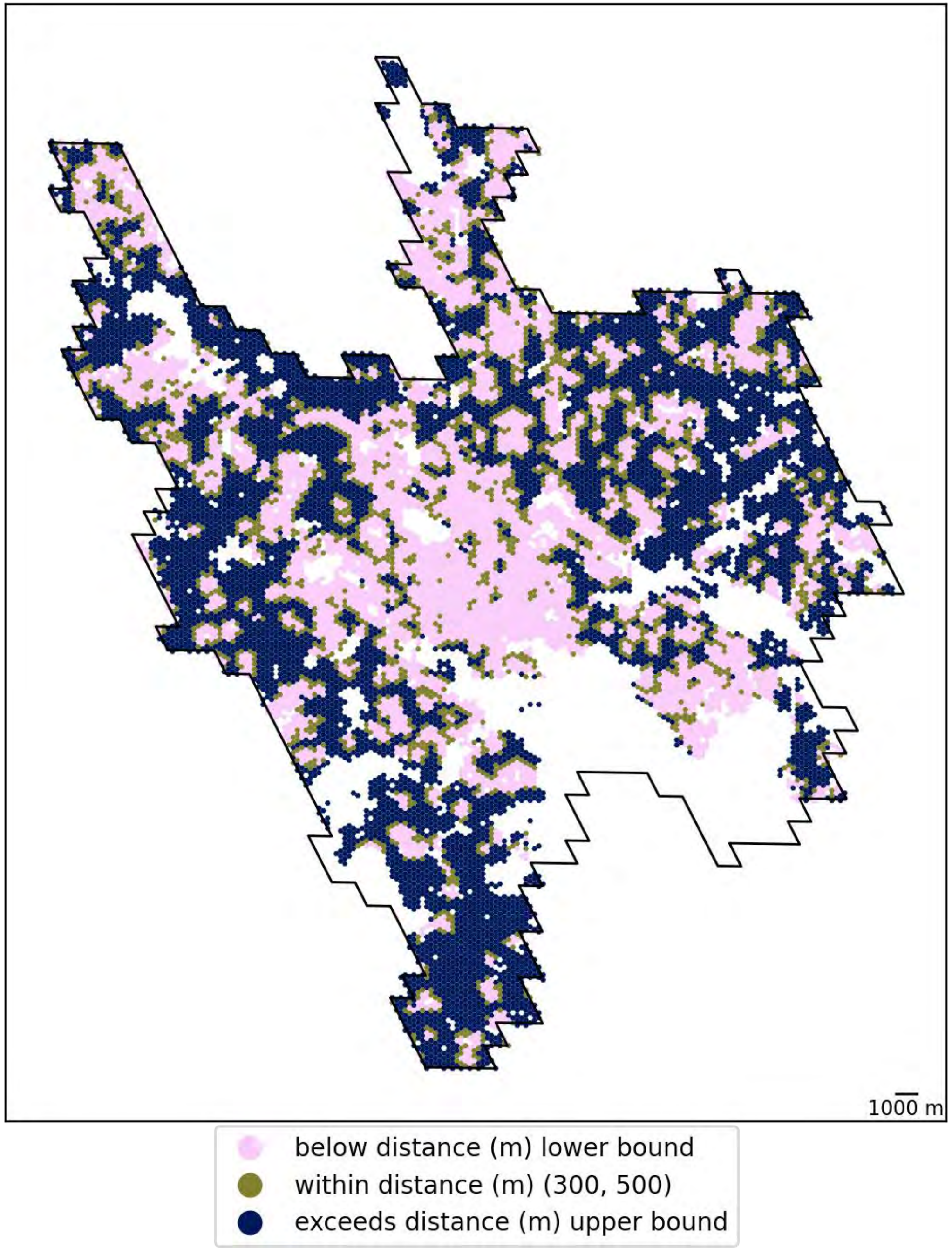

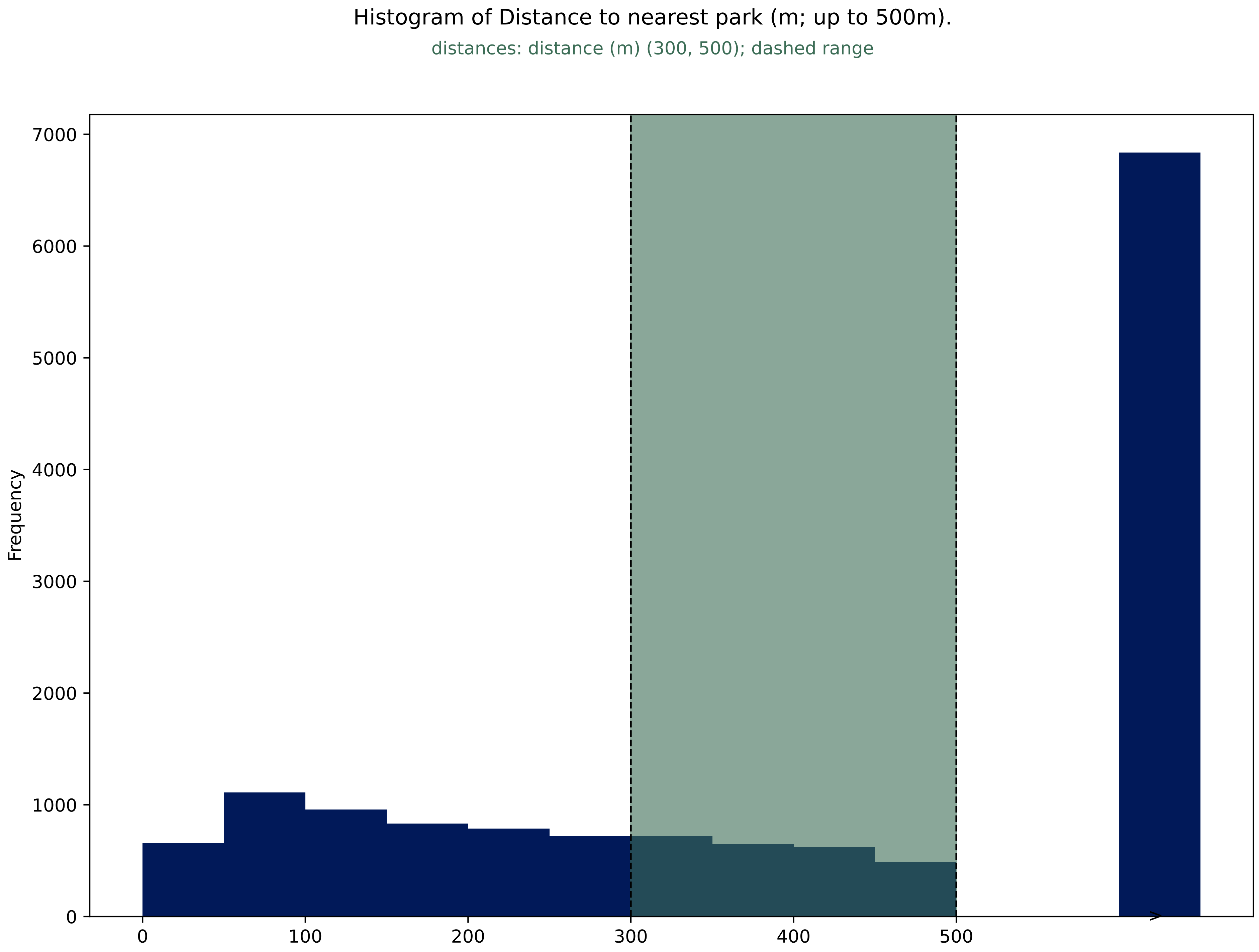

Histogram of Distance to nearest park (m; up to 500m).
distances: distance (m) (300, 500); dashed range
Frequency
0
1000
2000
3000
4000
5000
6000
7000
0
100
200
300
400
500
>

America, North, United States, Phoenix

Satellite imagery of urban study region (Bing)

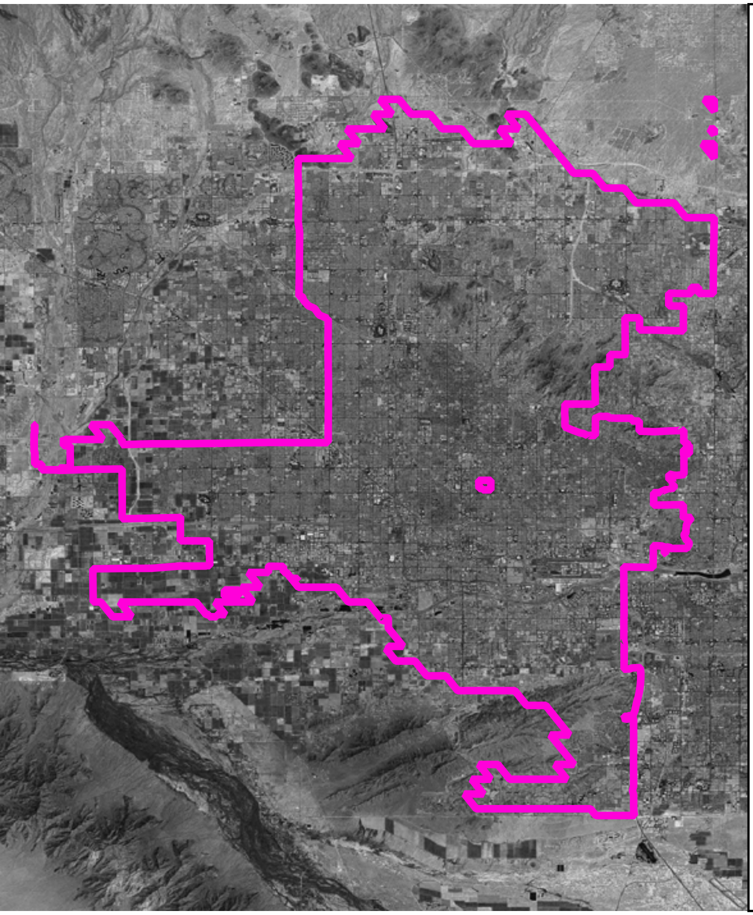

Urban boundary

Walkability, relative to city

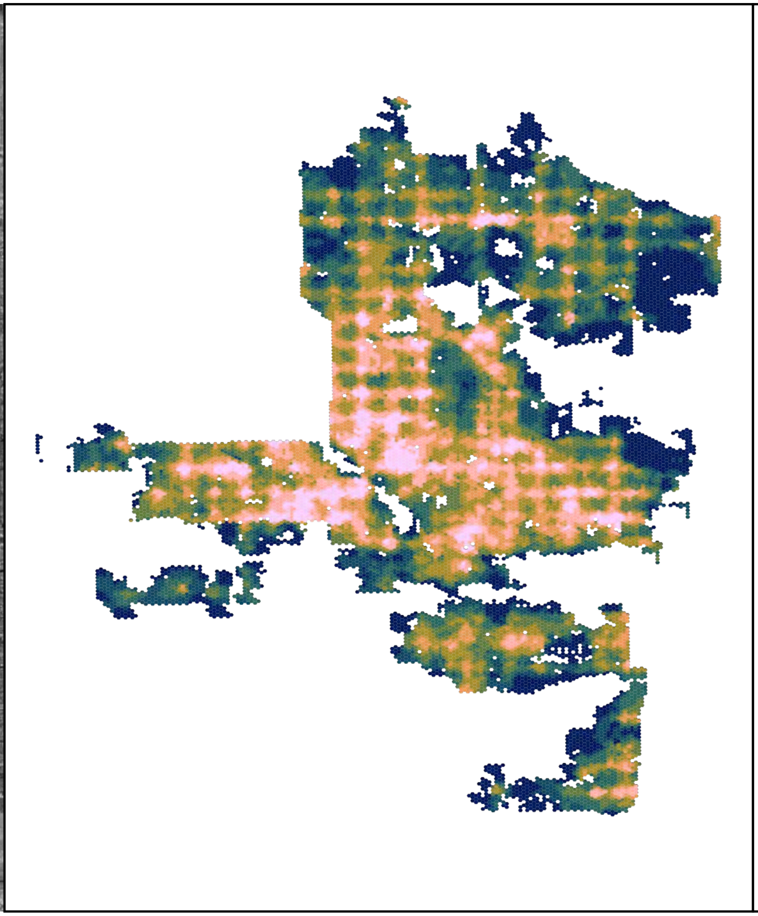

Walkability, relative to 25 global cities

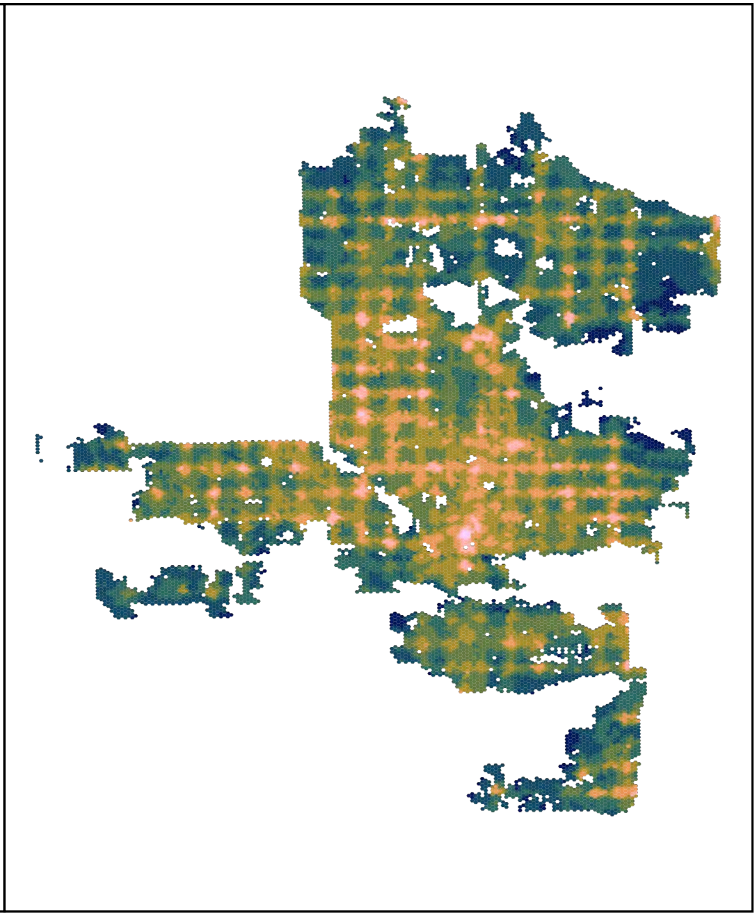

0 18 36 km

Walkability score

- <-3
- -3 to -2
- -2 to -1
- -1 to 0
- 0 to 1
- 1 to 2
- 2 to 3
- ≥3

Walkability relative to all cities by component variables (2D histograms), and overall (histogram)

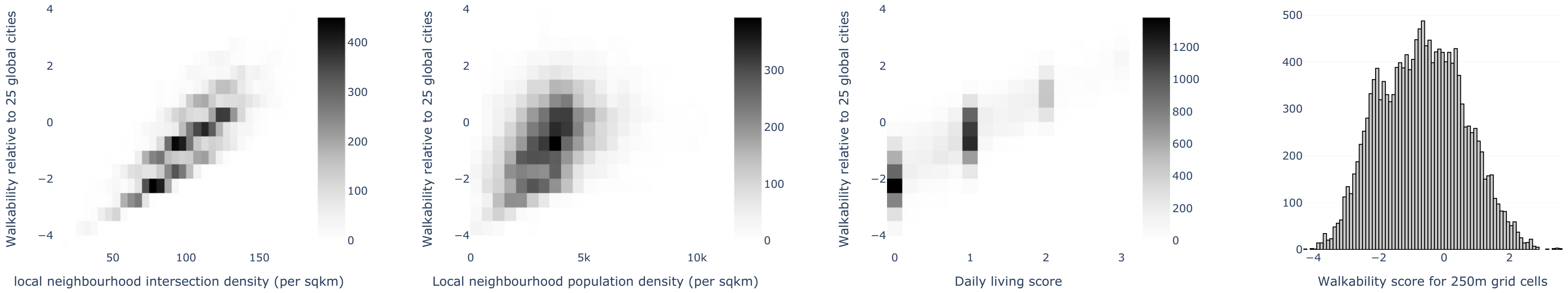

Mean 1000 m neighbourhood population per km²

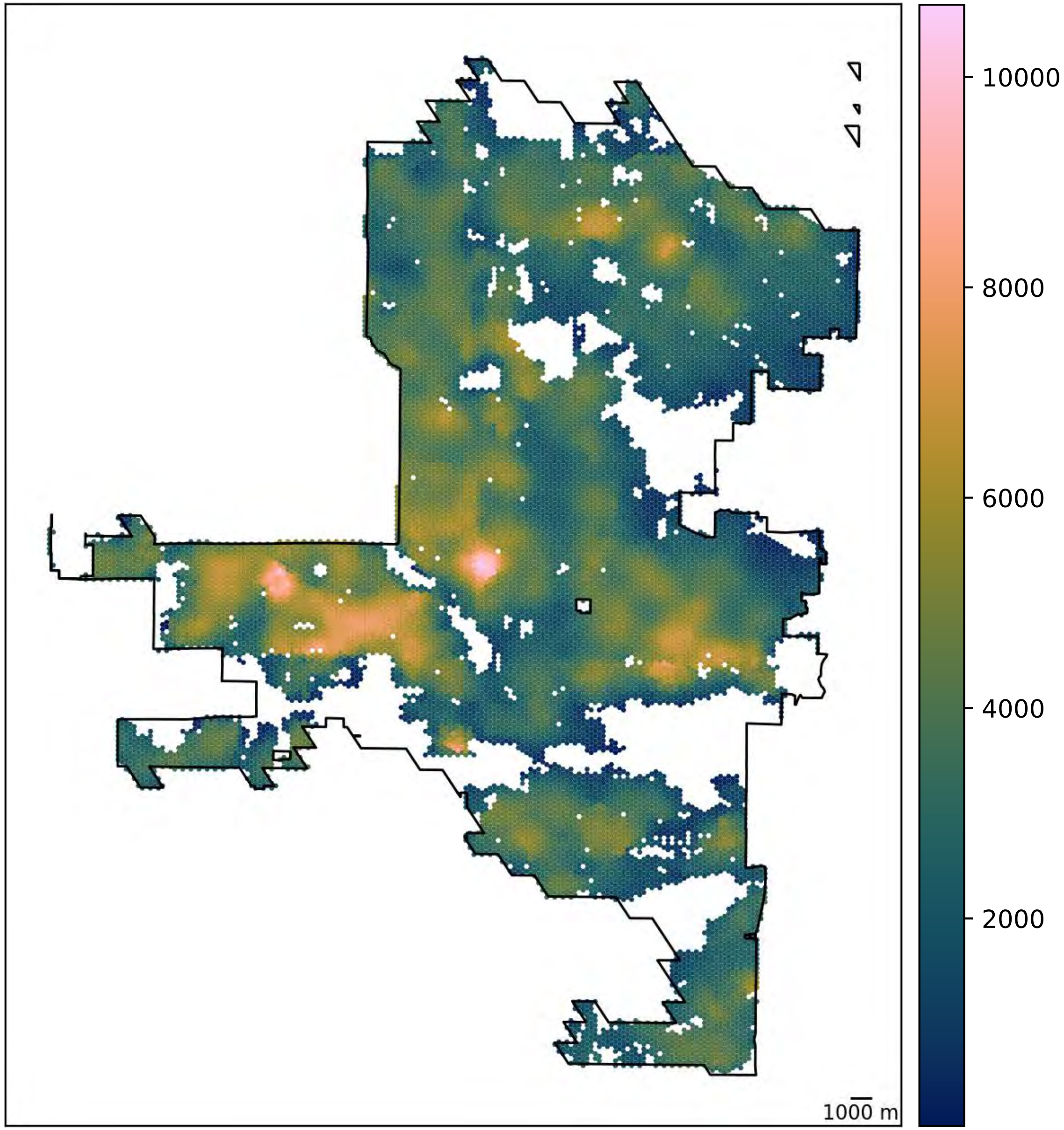

A: Estimated Mean 1000 m neighbourhood population per km² requirement for ≥80% probability of engaging in walking for transport

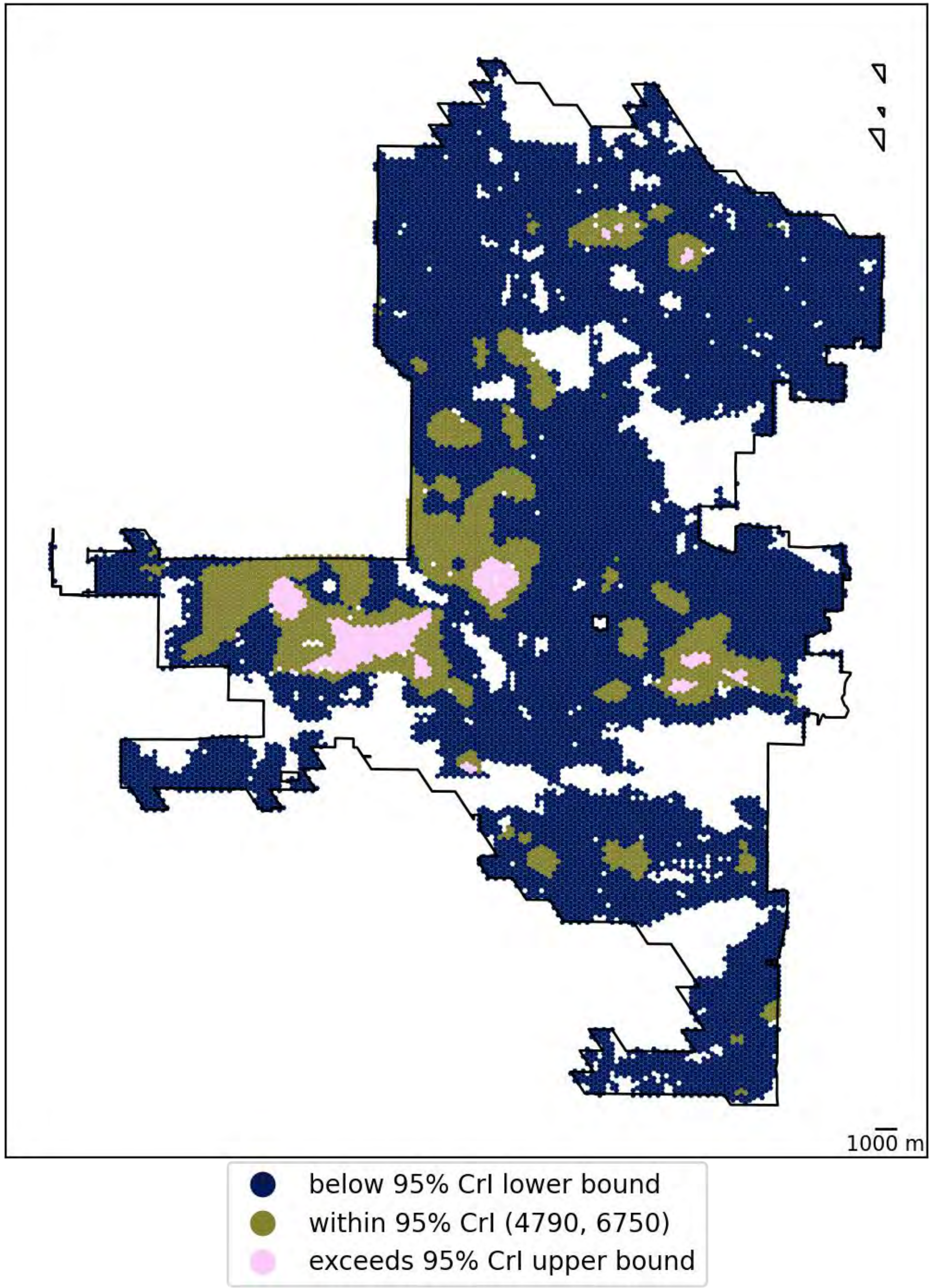

B: Estimated Mean 1000 m neighbourhood population per km² requirement for reaching the WHO's target of a ≥15% relative reduction in insufficient physical activity through walking

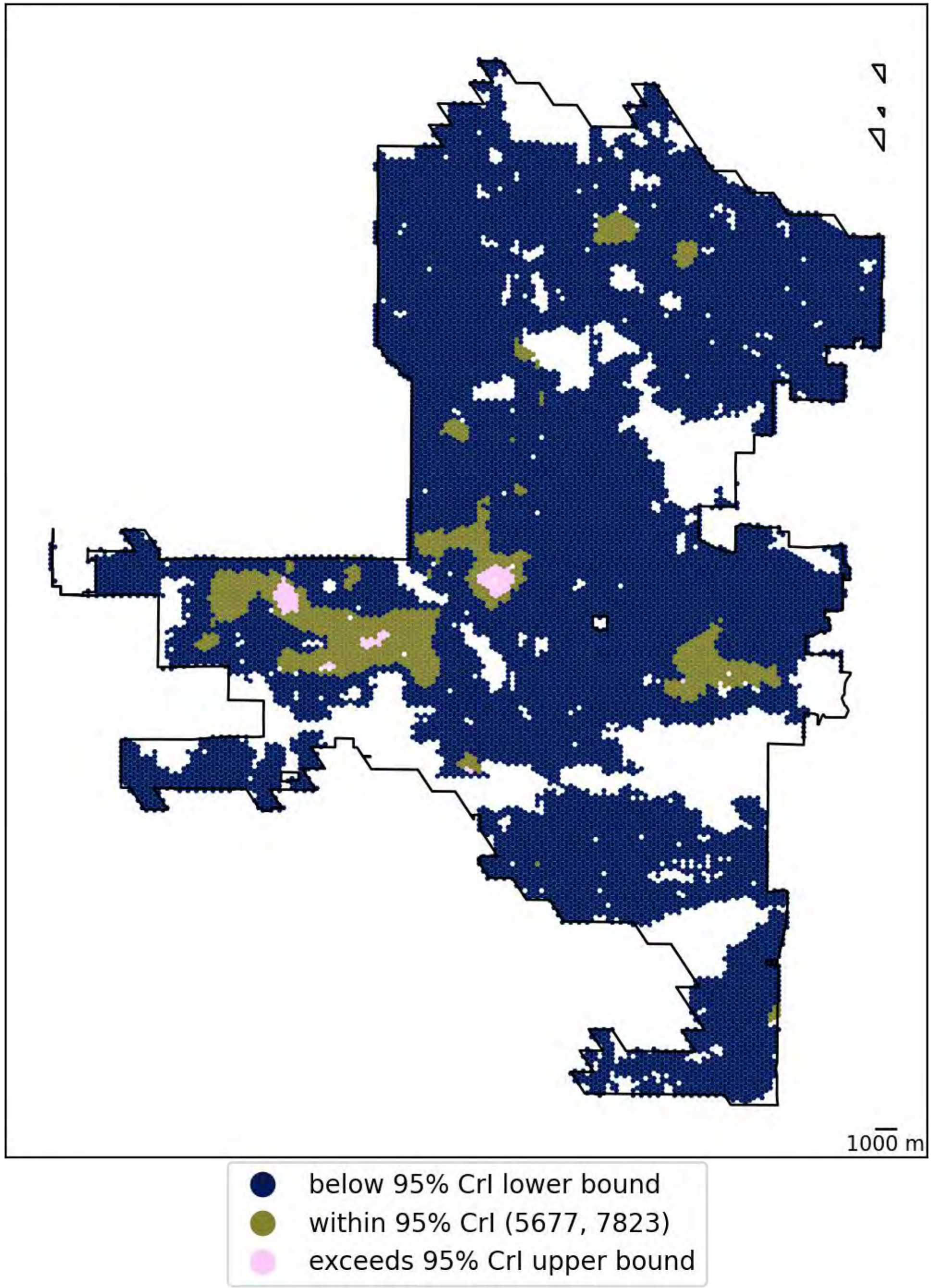

Histogram of Mean 1000 m neighbourhood population per km².

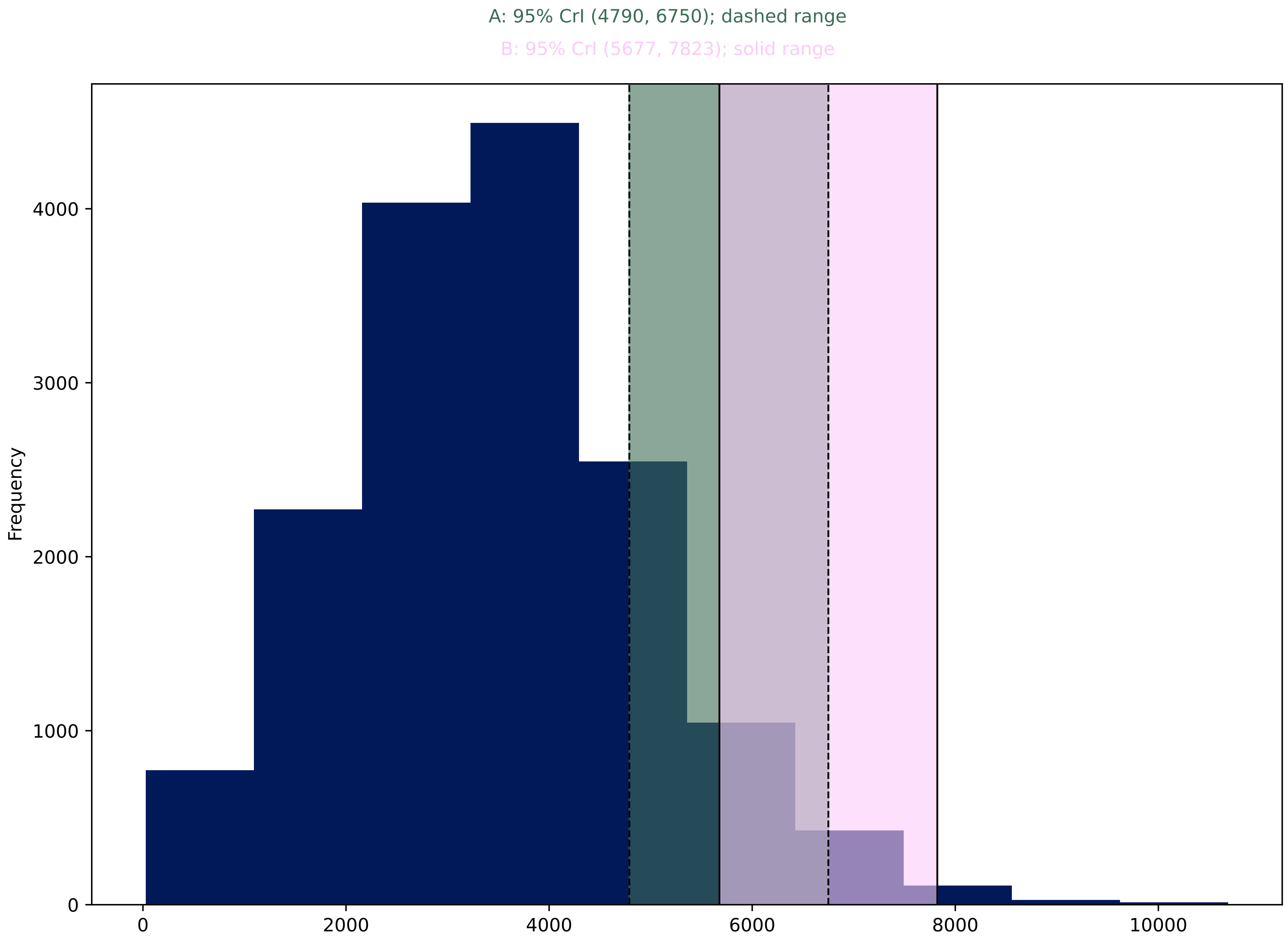

Mean 1000 m neighbourhood street intersections per km²

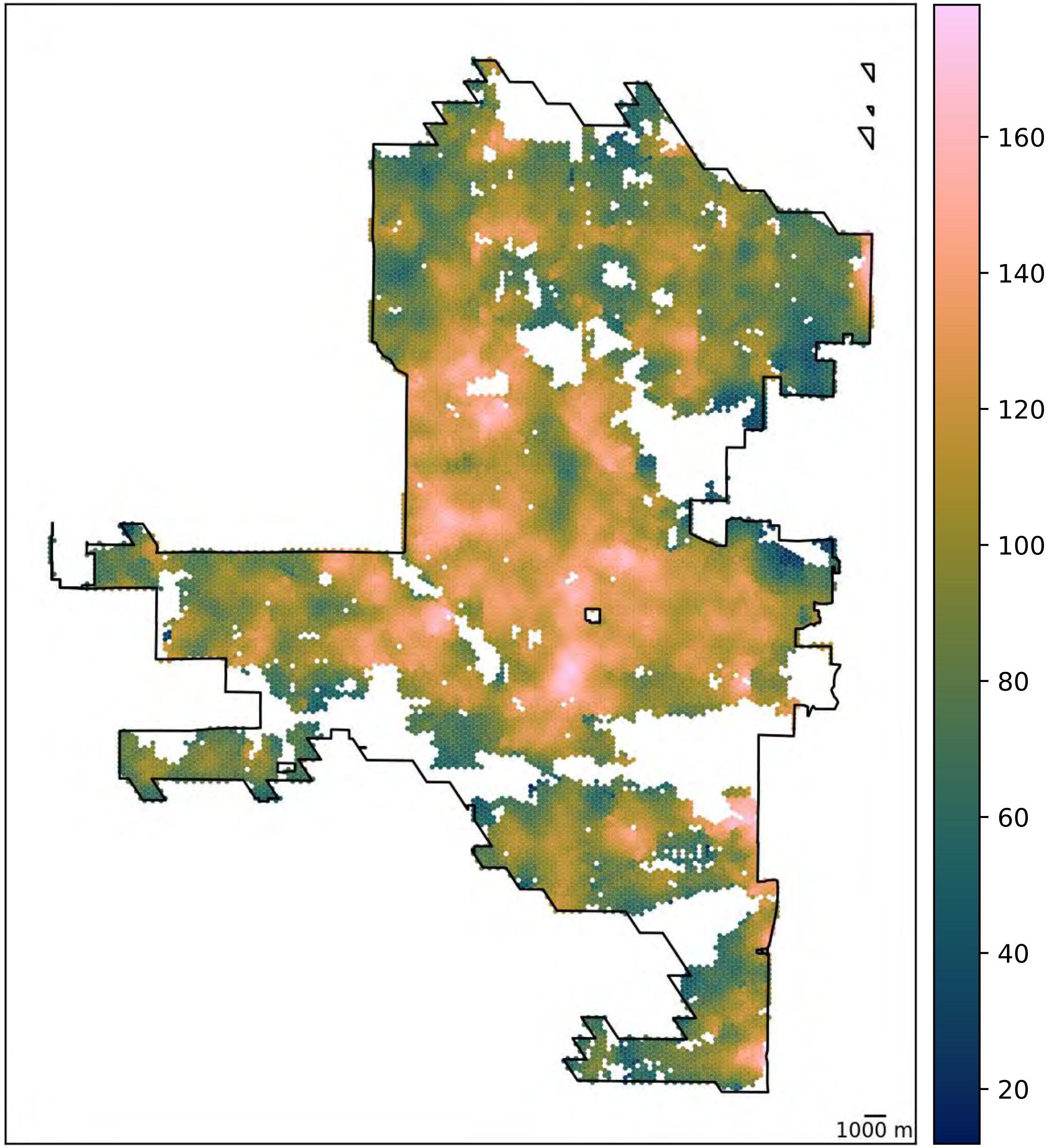

A: Estimated Mean 1000 m neighbourhood street intersections per km² requirement for ≥80% probability of engaging in walking for transport

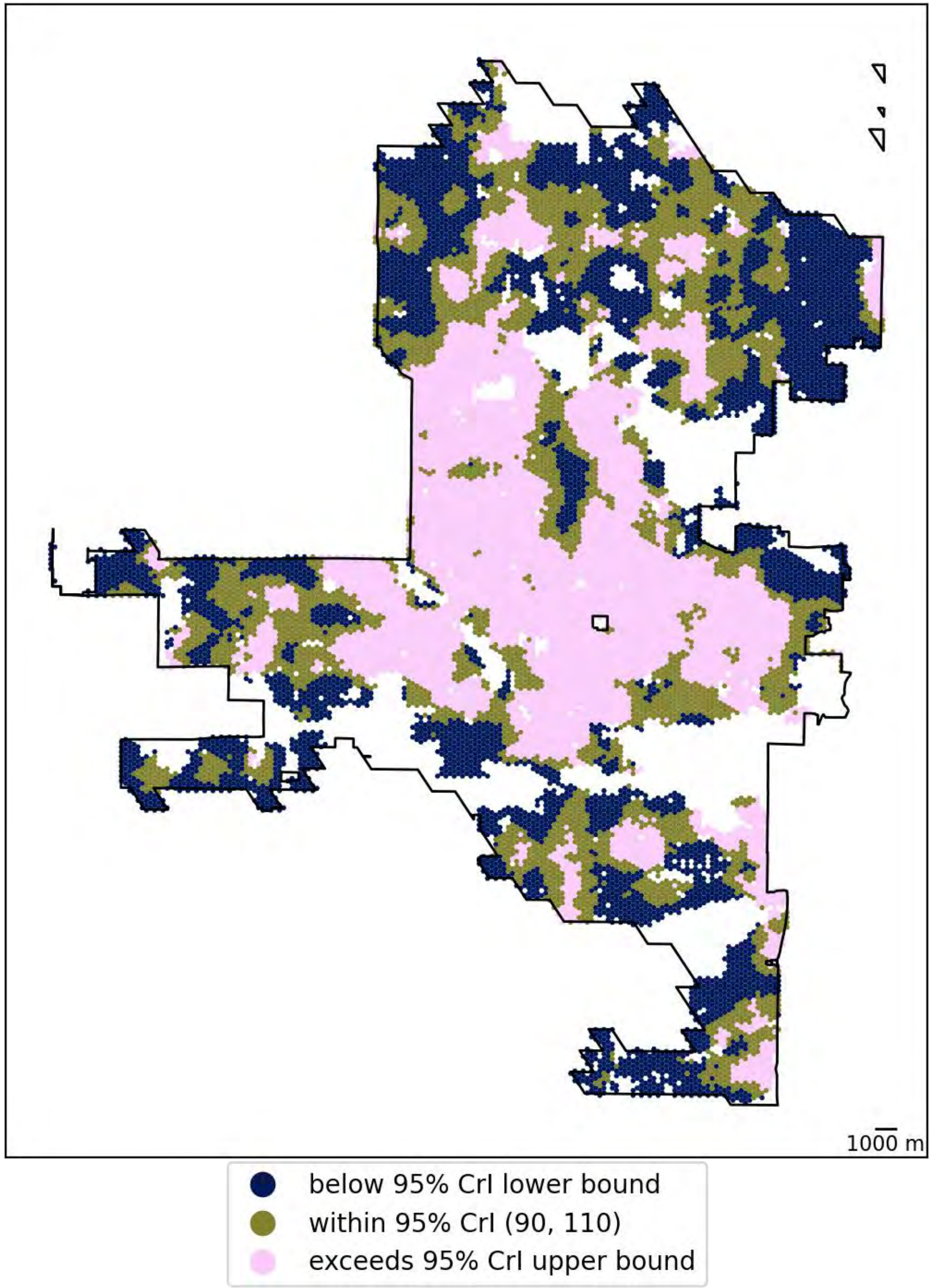

B: Estimated Mean 1000 m neighbourhood street intersections per km² requirement for reaching the WHO's target of a ≥15% relative reduction in insufficient physical activity through walking

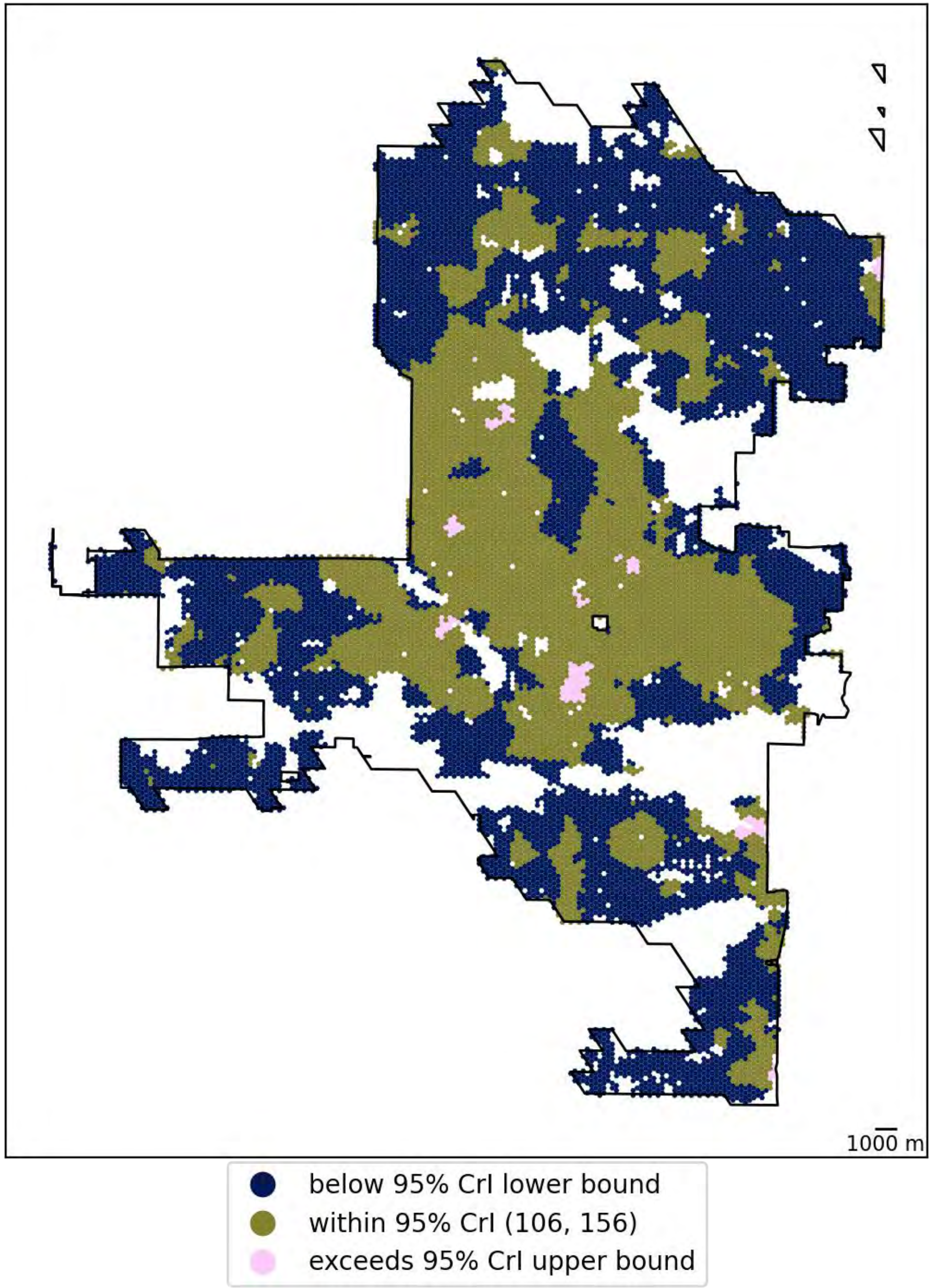

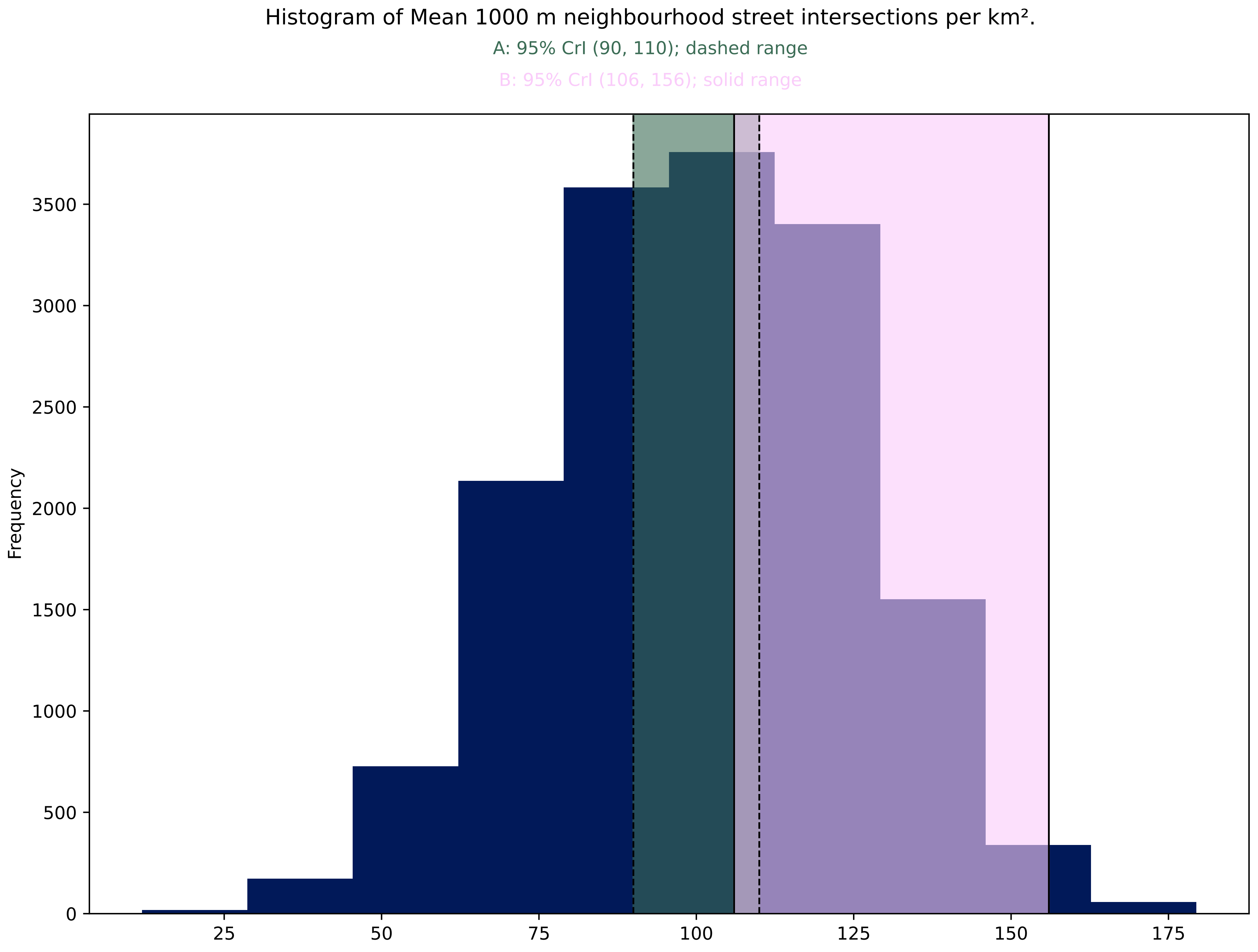
Histogram of Mean 1000 m neighbourhood street intersections per km².
A: 95% CrI (90, 110); dashed range
B: 95% CrI (106, 156); solid range
Frequency
0
500
1000
1500
2000
2500
3000
3500
25
50
75
100
125
150
175

Distance to nearest public transport stops (m; up to 500m)

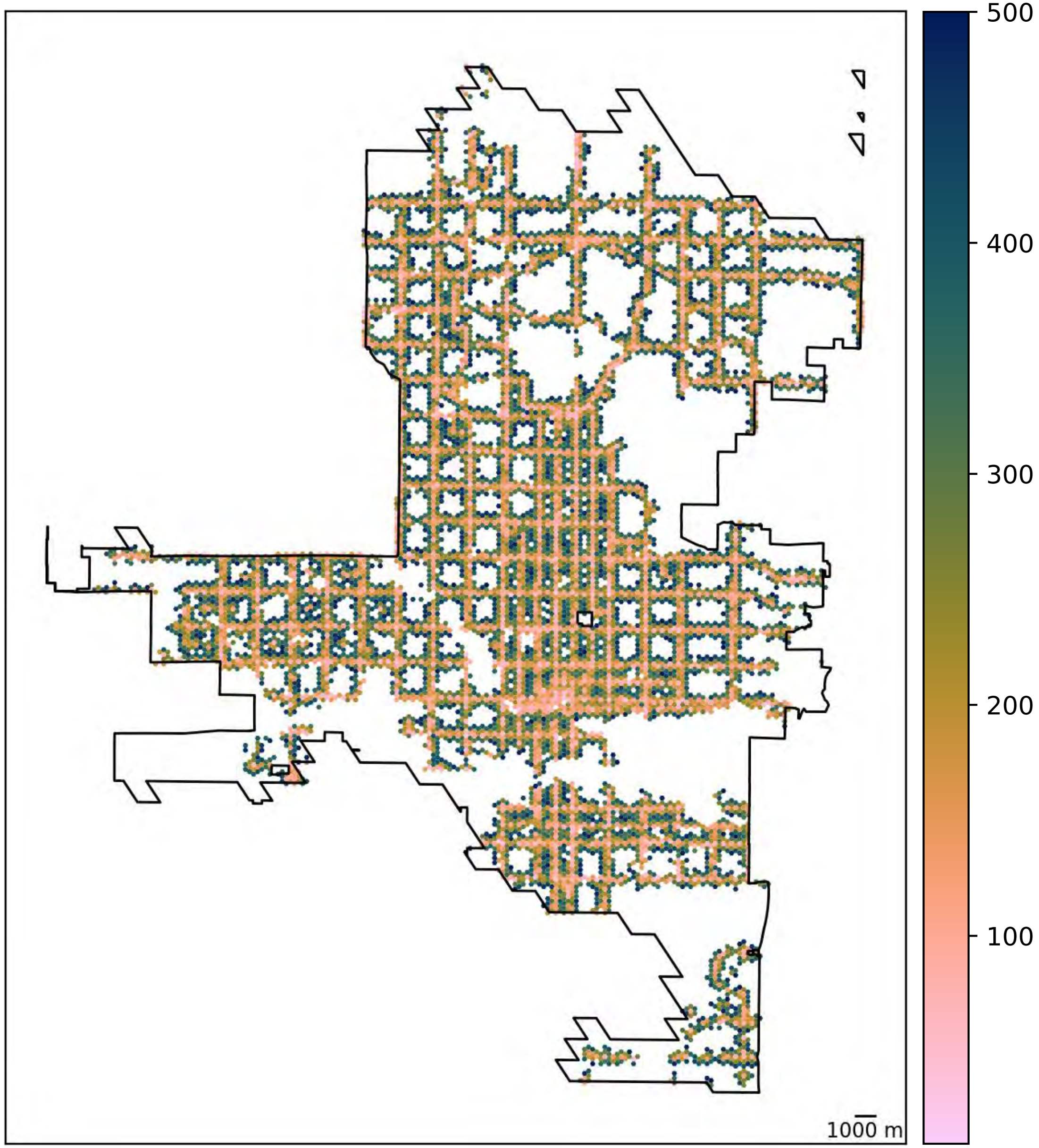

distances: Estimated Distance to nearest public transport stops (m; up to 500m) requirement for distances to destinations, measured up to a maximum distance target threshold of 500 metres

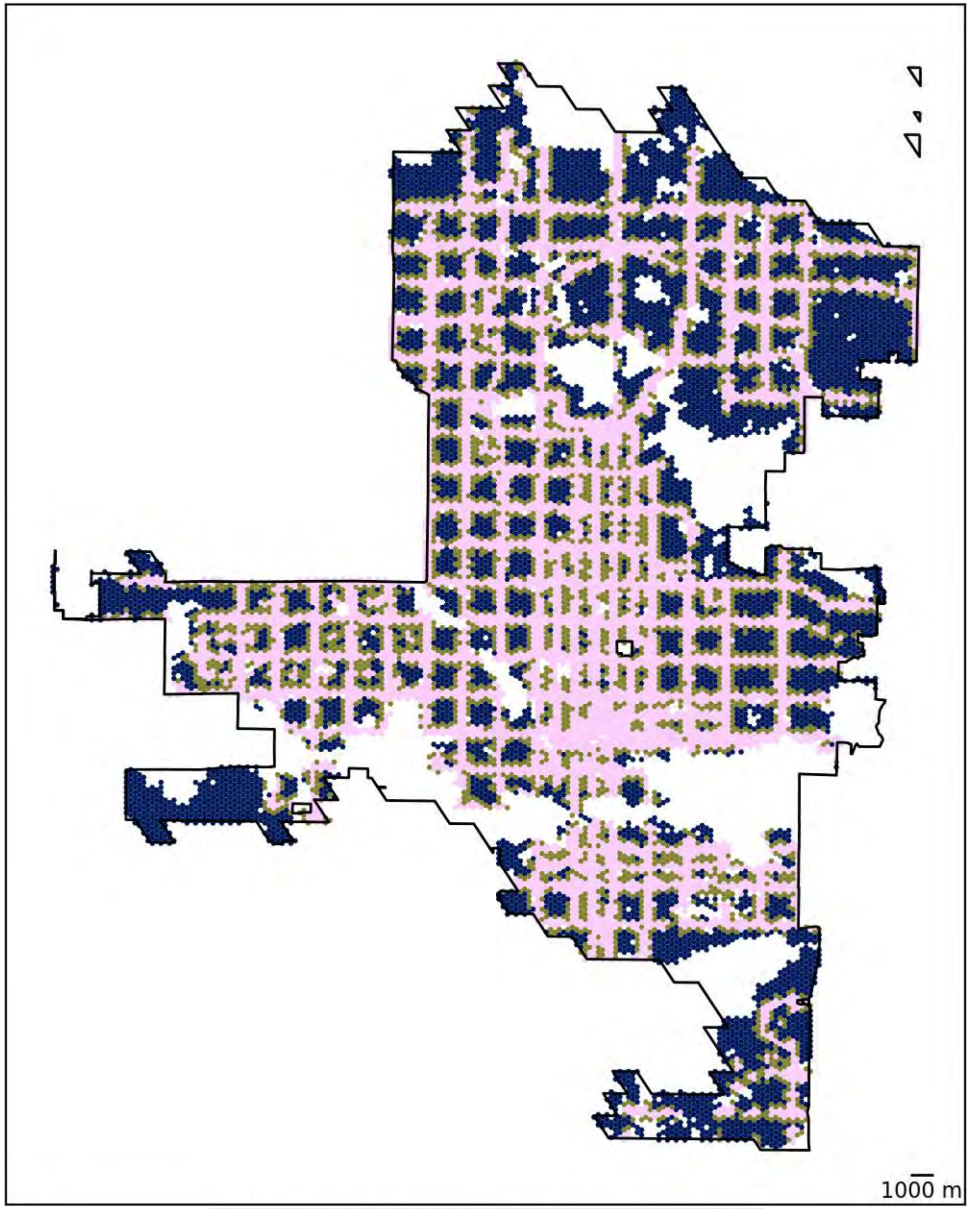

below distance (m) lower bound
within distance (m) (300, 500)
exceeds distance (m) upper bound

Histogram of Distance to nearest public transport stops (m; up to 500m).

distances: distance (m) (300, 500); dashed range

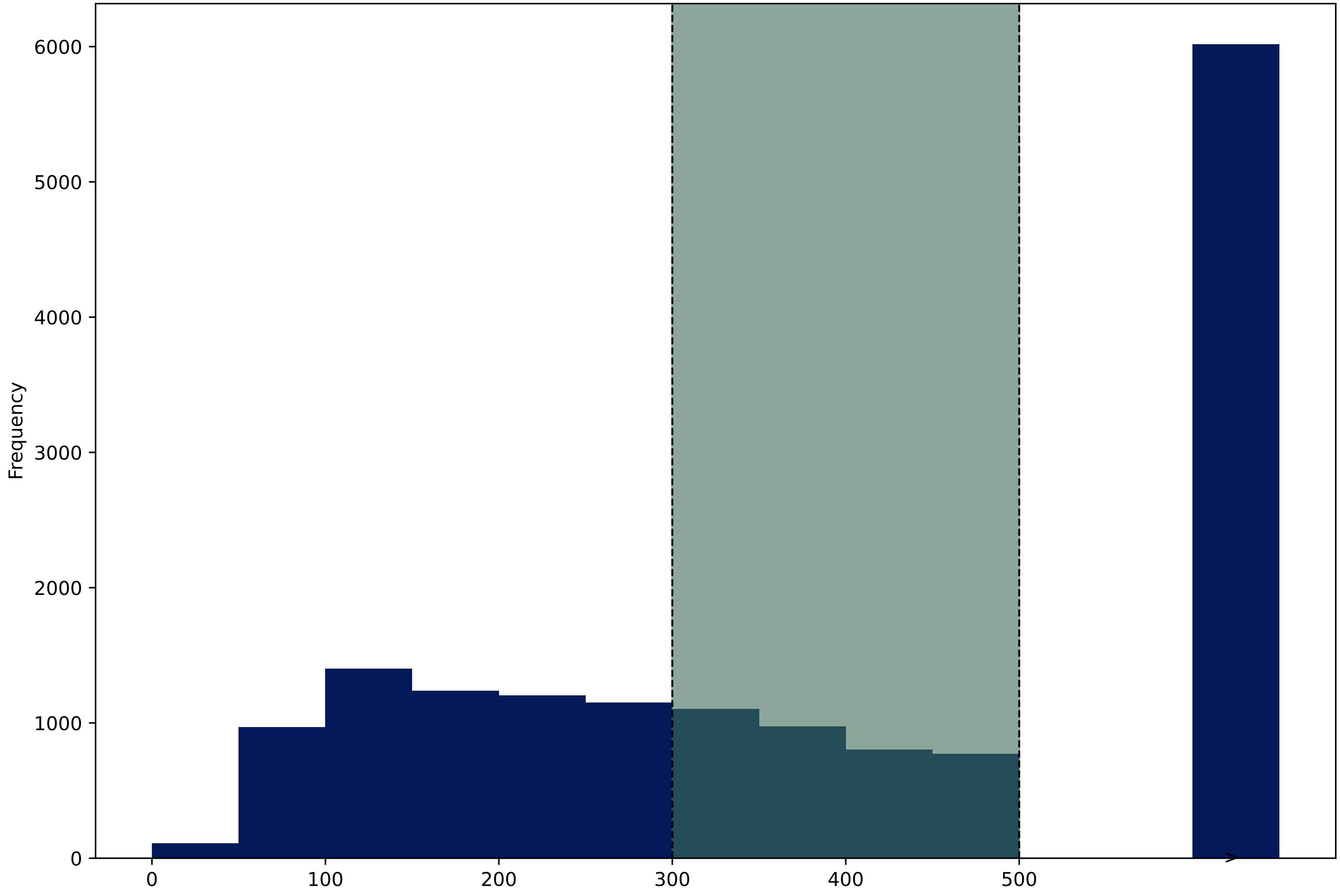

Distance to nearest park (m; up to 500m)

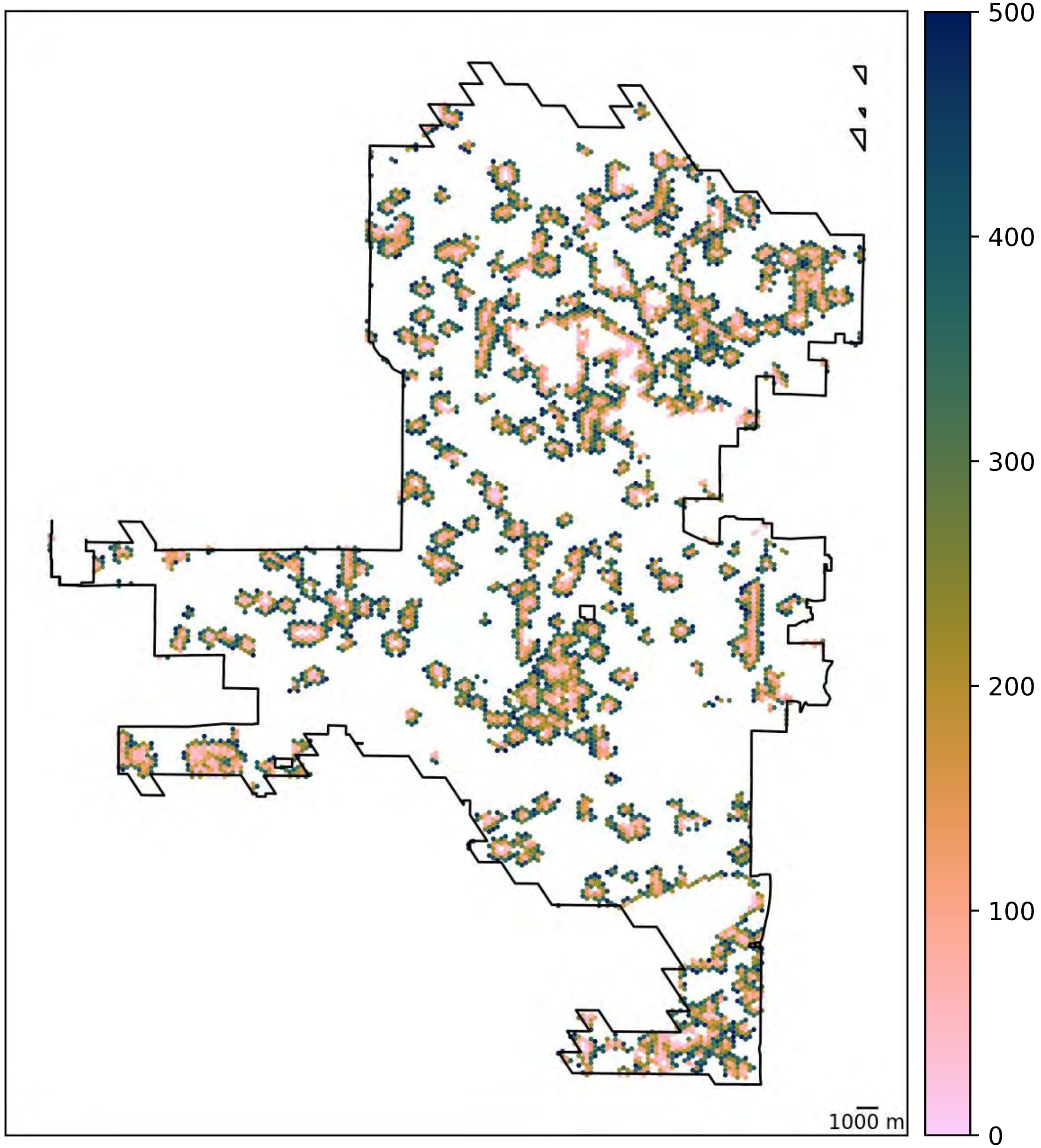

distances: Estimated Distance to nearest park (m; up to 500m) requirement for distances to destinations, measured up to a maximum distance target threshold of 500 metres

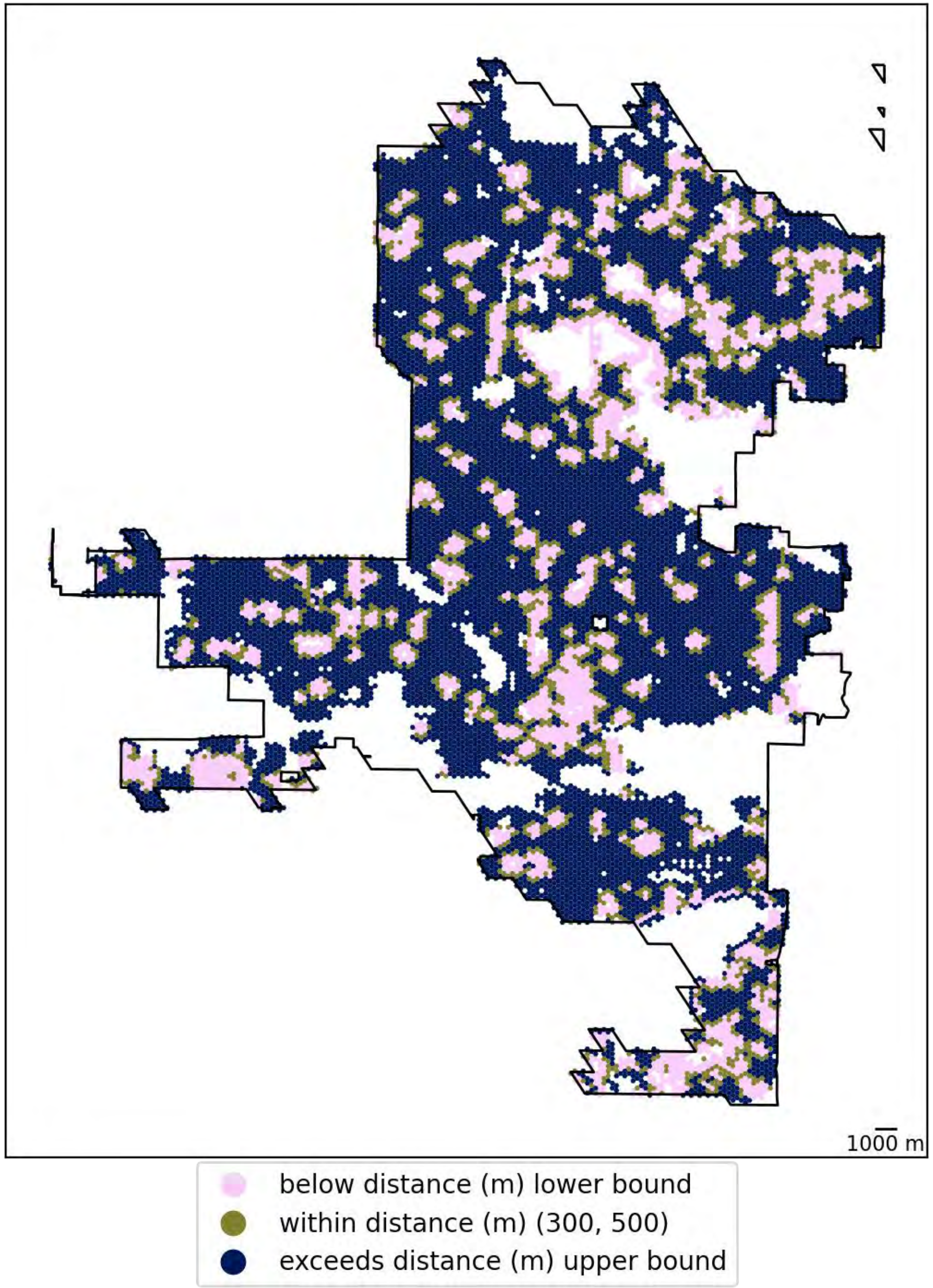

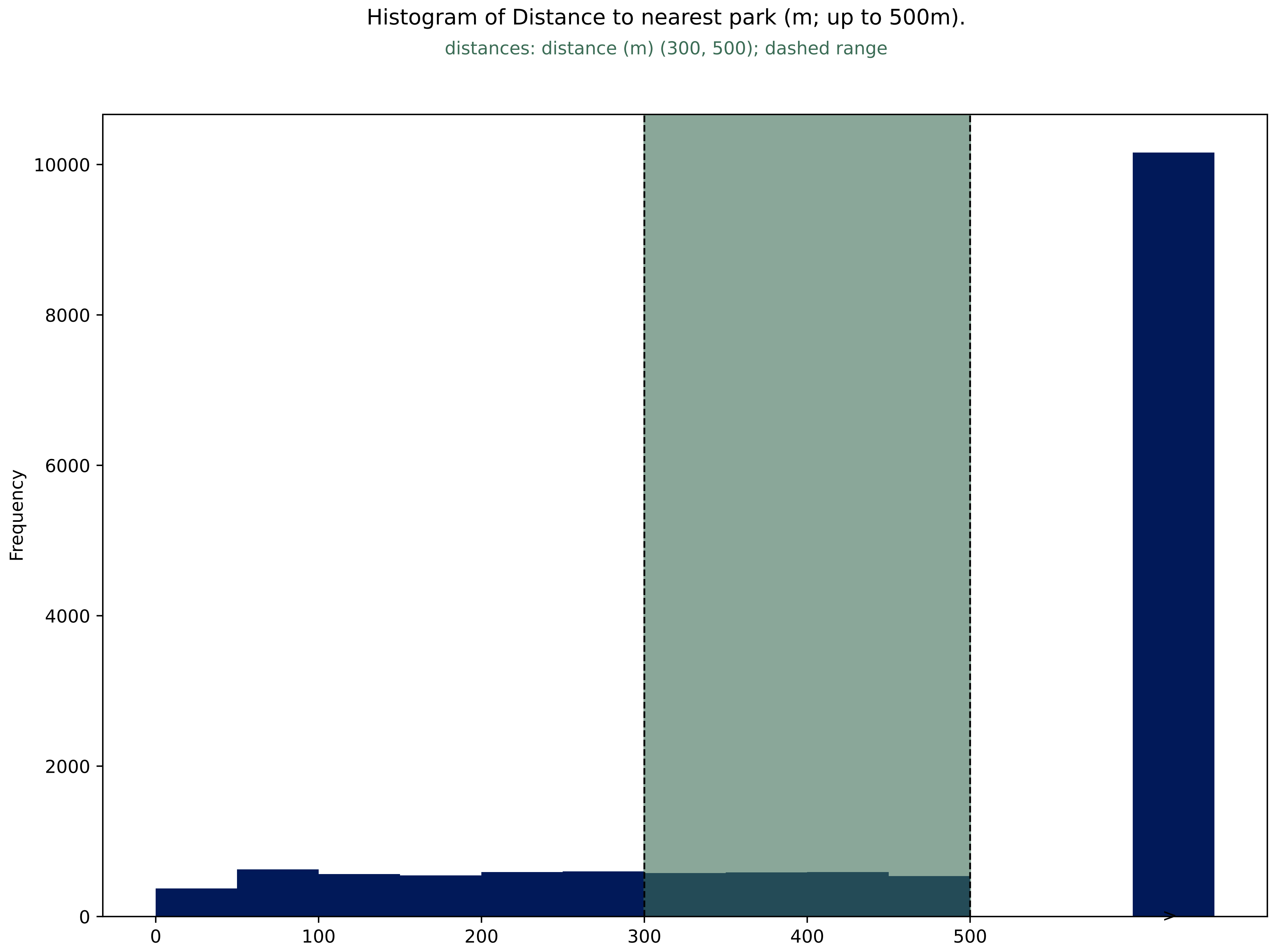
Histogram of Distance to nearest park (m; up to 500m).
distances: distance (m) (300, 500); dashed range
Frequency
10000
8000
6000
4000
2000
0
0
100
200
300
400
500
>

America, North, United States, Seattle

Satellite imagery of urban study region (Bing) | Walkability, relative to city | Walkability, relative to 25 global cities

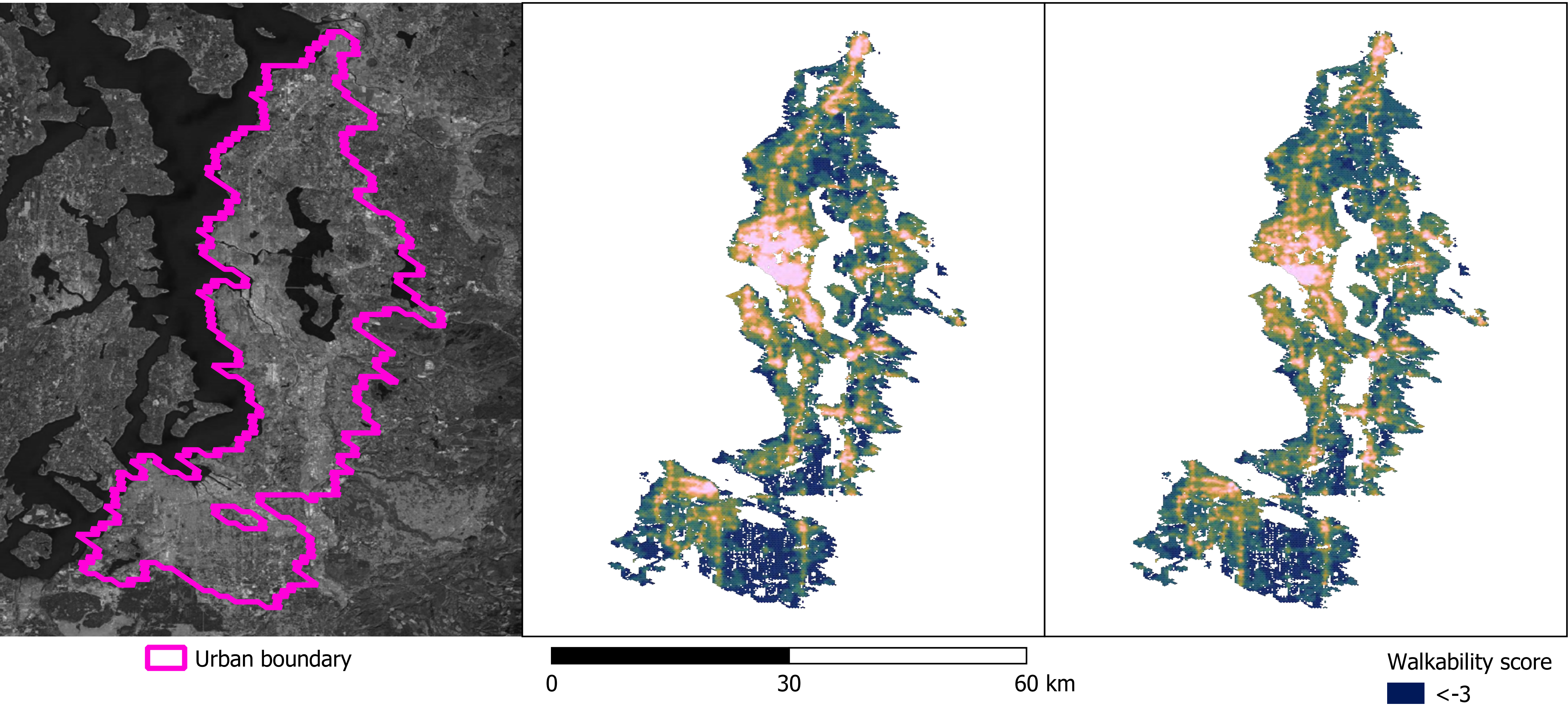

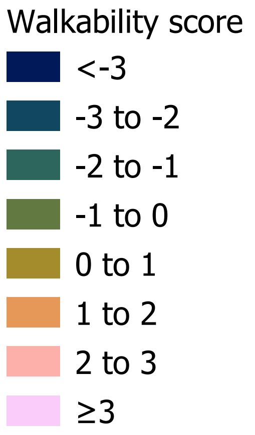

Walkability relative to all cities by component variables (2D histograms), and overall (histogram)

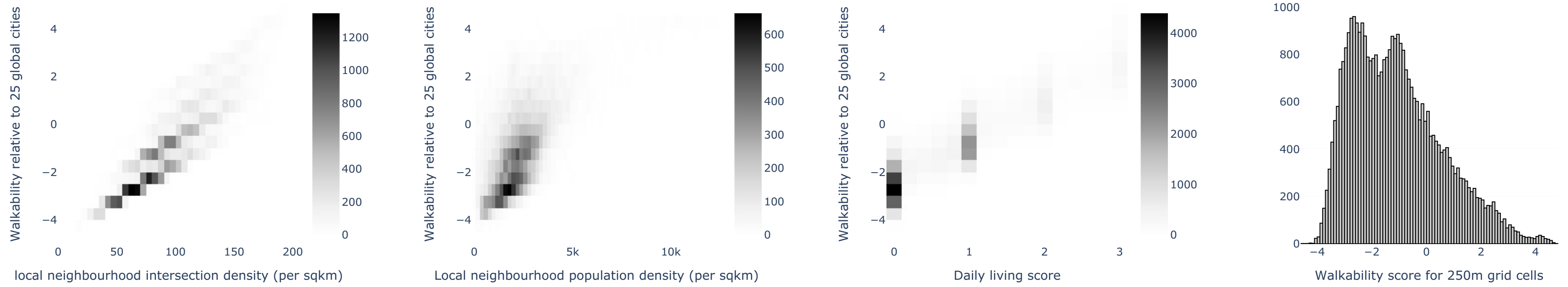

Mean 1000 m neighbourhood population per km²

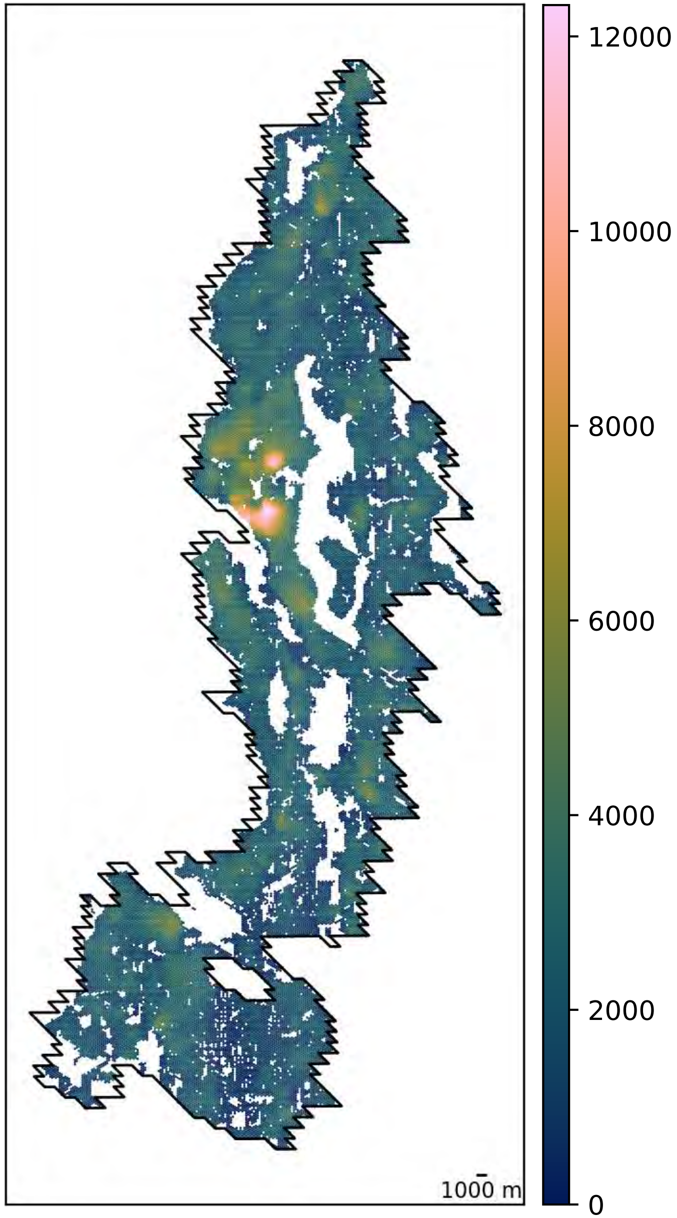

A: Estimated Mean 1000 m neighbourhood population per km² requirement for ≥80% probability of engaging in walking for transport

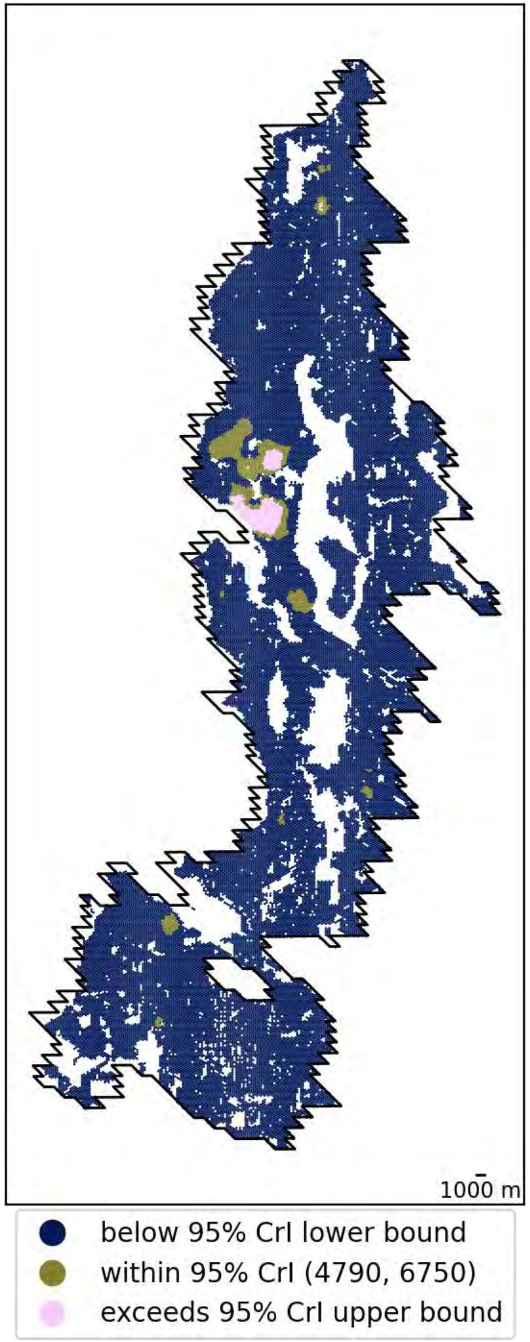

B: Estimated Mean 1000 m neighbourhood population per km² requirement for reaching the WHO's target of a ≥15% relative reduction in insufficient physical activity through walking

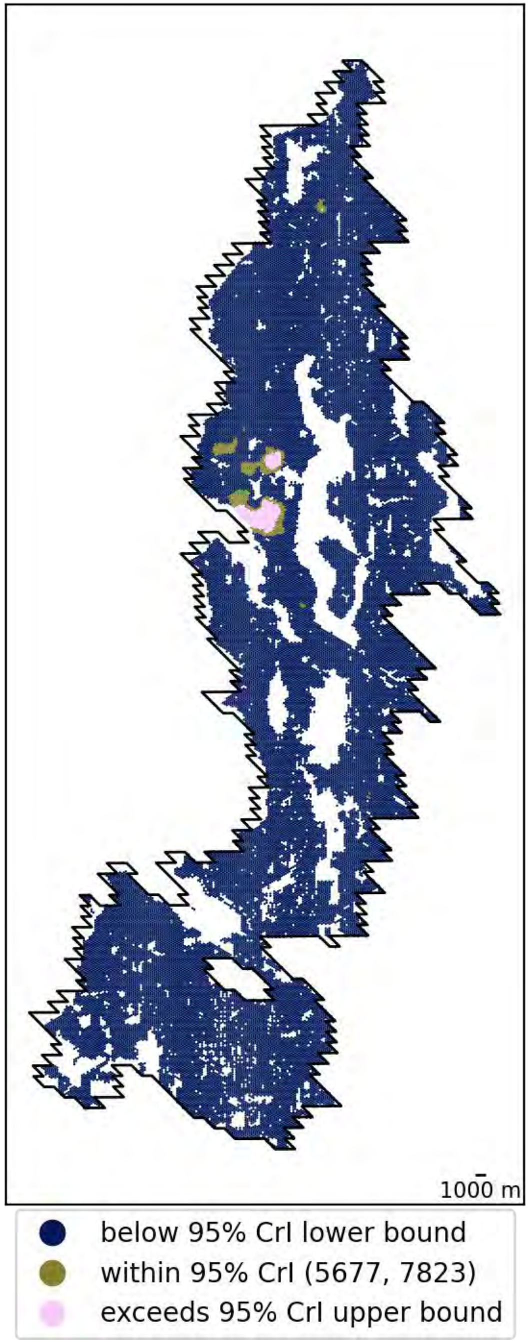

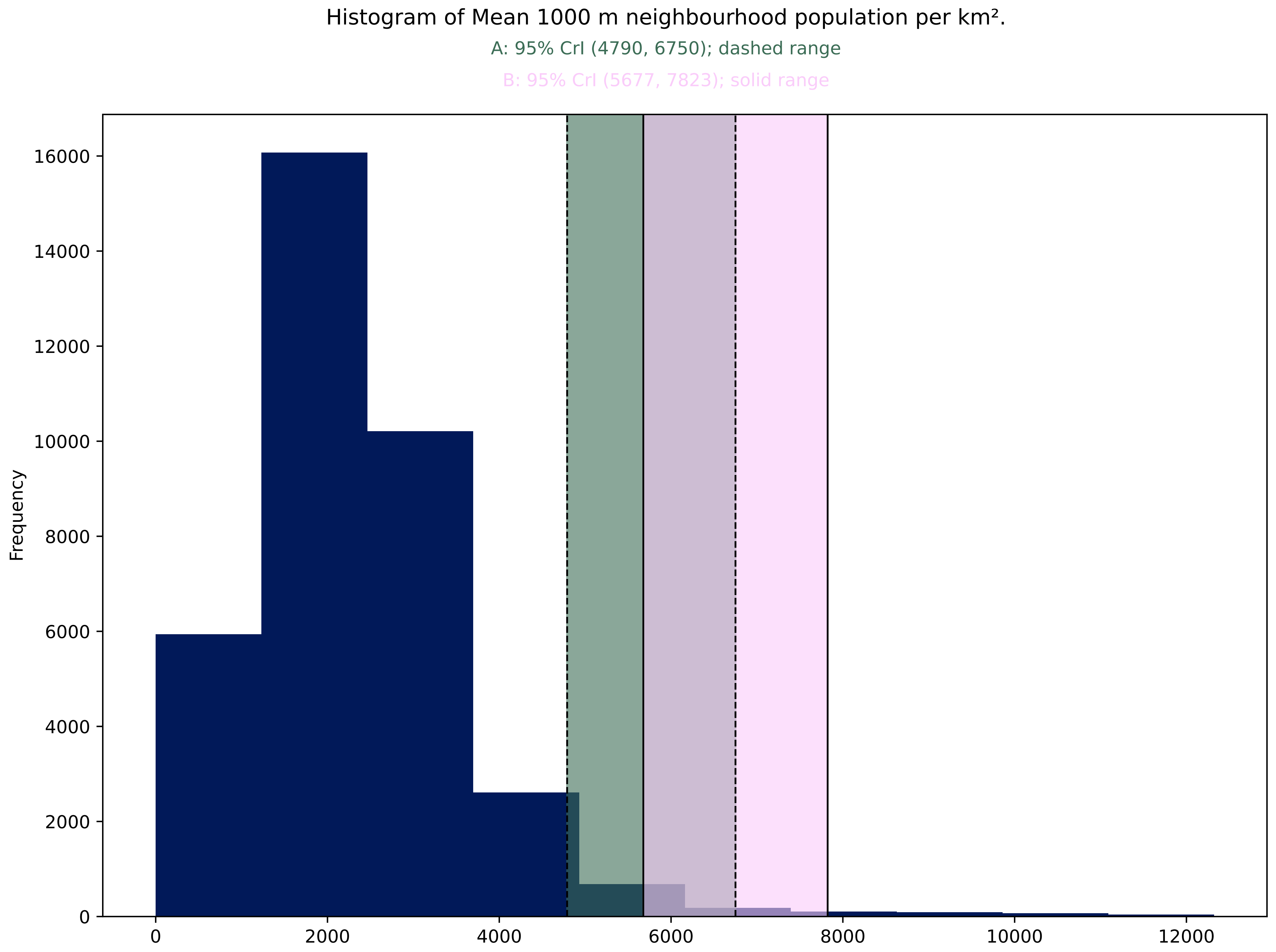

Histogram of Mean 1000 m neighbourhood population per km².
A: 95% CrI (4790, 6750); dashed range
B: 95% CrI (5677, 7823); solid range
Frequency
0
2000
4000
6000
8000
10000
12000
14000
16000

Mean 1000 m neighbourhood street intersections per km²

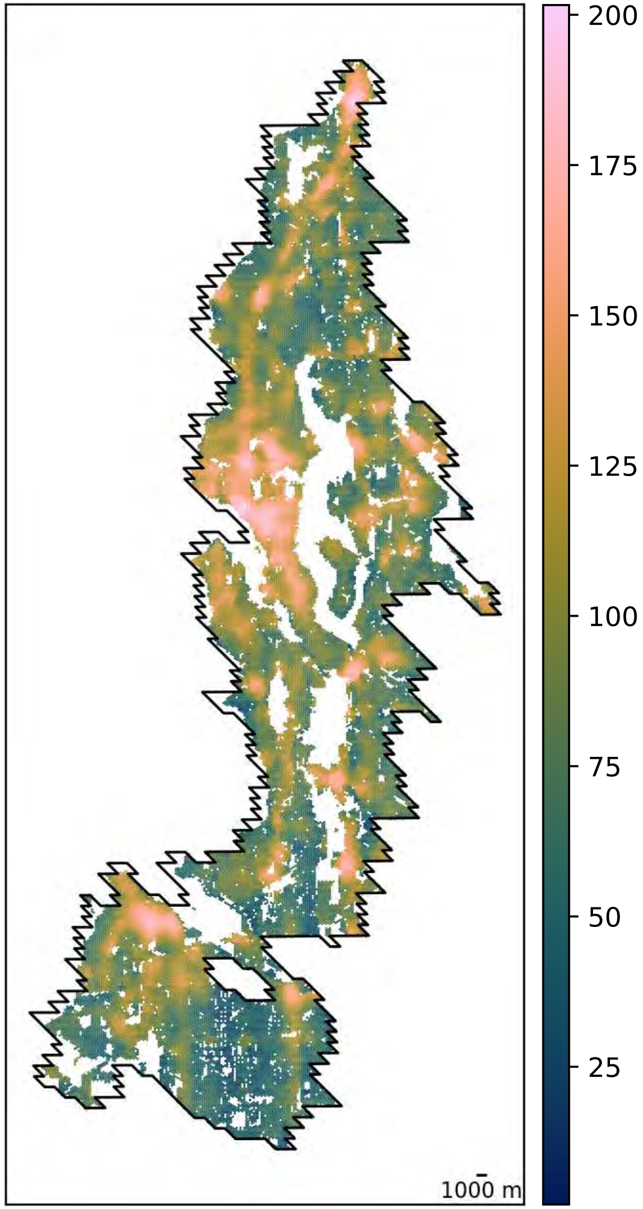

A: Estimated Mean 1000 m neighbourhood street intersections per km² requirement for ≥80% probability of engaging in walking for transport

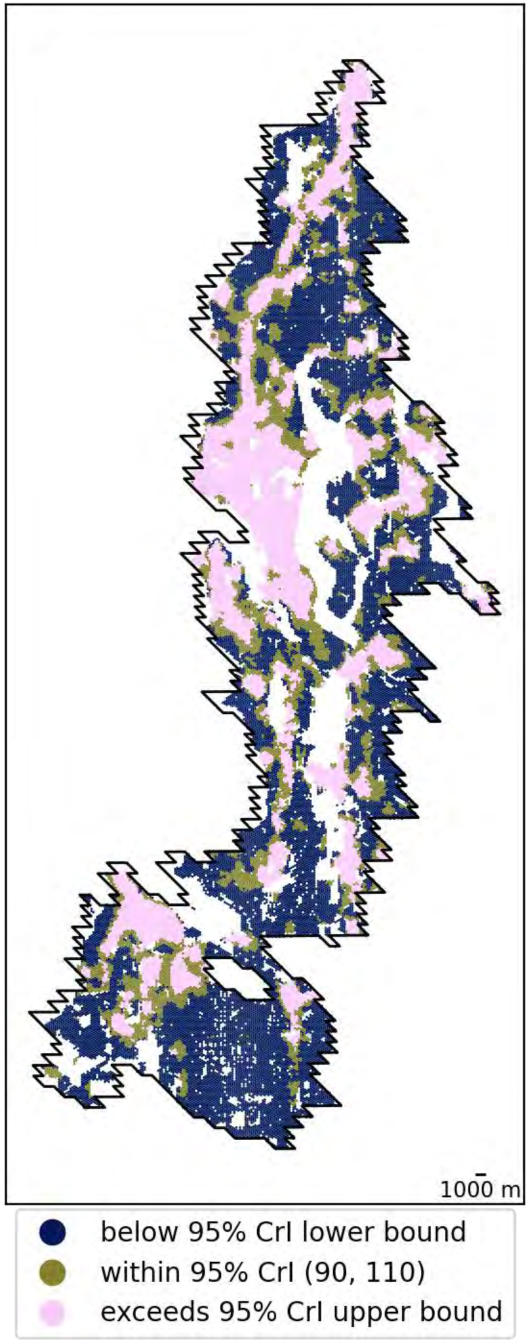

B: Estimated Mean 1000 m neighbourhood street intersections per km² requirement for reaching the WHO's target of a ≥15% relative reduction in insufficient physical activity through walking

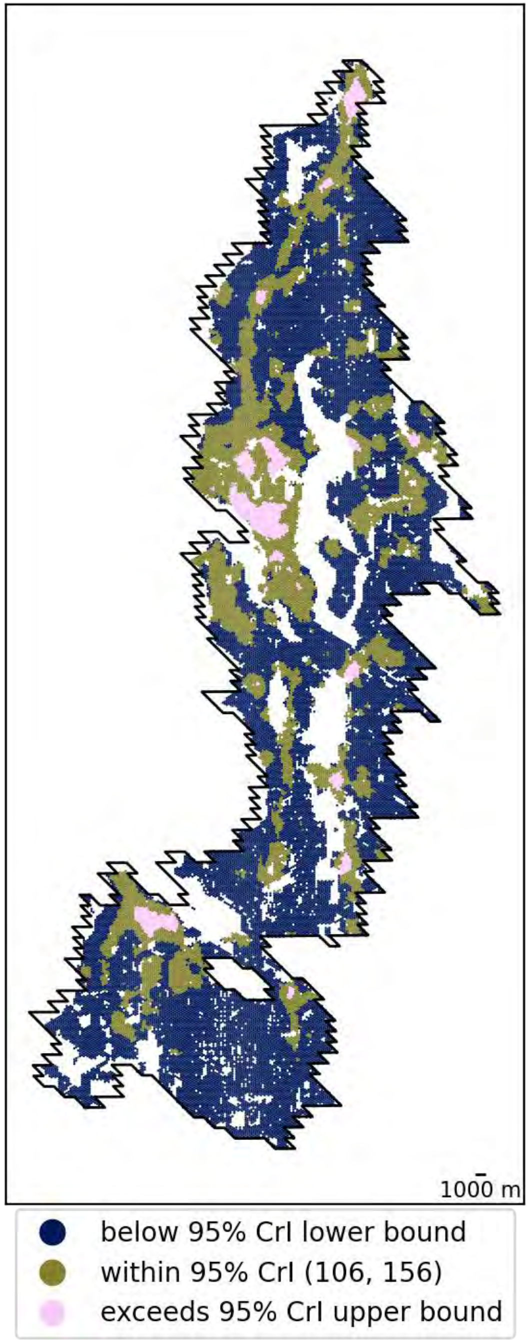

Histogram of Mean 1000 m neighbourhood street intersections per km².

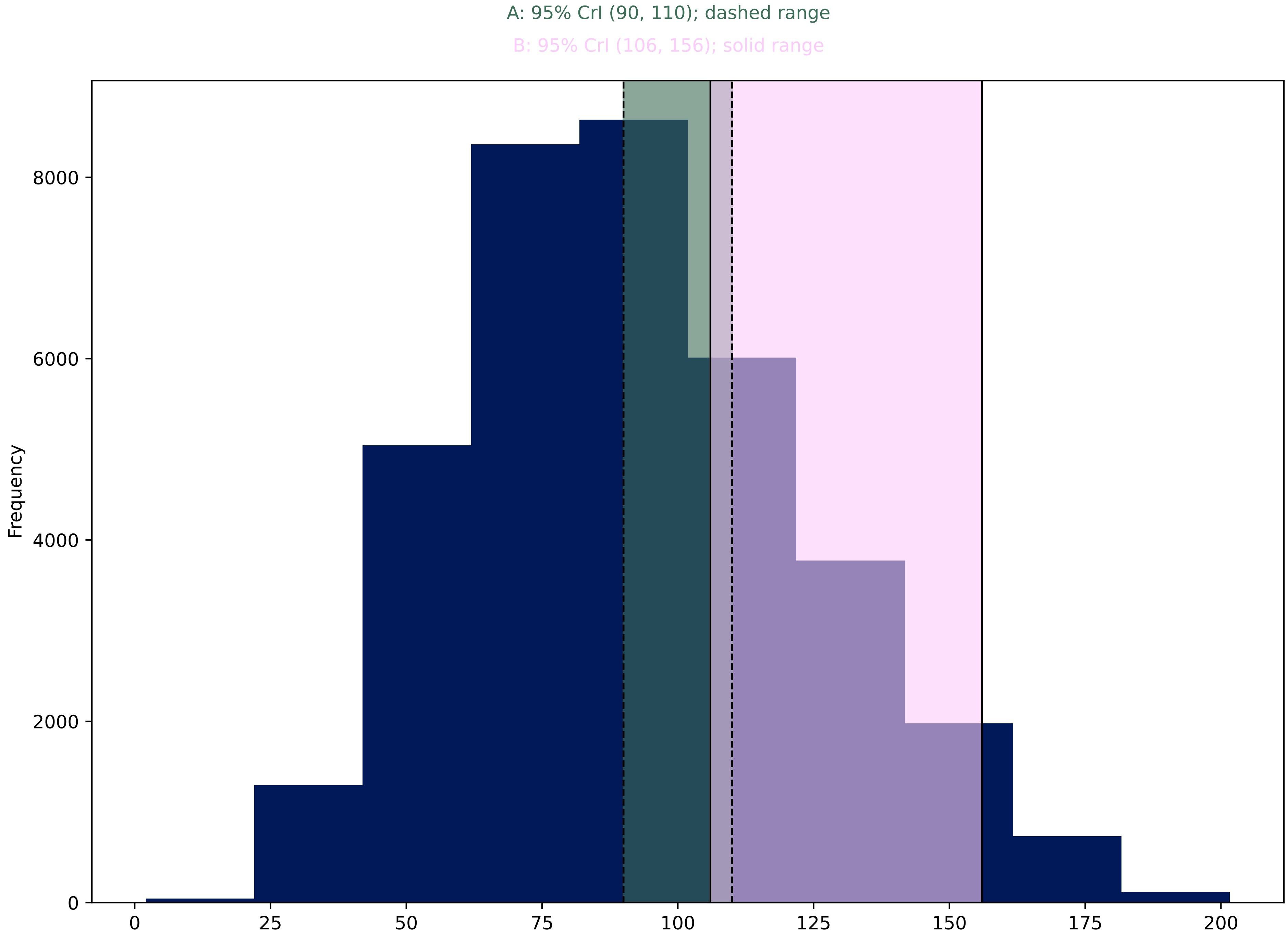

Distance to nearest public transport stops (m; up to 500m)

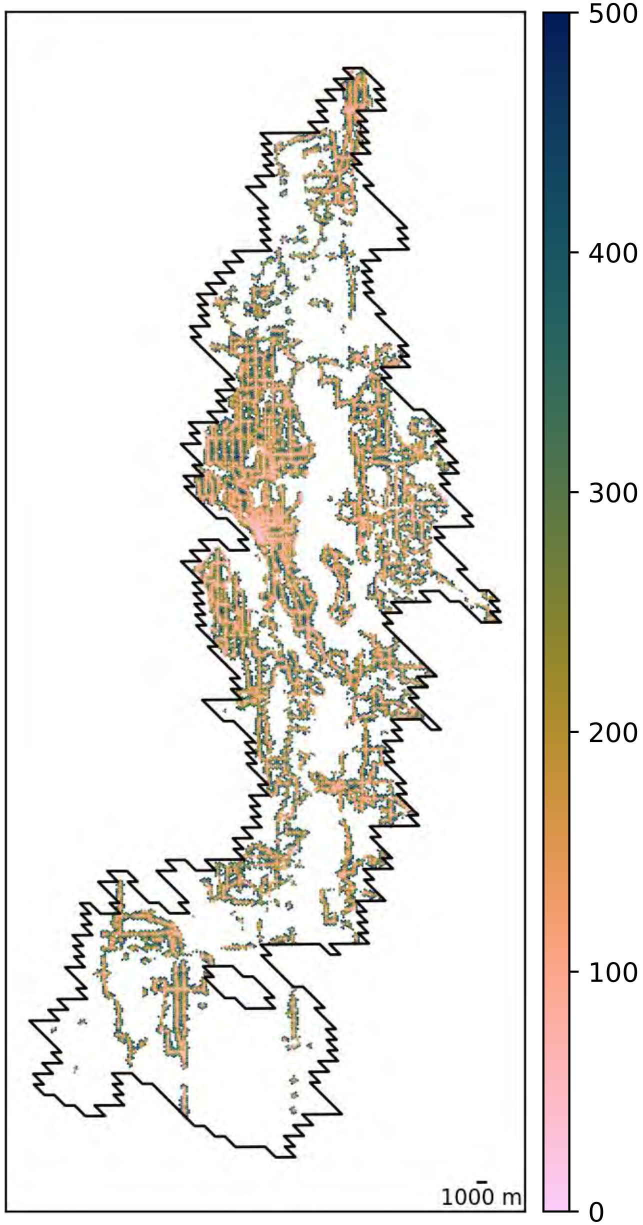

distances: Estimated Distance to nearest public transport stops (m; up to 500m) requirement for distances to destinations, measured up to a maximum distance target threshold of 500 metres

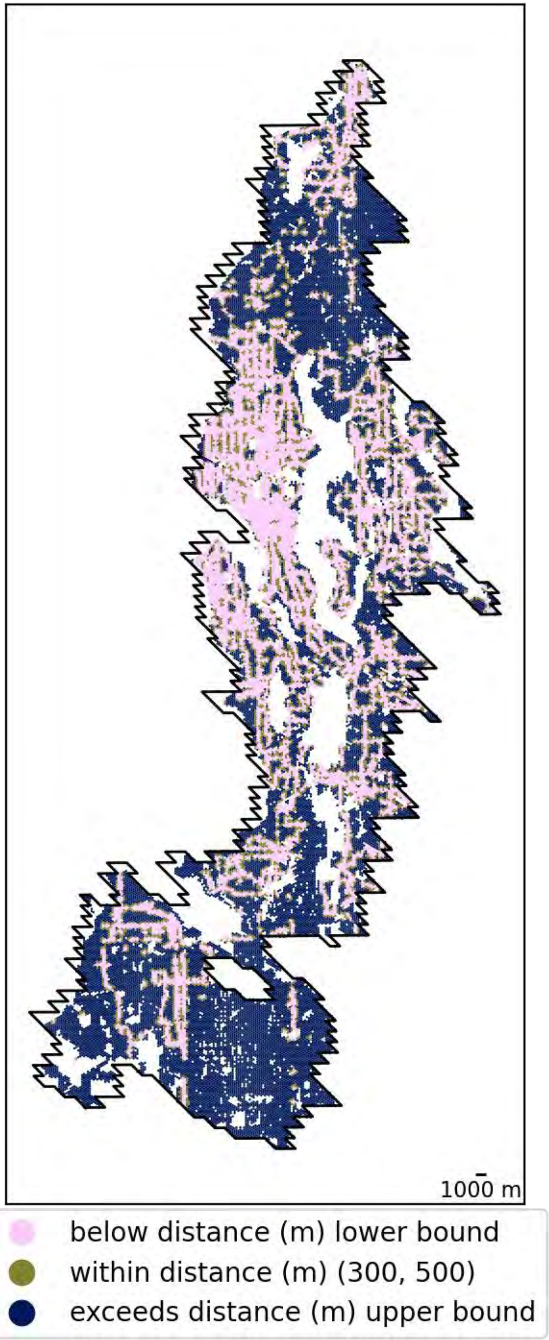

Histogram of Distance to nearest public transport stops (m; up to 500m).

distances: distance (m) (300, 500); dashed range

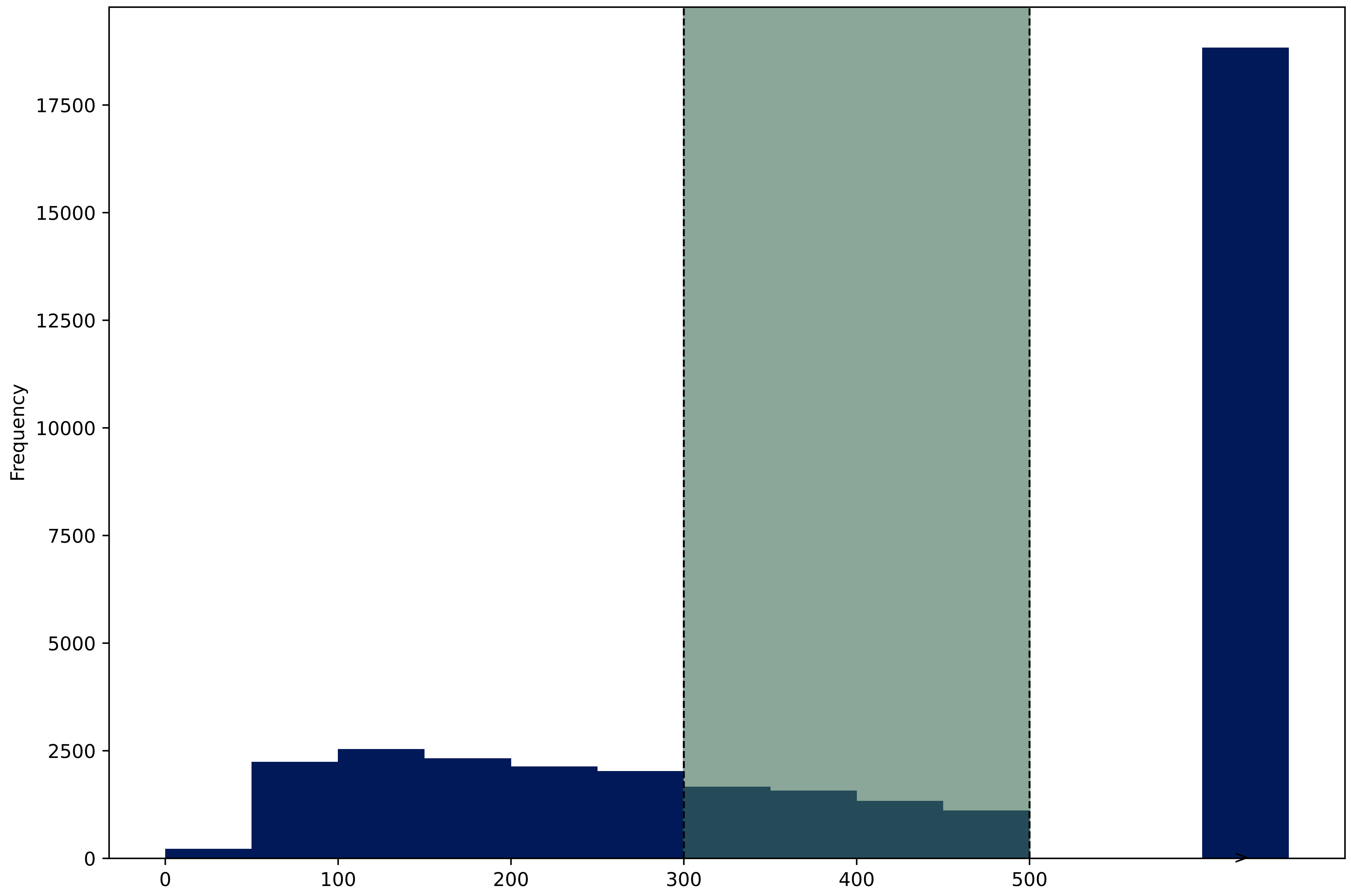

Distance to nearest park (m; up to 500m)

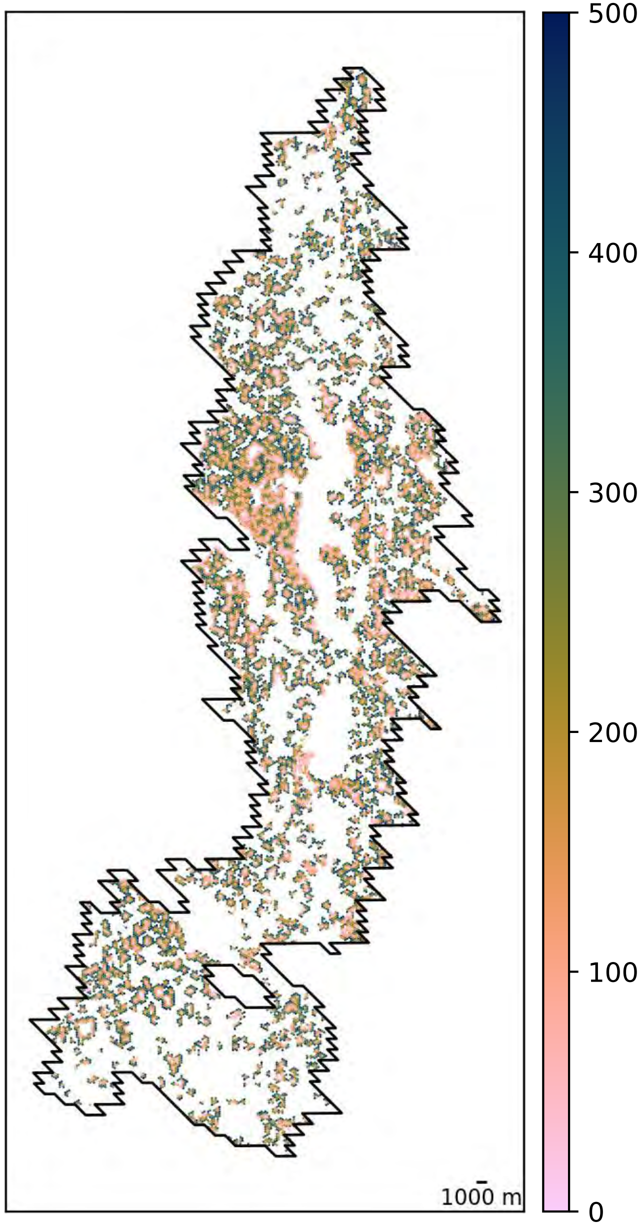

distances: Estimated Distance to nearest park (m; up to 500m) requirement for distances to destinations, measured up to a maximum distance target threshold of 500 metres

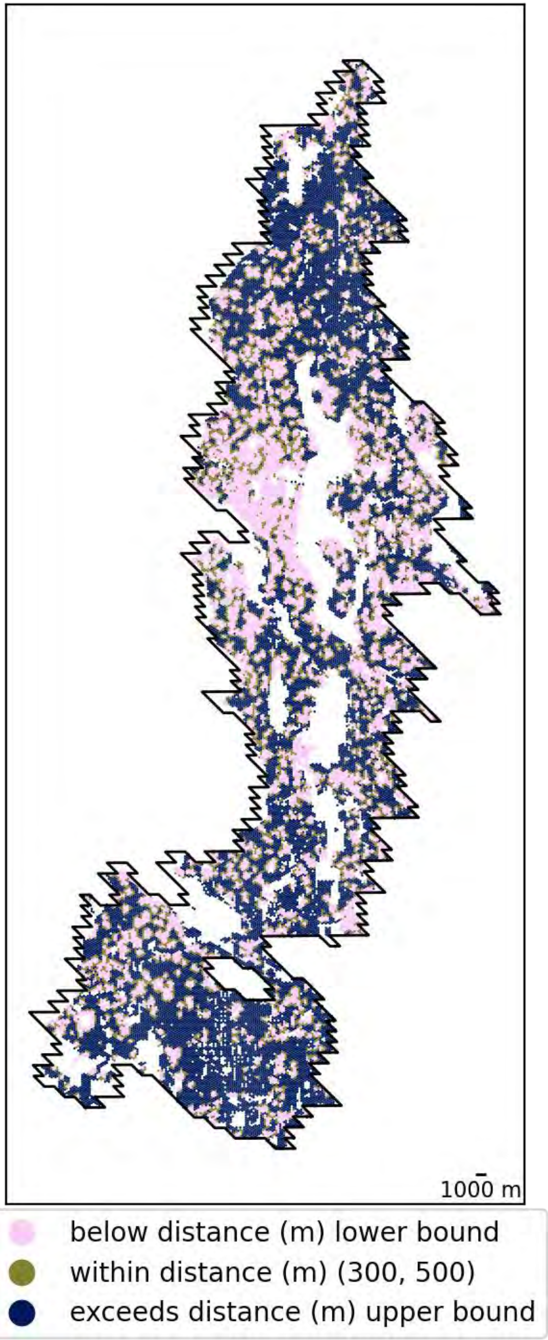

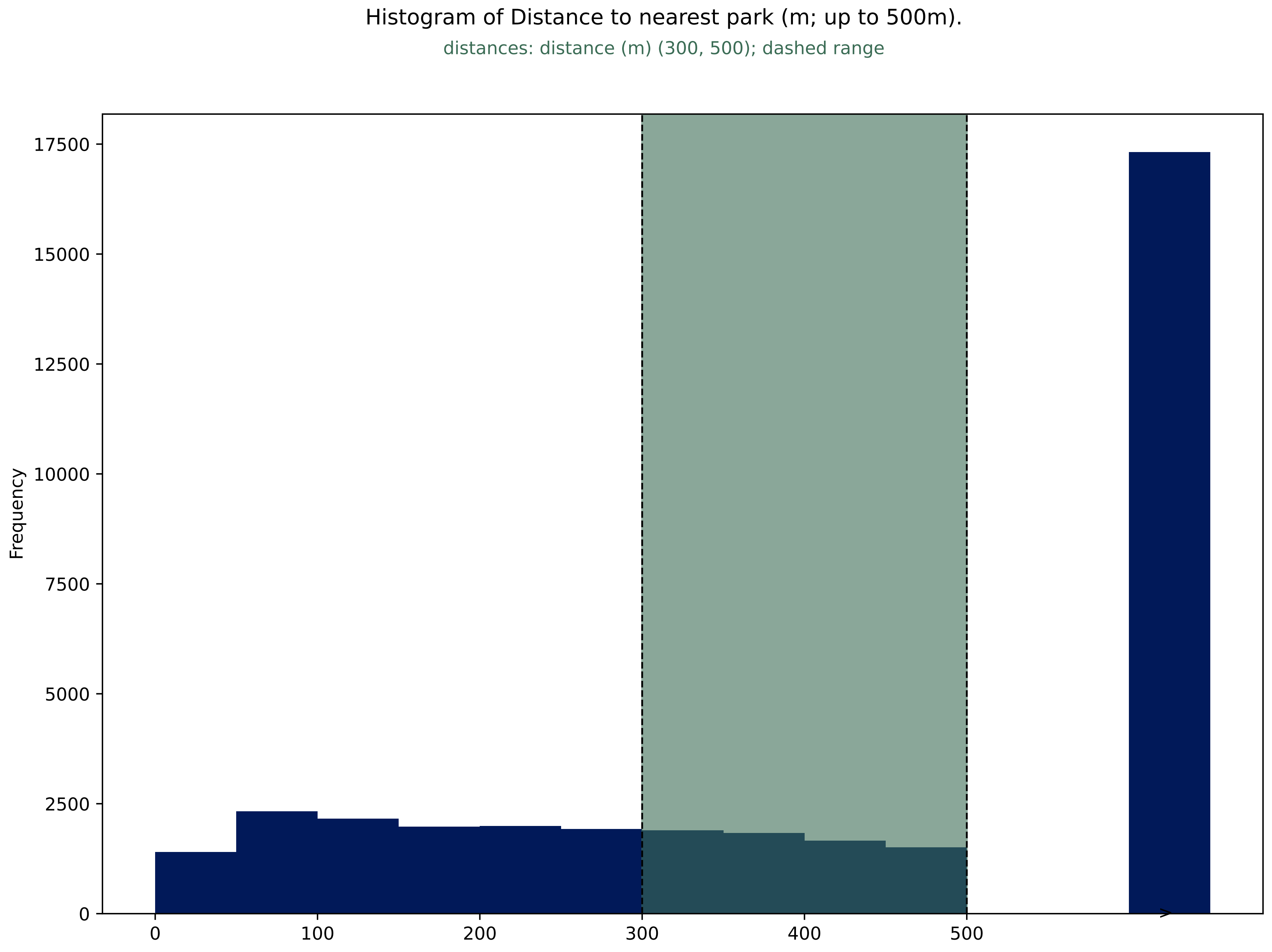

Histogram of Distance to nearest park (m; up to 500m).
distances: distance (m) (300, 500); dashed range
Frequency
17500
15000
12500
10000
7500
5000
2500
0
0
100
200
300
400
500
>

America, South, Brazil, São Paulo

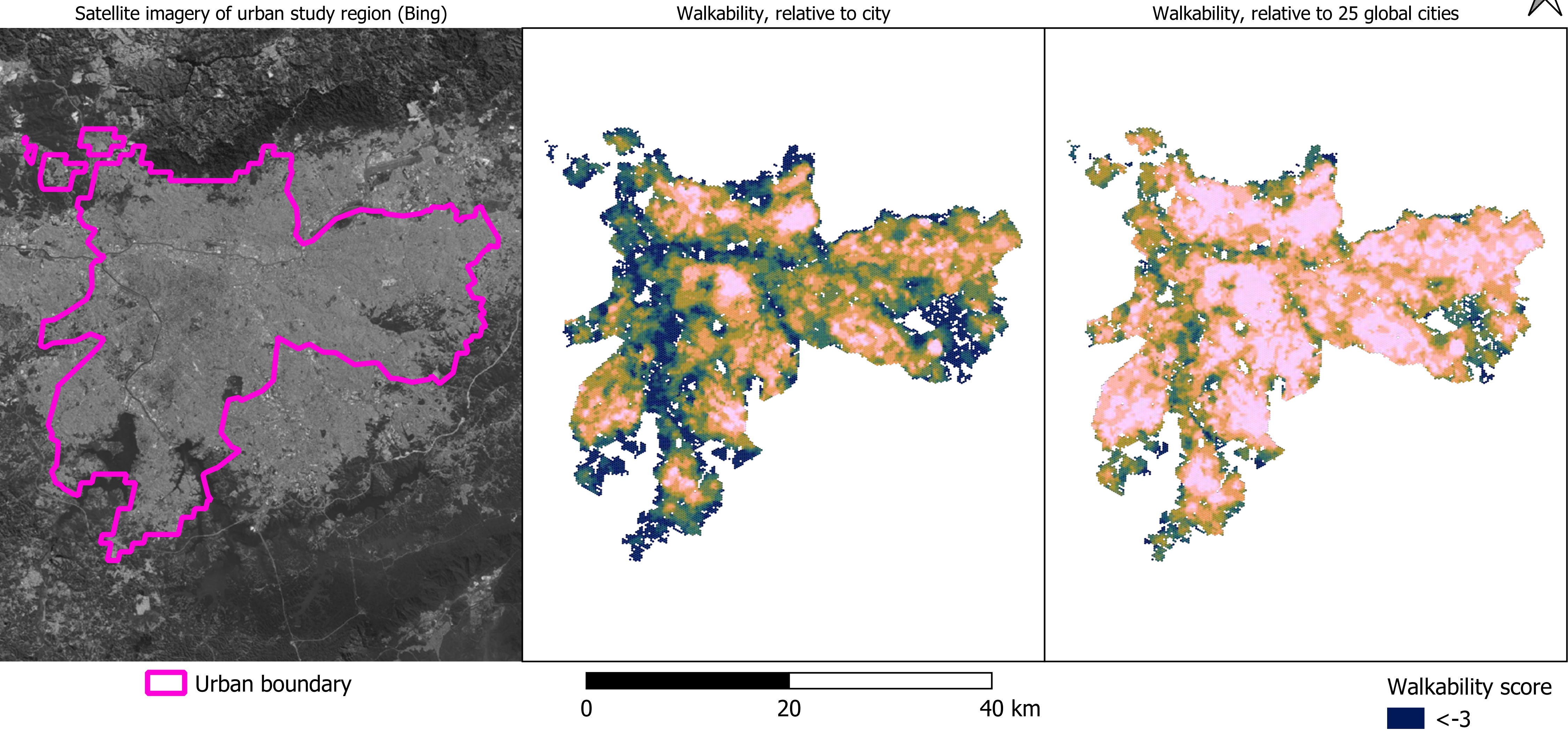

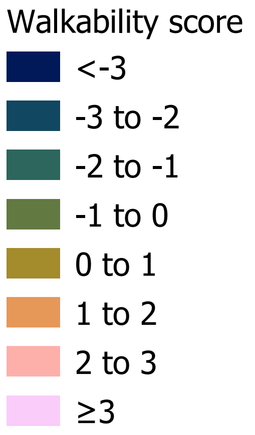

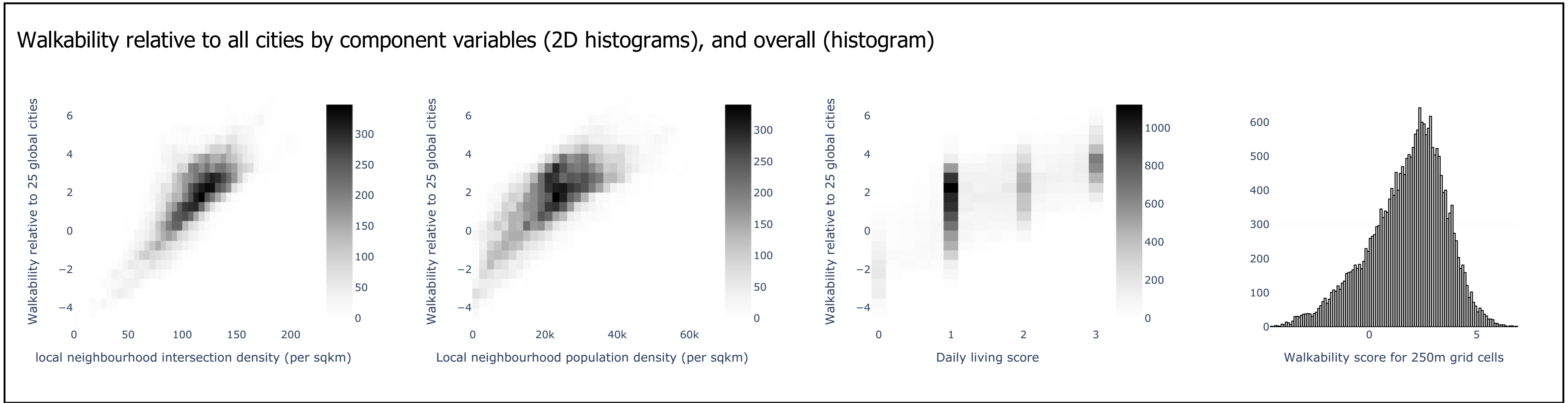

Mean 1000 m neighbourhood population per km²

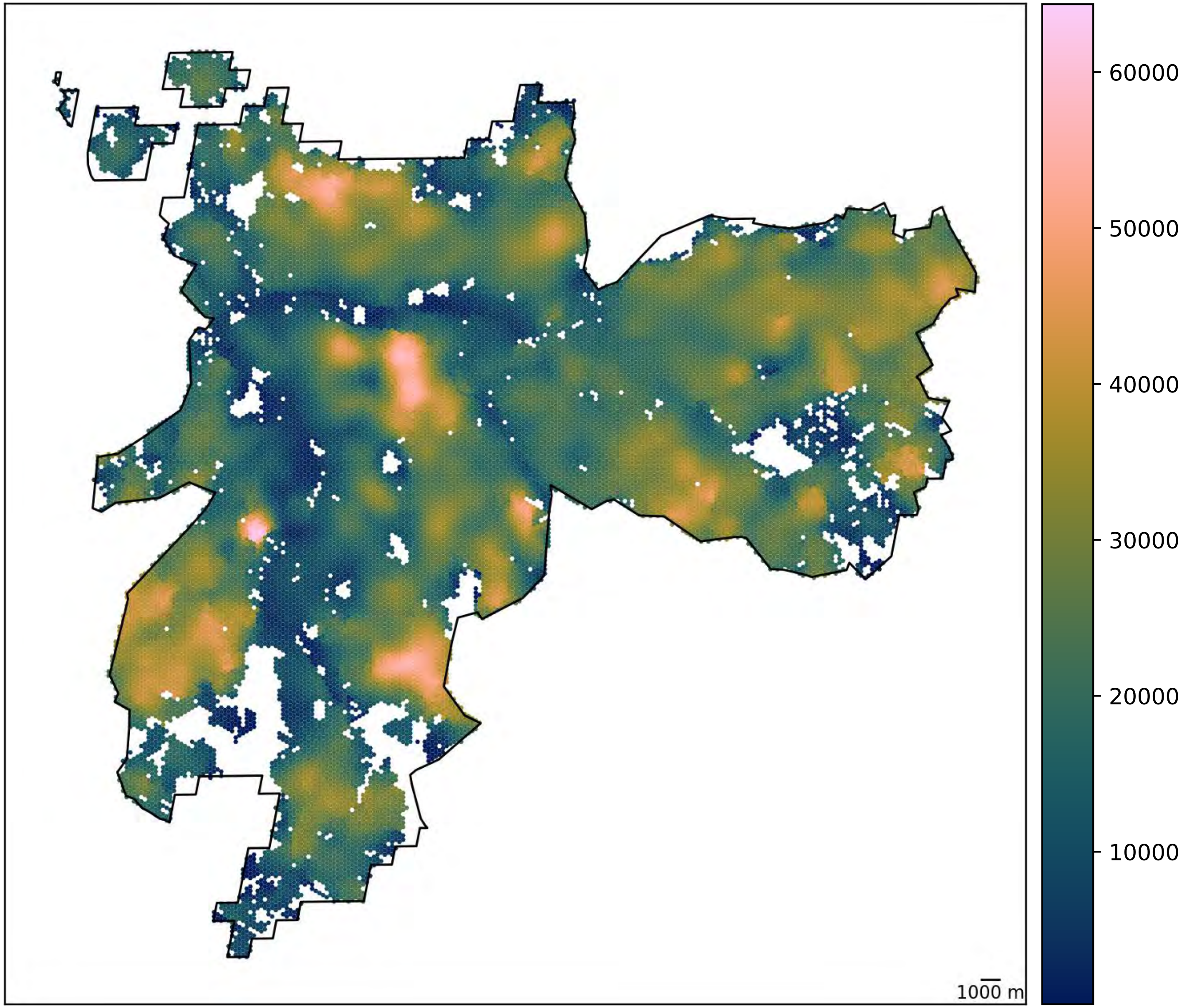

A: Estimated Mean 1000 m neighbourhood population per km² requirement for ≥80% probability of engaging in walking for transport

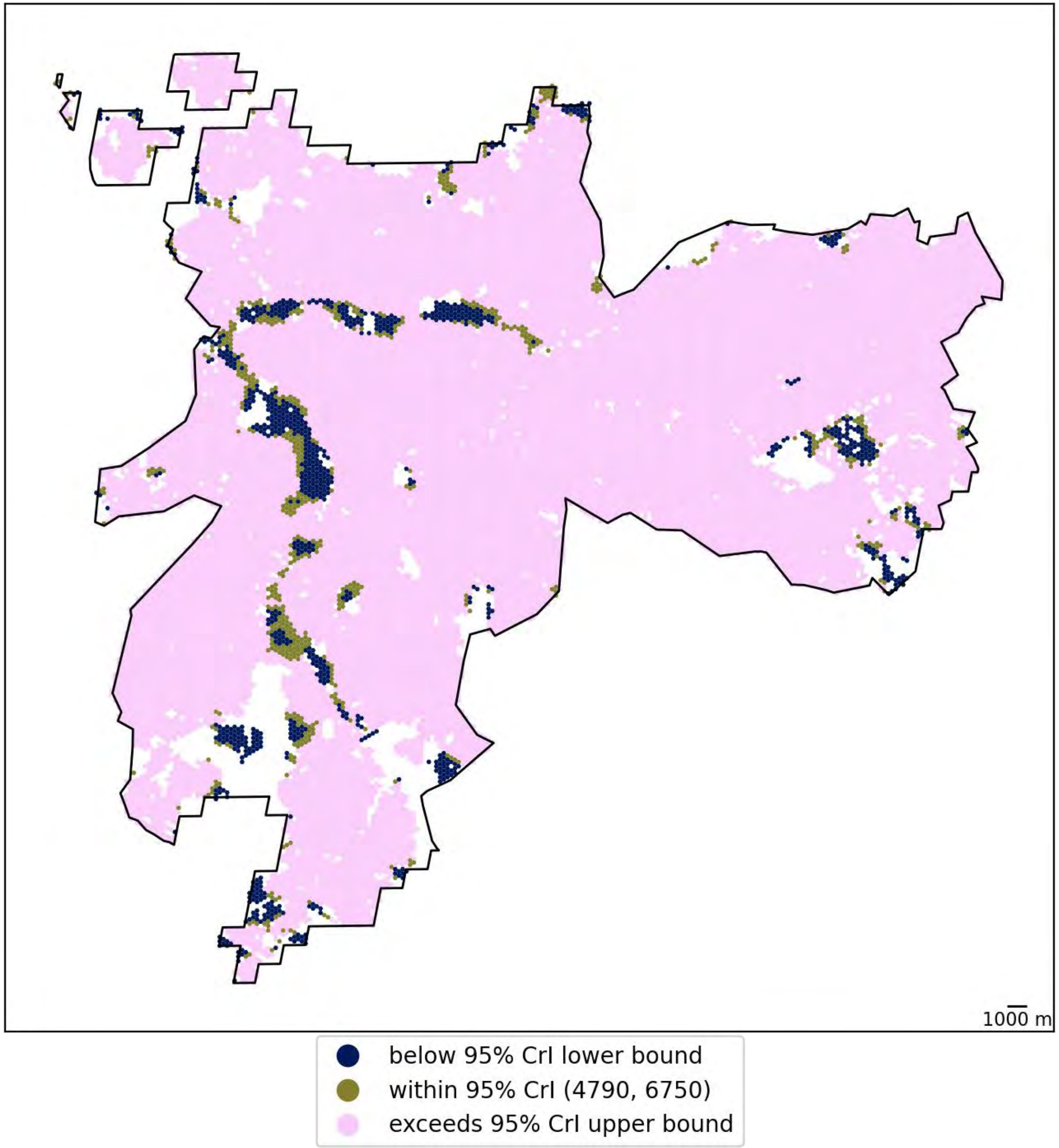

B: Estimated Mean 1000 m neighbourhood population per km² requirement for reaching the WHO's target of a ≥15% relative reduction in insufficient physical activity through walking

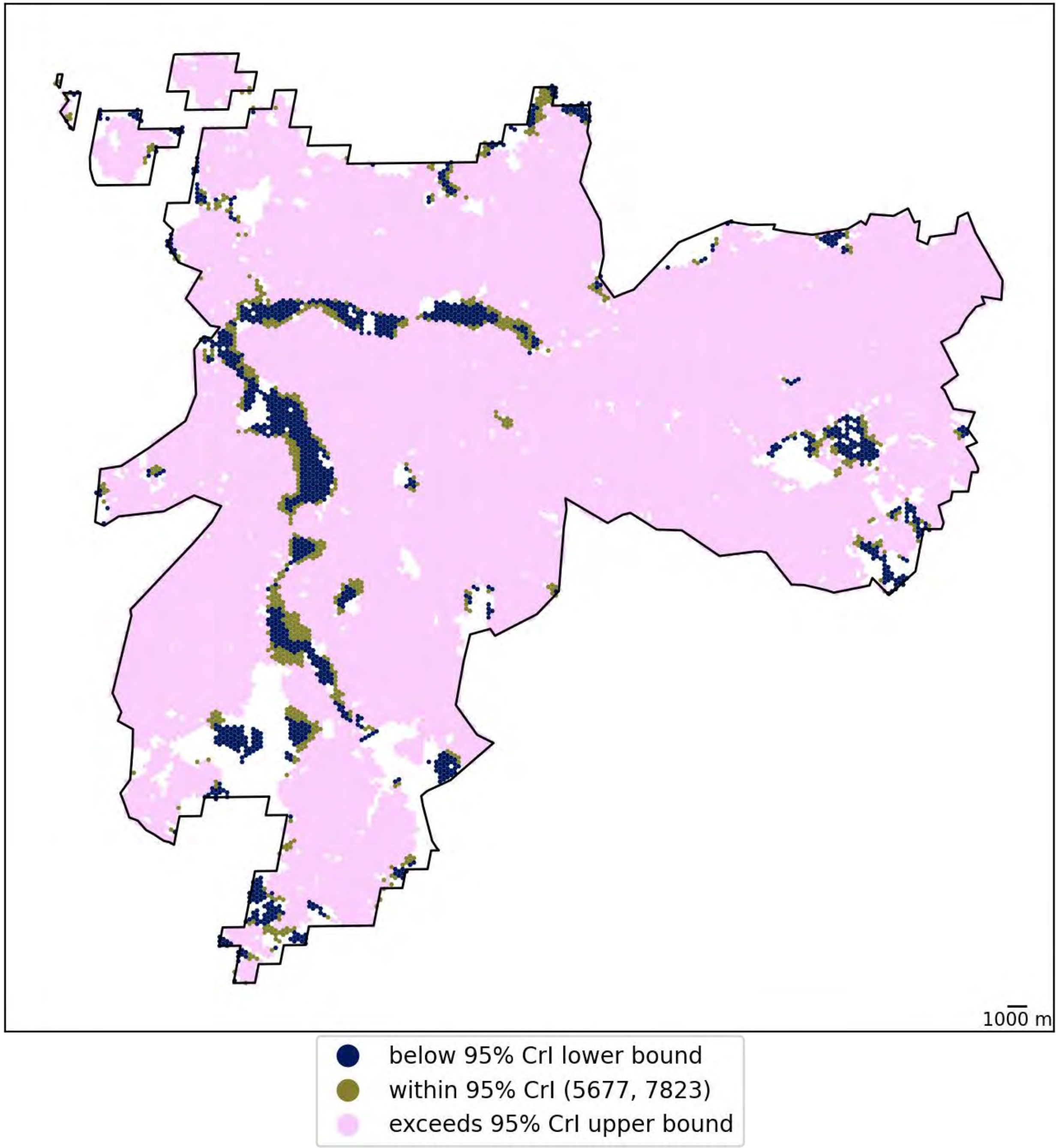

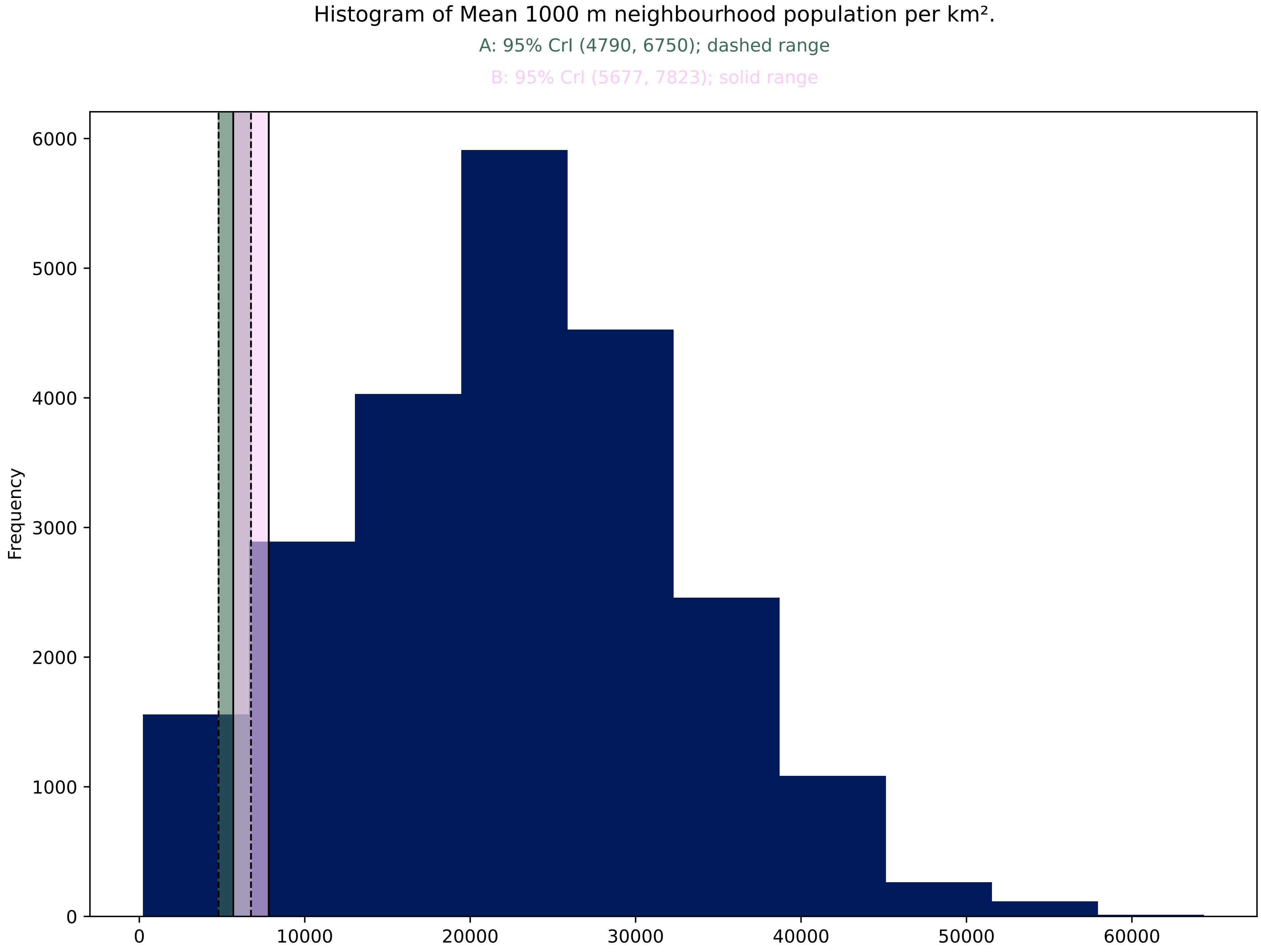
Histogram of Mean 1000 m neighbourhood population per km².
A: 95% CrI (4790, 6750); dashed range
B: 95% CrI (5677, 7823); solid range
Frequency
0
1000
2000
3000
4000
5000
6000
0
10000
20000
30000
40000
50000
60000

Mean 1000 m neighbourhood street intersections per km²

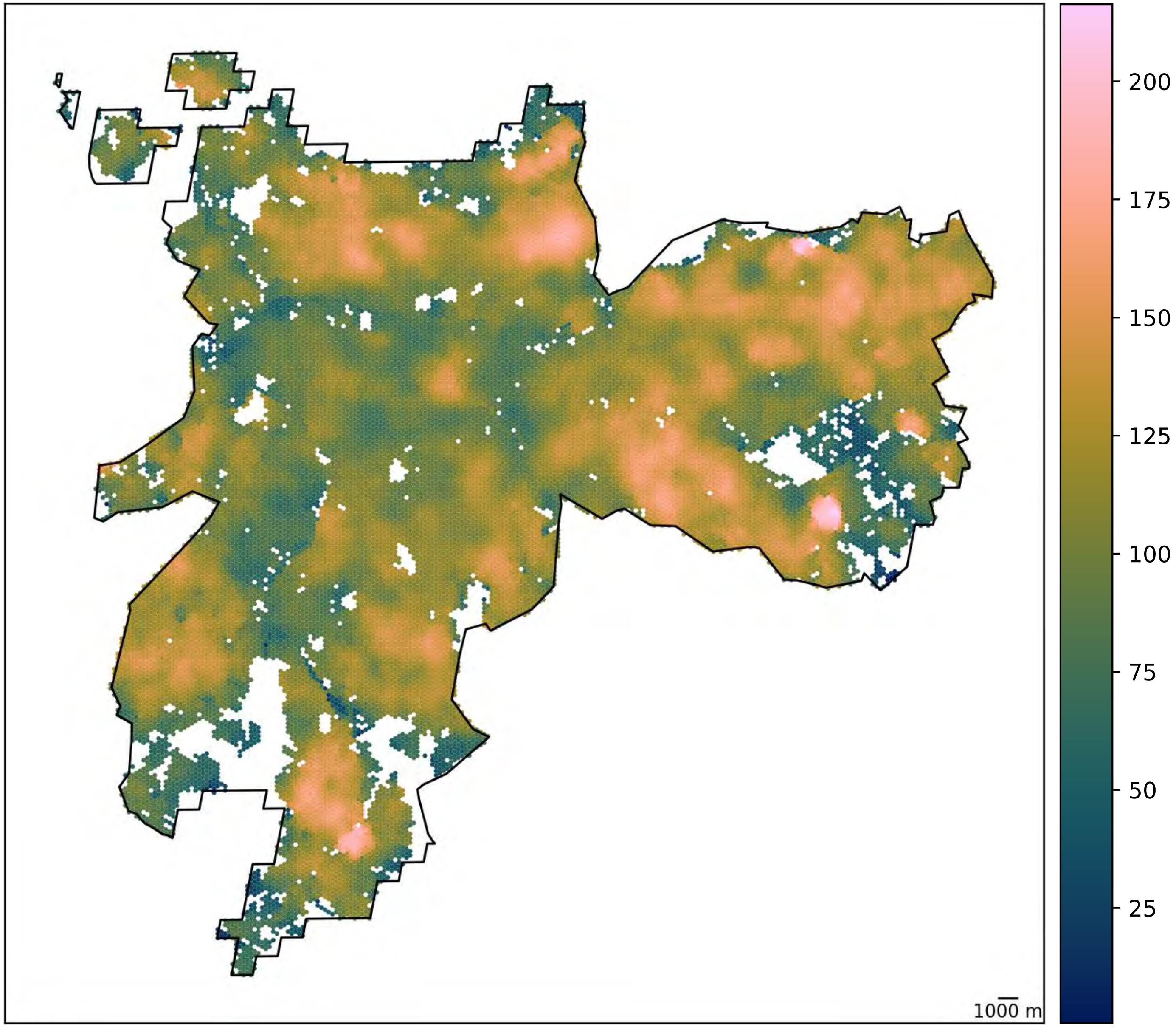

A: Estimated Mean 1000 m neighbourhood street intersections per km² requirement for ≥80% probability of engaging in walking for transport

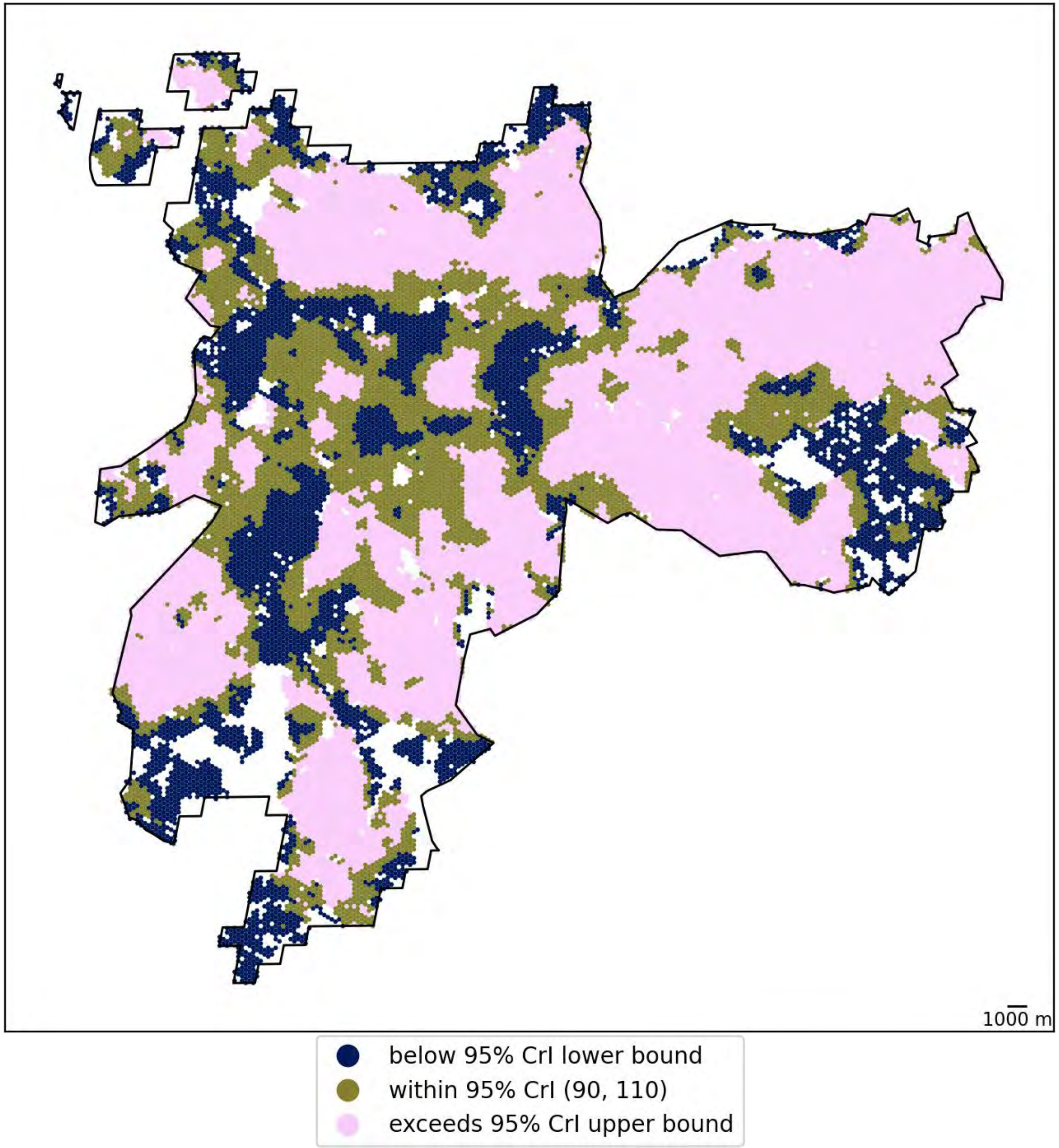

B: Estimated Mean 1000 m neighbourhood street intersections per km² requirement for reaching the WHO's target of a ≥15% relative reduction in insufficient physical activity through walking

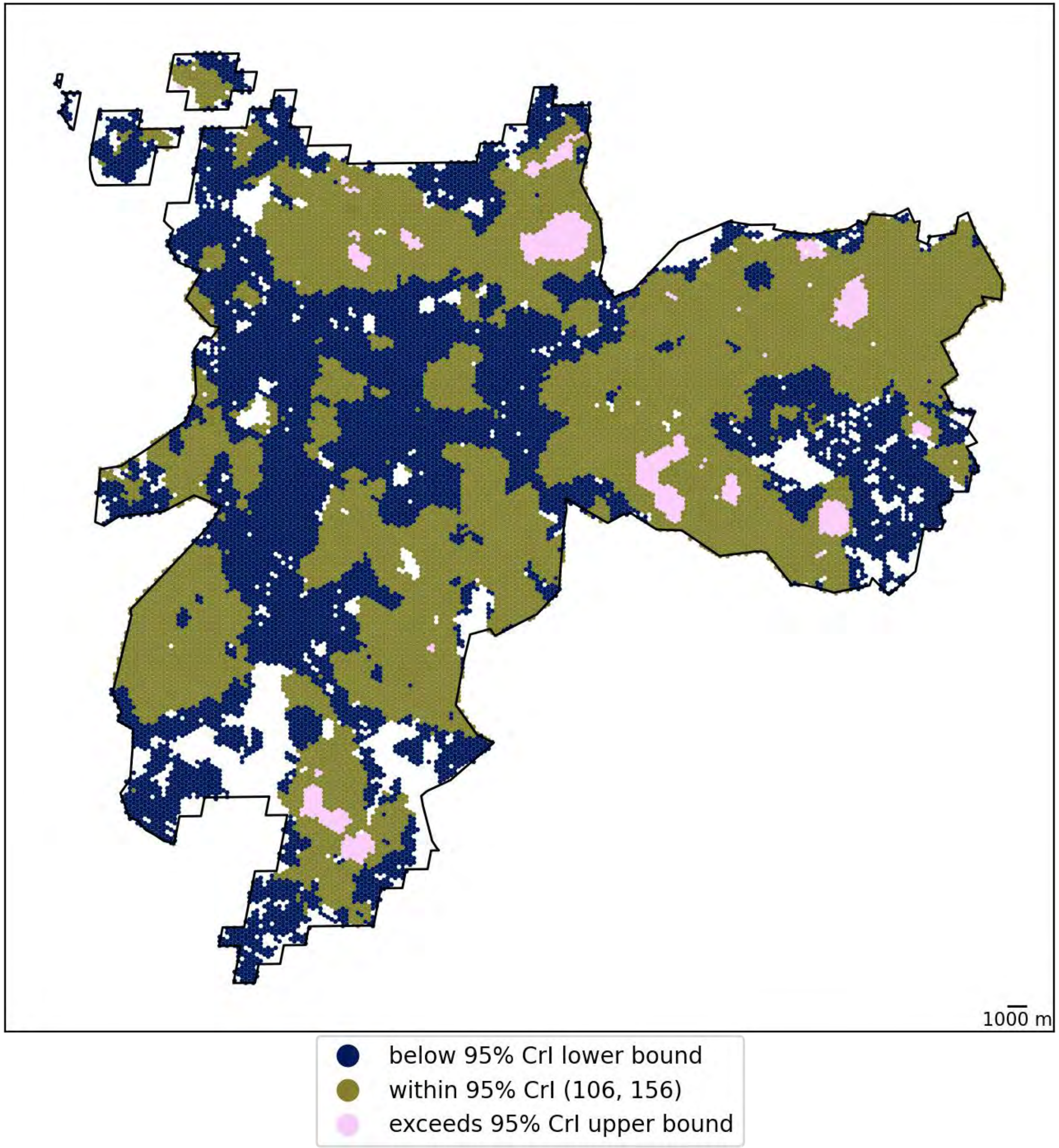

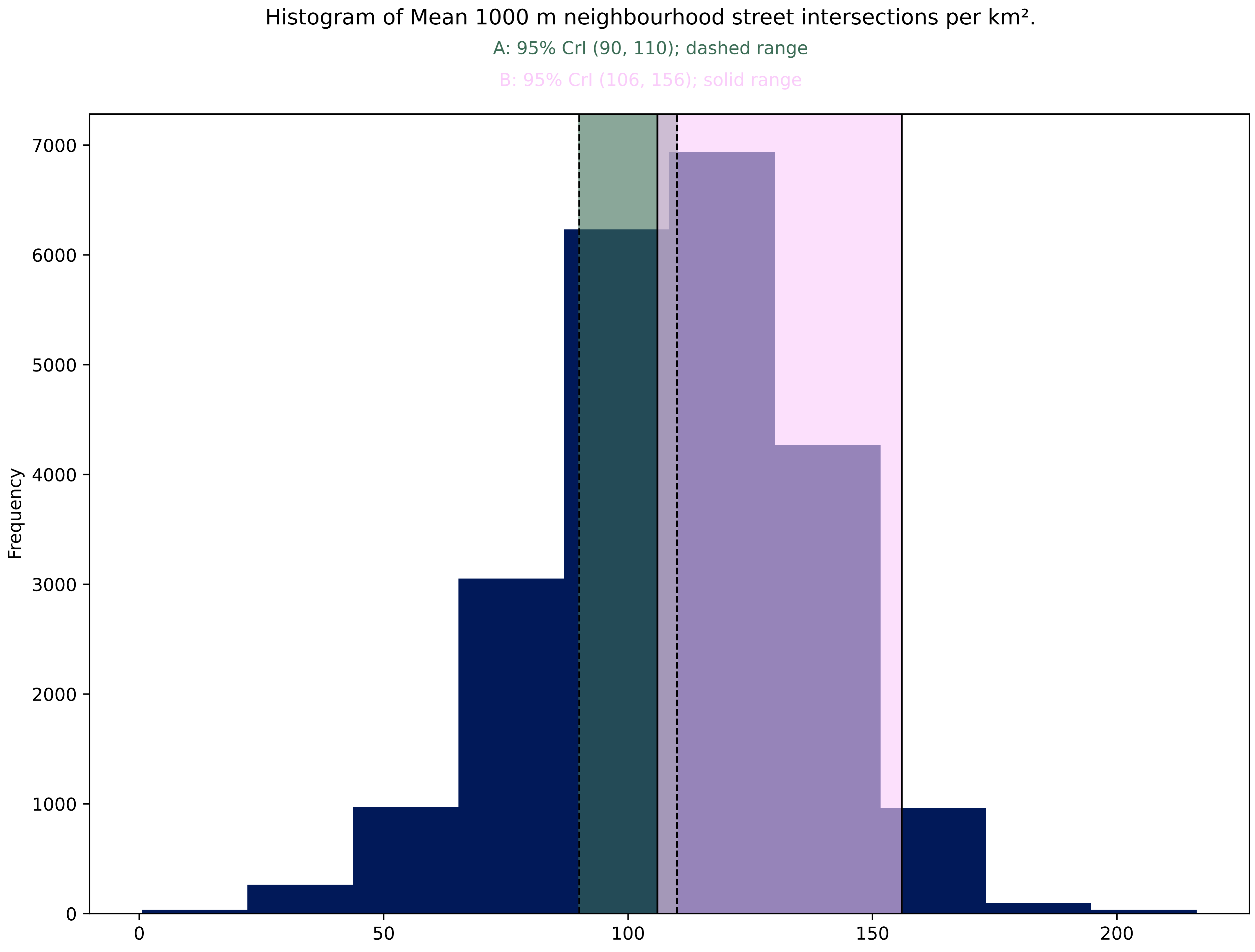
Histogram of Mean 1000 m neighbourhood street intersections per km².
A: 95% CrI (90, 110); dashed range
B: 95% CrI (106, 156); solid range
Frequency
0
1000
2000
3000
4000
5000
6000
7000
0
50
100
150
200

Distance to nearest public transport stops (m; up to 500m)

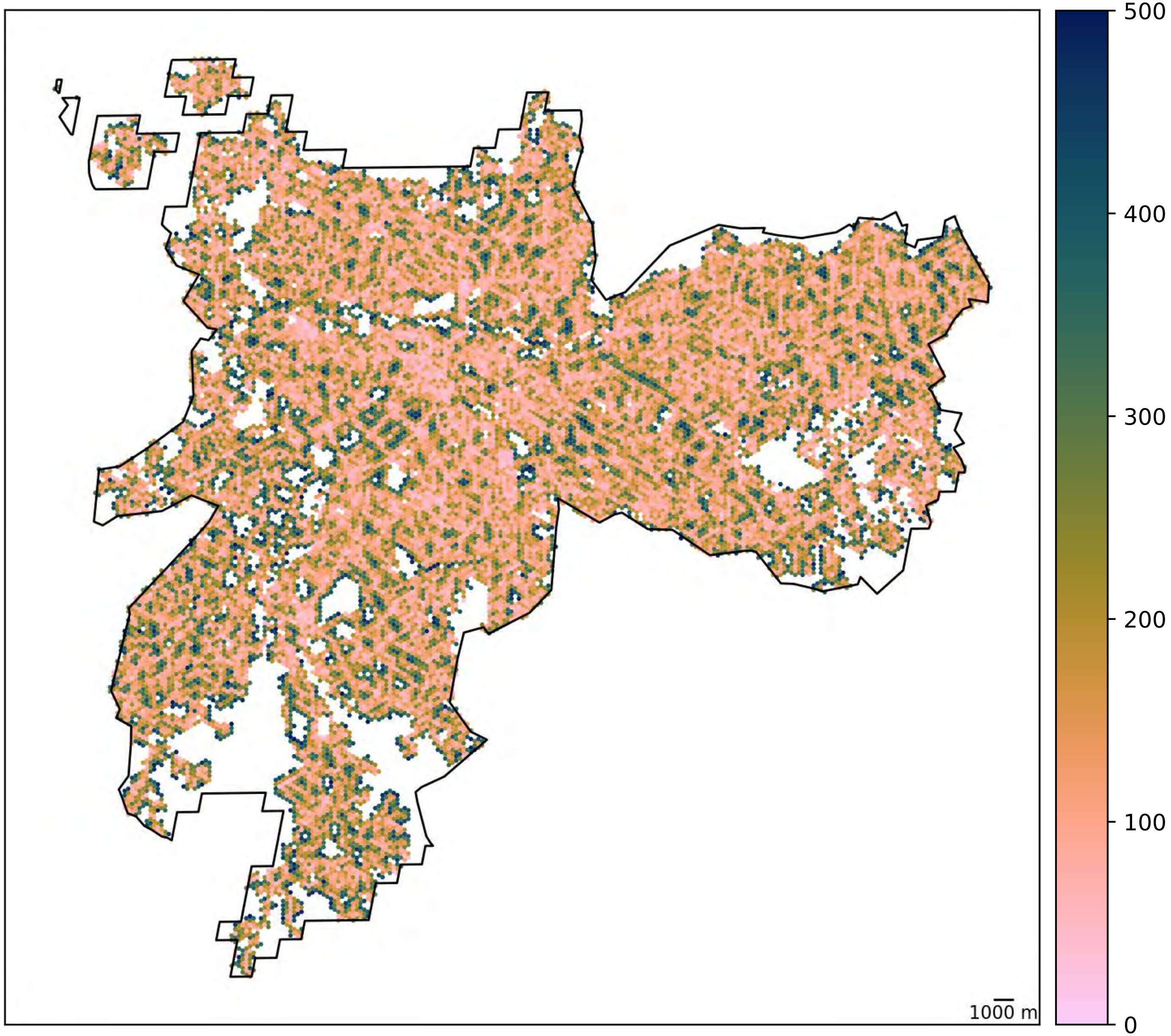

distances: Estimated Distance to nearest public transport stops (m; up to 500m) requirement for distances to destinations, measured up to a maximum distance target threshold of 500 metres

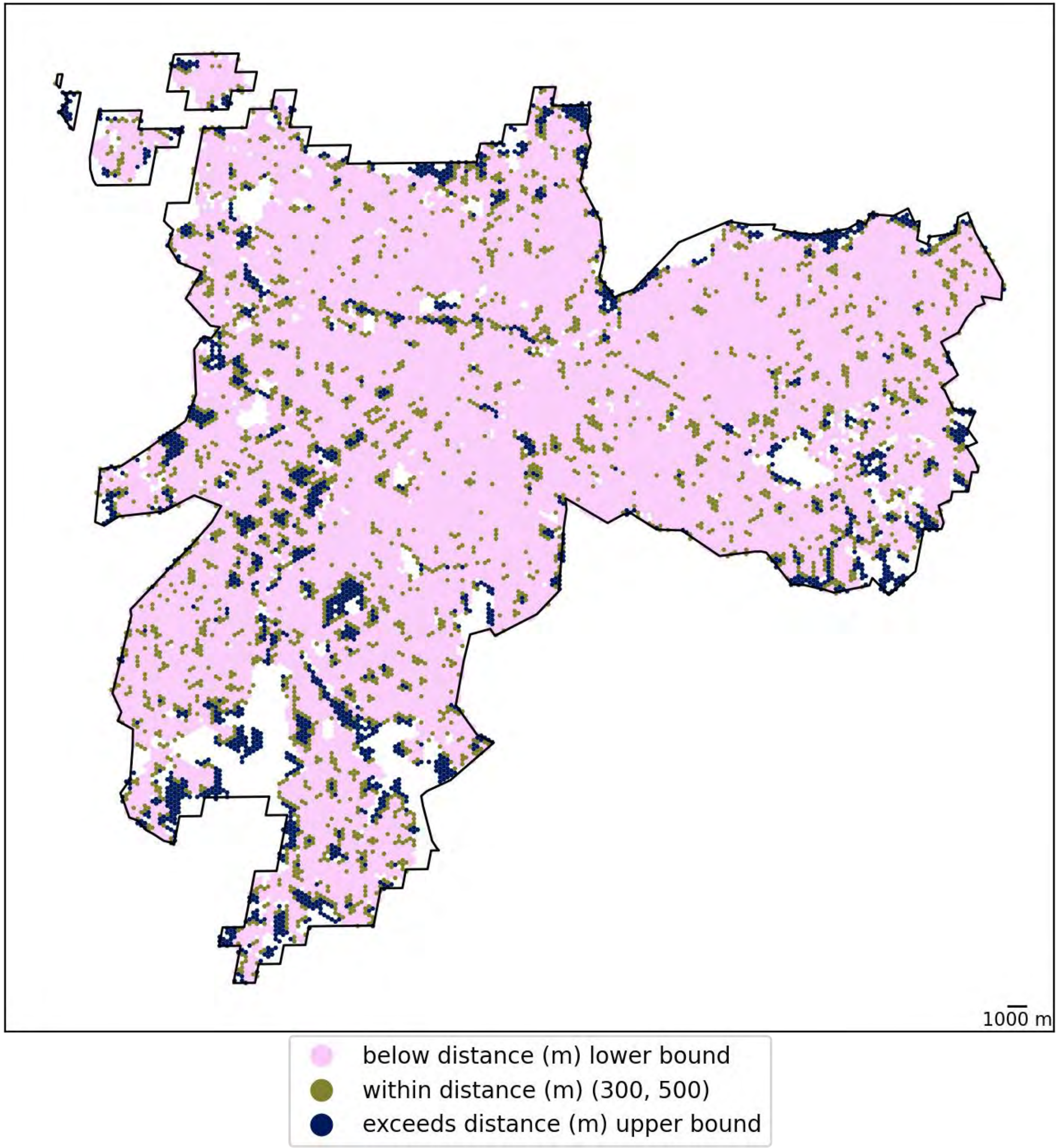

Histogram of Distance to nearest public transport stops (m; up to 500m).

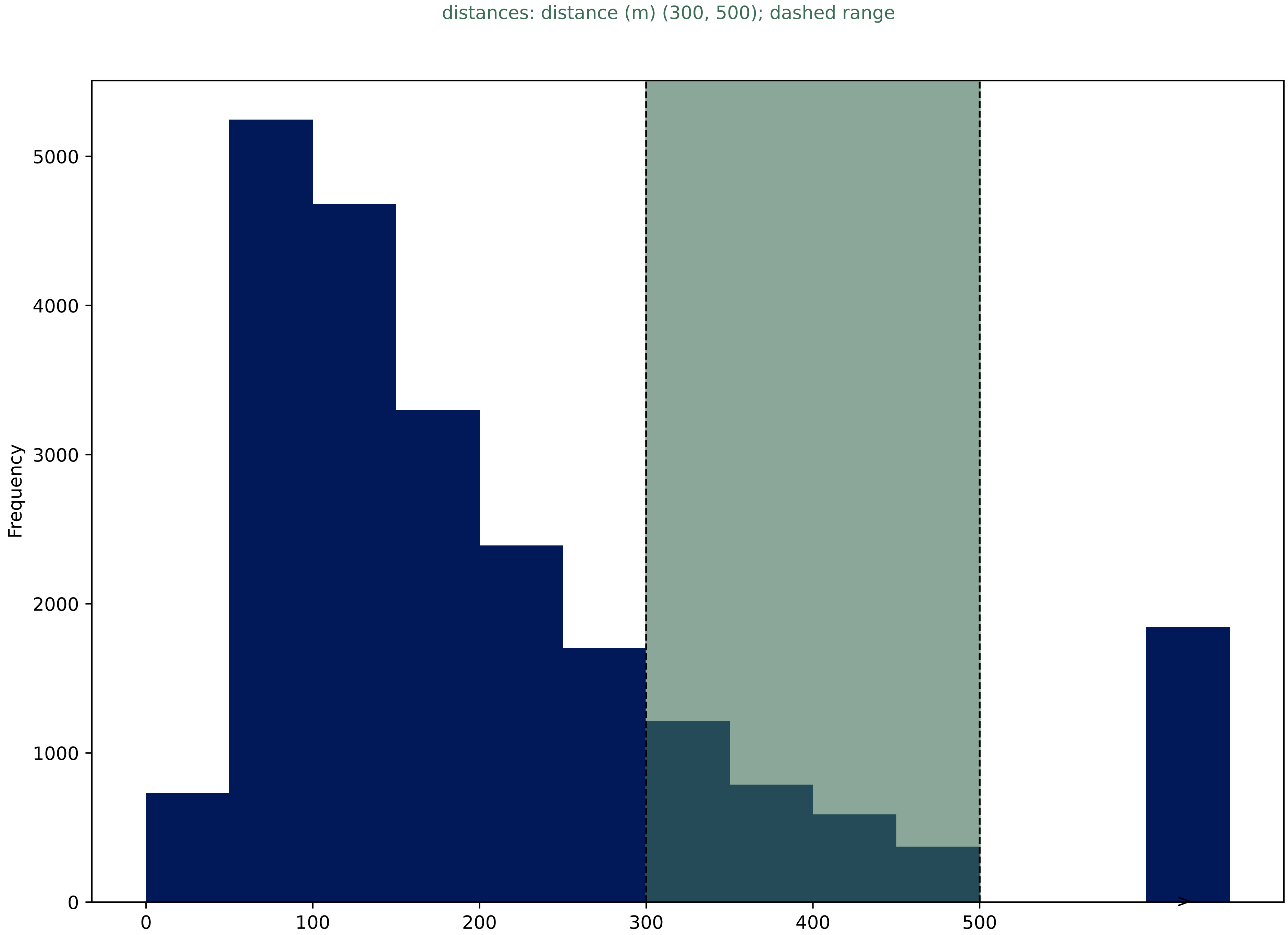

Distance to nearest park (m; up to 500m)

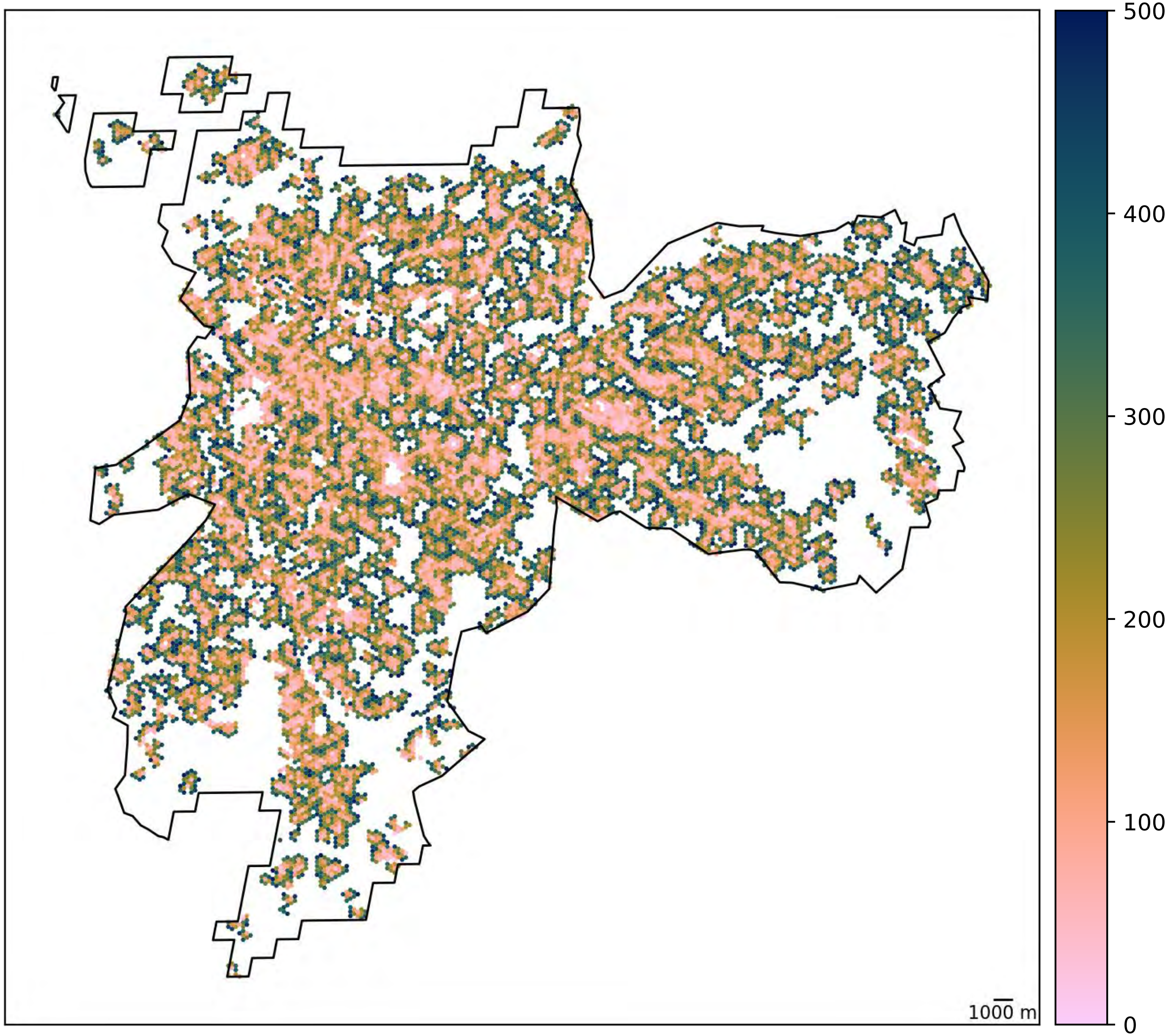

distances: Estimated Distance to nearest park (m; up to 500m) requirement for distances to destinations, measured up to a maximum distance target threshold of 500 metres

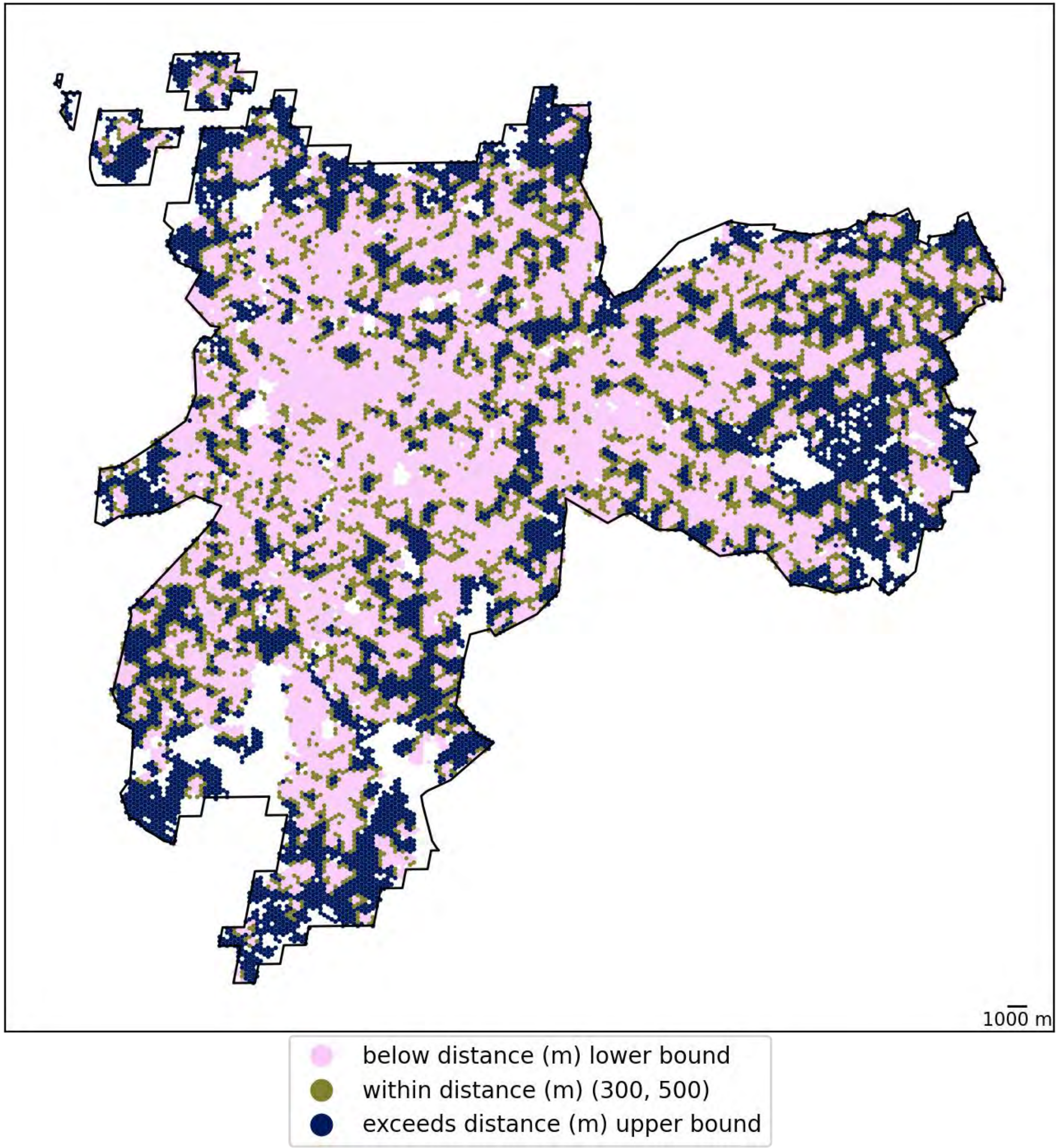

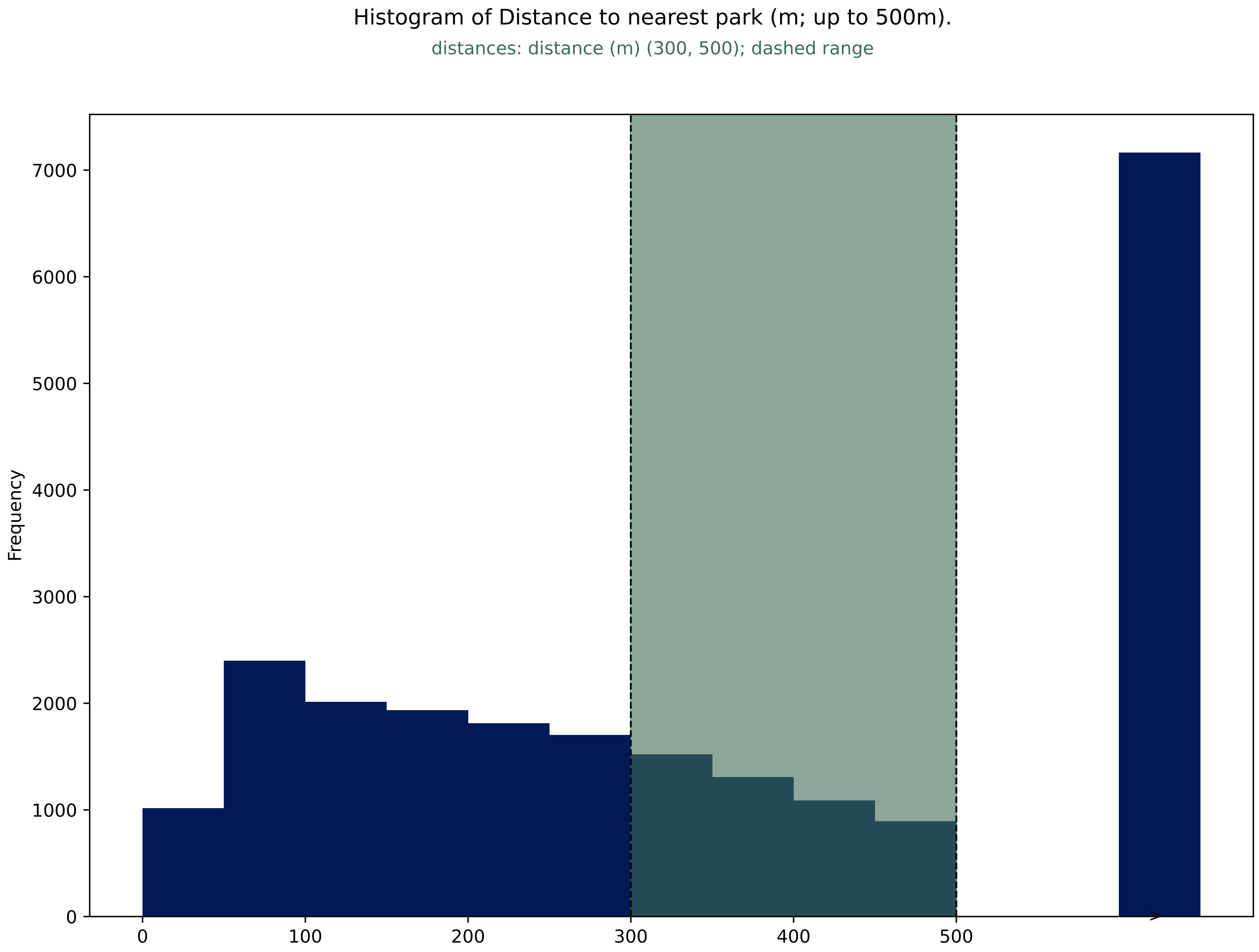

Histogram of Distance to nearest park (m; up to 500m).
distances: distance (m) (300, 500); dashed range
Frequency
0
1000
2000
3000
4000
5000
6000
7000
0
100
200
300
400
500
>

Asia, China (SAR), Hong Kong

Satellite imagery of urban study region (Bing)

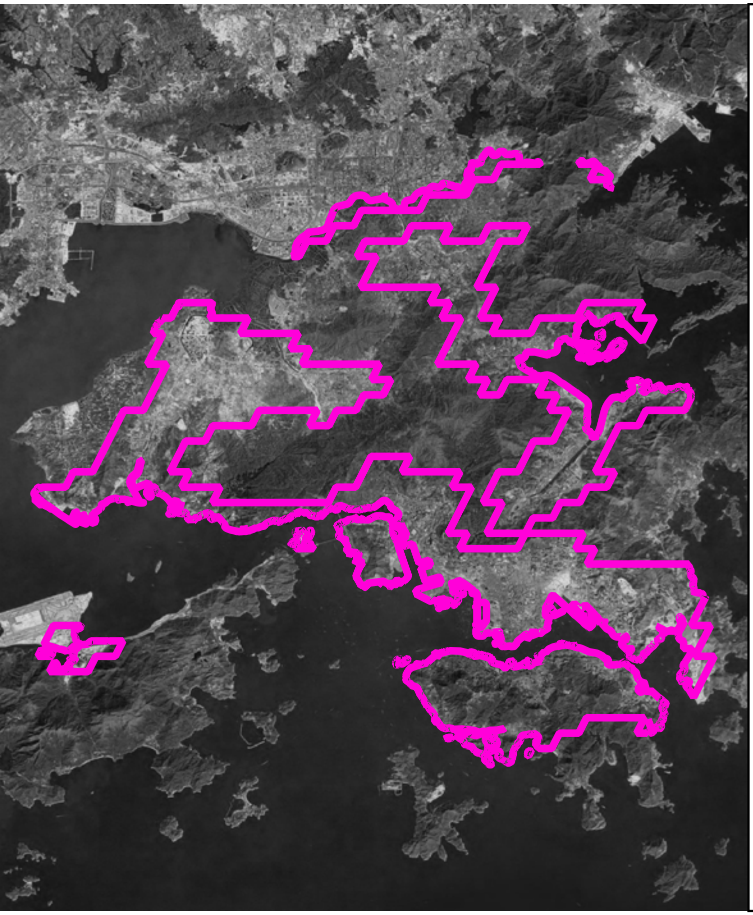

Walkability, relative to city

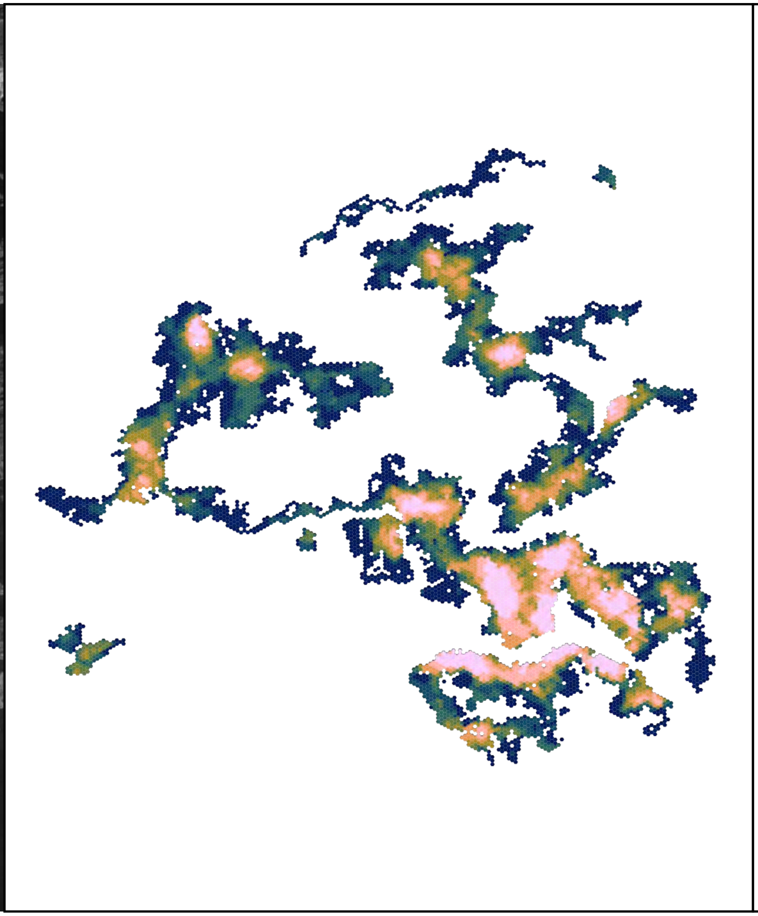

Walkability, relative to 25 global cities

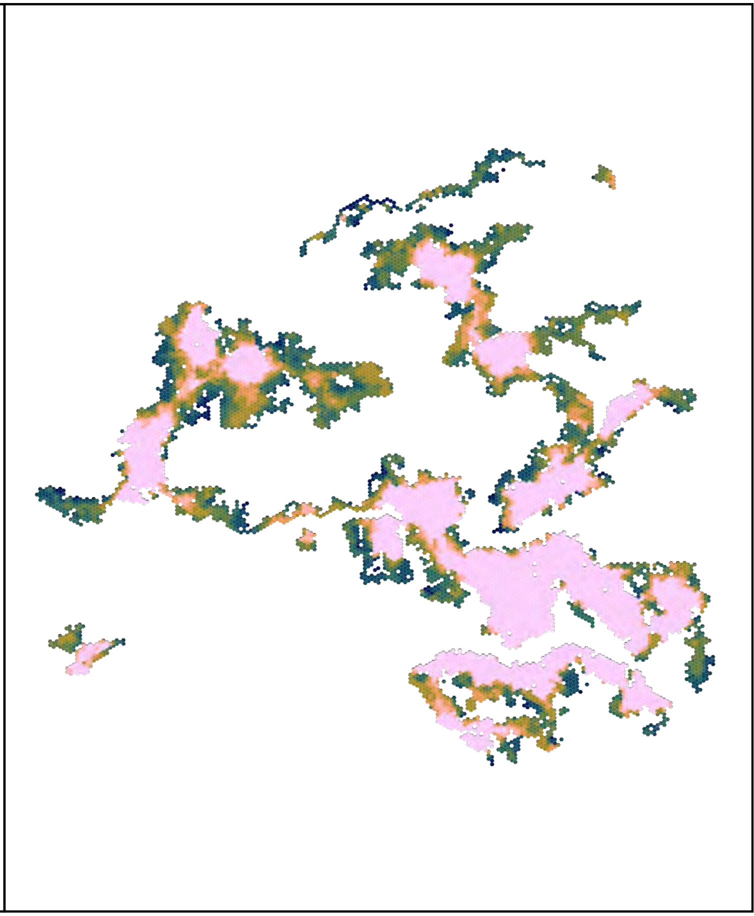

Urban boundary

0 19 38 km

Walkability score

- <-3
- -3 to -2
- -2 to -1
- -1 to 0
- 0 to 1
- 1 to 2
- 2 to 3
- ≥3

Walkability relative to all cities by component variables (2D histograms), and overall (histogram)

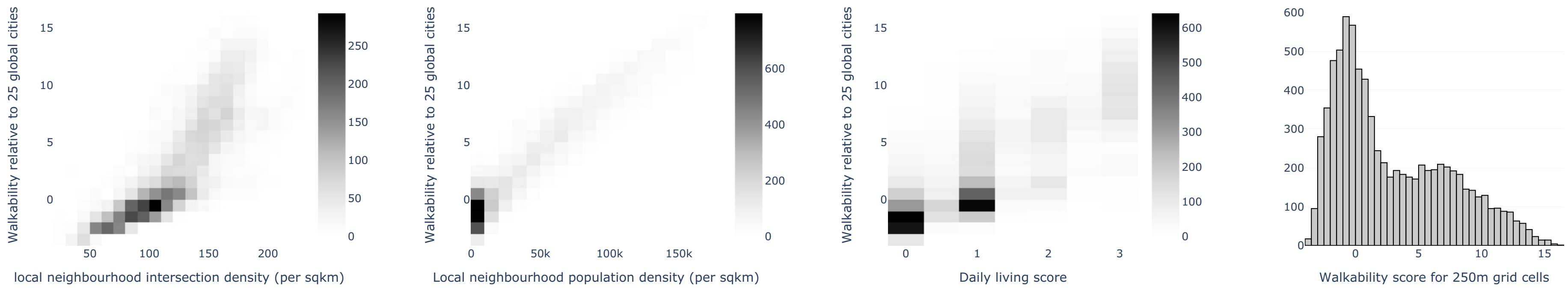

Mean 1000 m neighbourhood population per km²

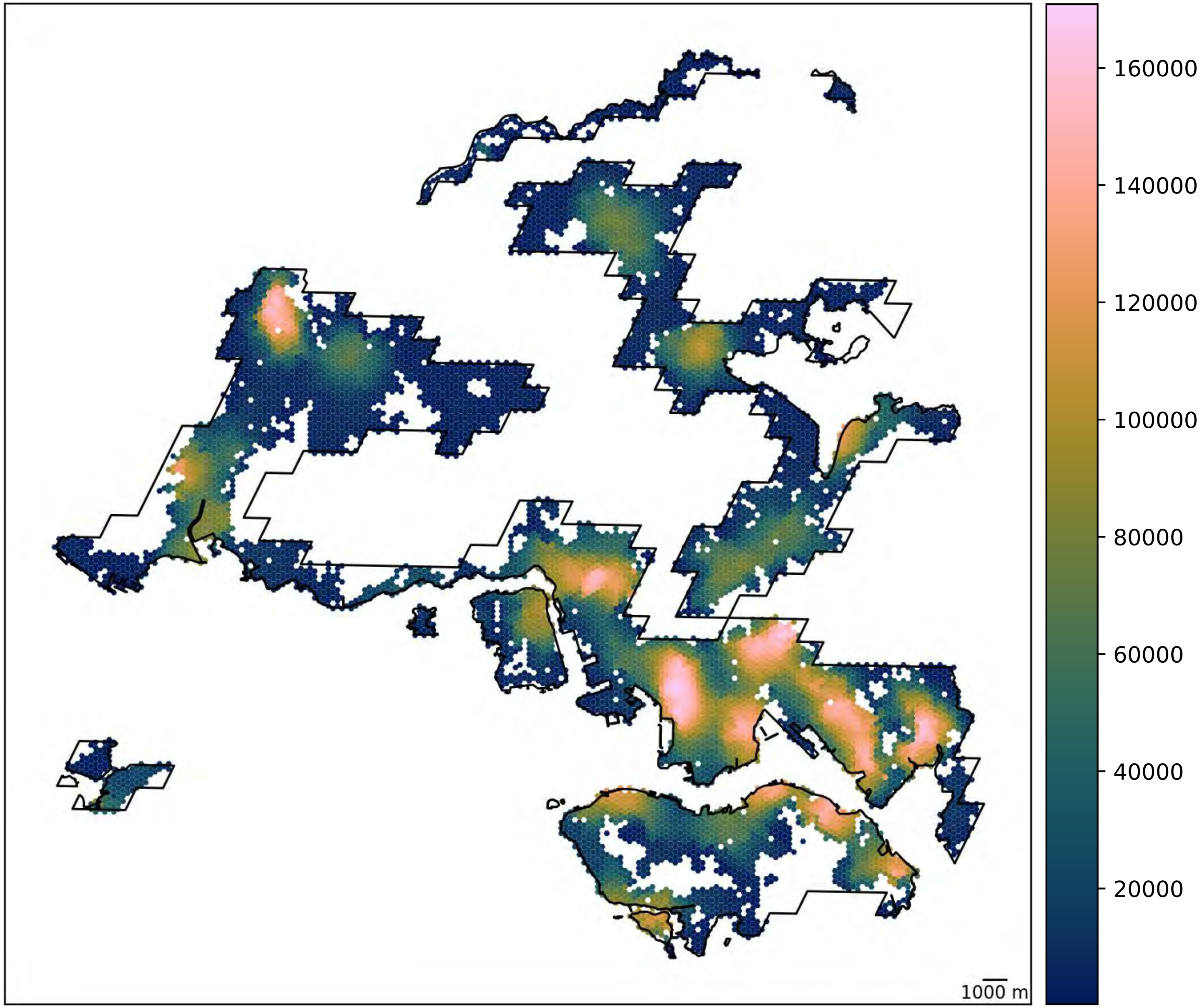

A: Estimated Mean 1000 m neighbourhood population per km² requirement for ≥80% probability of engaging in walking for transport

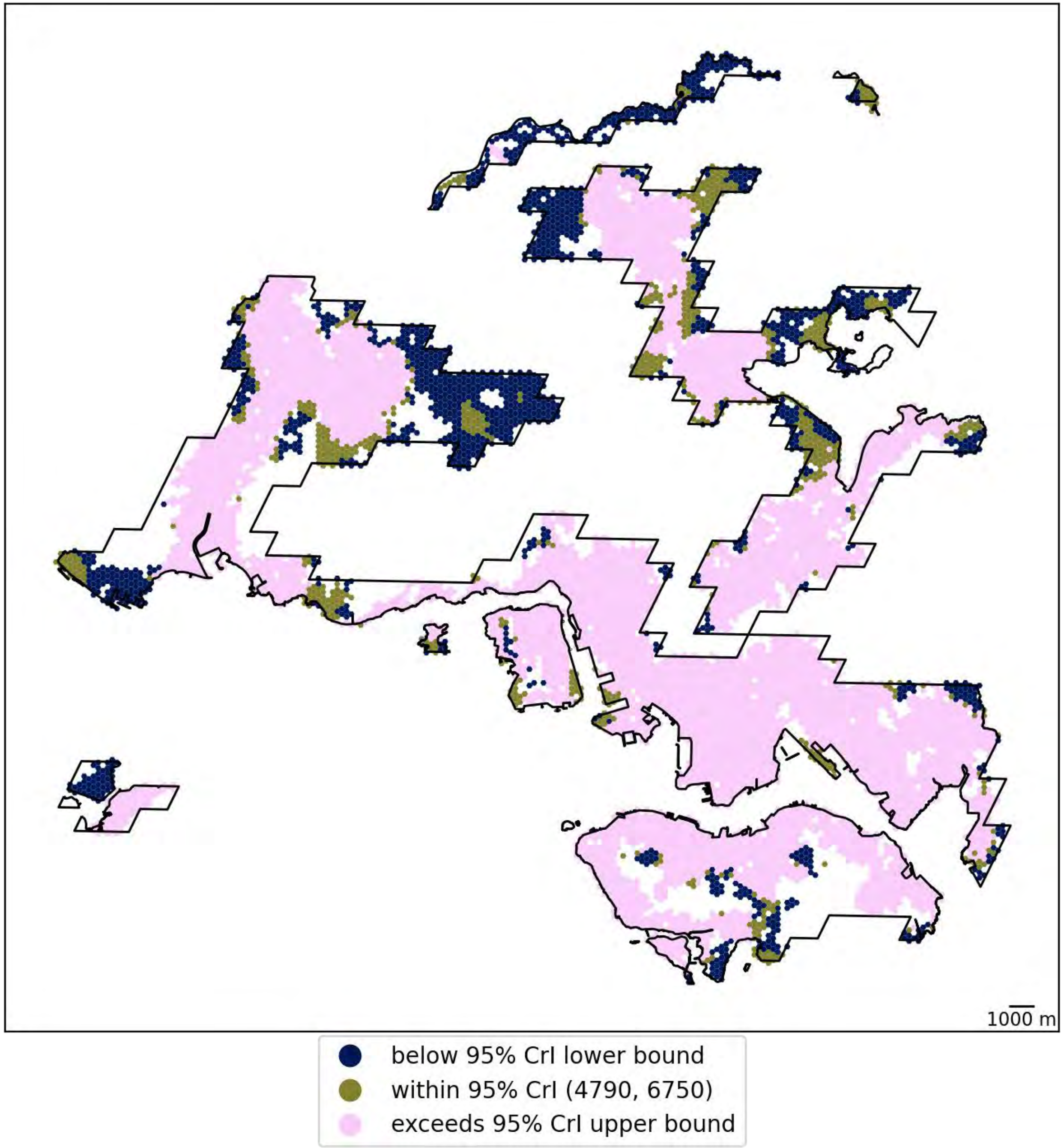

B: Estimated Mean 1000 m neighbourhood population per km² requirement for reaching the WHO's target of a ≥15% relative reduction in insufficient physical activity through walking

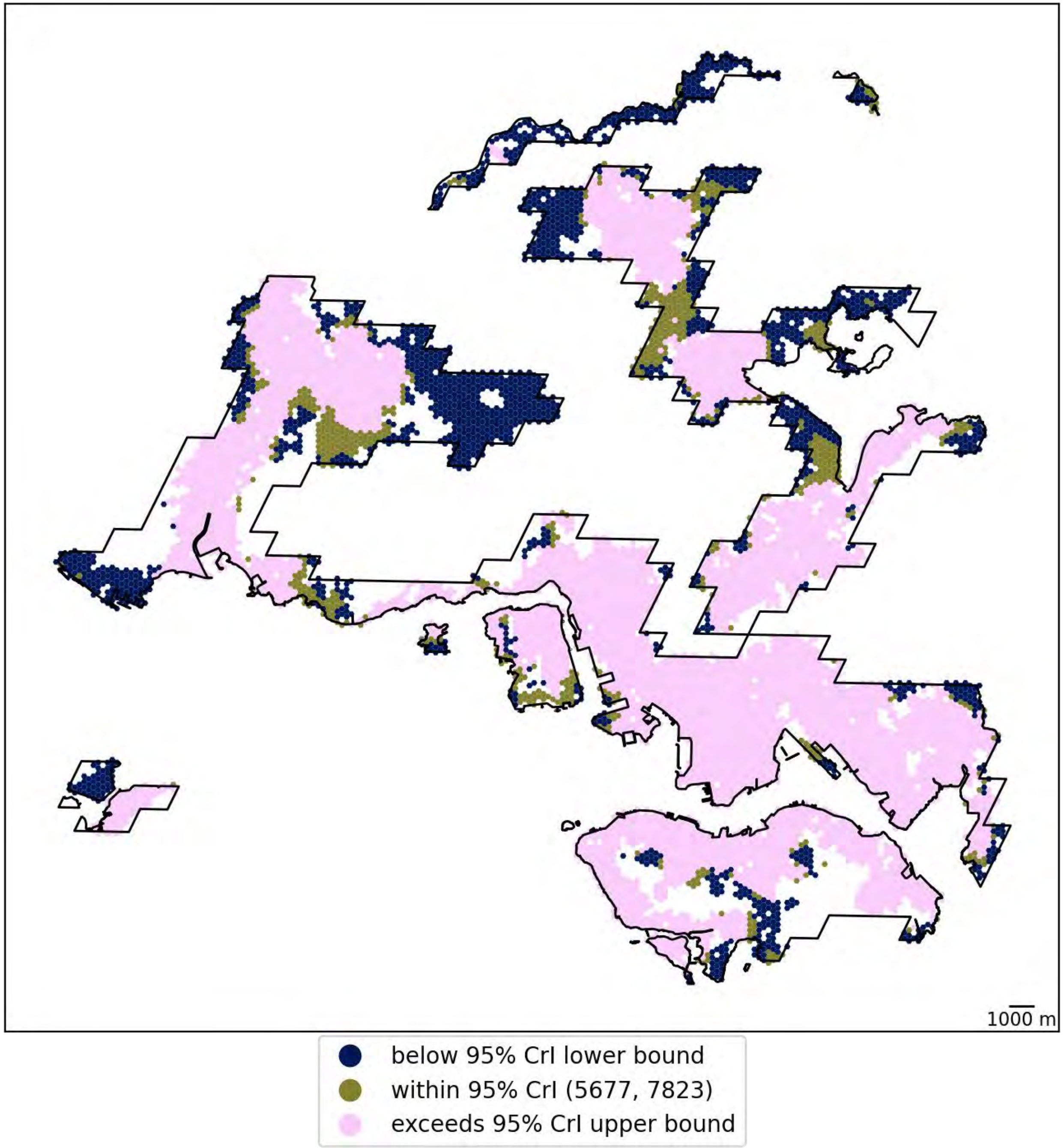

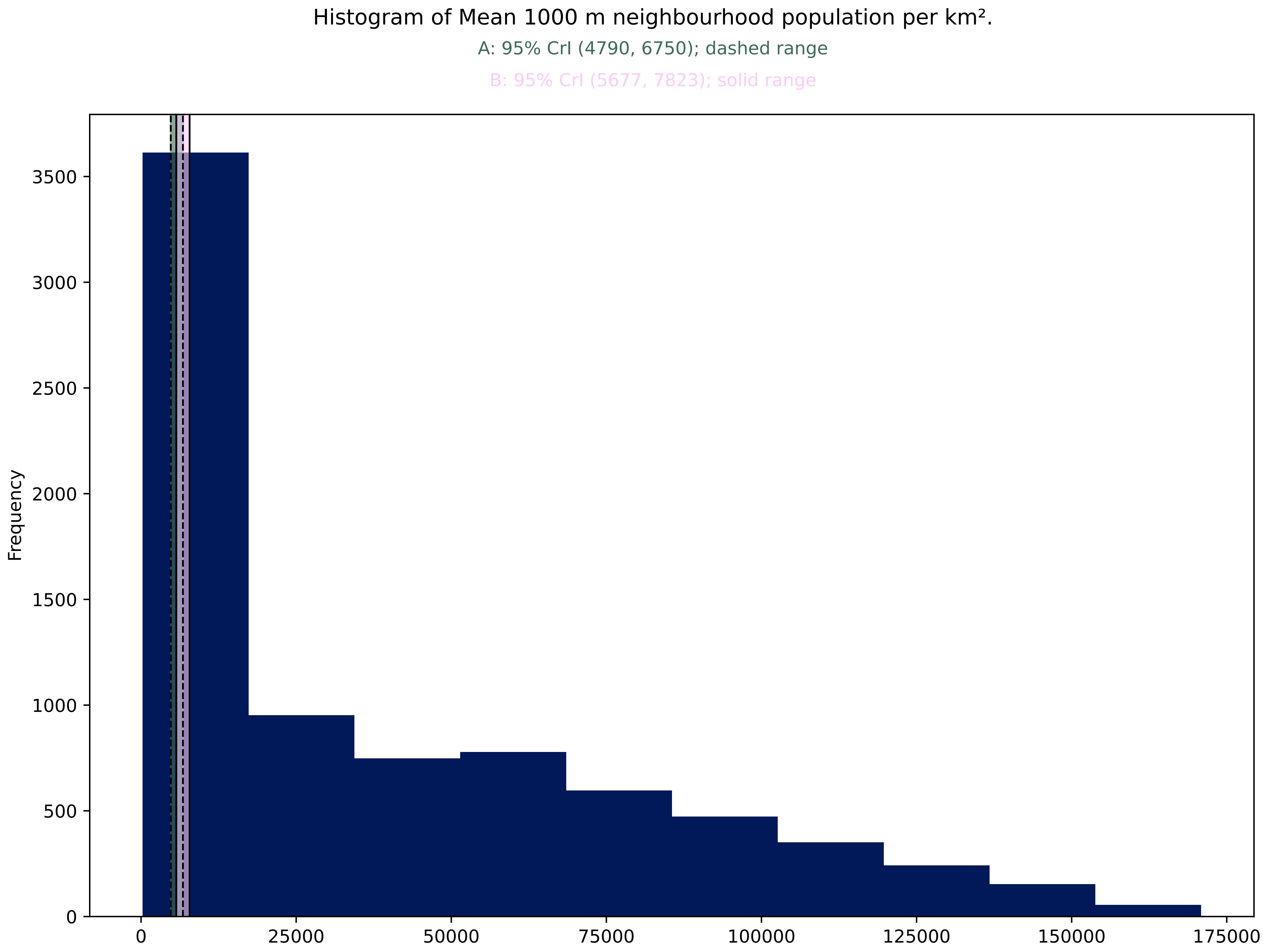
Histogram of Mean 1000 m neighbourhood population per km².
A: 95% CrI (4790, 6750); dashed range
B: 95% CrI (5677, 7823); solid range
Frequency
0
500
1000
1500
2000
2500
3000
3500
0
25000
50000
75000
100000
125000
150000
175000

Mean 1000 m neighbourhood street intersections per km²

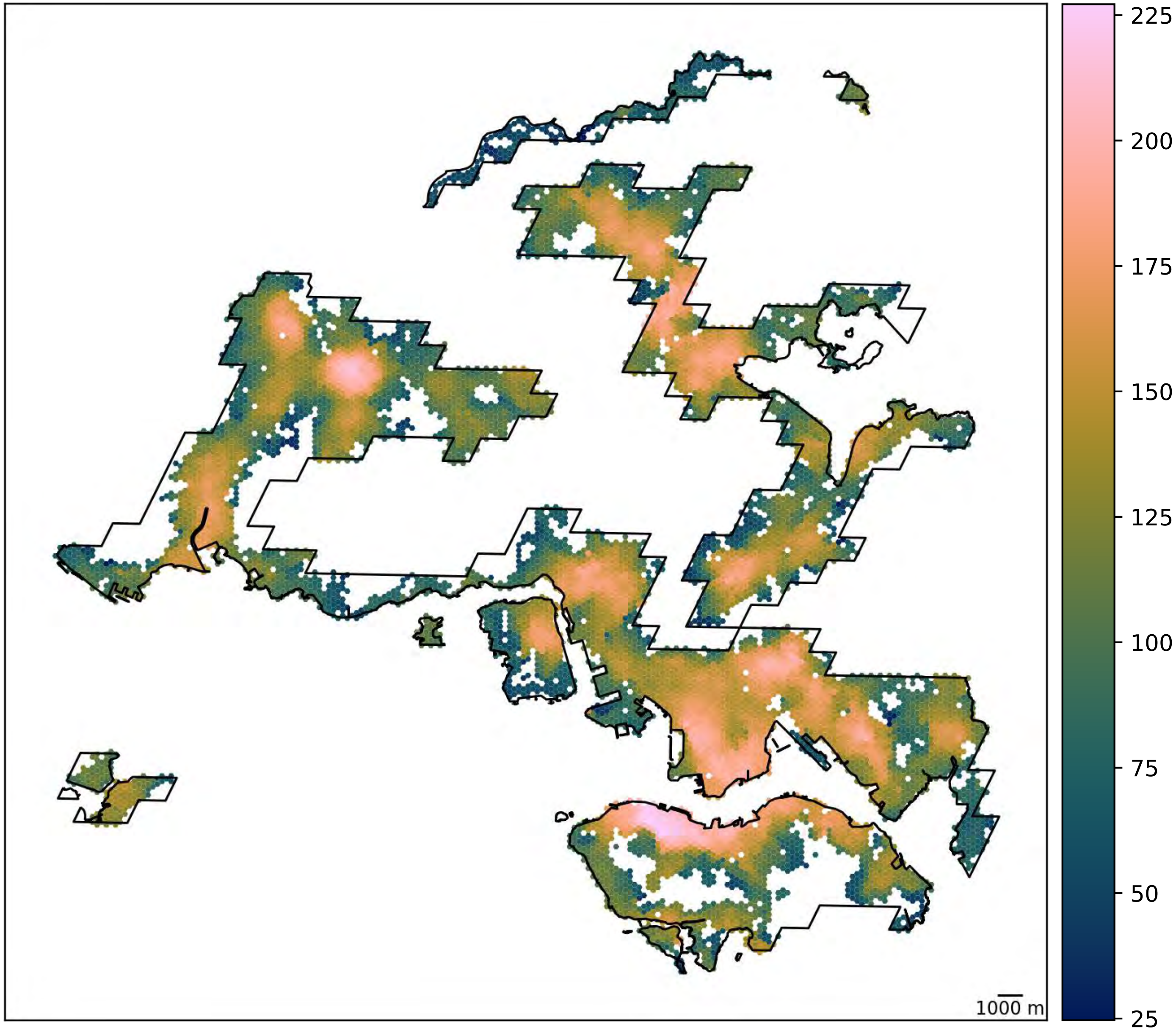

A: Estimated Mean 1000 m neighbourhood street intersections per km² requirement for ≥80% probability of engaging in walking for transport

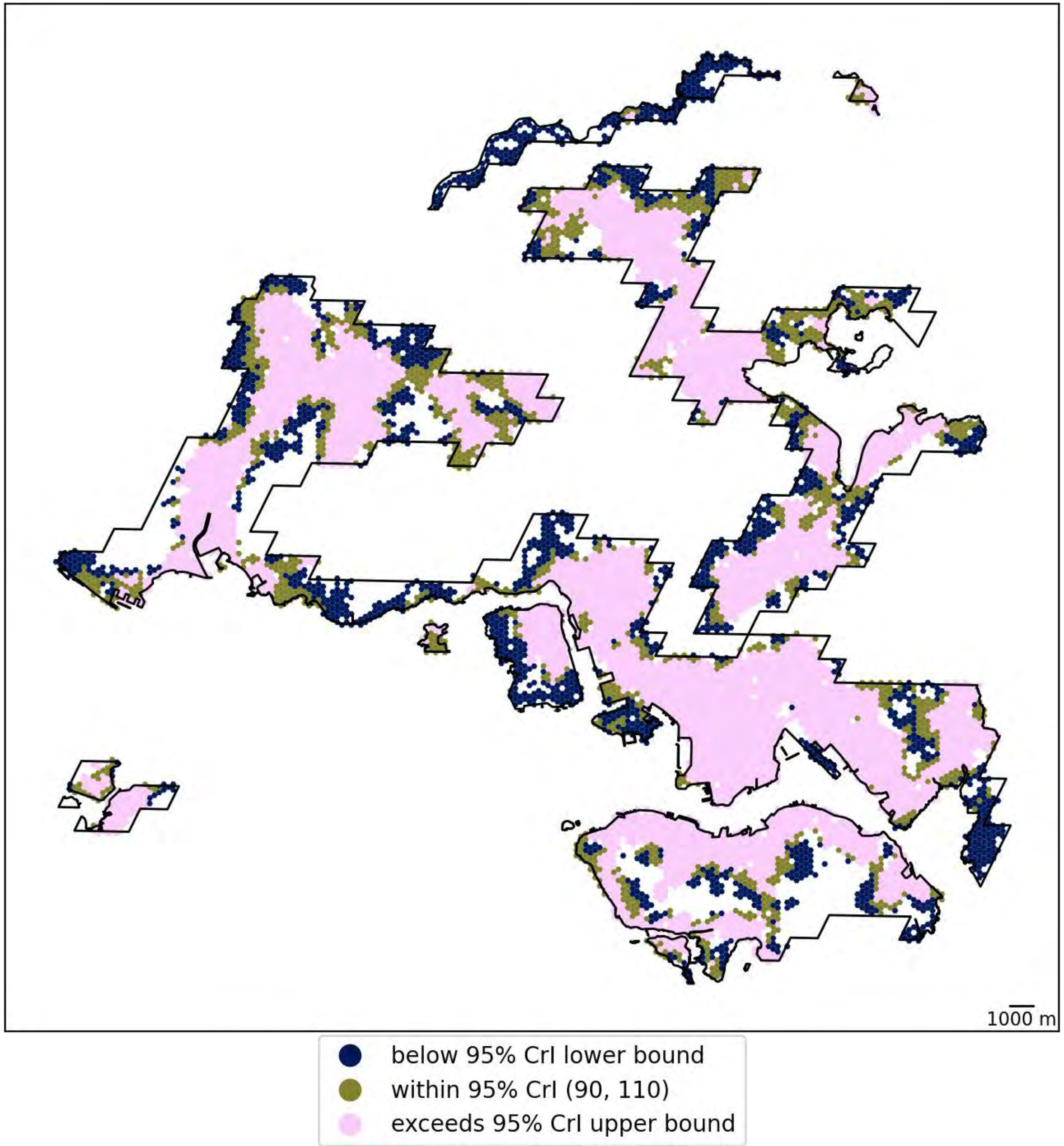

B: Estimated Mean 1000 m neighbourhood street intersections per km² requirement for reaching the WHO's target of a ≥15% relative reduction in insufficient physical activity through walking

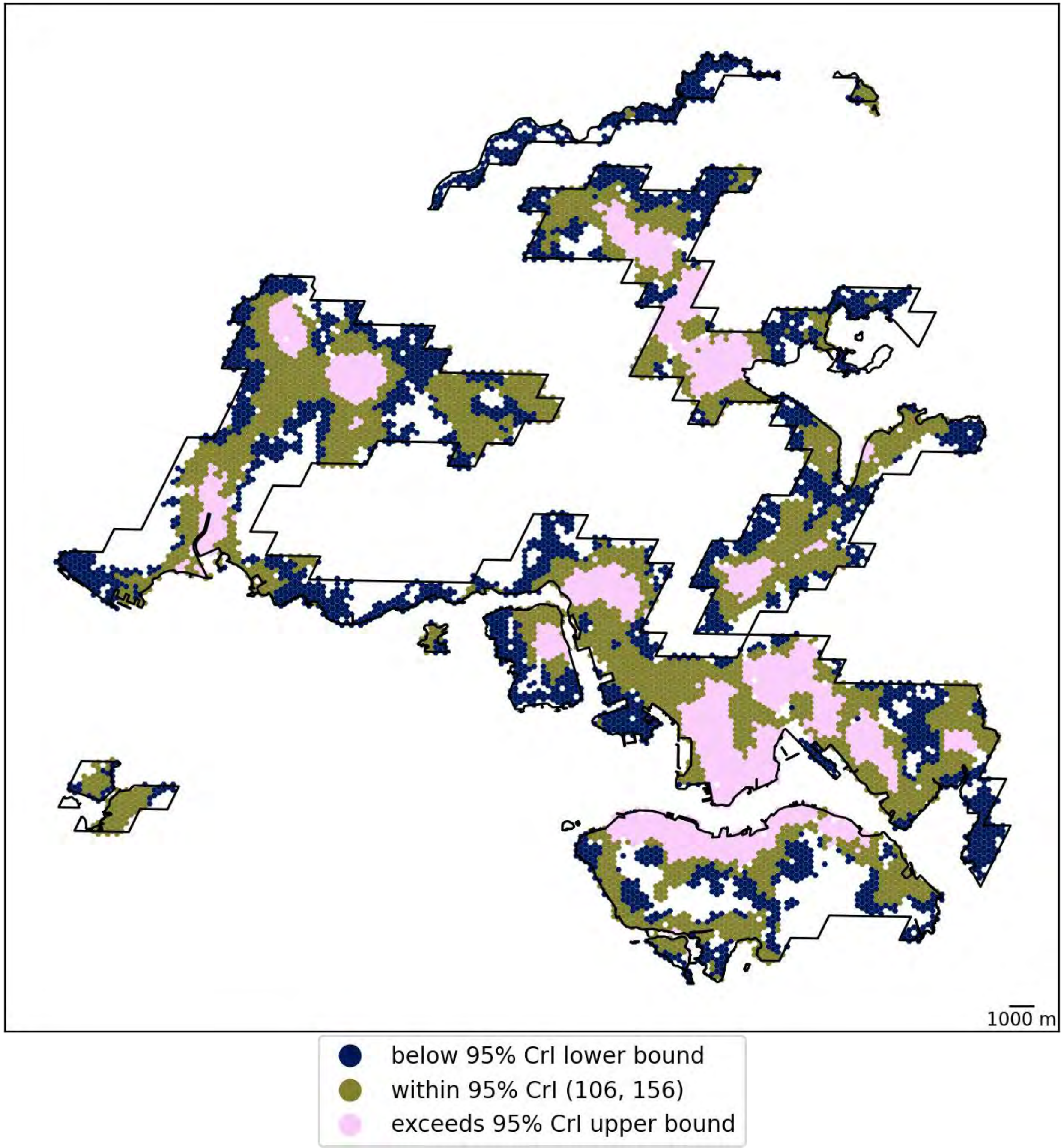

Histogram of Mean 1000 m neighbourhood street intersections per km².

A: 95% CrI (90, 110); dashed range

B: 95% CrI (106, 156); solid range

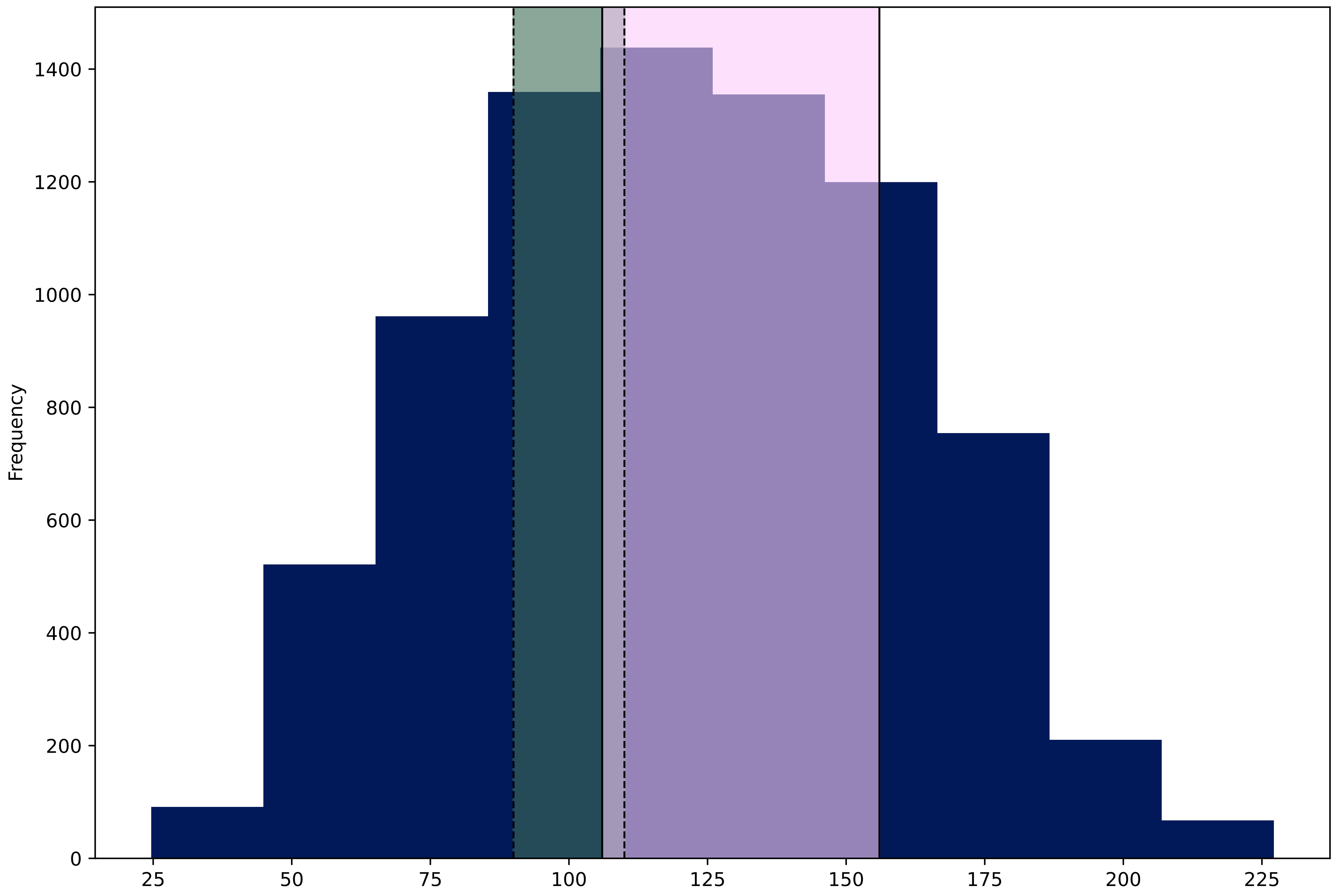

Asia, India, Chennai

Satellite imagery of urban study region (Bing) | Walkability, relative to city | Walkability, relative to 25 global cities

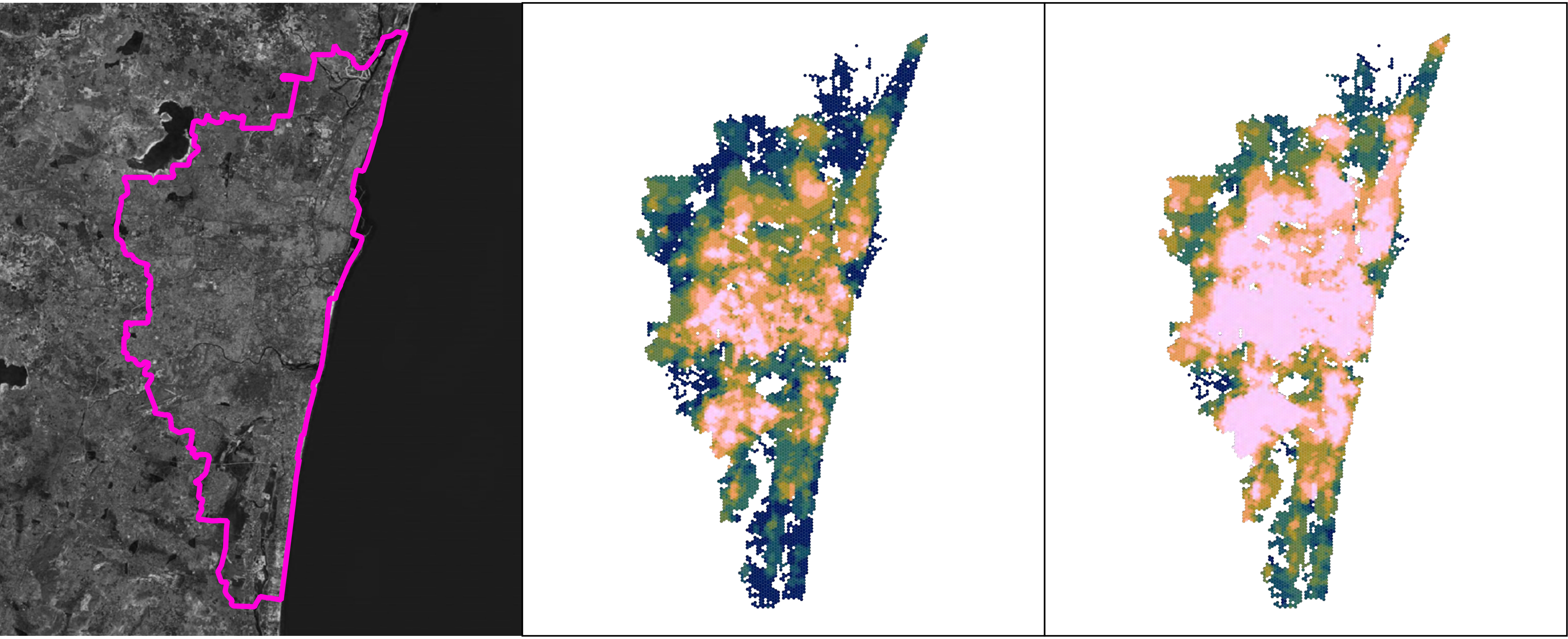

Urban boundary

0 10 20 km

Walkability score
- <-3
- -3 to -2
- -2 to -1
- -1 to 0
- 0 to 1
- 1 to 2
- 2 to 3
- ≥3

Walkability relative to all cities by component variables (2D histograms), and overall (histogram)

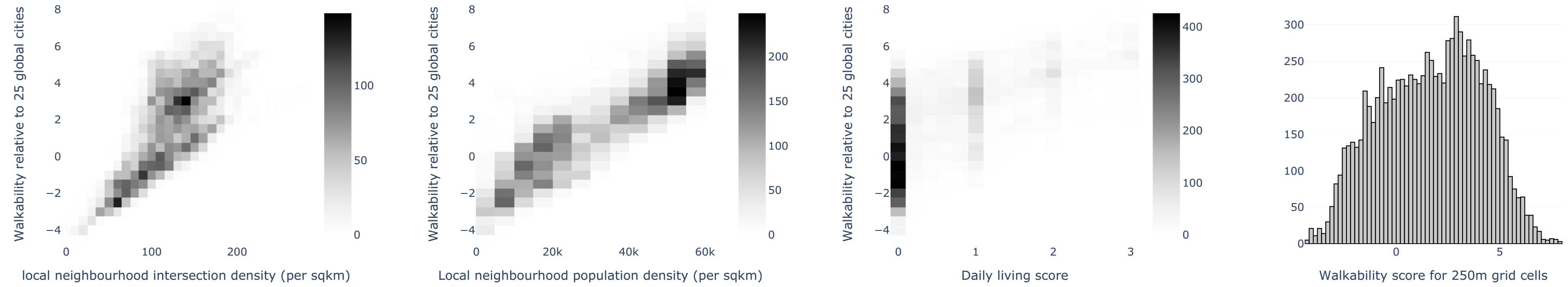

Distance to nearest public transport stops (m; up to 500m)

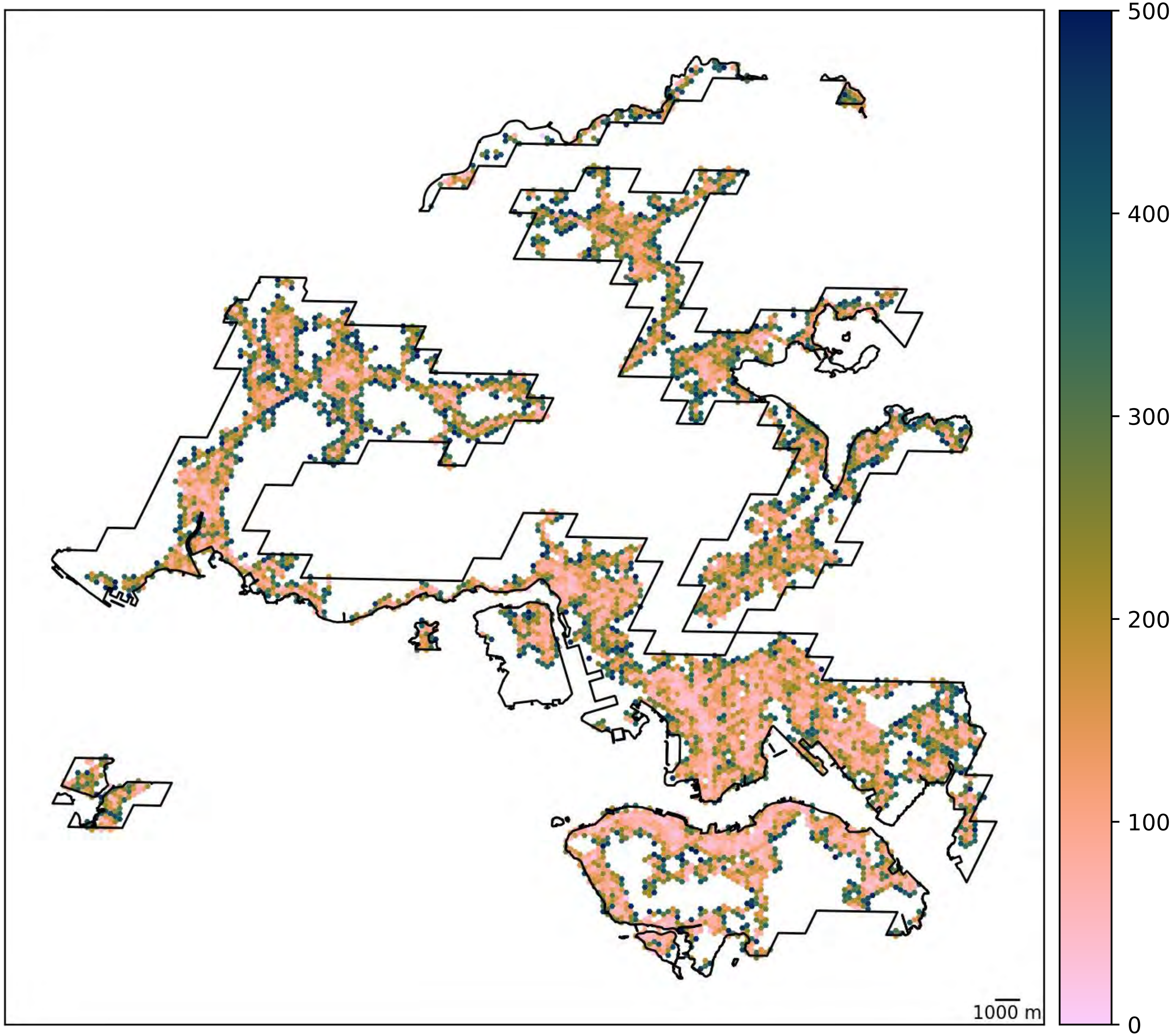

distances: Estimated Distance to nearest public transport stops (m; up to 500m) requirement for distances to destinations, measured up to a maximum distance target threshold of 500 metres

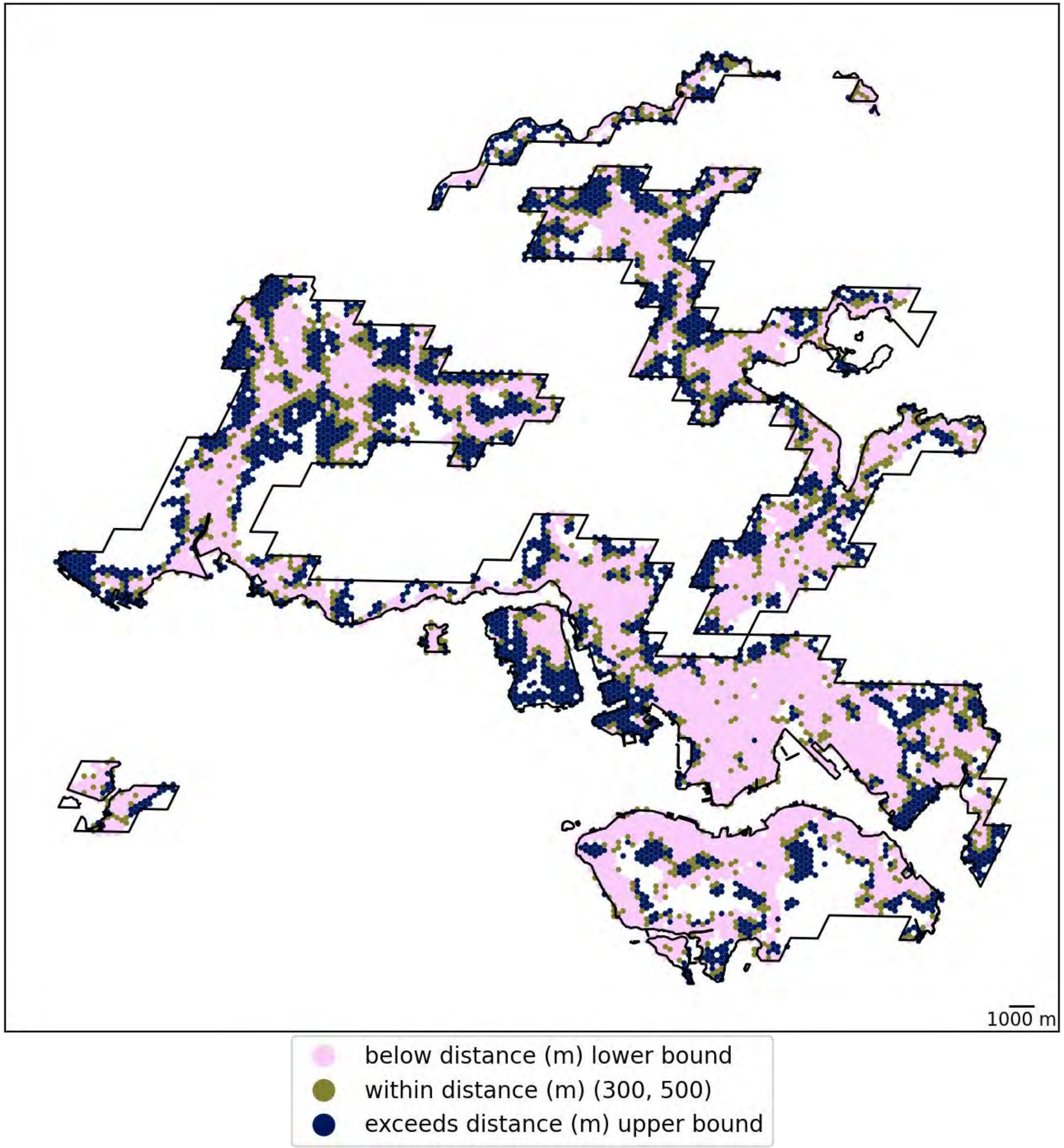

Histogram of Distance to nearest public transport stops (m; up to 500m).

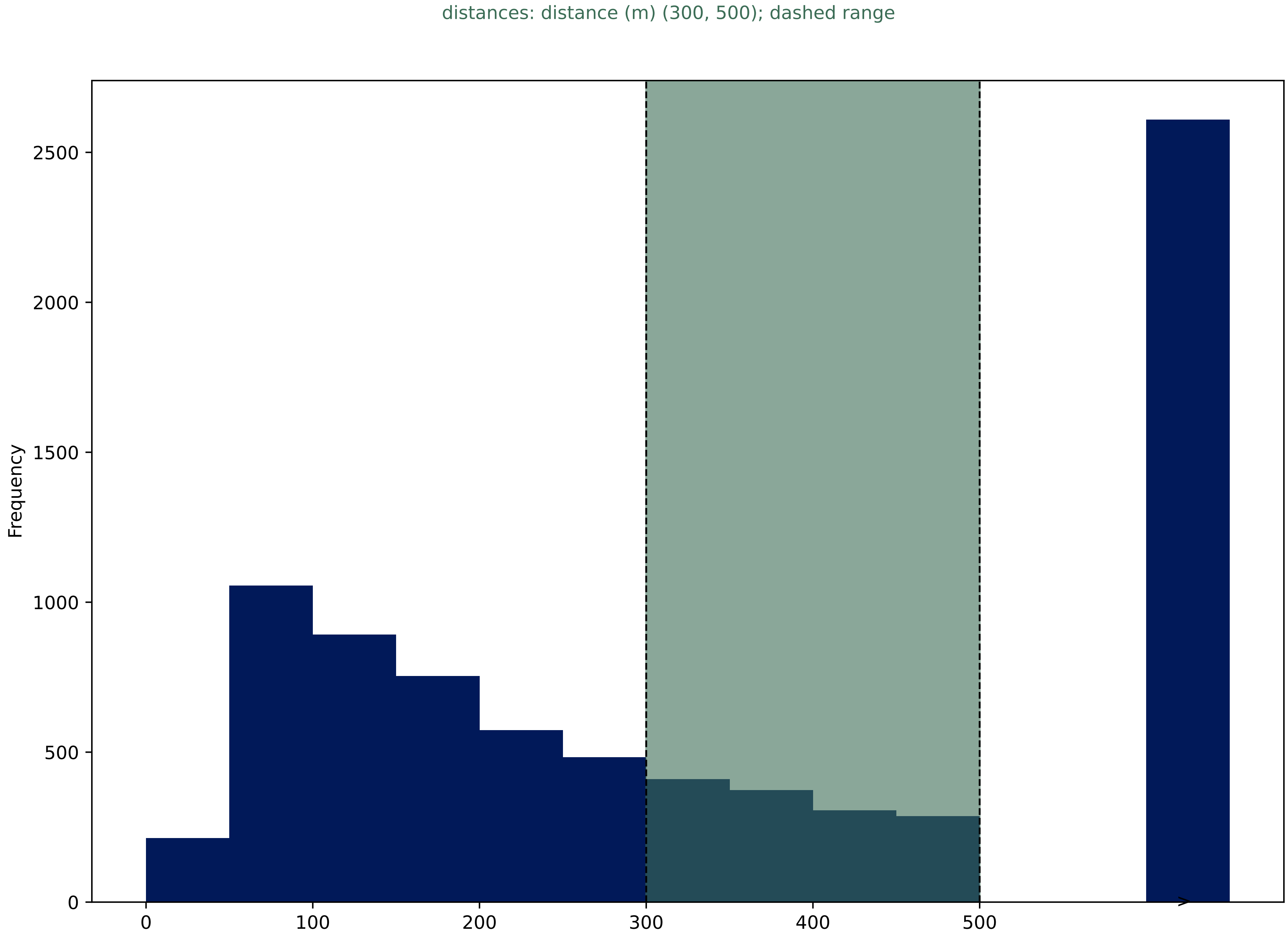

Distance to nearest park (m; up to 500m)

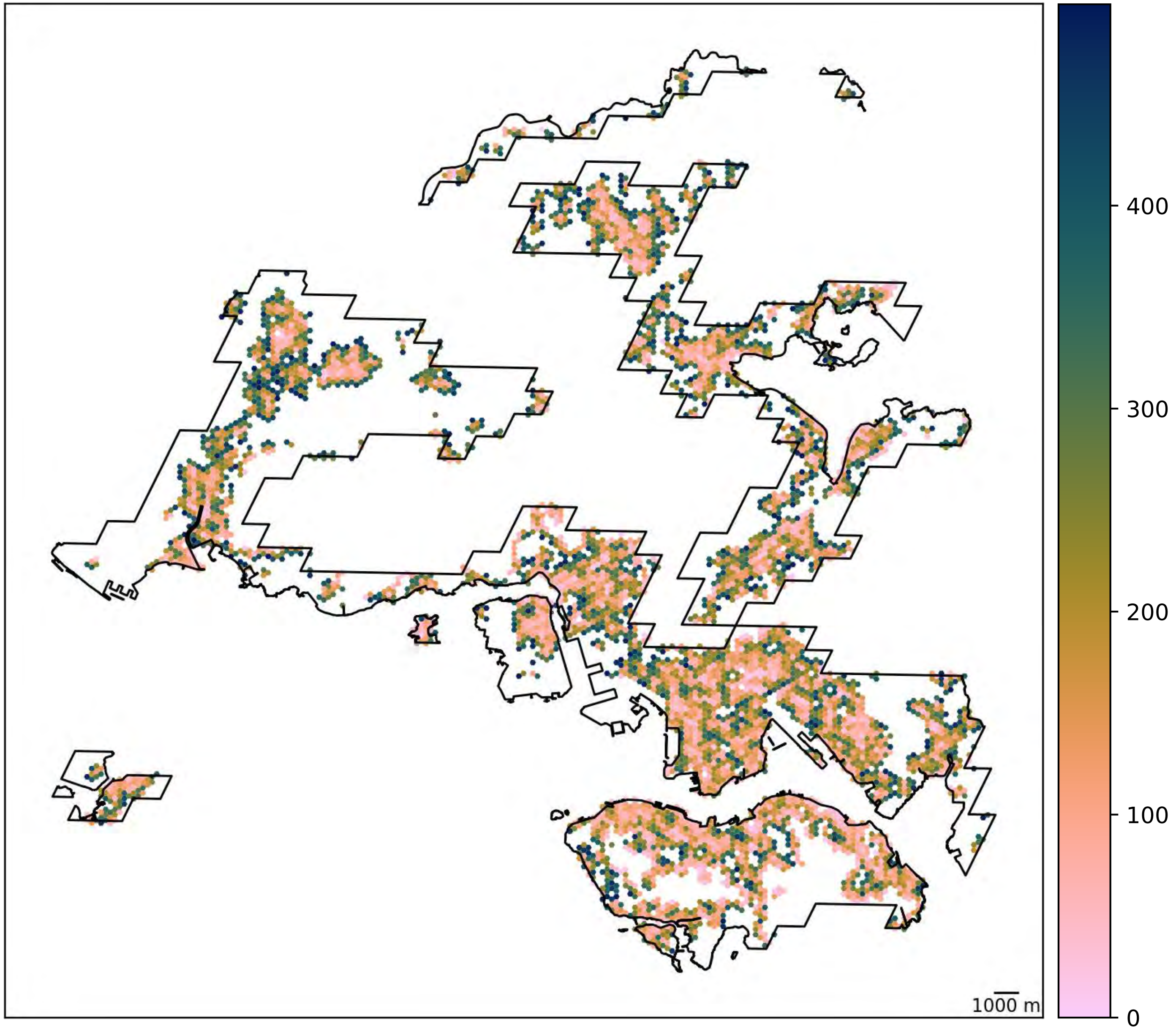

distances: Estimated Distance to nearest park (m; up to 500m) requirement for distances to destinations, measured up to a maximum distance target threshold of 500 metres

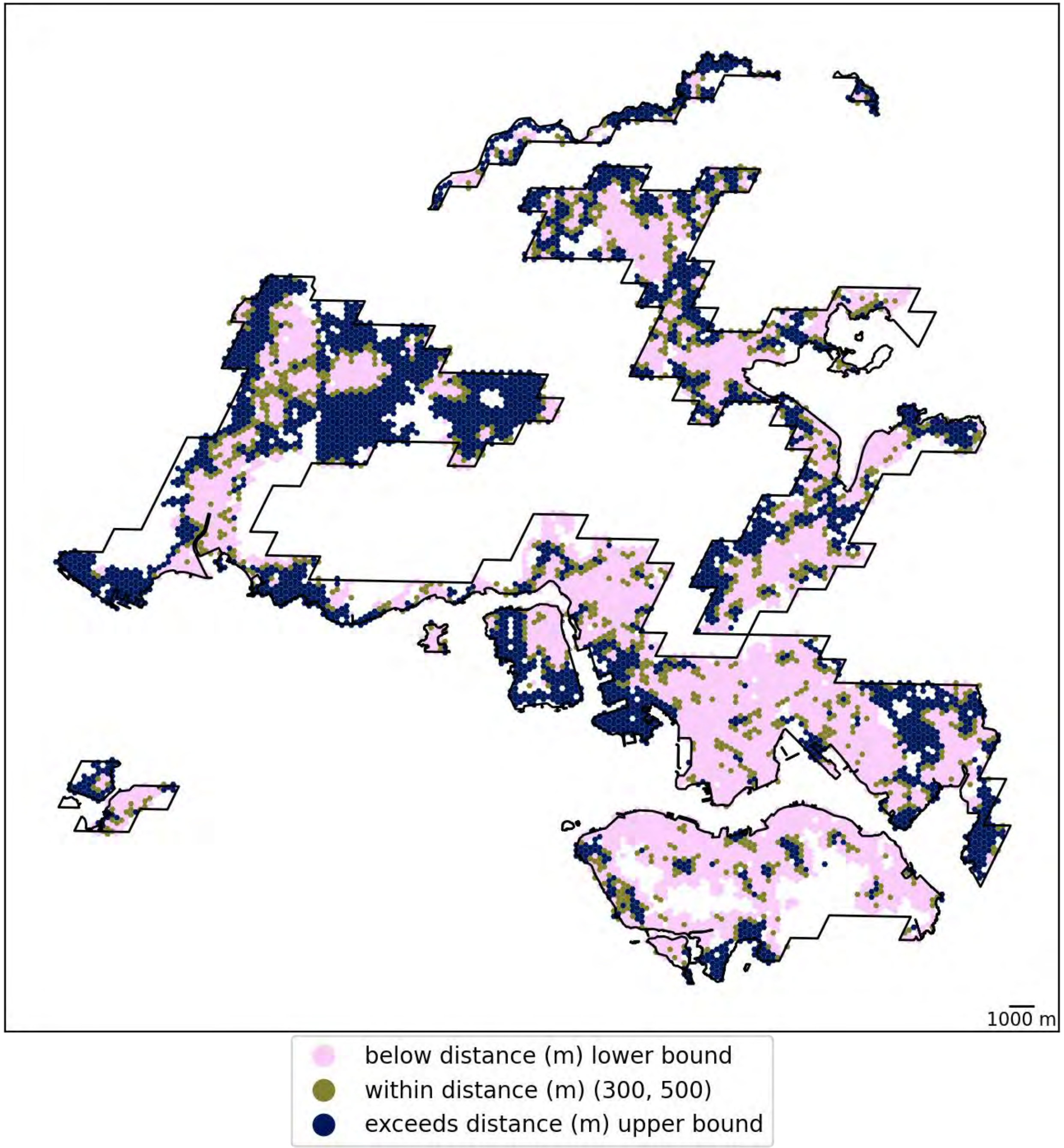

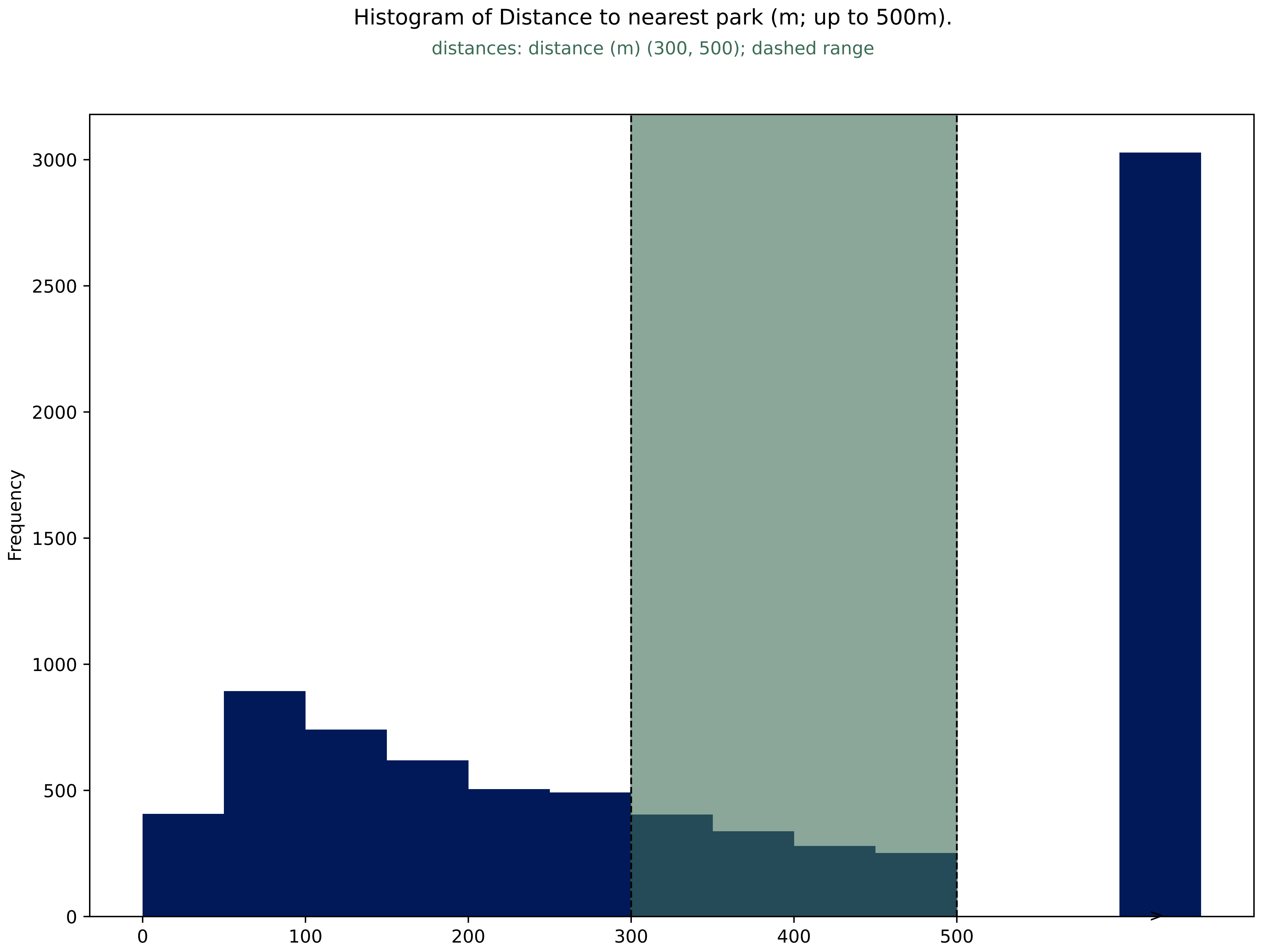

Histogram of Distance to nearest park (m; up to 500m).
distances: distance (m) (300, 500); dashed range
Frequency
3000
2500
2000
1500
1000
500
0
0
100
200
300
400
500
>

Asia, India, Chennai

Satellite imagery of urban study region (Bing) | Walkability, relative to city | Walkability, relative to 25 global cities

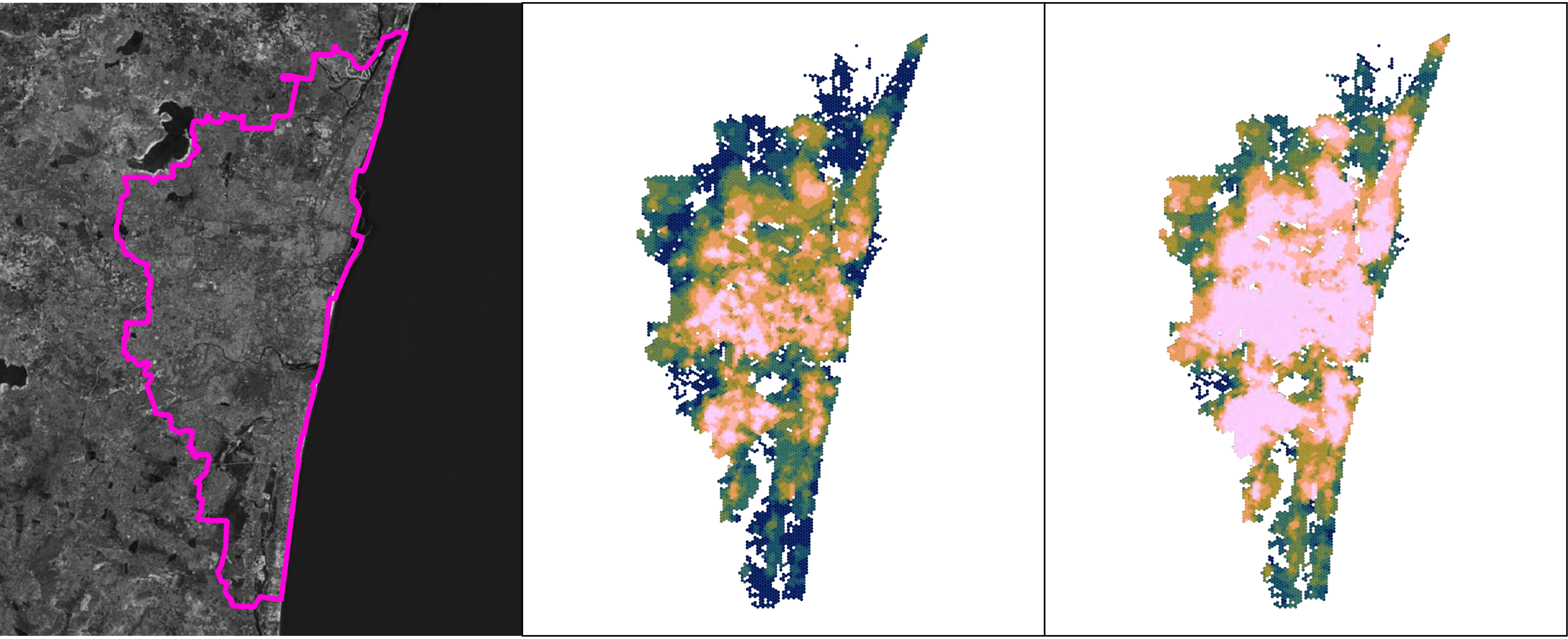

Urban boundary

0 10 20 km

Walkability score
- <-3
- -3 to -2
- -2 to -1
- -1 to 0
- 0 to 1
- 1 to 2
- 2 to 3
- ≥3

Walkability relative to all cities by component variables (2D histograms), and overall (histogram)

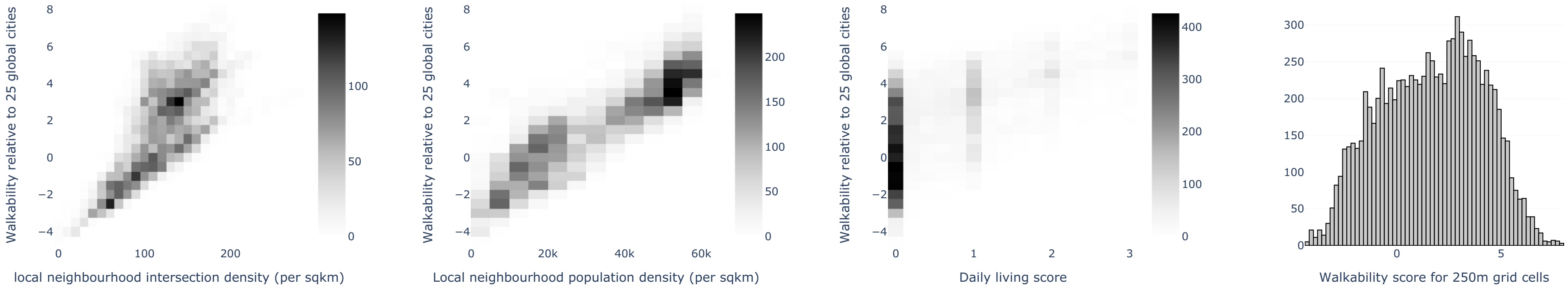

Mean 1000 m neighbourhood population per km²

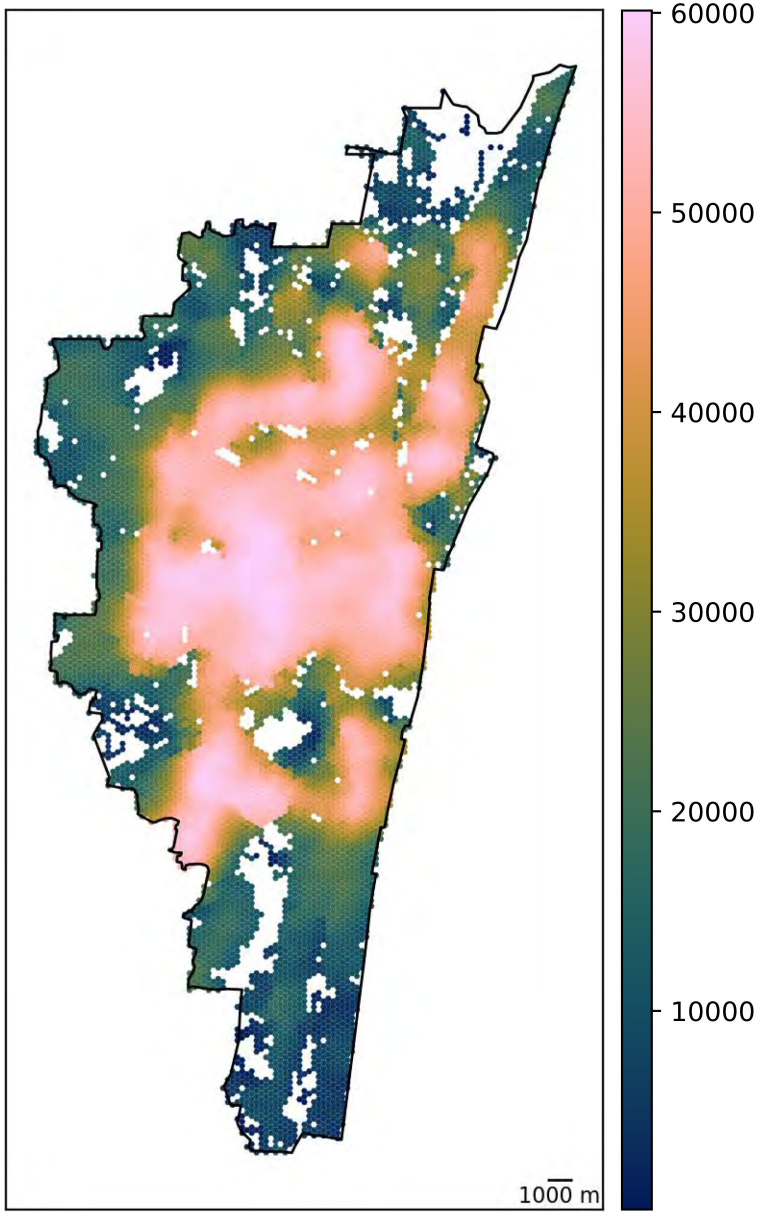

A: Estimated Mean 1000 m neighbourhood population per km² requirement for ≥80% probability of engaging in walking for transport

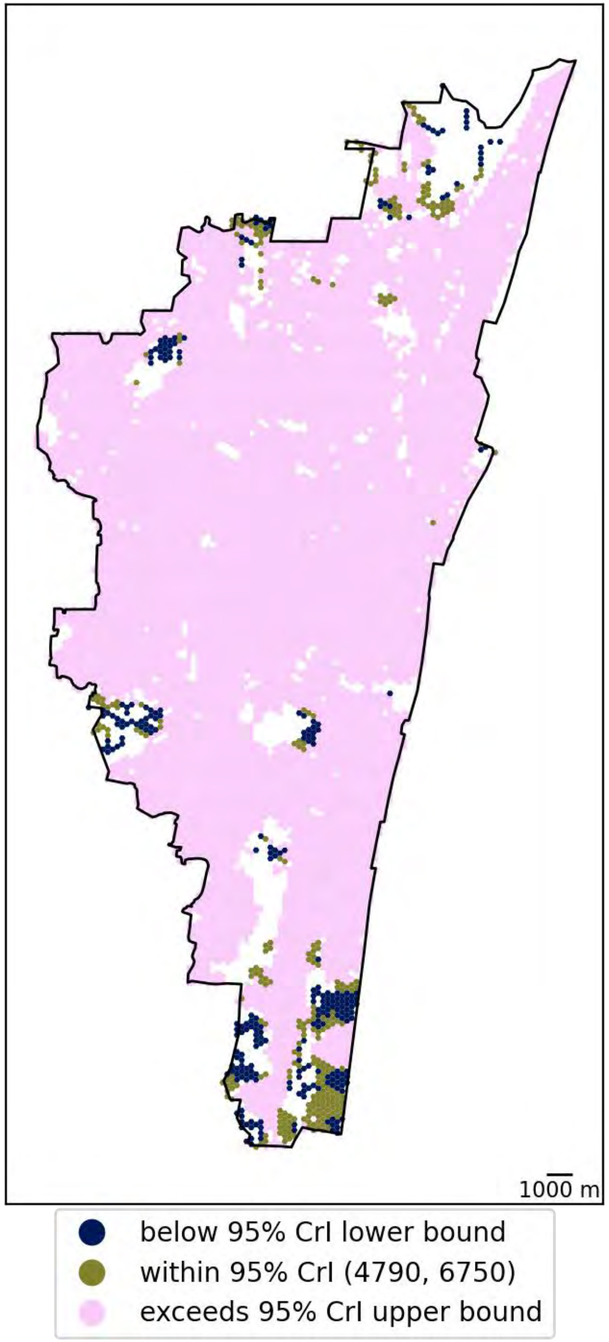

B: Estimated Mean 1000 m neighbourhood population per km² requirement for reaching the WHO's target of a ≥15% relative reduction in insufficient physical activity through walking

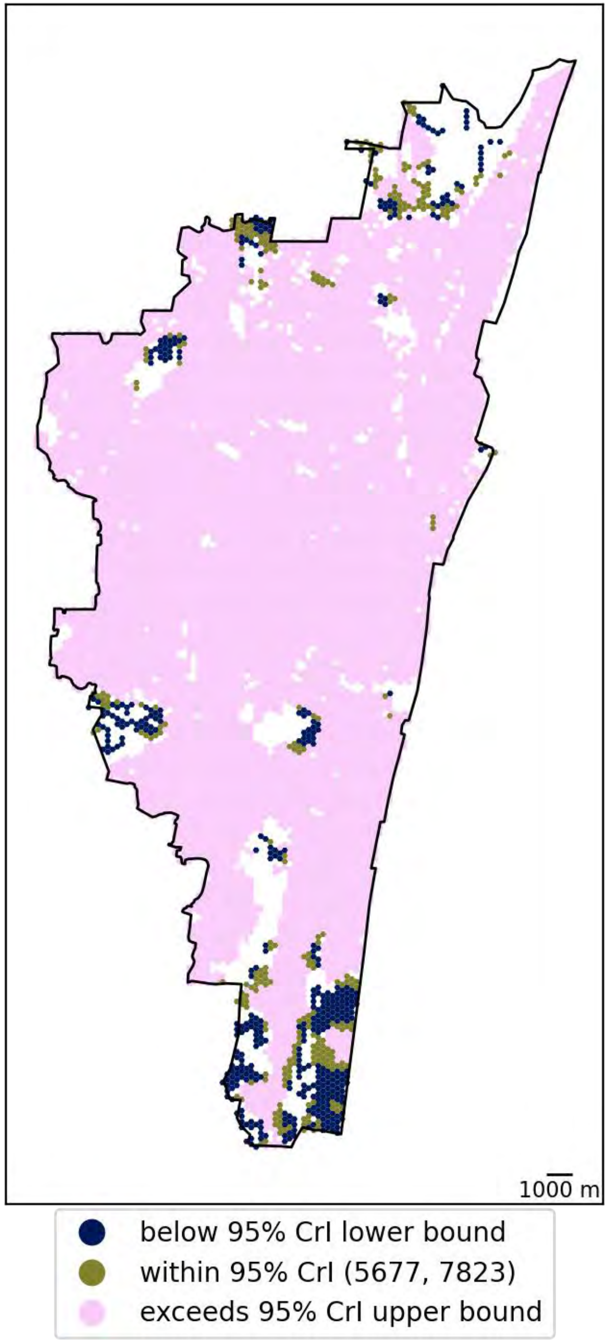

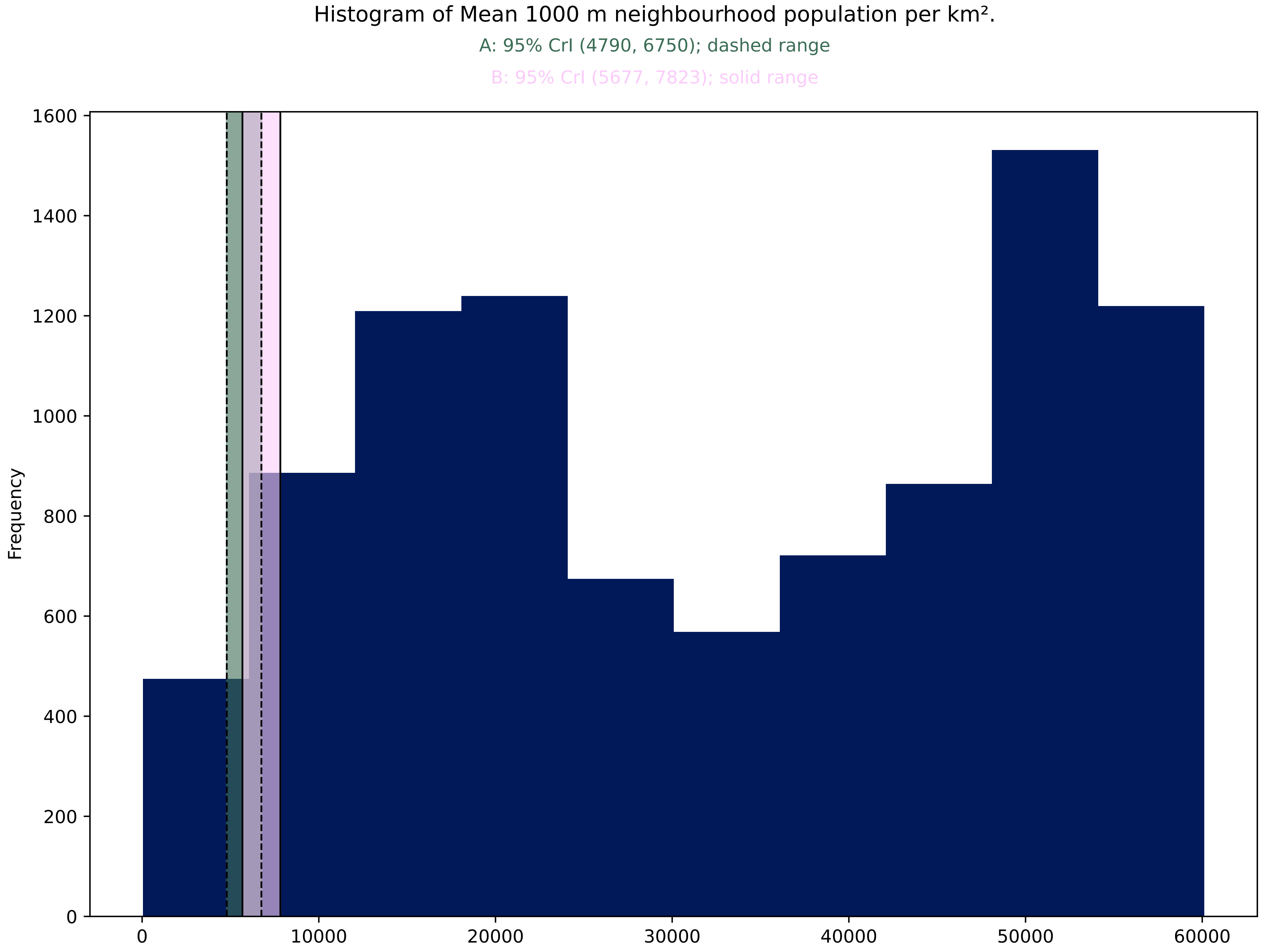
Histogram of Mean 1000 m neighbourhood population per km².
A: 95% CrI (4790, 6750); dashed range
B: 95% CrI (5677, 7823); solid range
Frequency
0
200
400
600
800
1000
1200
1400
1600
0
10000
20000
30000
40000
50000
60000

Mean 1000 m neighbourhood street intersections per km²

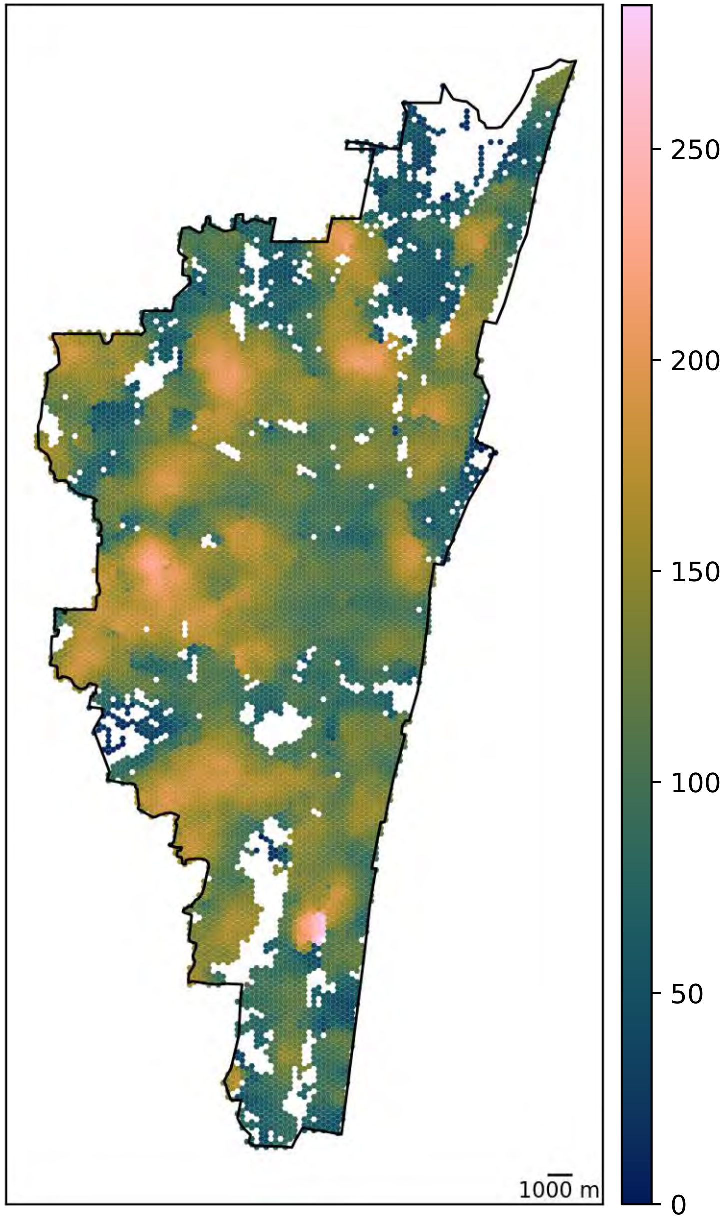

A: Estimated Mean 1000 m neighbourhood street intersections per km² requirement for ≥80% probability of engaging in walking for transport

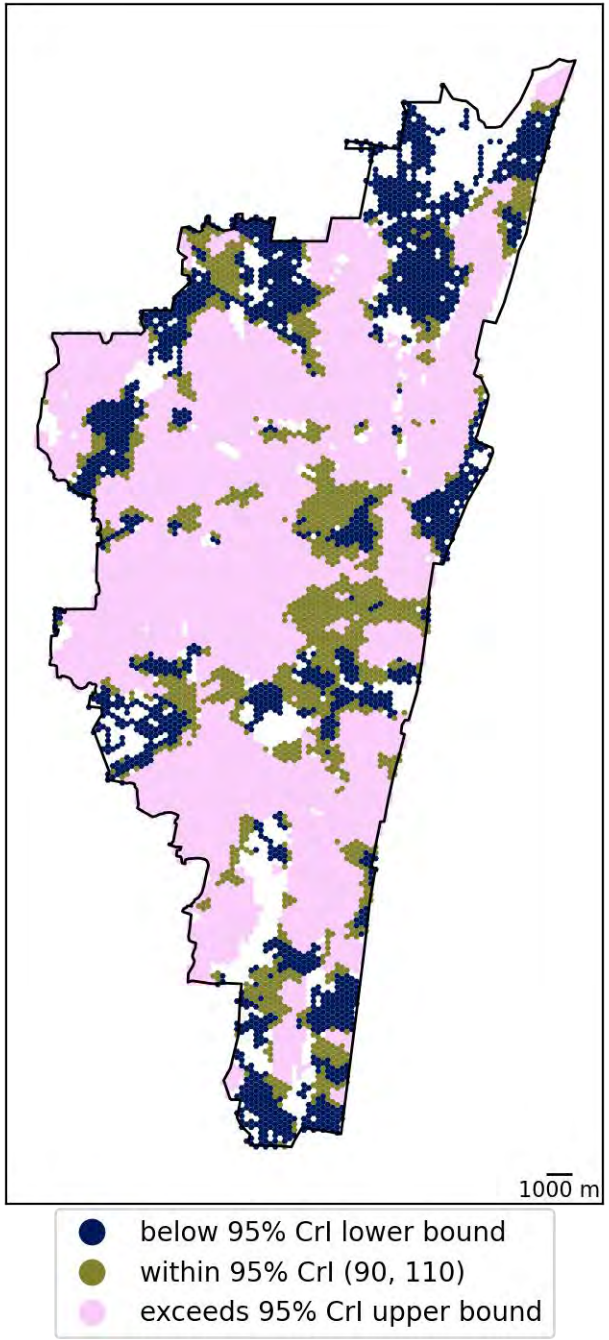

B: Estimated Mean 1000 m neighbourhood street intersections per km² requirement for reaching the WHO's target of a ≥15% relative reduction in insufficient physical activity through walking

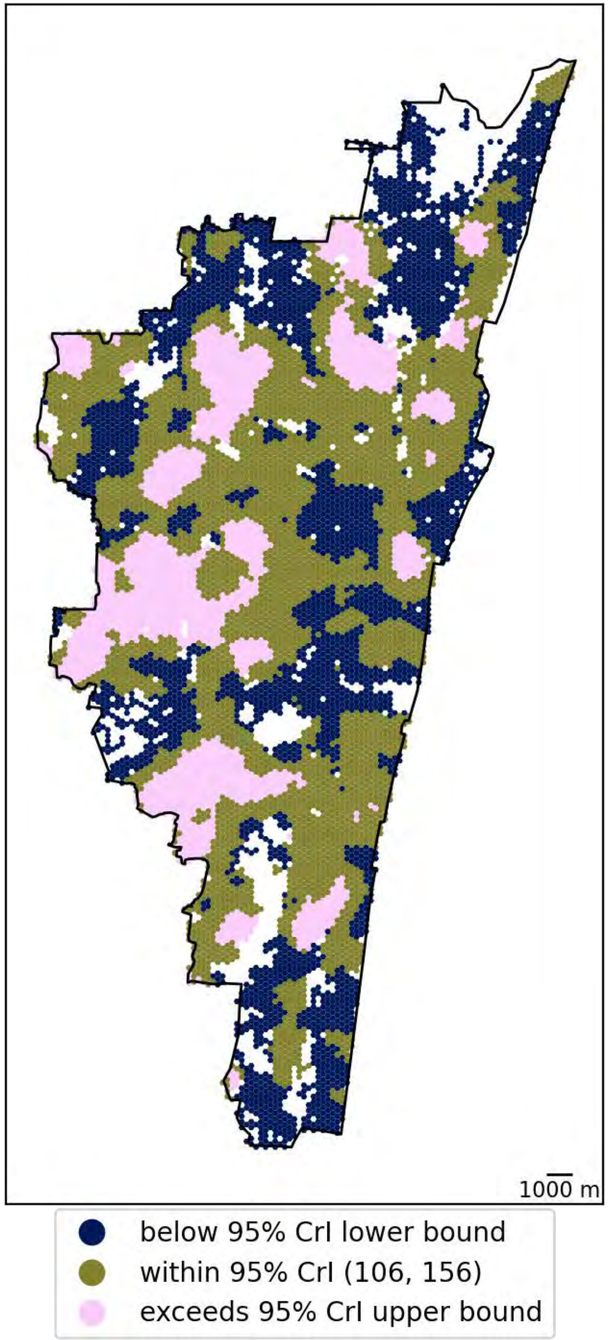

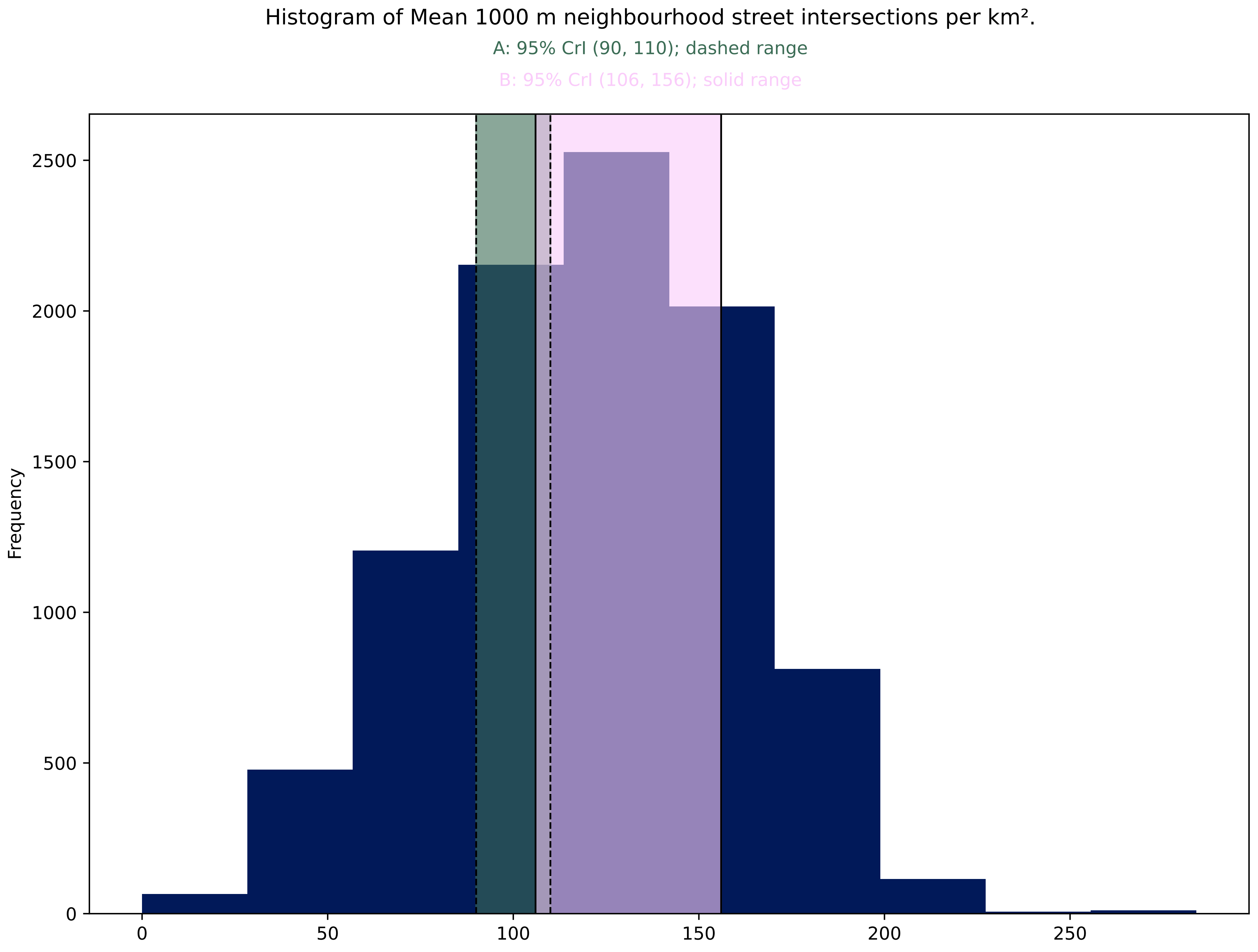
Histogram of Mean 1000 m neighbourhood street intersections per km².
A: 95% CrI (90, 110); dashed range
B: 95% CrI (106, 156); solid range
Frequency
0
500
1000
1500
2000
2500
0
50
100
150
200
250

Distance to nearest public transport stops (m; up to 500m)

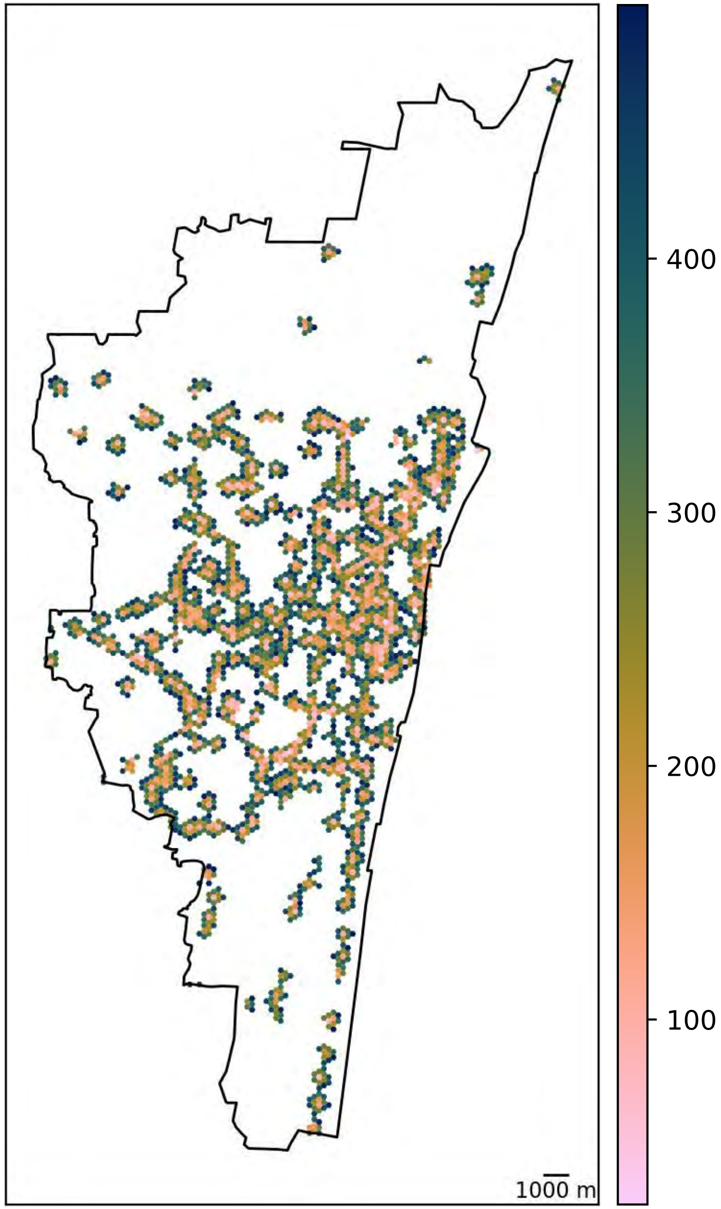

distances: Estimated Distance to nearest public transport stops (m; up to 500m) requirement for distances to destinations, measured up to a maximum distance target threshold of 500 metres

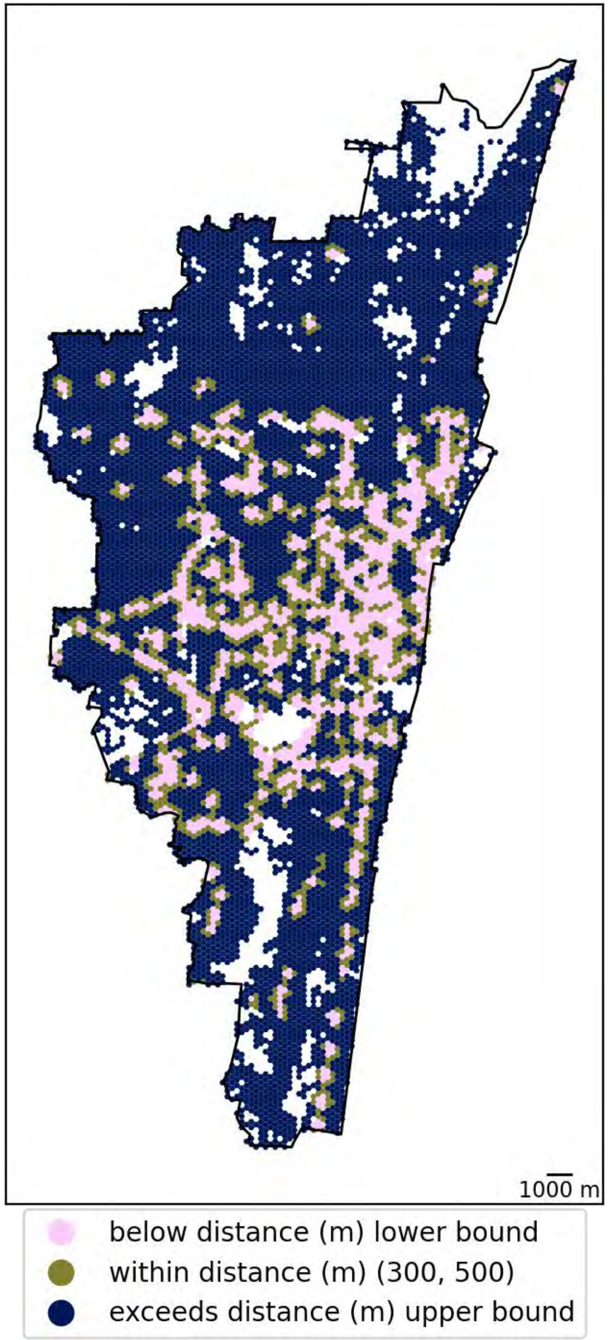

Histogram of Distance to nearest public transport stops (m; up to 500m).

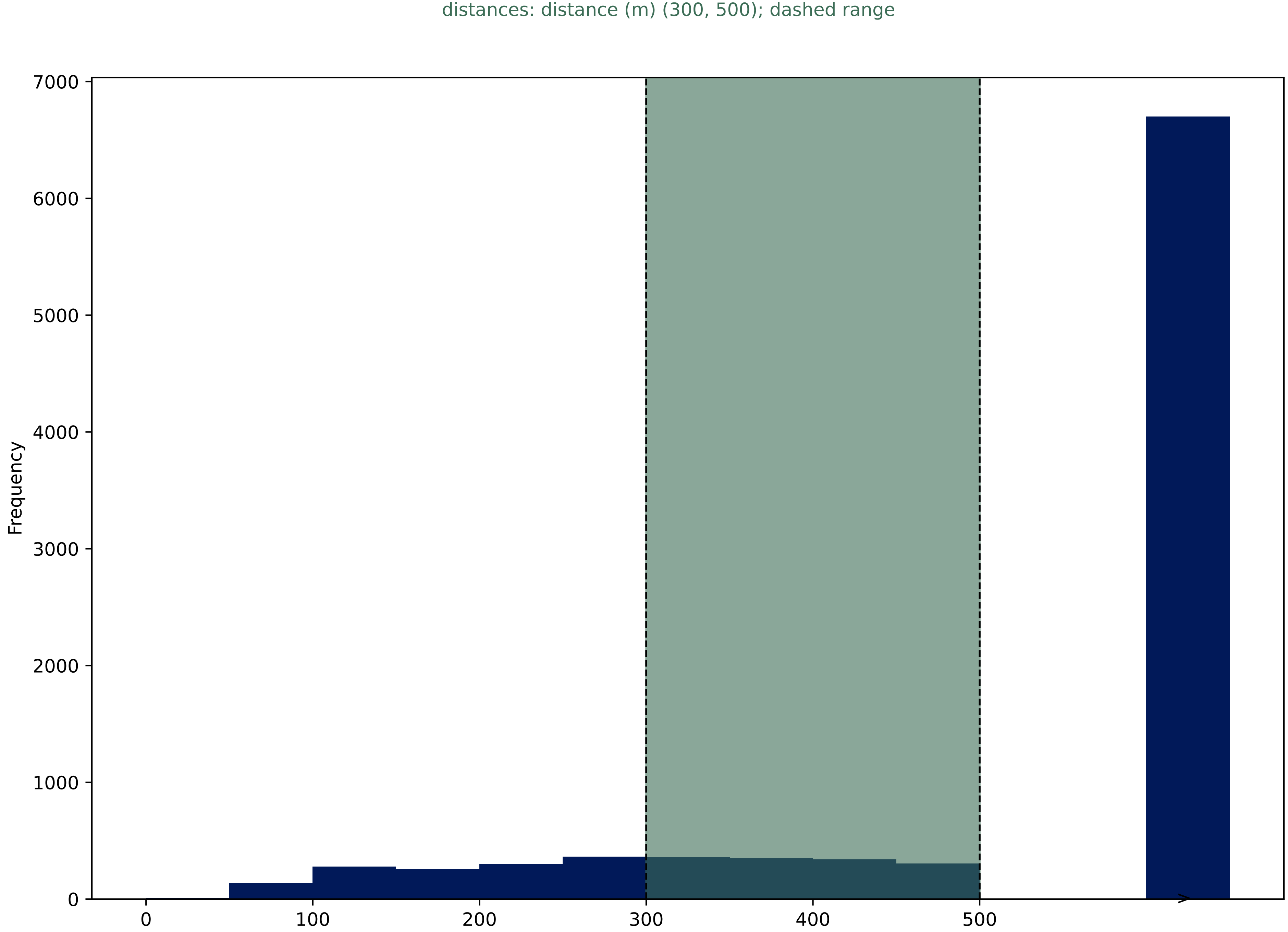

Distance to nearest park (m; up to 500m)

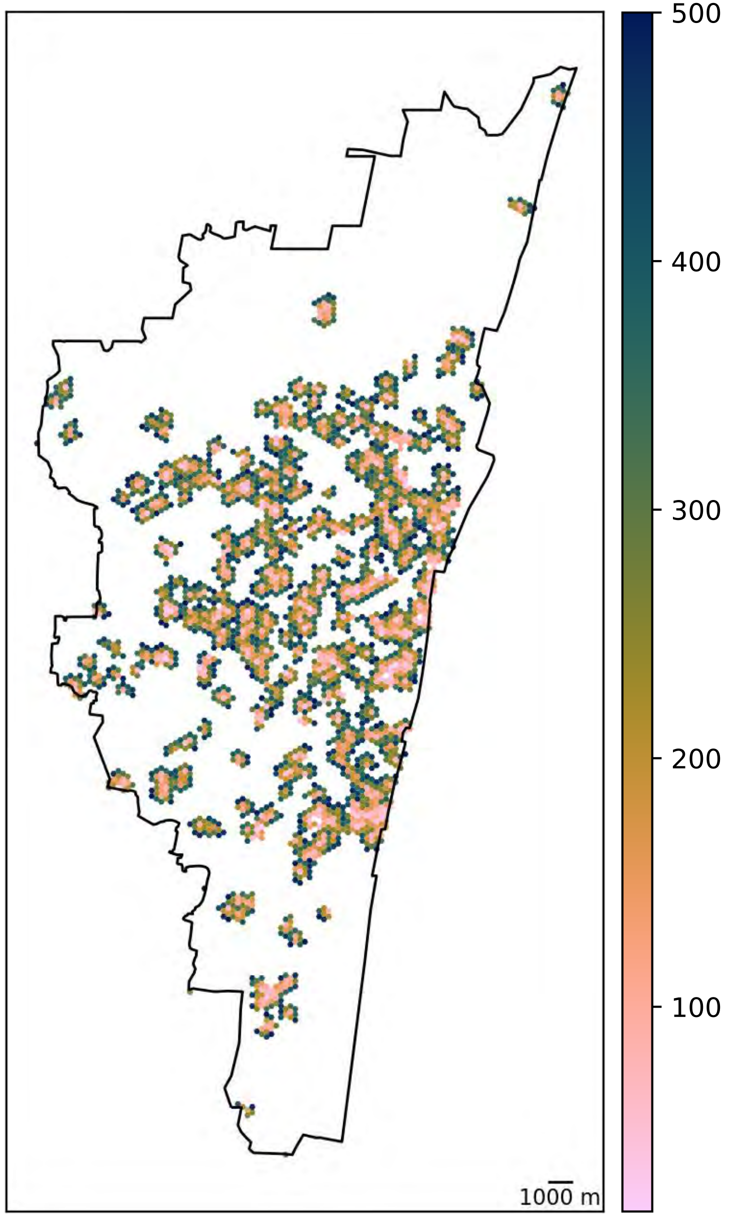

distances: Estimated Distance to nearest park (m; up to 500m) requirement for distances to destinations, measured up to a maximum distance target threshold of 500 metres

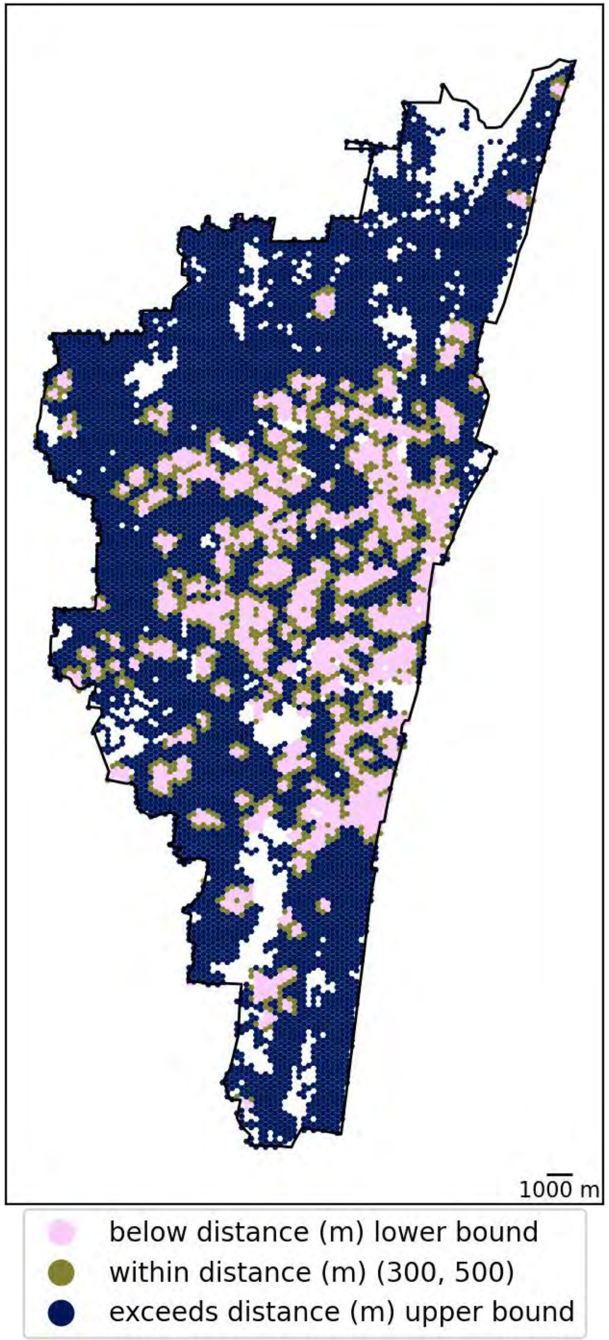

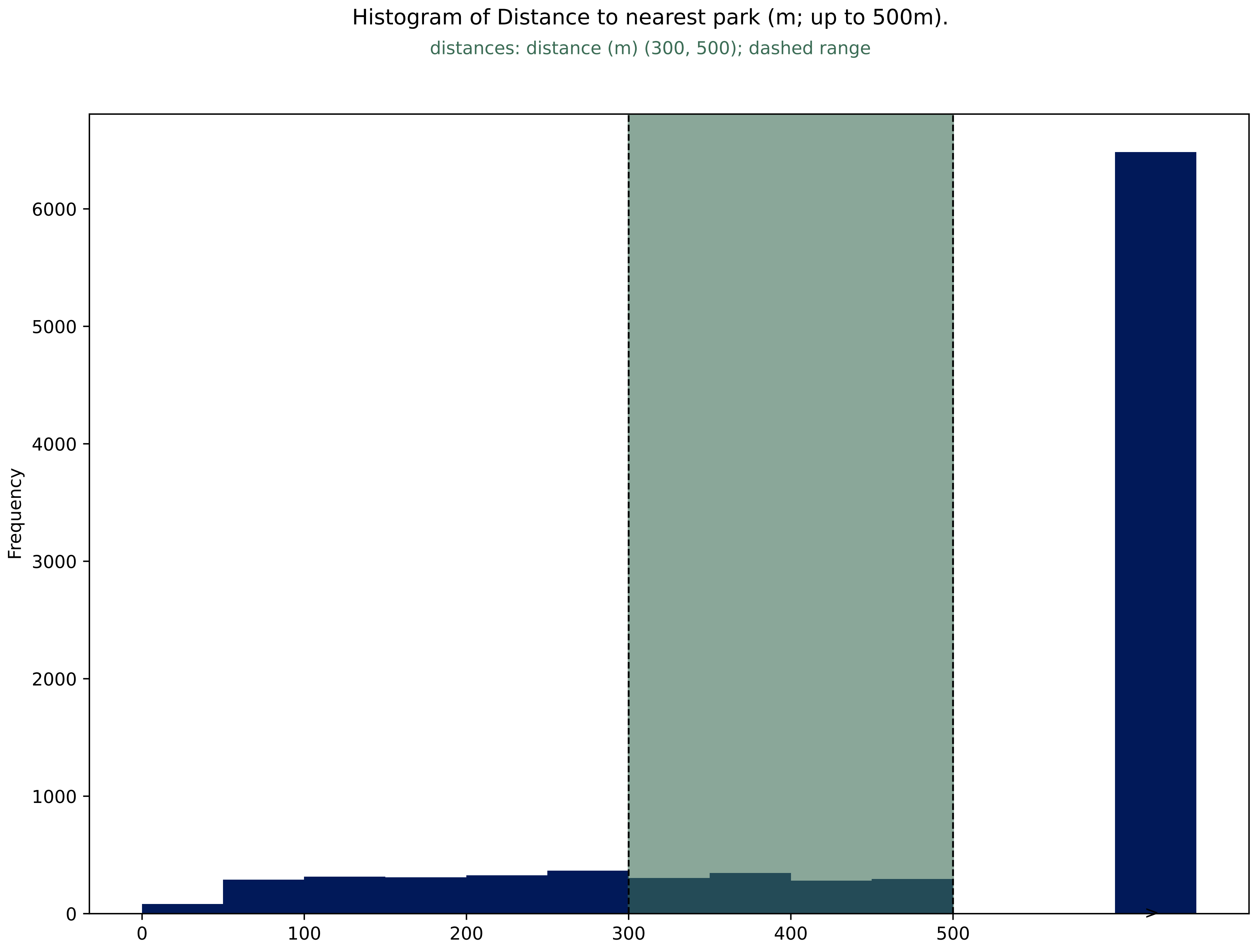
Histogram of Distance to nearest park (m; up to 500m).
distances: distance (m) (300, 500); dashed range
Frequency
0
1000
2000
3000
4000
5000
6000
0
100
200
300
400
500
>

Asia, Thailand, Bangkok

Satellite imagery of urban study region (Bing) | Walkability, relative to city | Walkability, relative to 25 global cities

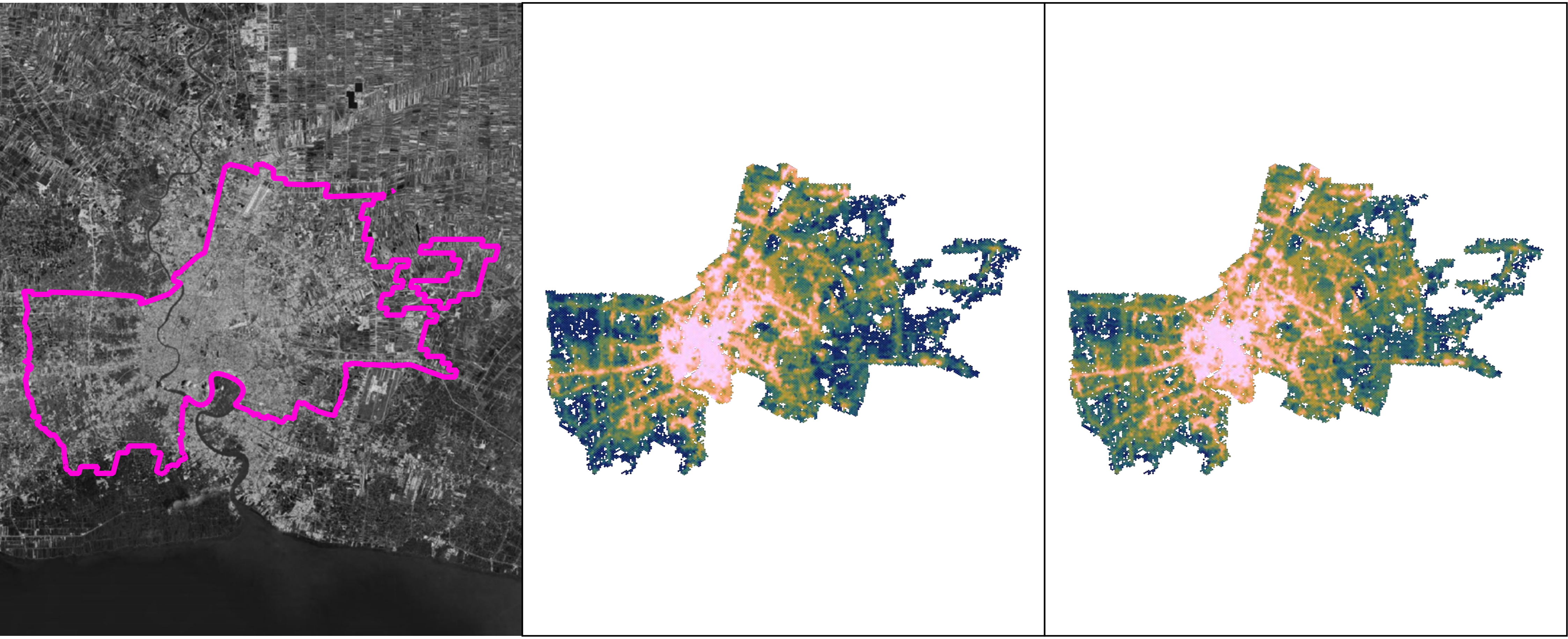

Urban boundary

Walkability score
- <-3
- -3 to -2
- -2 to -1
- -1 to 0
- 0 to 1
- 1 to 2
- 2 to 3
- ≥3

Walkability relative to all cities by component variables (2D histograms), and overall (histogram)

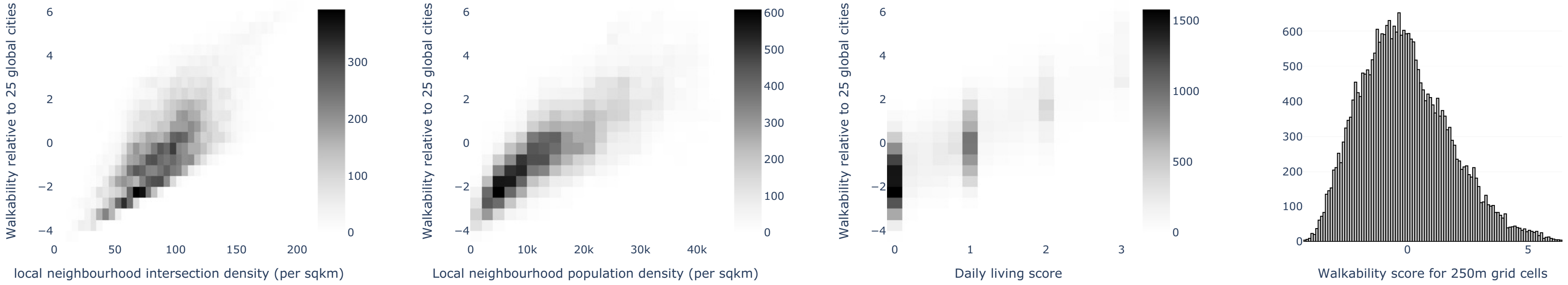

Mean 1000 m neighbourhood population per km²

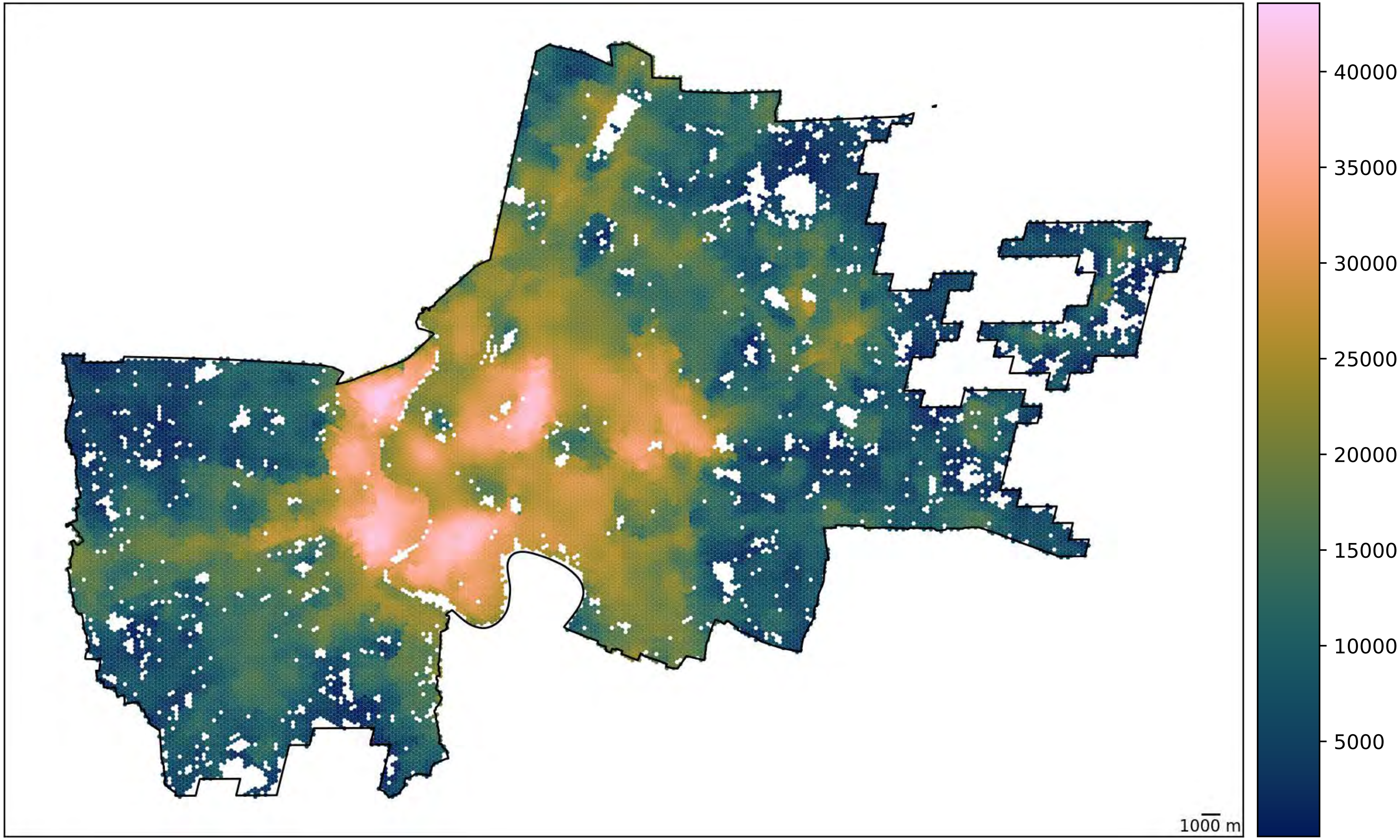

A: Estimated Mean 1000 m neighbourhood population per km² requirement for ≥80% probability of engaging in walking for transport

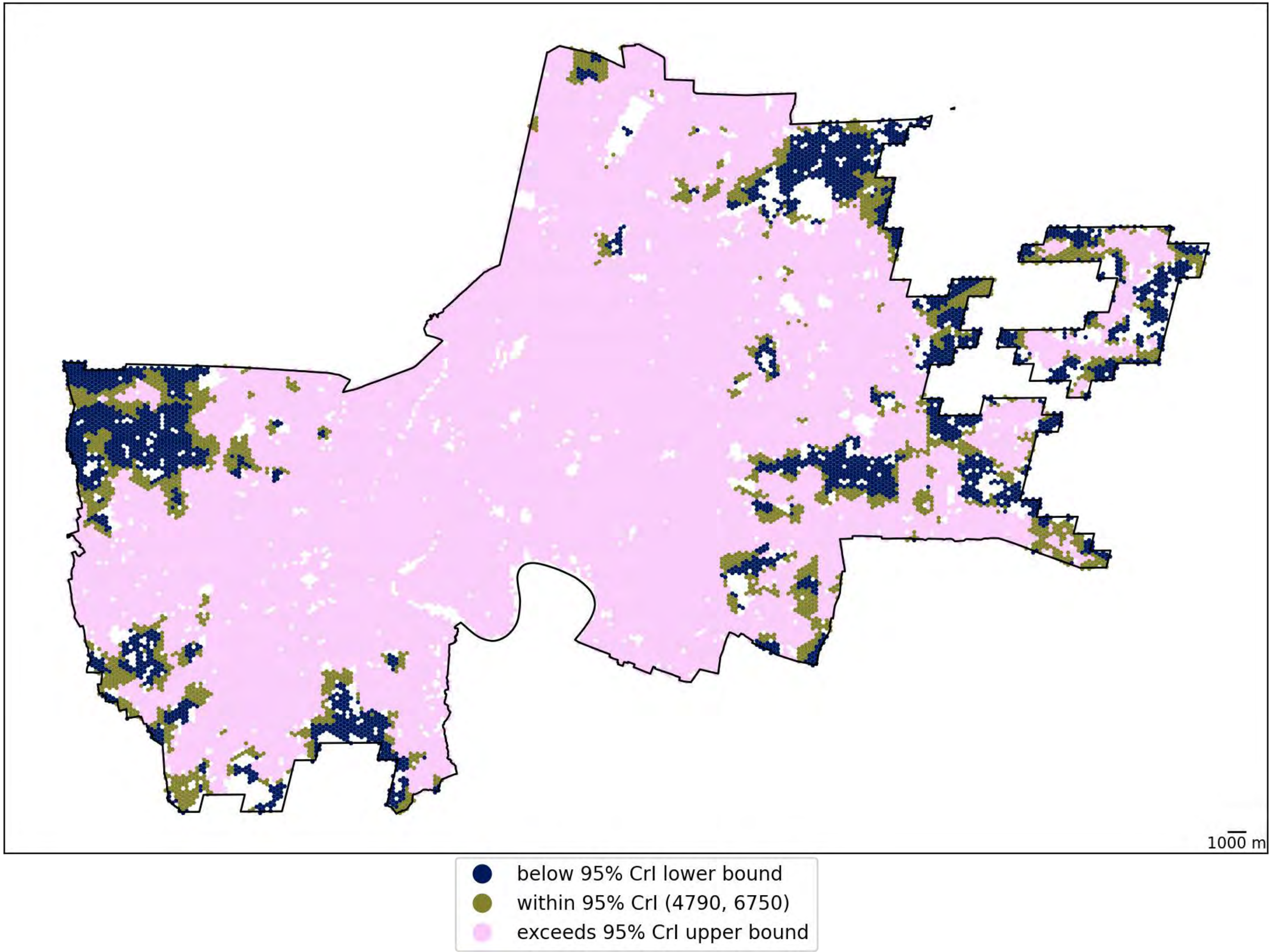

B: Estimated Mean 1000 m neighbourhood population per km² requirement for reaching the WHO's target of a ≥15% relative reduction in insufficient physical activity through walking

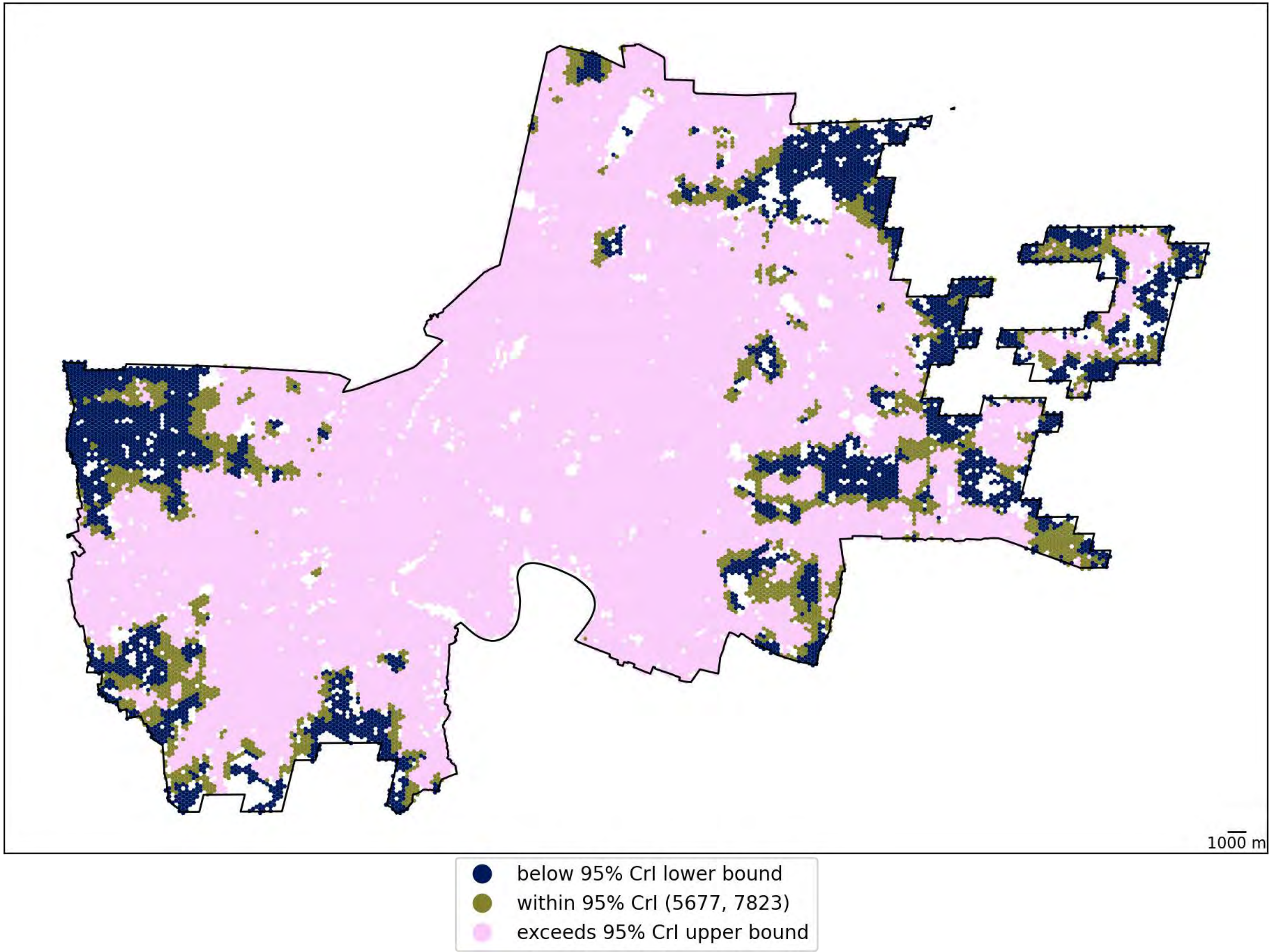

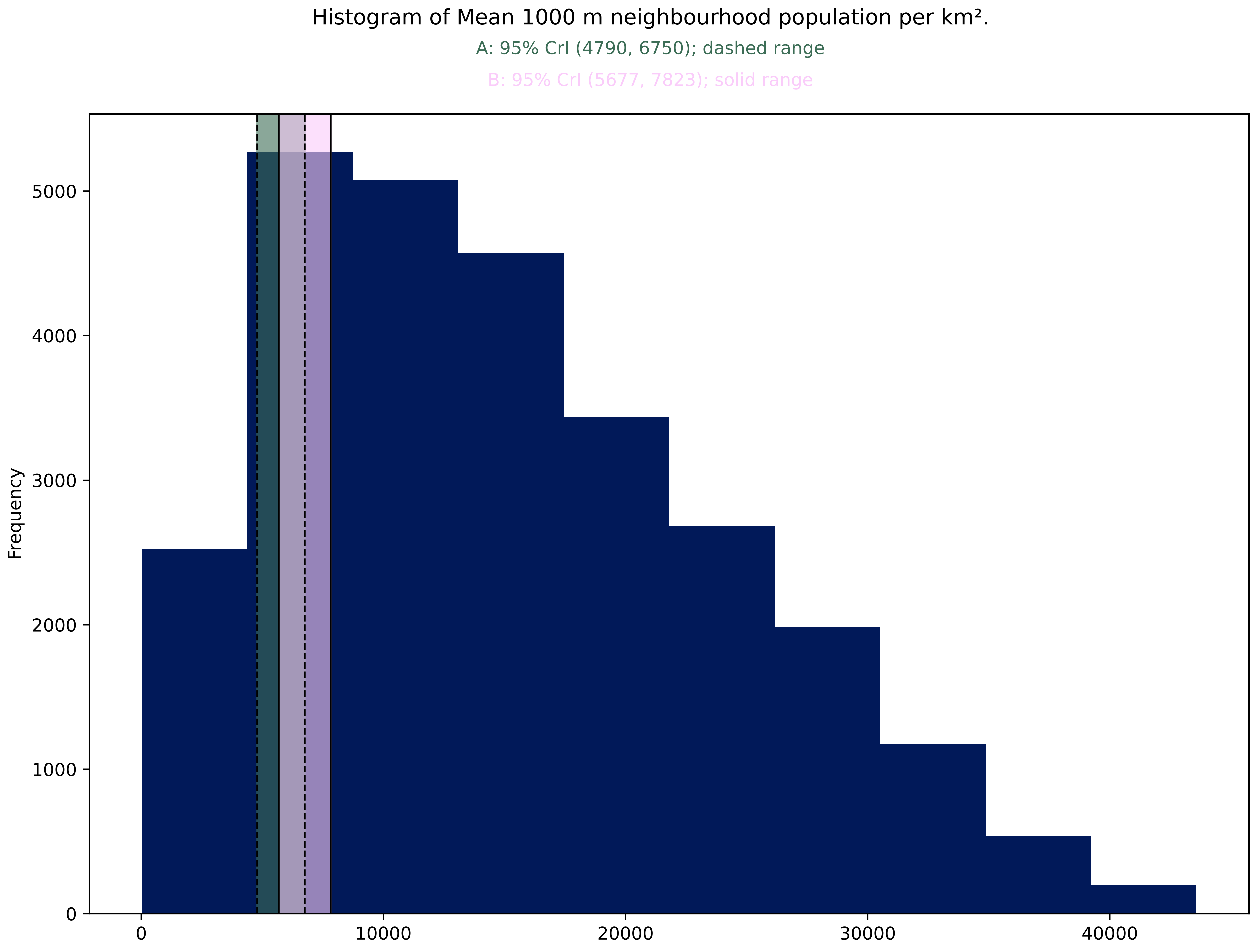
Histogram of Mean 1000 m neighbourhood population per km².
A: 95% CrI (4790, 6750); dashed range
B: 95% CrI (5677, 7823); solid range
Frequency
0
1000
2000
3000
4000
5000
0
10000
20000
30000
40000

Asia, Thailand, Bangkok

Satellite imagery of urban study region (Bing)

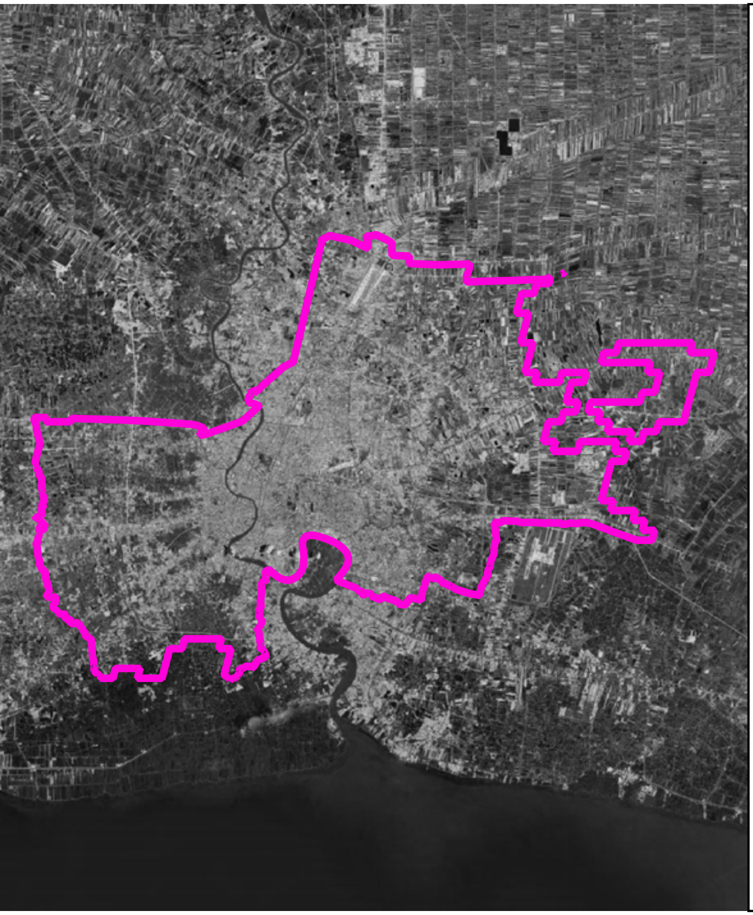

Urban boundary

Walkability, relative to city

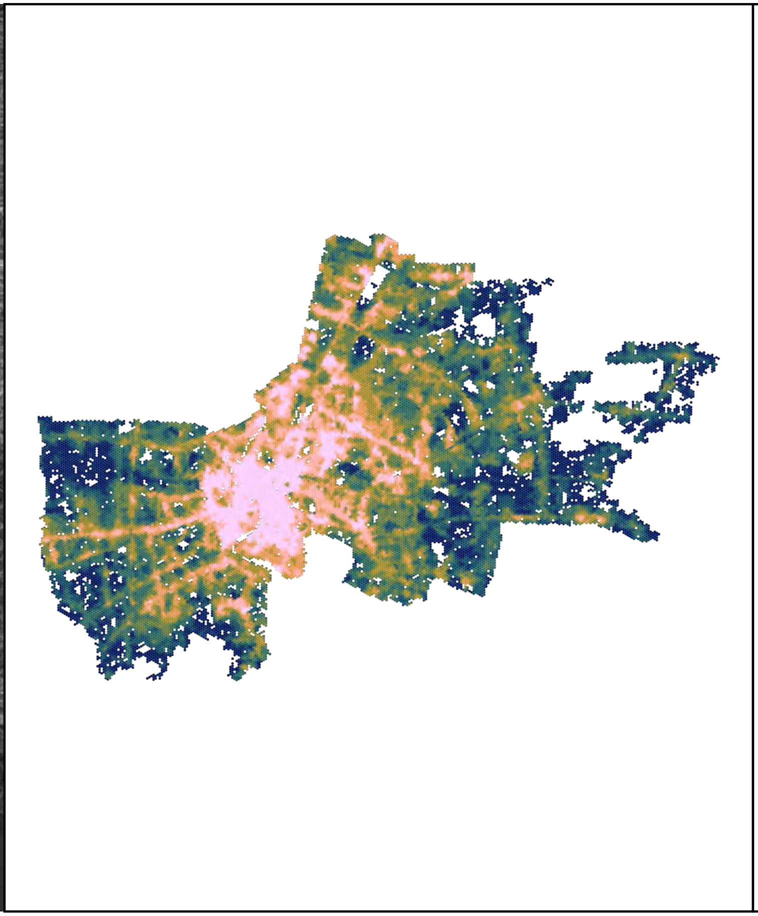

Walkability, relative to 25 global cities

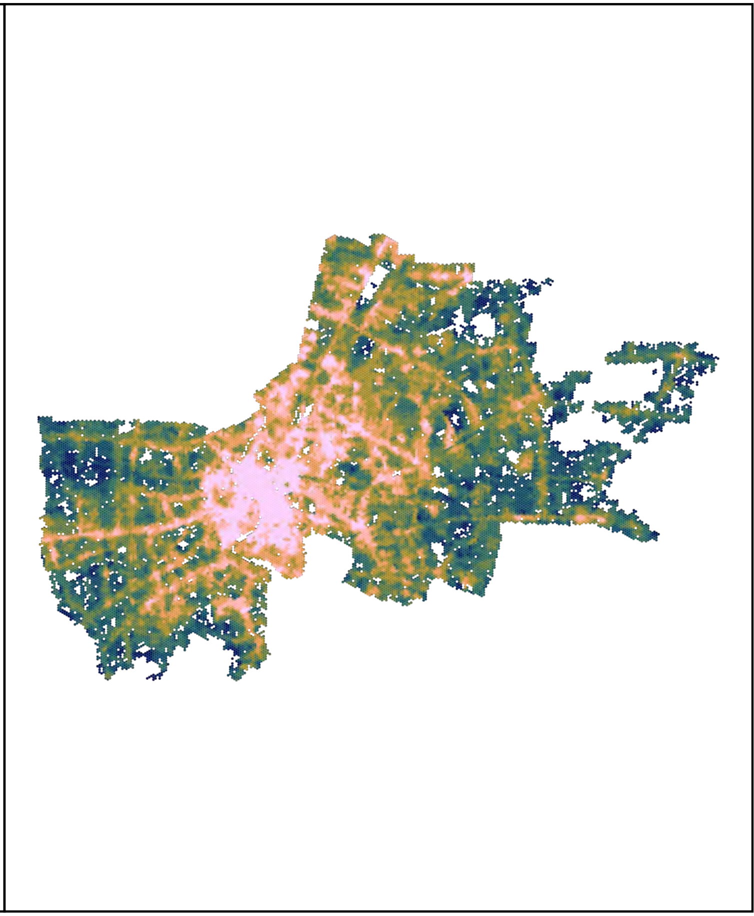

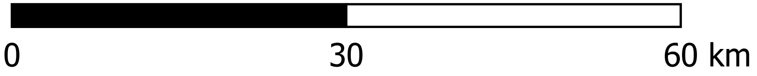

Walkability score

- <-3
- -3 to -2
- -2 to -1
- -1 to 0
- 0 to 1
- 1 to 2
- 2 to 3
- ≥3

Walkability relative to all cities by component variables (2D histograms), and overall (histogram)

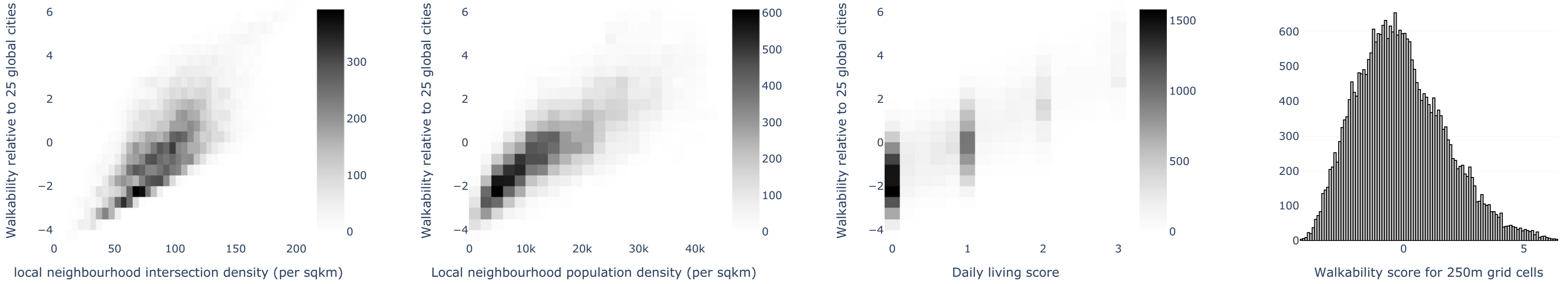

Mean 1000 m neighbourhood street intersections per km²

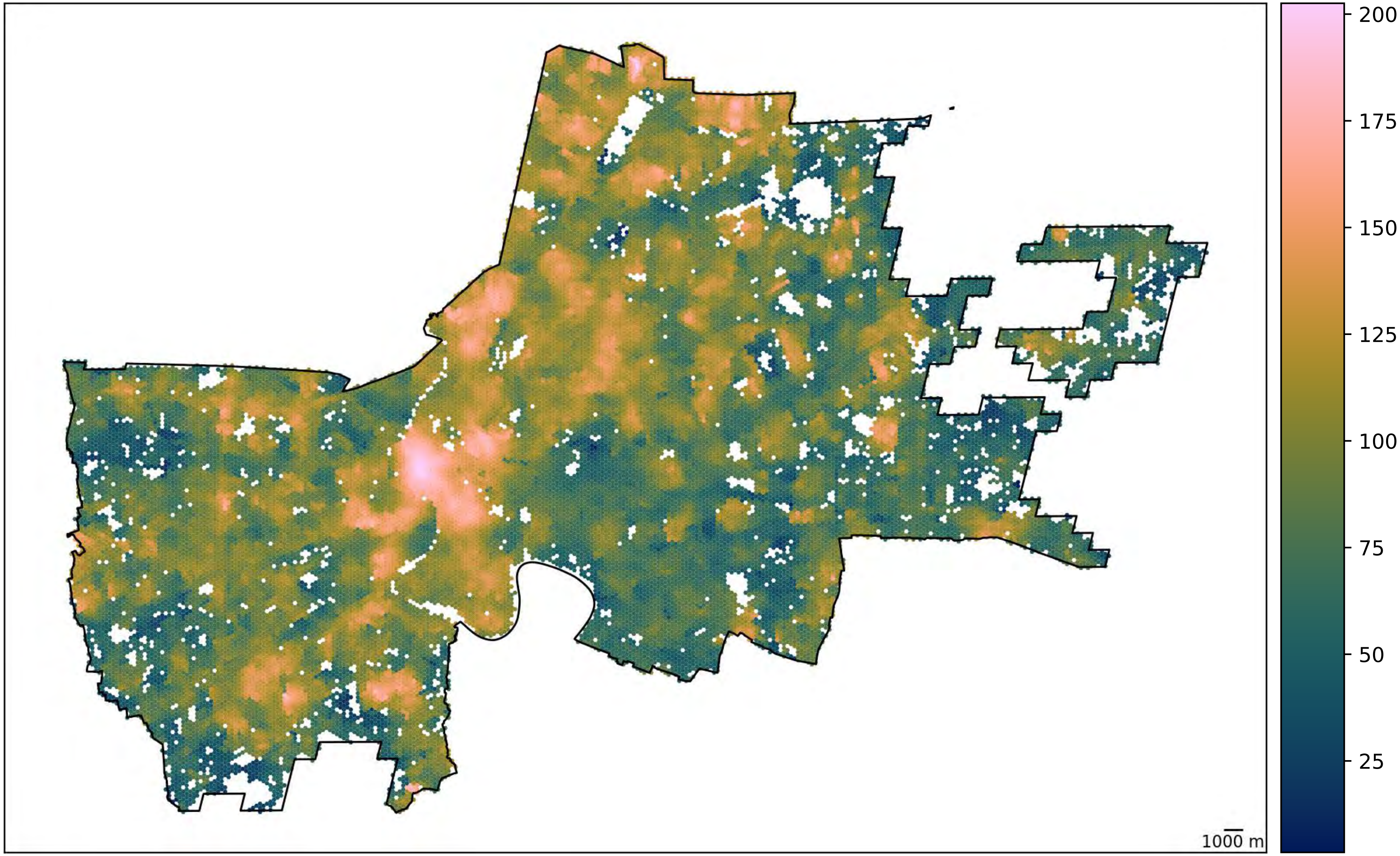

A: Estimated Mean 1000 m neighbourhood street intersections per km² requirement for ≥80% probability of engaging in walking for transport

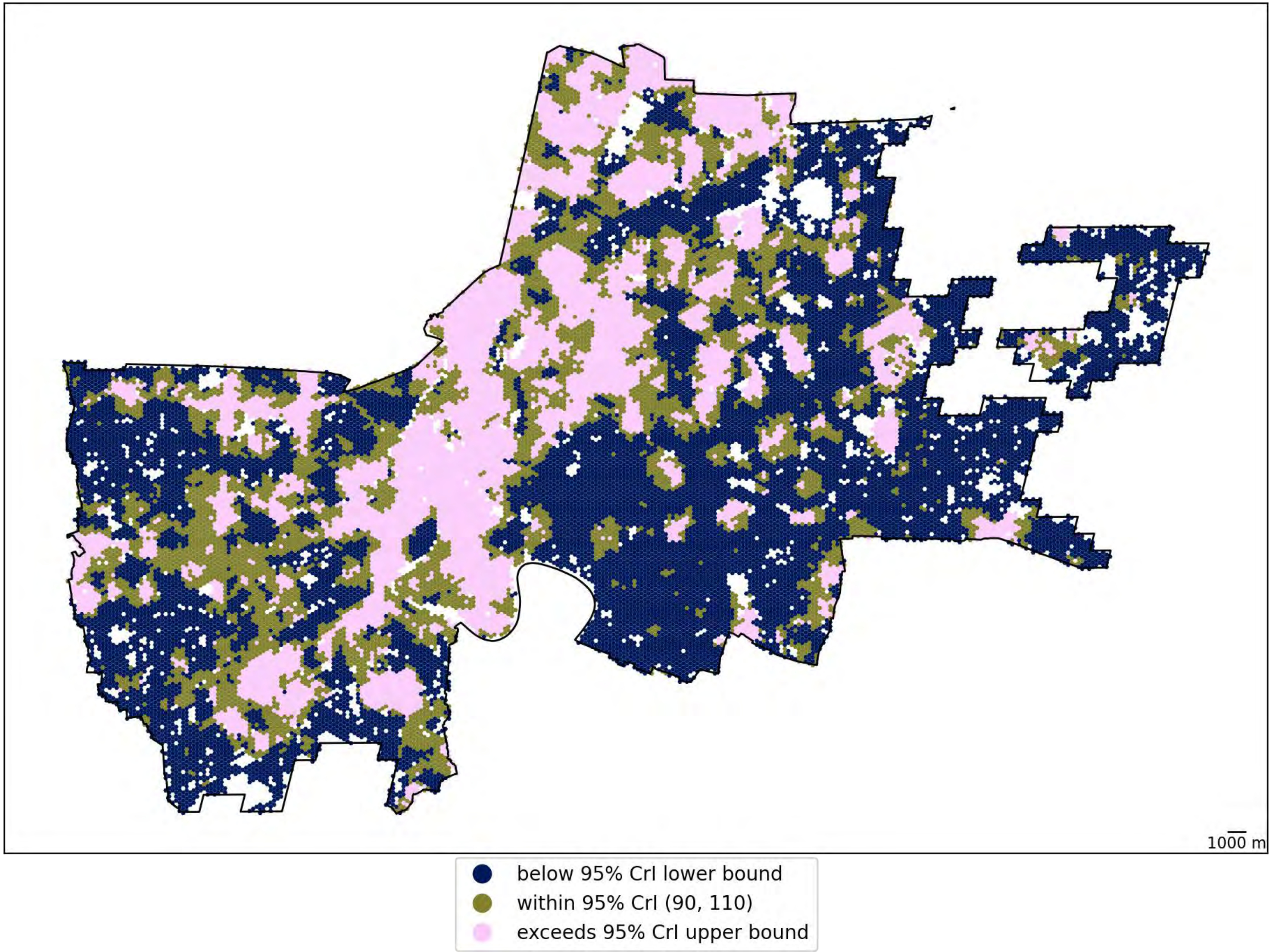

B: Estimated Mean 1000 m neighbourhood street intersections per km² requirement for reaching the WHO's target of a ≥15% relative reduction in insufficient physical activity through walking

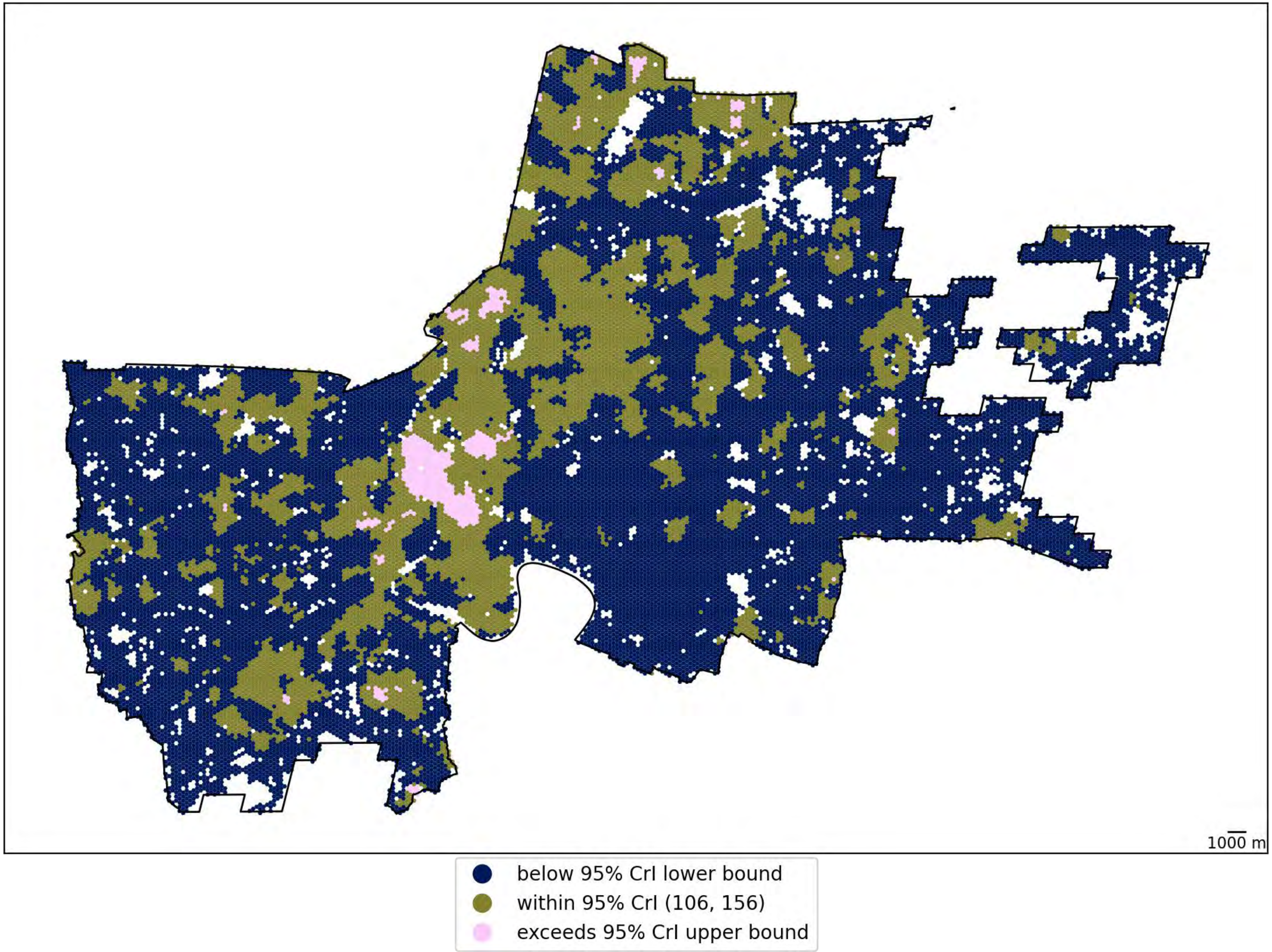

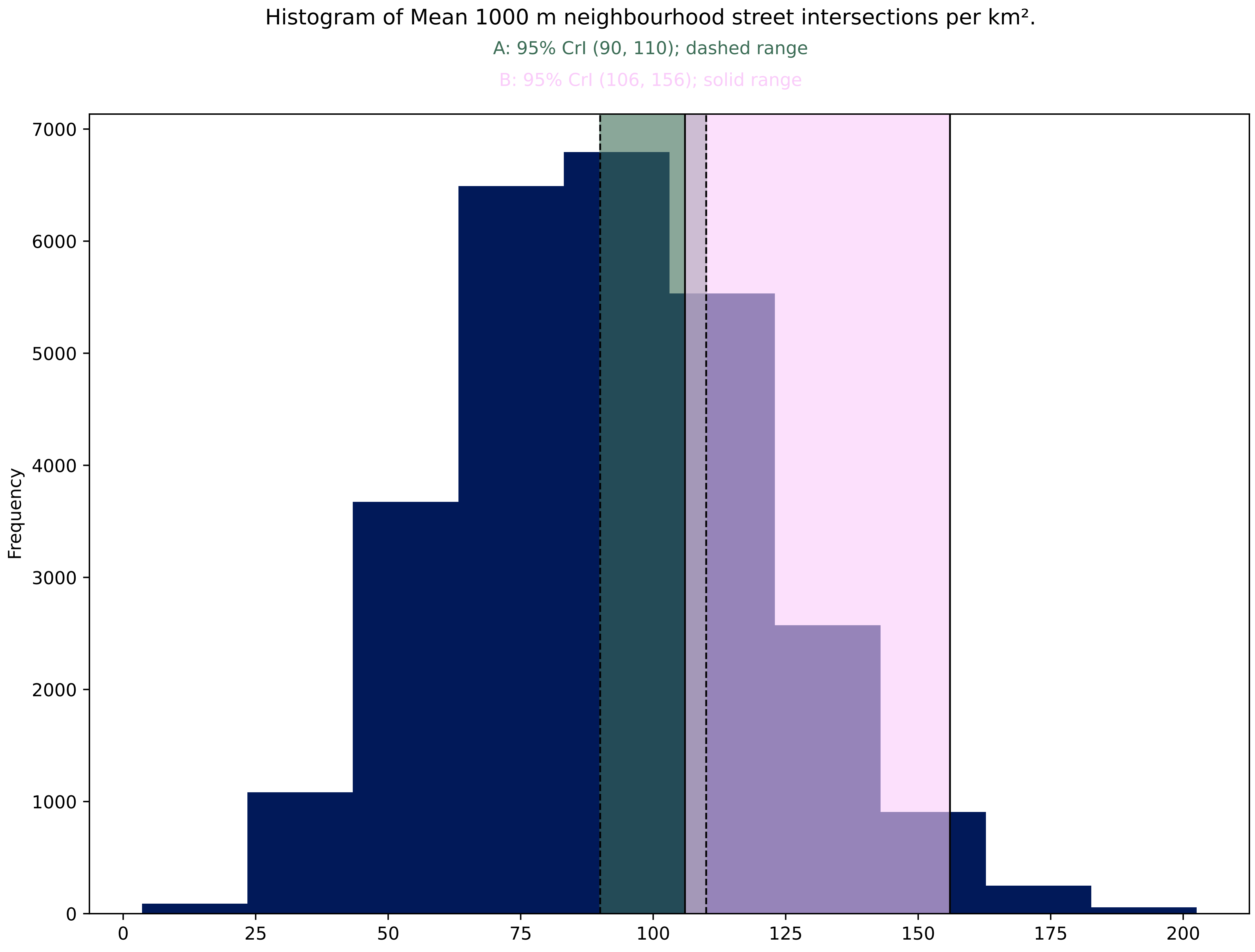

Histogram of Mean 1000 m neighbourhood street intersections per km².
A: 95% CrI (90, 110); dashed range
B: 95% CrI (106, 156); solid range
Frequency
7000
6000
5000
4000
3000
2000
1000
0
0
25
50
75
100
125
150
175
200

Distance to nearest public transport stops (m; up to 500m)

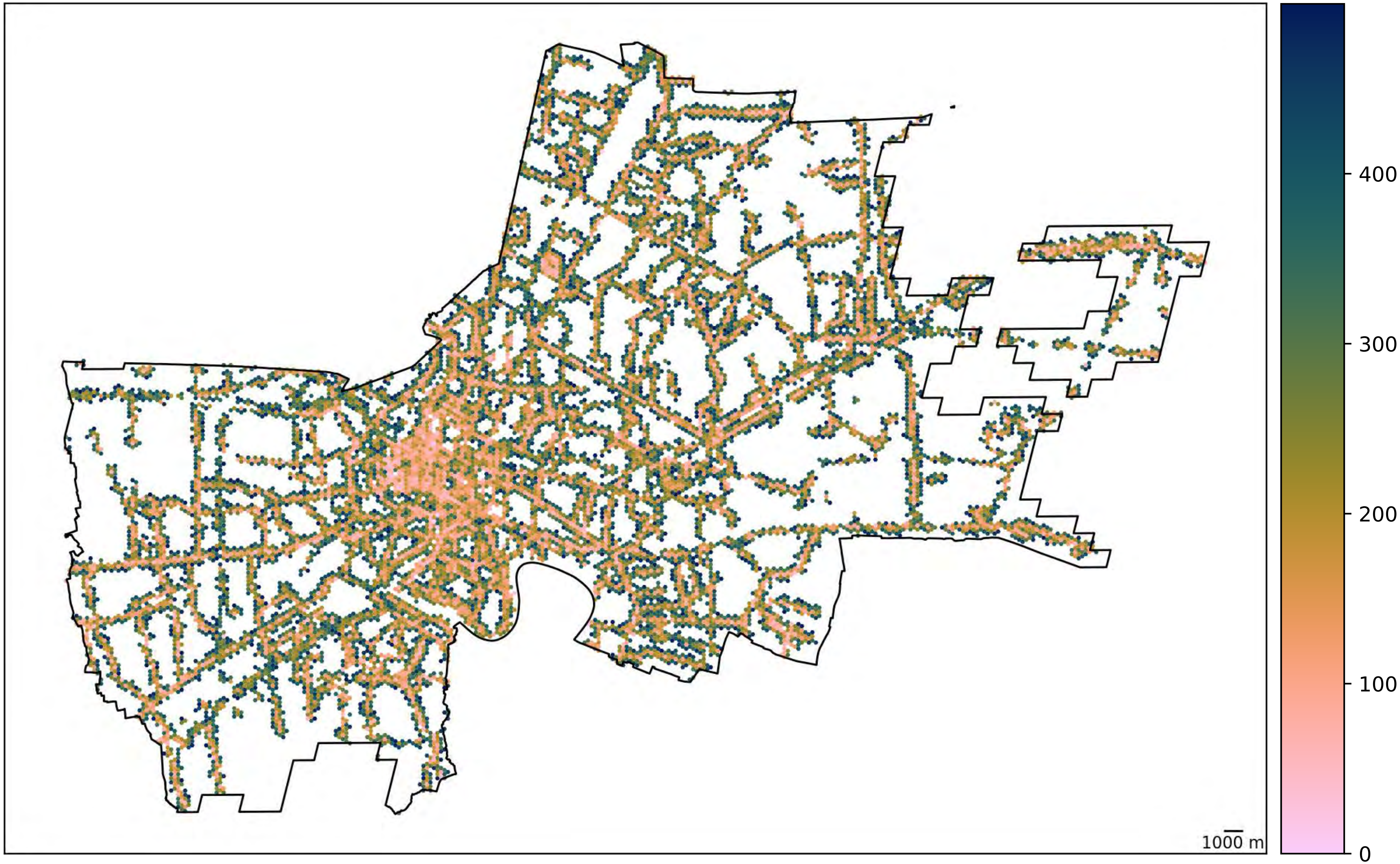

distances: Estimated Distance to nearest public transport stops (m; up to 500m) requirement for distances to destinations, measured up to a maximum distance target threshold of 500 metres

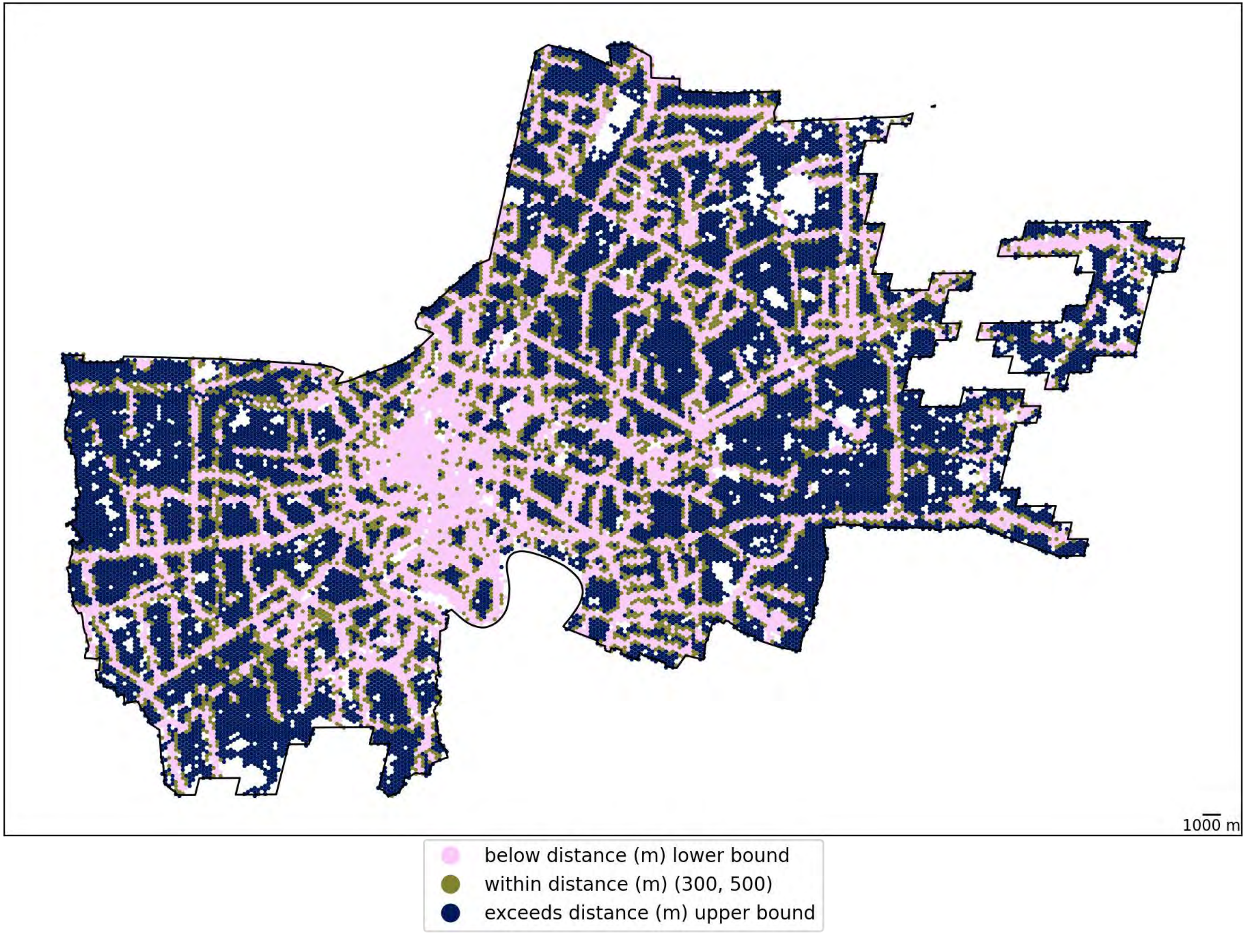

Histogram of Distance to nearest public transport stops (m; up to 500m).

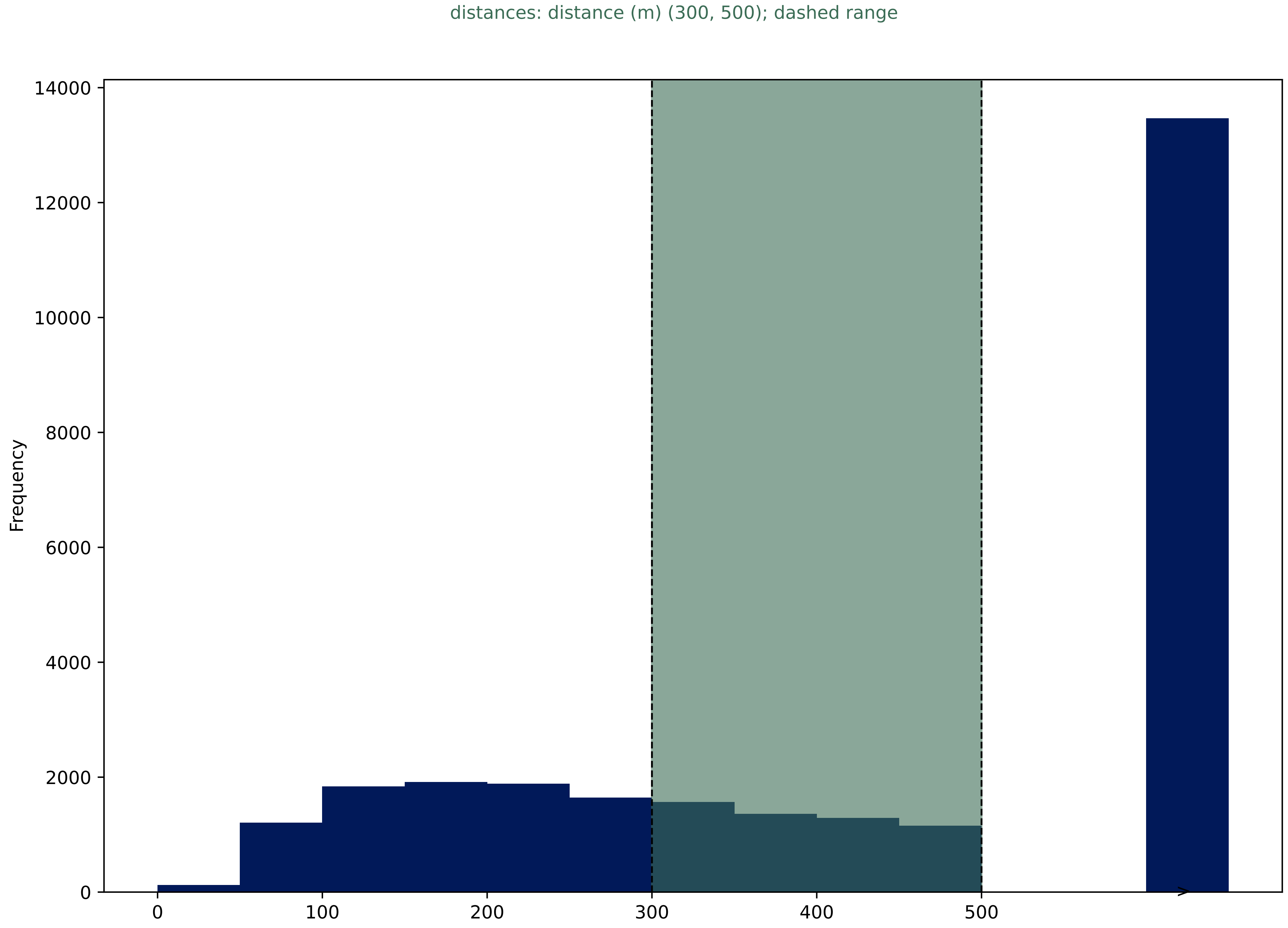

Distance to nearest park (m; up to 500m)

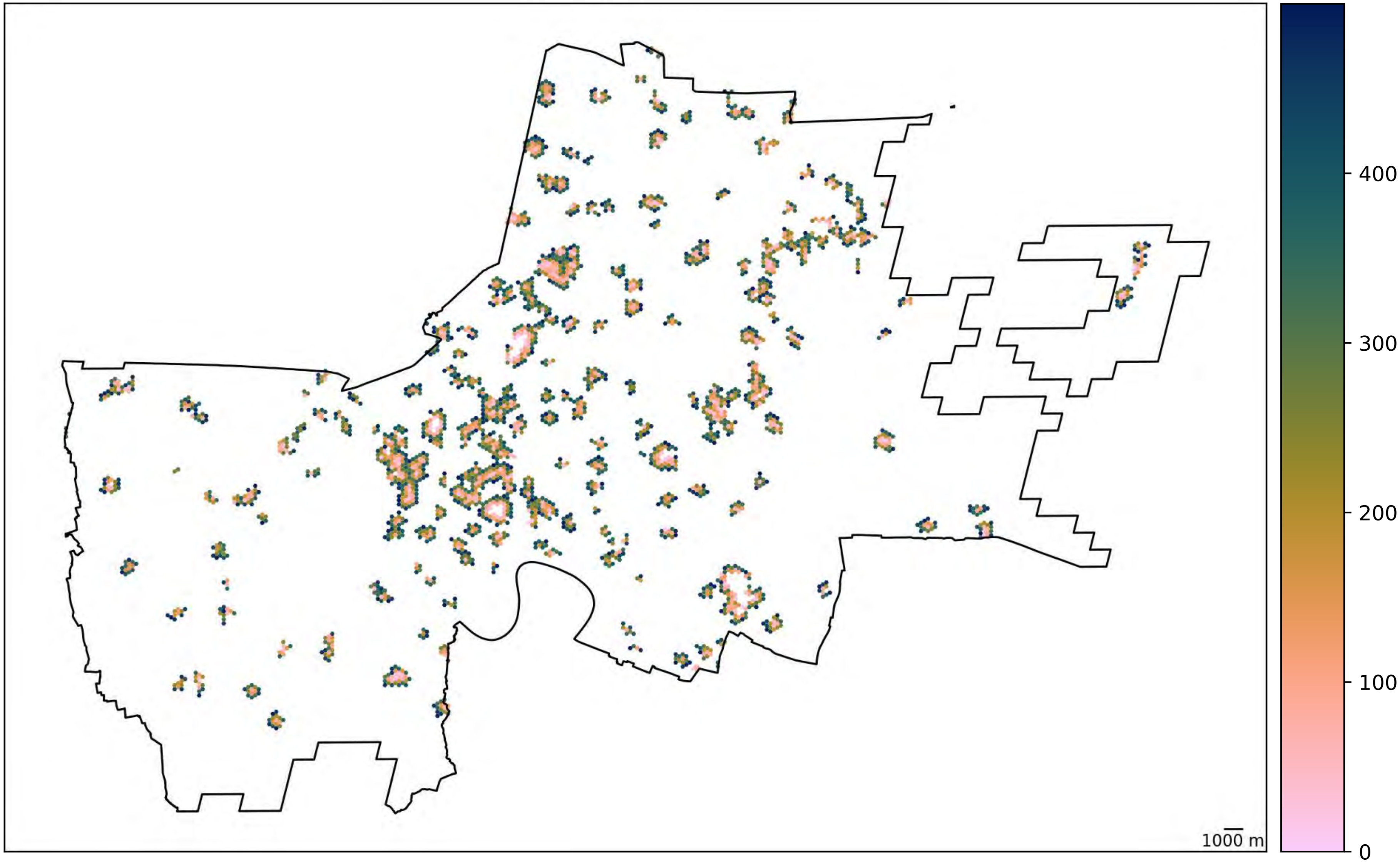

distances: Estimated Distance to nearest park (m; up to 500m) requirement for distances to destinations, measured up to a maximum distance target threshold of 500 metres

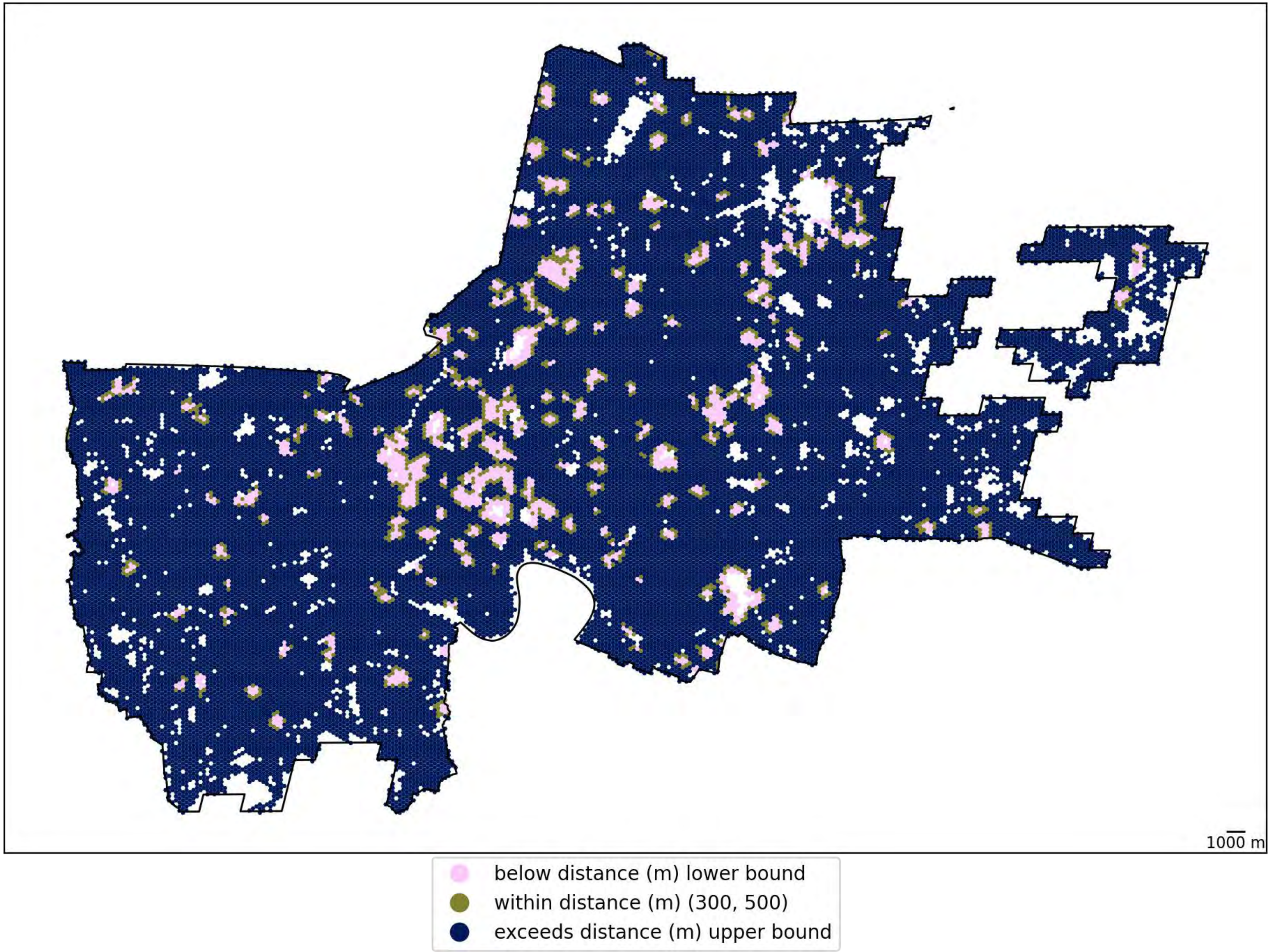

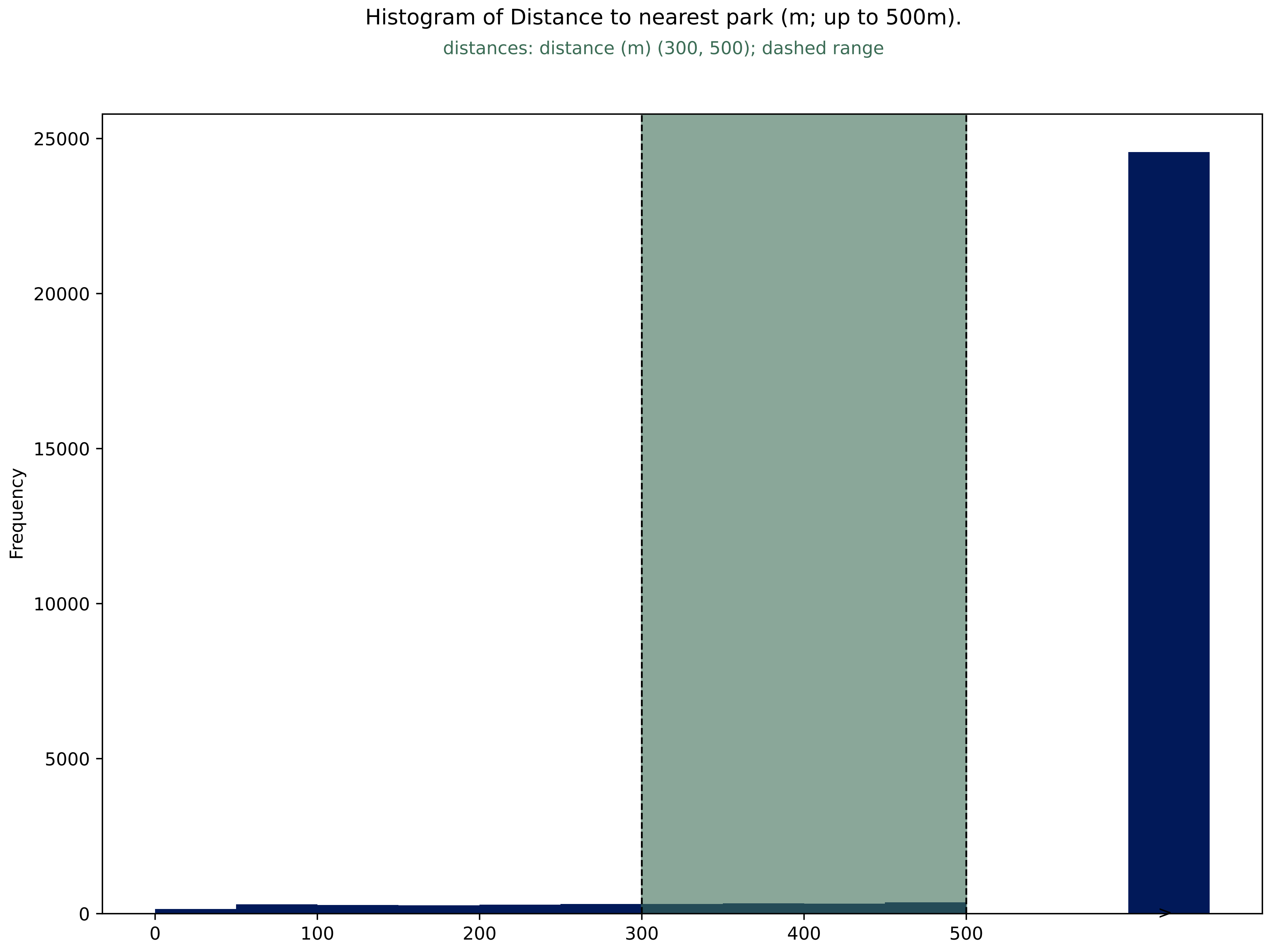
Histogram of Distance to nearest park (m; up to 500m).
distances: distance (m) (300, 500); dashed range
Frequency
25000
20000
15000
10000
5000
0
0
100
200
300
400
500
>

Asia, Vietnam, Hanoi

Satellite imagery of urban study region (Bing)

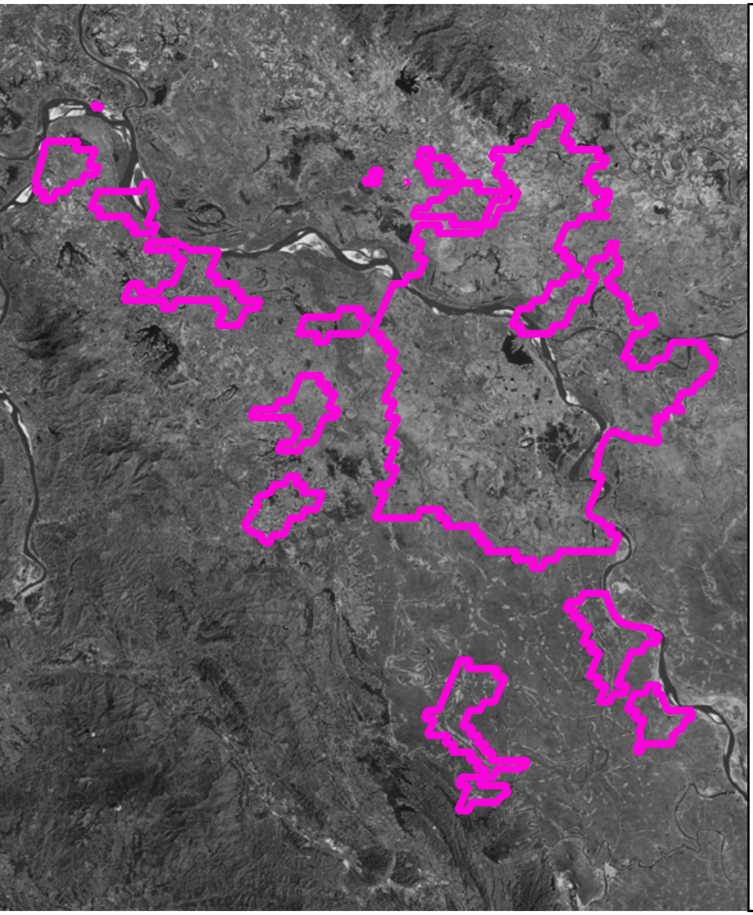

Walkability, relative to city

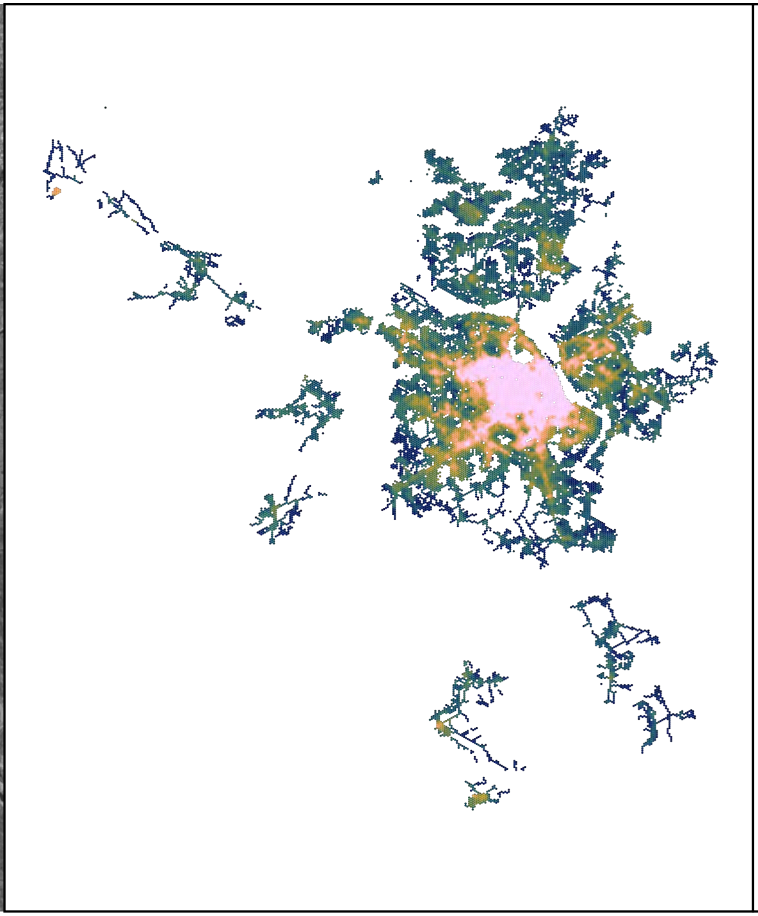

Walkability, relative to 25 global cities

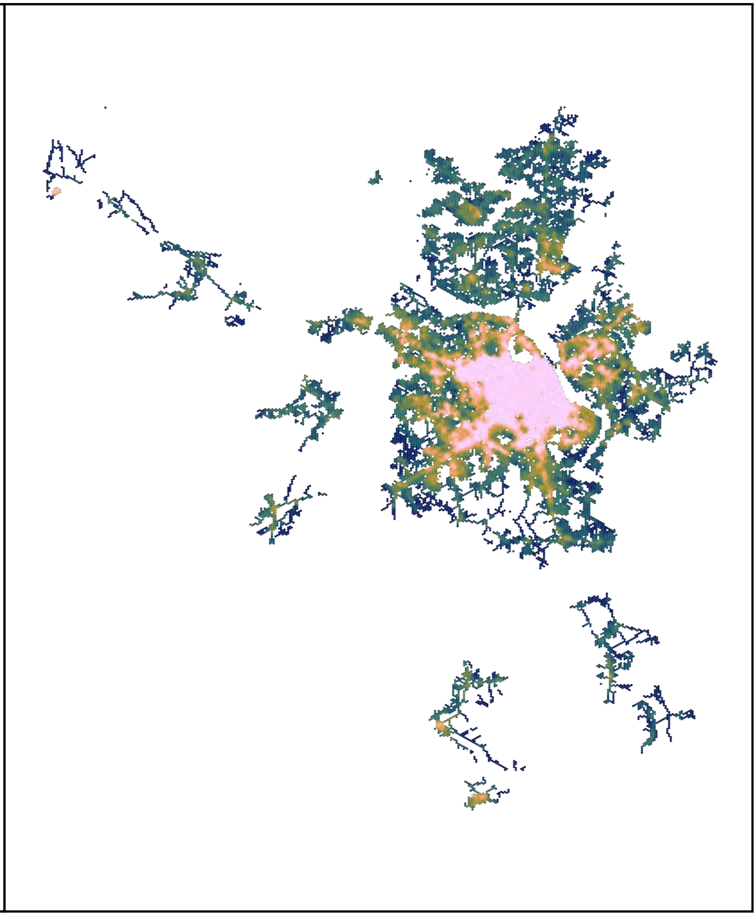

Urban boundary

0 30 60 km

Walkability score

- <-3
- -3 to -2
- -2 to -1
- -1 to 0
- 0 to 1
- 1 to 2
- 2 to 3
- ≥3

Walkability relative to all cities by component variables (2D histograms), and overall (histogram)

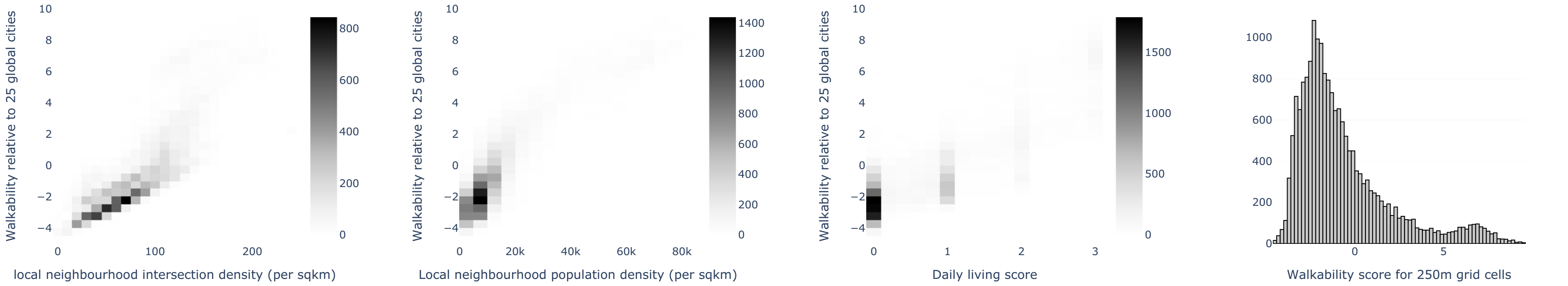

Mean 1000 m neighbourhood population per km²

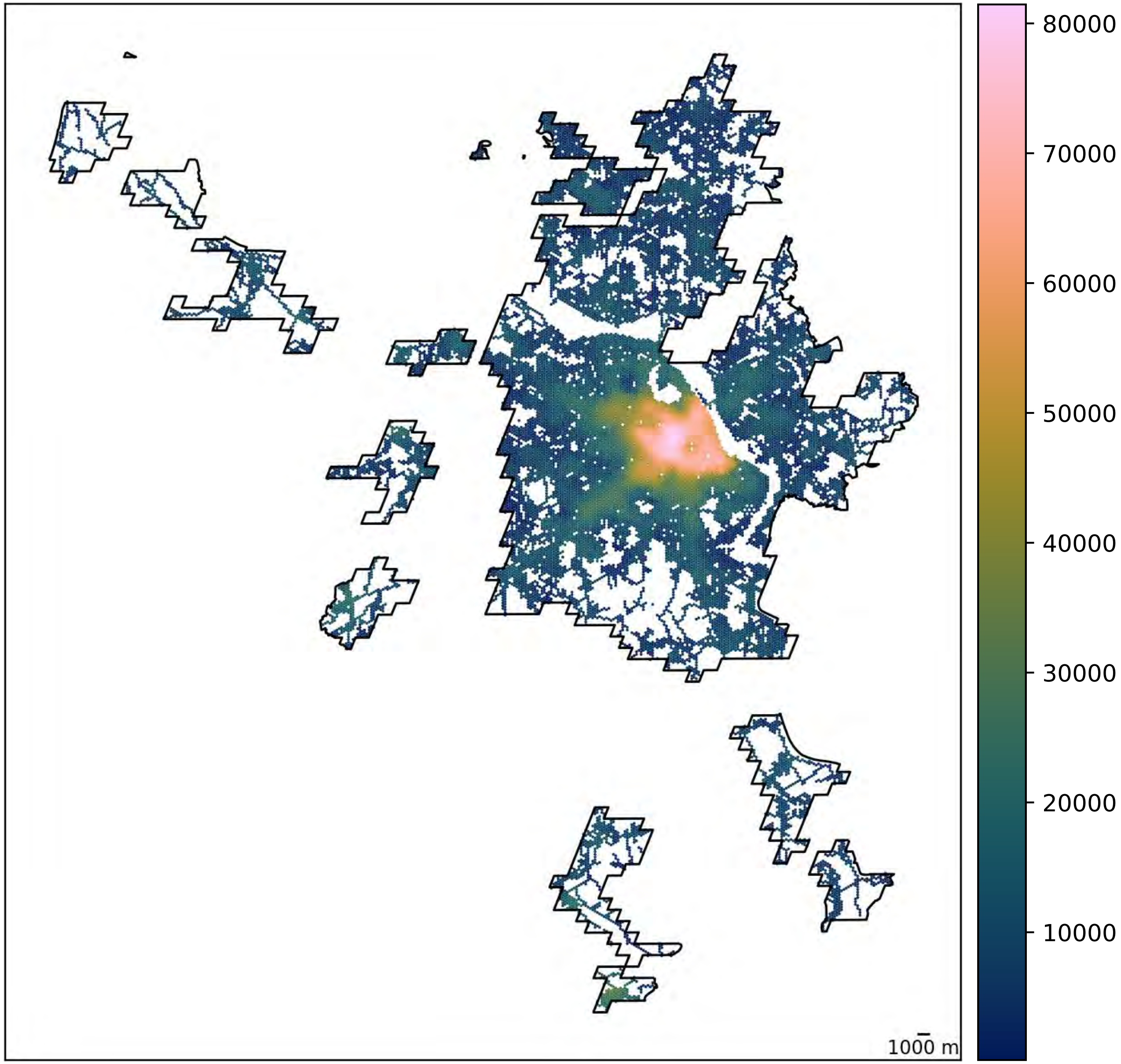

A: Estimated Mean 1000 m neighbourhood population per km² requirement for ≥80% probability of engaging in walking for transport

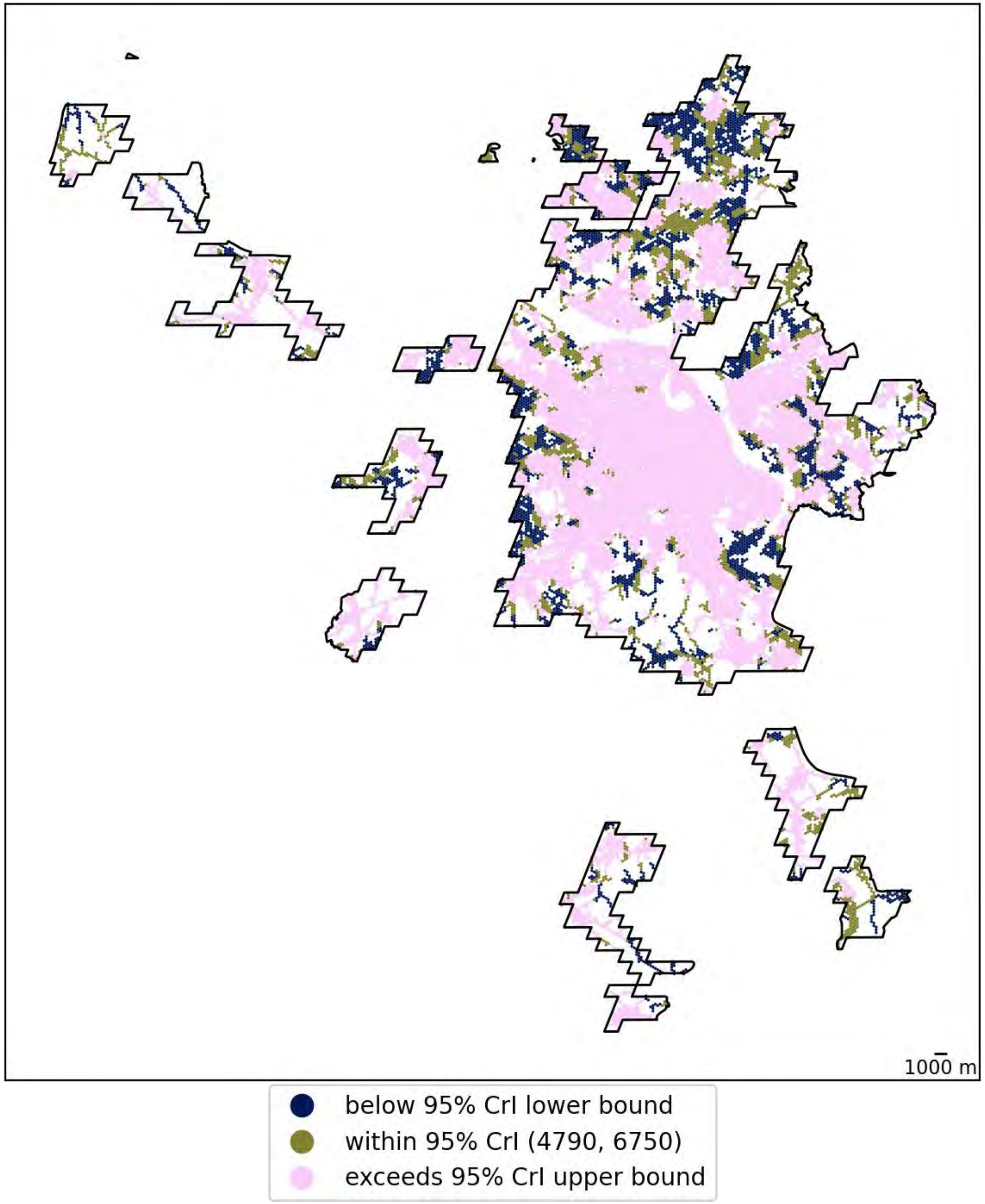

B: Estimated Mean 1000 m neighbourhood population per km² requirement for reaching the WHO's target of a ≥15% relative reduction in insufficient physical activity through walking

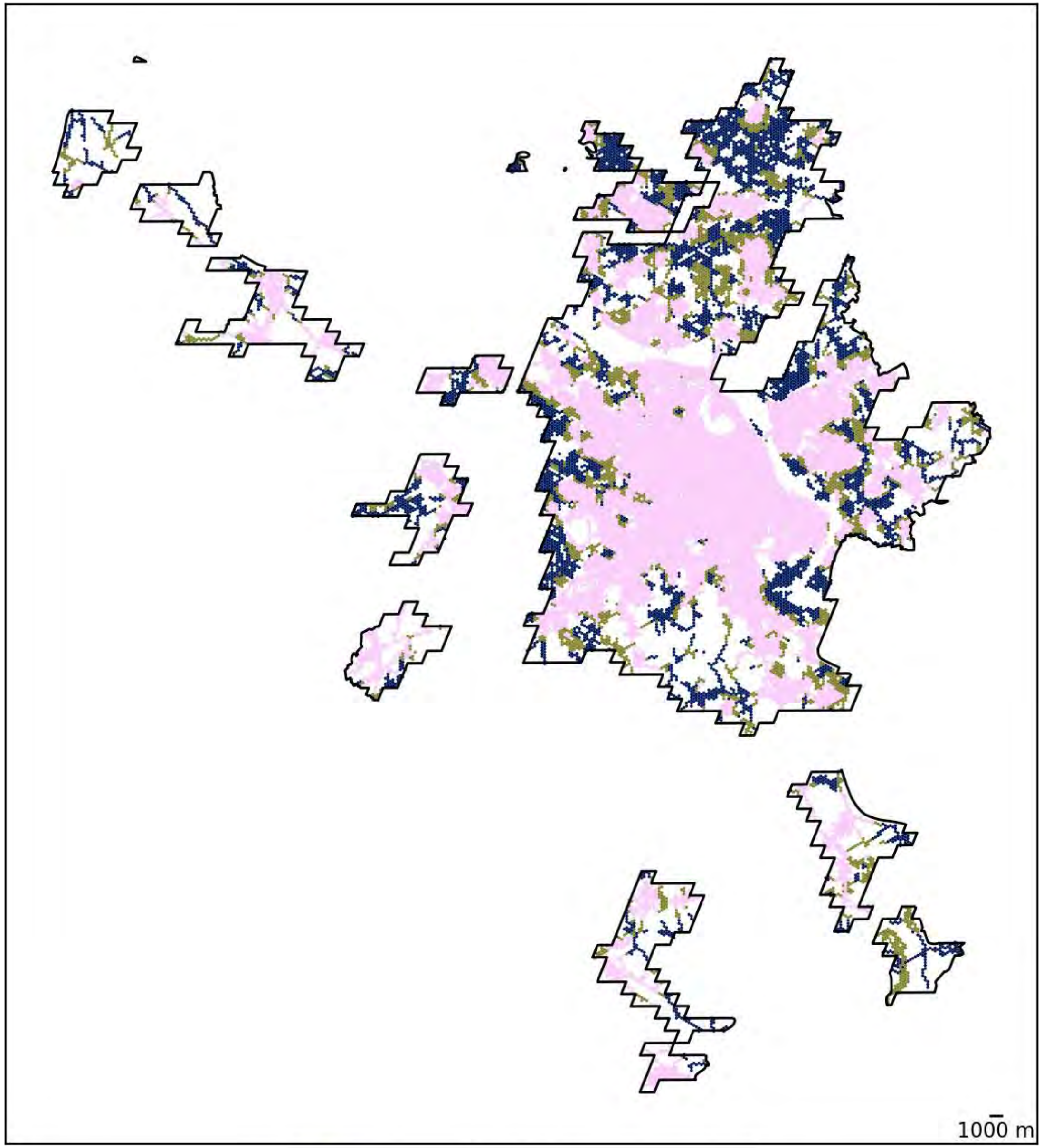

below 95% CrI lower bound
within 95% CrI (5677, 7823)
exceeds 95% CrI upper bound

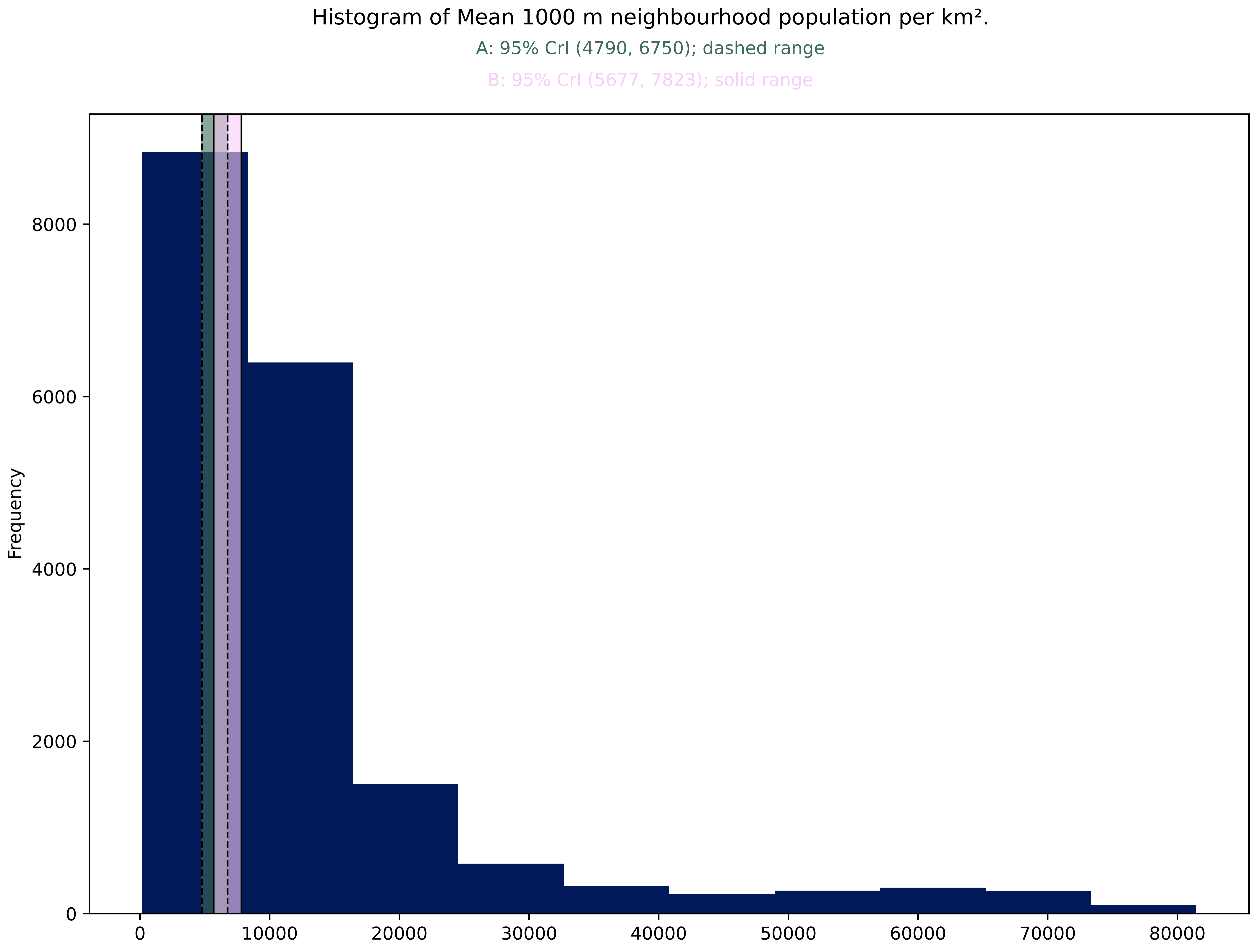

Histogram of Mean 1000 m neighbourhood population per km².
A: 95% CrI (4790, 6750); dashed range
B: 95% CrI (5677, 7823); solid range
Frequency
0
2000
4000
6000
8000
0
10000
20000
30000
40000
50000
60000
70000
80000

Mean 1000 m neighbourhood street intersections per km²

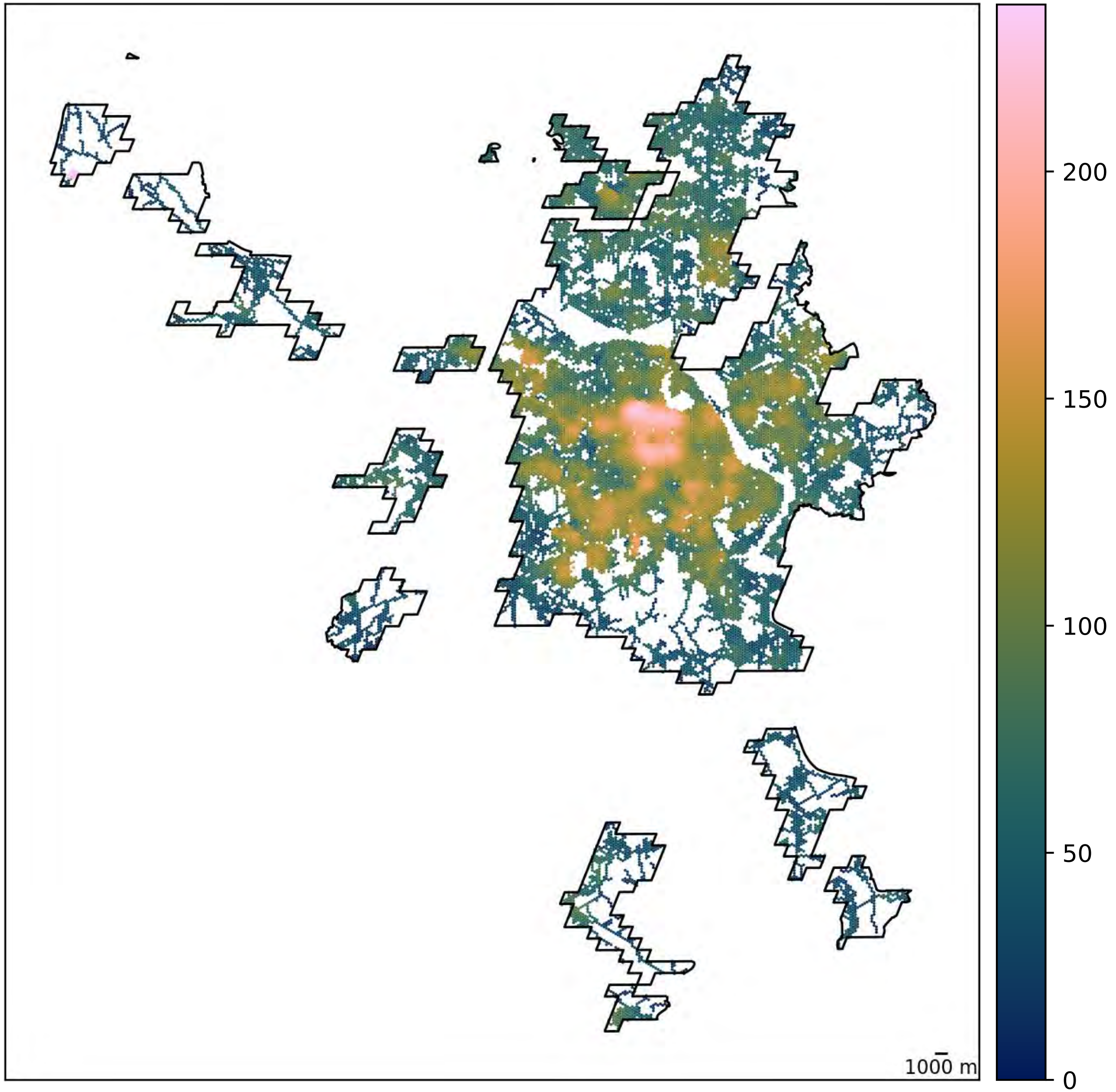

A: Estimated Mean 1000 m neighbourhood street intersections per km² requirement for ≥80% probability of engaging in walking for transport

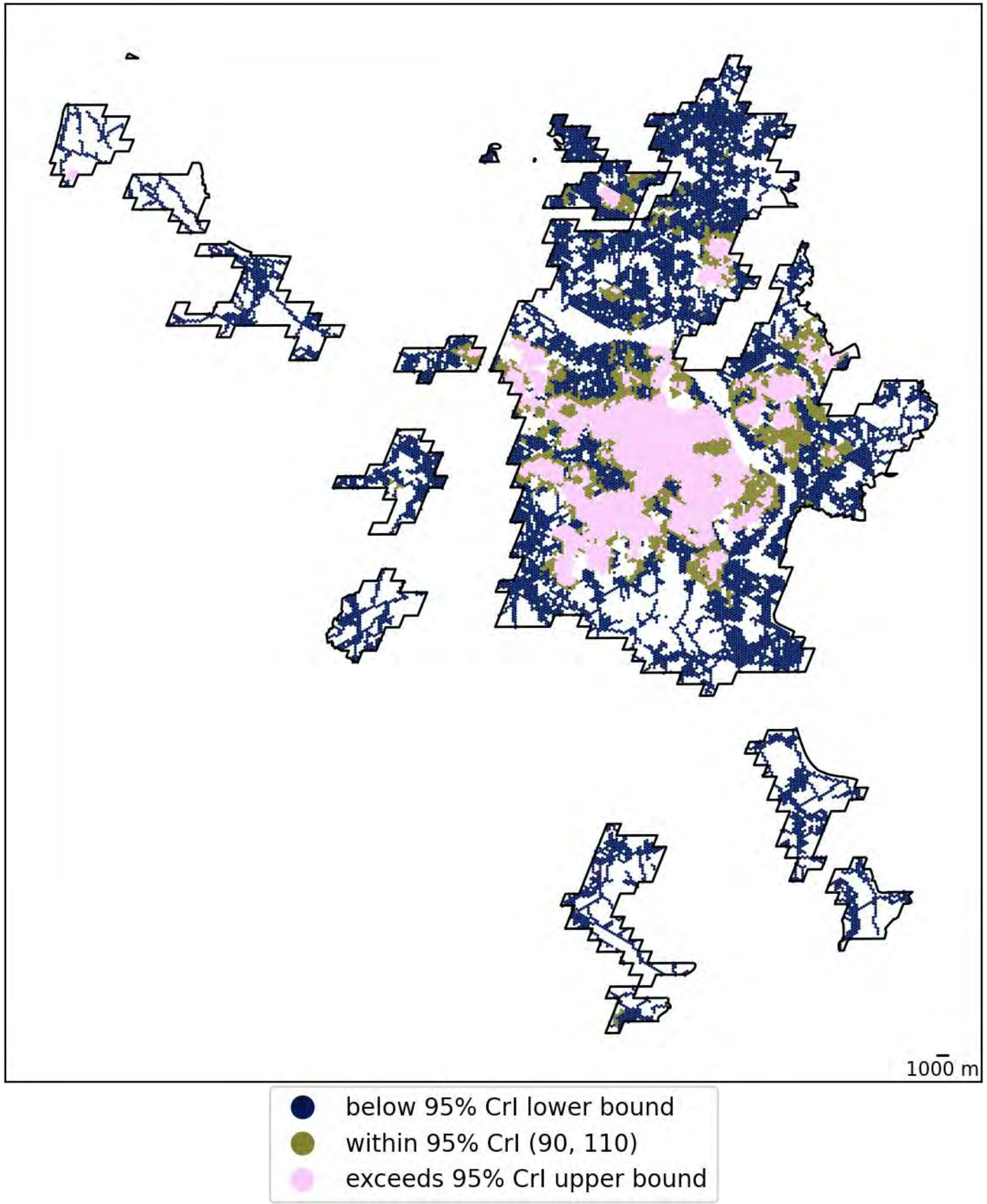

B: Estimated Mean 1000 m neighbourhood street intersections per km² requirement for reaching the WHO's target of a ≥15% relative reduction in insufficient physical activity through walking

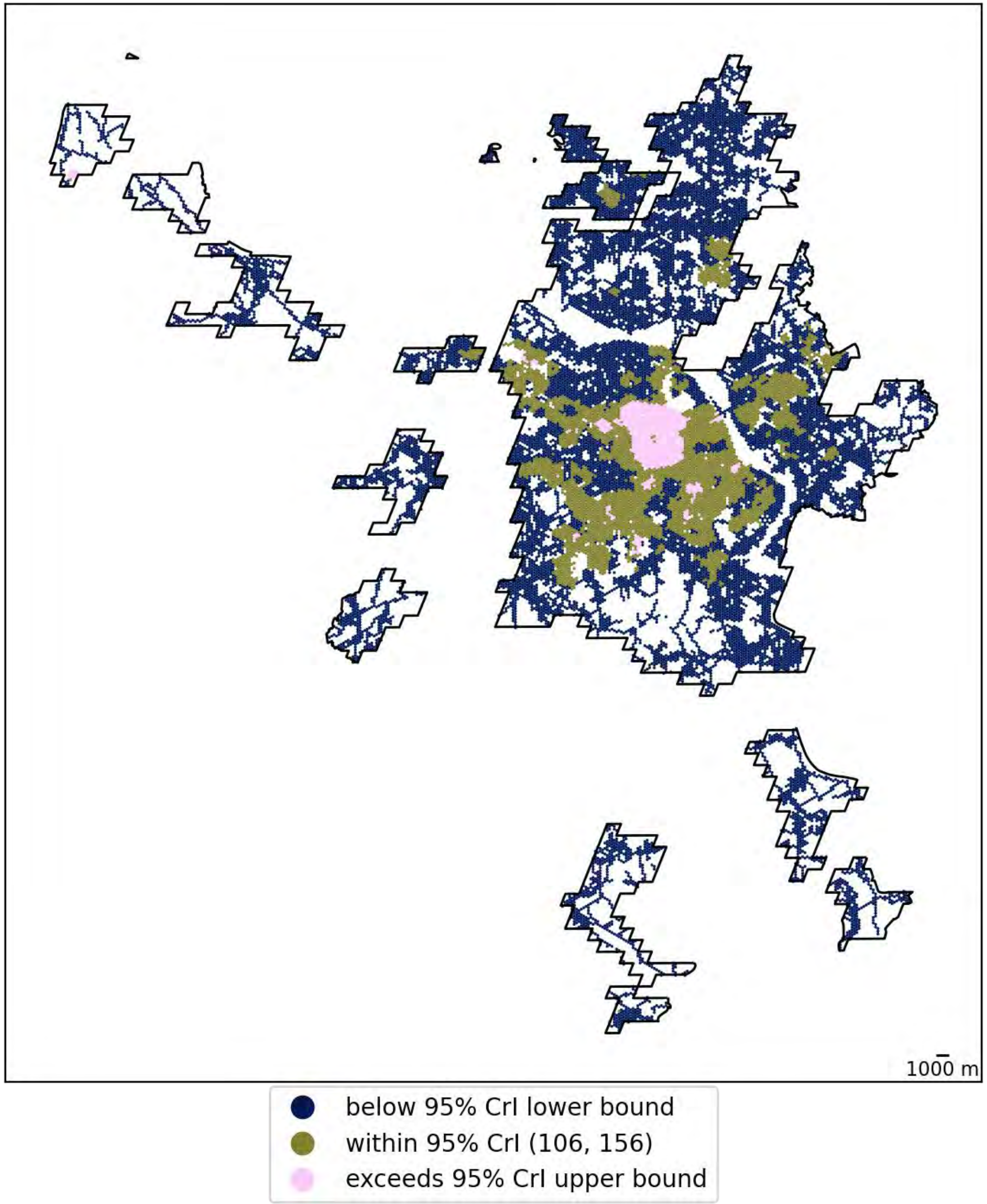

Histogram of Mean 1000 m neighbourhood street intersections per km².

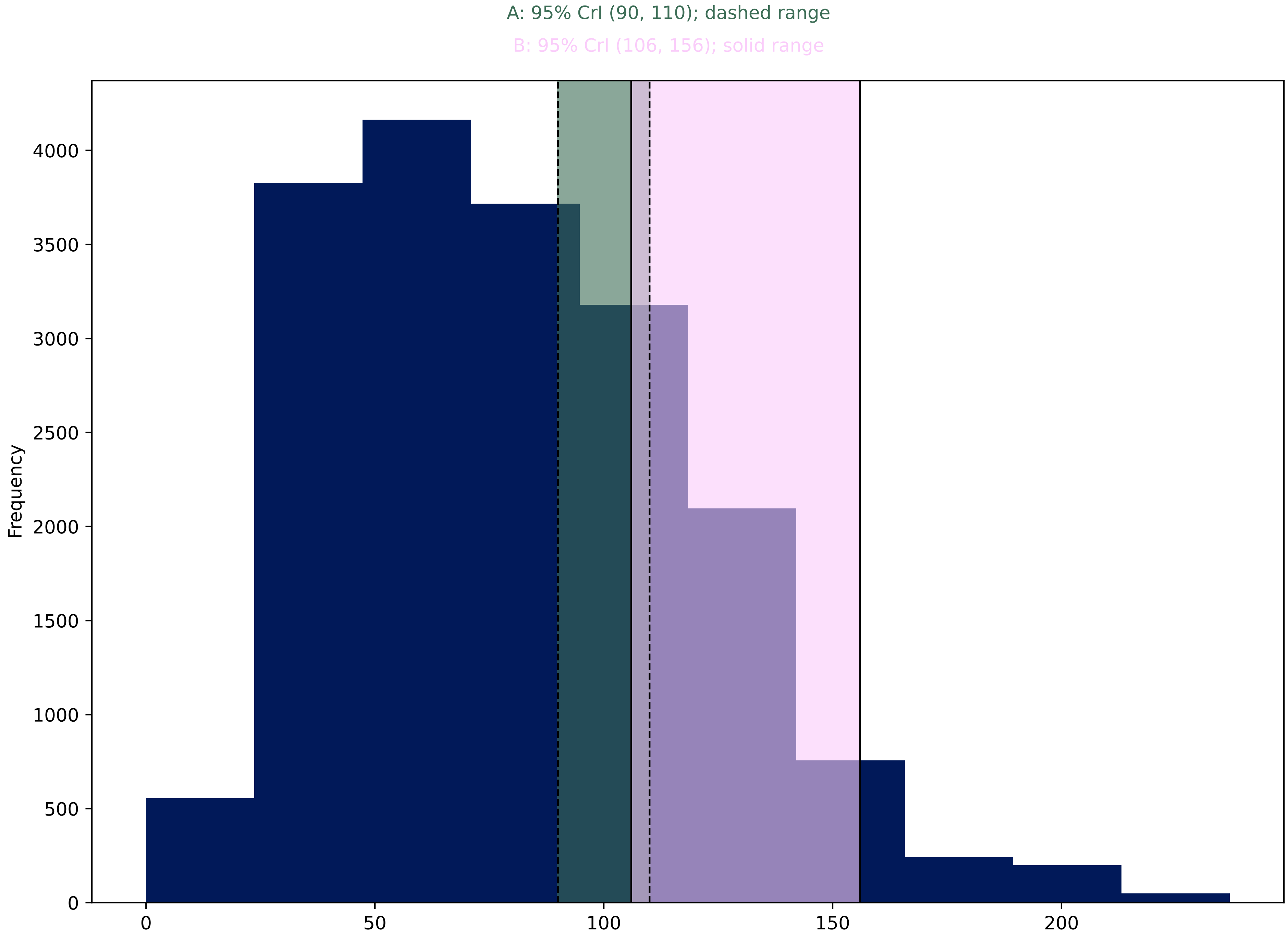

Distance to nearest public transport stops (m; up to 500m)

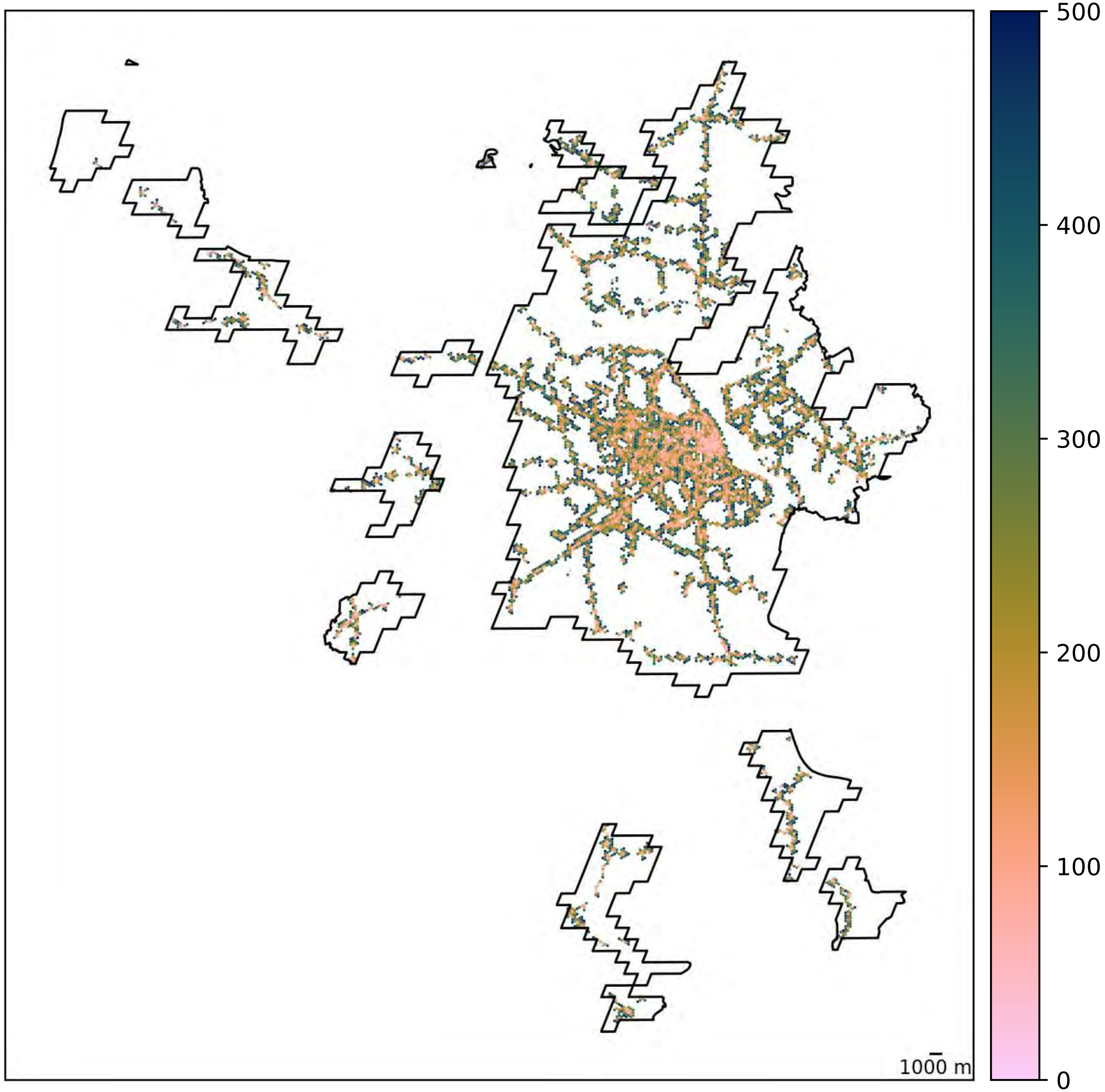

distances: Estimated Distance to nearest public transport stops (m; up to 500m) requirement for distances to destinations, measured up to a maximum distance target threshold of 500 metres

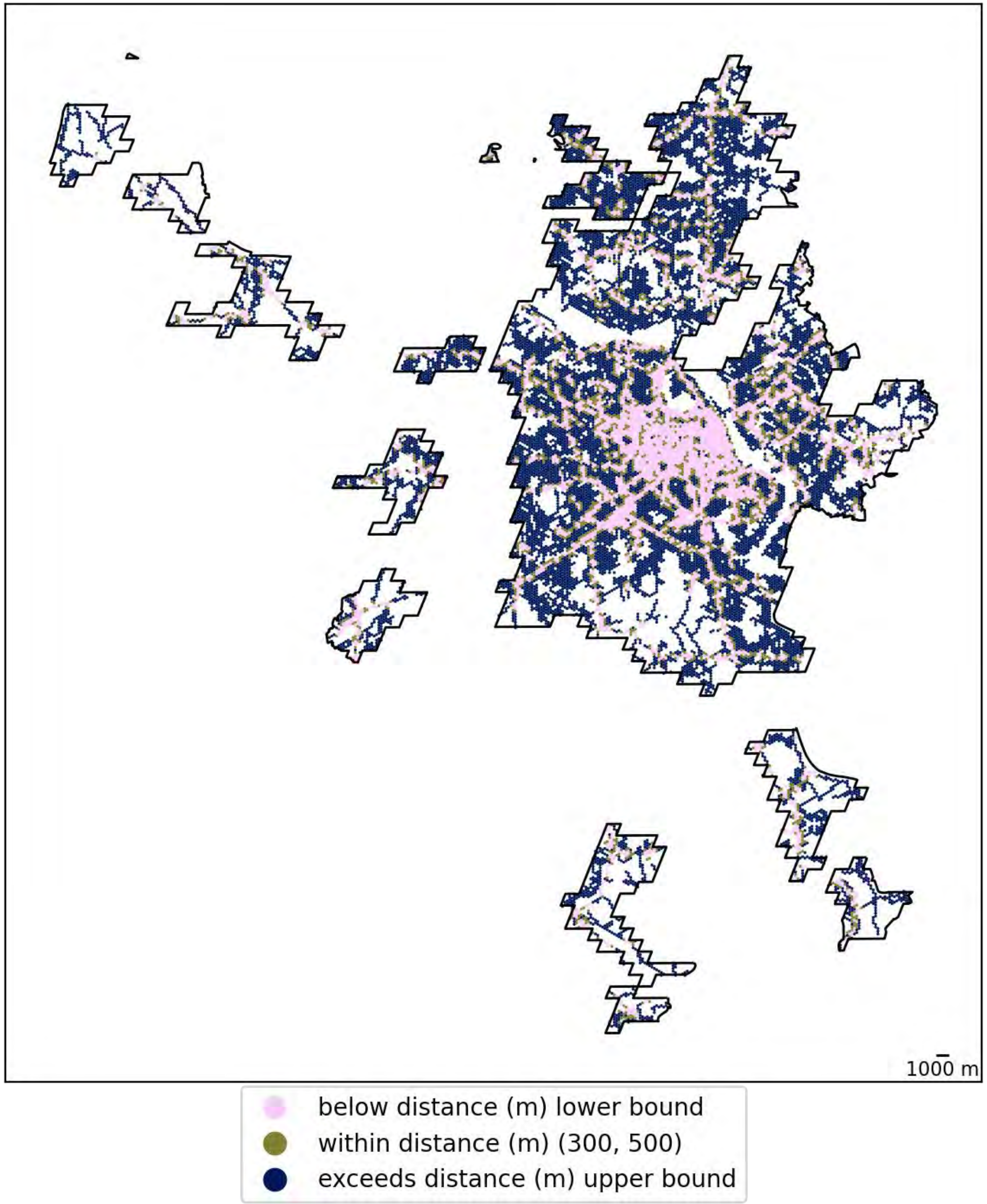

Histogram of Distance to nearest public transport stops (m; up to 500m).

distances: distance (m) (300, 500); dashed range

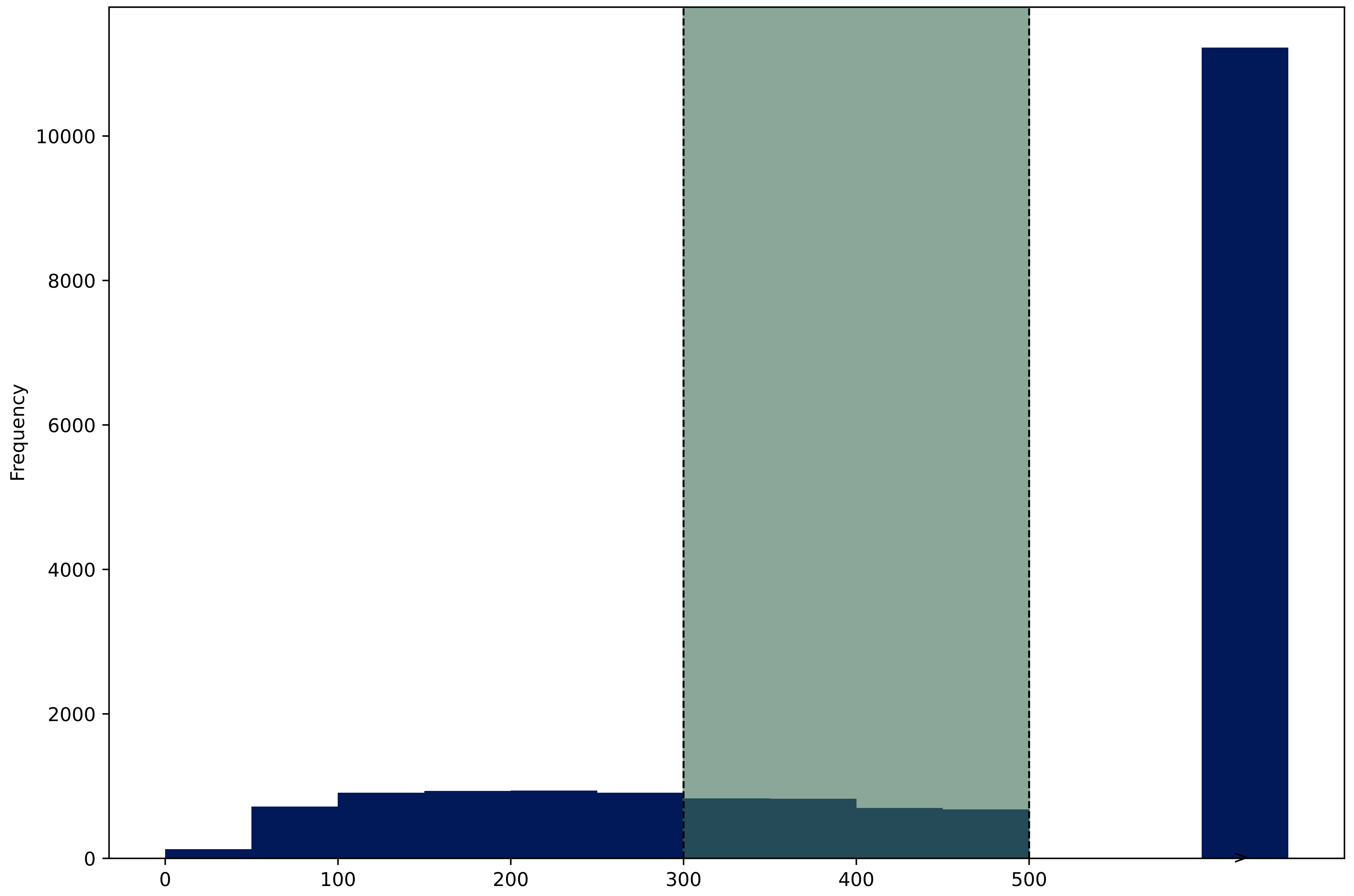

Distance to nearest park (m; up to 500m)

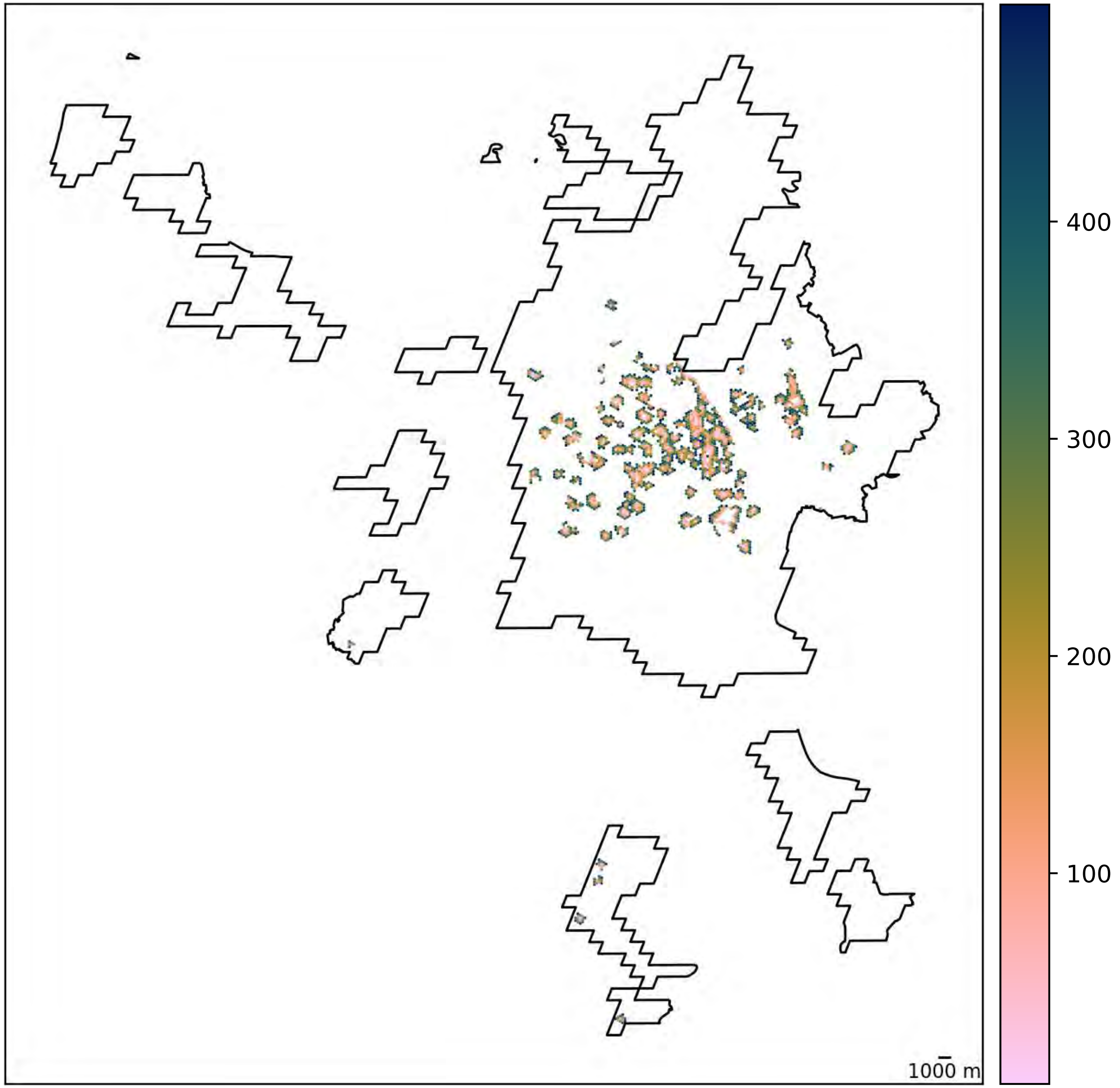

distances: Estimated Distance to nearest park (m; up to 500m) requirement for distances to destinations, measured up to a maximum distance target threshold of 500 metres

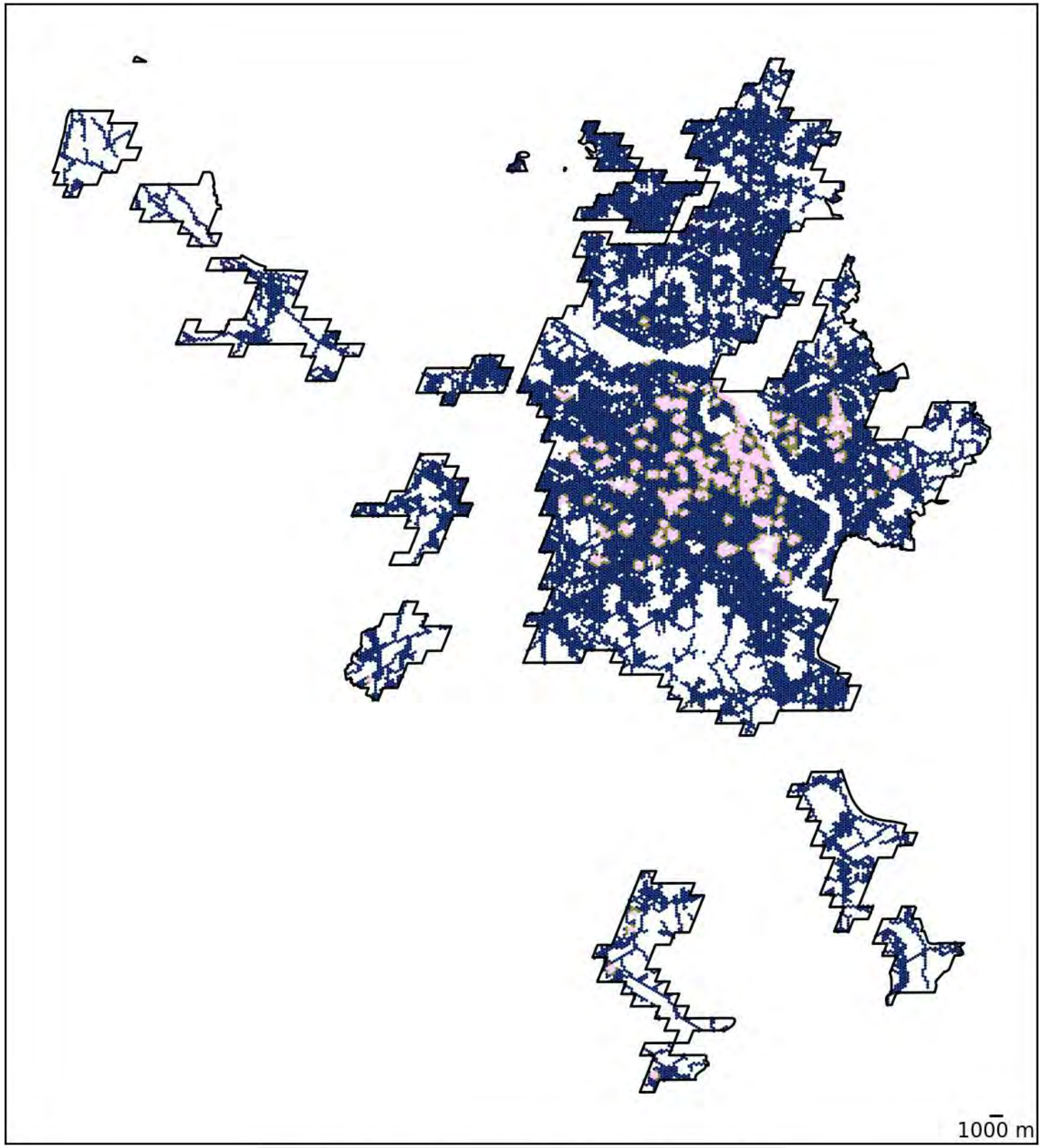

below distance (m) lower bound
within distance (m) (300, 500)
exceeds distance (m) upper bound

Histogram of Distance to nearest park (m; up to 500m).

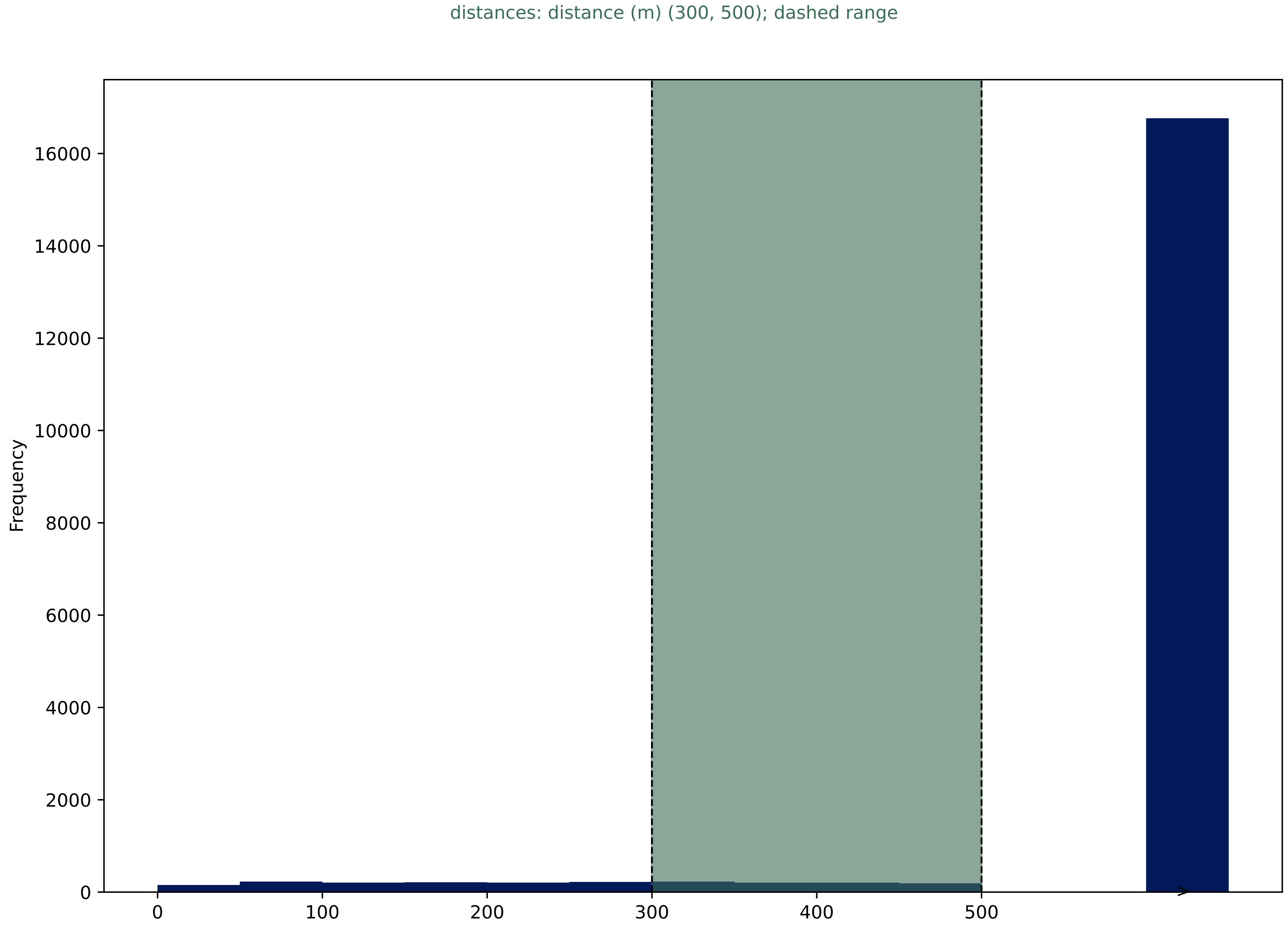

Australasia, Australia, Adelaide

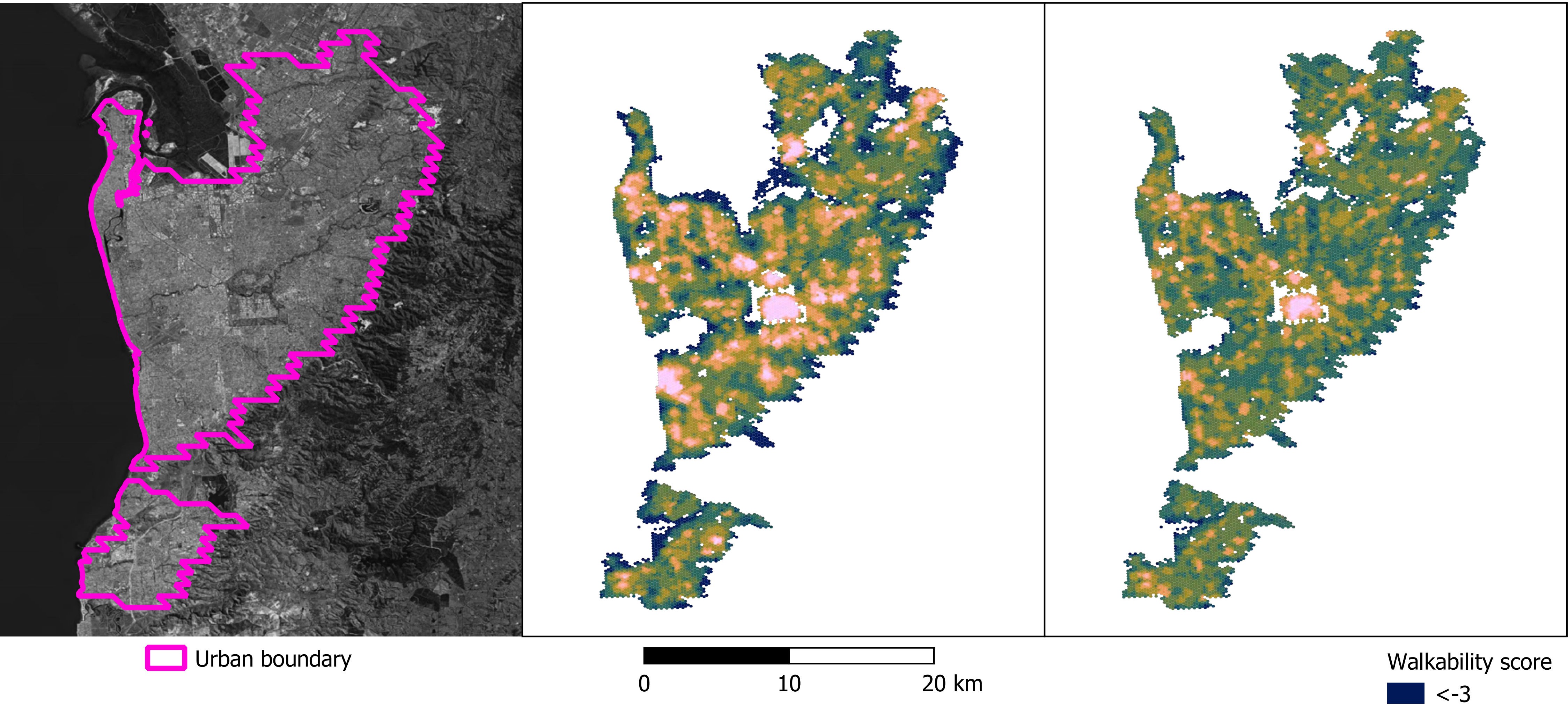

Walkability relative to all cities by component variables (2D histograms), and overall (histogram)

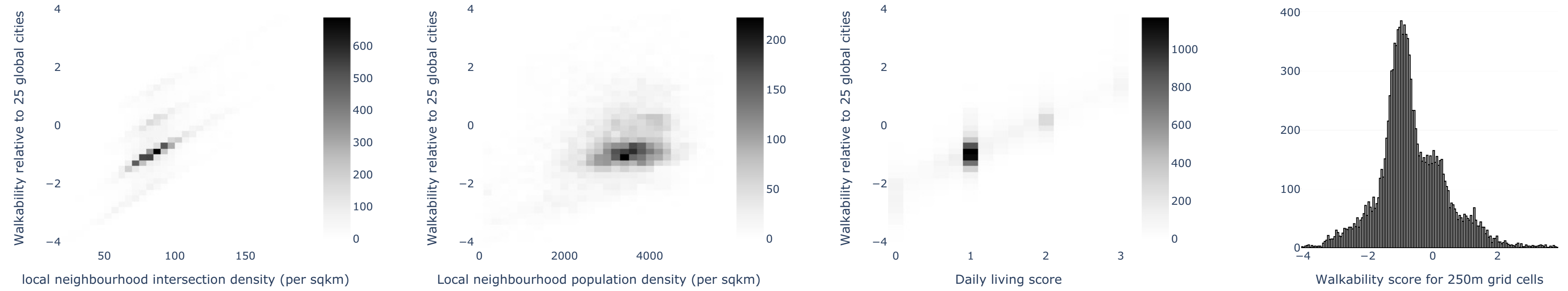

Mean 1000 m neighbourhood population per km²

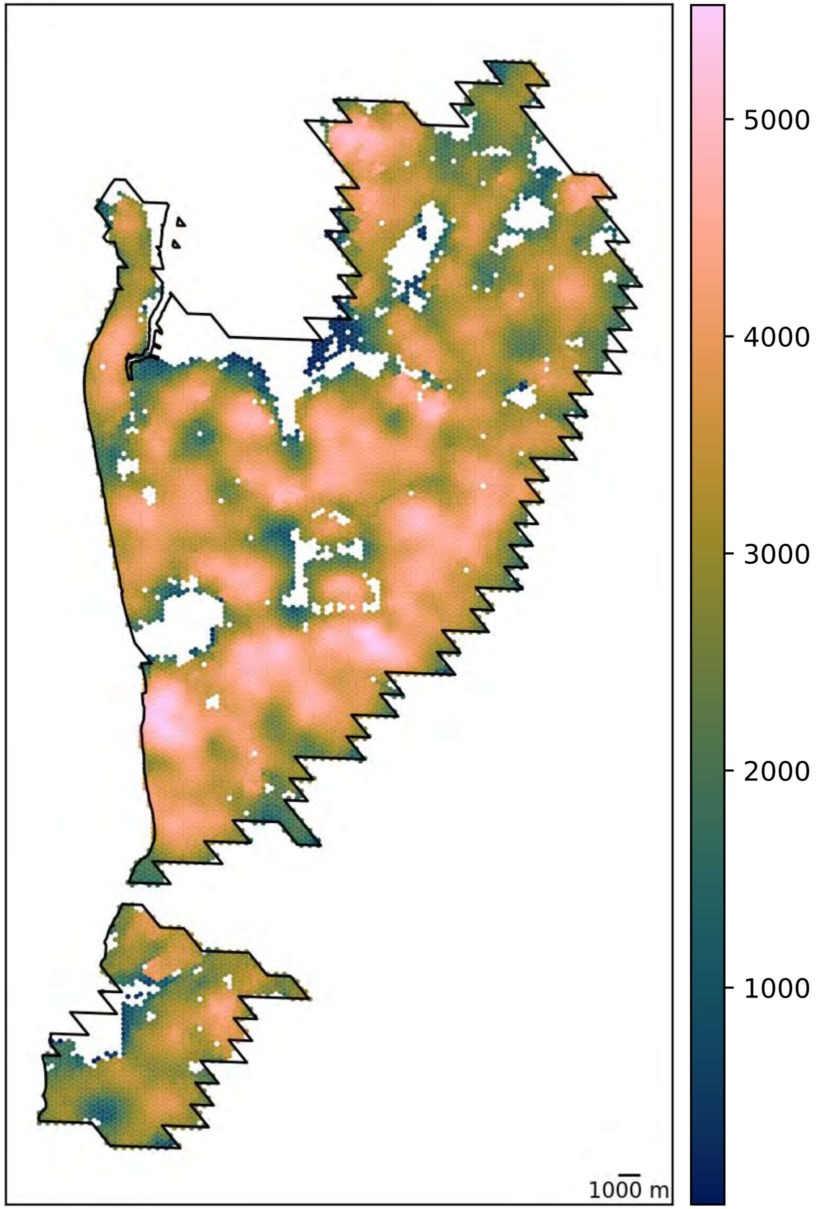

A: Estimated Mean 1000 m neighbourhood population per km² requirement for ≥80% probability of engaging in walking for transport

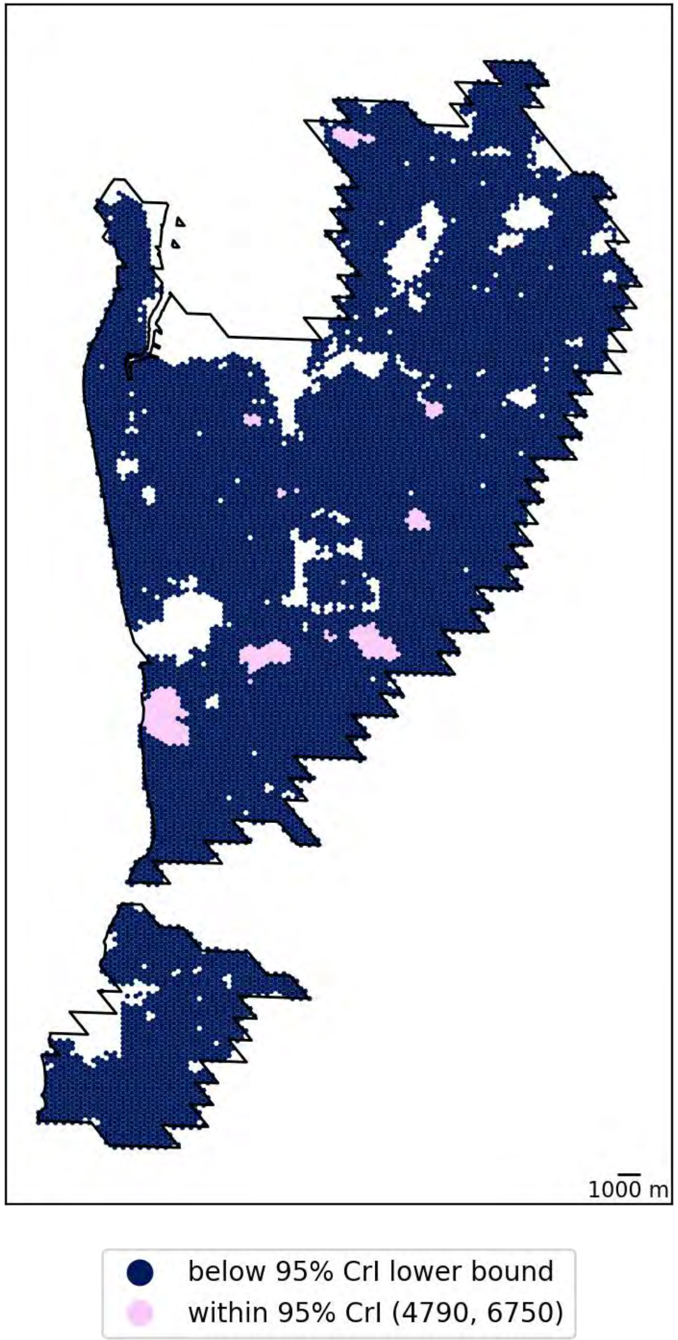

B: Estimated Mean 1000 m neighbourhood population per km² requirement for reaching the WHO's target of a ≥15% relative reduction in insufficient physical activity through walking

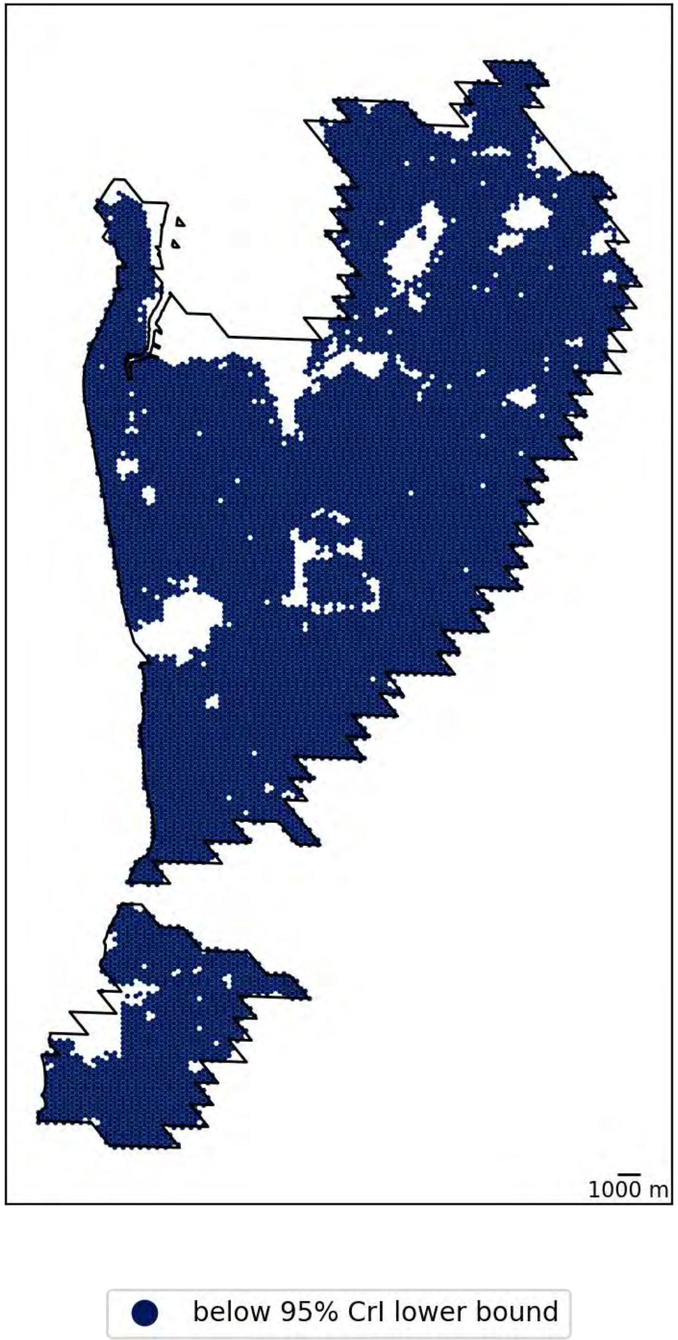

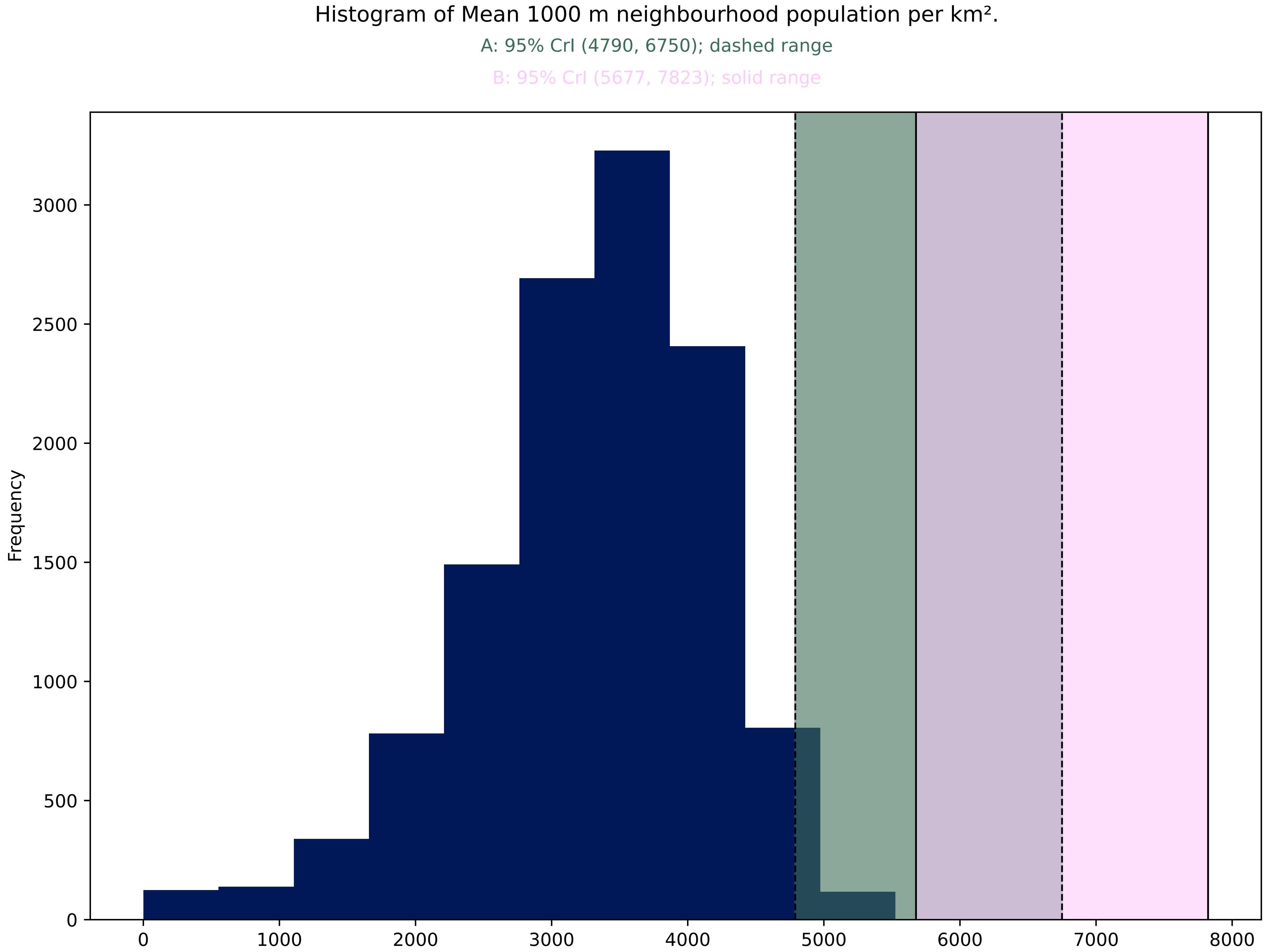
Histogram of Mean 1000 m neighbourhood population per km².
A: 95% CrI (4790, 6750); dashed range
B: 95% CrI (5677, 7823); solid range
Frequency
0
500
1000
1500
2000
2500
3000
0
1000
2000
3000
4000
5000
6000
7000
8000

Mean 1000 m neighbourhood street intersections per km²

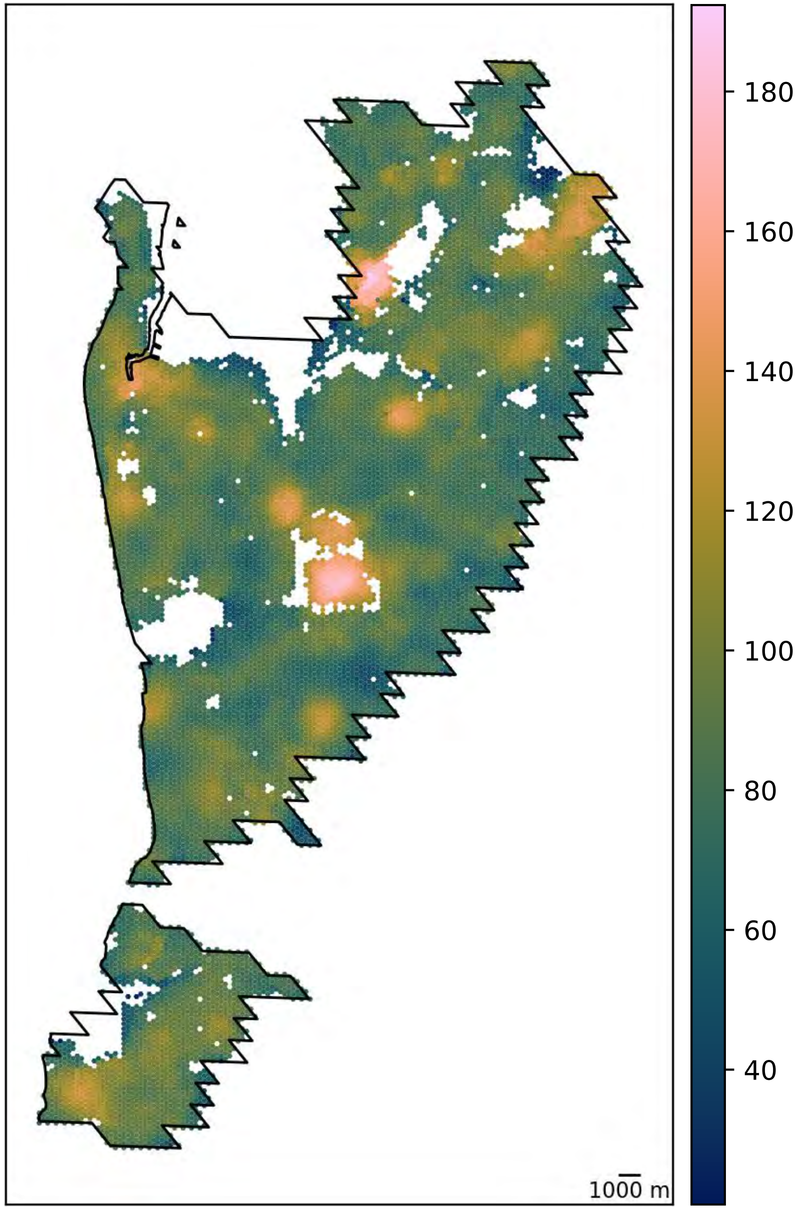

A: Estimated Mean 1000 m neighbourhood street intersections per km² requirement for ≥80% probability of engaging in walking for transport

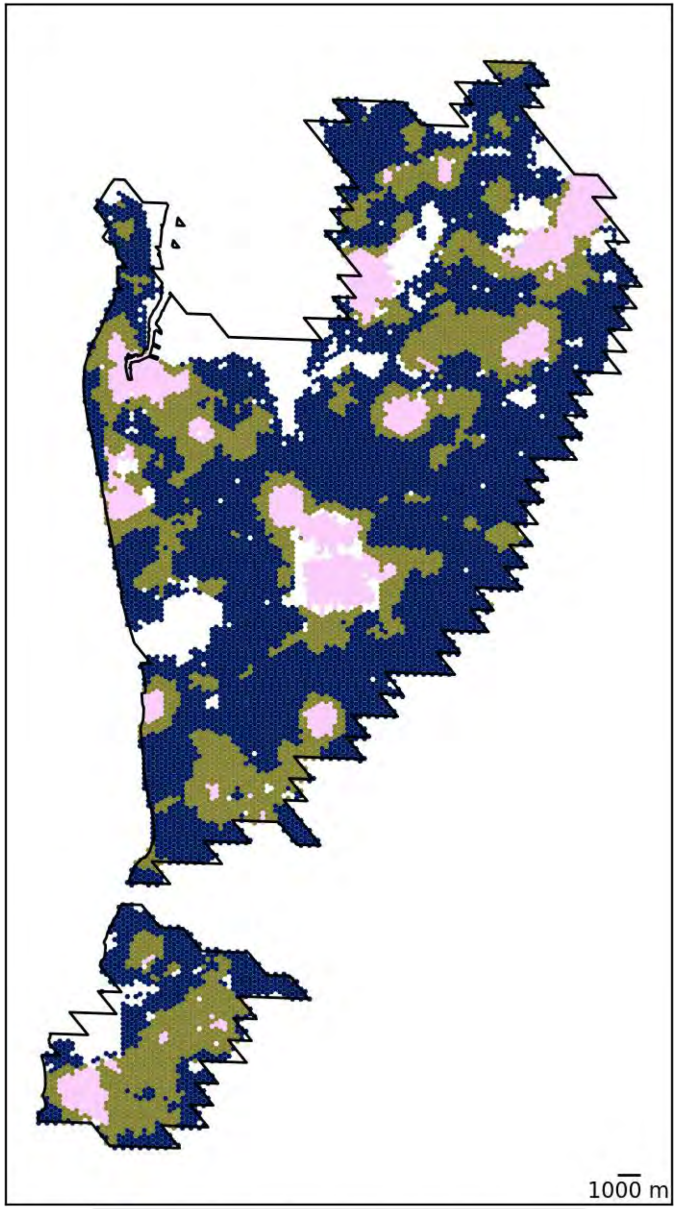

below 95% CrI lower bound
within 95% CrI (90, 110)
exceeds 95% CrI upper bound

B: Estimated Mean 1000 m neighbourhood street intersections per km² requirement for reaching the WHO's target of a ≥15% relative reduction in insufficient physical activity through walking

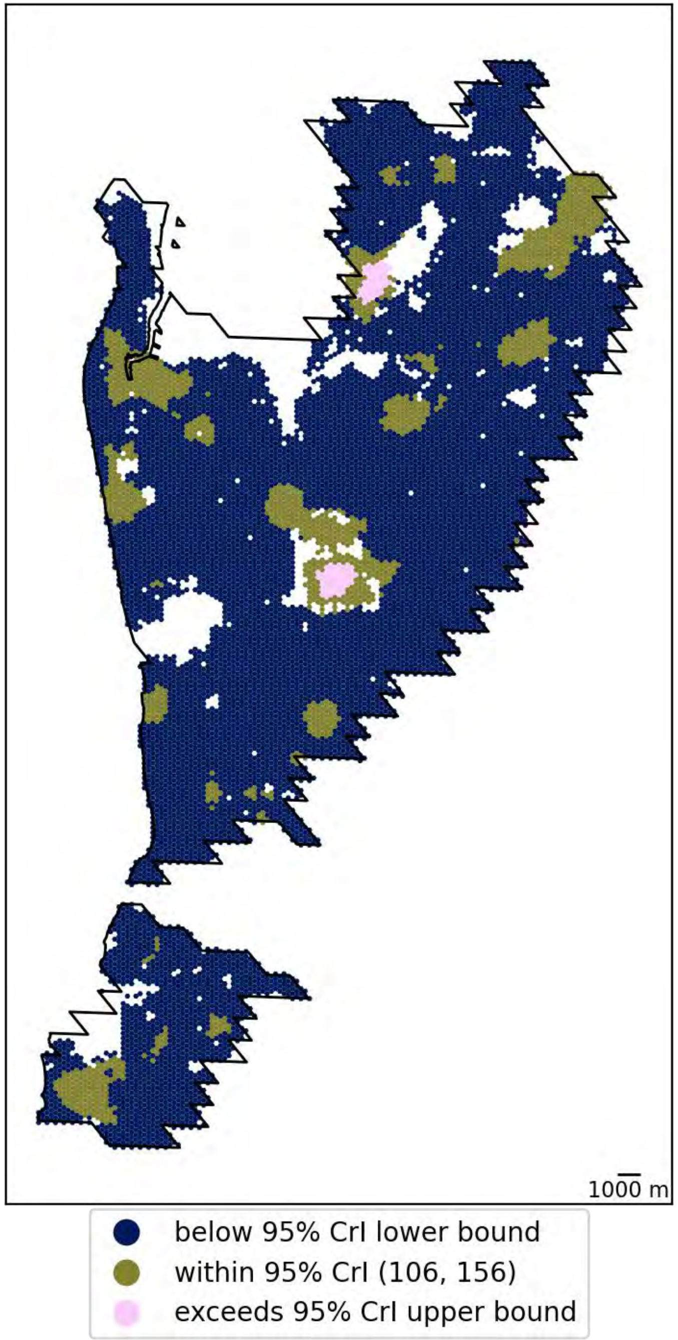

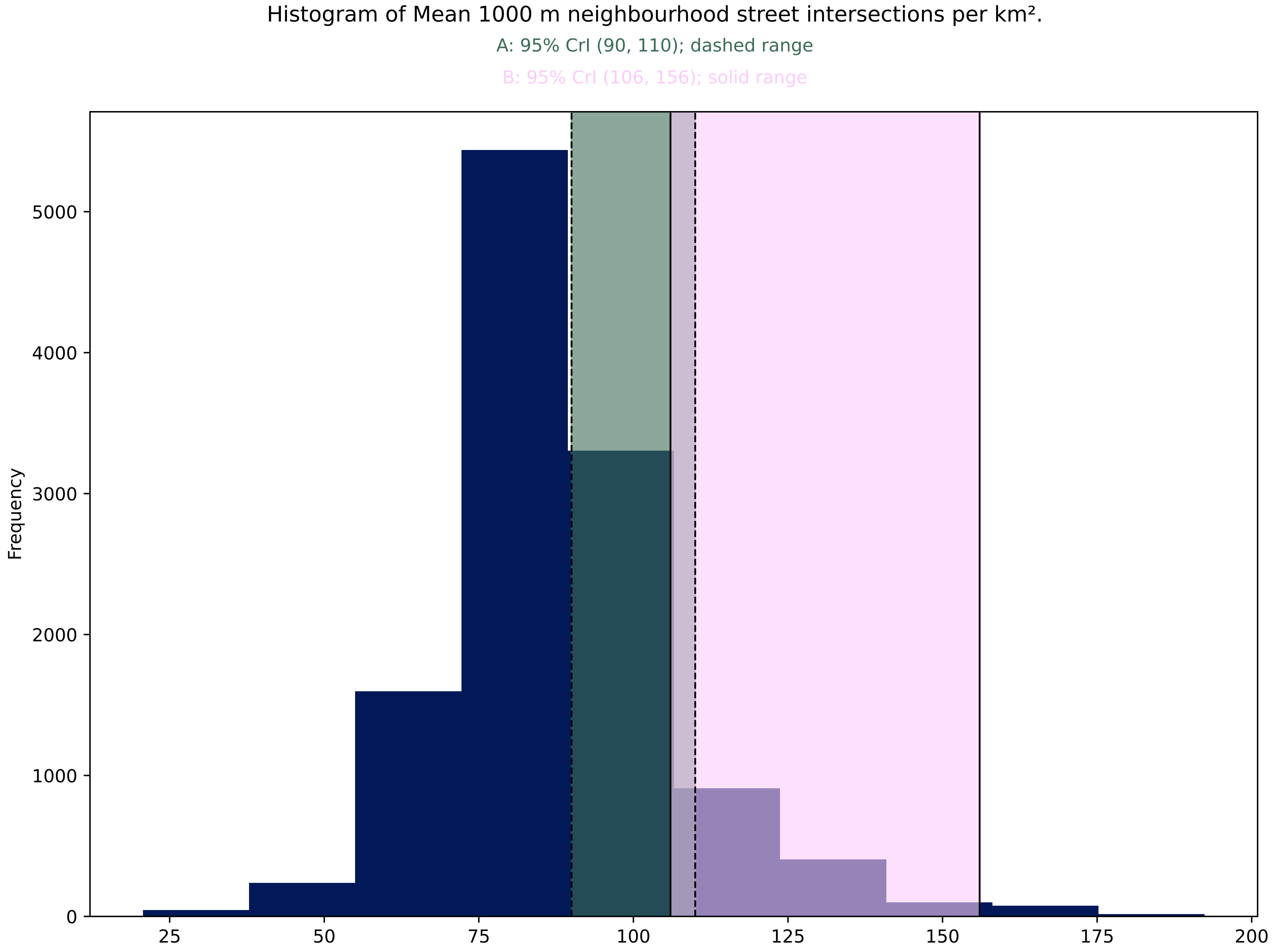
Histogram of Mean 1000 m neighbourhood street intersections per km².
A: 95% CrI (90, 110); dashed range
B: 95% CrI (106, 156); solid range
Frequency
0
1000
2000
3000
4000
5000
25
50
75
100
125
150
175
200

Distance to nearest public transport stops (m; up to 500m)

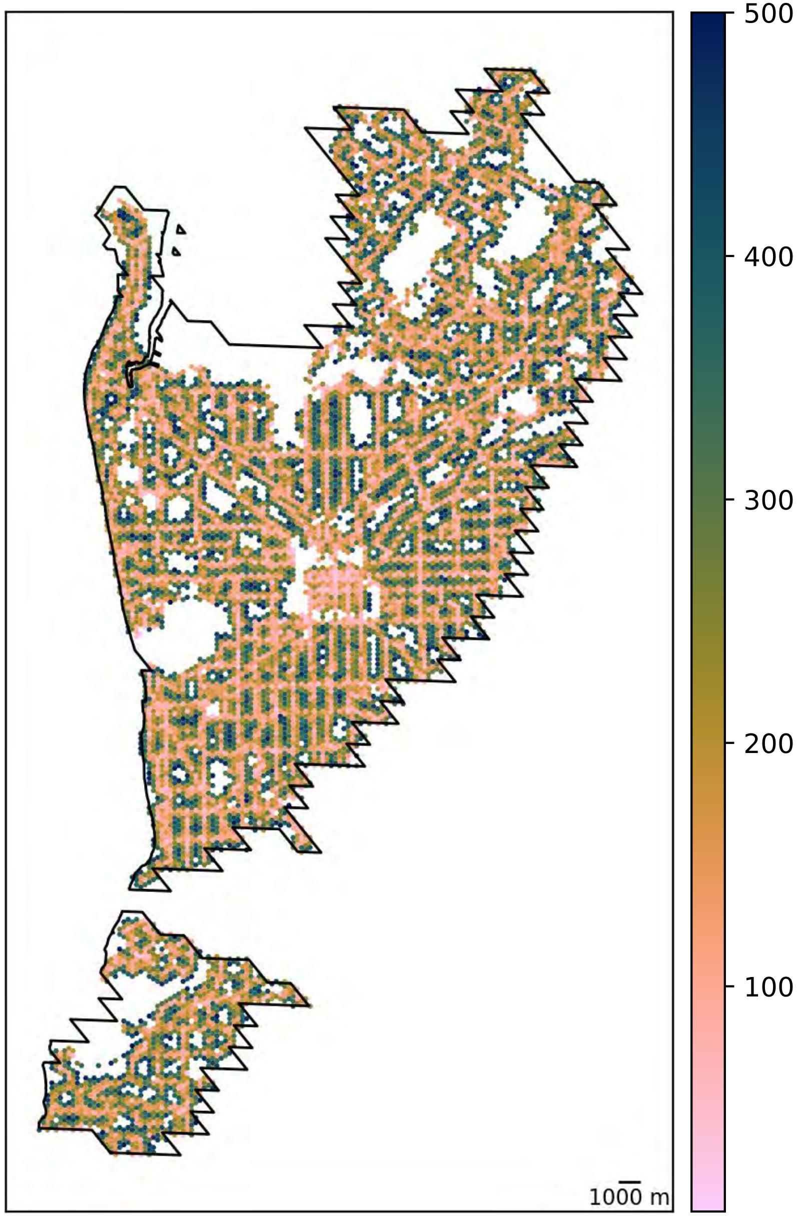

distances: Estimated Distance to nearest public transport stops (m; up to 500m) requirement for distances to destinations, measured up to a maximum distance target threshold of 500 metres

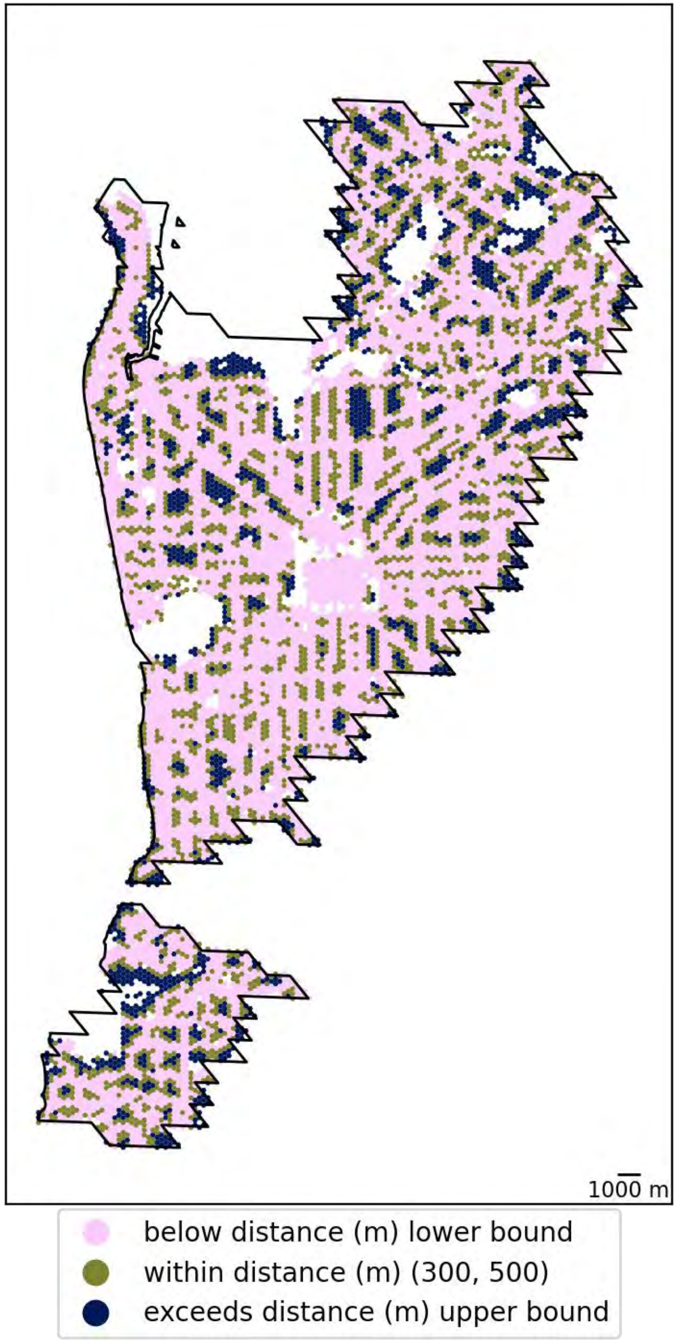

Histogram of Distance to nearest public transport stops (m; up to 500m).

distances: distance (m) (300, 500); dashed range

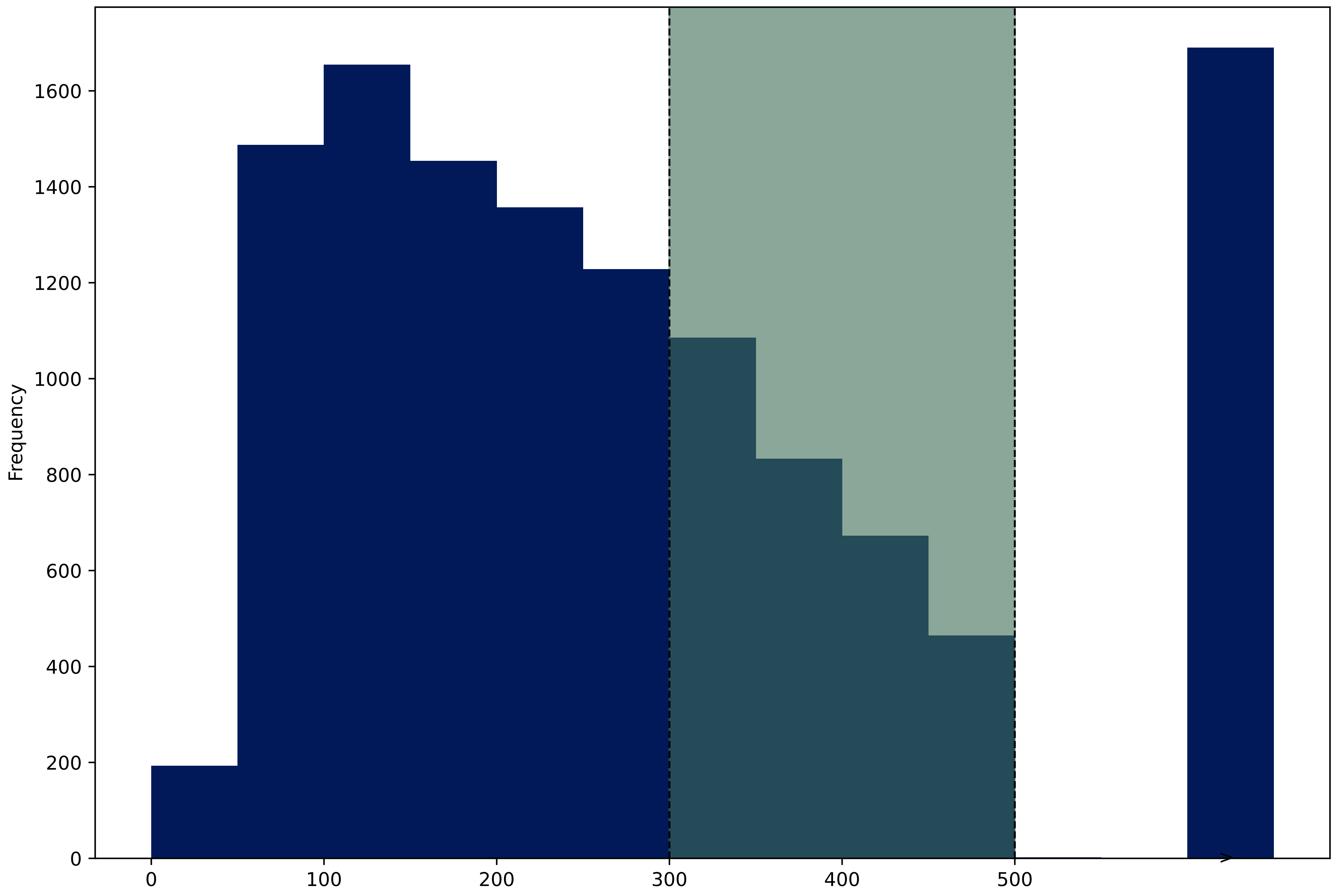

Distance to nearest park (m; up to 500m)

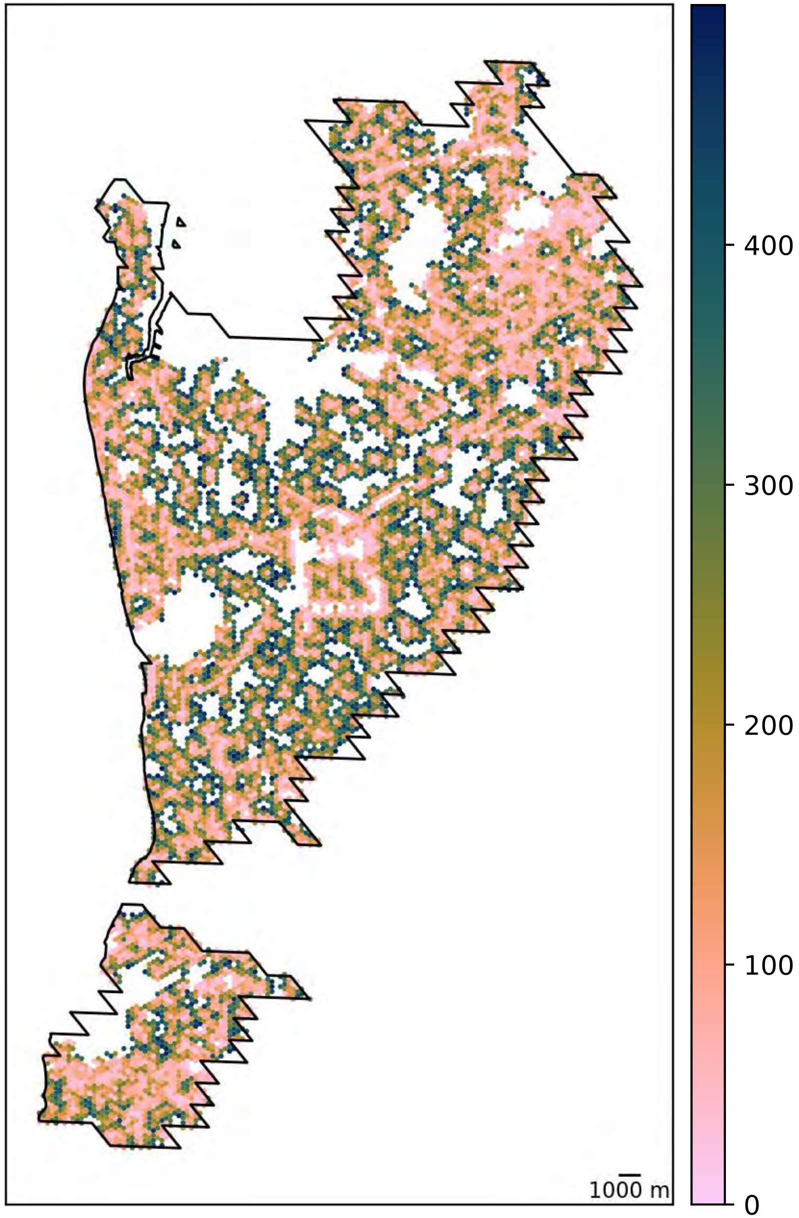

distances: Estimated Distance to nearest park (m; up to 500m) requirement for distances to destinations, measured up to a maximum distance target threshold of 500 metres

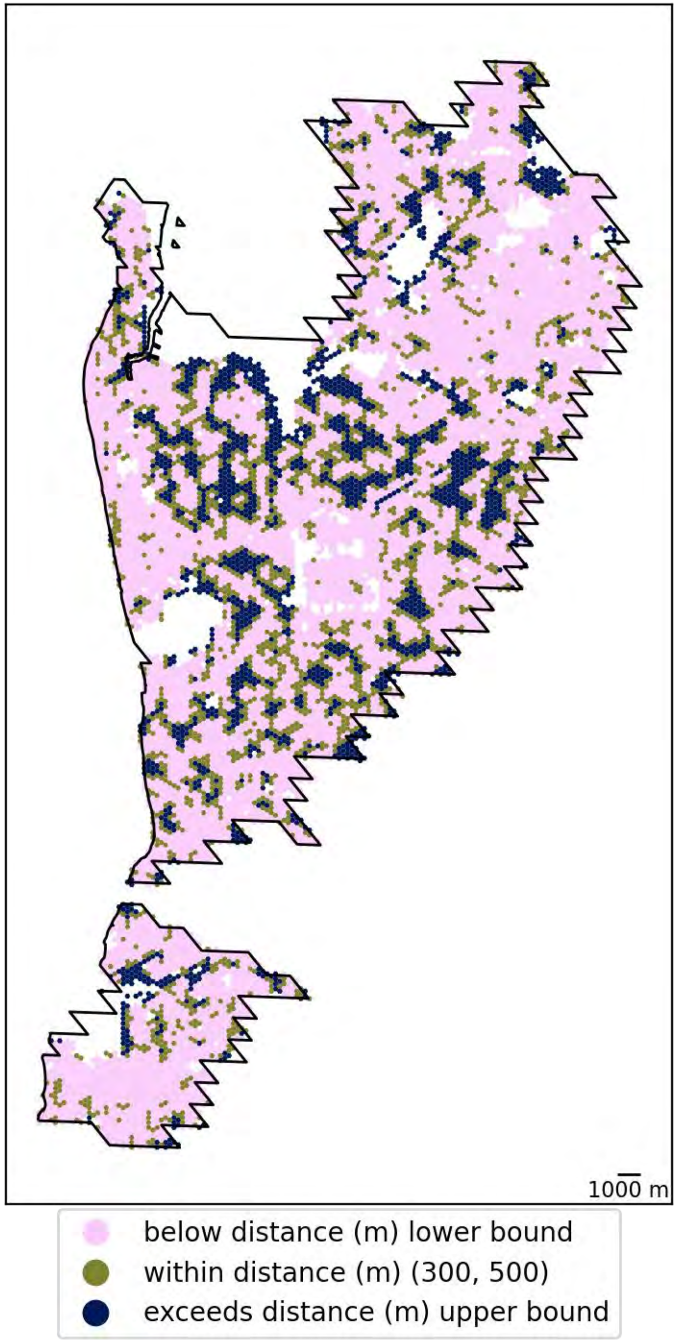

Histogram of Distance to nearest park (m; up to 500m).

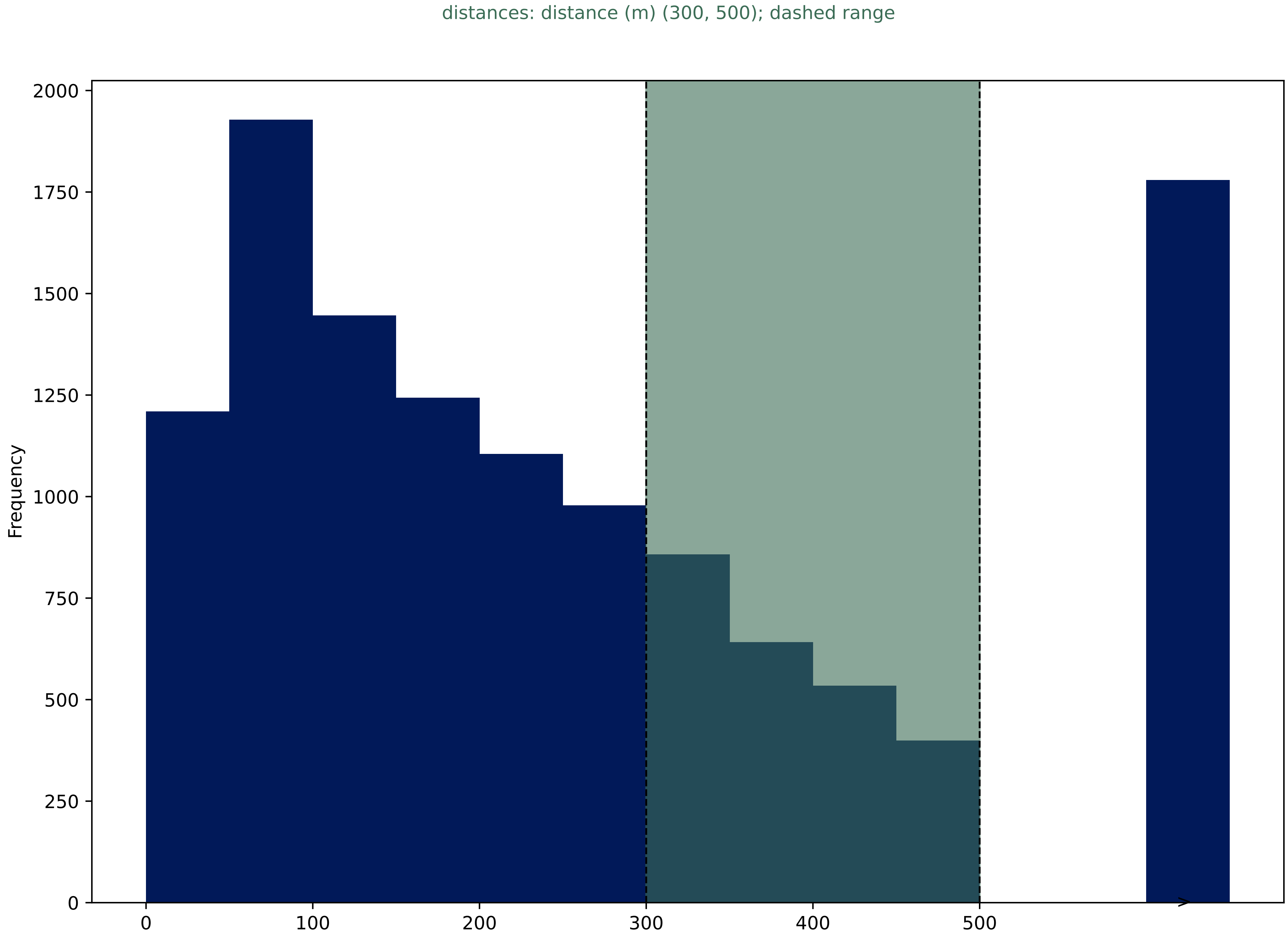

Australasia, Australia, Melbourne

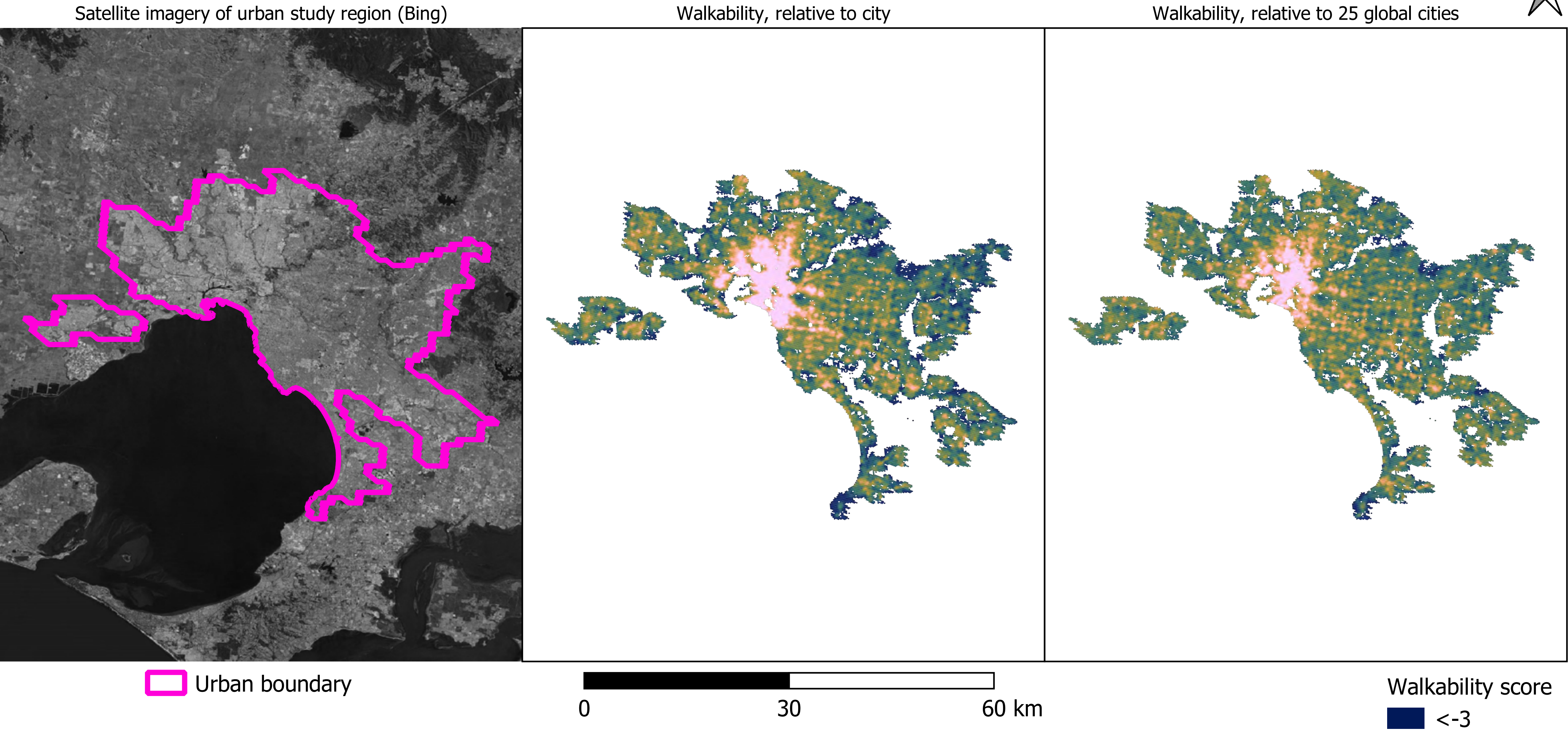

Walkability score

- <-3
- -3 to -2
- -2 to -1
- -1 to 0
- 0 to 1
- 1 to 2
- 2 to 3
- ≥3

Walkability relative to all cities by component variables (2D histograms), and overall (histogram)

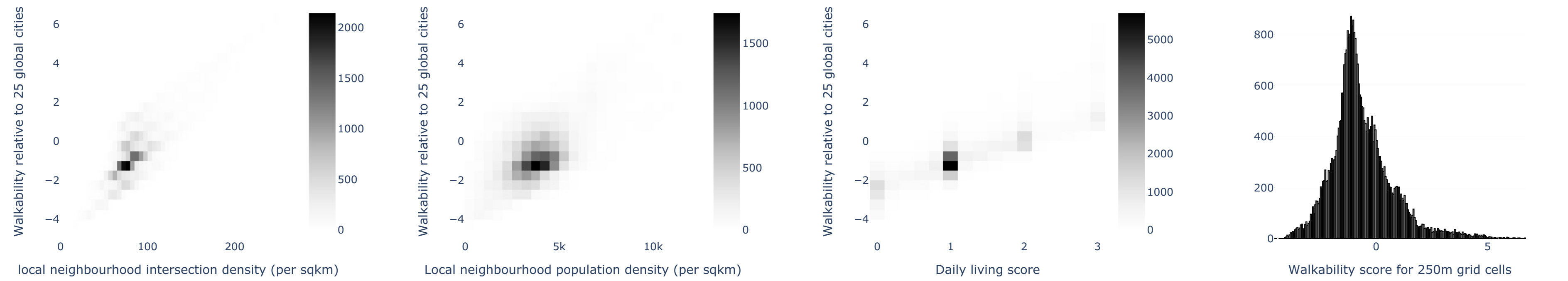

Mean 1000 m neighbourhood population per km²

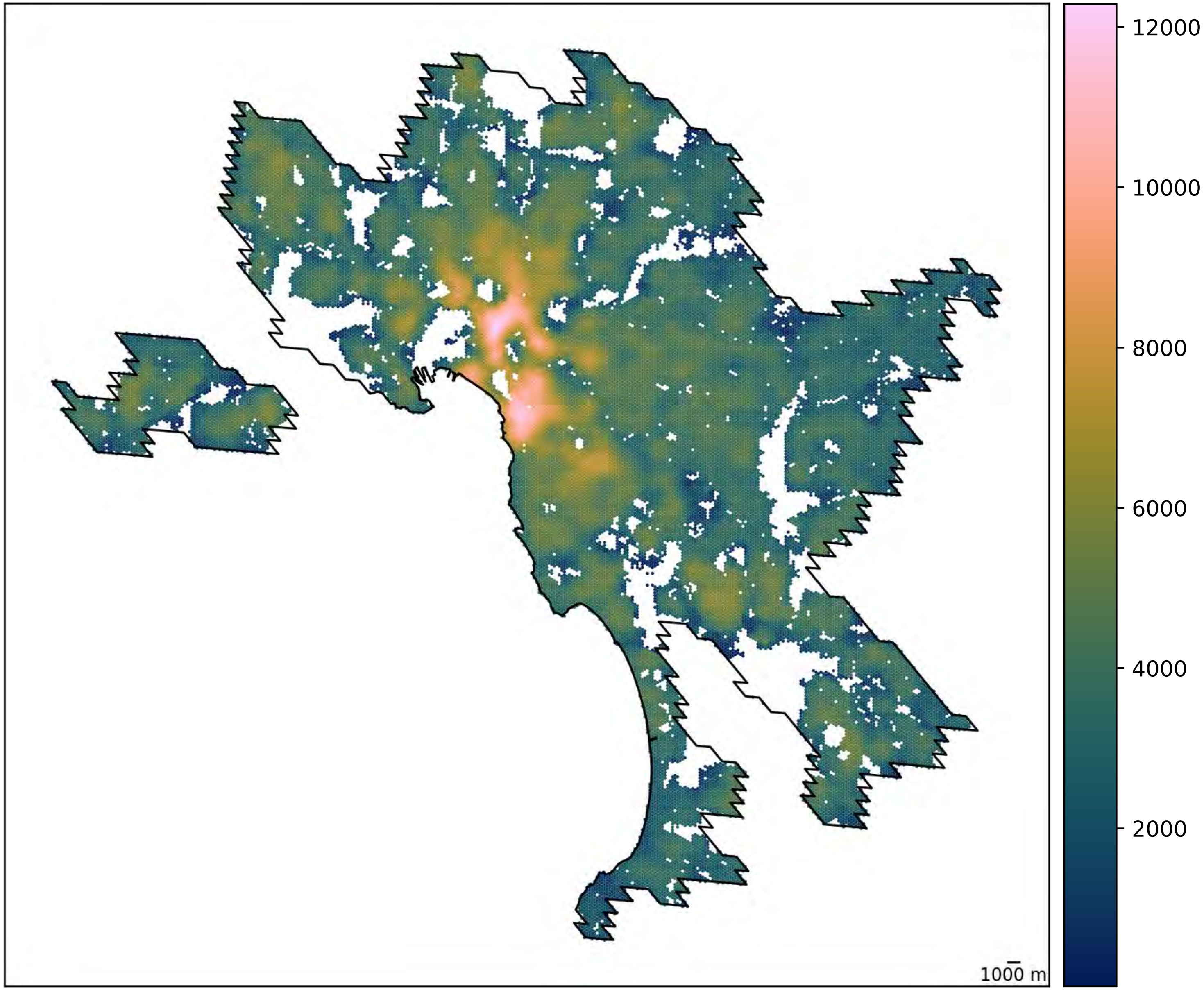

A: Estimated Mean 1000 m neighbourhood population per km² requirement for ≥80% probability of engaging in walking for transport

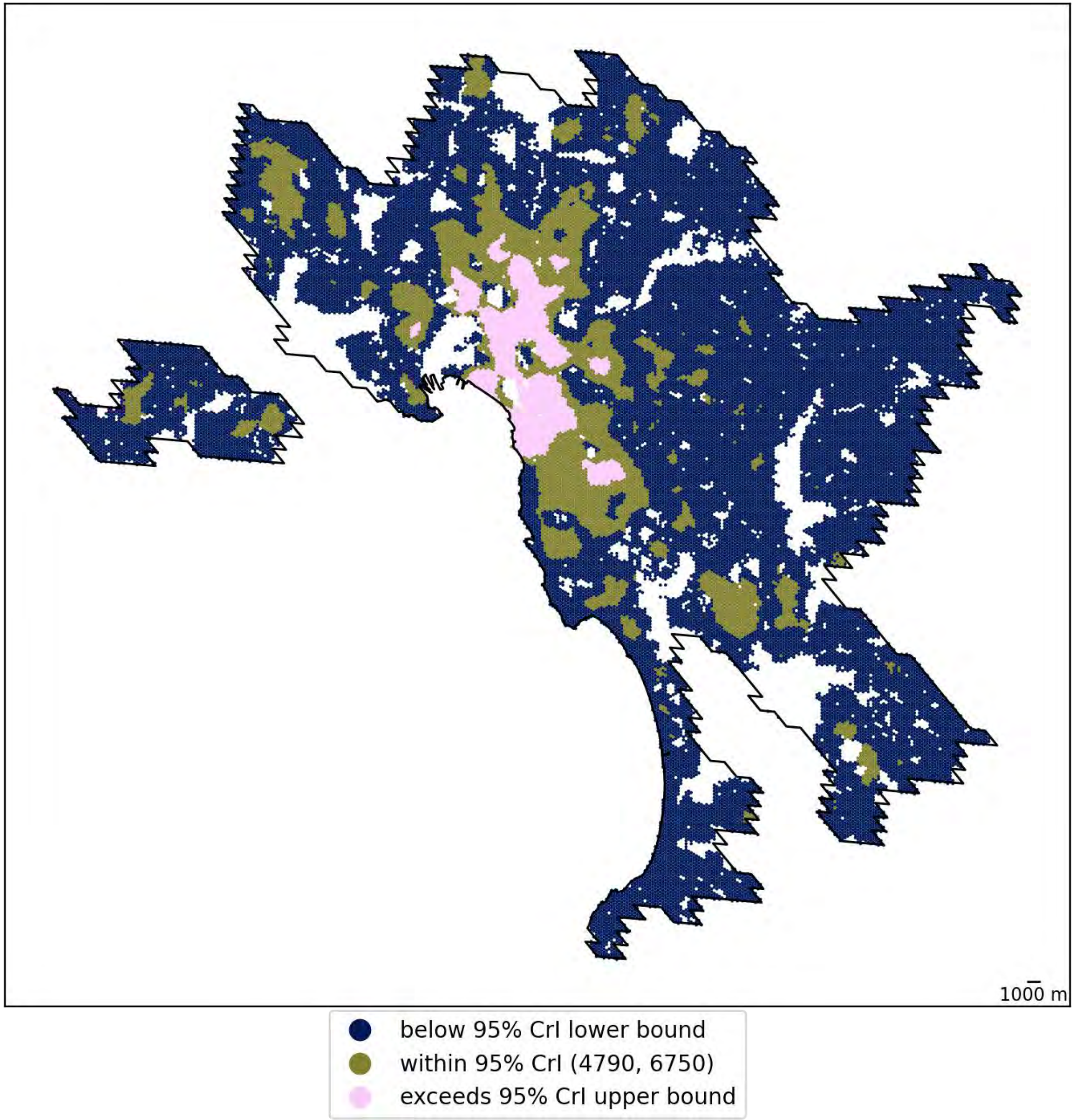

B: Estimated Mean 1000 m neighbourhood population per km² requirement for reaching the WHO's target of a ≥15% relative reduction in insufficient physical activity through walking

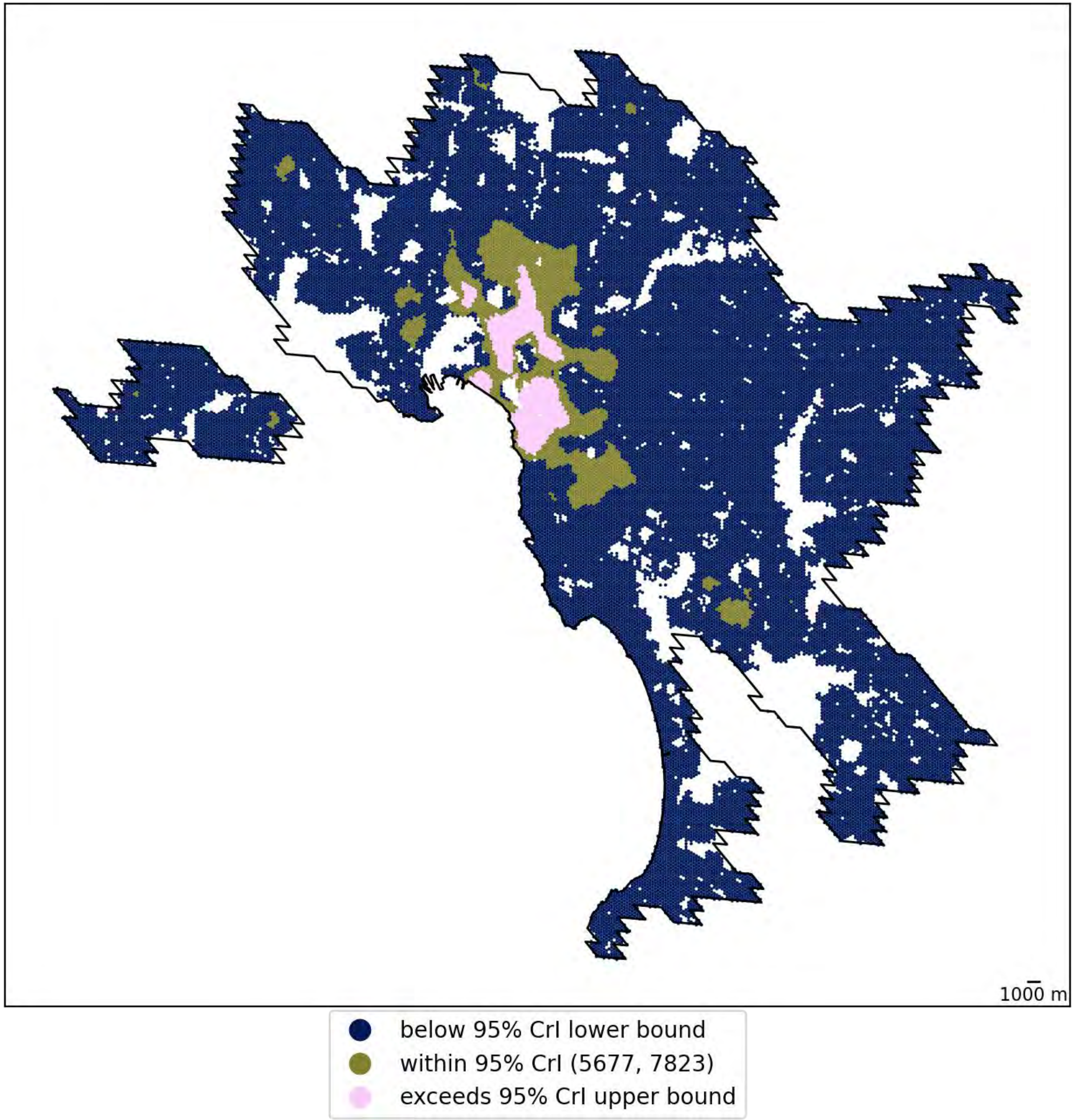

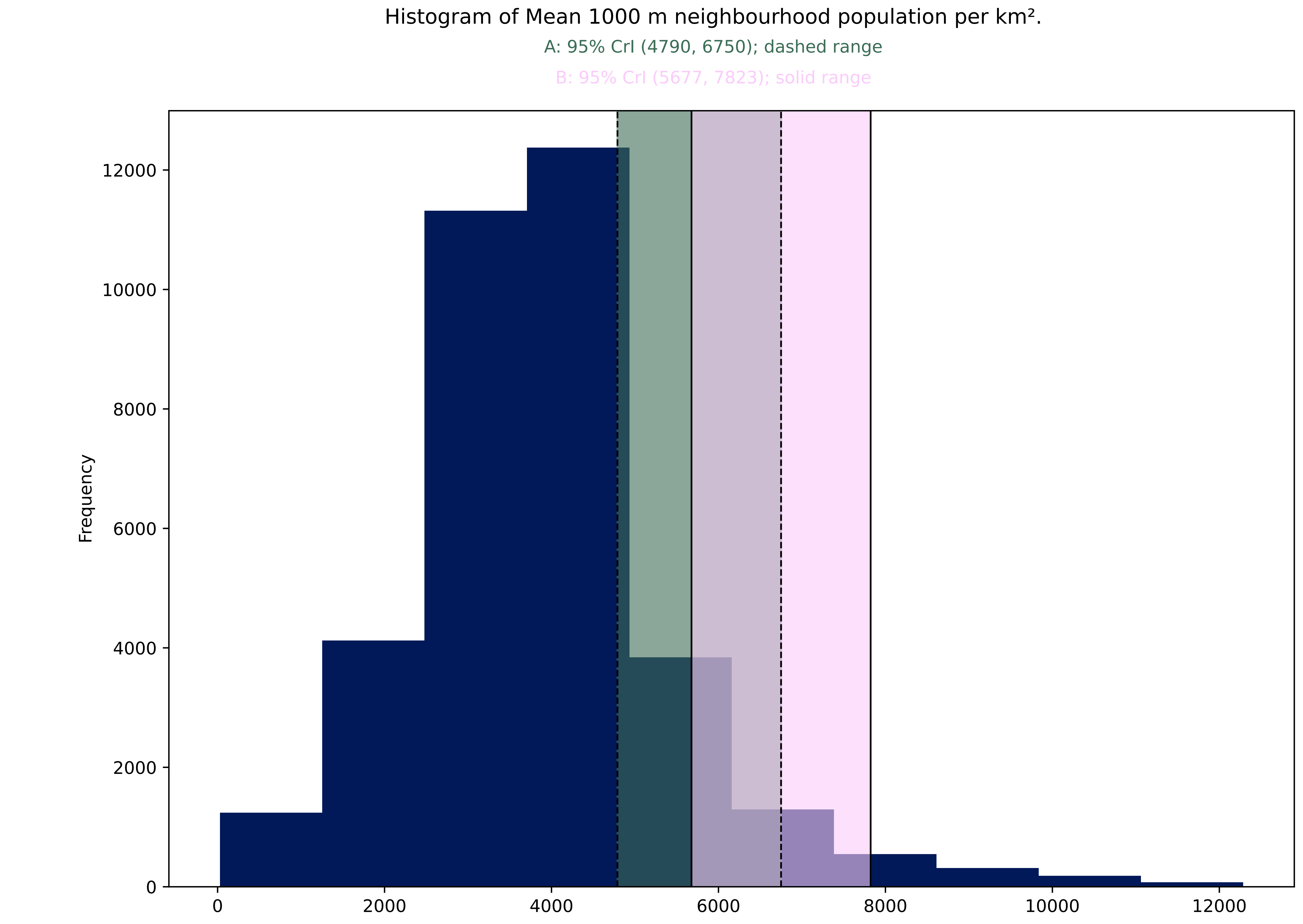

Histogram of Mean 1000 m neighbourhood population per km².
A: 95% CrI (4790, 6750); dashed range
B: 95% CrI (5677, 7823); solid range
Frequency
0
2000
4000
6000
8000
10000
12000

Mean 1000 m neighbourhood street intersections per km²

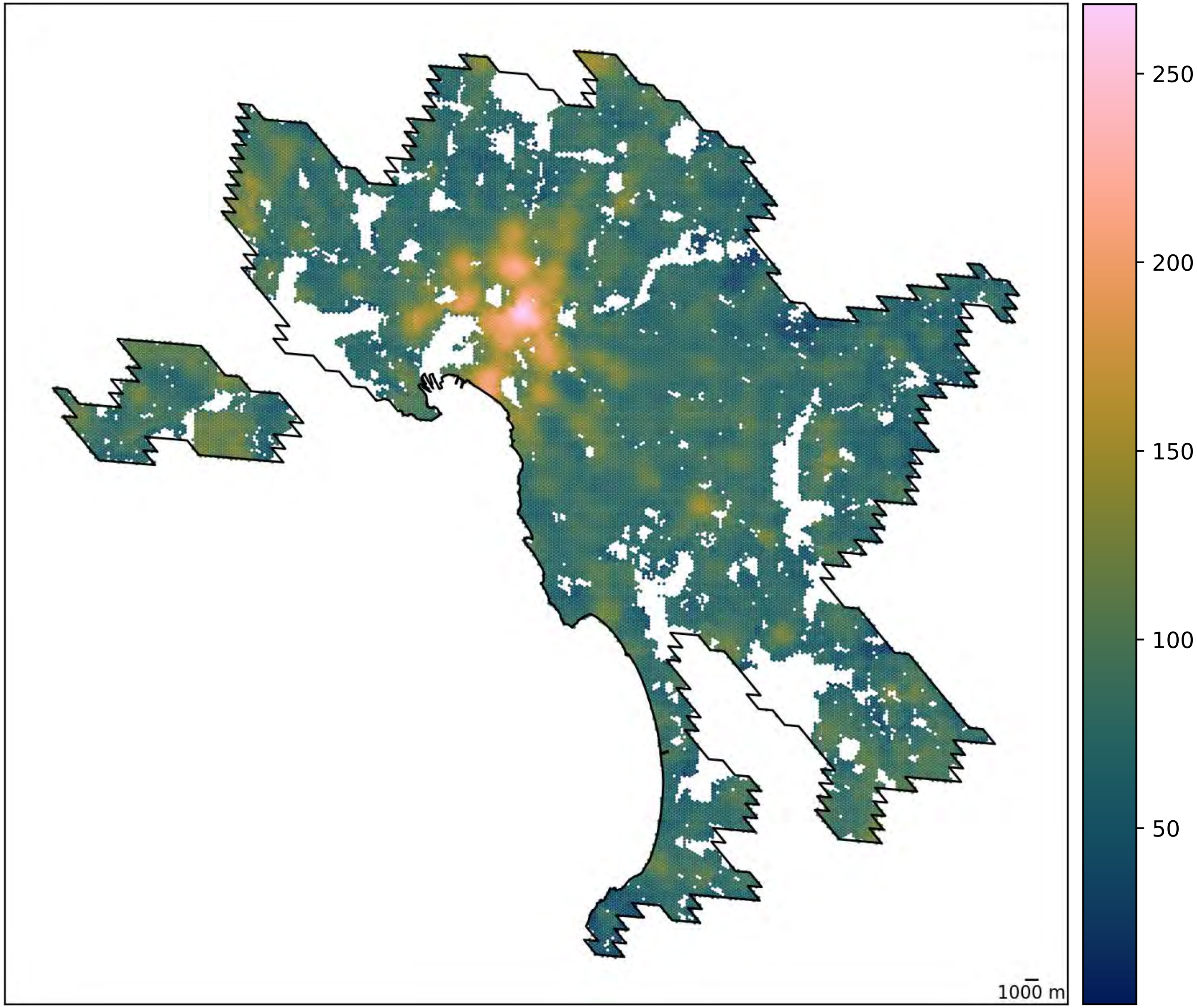

A: Estimated Mean 1000 m neighbourhood street intersections per km² requirement for ≥80% probability of engaging in walking for transport

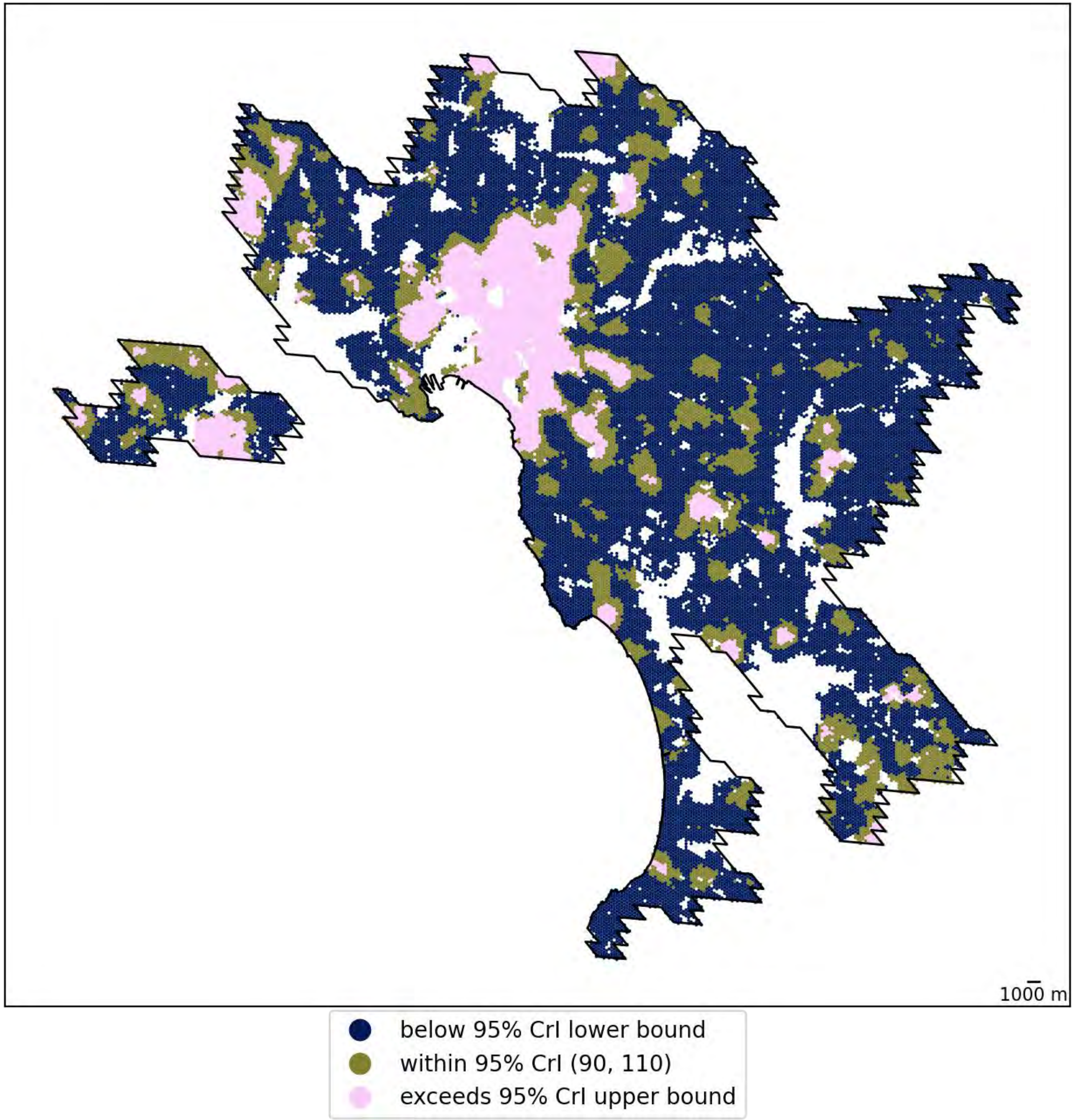

B: Estimated Mean 1000 m neighbourhood street intersections per km² requirement for reaching the WHO's target of a ≥15% relative reduction in insufficient physical activity through walking

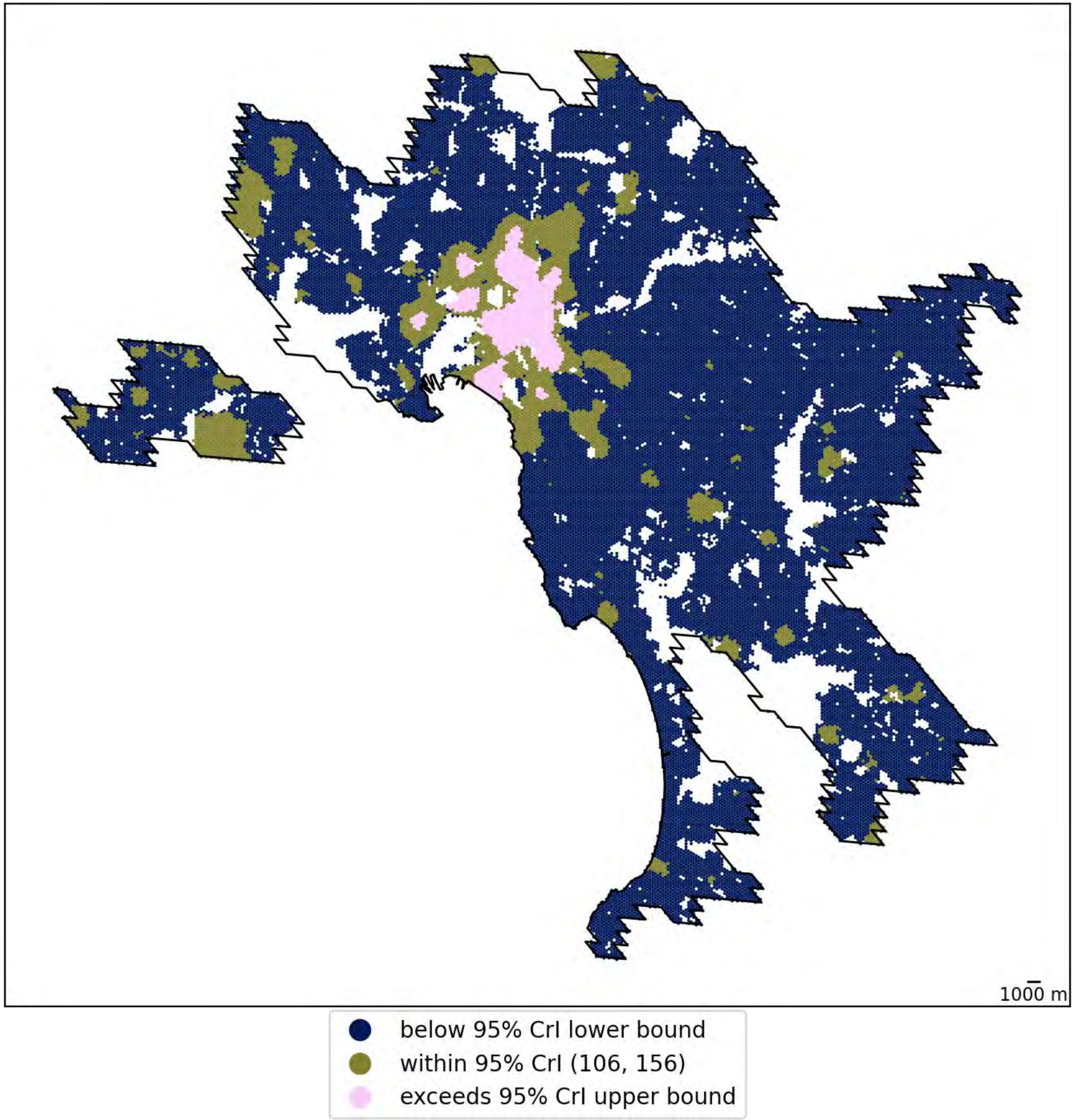

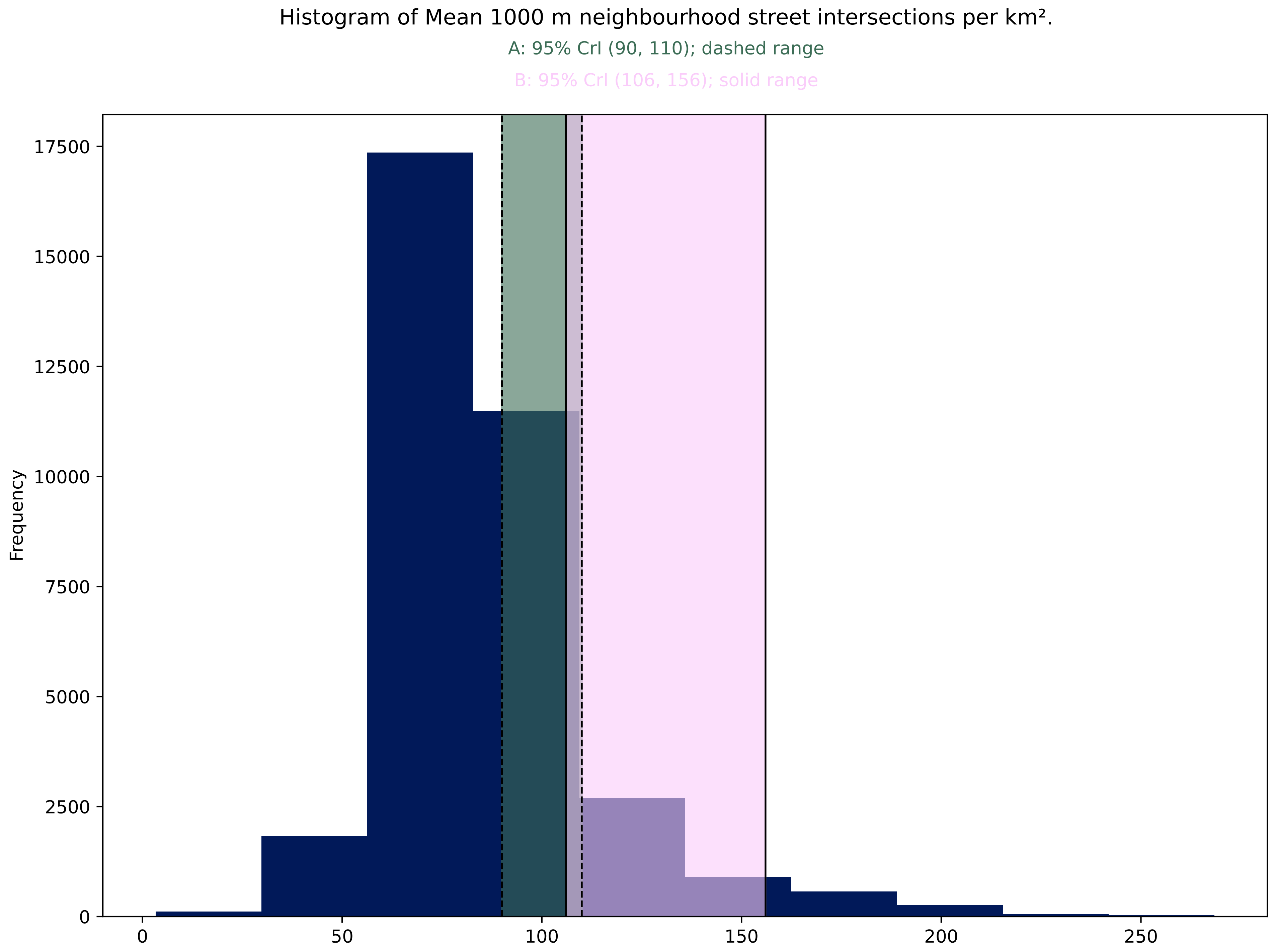
Histogram of Mean 1000 m neighbourhood street intersections per km².
A: 95% CrI (90, 110); dashed range
B: 95% CrI (106, 156); solid range
Frequency
0
2500
5000
7500
10000
12500
15000
17500
0
50
100
150
200
250

Distance to nearest public transport stops (m; up to 500m)

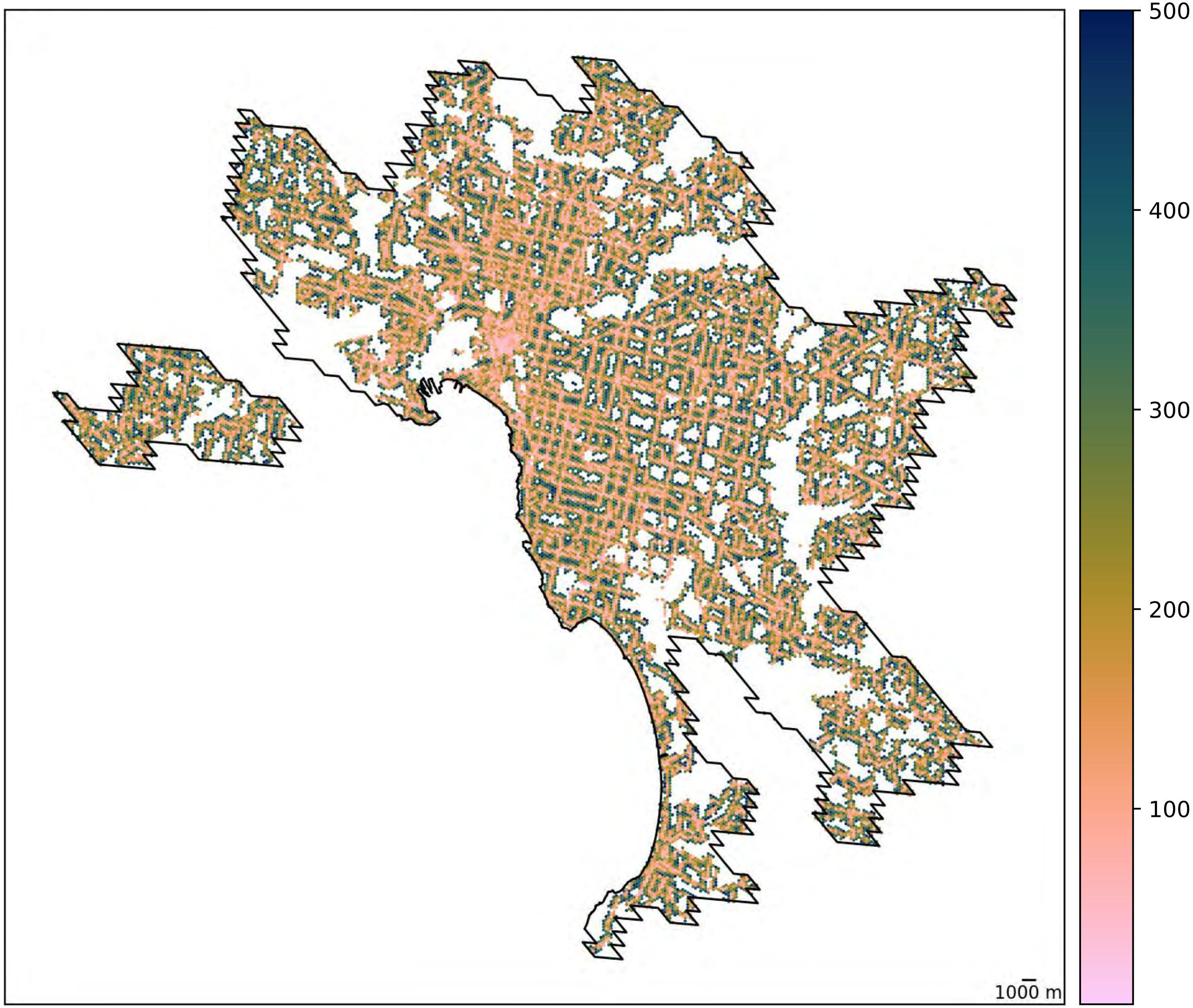

distances: Estimated Distance to nearest public transport stops (m; up to 500m) requirement for distances to destinations, measured up to a maximum distance target threshold of 500 metres

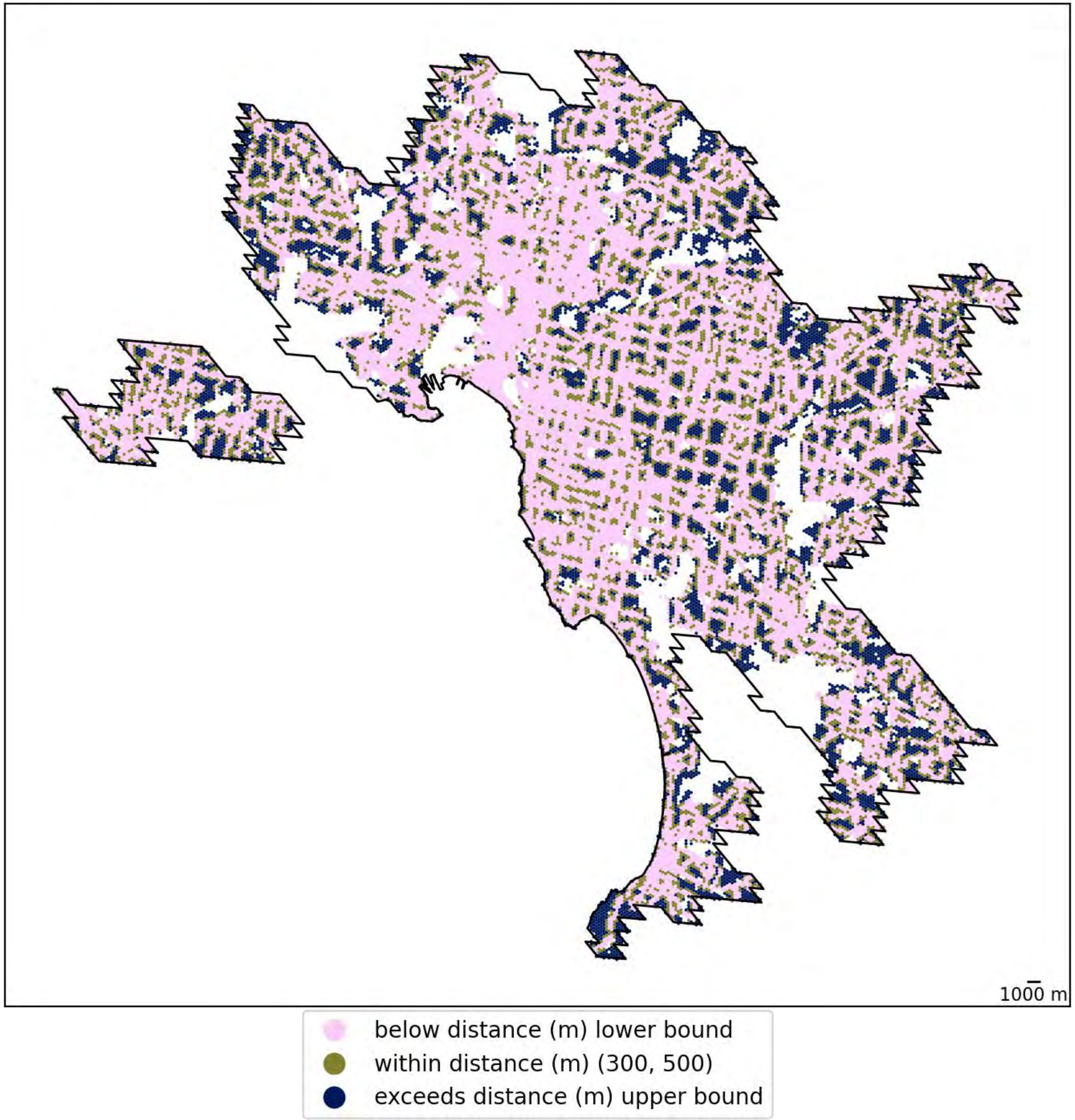

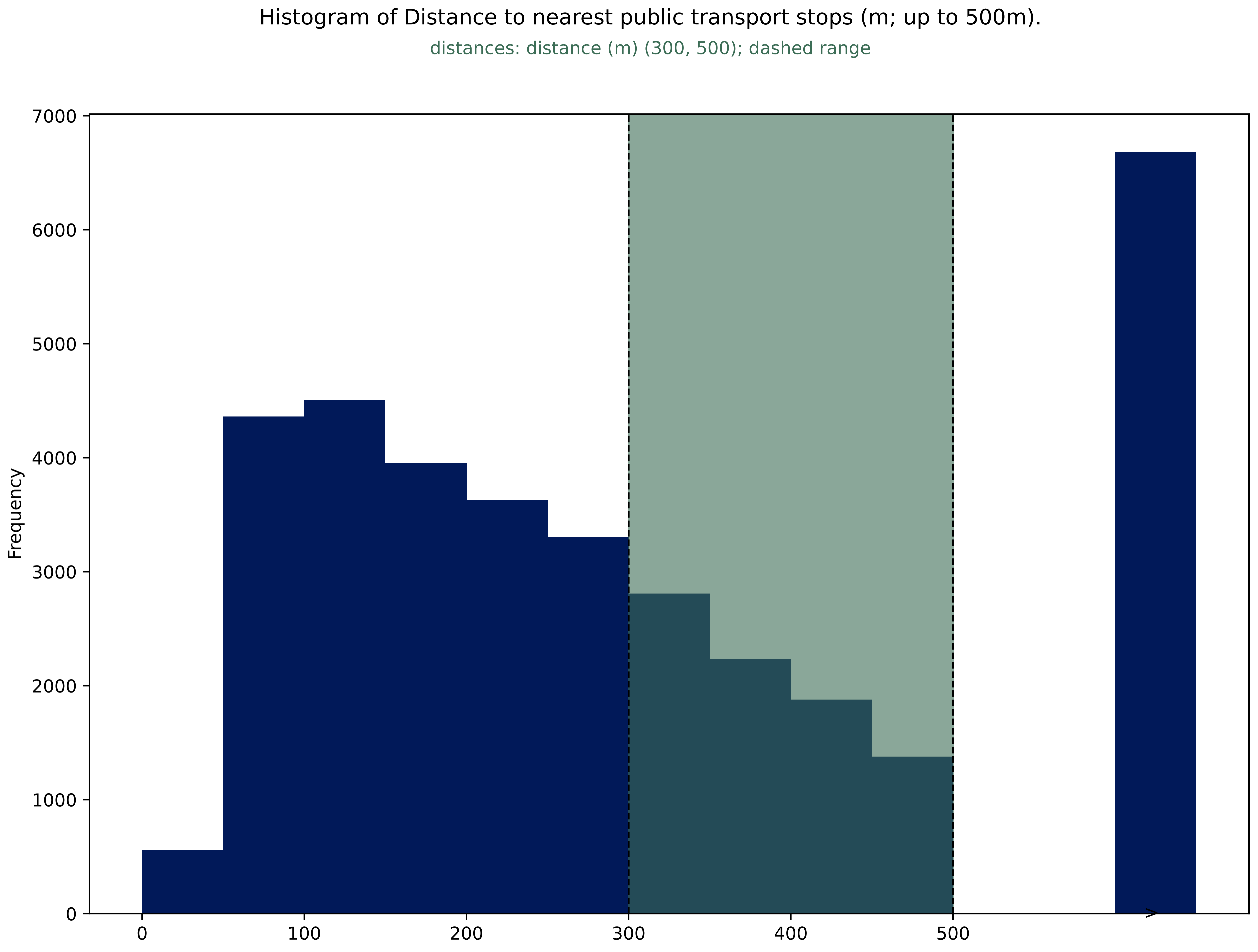

Histogram of Distance to nearest public transport stops (m; up to 500m).
distances: distance (m) (300, 500); dashed range
Frequency
0
1000
2000
3000
4000
5000
6000
7000
0
100
200
300
400
500
>

Distance to nearest park (m; up to 500m)

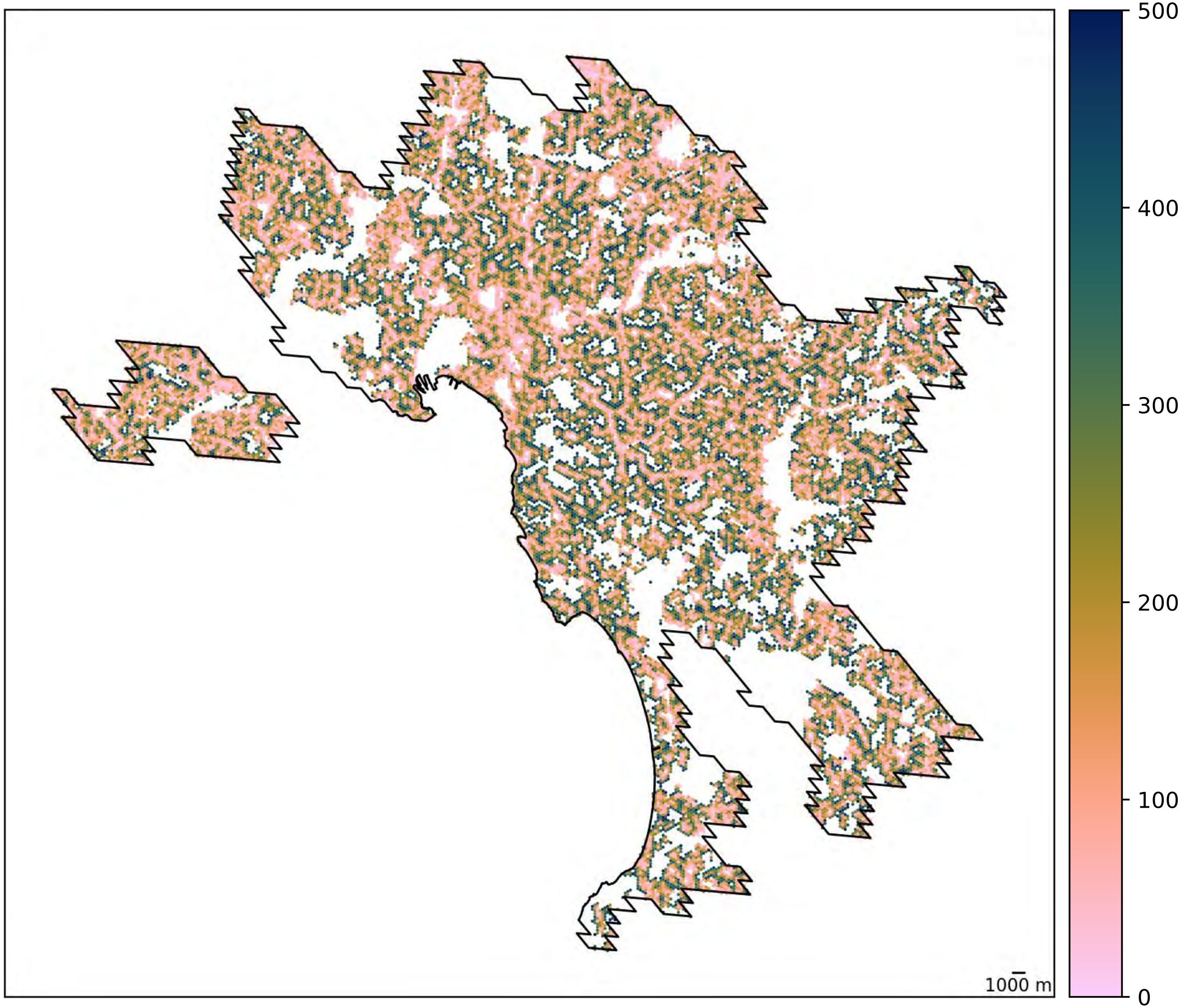

distances: Estimated Distance to nearest park (m; up to 500m) requirement for distances to destinations, measured up to a maximum distance target threshold of 500 metres

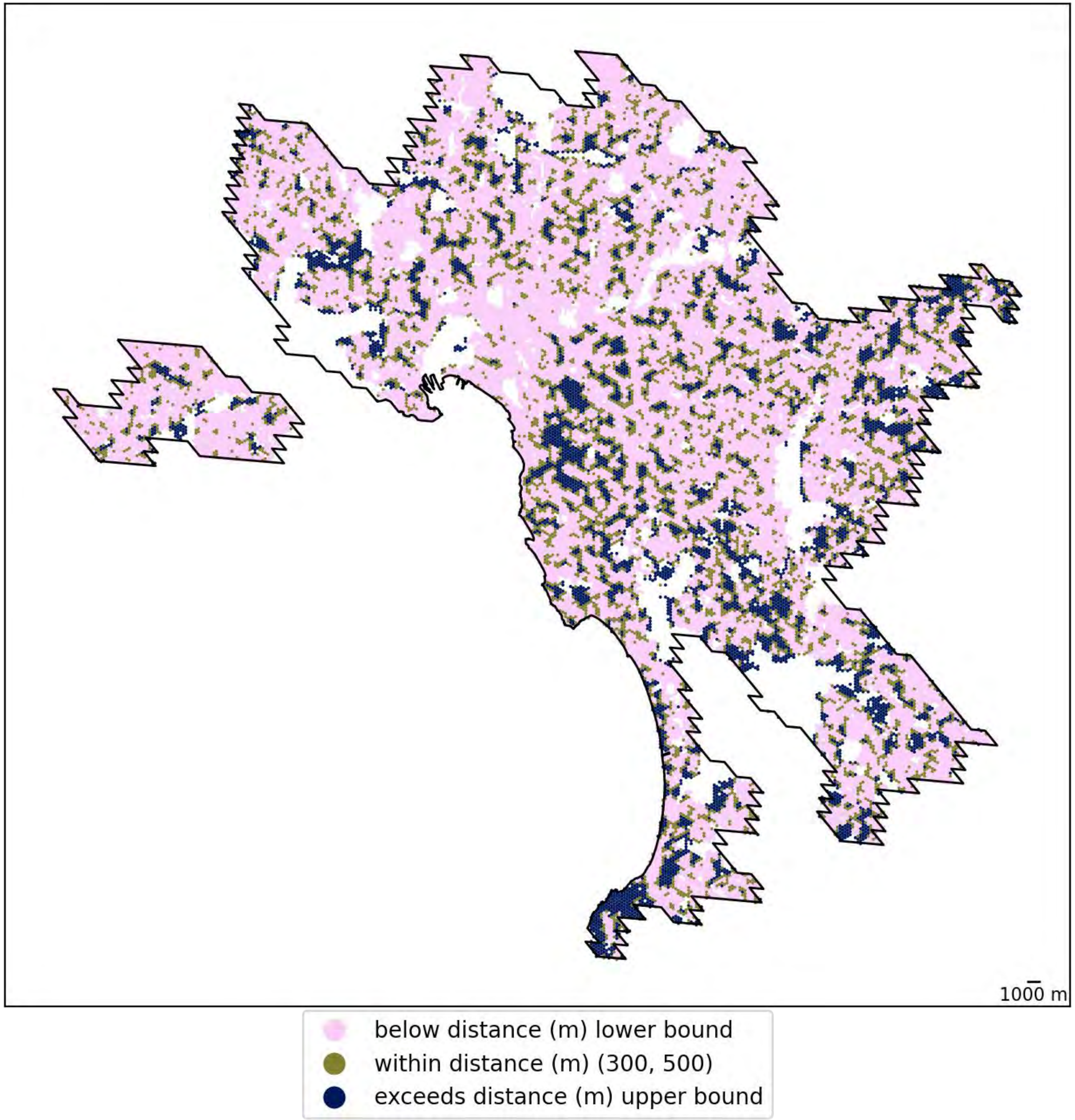

Histogram of Distance to nearest park (m; up to 500m).

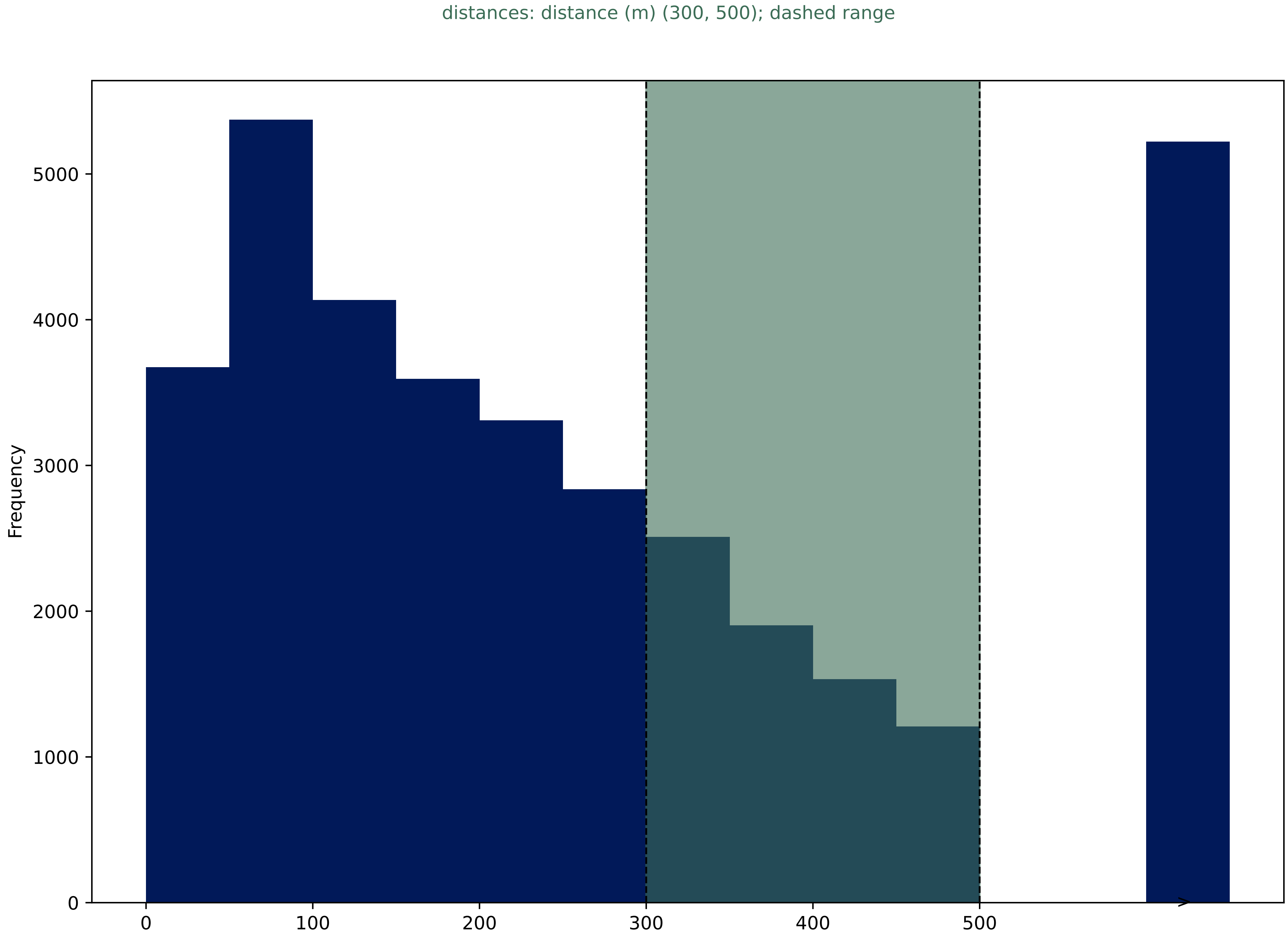

Australasia, Australia, Sydney

Satellite imagery of urban study region (Bing) | Walkability, relative to city | Walkability, relative to 25 global cities

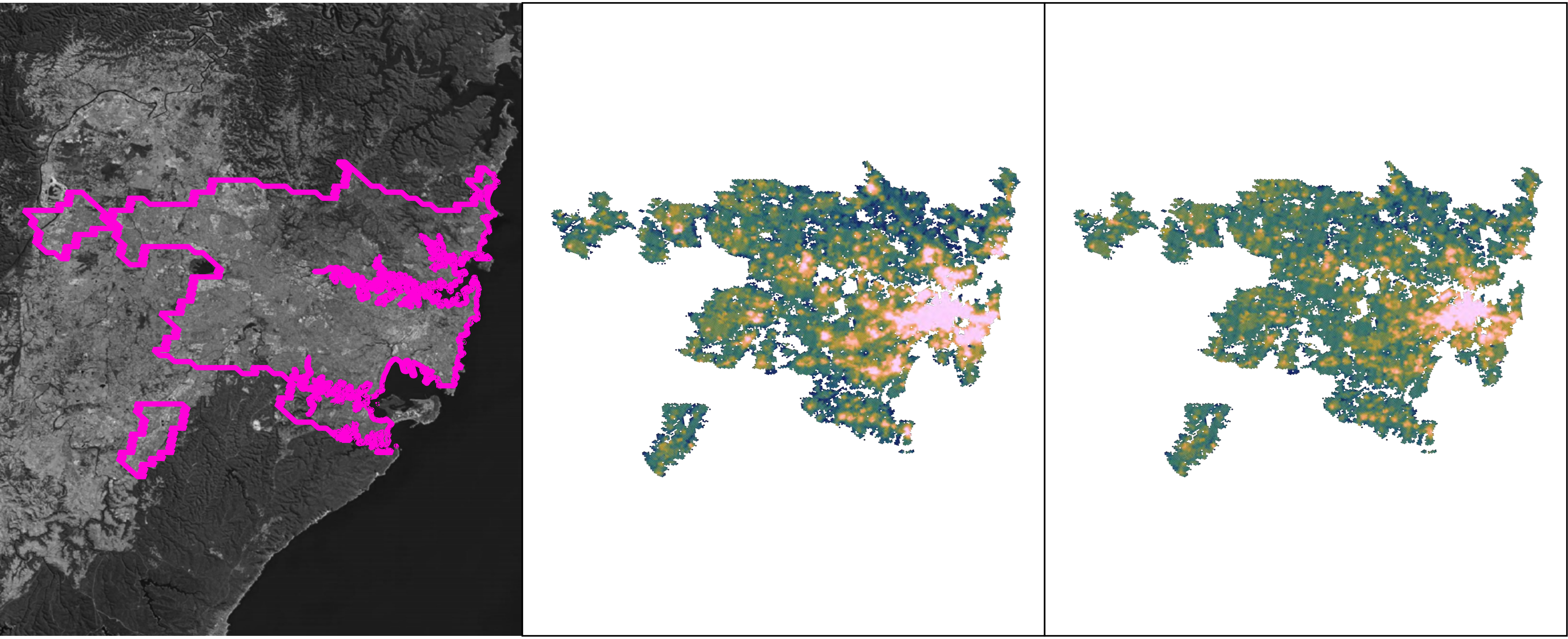

Urban boundary

0 30 60 km

Walkability score
- <-3
- -3 to -2
- -2 to -1
- -1 to 0
- 0 to 1
- 1 to 2
- 2 to 3
- ≥3

Walkability relative to all cities by component variables (2D histograms), and overall (histogram)

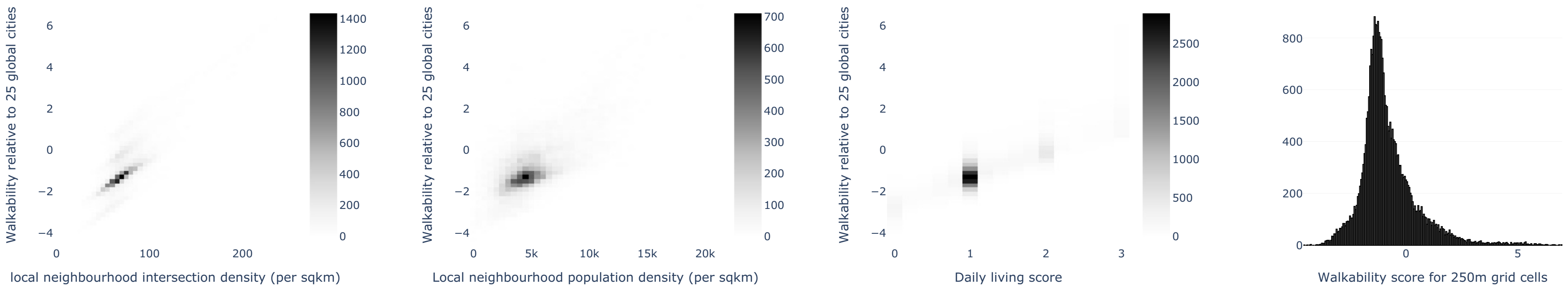

Mean 1000 m neighbourhood population per km²

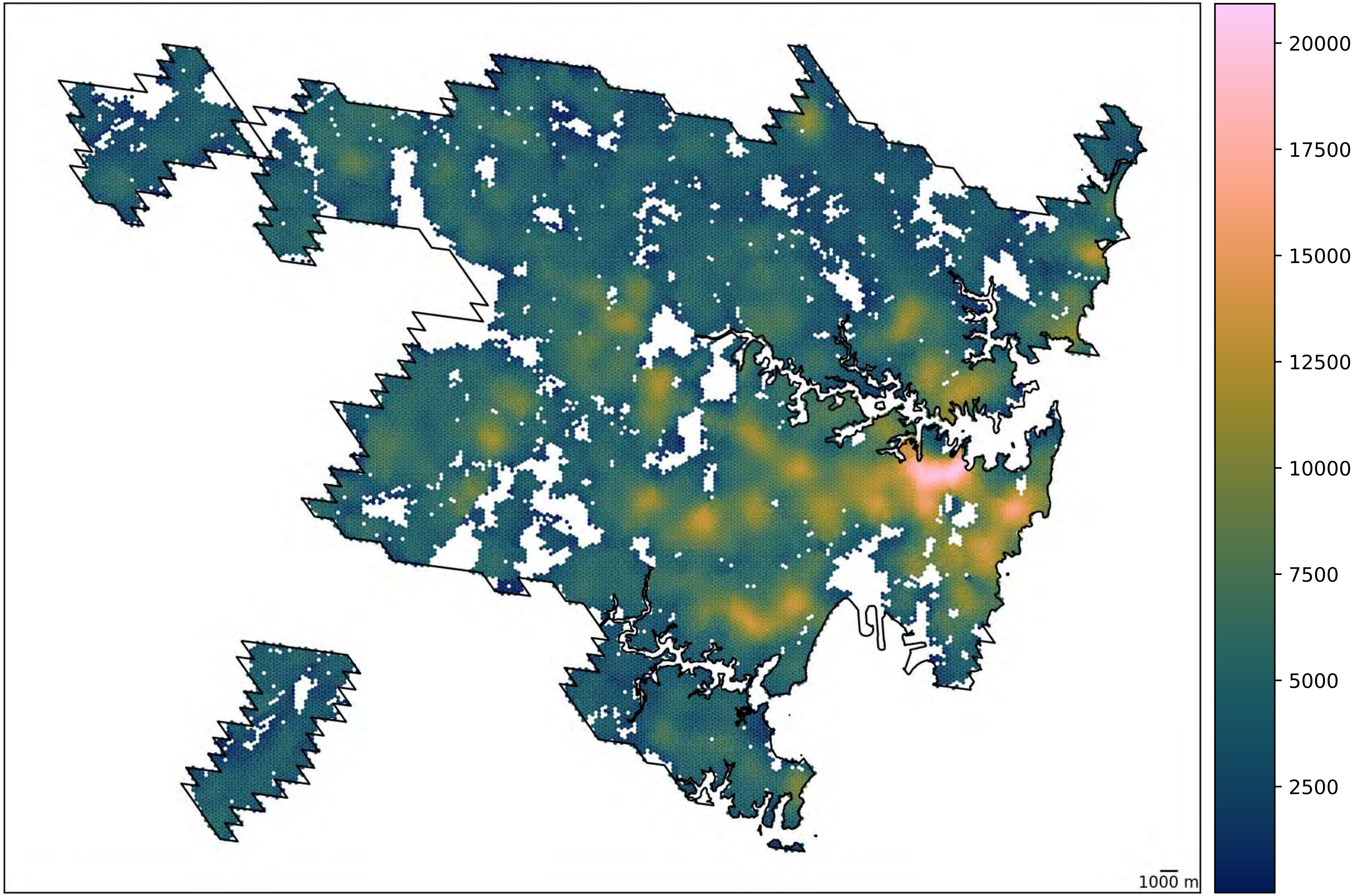

A: Estimated Mean 1000 m neighbourhood population per km² requirement for ≥80% probability of engaging in walking for transport

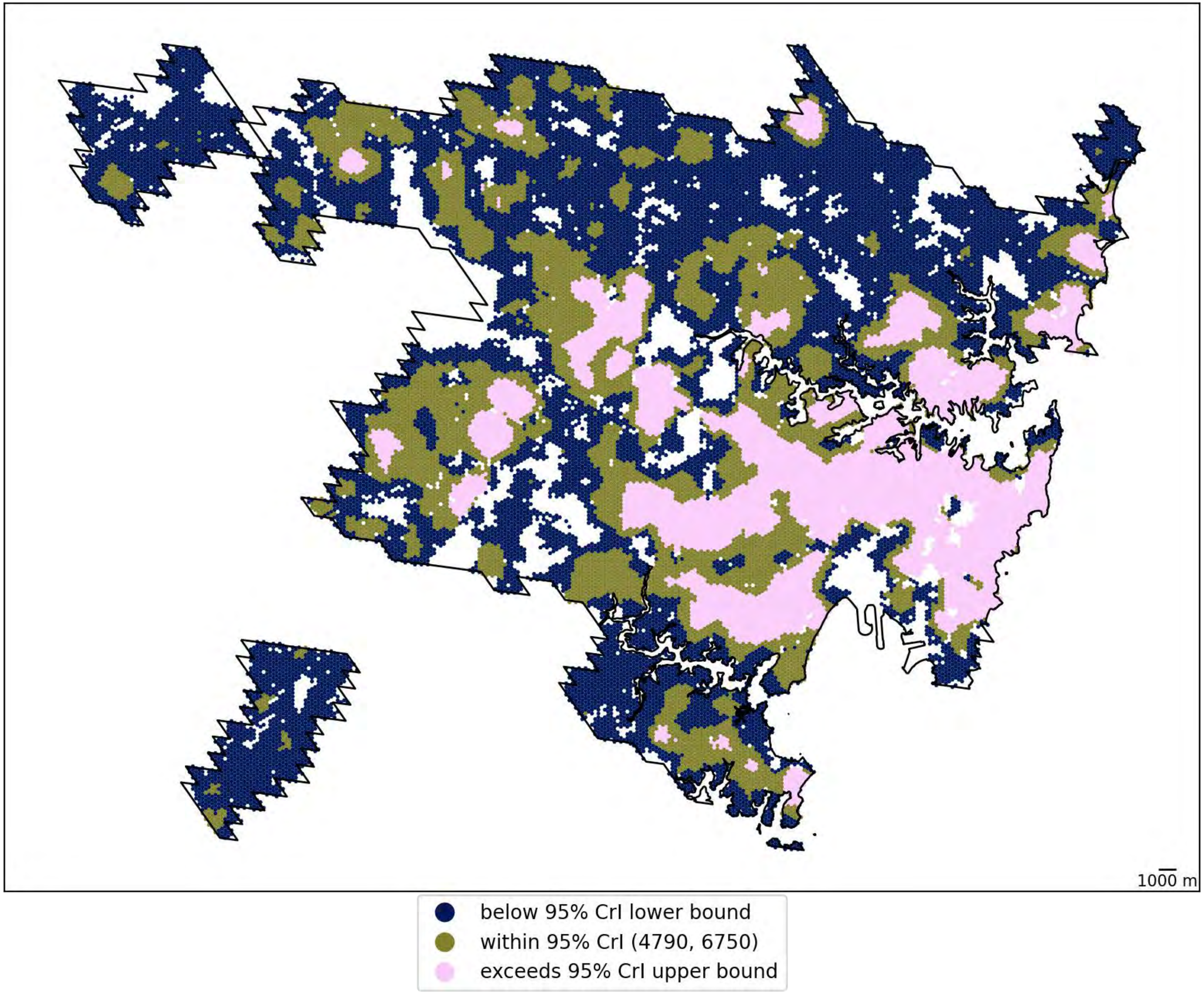

B: Estimated Mean 1000 m neighbourhood population per km² requirement for reaching the WHO's target of a ≥15% relative reduction in insufficient physical activity through walking

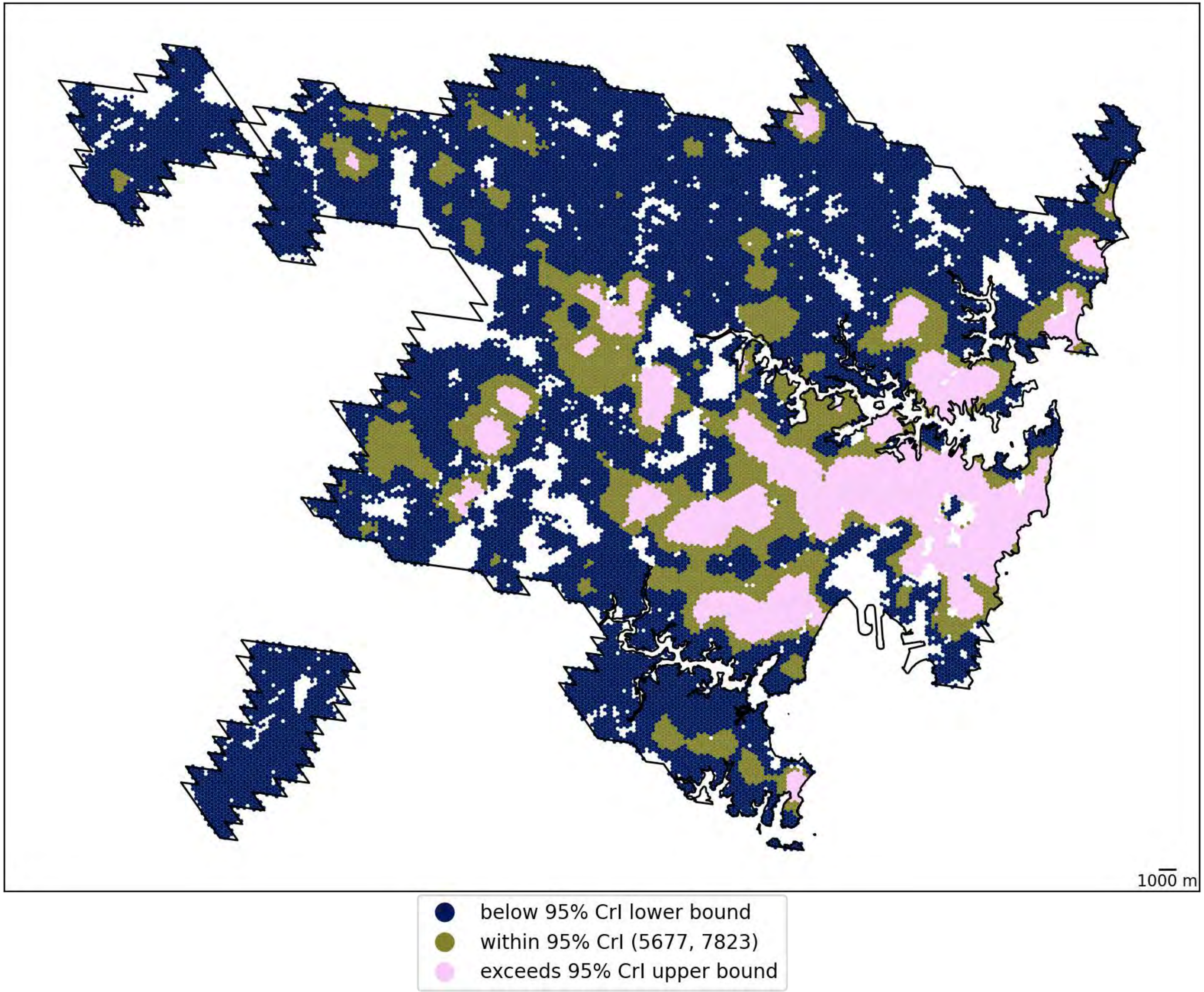

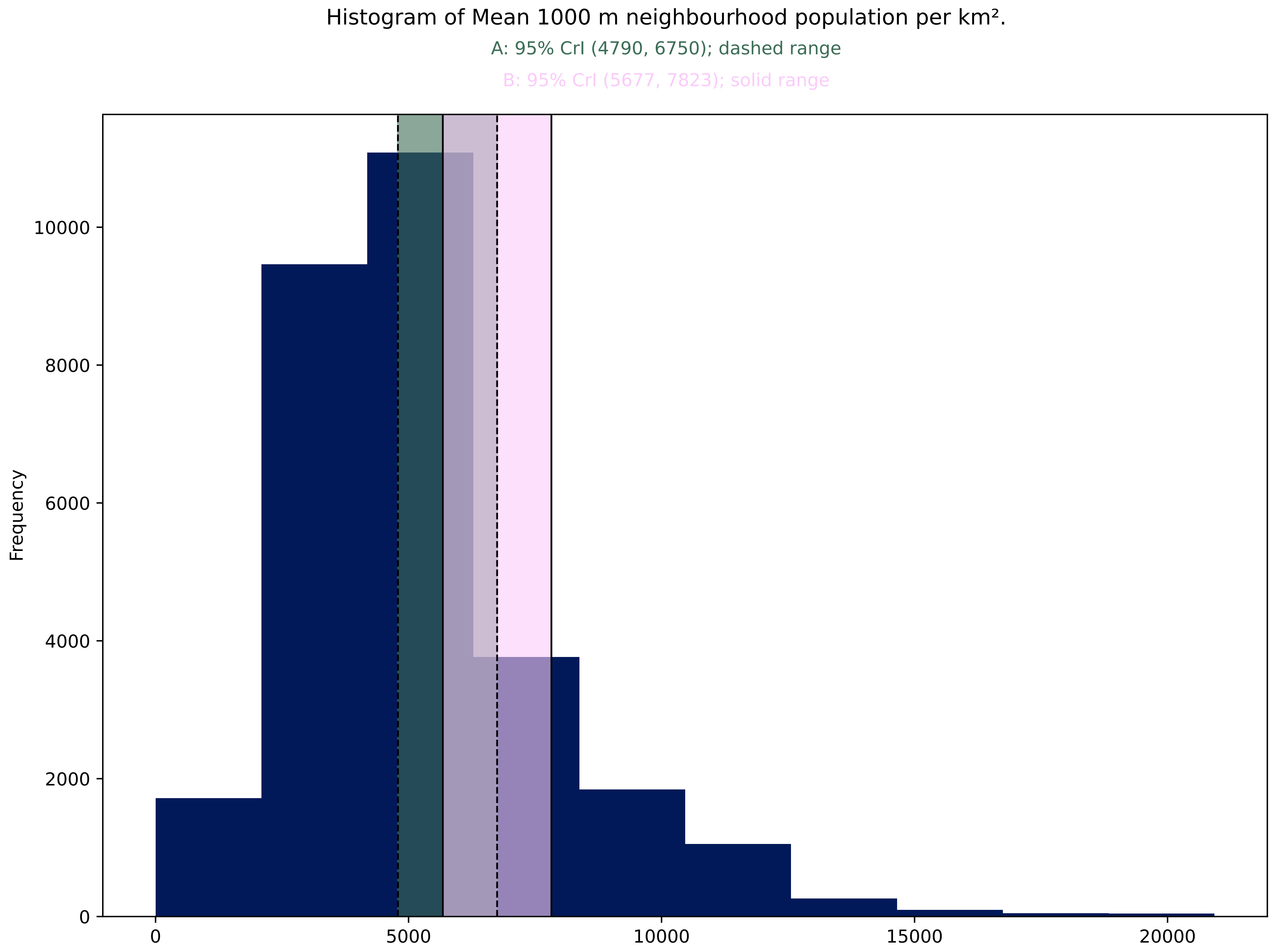

Histogram of Mean 1000 m neighbourhood population per km².
A: 95% CrI (4790, 6750); dashed range
B: 95% CrI (5677, 7823); solid range
Frequency
0
2000
4000
6000
8000
10000
0
5000
10000
15000
20000

Mean 1000 m neighbourhood street intersections per km²

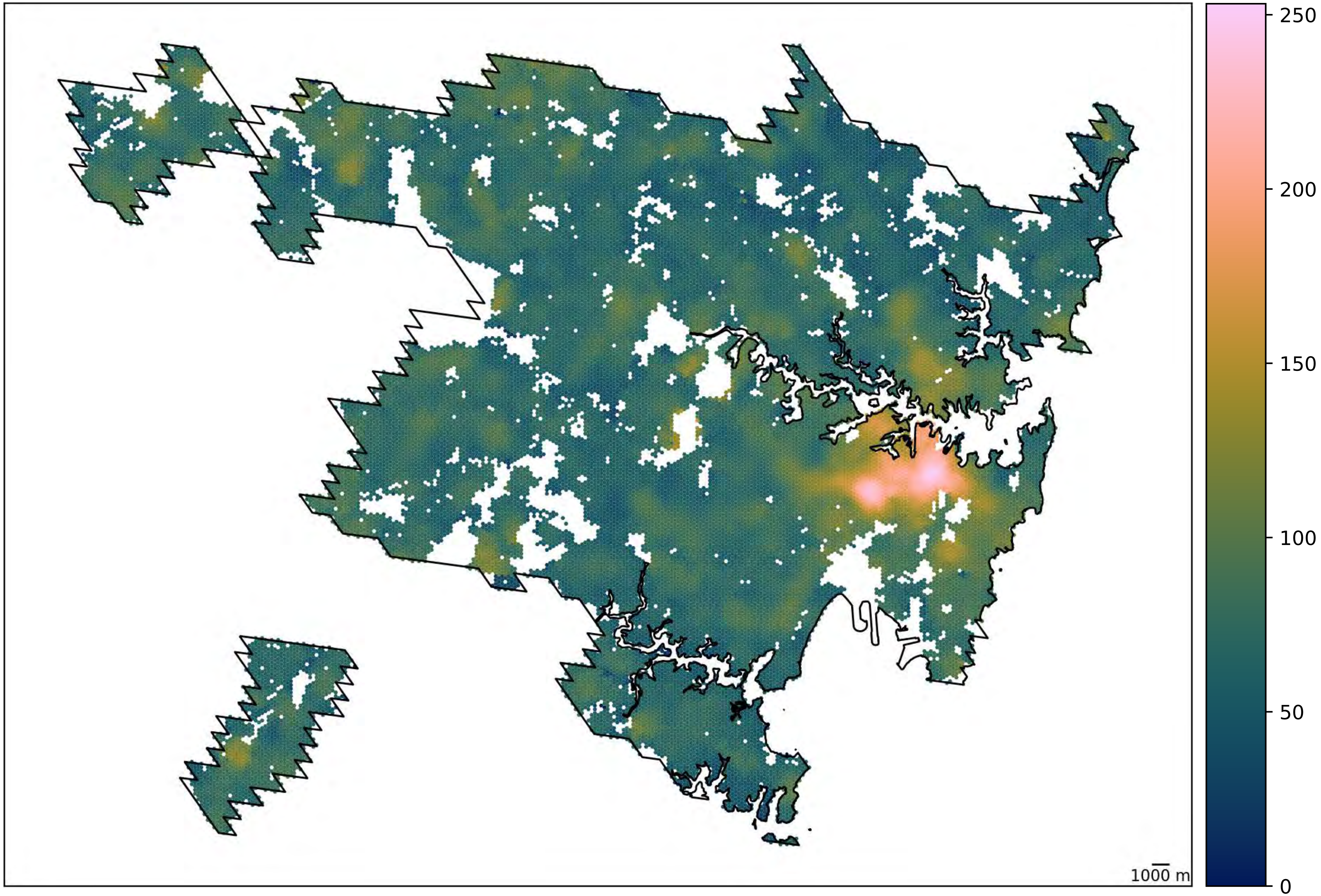

A: Estimated Mean 1000 m neighbourhood street intersections per km² requirement for ≥80% probability of engaging in walking for transport

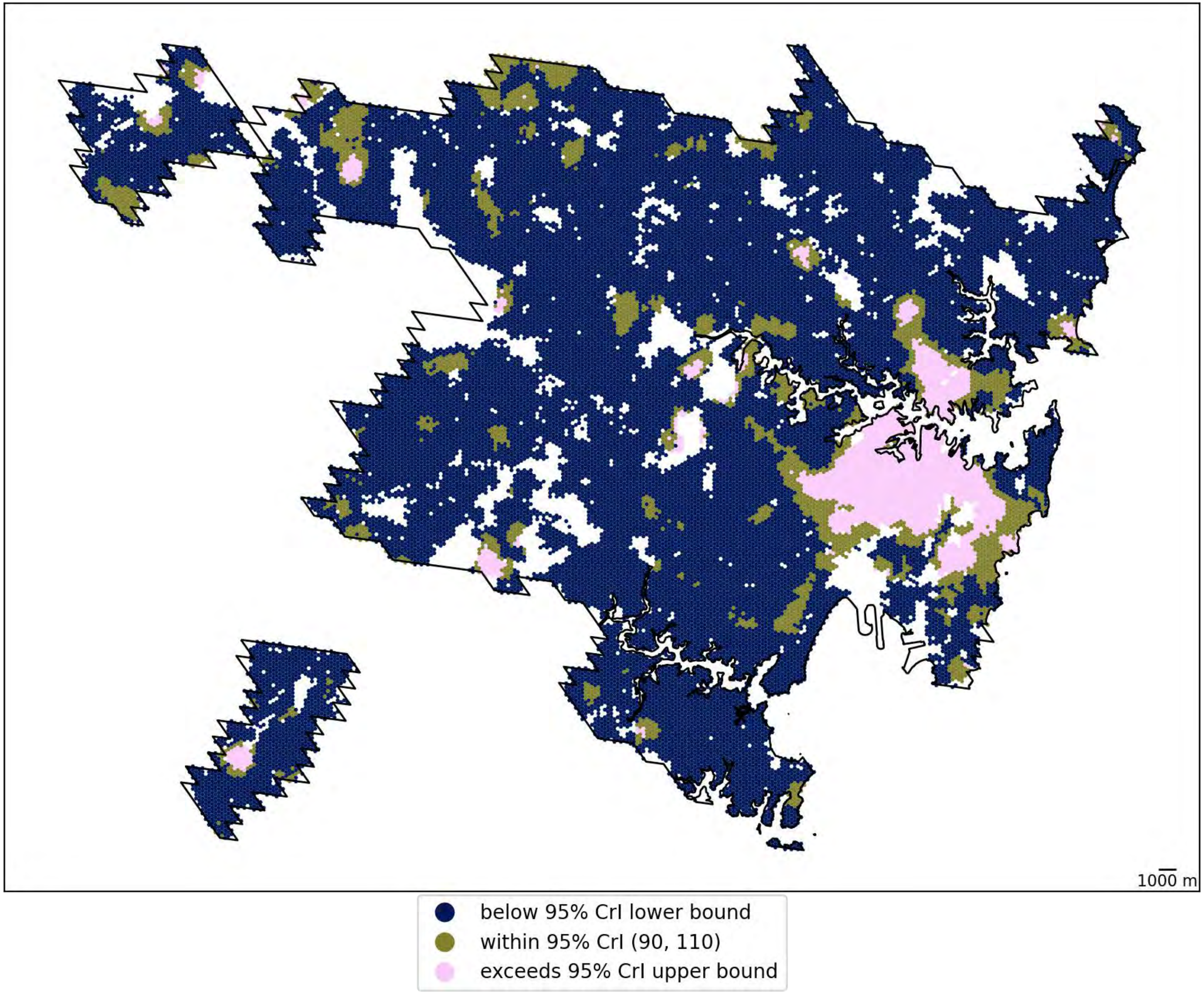

B: Estimated Mean 1000 m neighbourhood street intersections per km² requirement for reaching the WHO's target of a ≥15% relative reduction in insufficient physical activity through walking

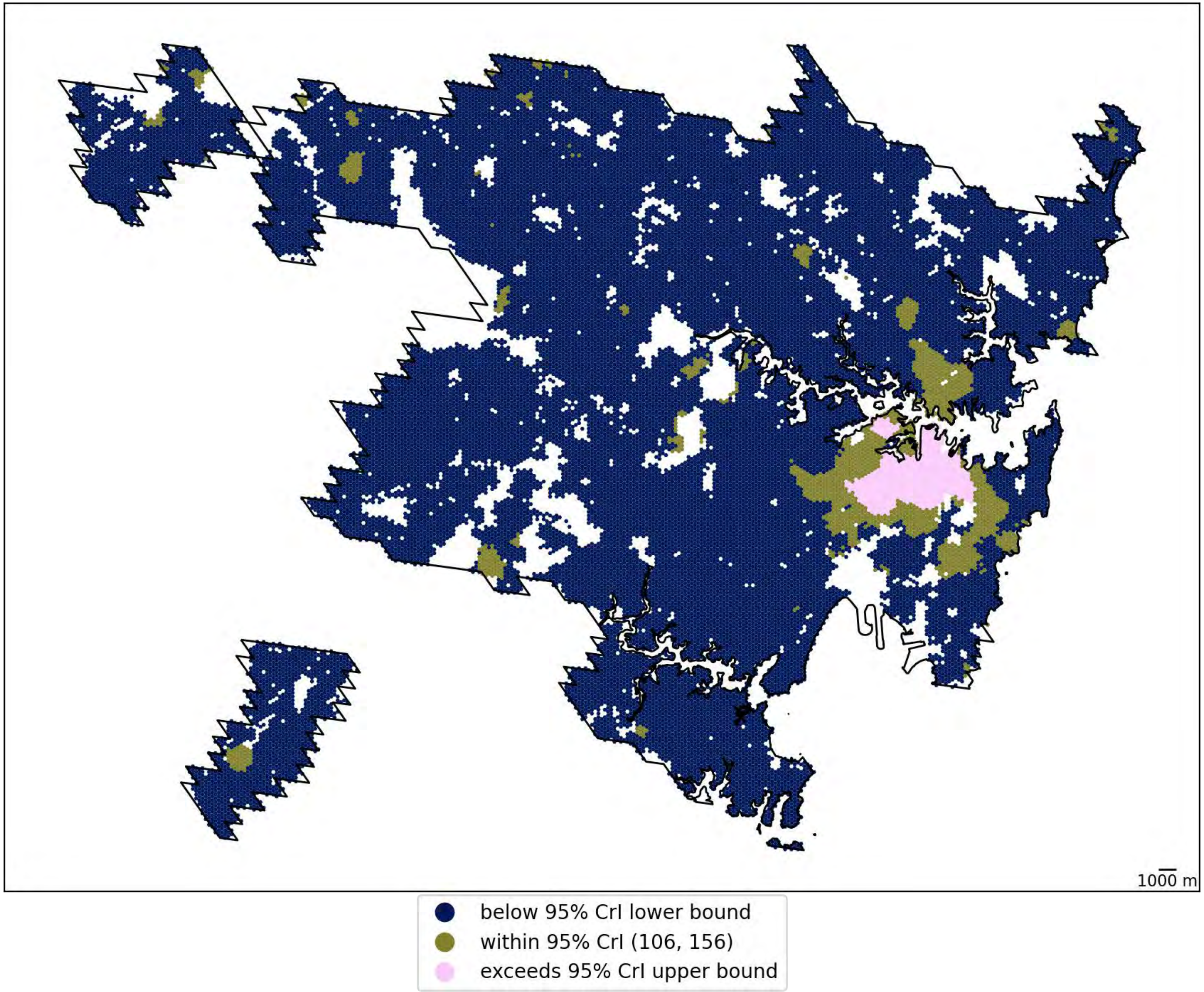

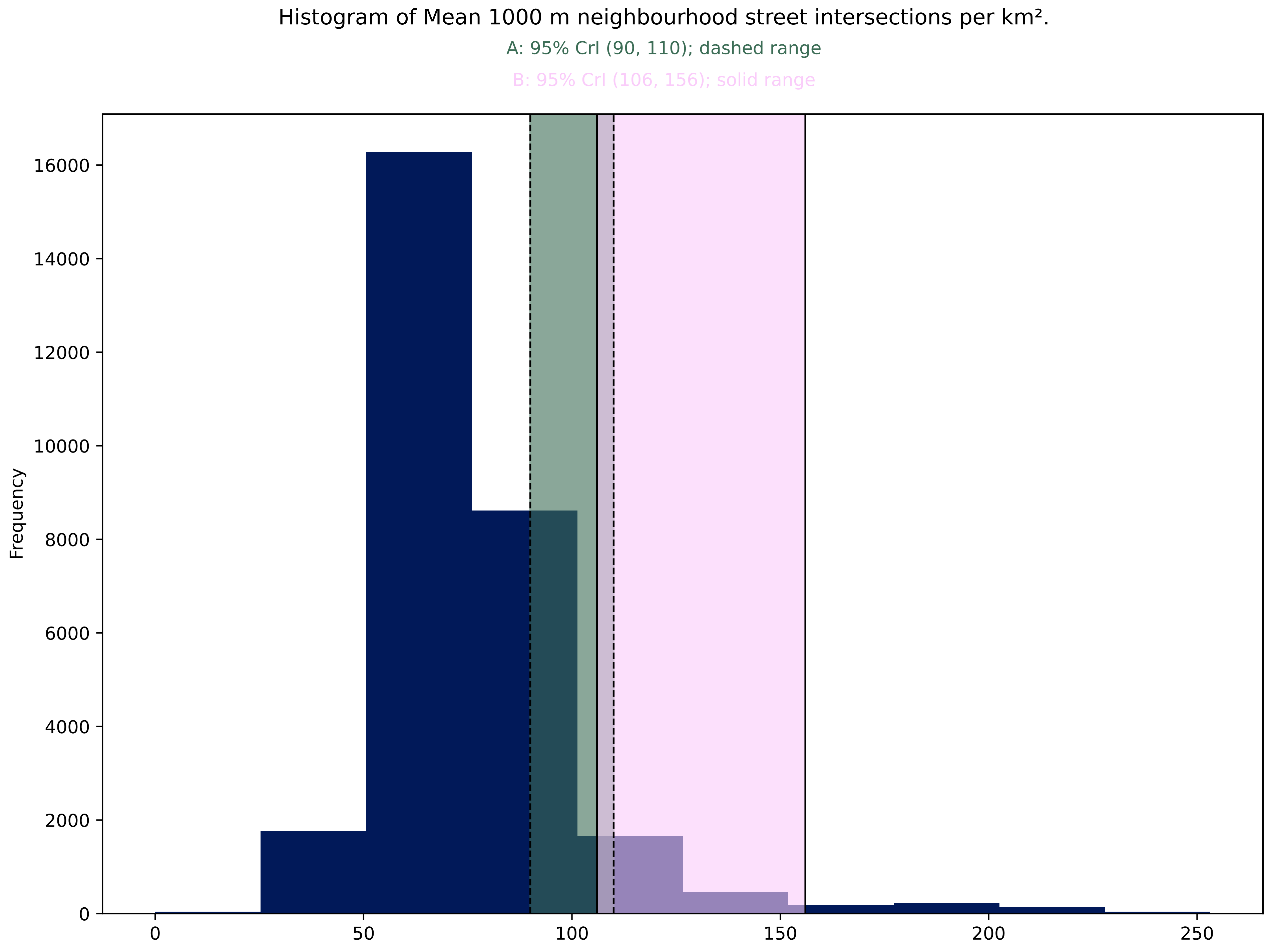
Histogram of Mean 1000 m neighbourhood street intersections per km².
A: 95% CrI (90, 110); dashed range
B: 95% CrI (106, 156); solid range
Frequency
0
2000
4000
6000
8000
10000
12000
14000
16000
0
50
100
150
200
250

Distance to nearest public transport stops (m; up to 500m)

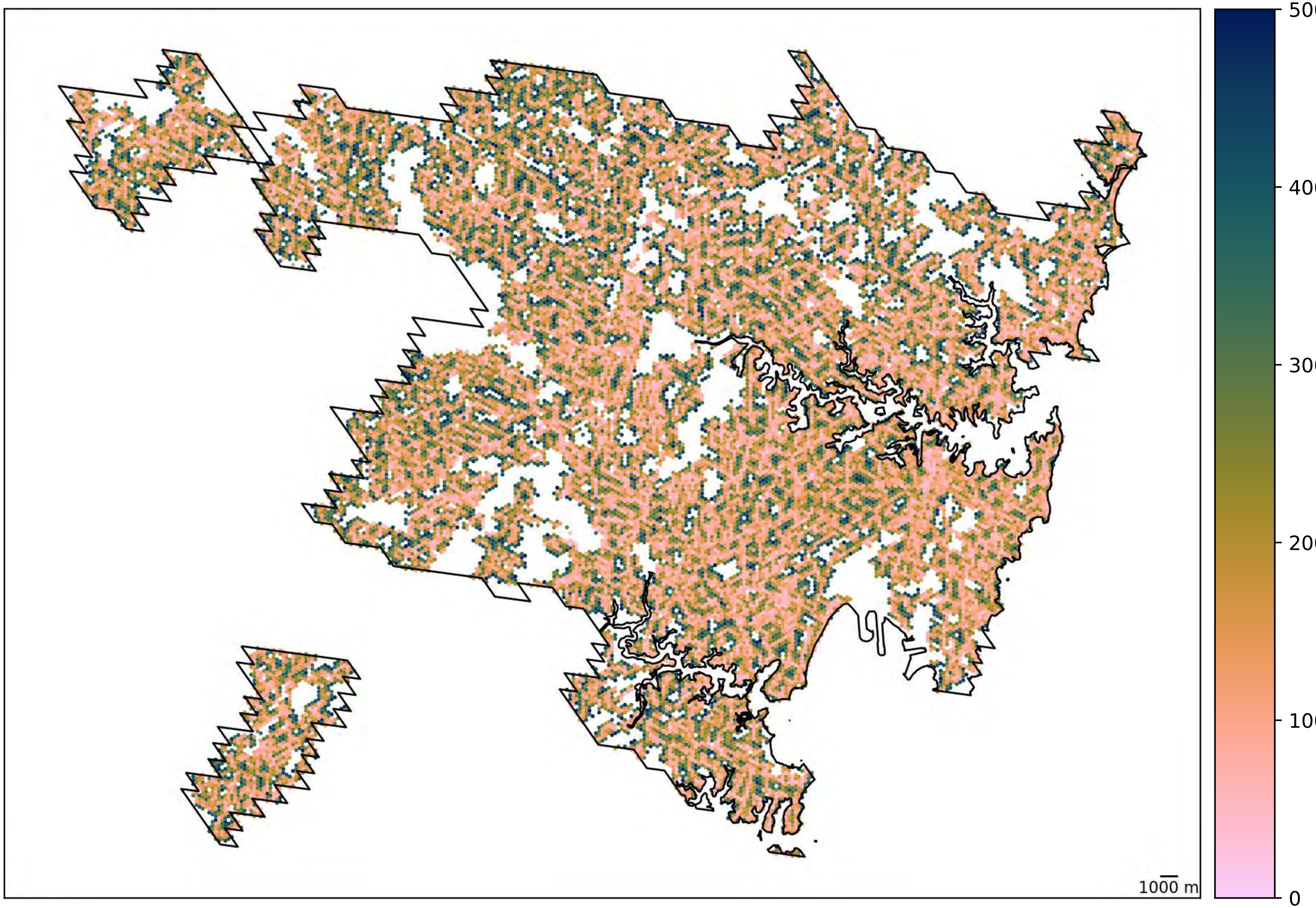

distances: Estimated Distance to nearest public transport stops (m; up to 500m) requirement for distances to destinations, measured up to a maximum distance target threshold of 500 metres

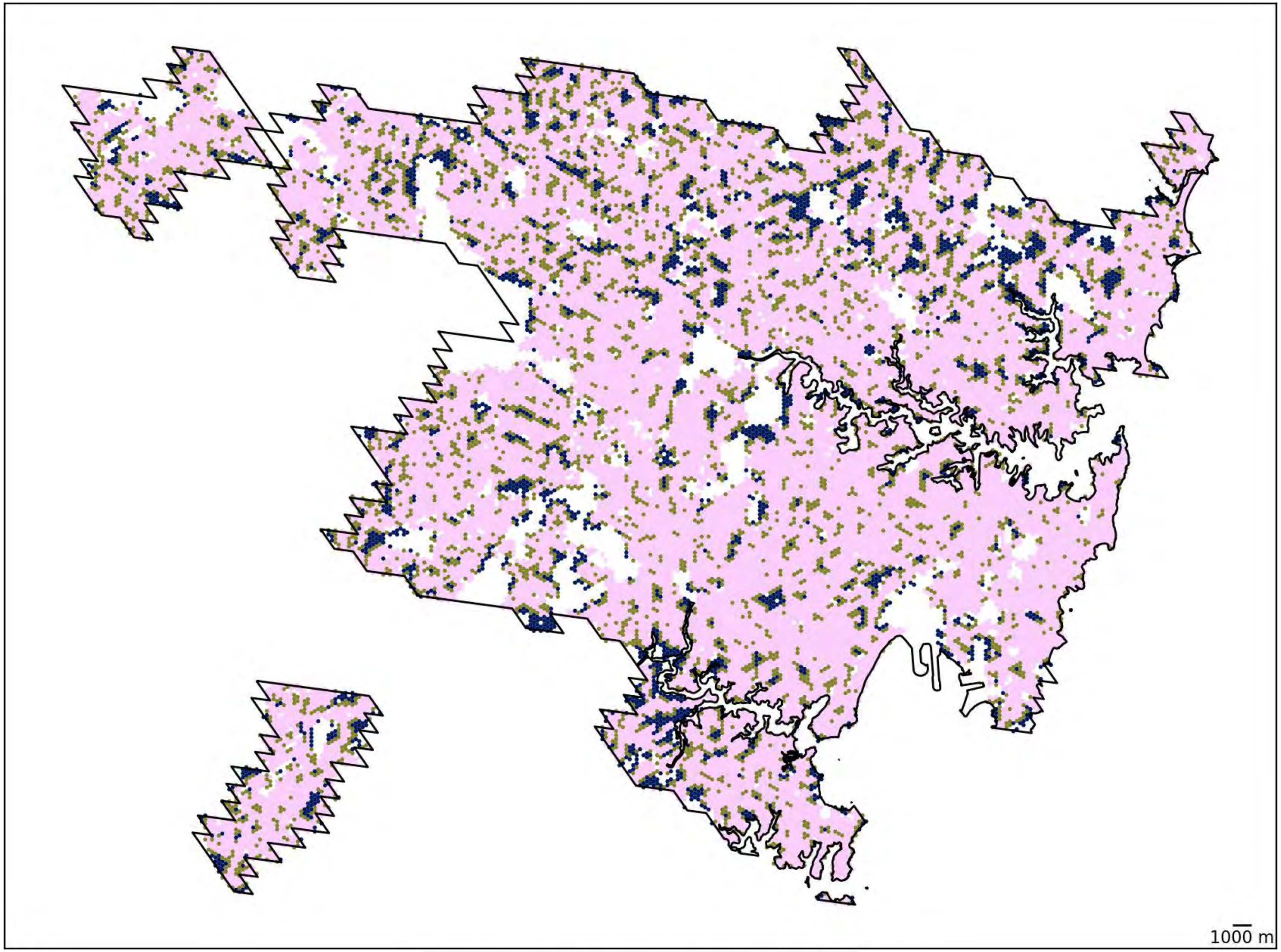

below distance (m) lower bound
within distance (m) (300, 500)
exceeds distance (m) upper bound

Histogram of Distance to nearest public transport stops (m; up to 500m).

distances: distance (m) (300, 500); dashed range

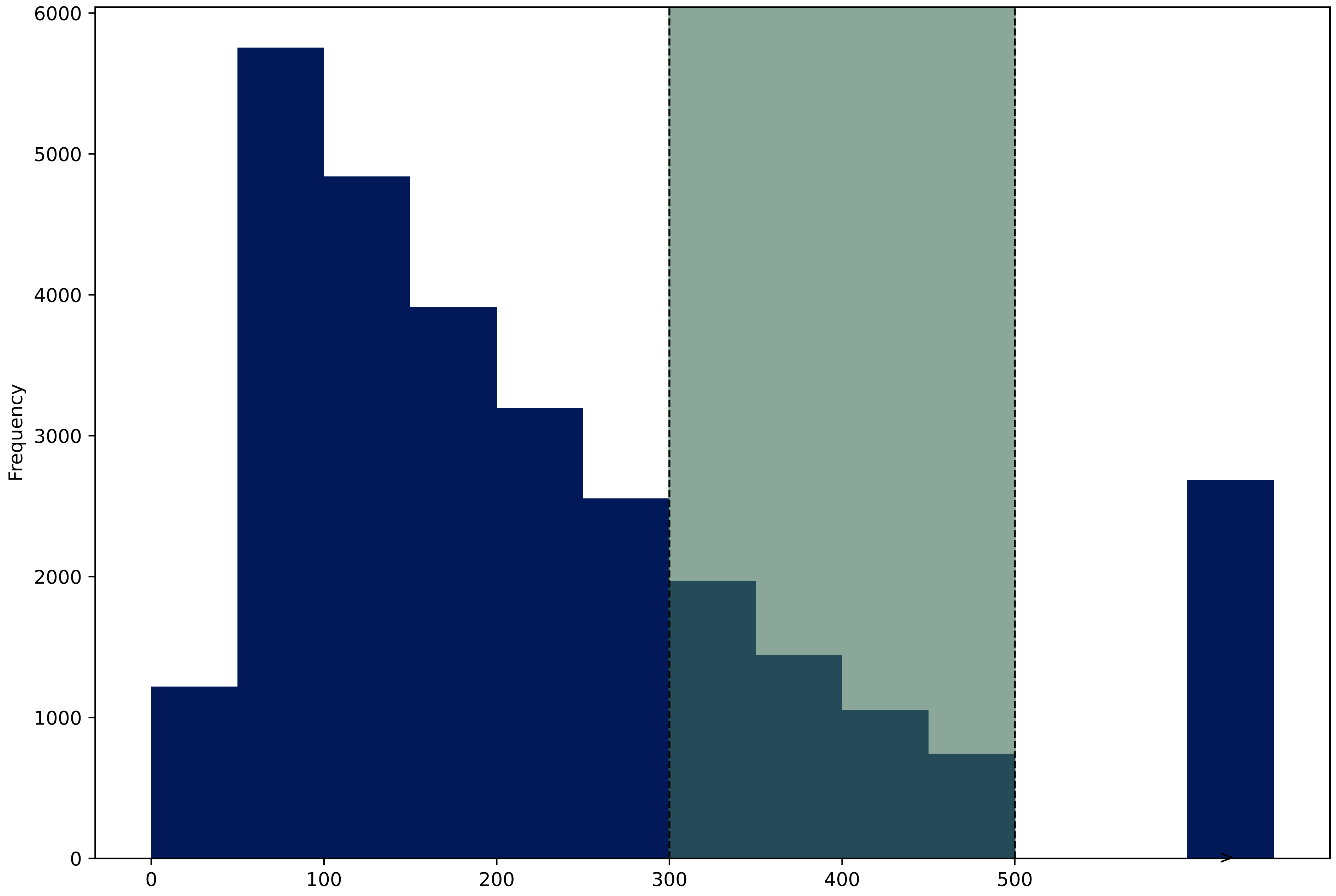

Distance to nearest park (m; up to 500m)

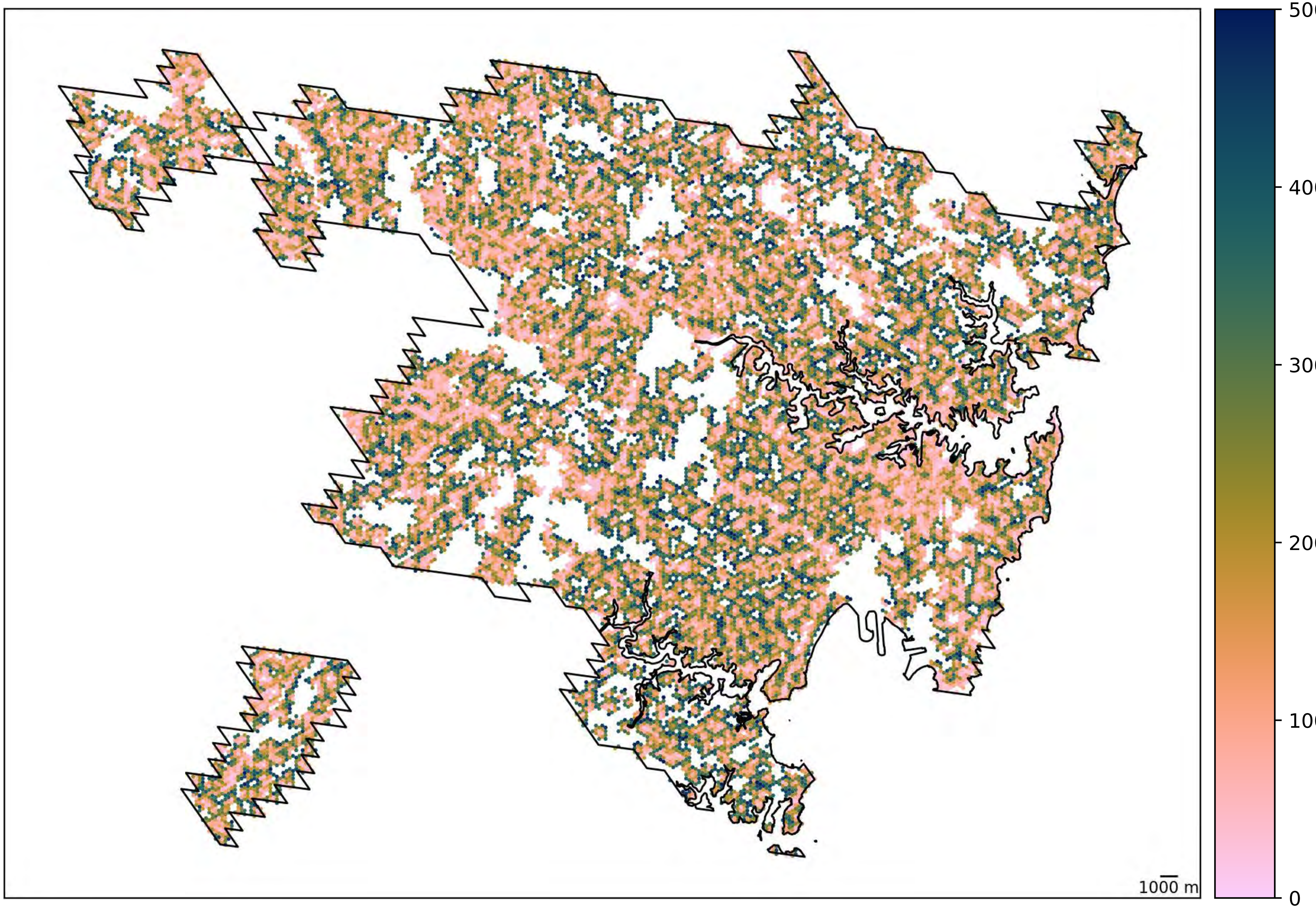

distances: Estimated Distance to nearest park (m; up to 500m) requirement for distances to destinations, measured up to a maximum distance target threshold of 500 metres

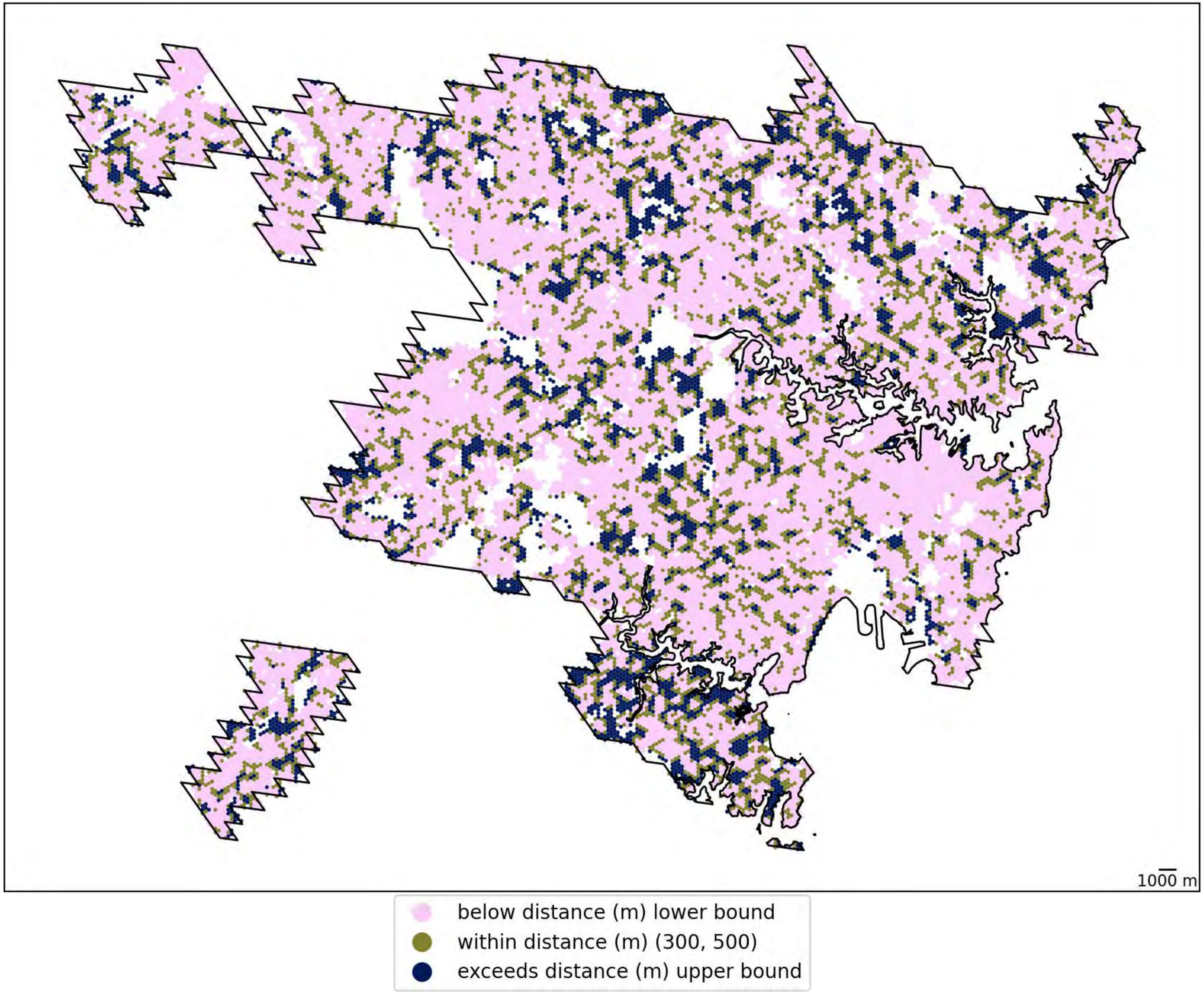

Histogram of Distance to nearest park (m; up to 500m).

distances: distance (m) (300, 500); dashed range

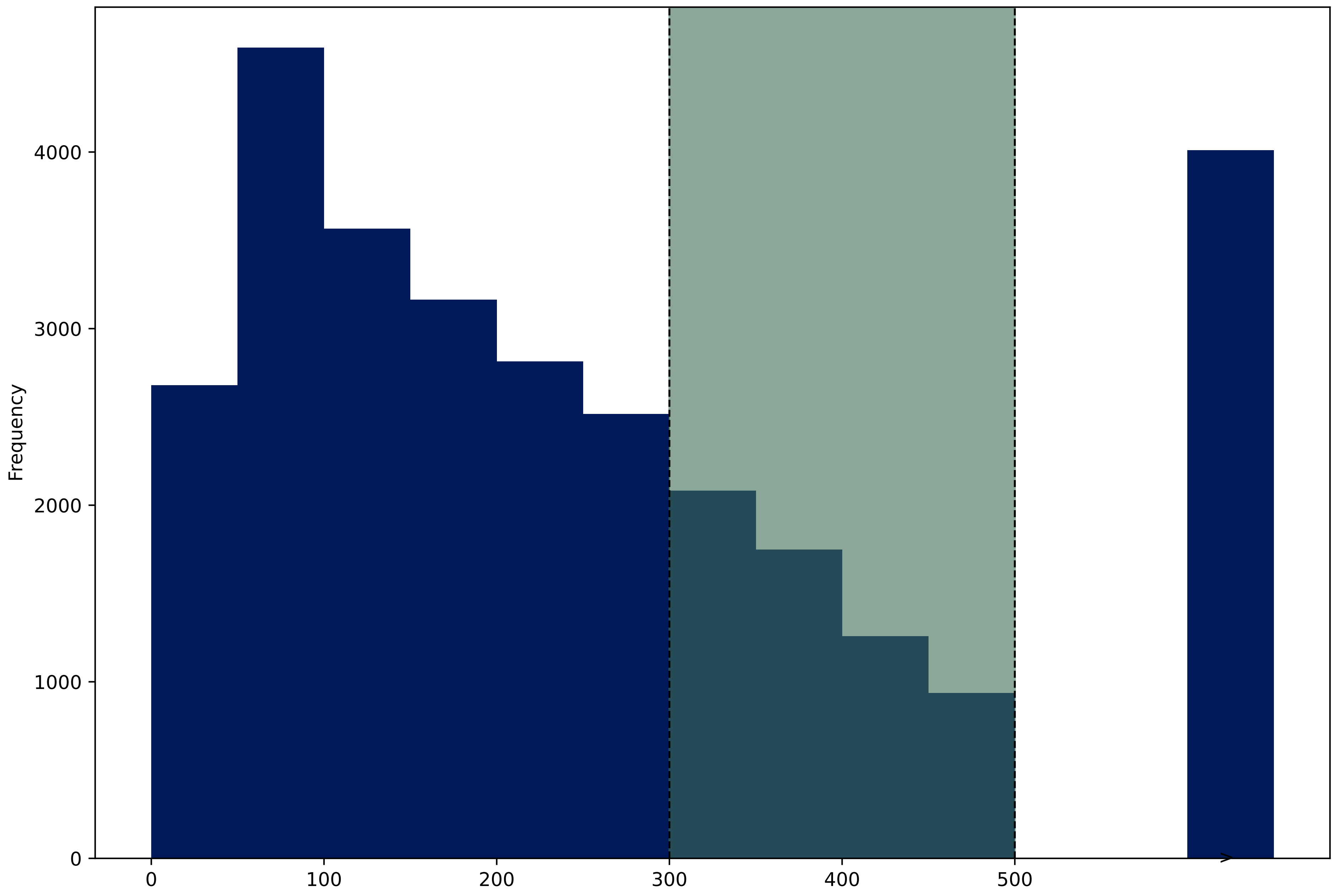

Australasia, New Zealand, Auckland

Satellite imagery of urban study region (Bing)

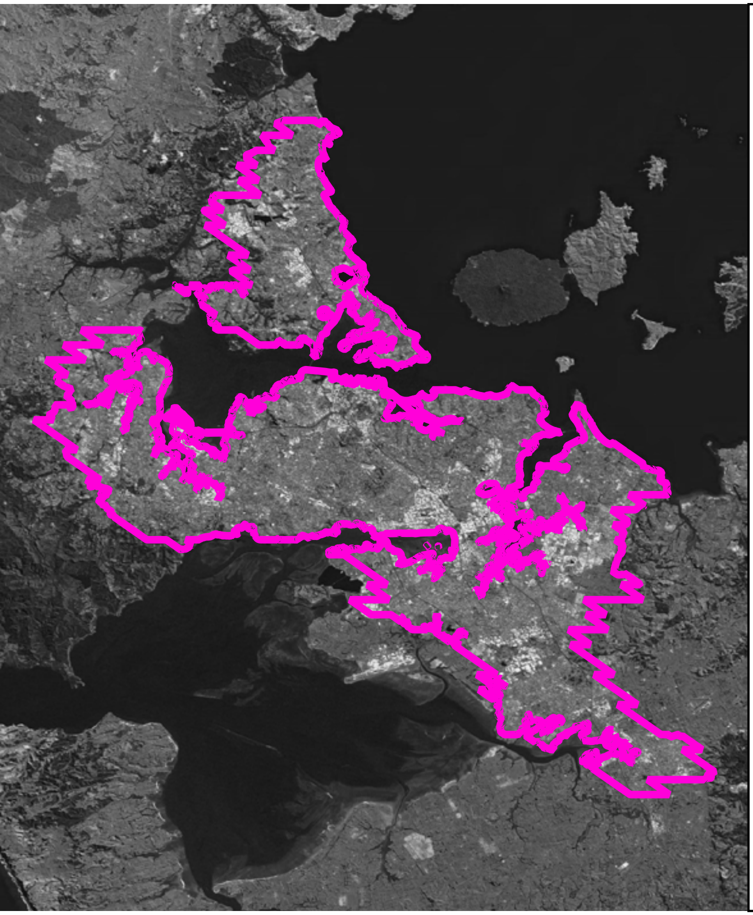

Urban boundary

Walkability, relative to city

Walkability, relative to 25 global cities

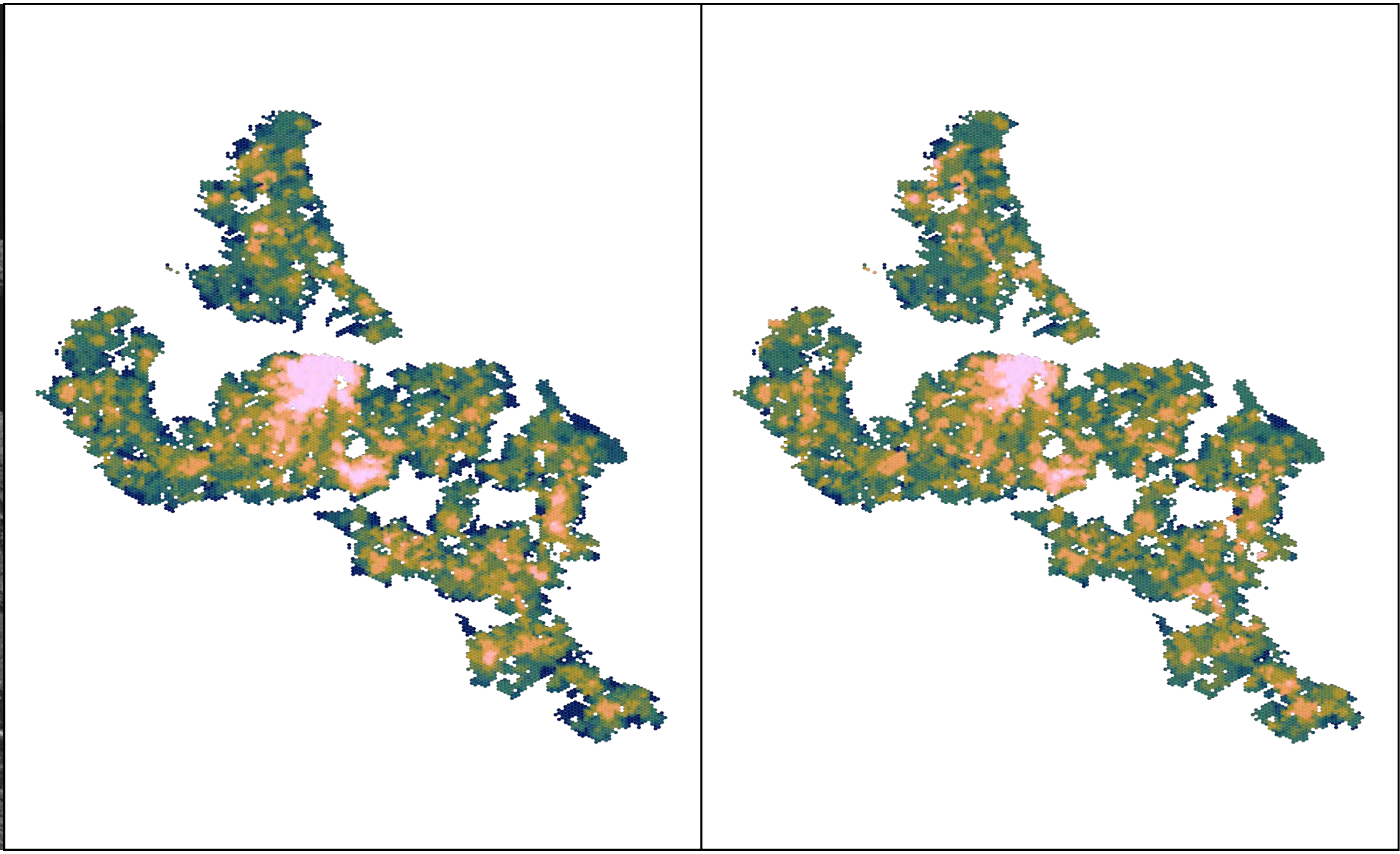

0 10 20 km

Walkability score

- <-3
- -3 to -2
- -2 to -1
- -1 to 0
- 0 to 1
- 1 to 2
- 2 to 3
- ≥3

Walkability relative to all cities by component variables (2D histograms), and overall (histogram)

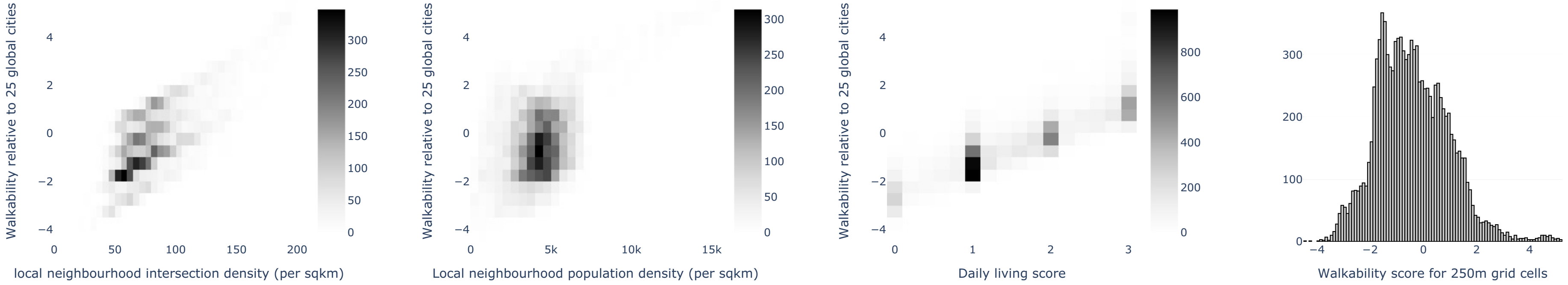

Mean 1000 m neighbourhood population per km²

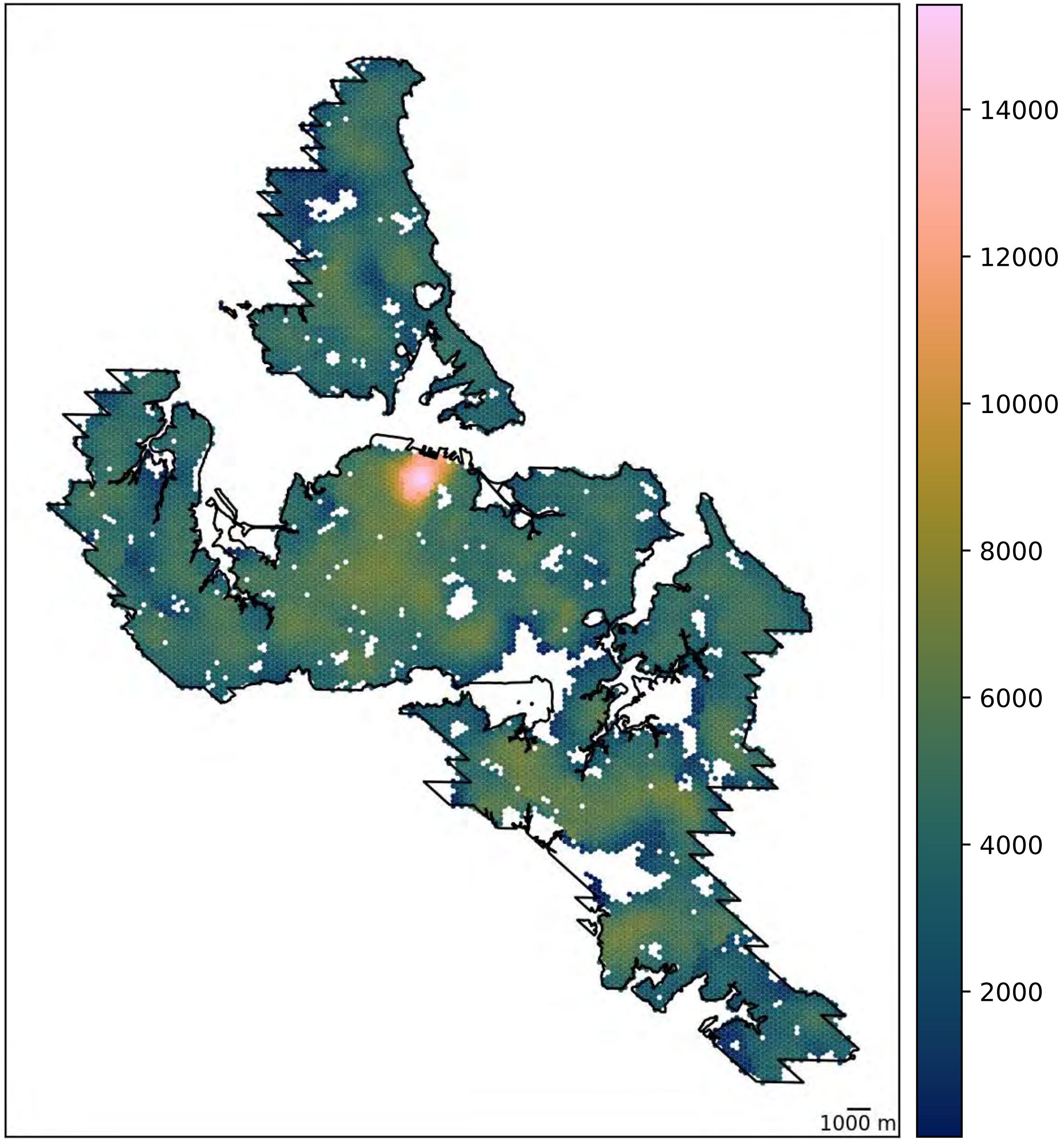

A: Estimated Mean 1000 m neighbourhood population per km² requirement for ≥80% probability of engaging in walking for transport

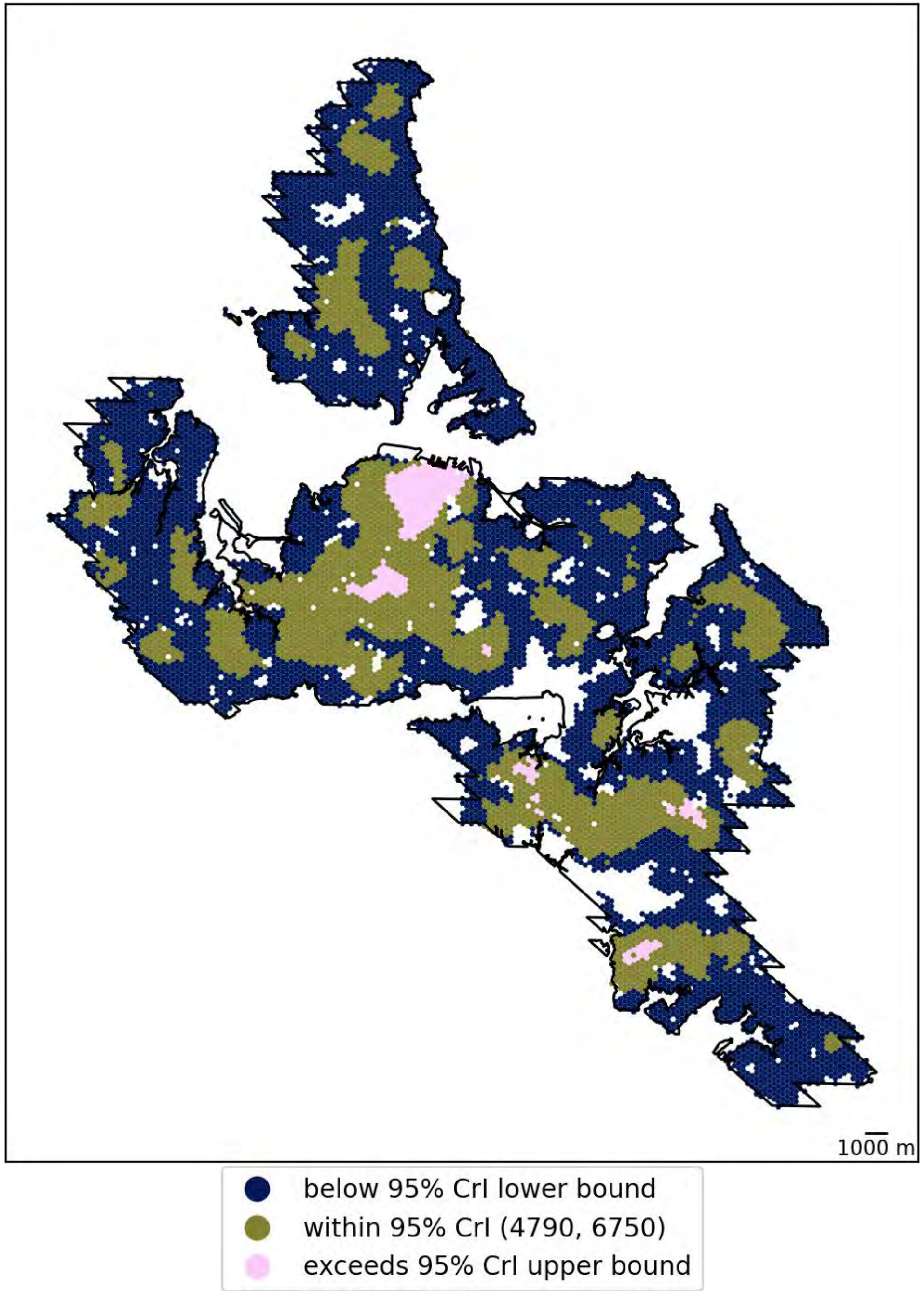

B: Estimated Mean 1000 m neighbourhood population per km² requirement for reaching the WHO's target of a ≥15% relative reduction in insufficient physical activity through walking

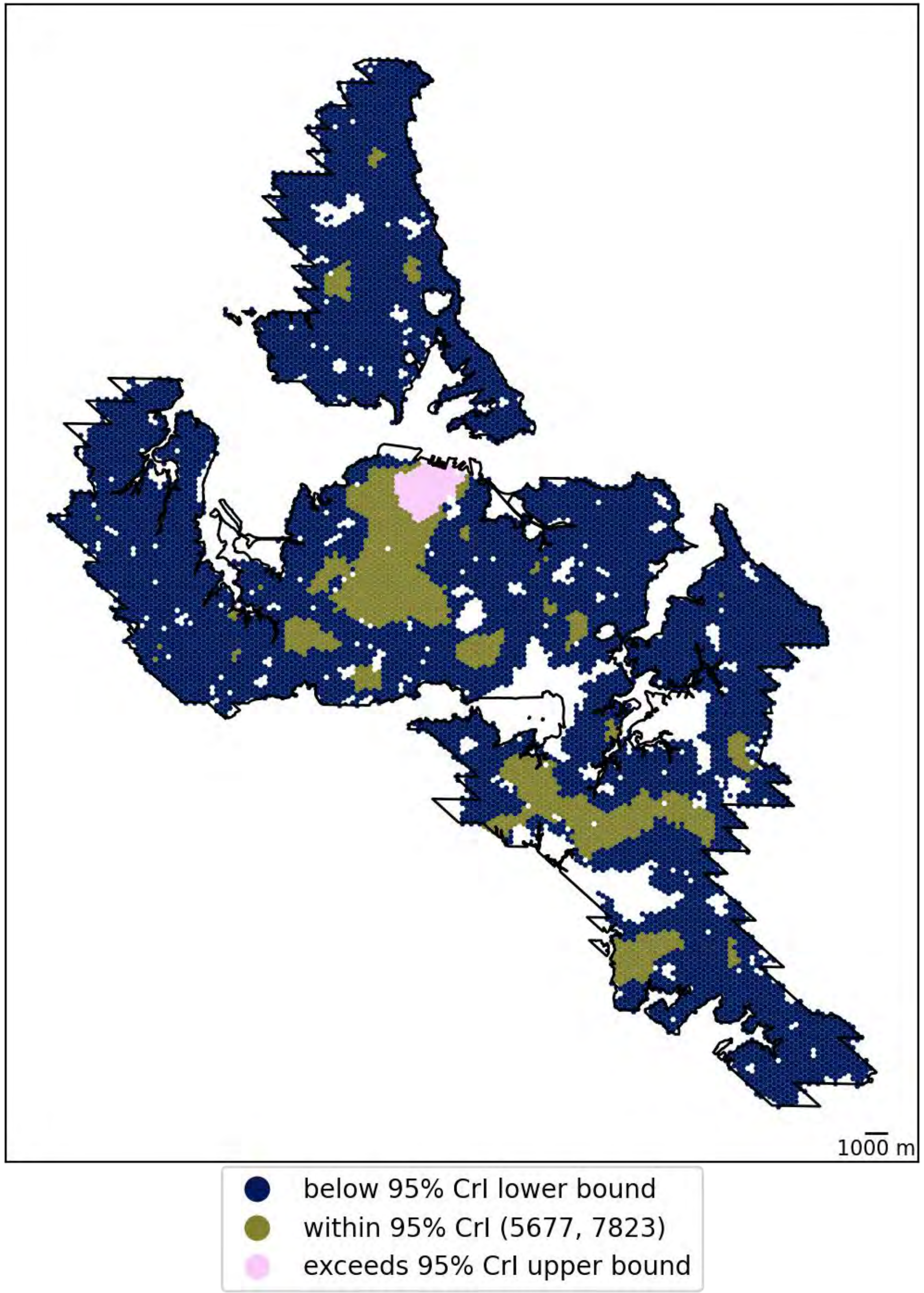

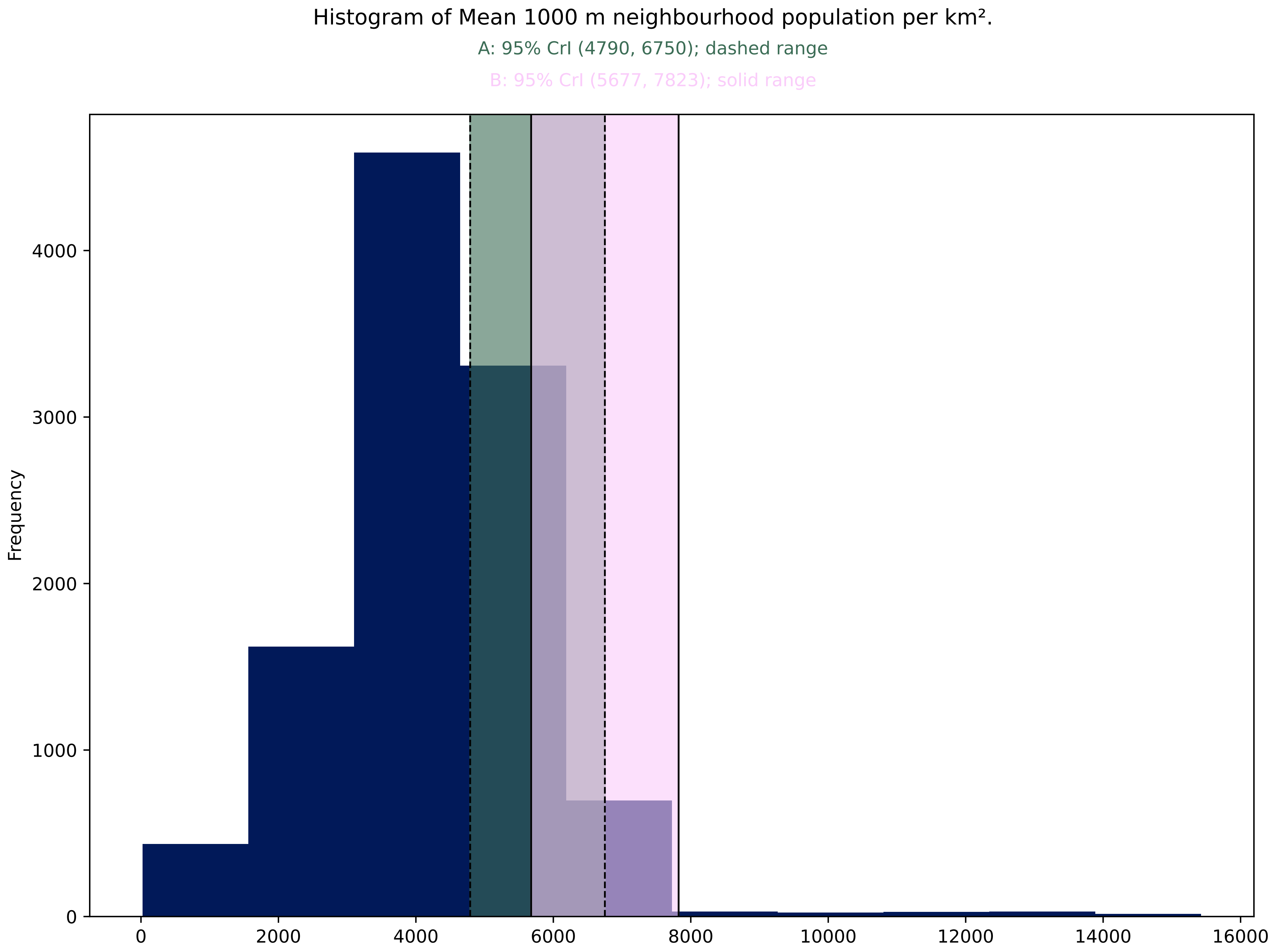
Histogram of Mean 1000 m neighbourhood population per km².
A: 95% CrI (4790, 6750); dashed range
B: 95% CrI (5677, 7823); solid range
Frequency
0
1000
2000
3000
4000
0
2000
4000
6000
8000
10000
12000
14000
16000

Mean 1000 m neighbourhood street intersections per km²

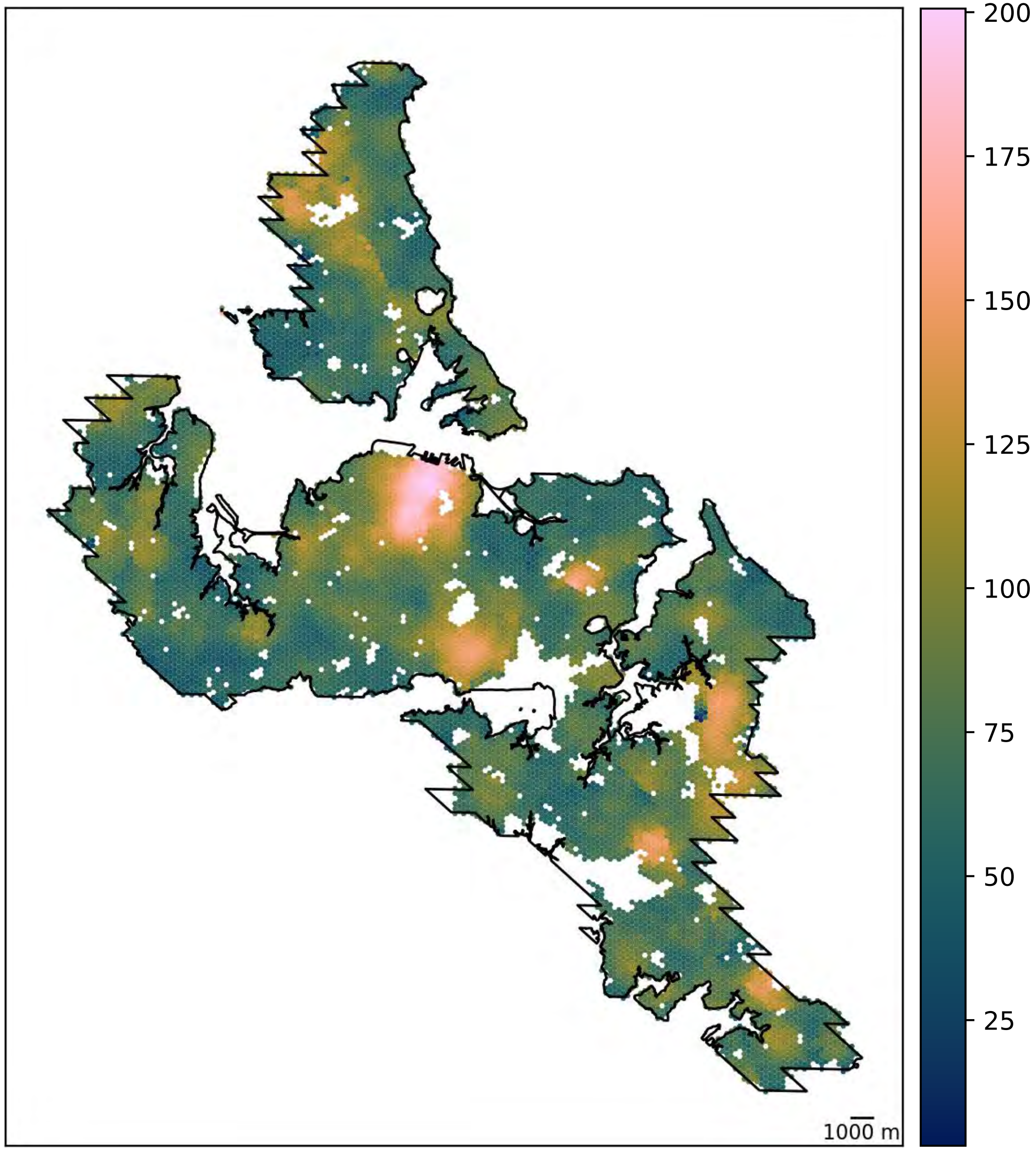

A: Estimated Mean 1000 m neighbourhood street intersections per km² requirement for ≥80% probability of engaging in walking for transport

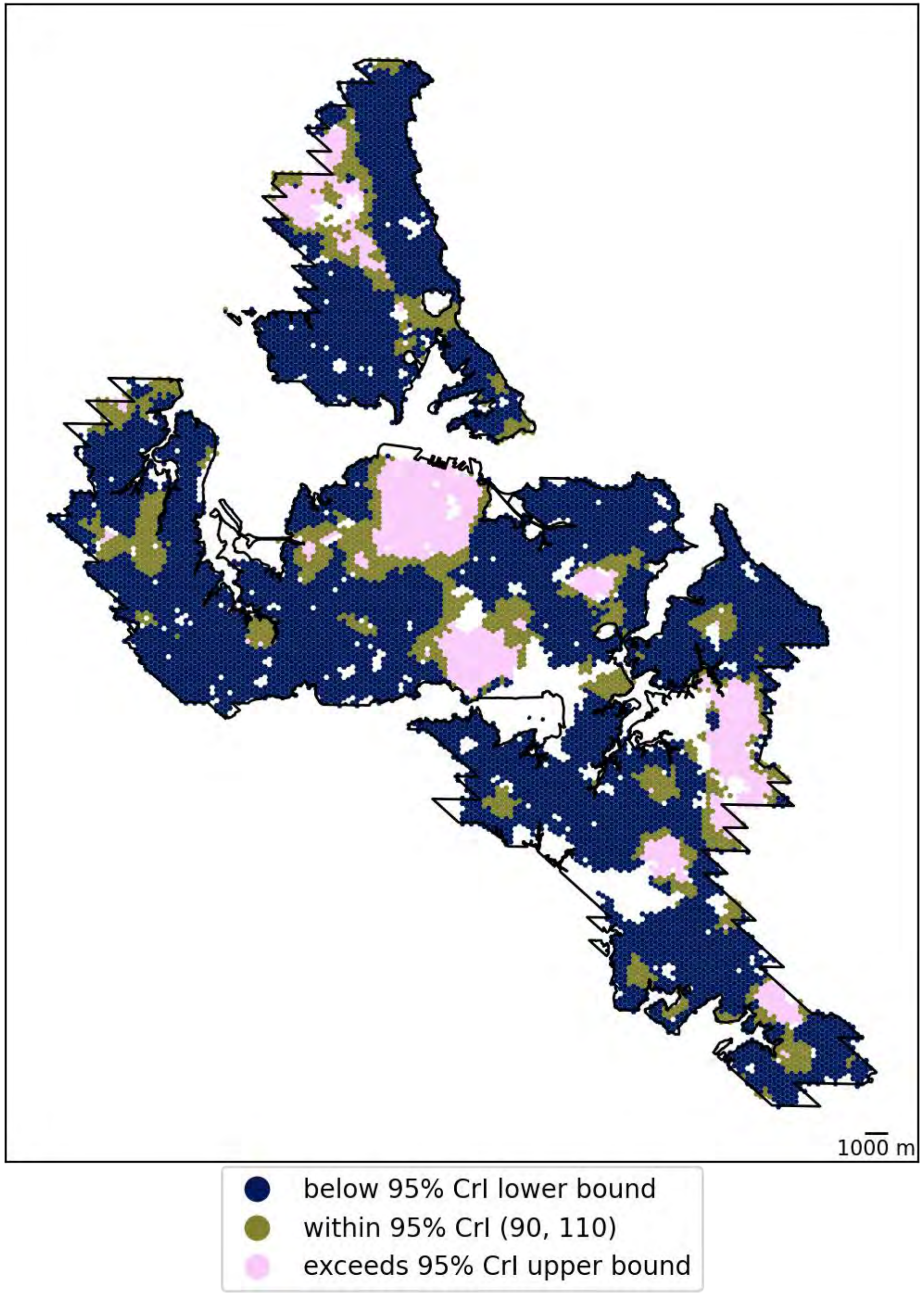

B: Estimated Mean 1000 m neighbourhood street intersections per km² requirement for reaching the WHO's target of a ≥15% relative reduction in insufficient physical activity through walking

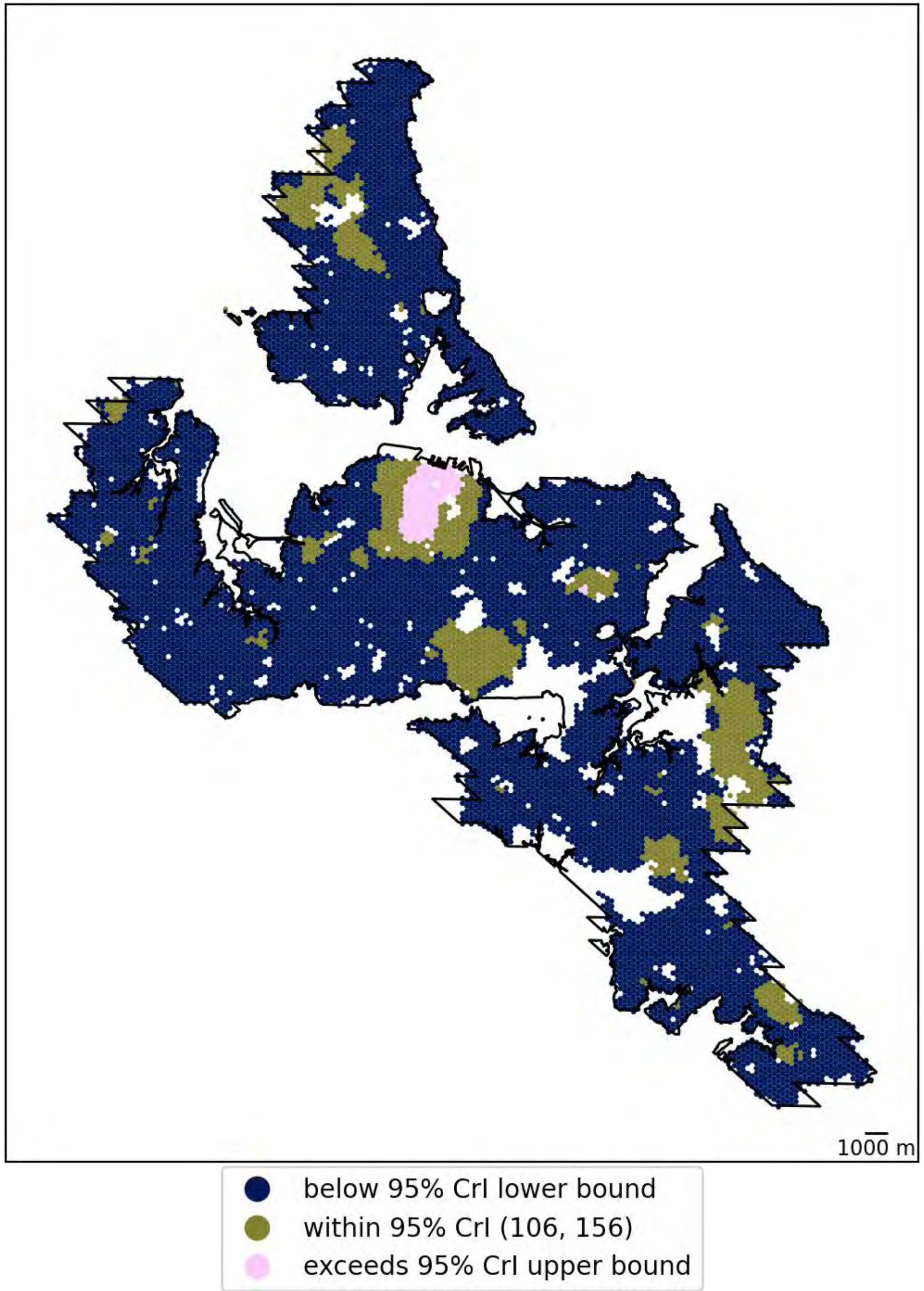

Histogram of Mean 1000 m neighbourhood street intersections per km².

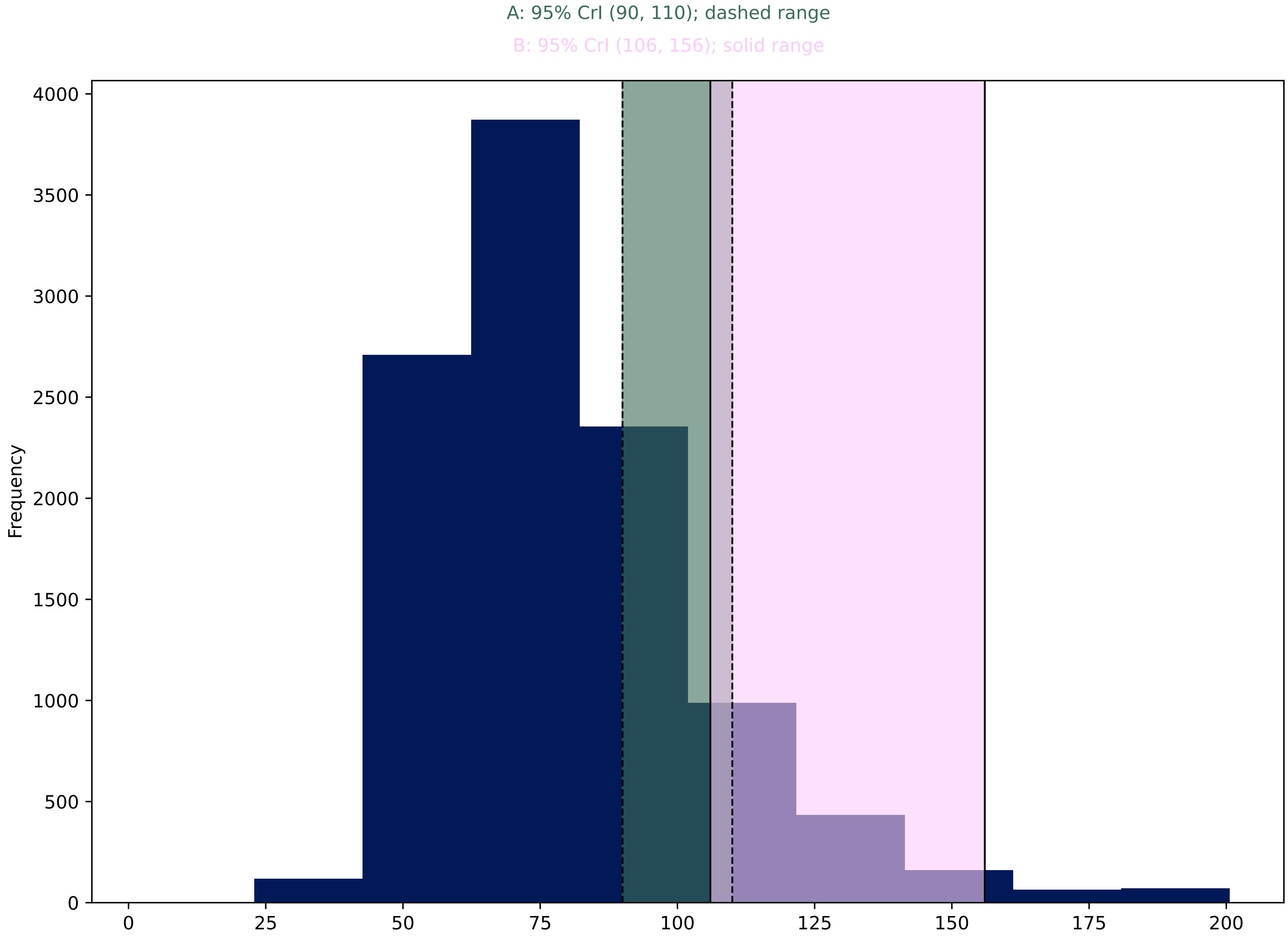

Distance to nearest public transport stops (m; up to 500m)

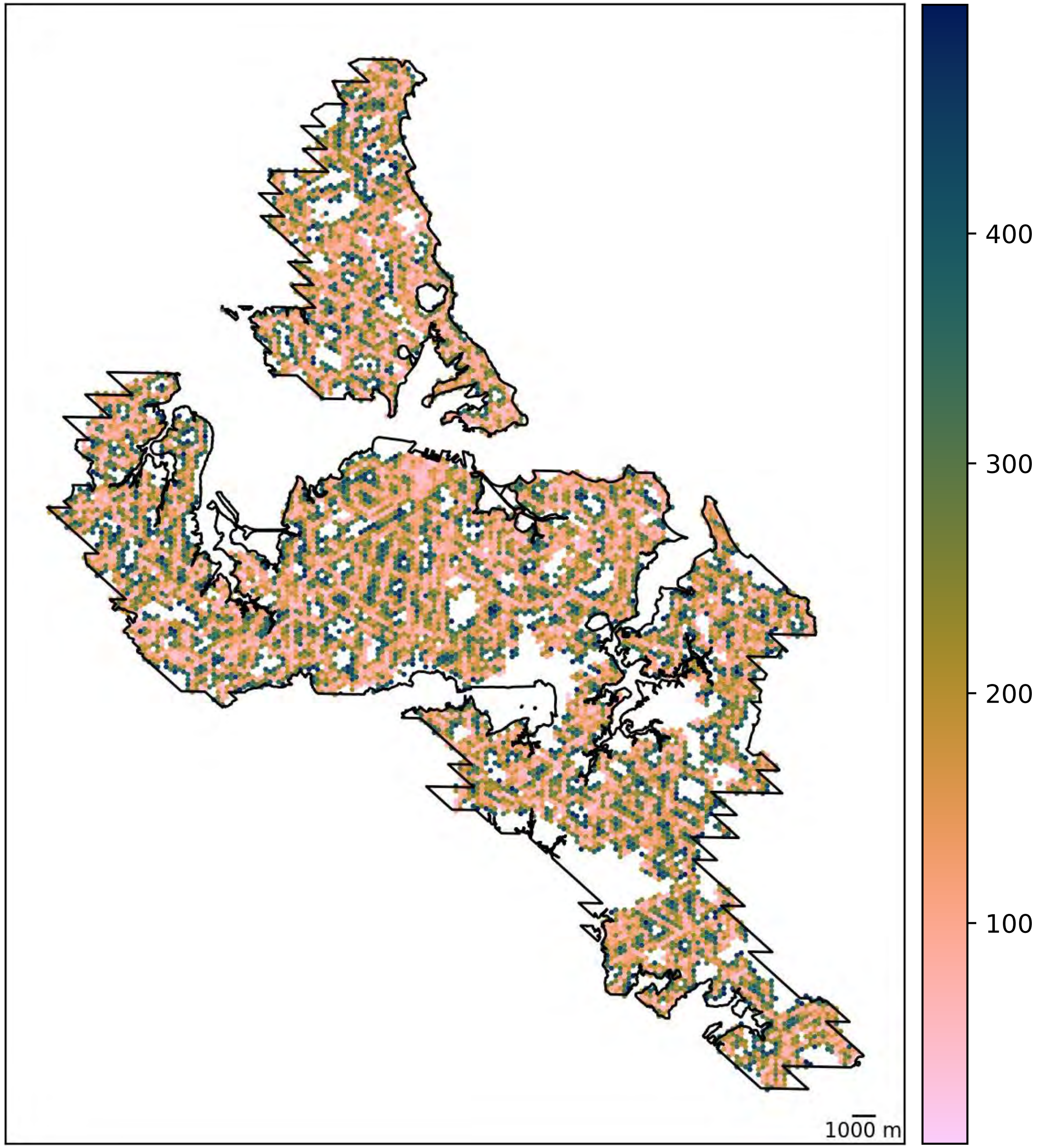

distances: Estimated Distance to nearest public transport stops (m; up to 500m) requirement for distances to destinations, measured up to a maximum distance target threshold of 500 metres

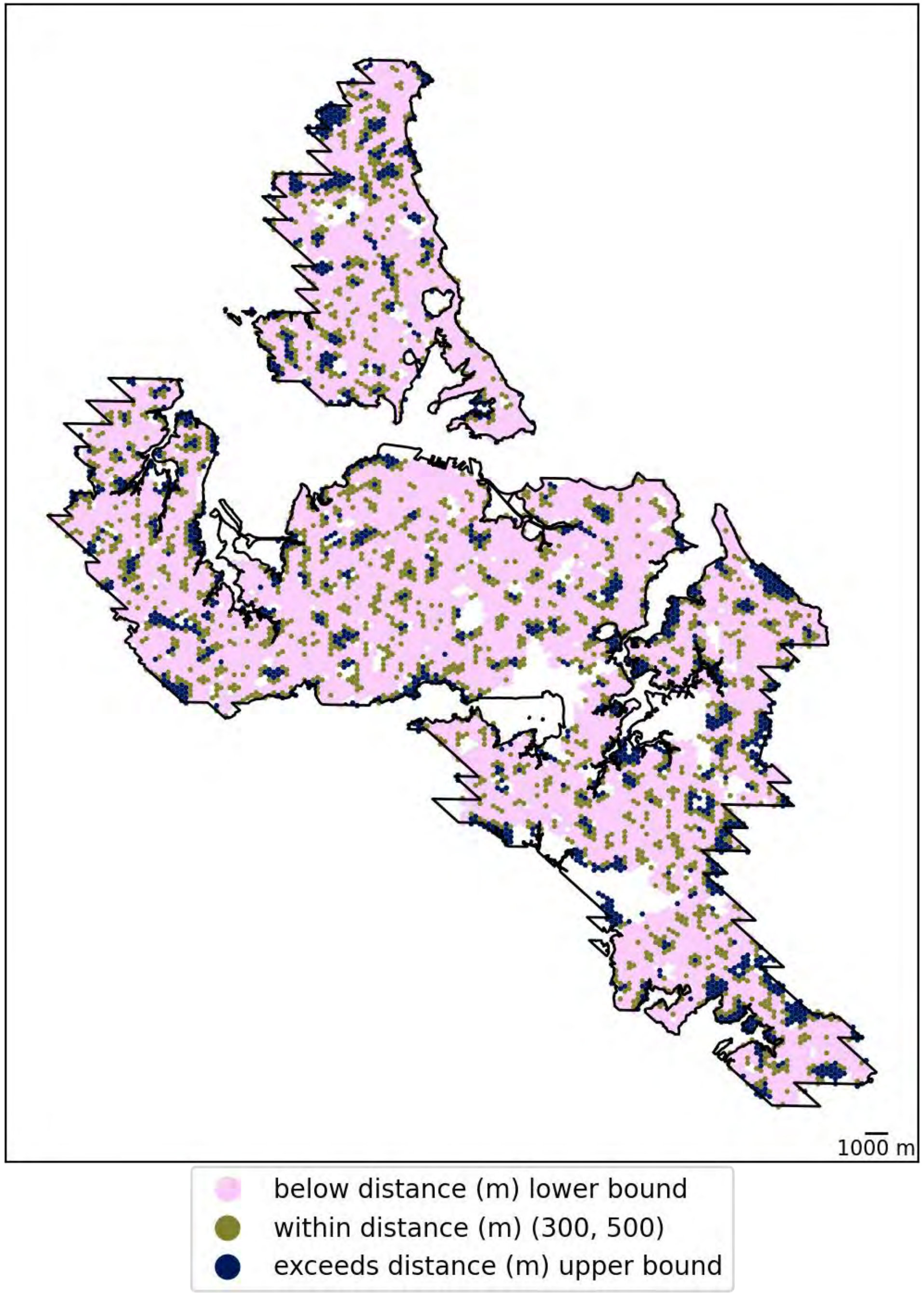

Histogram of Distance to nearest public transport stops (m; up to 500m).

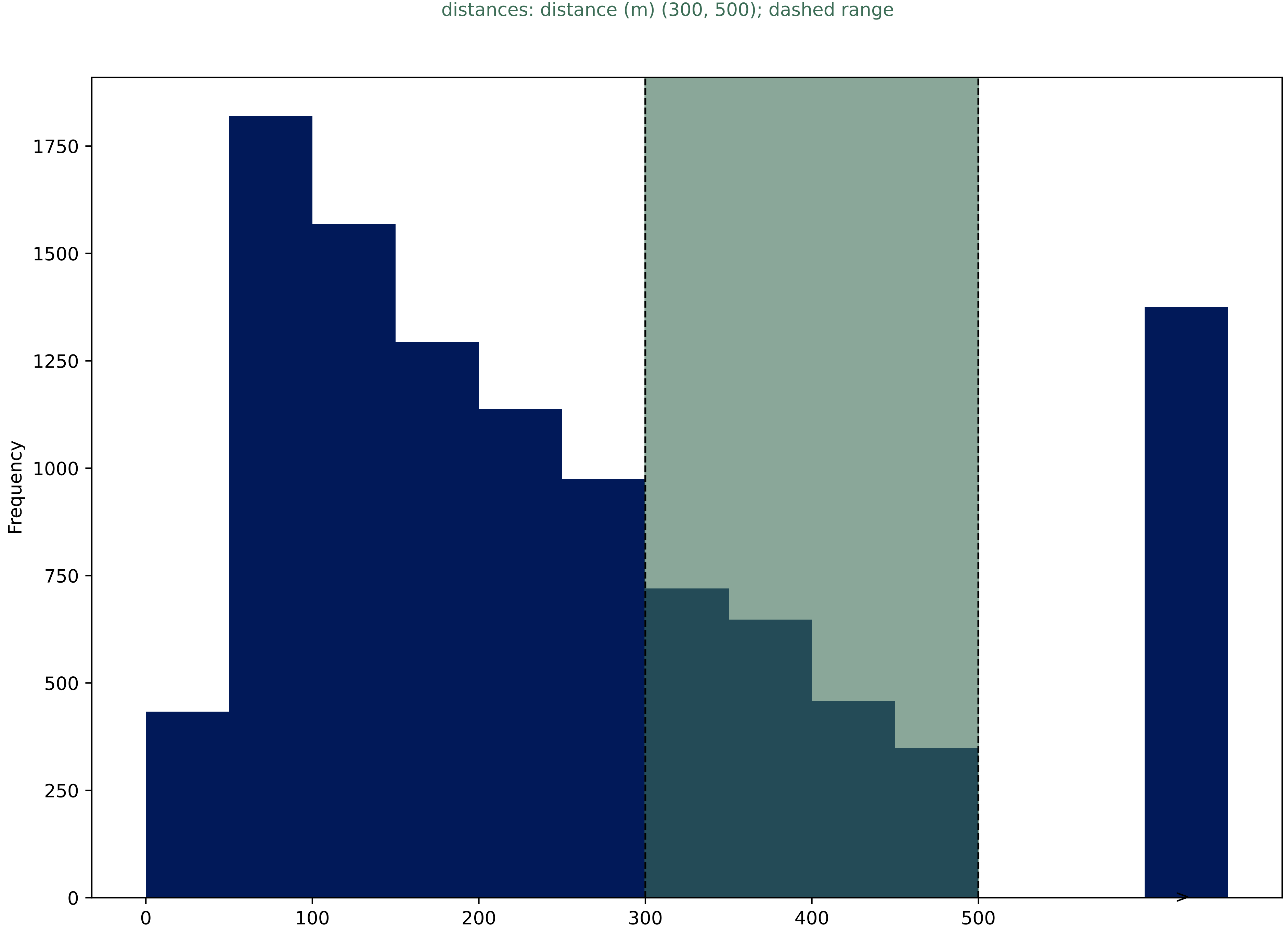

Distance to nearest park (m; up to 500m)

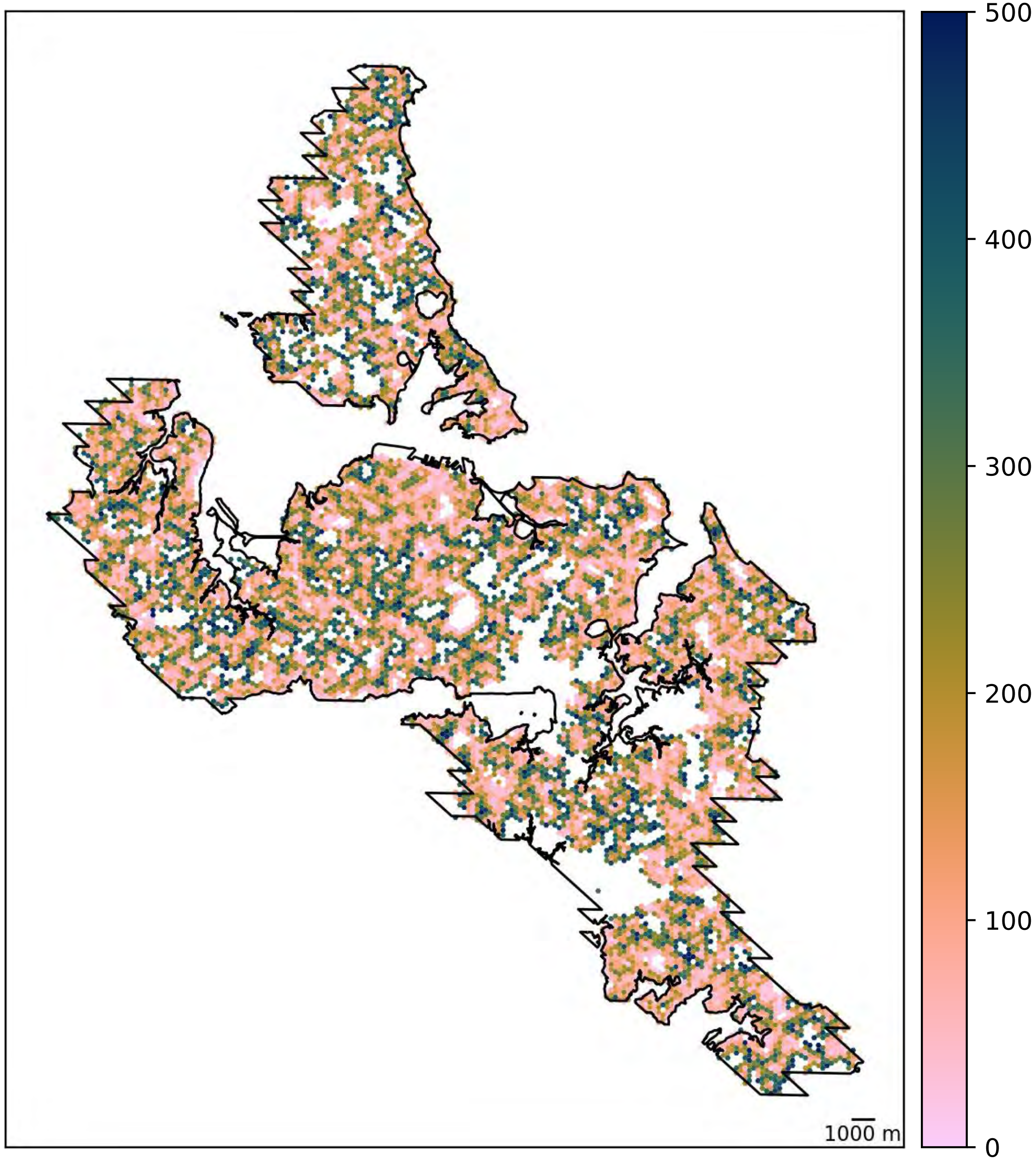

distances: Estimated Distance to nearest park (m; up to 500m) requirement for distances to destinations, measured up to a maximum distance target threshold of 500 metres

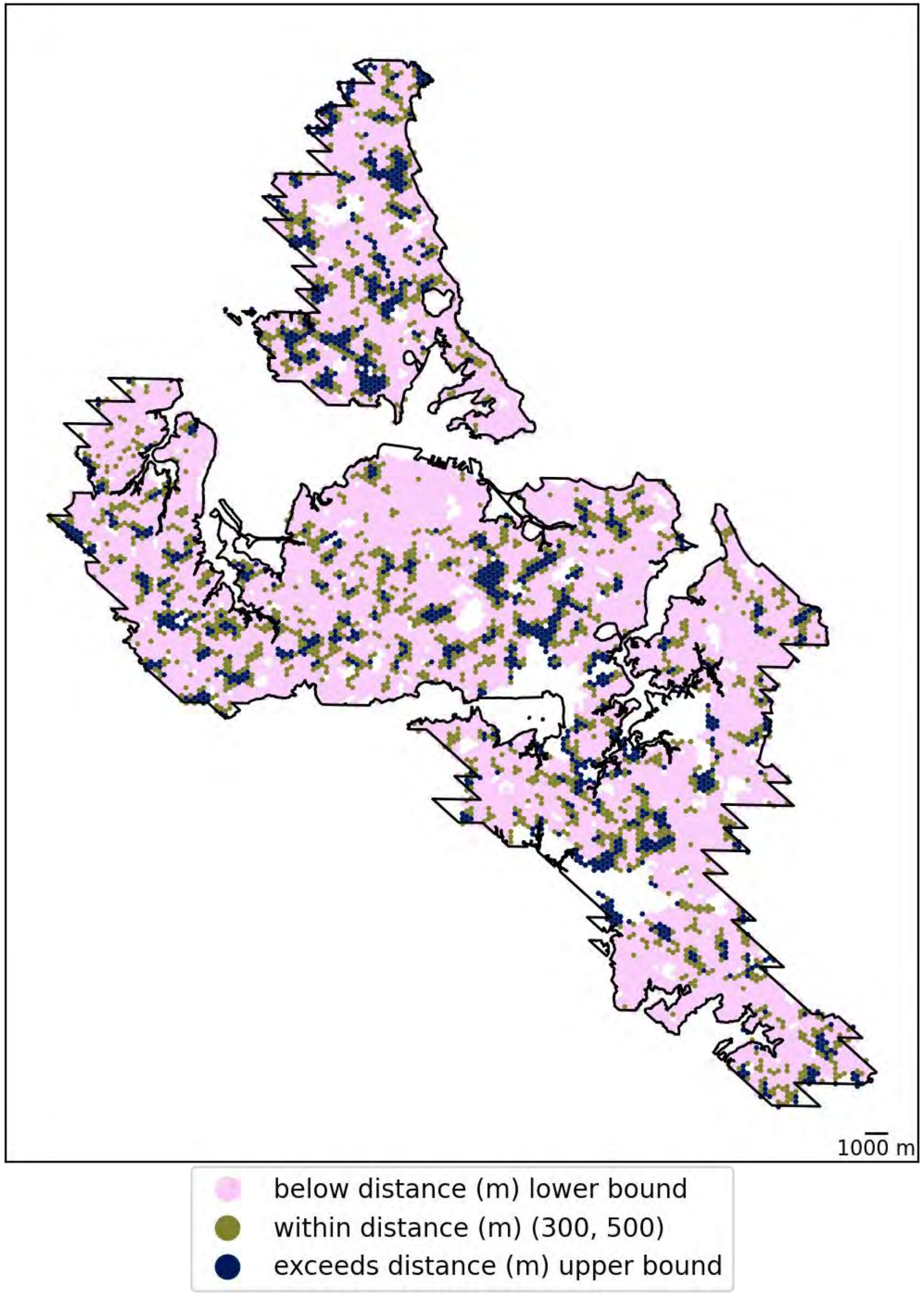

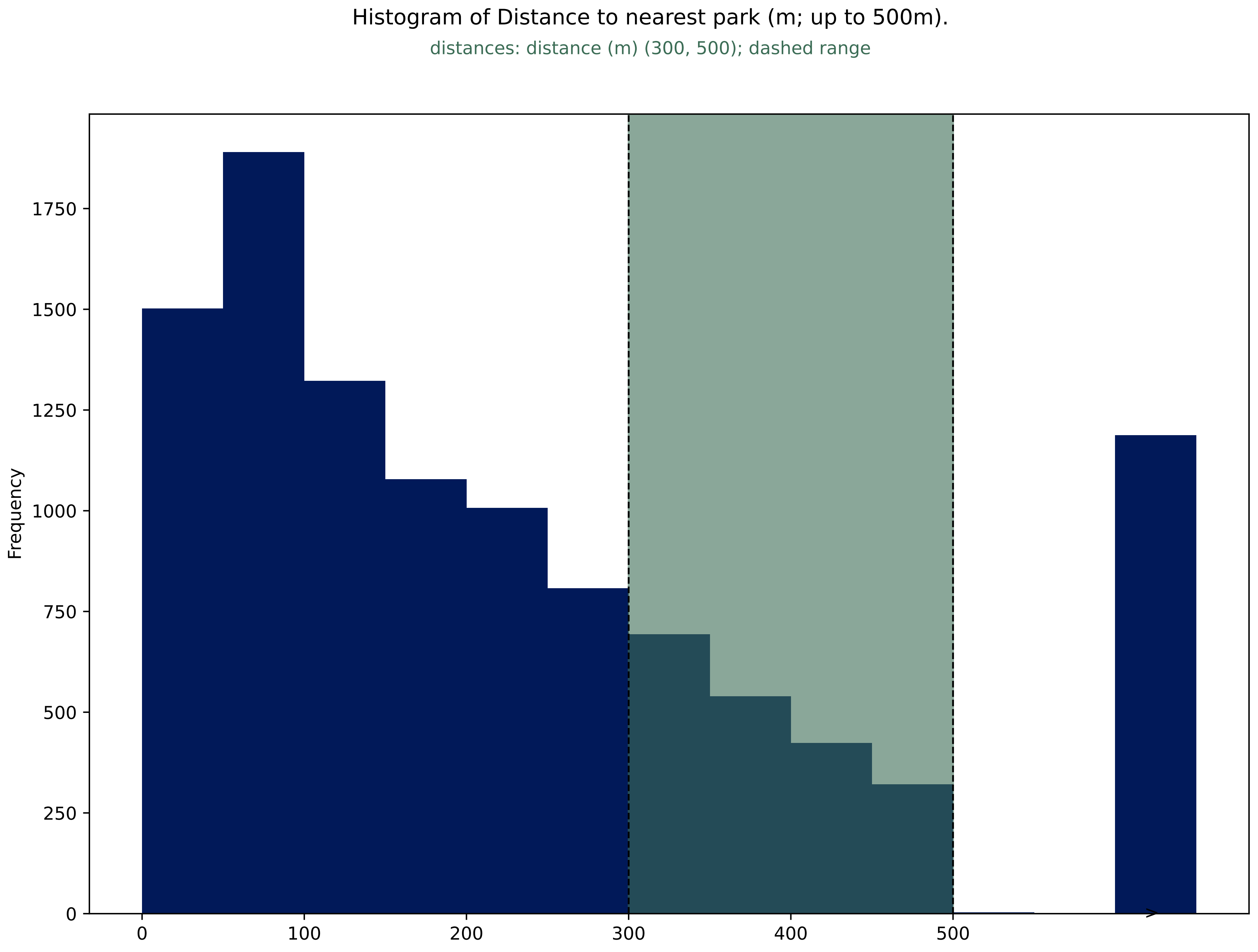
Histogram of Distance to nearest park (m; up to 500m).
distances: distance (m) (300, 500); dashed range
Frequency
0
250
500
750
1000
1250
1500
1750
0
100
200
300
400
500

Europe, Austria, Graz

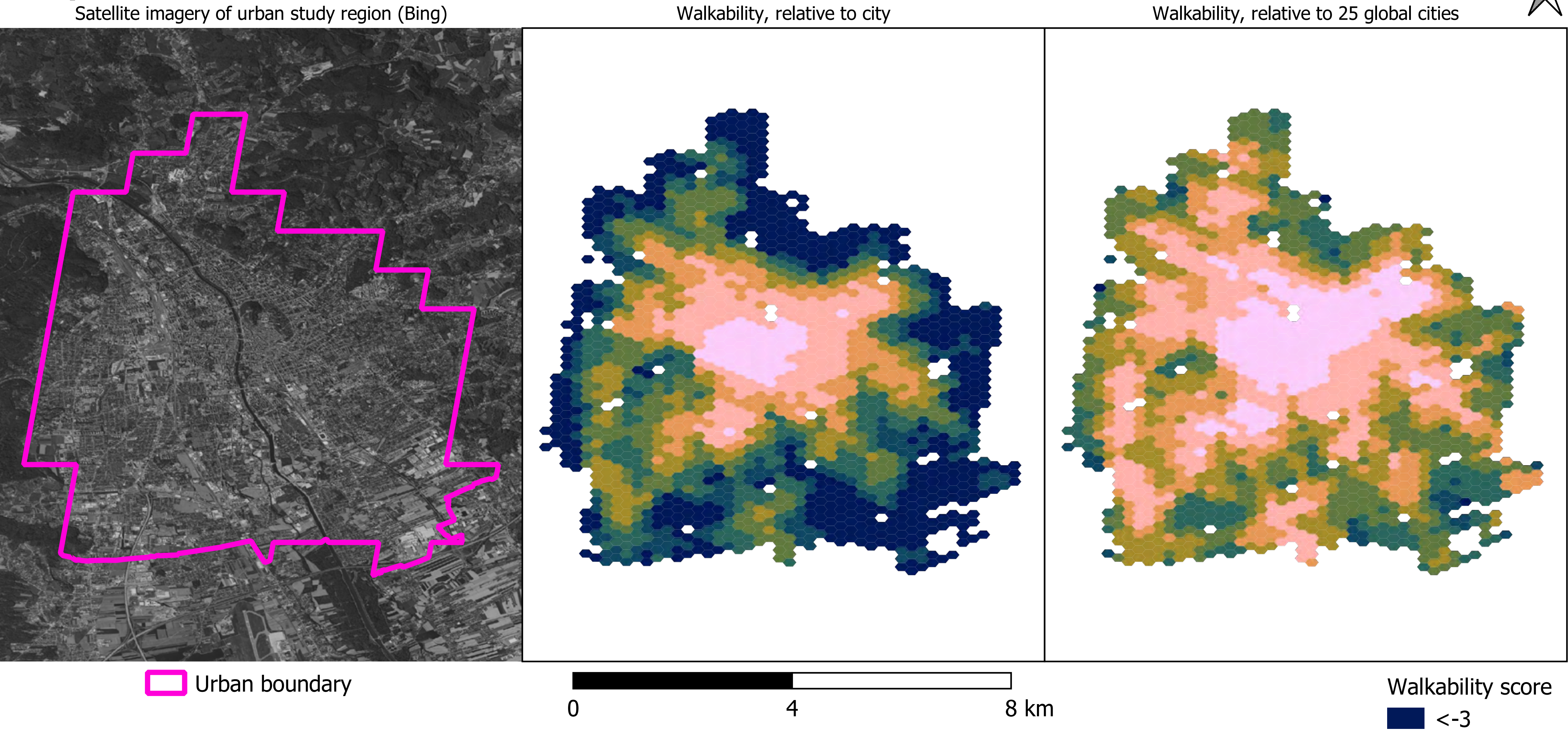

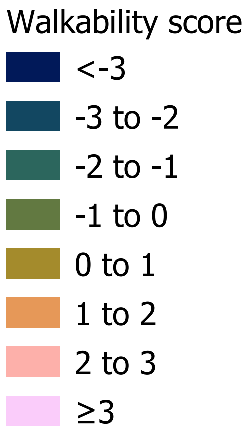

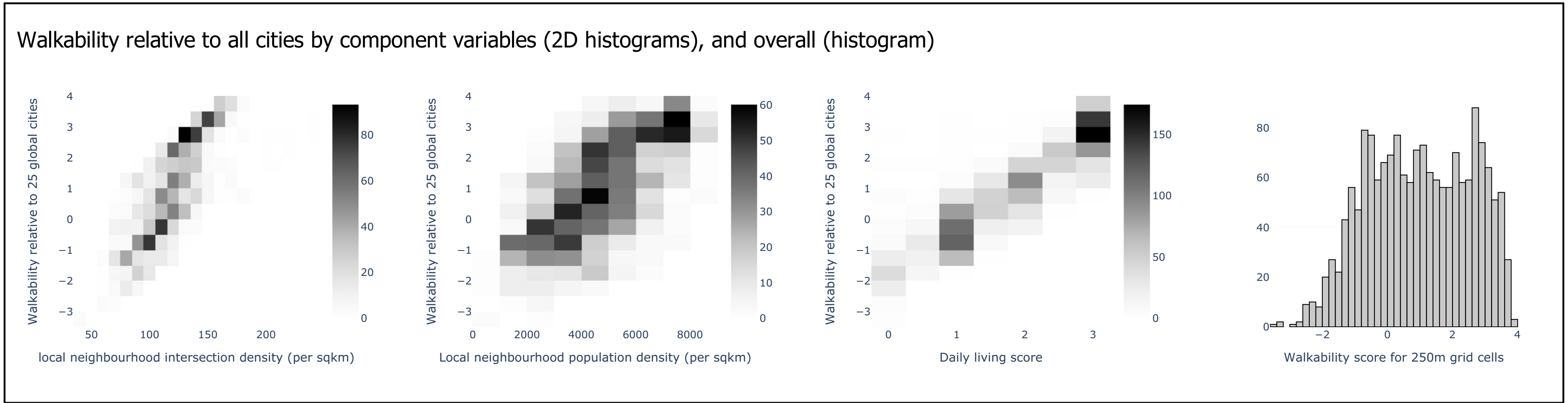

Mean 1000 m neighbourhood population per km²

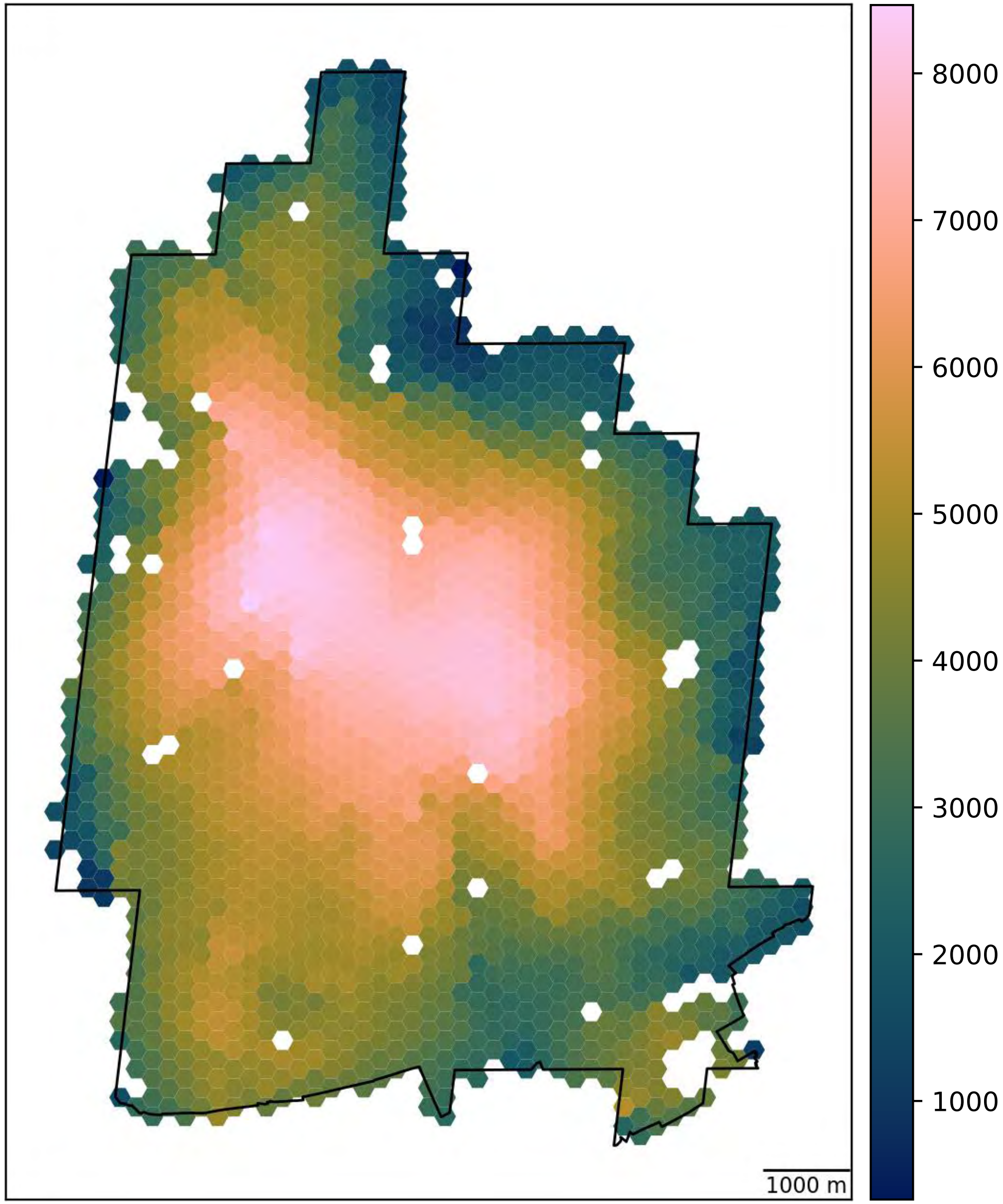

A: Estimated Mean 1000 m neighbourhood population per km² requirement for ≥80% probability of engaging in walking for transport

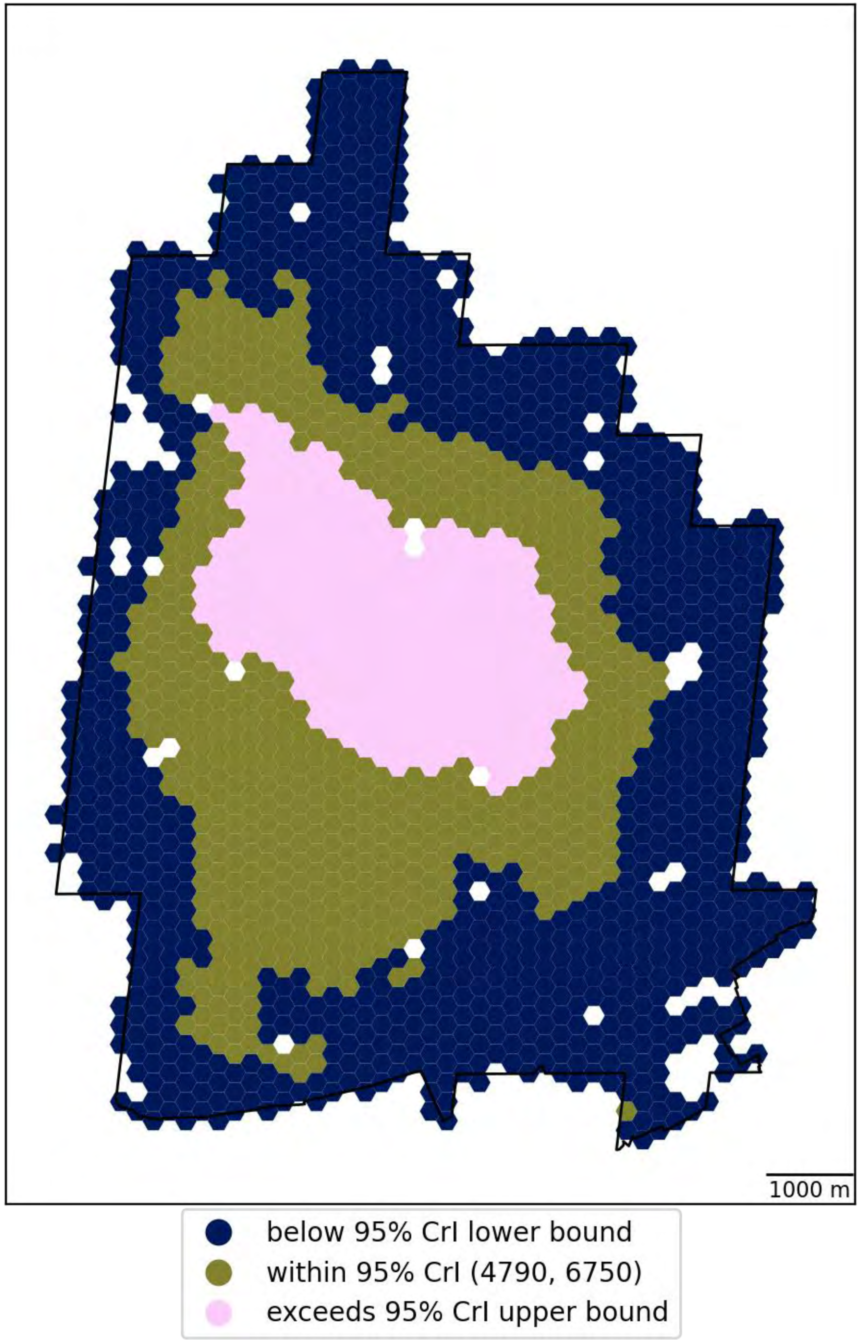

B: Estimated Mean 1000 m neighbourhood population per km² requirement for reaching the WHO's target of a ≥15% relative reduction in insufficient physical activity through walking

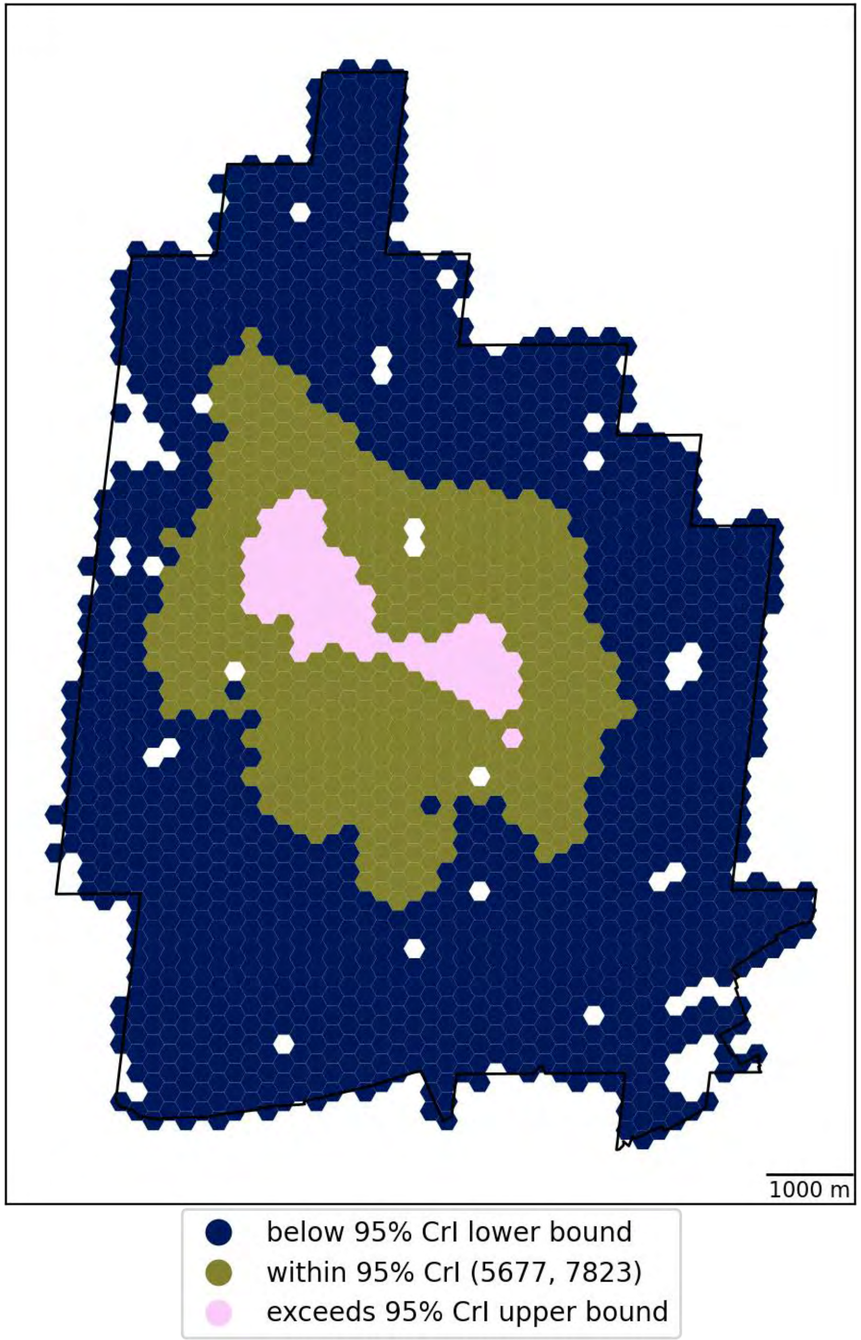

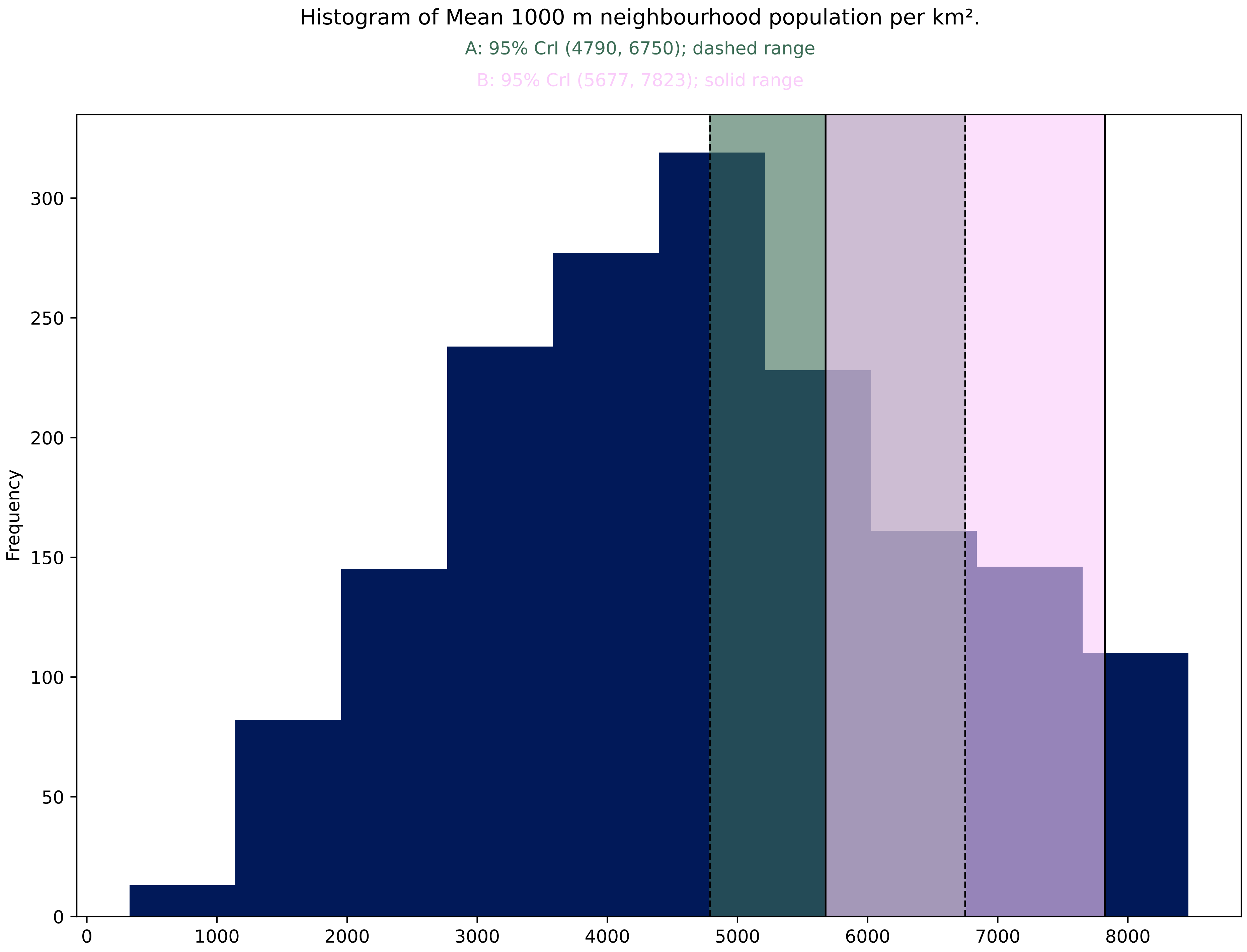

Histogram of Mean 1000 m neighbourhood population per km².
A: 95% CrI (4790, 6750); dashed range
B: 95% CrI (5677, 7823); solid range
Frequency
0
50
100
150
200
250
300
0
1000
2000
3000
4000
5000
6000
7000
8000

Mean 1000 m neighbourhood street intersections per km²

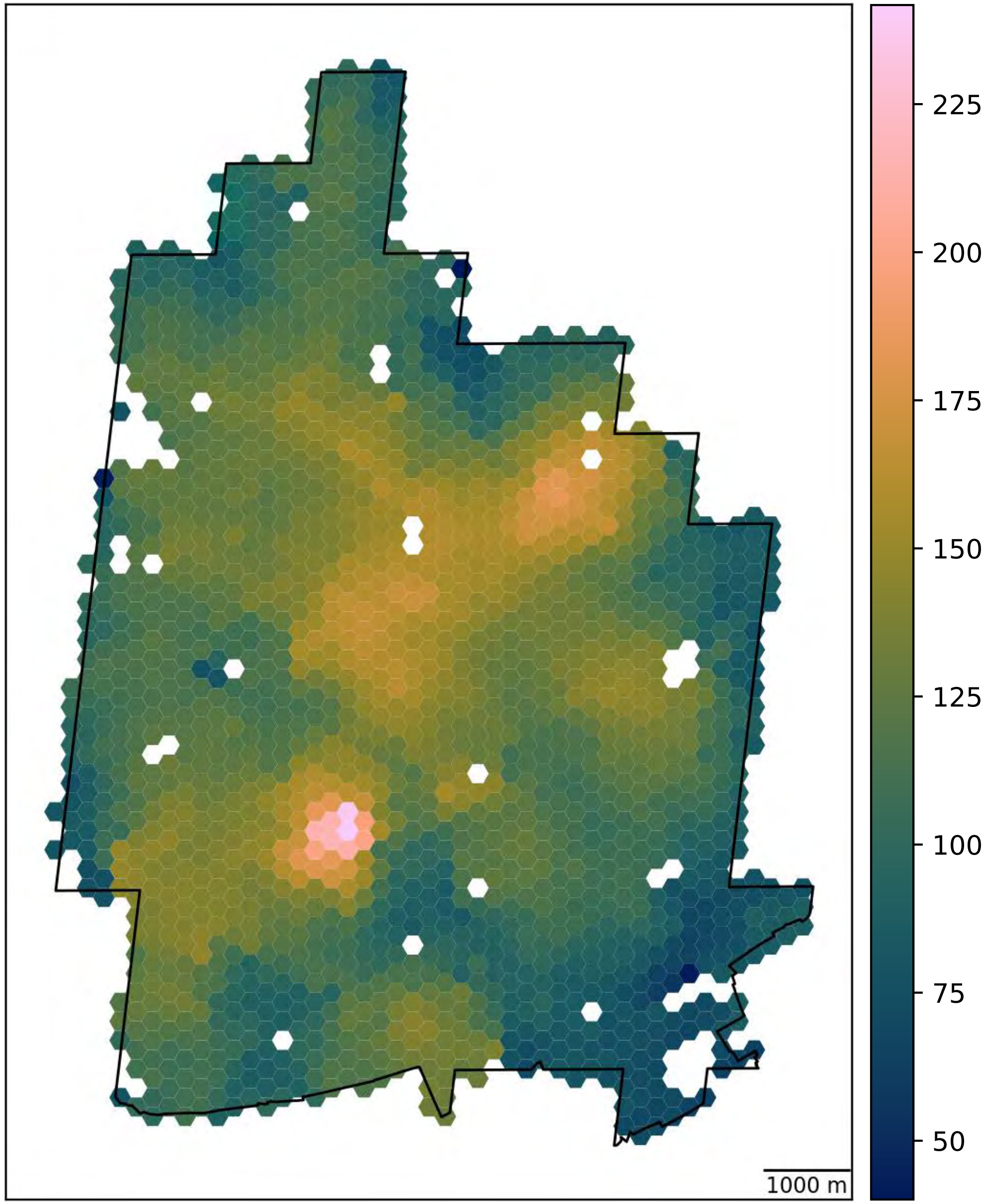

A: Estimated Mean 1000 m neighbourhood street intersections per km² requirement for ≥80% probability of engaging in walking for transport

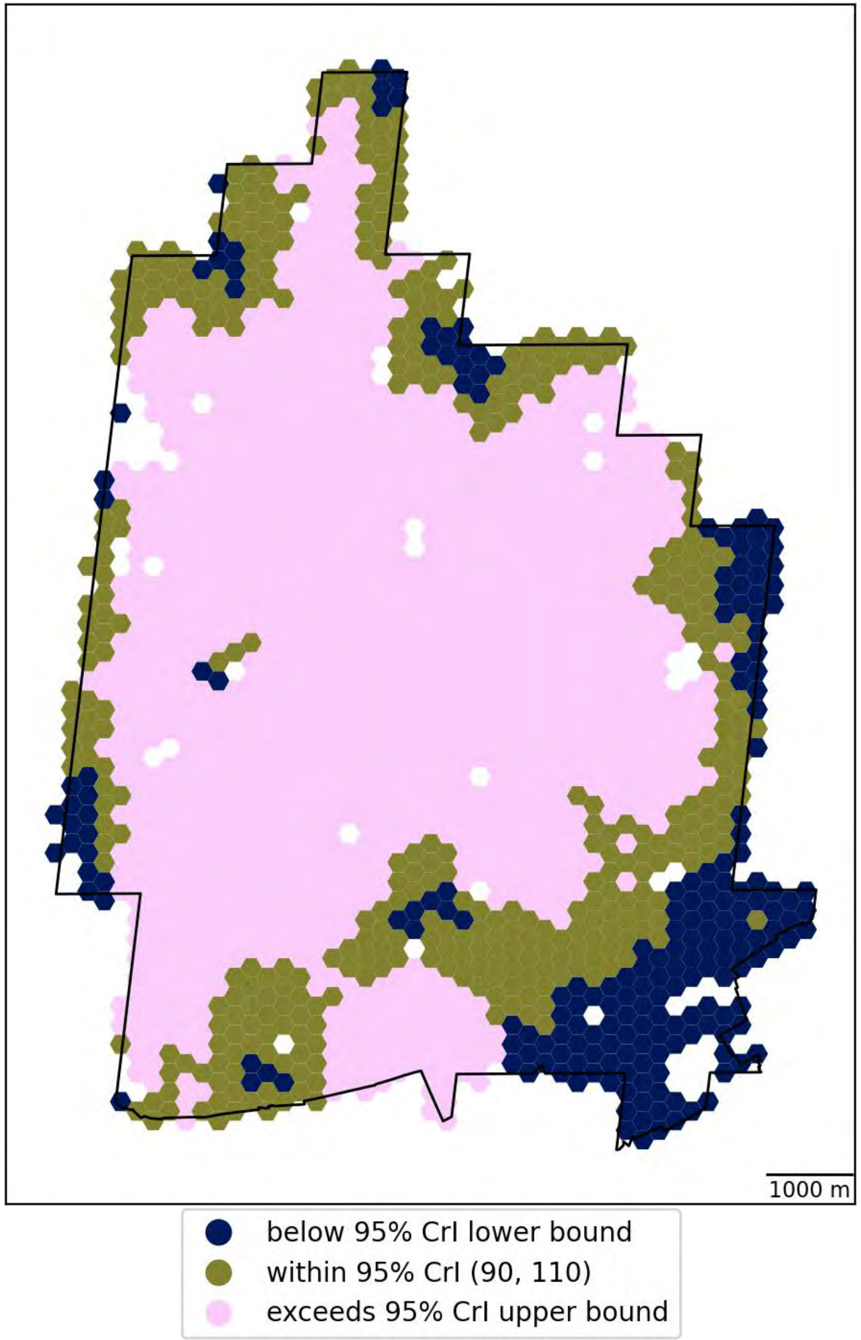

B: Estimated Mean 1000 m neighbourhood street intersections per km² requirement for reaching the WHO's target of a ≥15% relative reduction in insufficient physical activity through walking

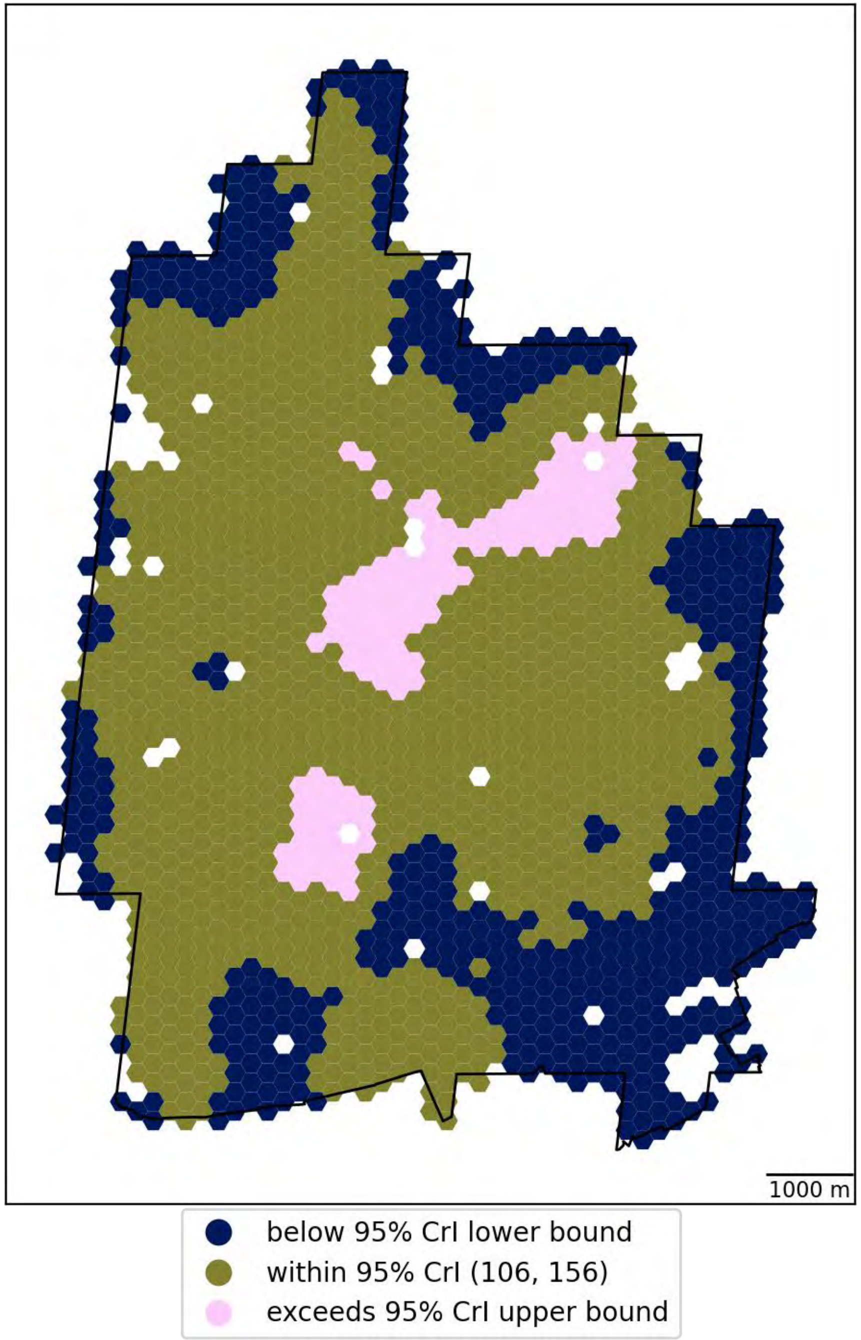

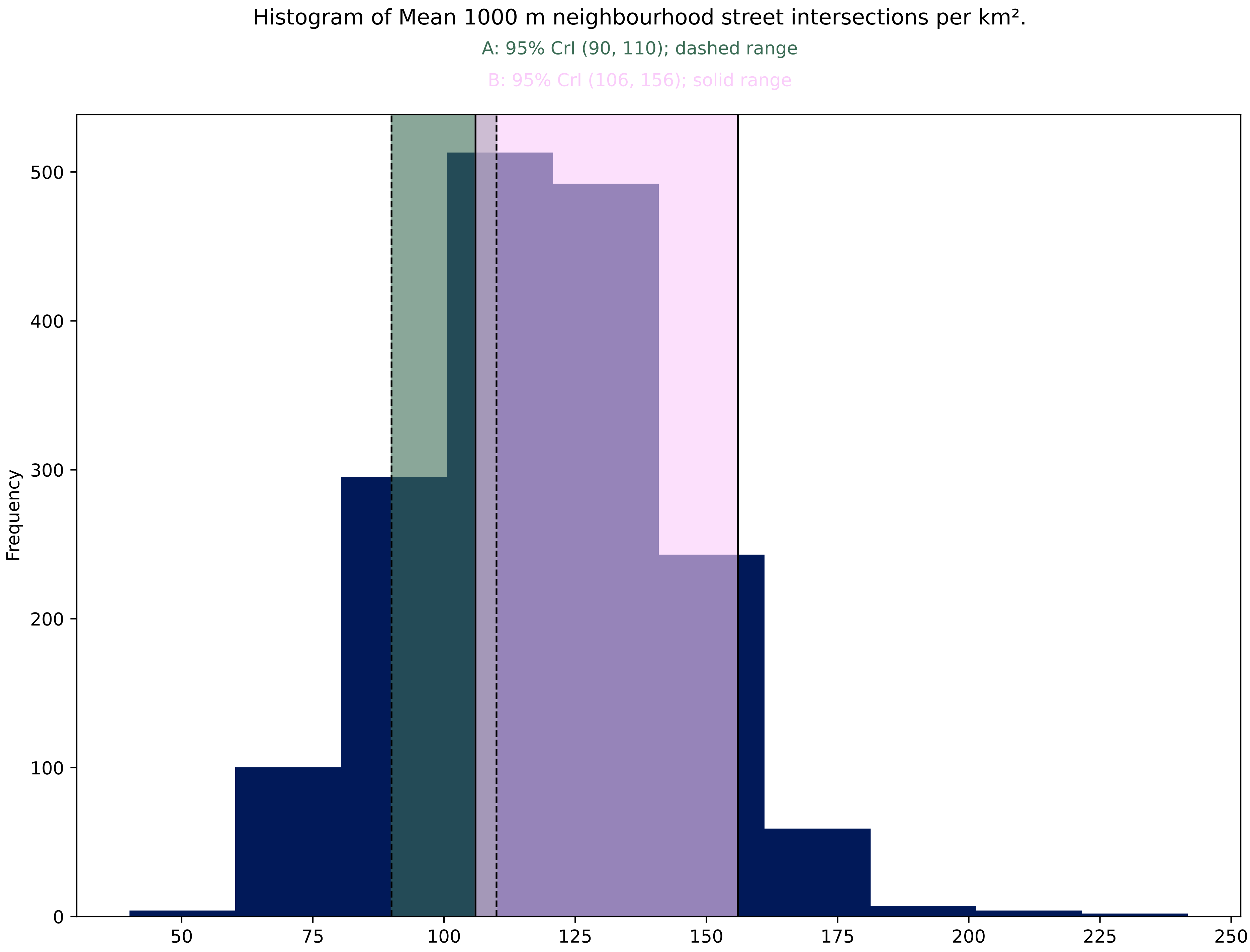
Histogram of Mean 1000 m neighbourhood street intersections per km².
A: 95% CrI (90, 110); dashed range
B: 95% CrI (106, 156); solid range
Frequency
0
100
200
300
400
500
50
75
100
125
150
175
200
225
250

Distance to nearest public transport stops (m; up to 500m)

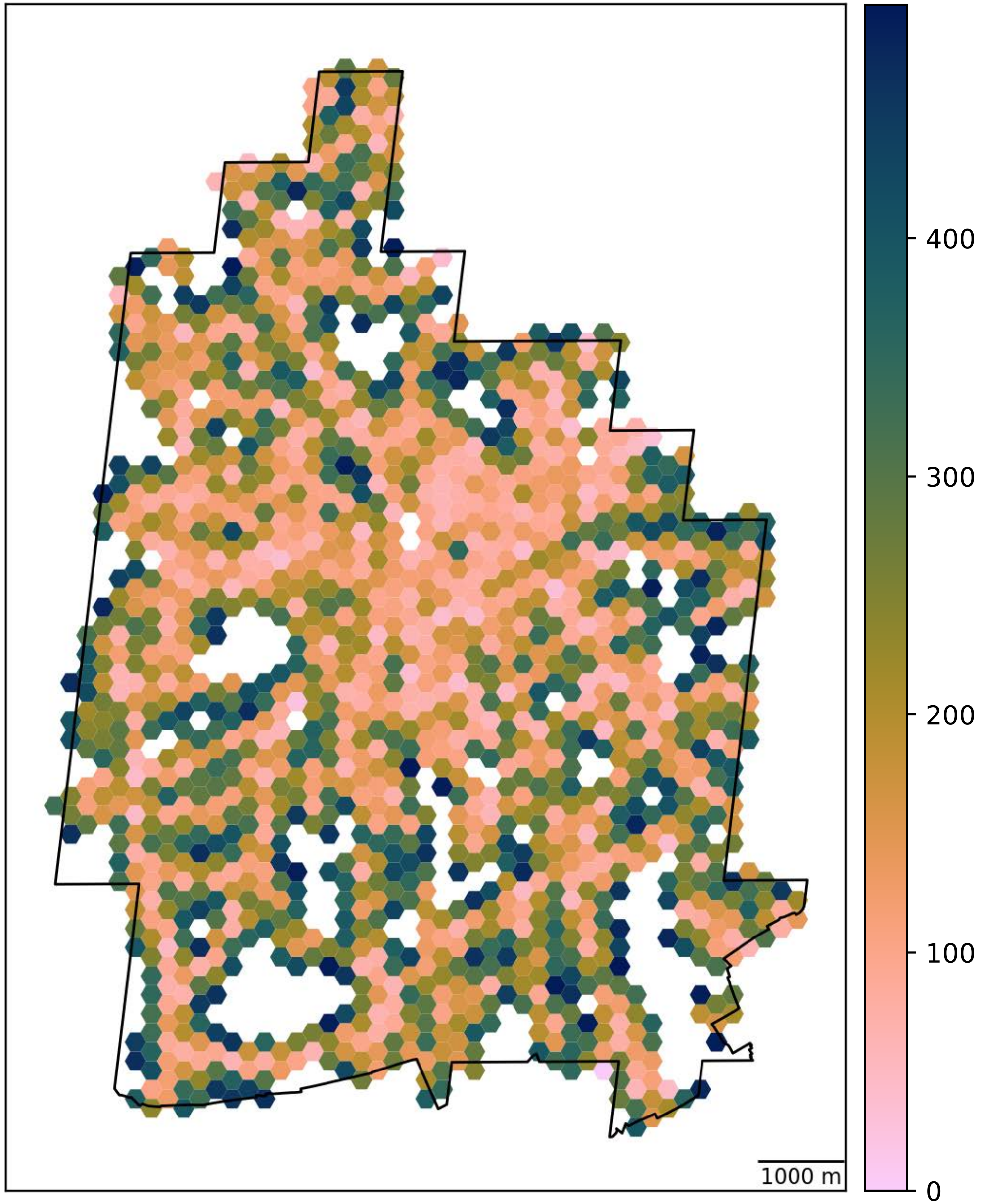

distances: Estimated Distance to nearest public transport stops (m; up to 500m) requirement for distances to destinations, measured up to a maximum distance target threshold of 500 metres

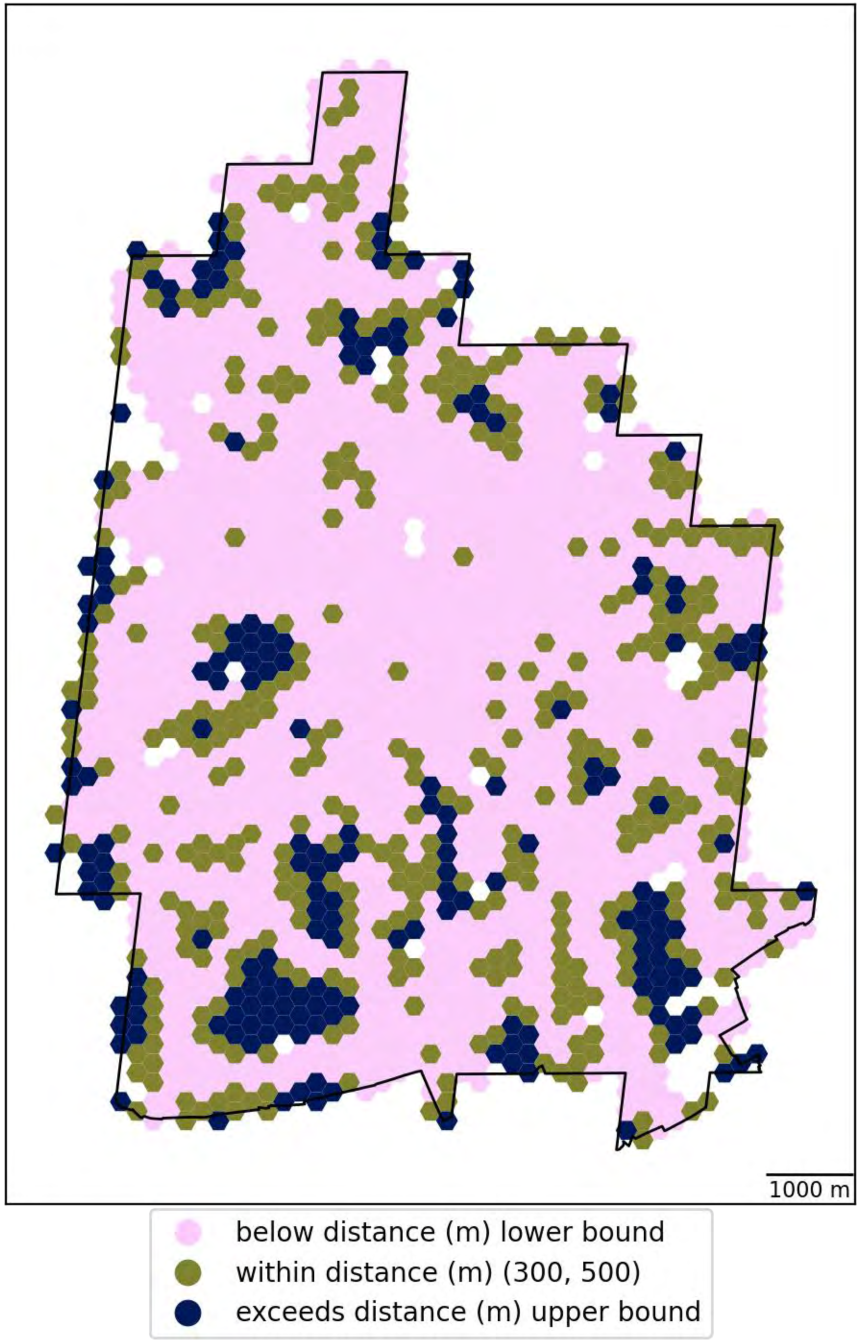

Histogram of Distance to nearest public transport stops (m; up to 500m).

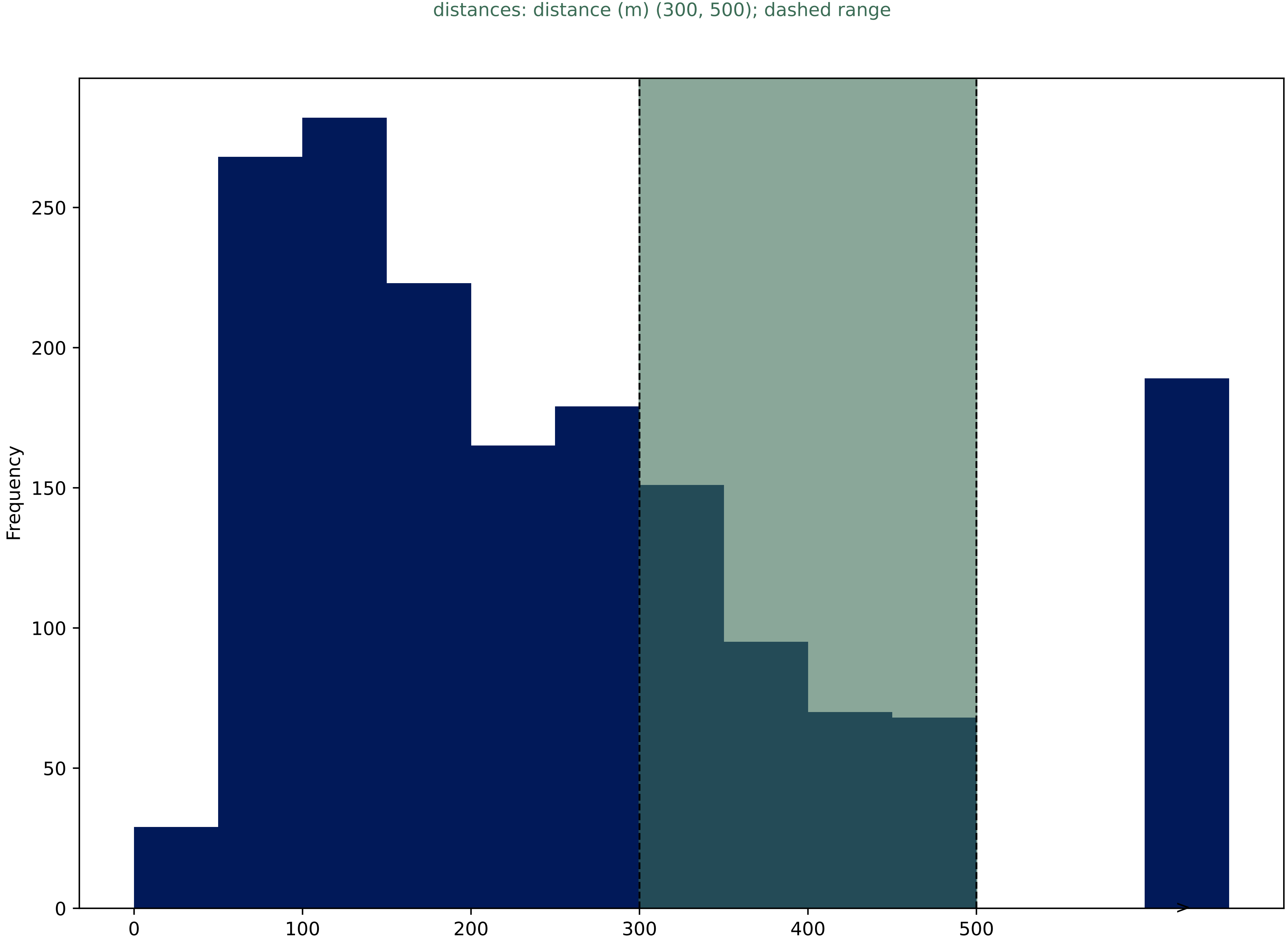

Distance to nearest park (m; up to 500m)

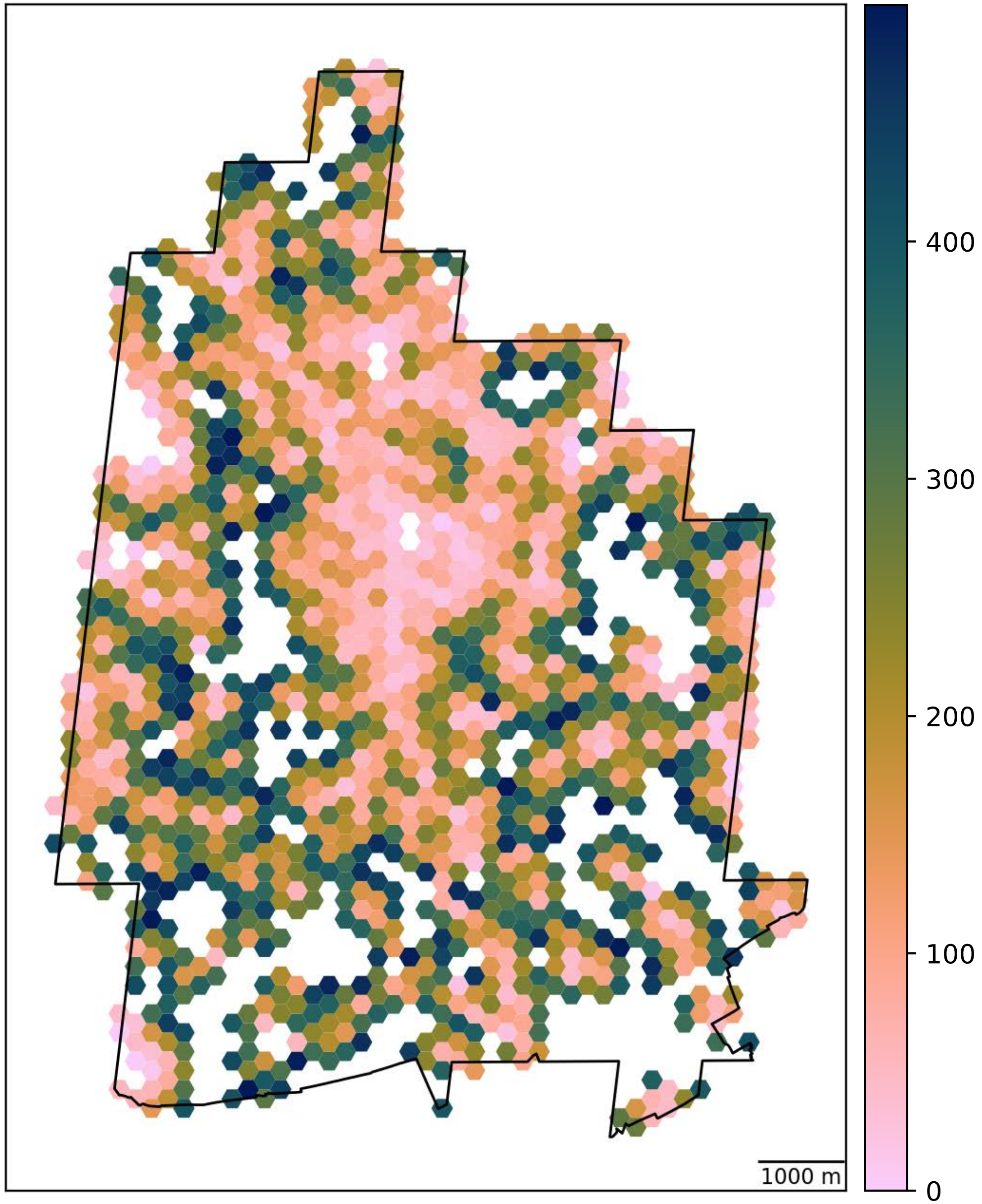

distances: Estimated Distance to nearest park (m; up to 500m) requirement for distances to destinations, measured up to a maximum distance target threshold of 500 metres

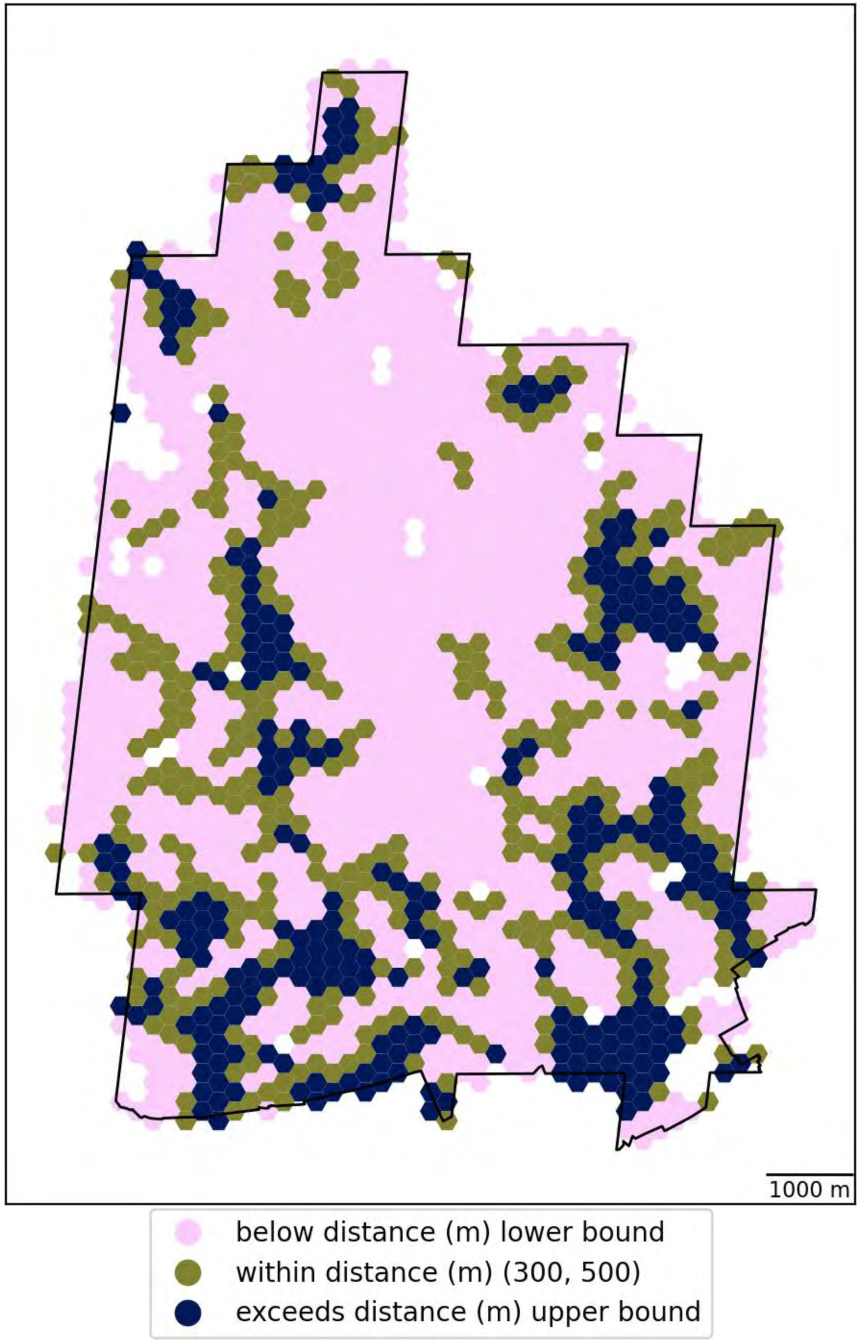

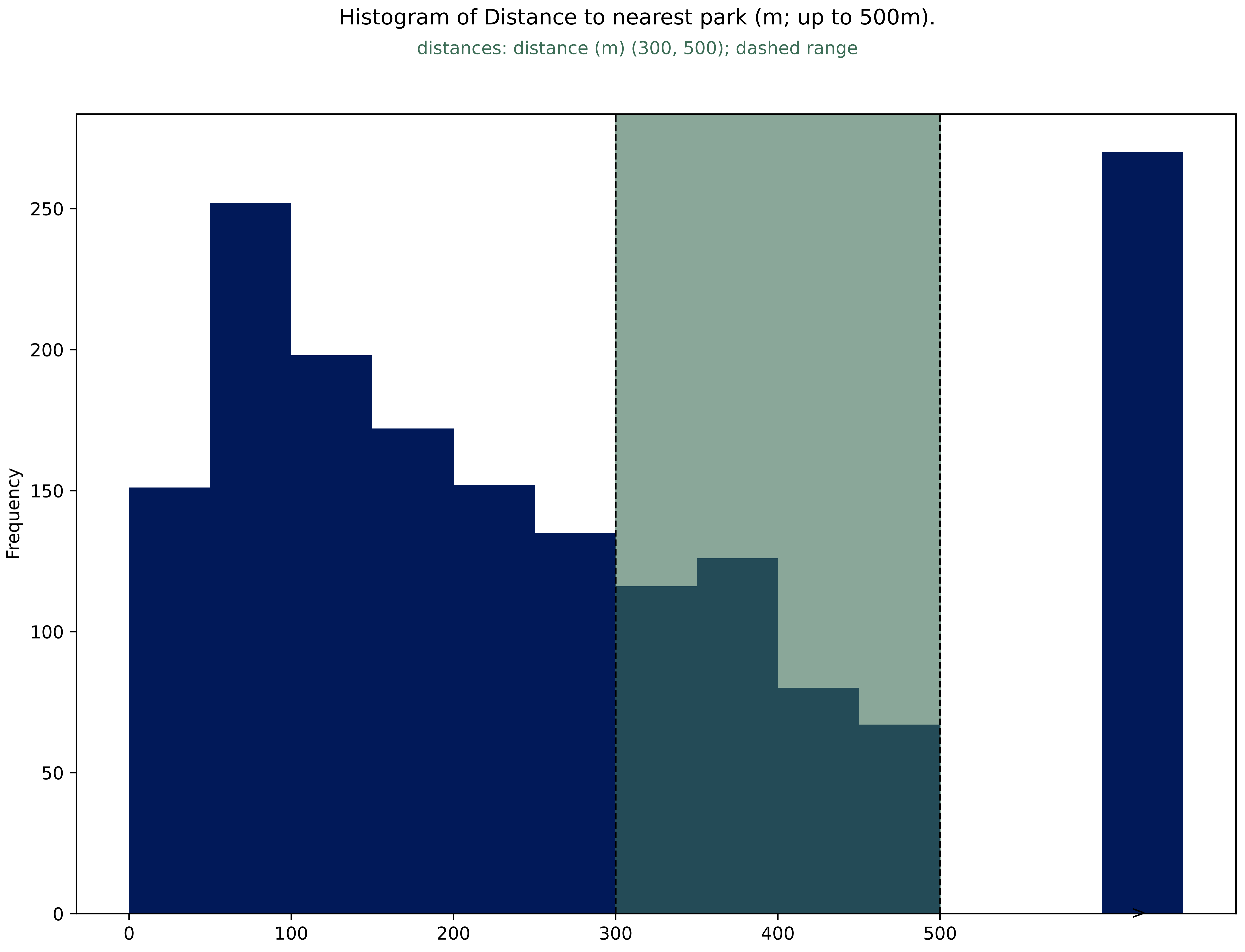
Histogram of Distance to nearest park (m; up to 500m).
distances: distance (m) (300, 500); dashed range
Frequency
0
50
100
150
200
250
0
100
200
300
400
500
>

Europe, Belgium, Ghent

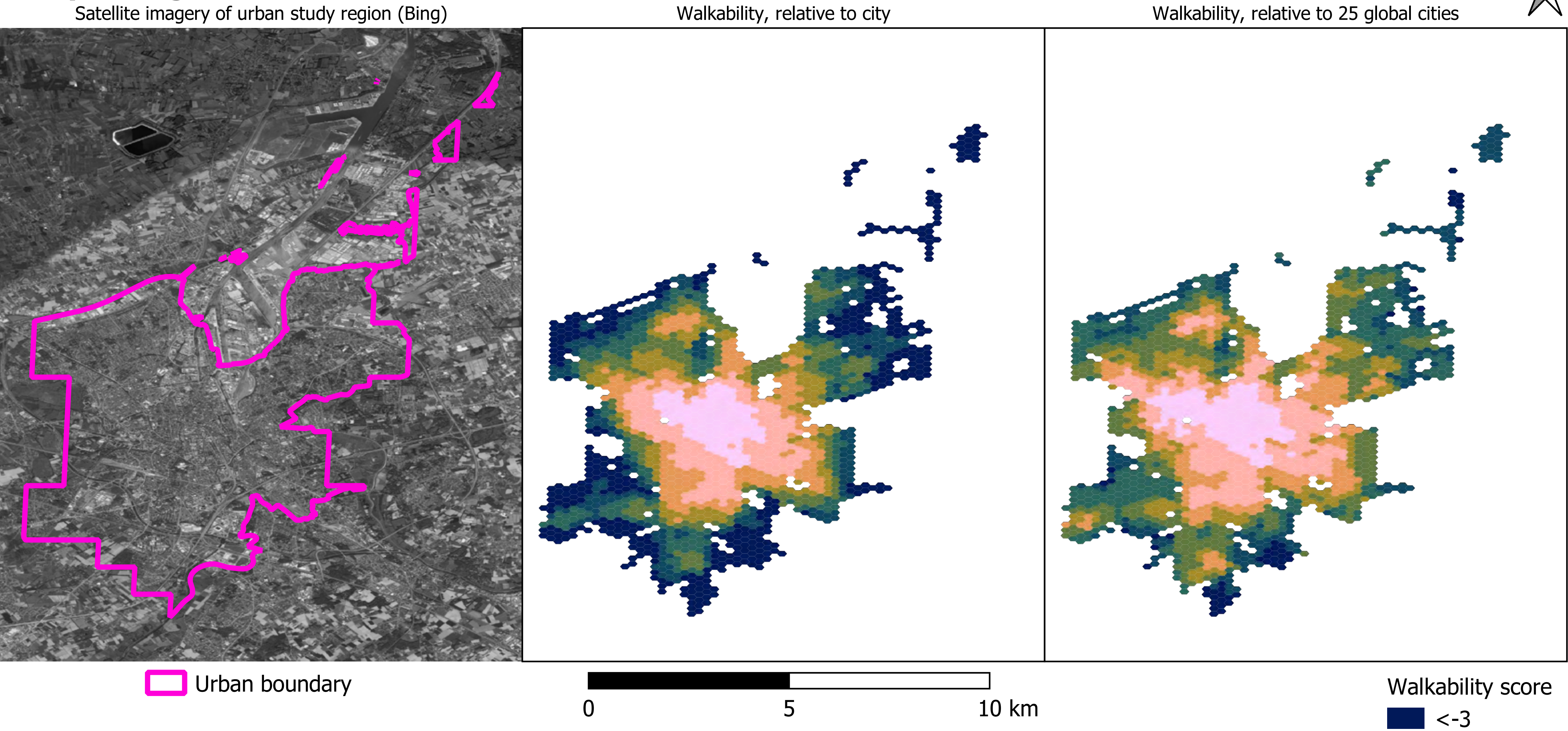

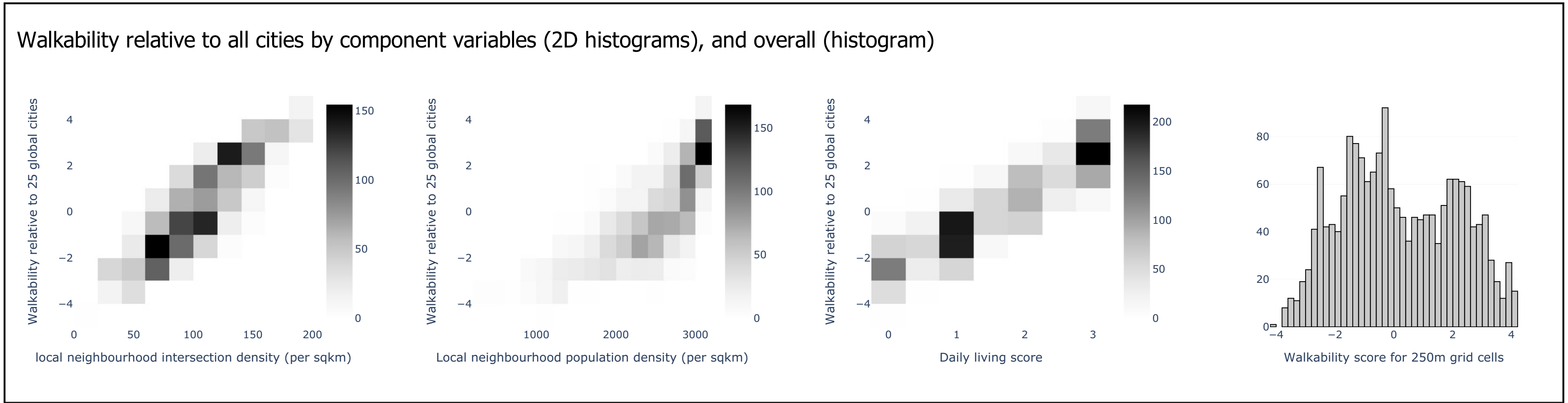

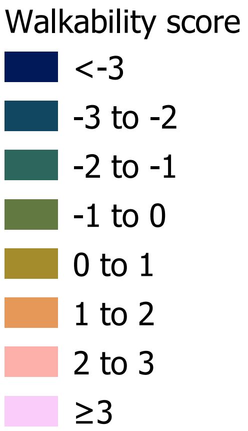

Mean 1000 m neighbourhood population per km²

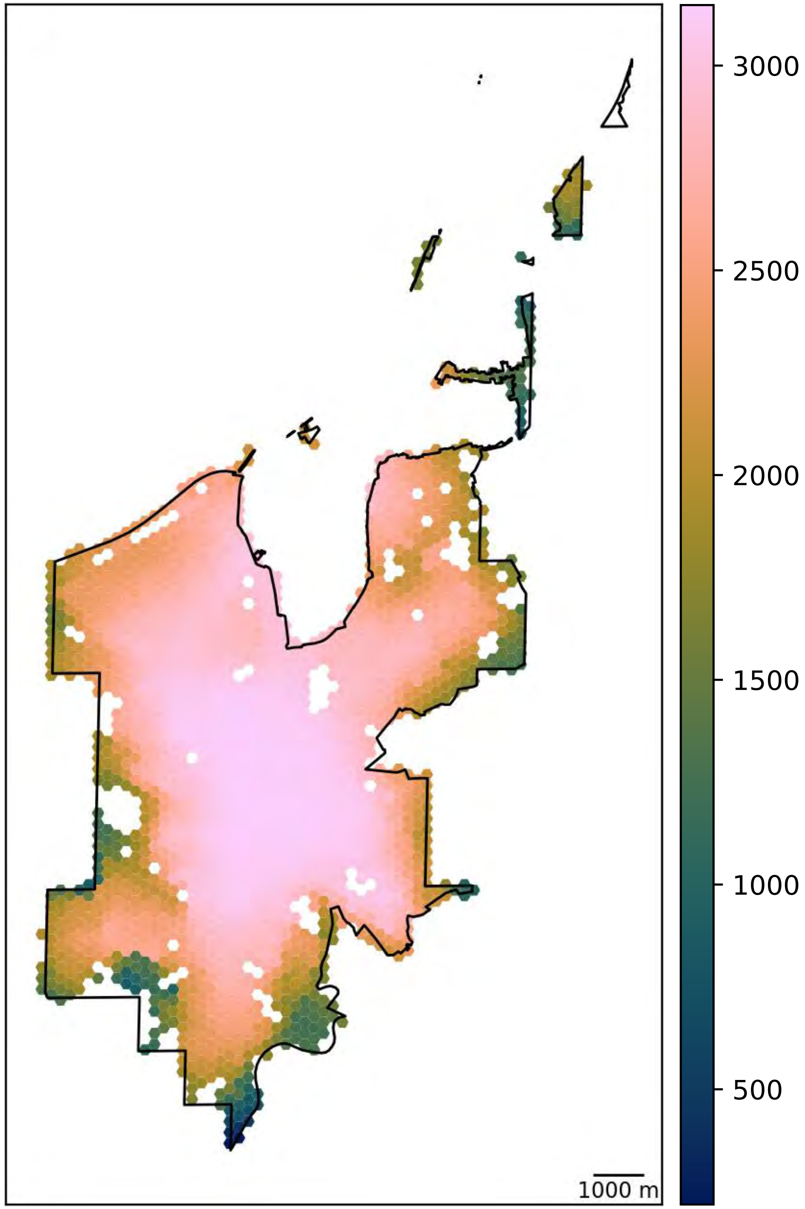

A: Estimated Mean 1000 m neighbourhood population per km² requirement for ≥80% probability of engaging in walking for transport

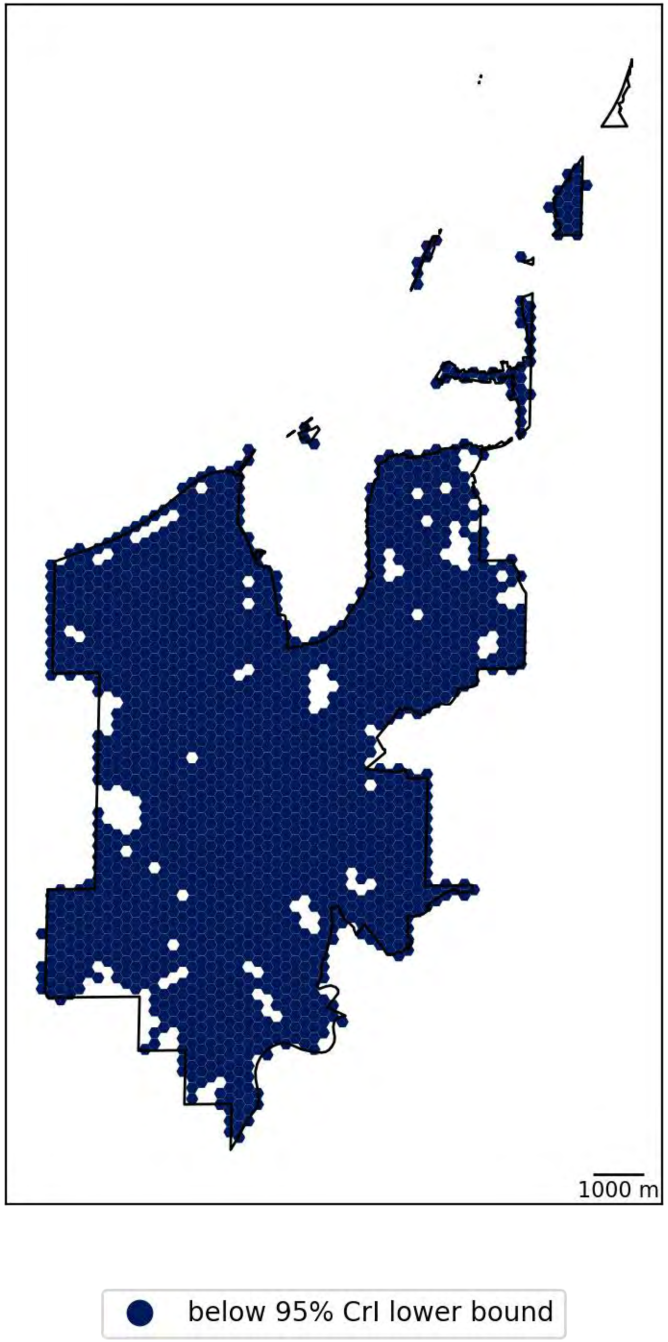

B: Estimated Mean 1000 m neighbourhood population per km² requirement for reaching the WHO's target of a ≥15% relative reduction in insufficient physical activity through walking

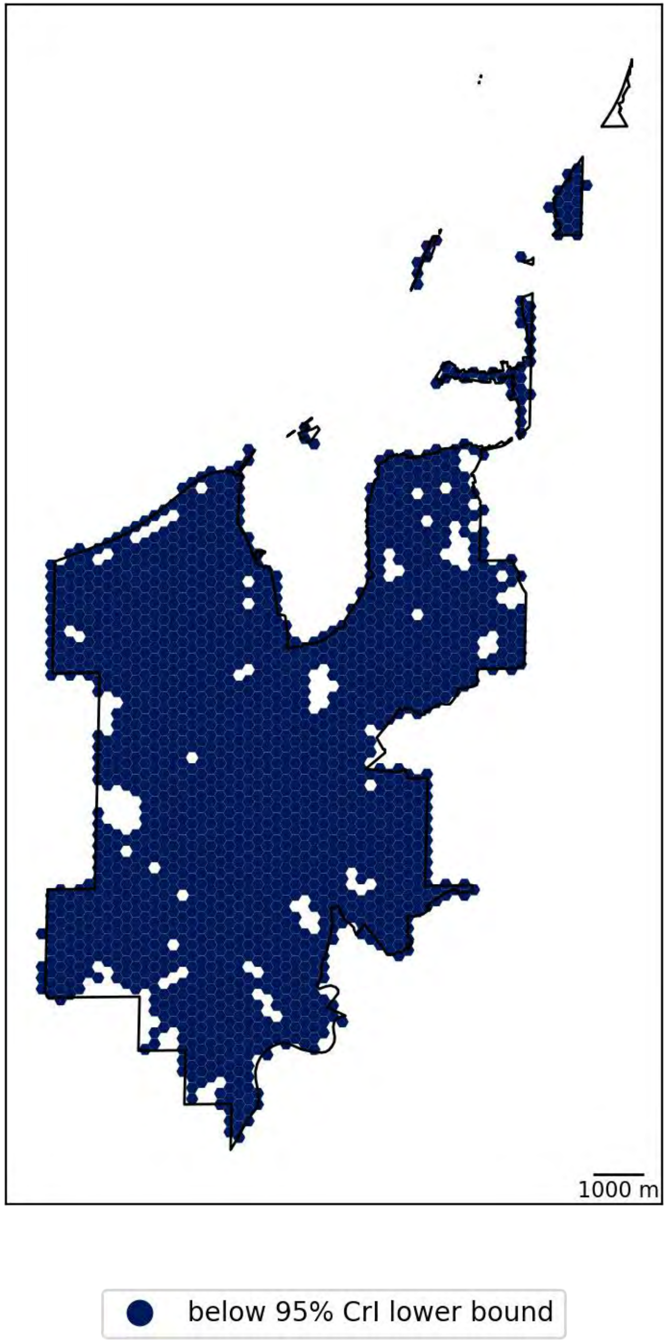

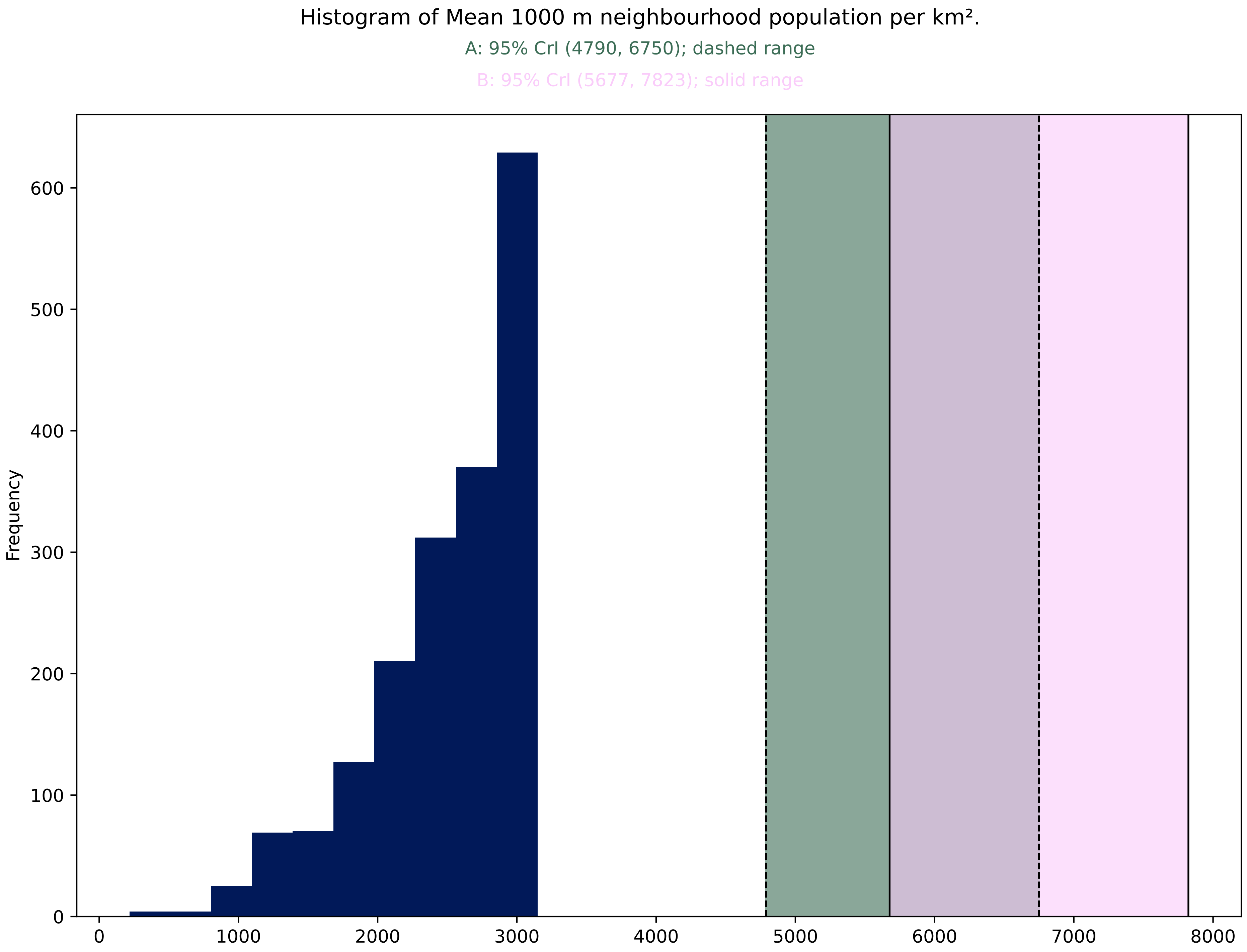
Histogram of Mean 1000 m neighbourhood population per km².
A: 95% CrI (4790, 6750); dashed range
B: 95% CrI (5677, 7823); solid range
Frequency
0
100
200
300
400
500
600
0
1000
2000
3000
4000
5000
6000
7000
8000

Mean 1000 m neighbourhood street intersections per km²

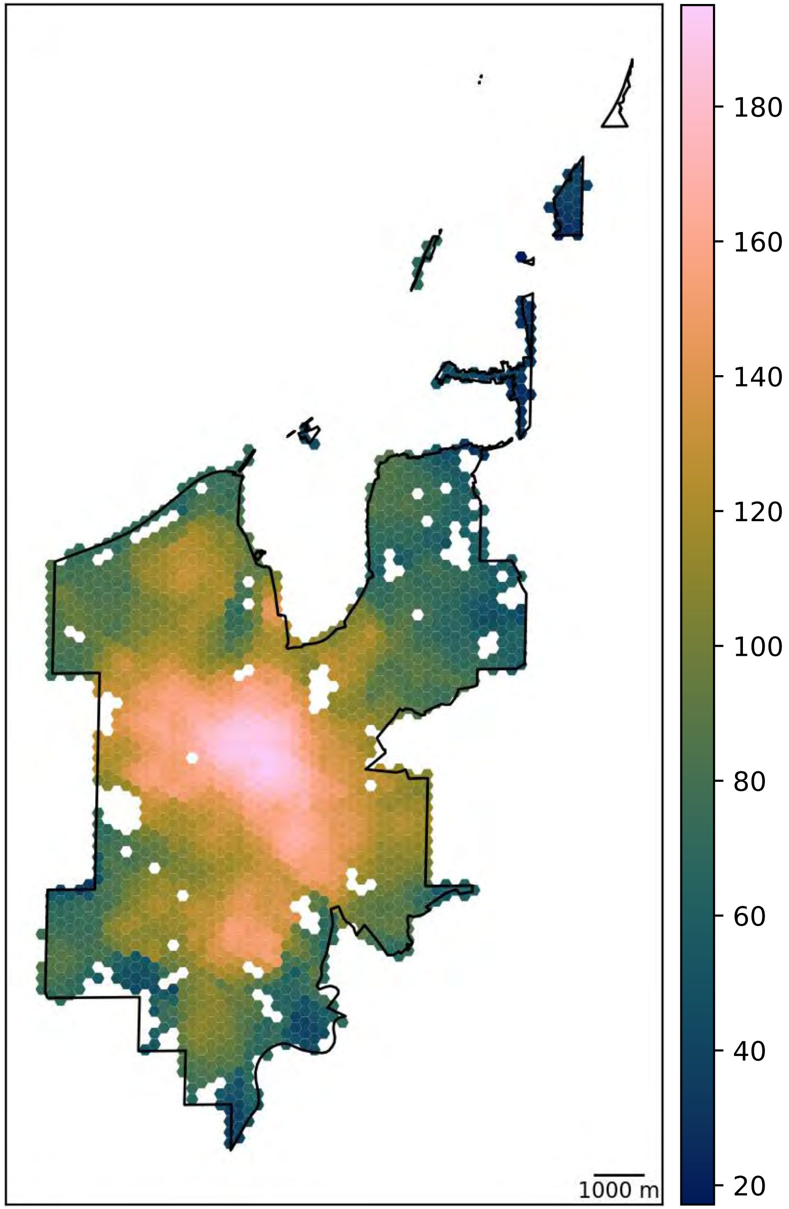

A: Estimated Mean 1000 m neighbourhood street intersections per km² requirement for ≥80% probability of engaging in walking for transport

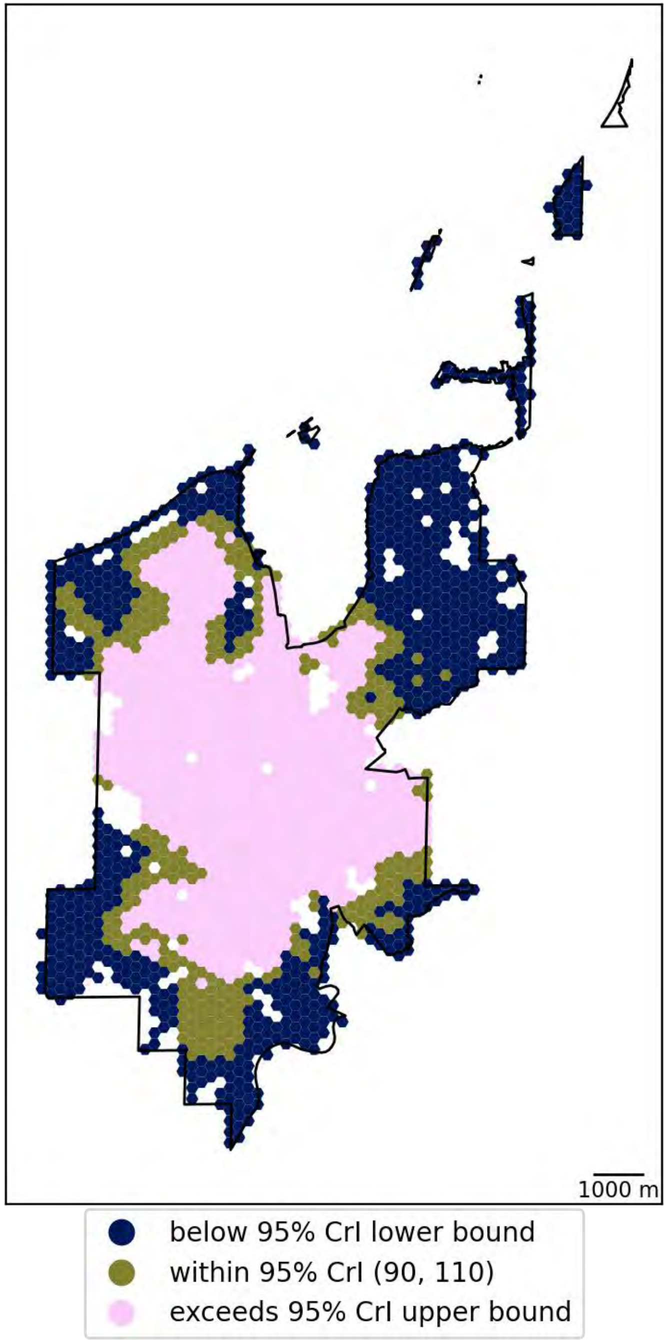

B: Estimated Mean 1000 m neighbourhood street intersections per km² requirement for reaching the WHO's target of a ≥15% relative reduction in insufficient physical activity through walking

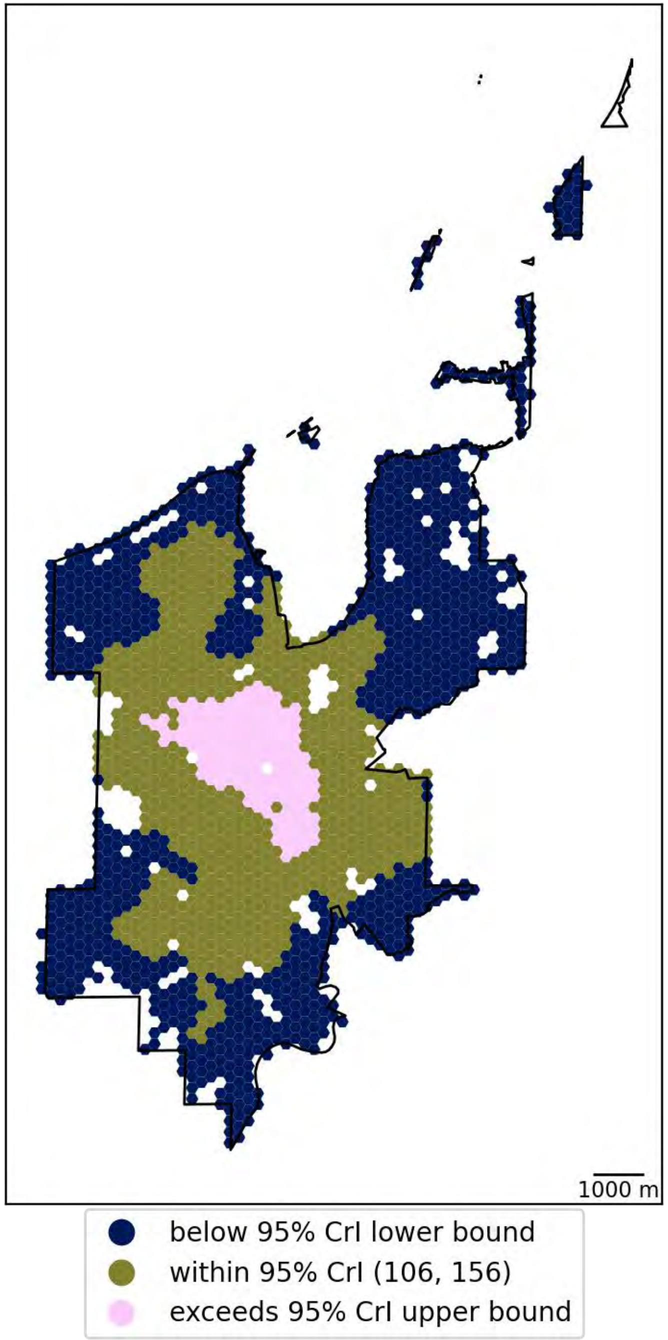

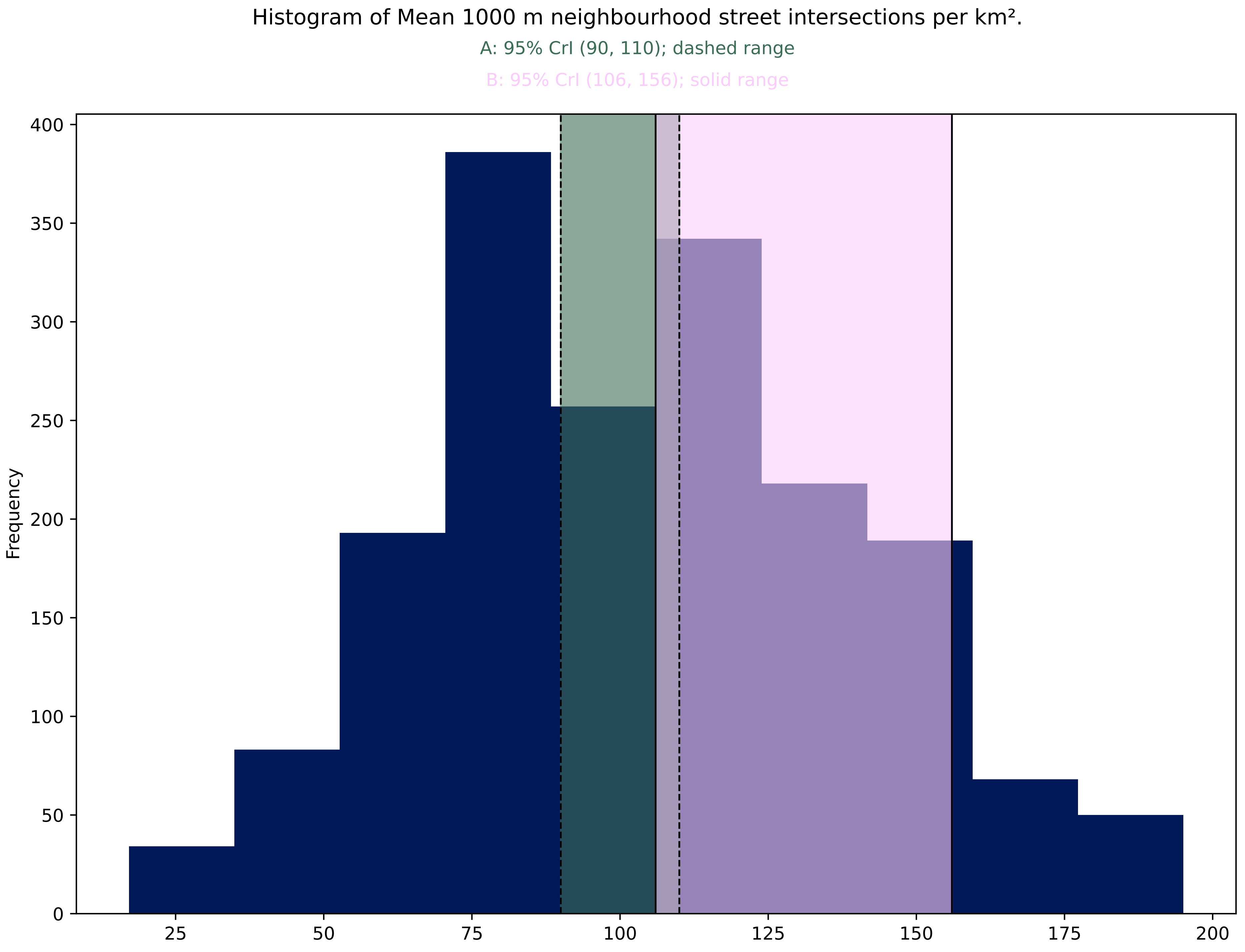
Histogram of Mean 1000 m neighbourhood street intersections per km².
A: 95% CrI (90, 110); dashed range
B: 95% CrI (106, 156); solid range
Frequency
0
50
100
150
200
250
300
350
400
25
50
75
100
125
150
175
200

Distance to nearest public transport stops (m; up to 500m)

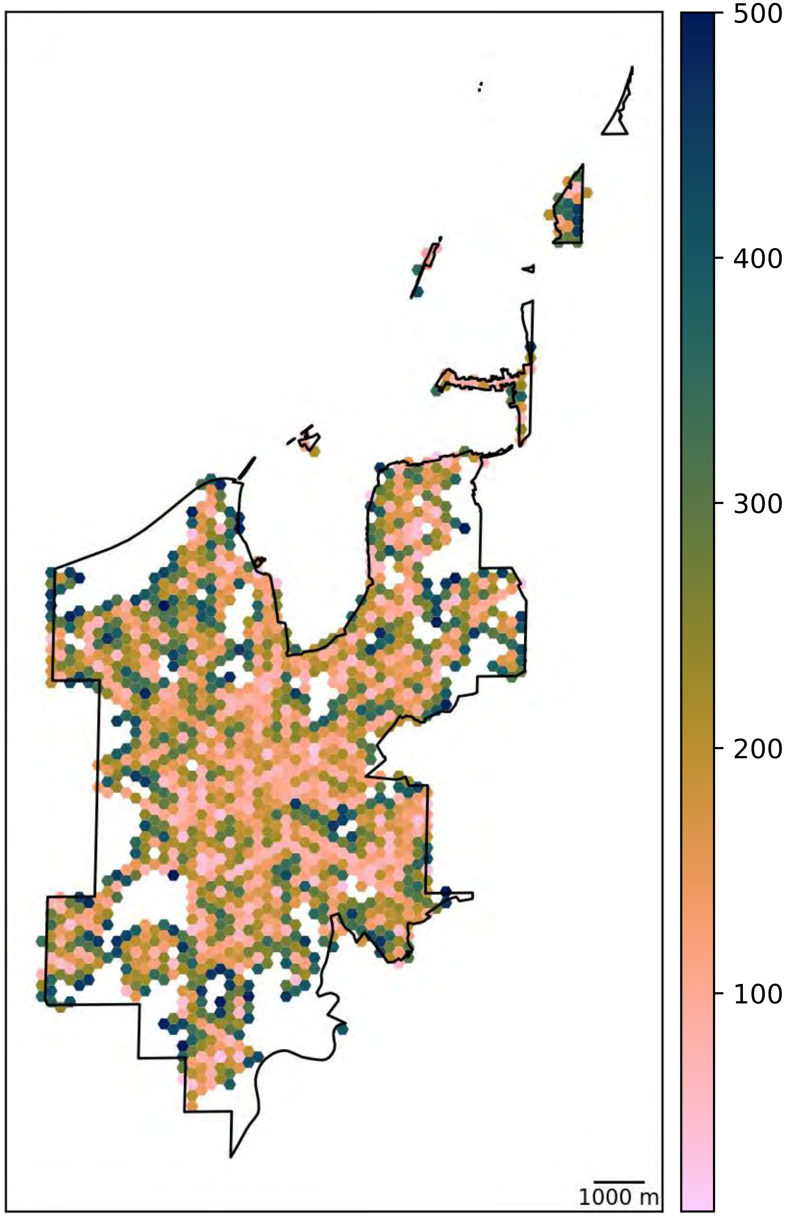

distances: Estimated Distance to nearest public transport stops (m; up to 500m) requirement for distances to destinations, measured up to a maximum distance target threshold of 500 metres

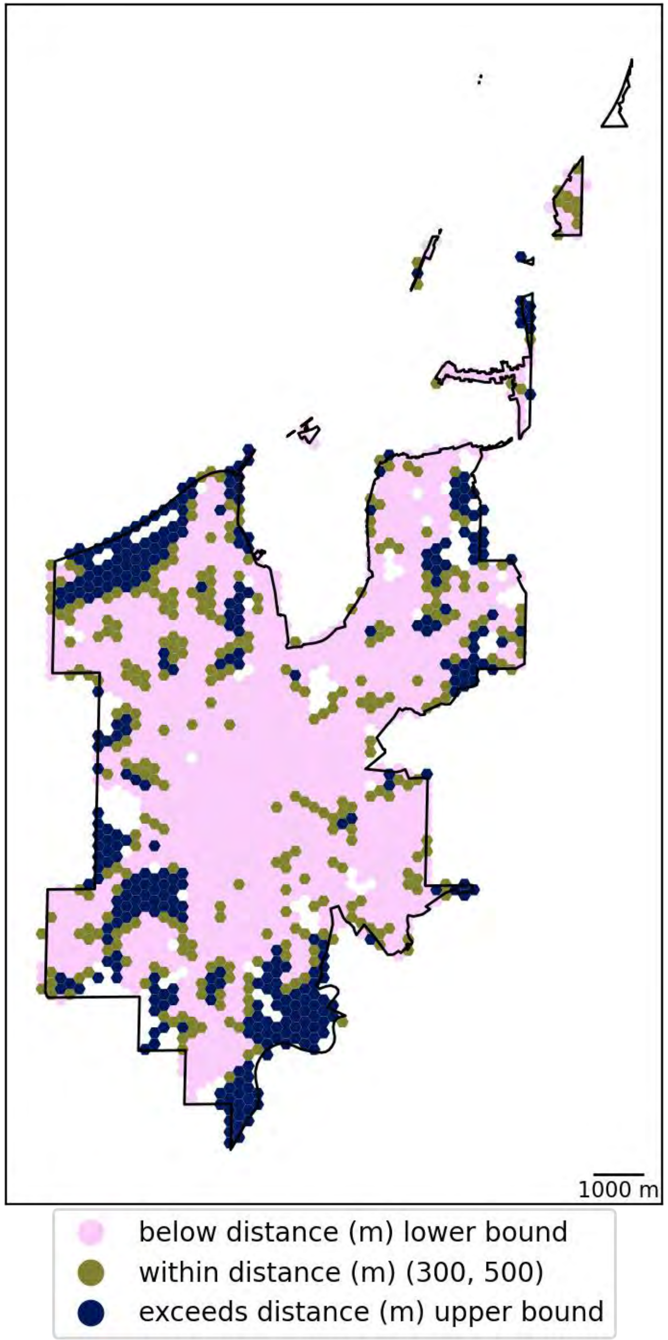

Histogram of Distance to nearest public transport stops (m; up to 500m).

distances: distance (m) (300, 500); dashed range

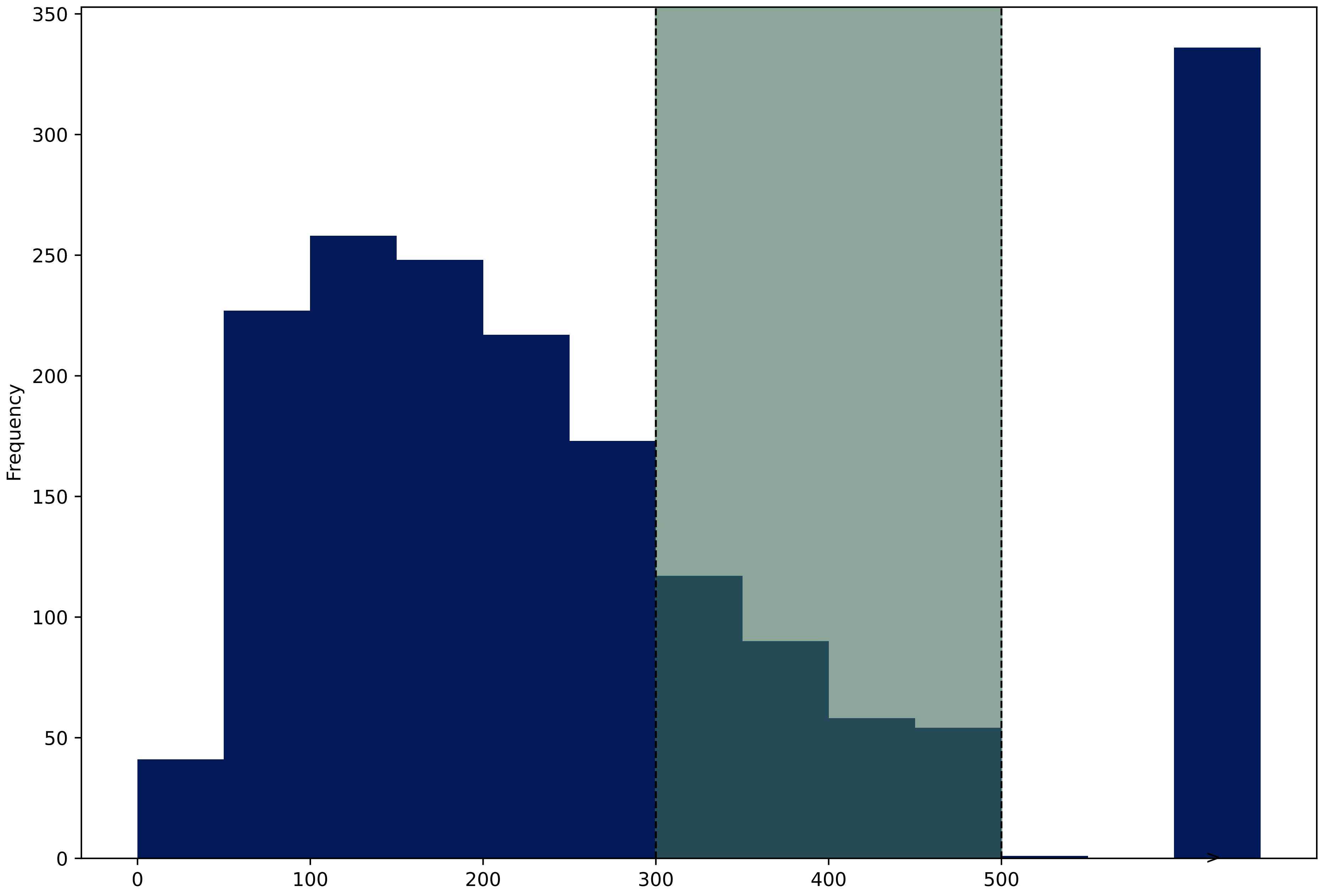

Distance to nearest park (m; up to 500m)

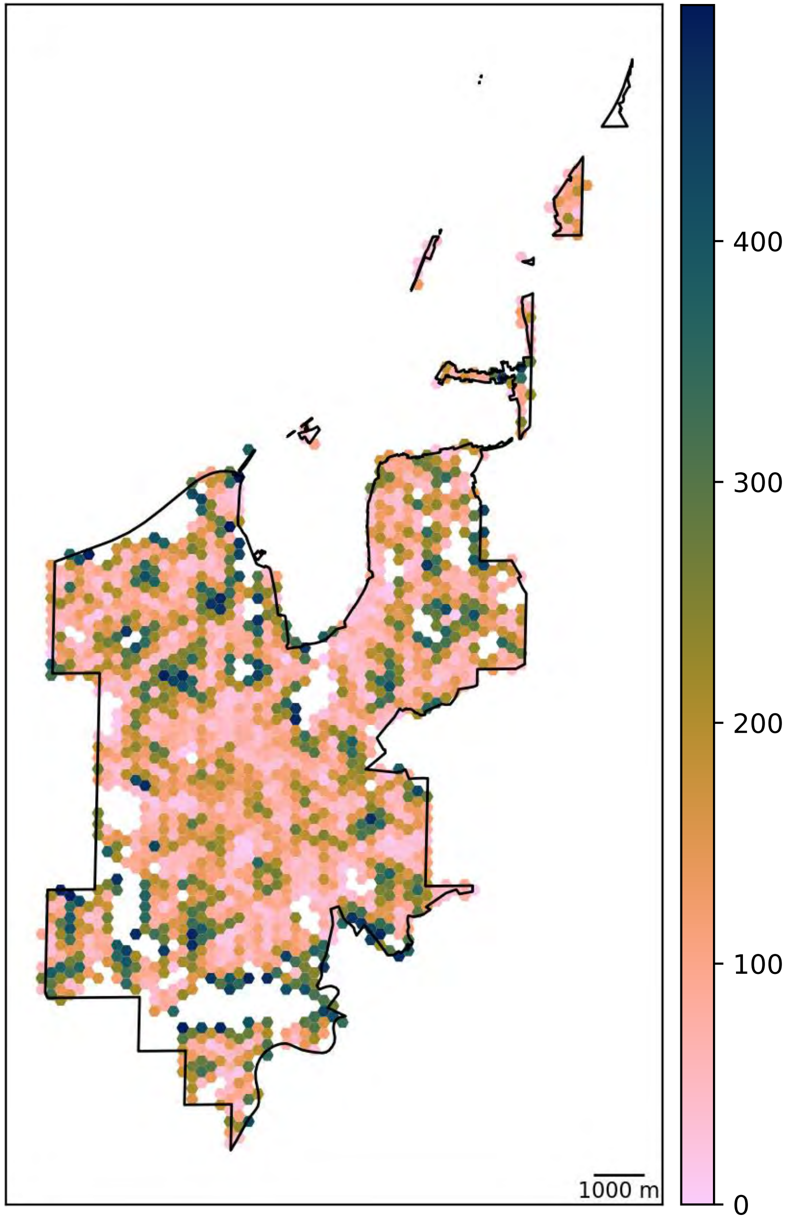

distances: Estimated Distance to nearest park (m; up to 500m) requirement for distances to destinations, measured up to a maximum distance target threshold of 500 metres

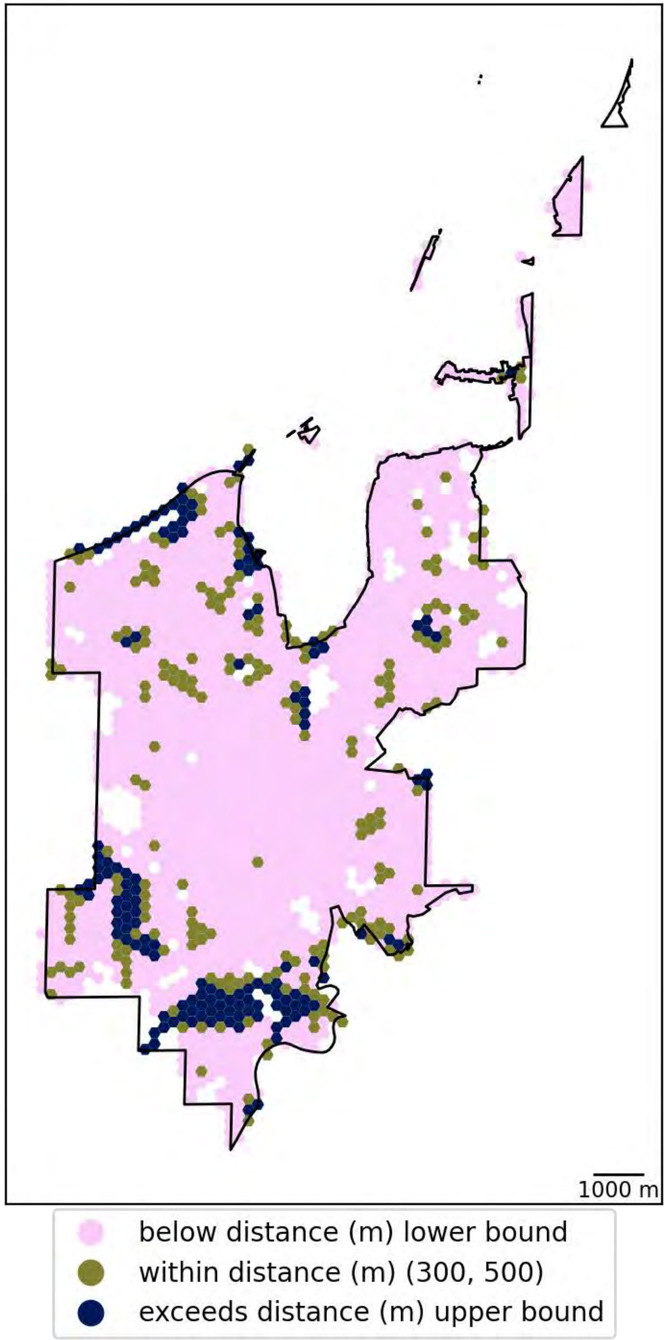

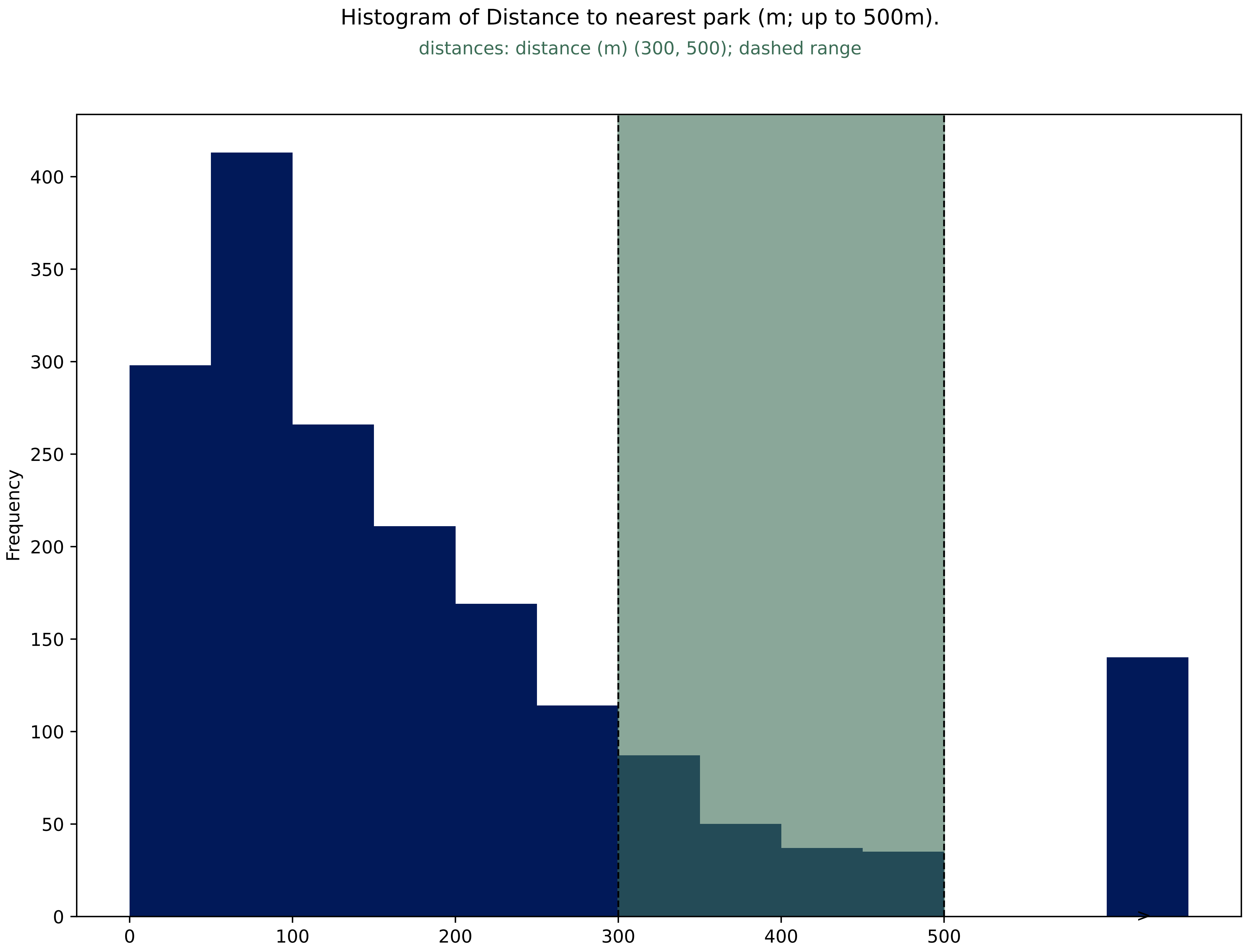

Histogram of Distance to nearest park (m; up to 500m).
distances: distance (m) (300, 500); dashed range
Frequency
0
50
100
150
200
250
300
350
400
0
100
200
300
400
500
>

Europe, Czech Republic , Olomouc

Satellite imagery of urban study region (Bing)

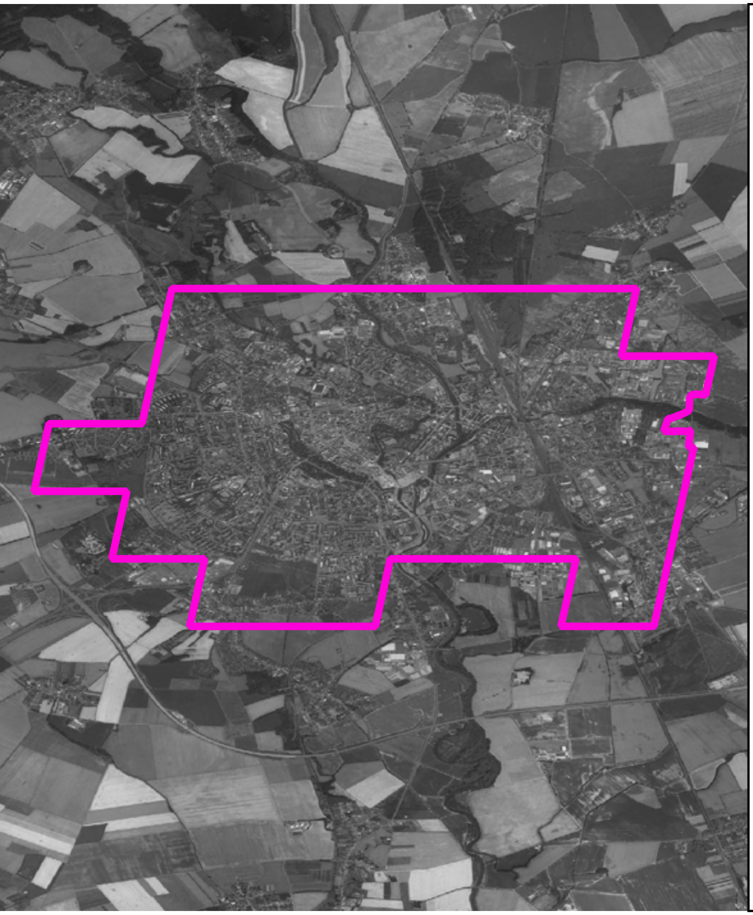

Walkability, relative to city

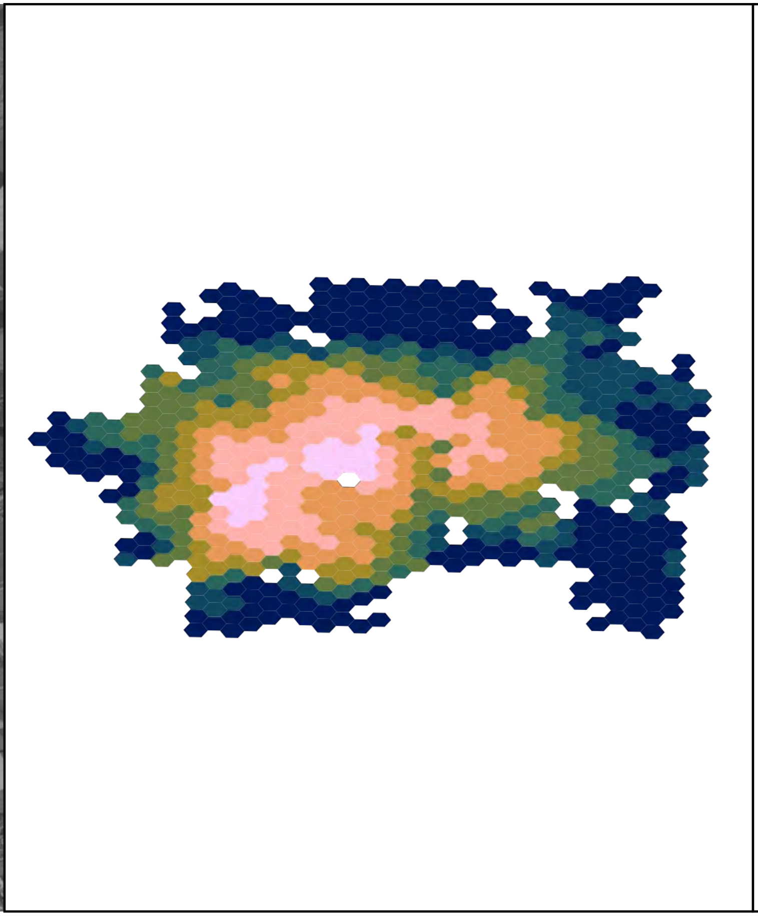

Walkability, relative to 25 global cities

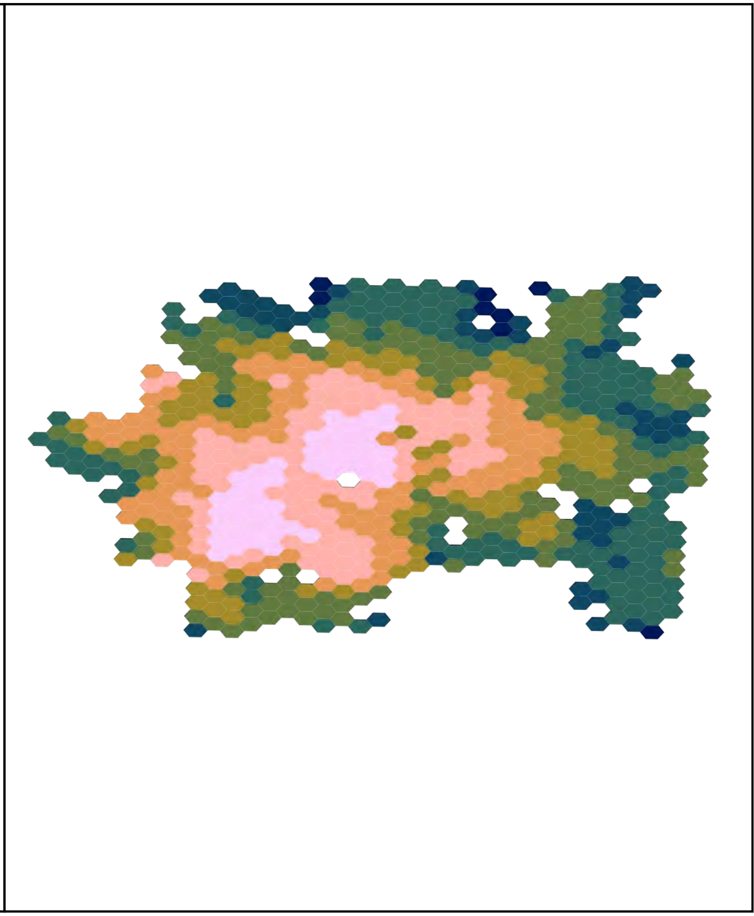

Urban boundary

0 3 6 km

Walkability score

- <-3
- -3 to -2
- -2 to -1
- -1 to 0
- 0 to 1
- 1 to 2
- 2 to 3
- ≥3

Walkability relative to all cities by component variables (2D histograms), and overall (histogram)

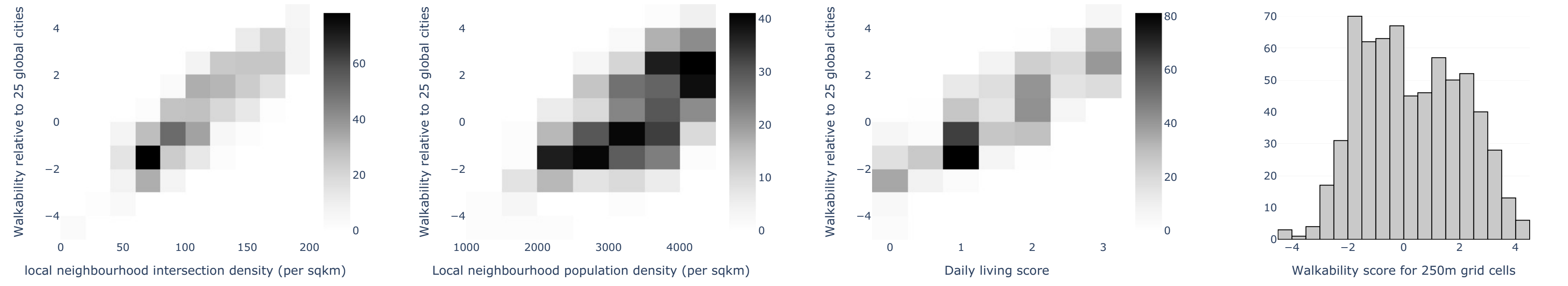

Mean 1000 m neighbourhood population per km²

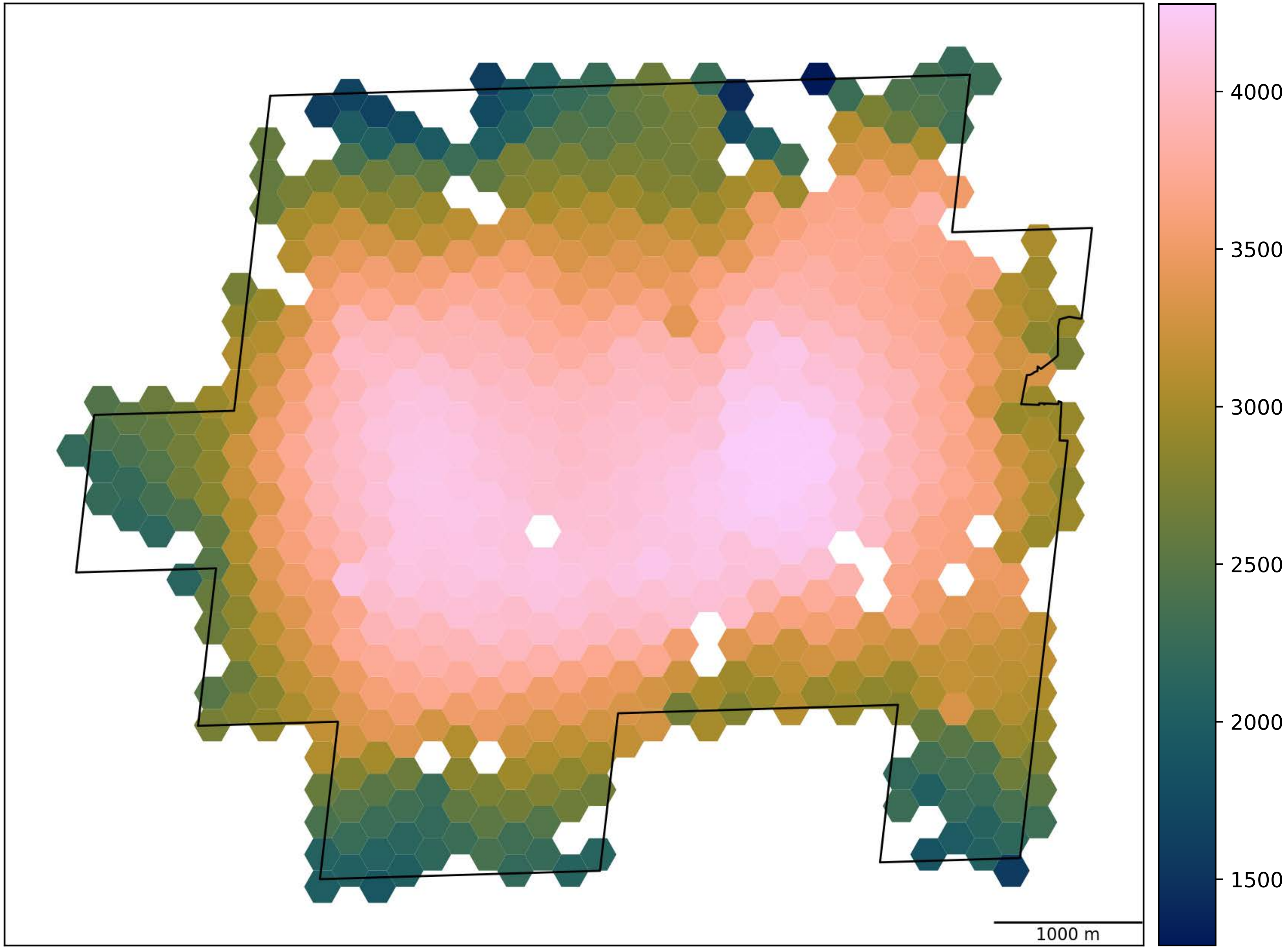

A: Estimated Mean 1000 m neighbourhood population per km² requirement for ≥80% probability of engaging in walking for transport

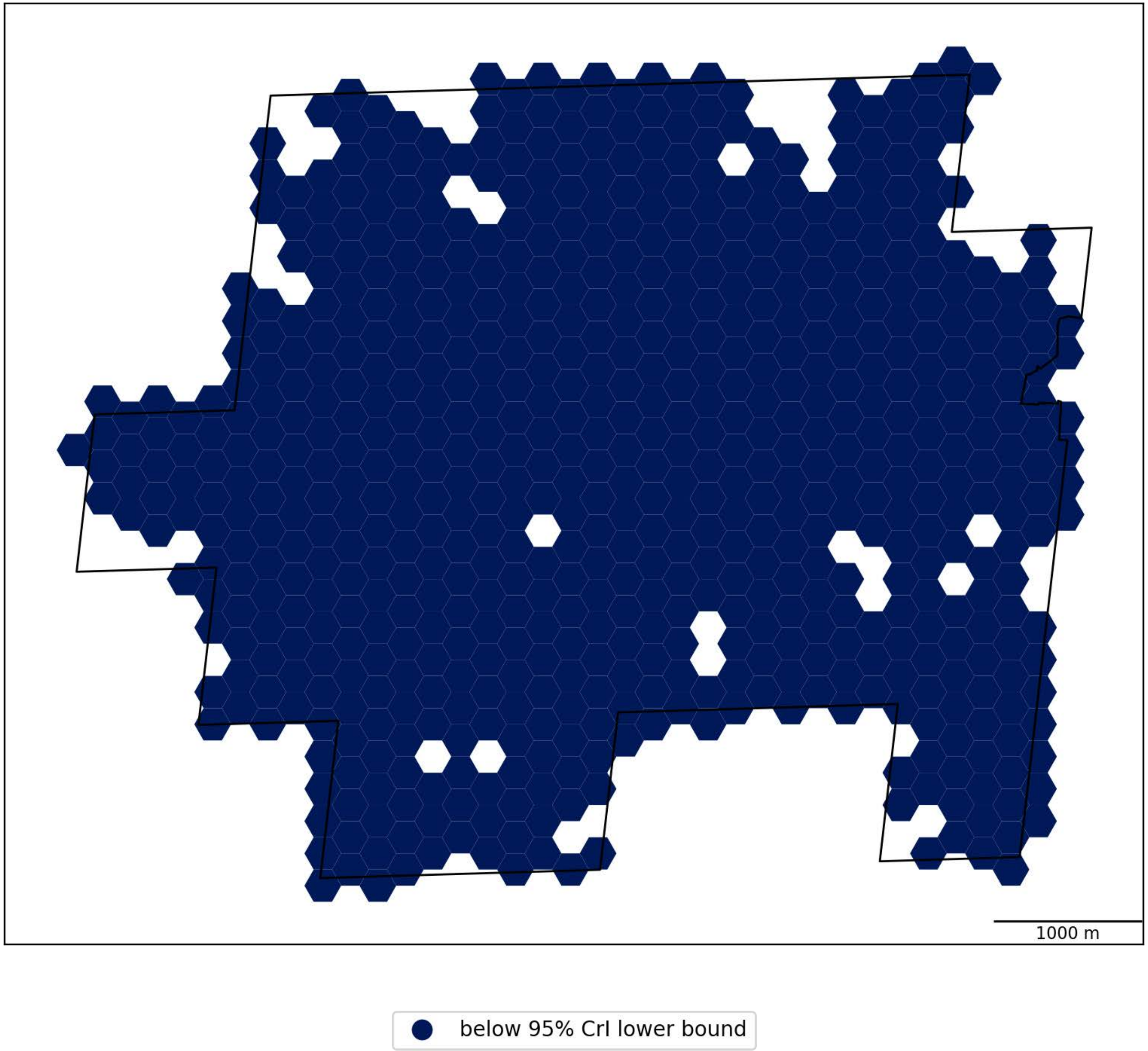

B: Estimated Mean 1000 m neighbourhood population per km² requirement for reaching the WHO's target of a ≥15% relative reduction in insufficient physical activity through walking

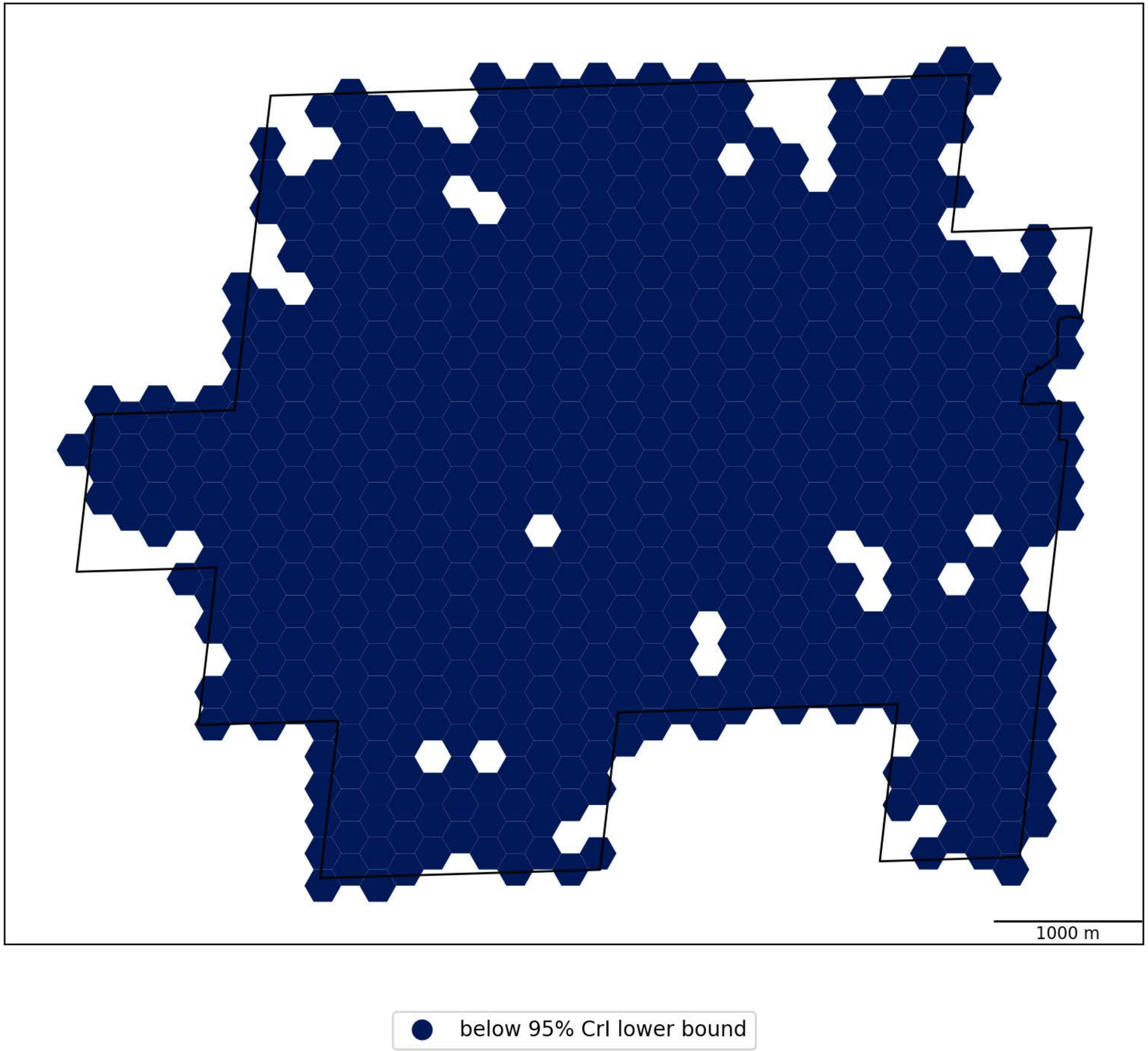

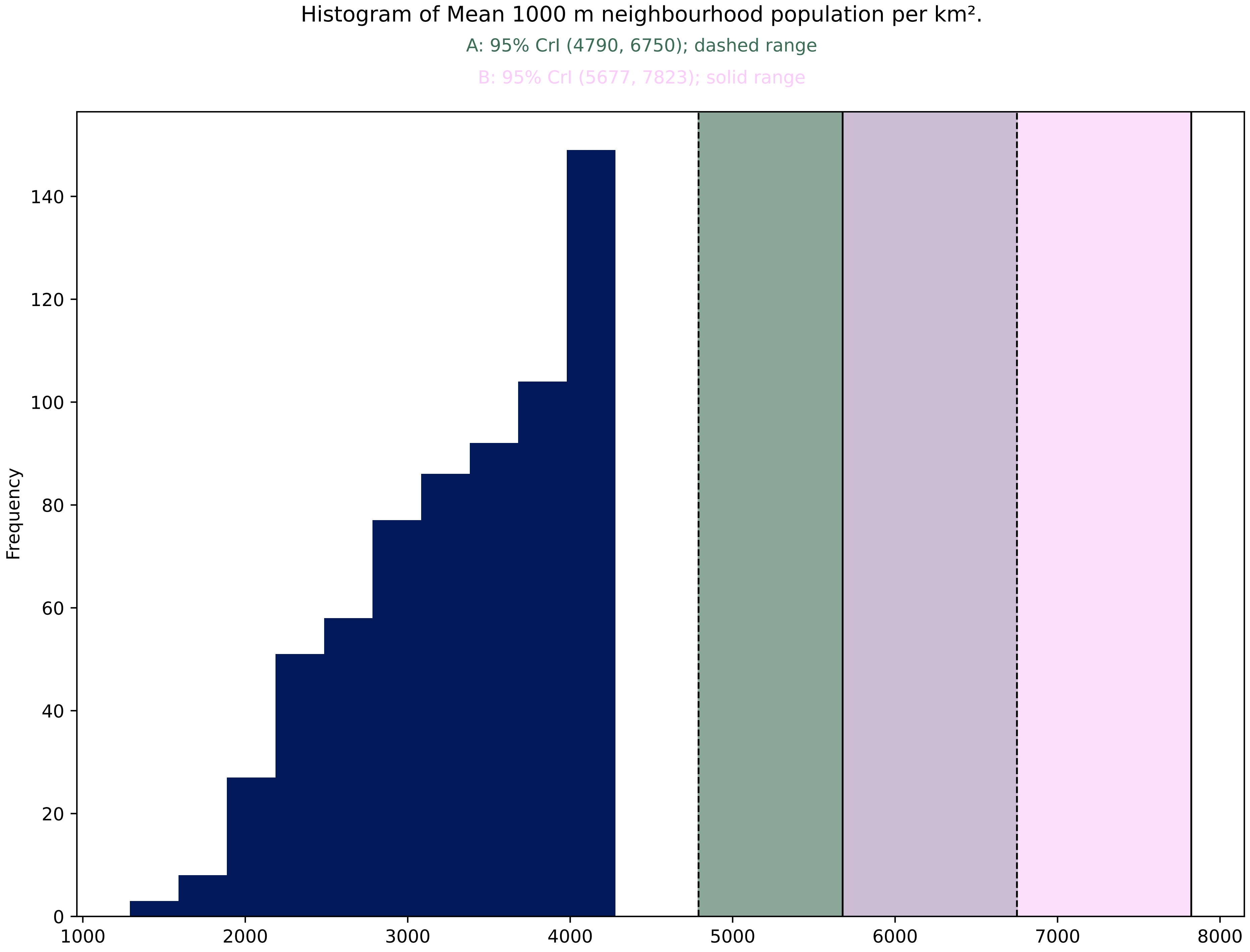
Histogram of Mean 1000 m neighbourhood population per km².
A: 95% CrI (4790, 6750); dashed range
B: 95% CrI (5677, 7823); solid range
Frequency
0
20
40
60
80
100
120
140
1000
2000
3000
4000
5000
6000
7000
8000

Mean 1000 m neighbourhood street intersections per km²

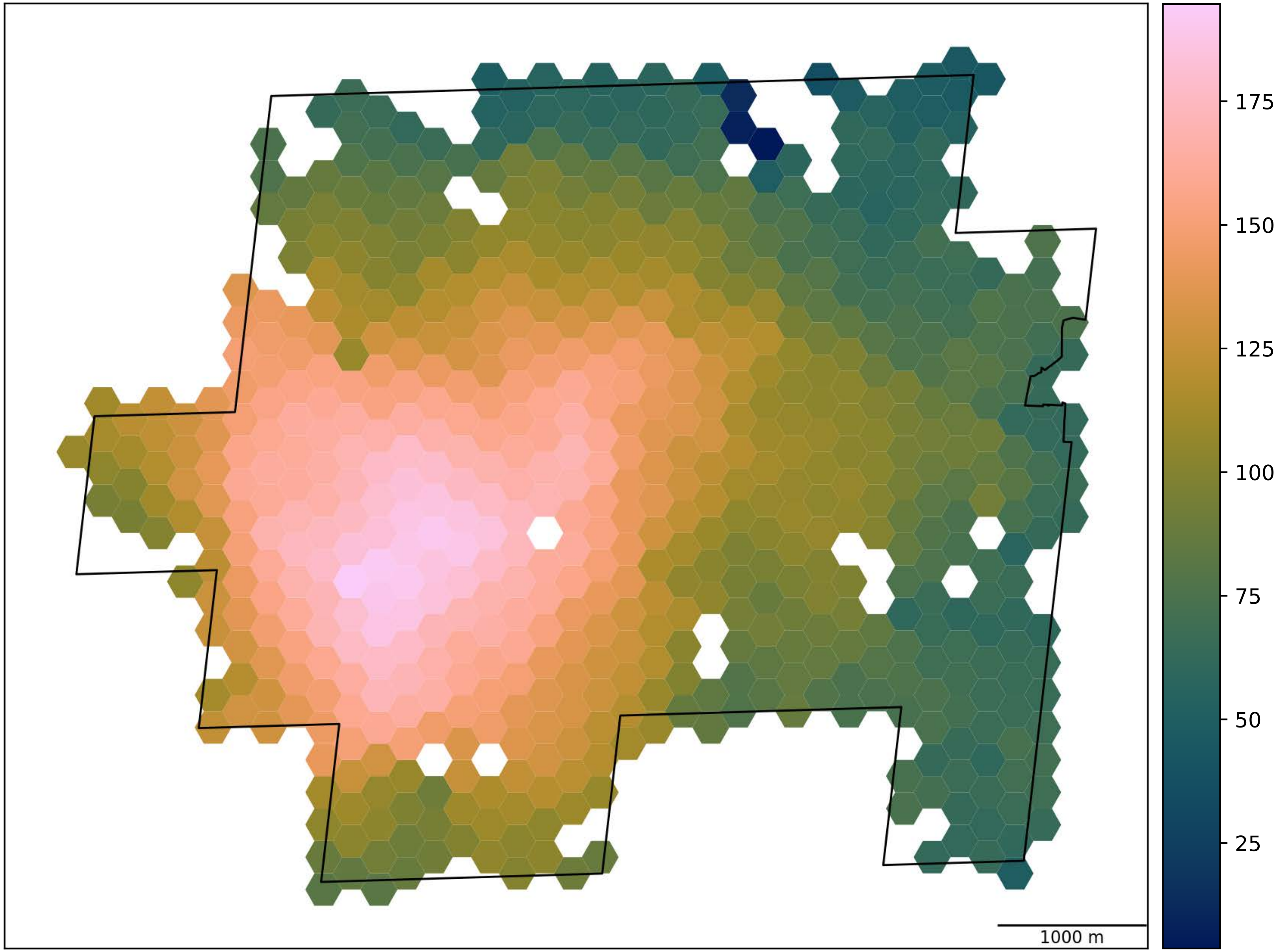

A: Estimated Mean 1000 m neighbourhood street intersections per km² requirement for ≥80% probability of engaging in walking for transport

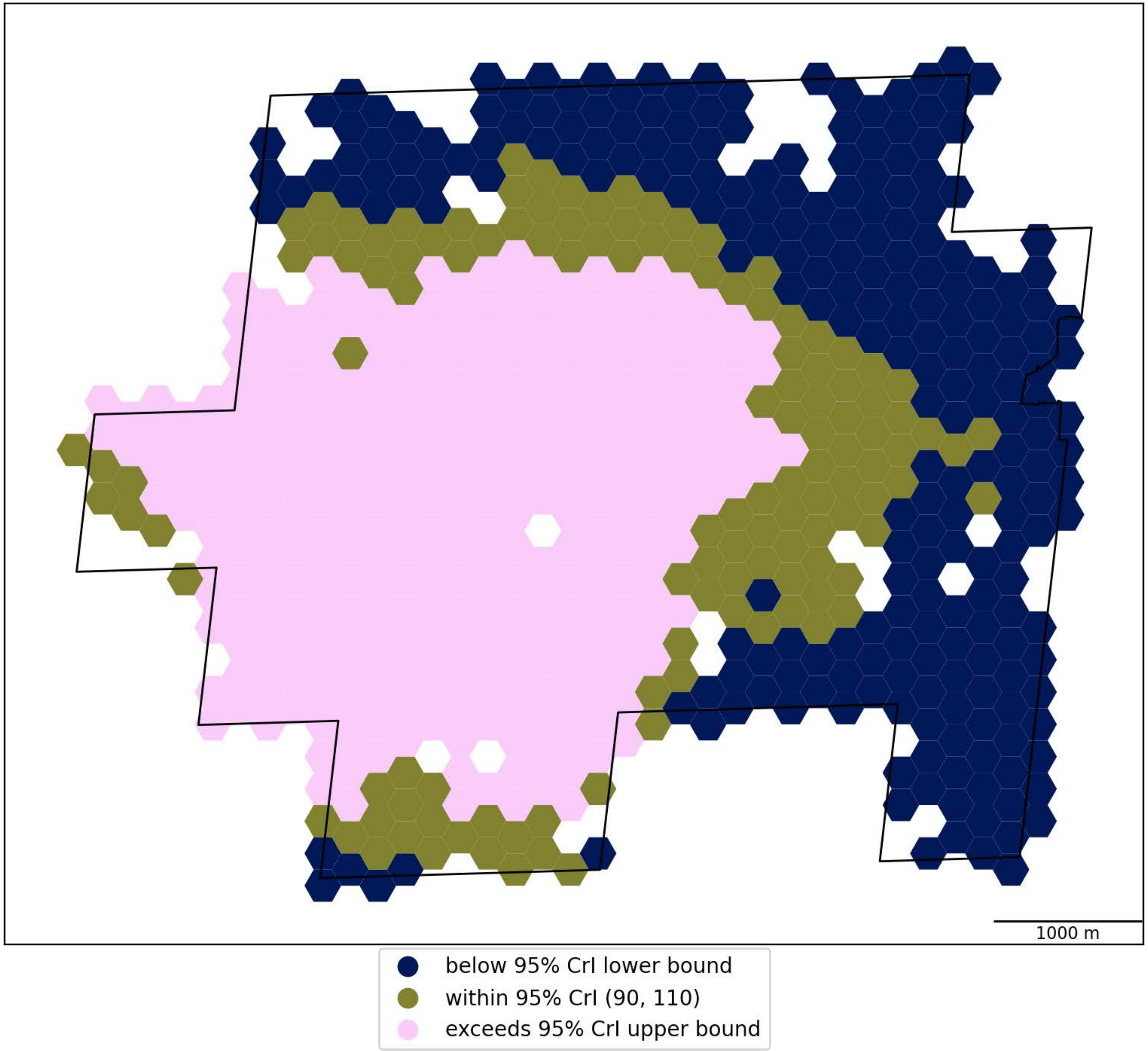

B: Estimated Mean 1000 m neighbourhood street intersections per km² requirement for reaching the WHO's target of a ≥15% relative reduction in insufficient physical activity through walking

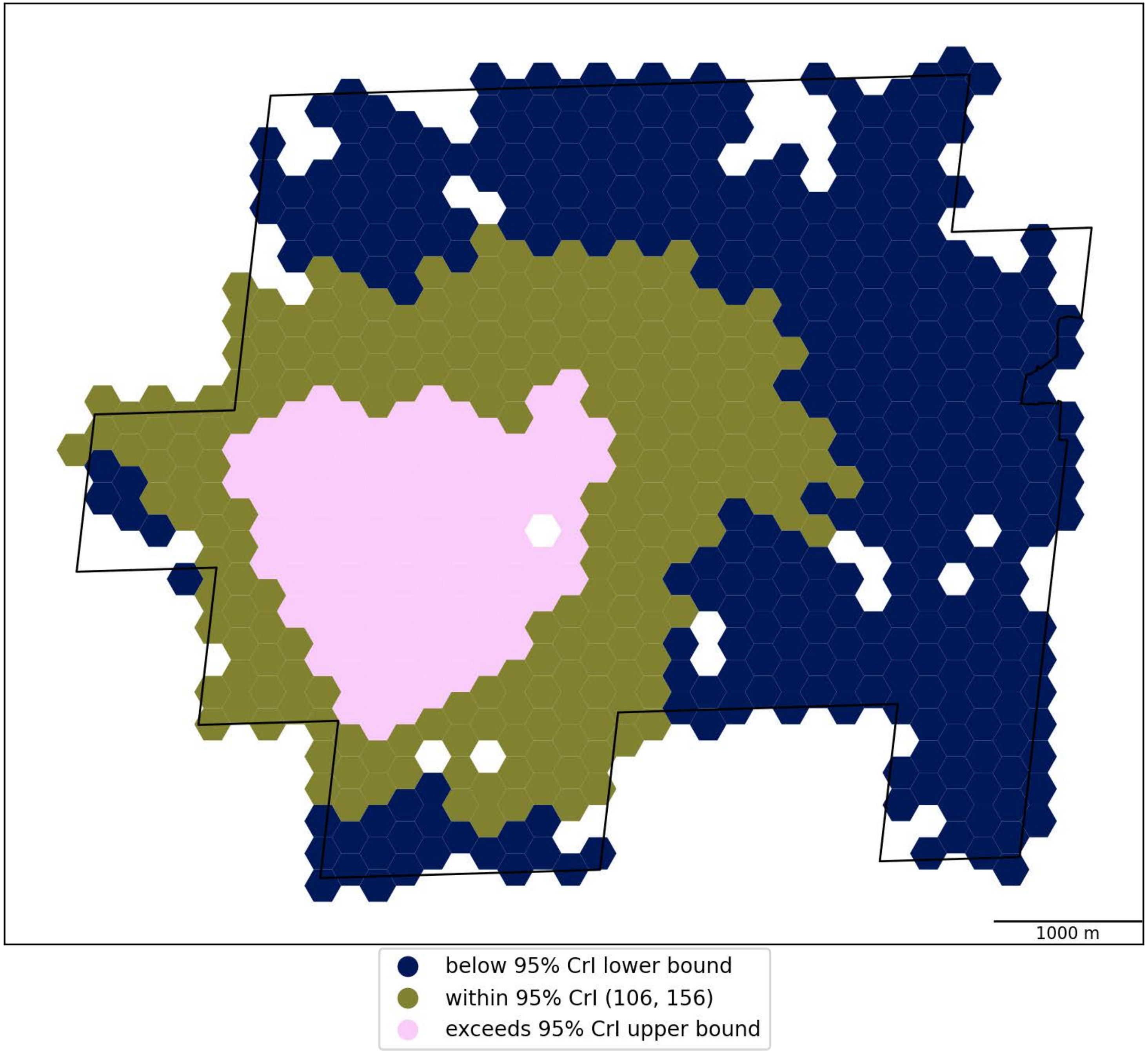

Histogram of Mean 1000 m neighbourhood street intersections per km².

A: 95% CrI (90, 110); dashed range

B: 95% CrI (106, 156); solid range

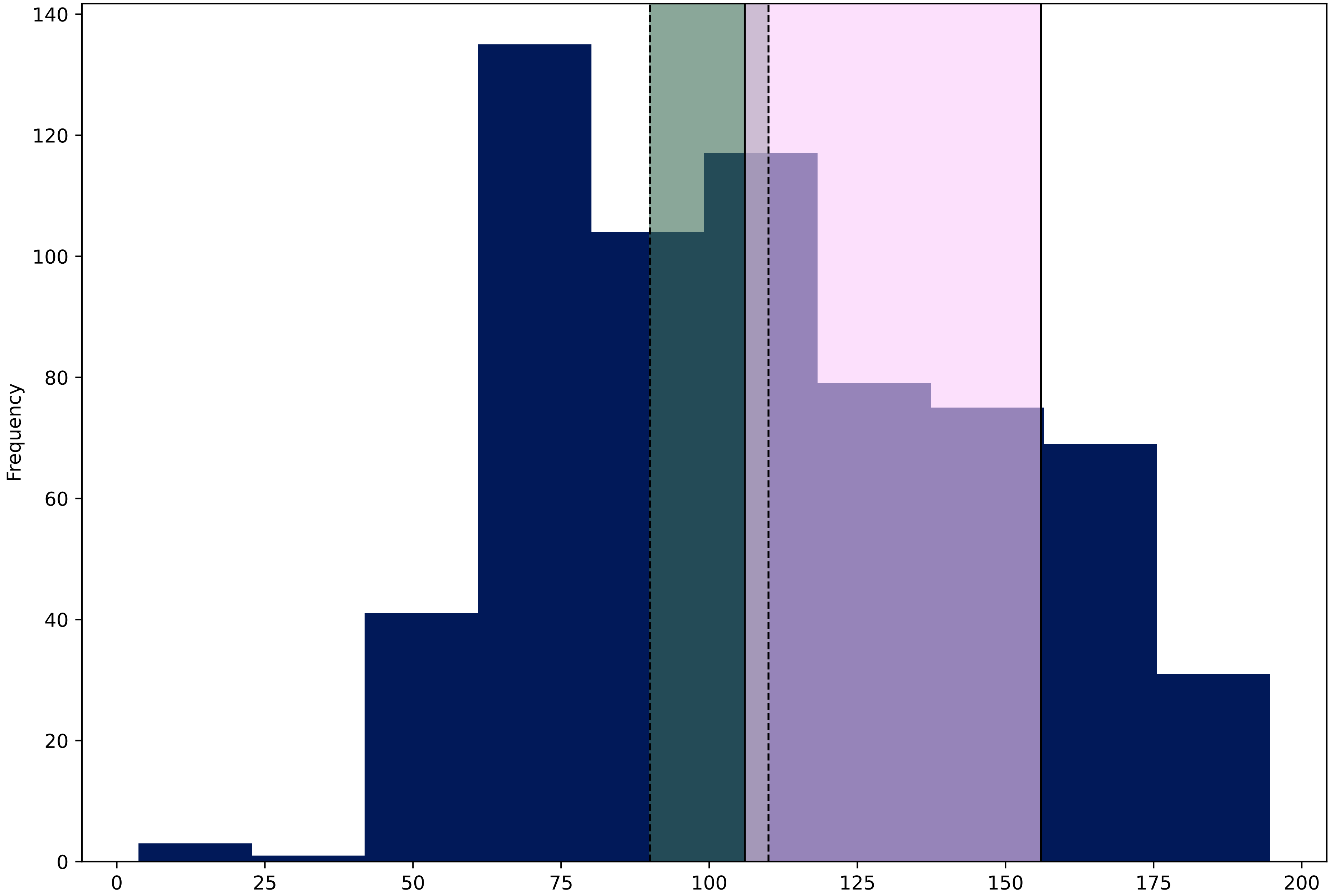

Distance to nearest public transport stops (m; up to 500m)

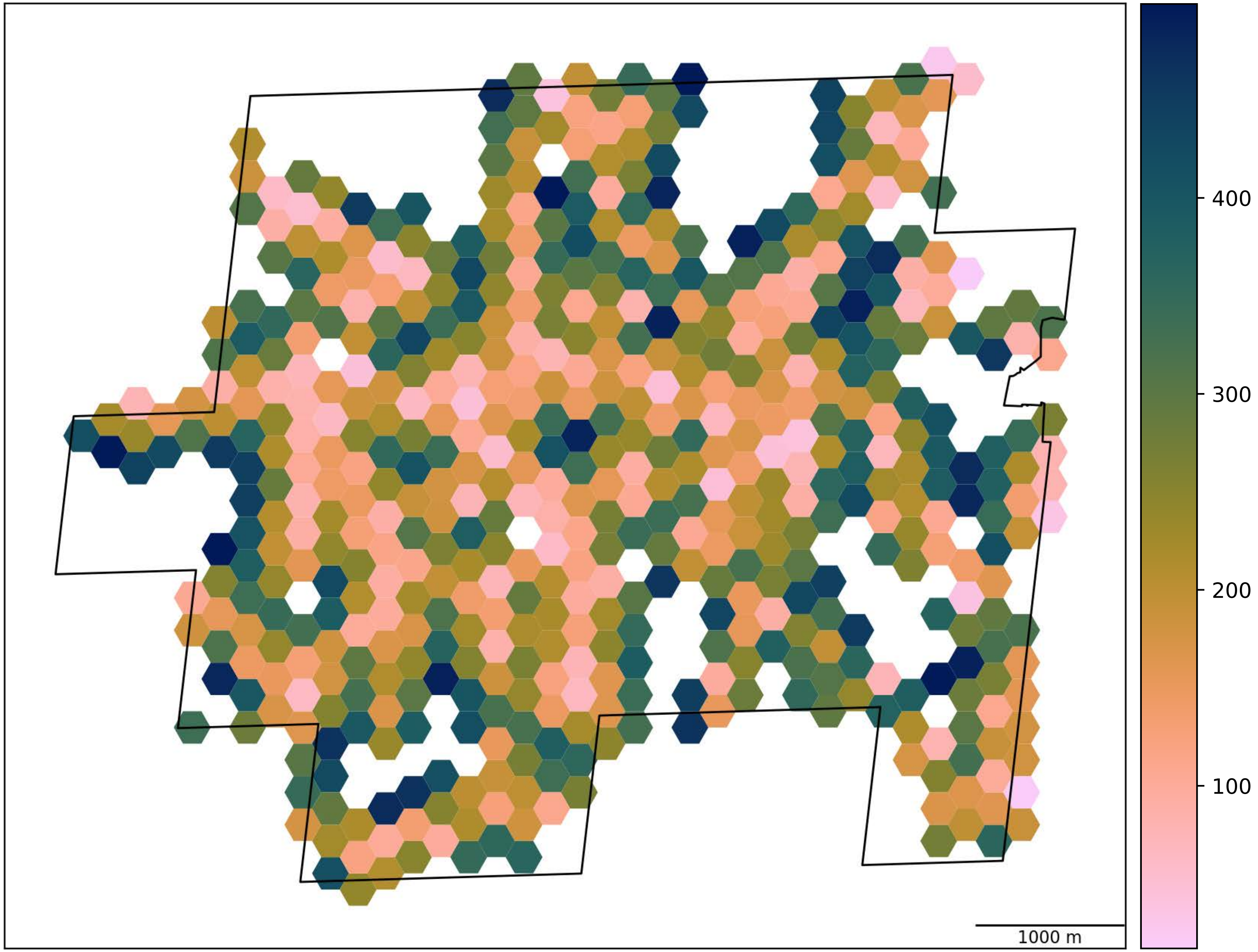

distances: Estimated Distance to nearest public transport stops (m; up to 500m) requirement for distances to destinations, measured up to a maximum distance target threshold of 500 metres

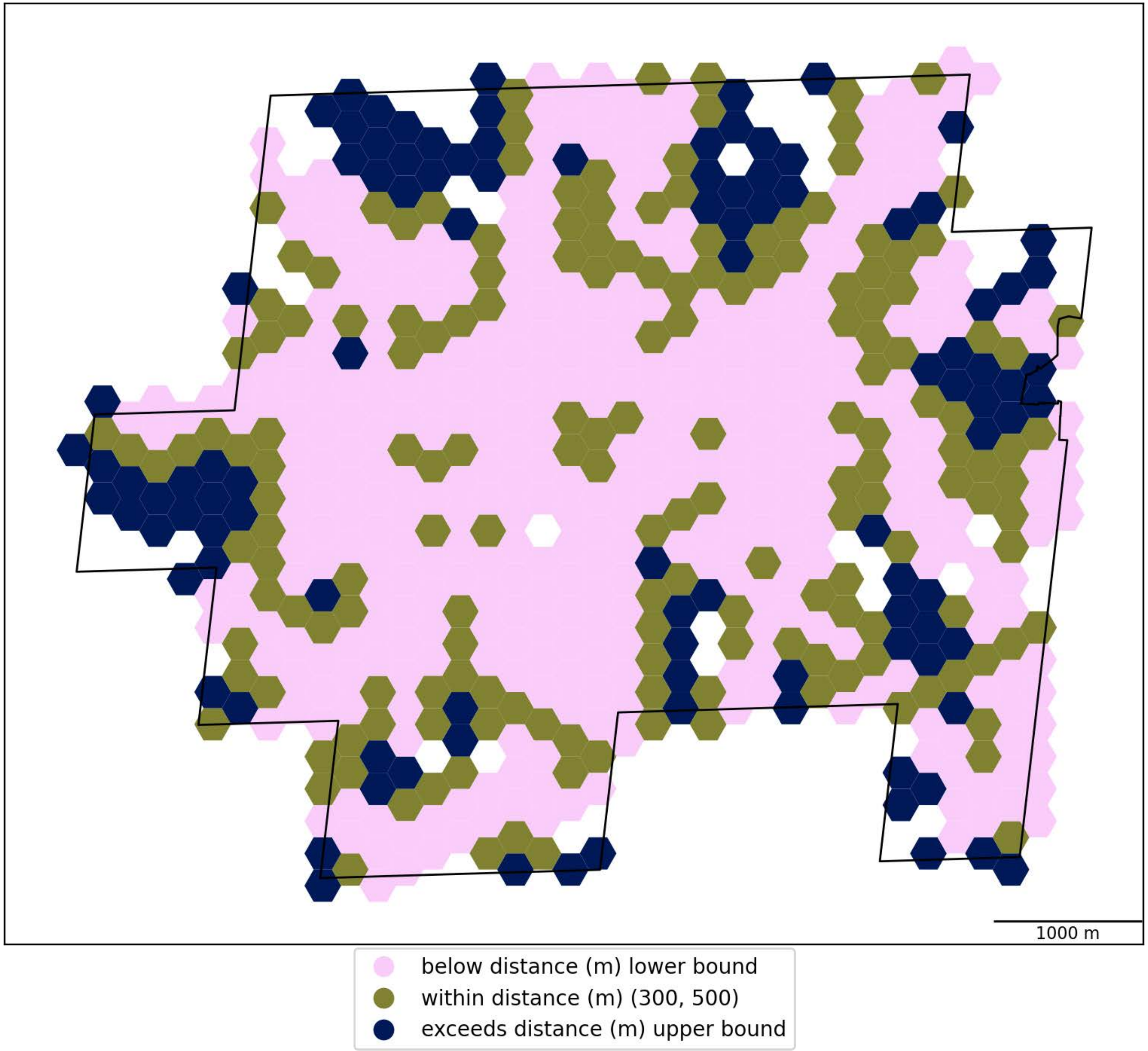

Histogram of Distance to nearest public transport stops (m; up to 500m).

distances: distance (m) (300, 500); dashed range

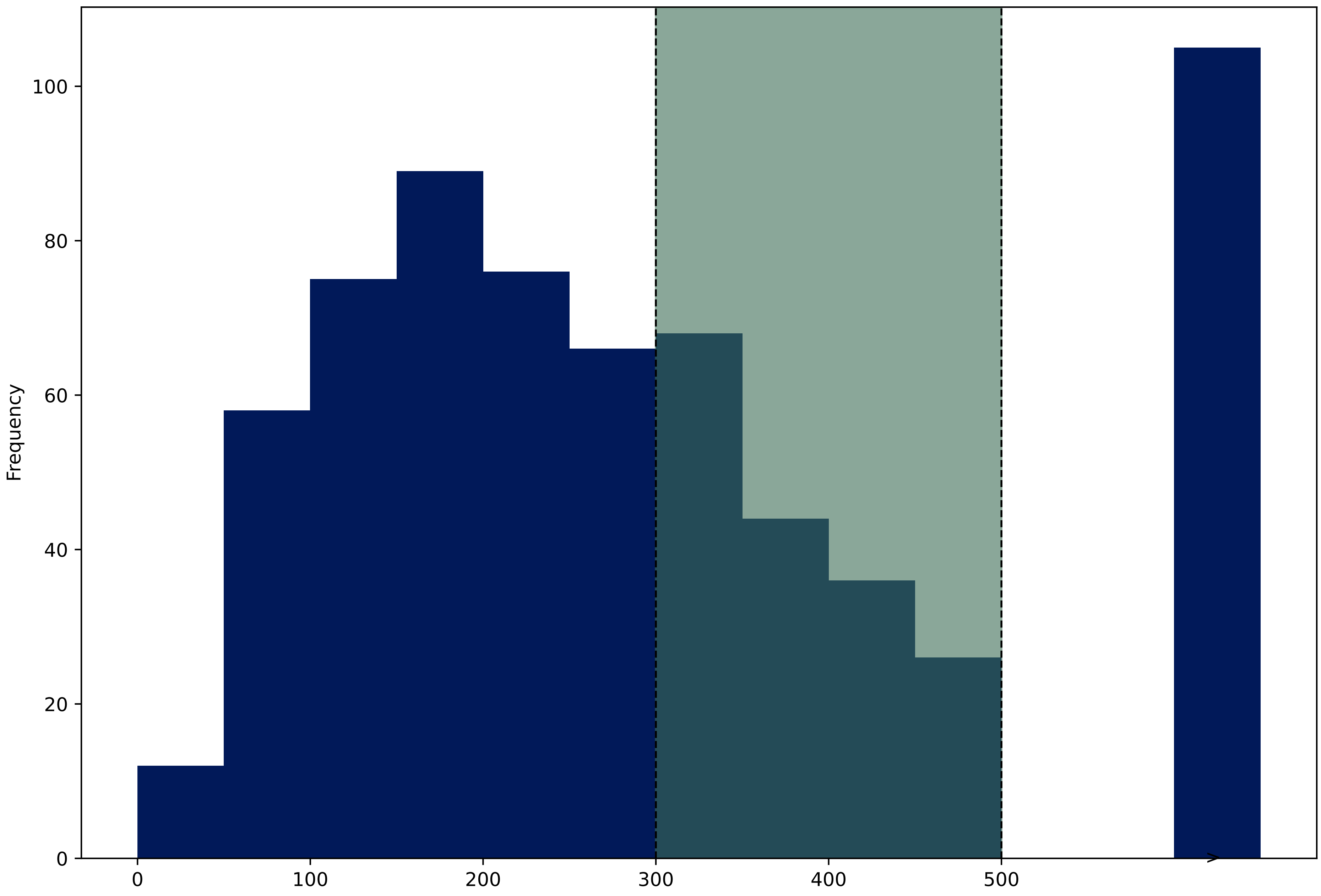

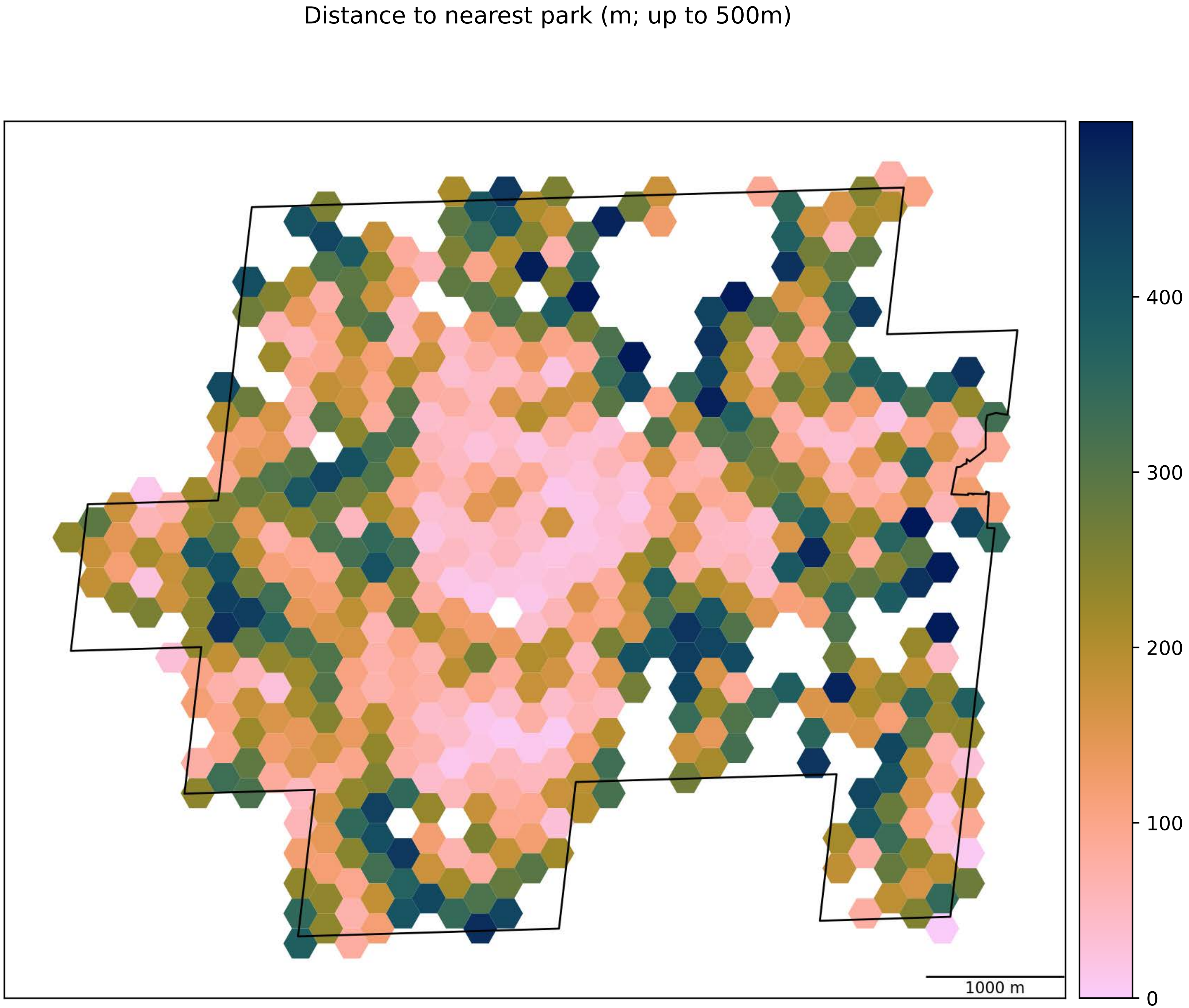
Distance to nearest park (m; up to 500m)
400
300
200
100
0
1000 m

distances: Estimated Distance to nearest park (m; up to 500m) requirement for distances to destinations, measured up to a maximum distance target threshold of 500 metres

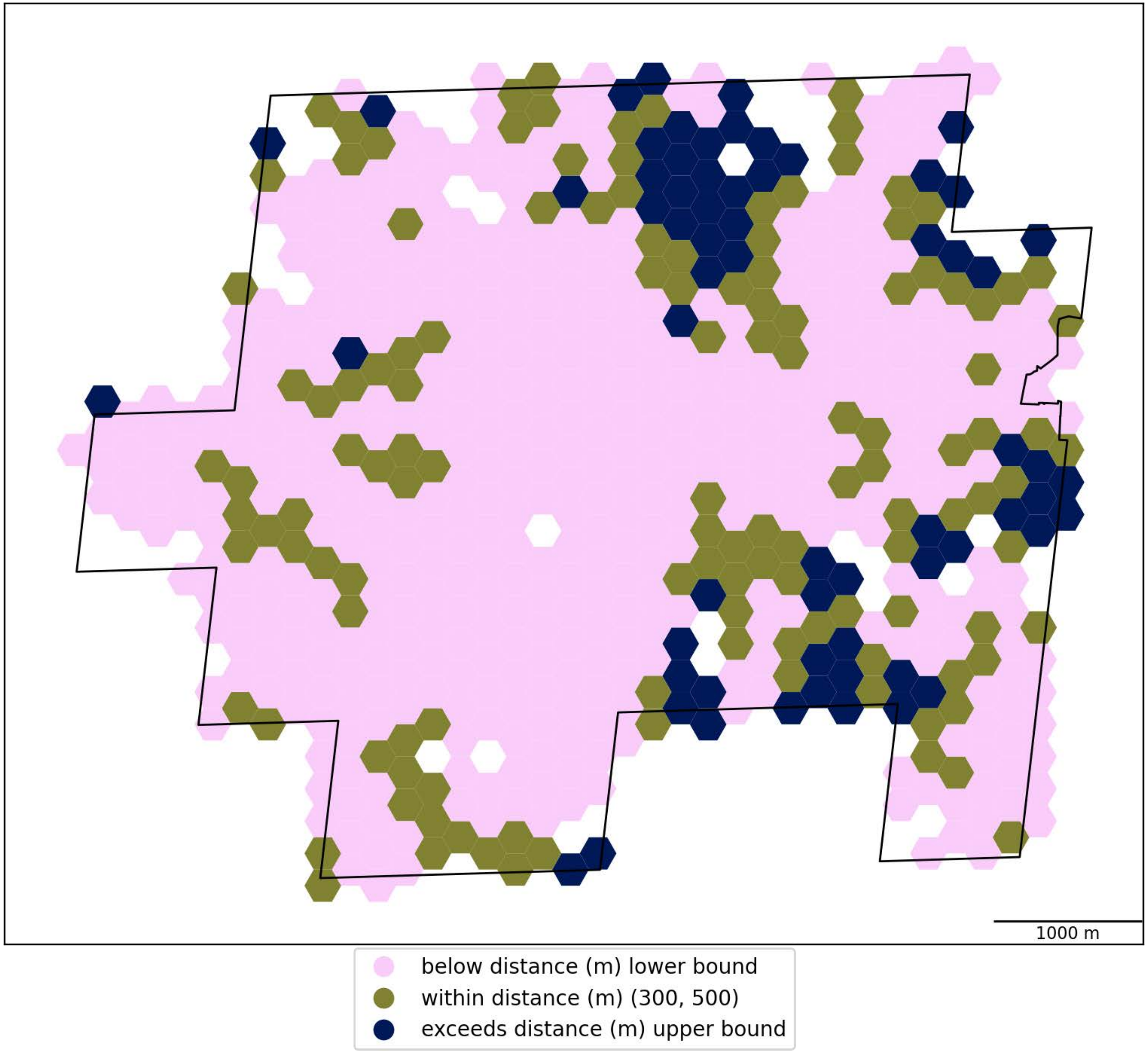

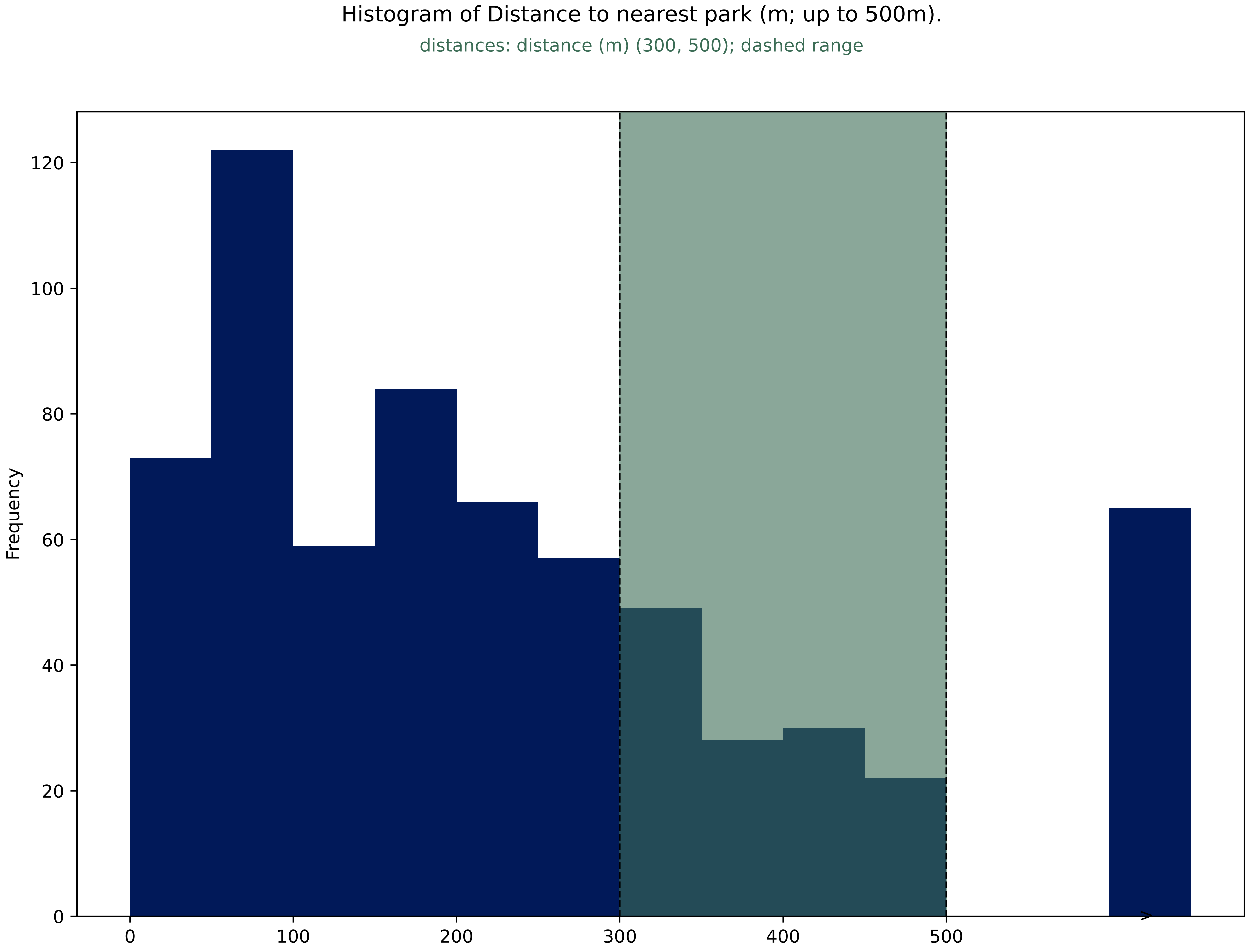

Histogram of Distance to nearest park (m; up to 500m).
distances: distance (m) (300, 500); dashed range
Frequency
0
20
40
60
80
100
120
0
100
200
300
400
500
>

Europe, Denmark, Odense

Satellite imagery of urban study region (Bing)

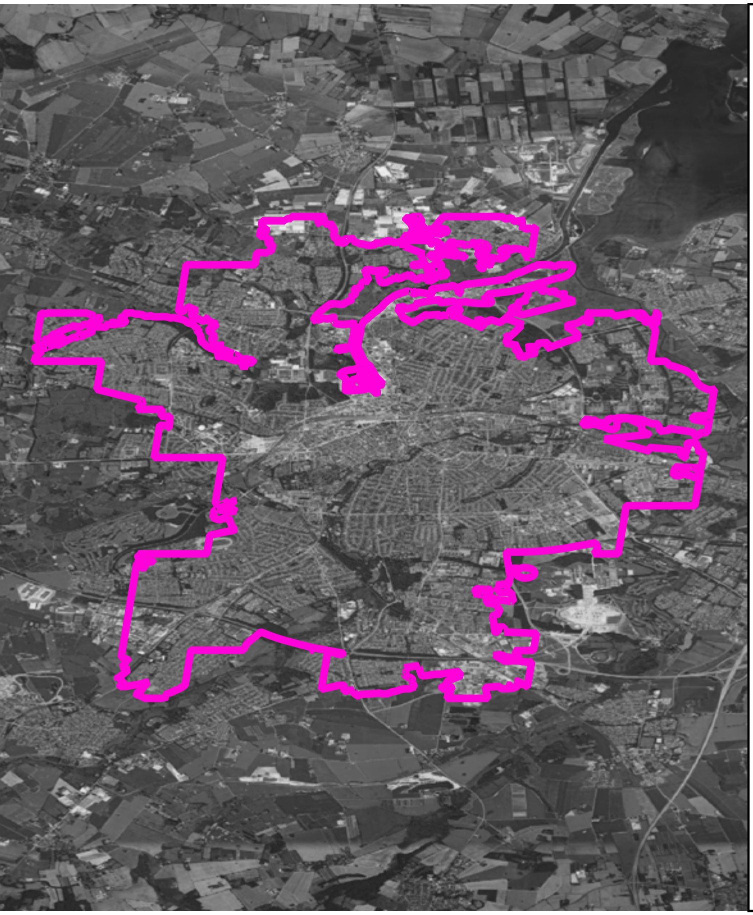

Walkability, relative to city

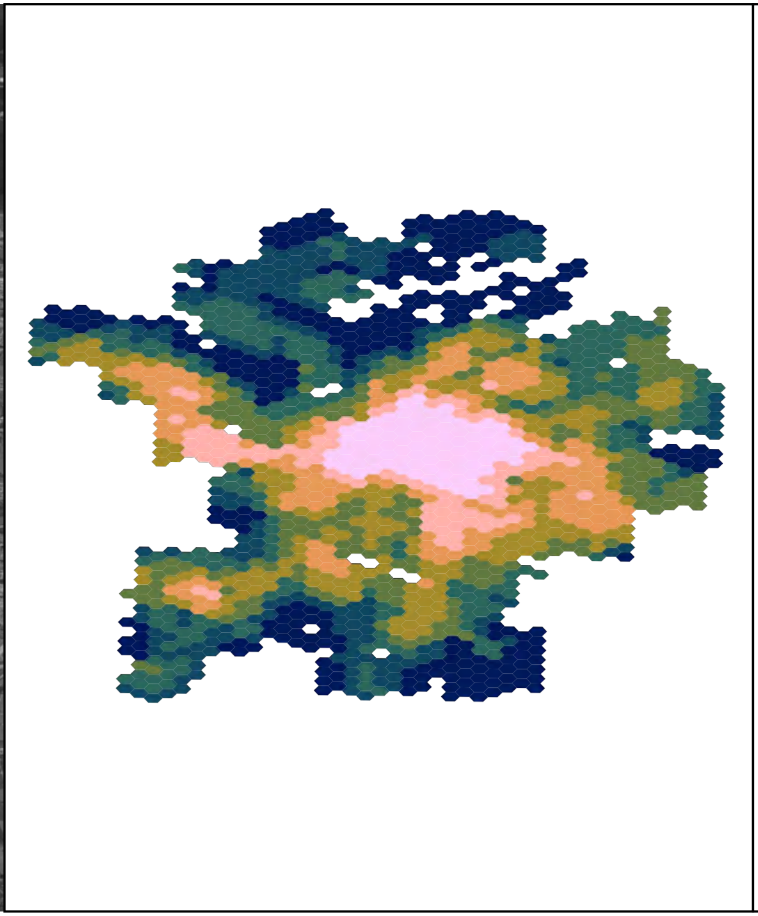

Walkability, relative to 25 global cities

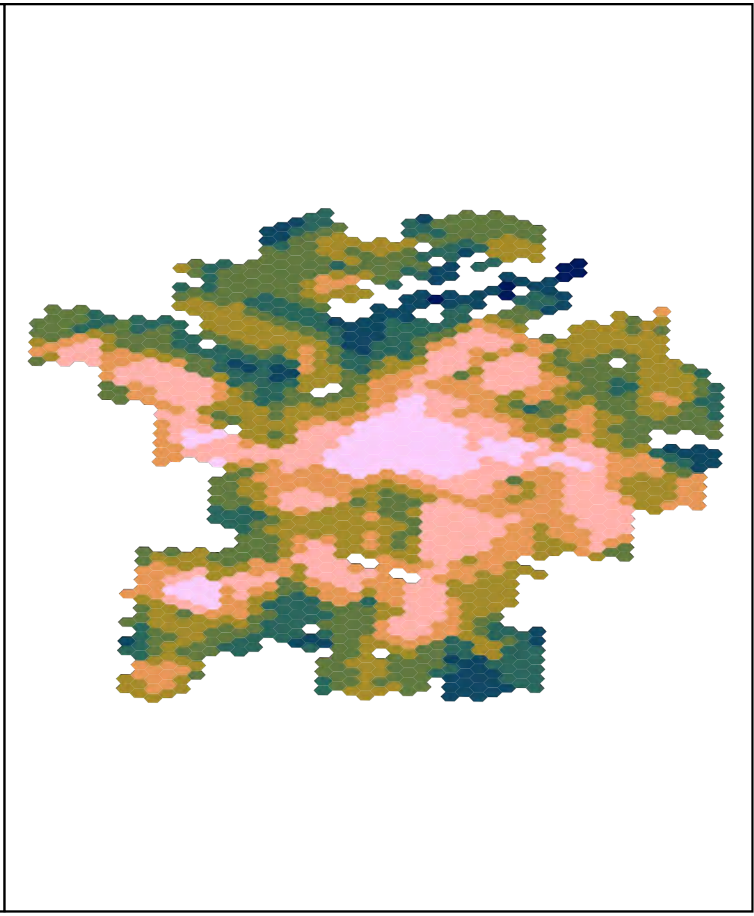

Urban boundary

0 4 8 km

Walkability score

- <-3
- -3 to -2
- -2 to -1
- -1 to 0
- 0 to 1
- 1 to 2
- 2 to 3
- ≥3

Walkability relative to all cities by component variables (2D histograms), and overall (histogram)

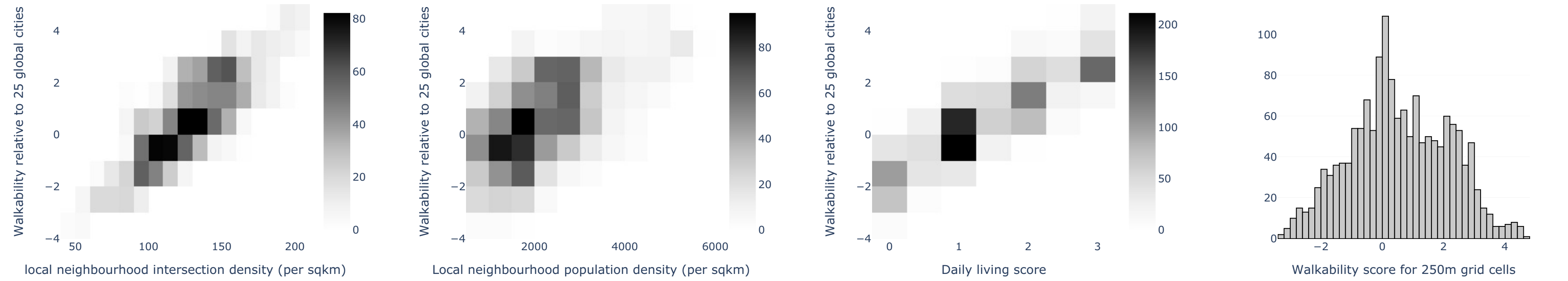

Mean 1000 m neighbourhood population per km²

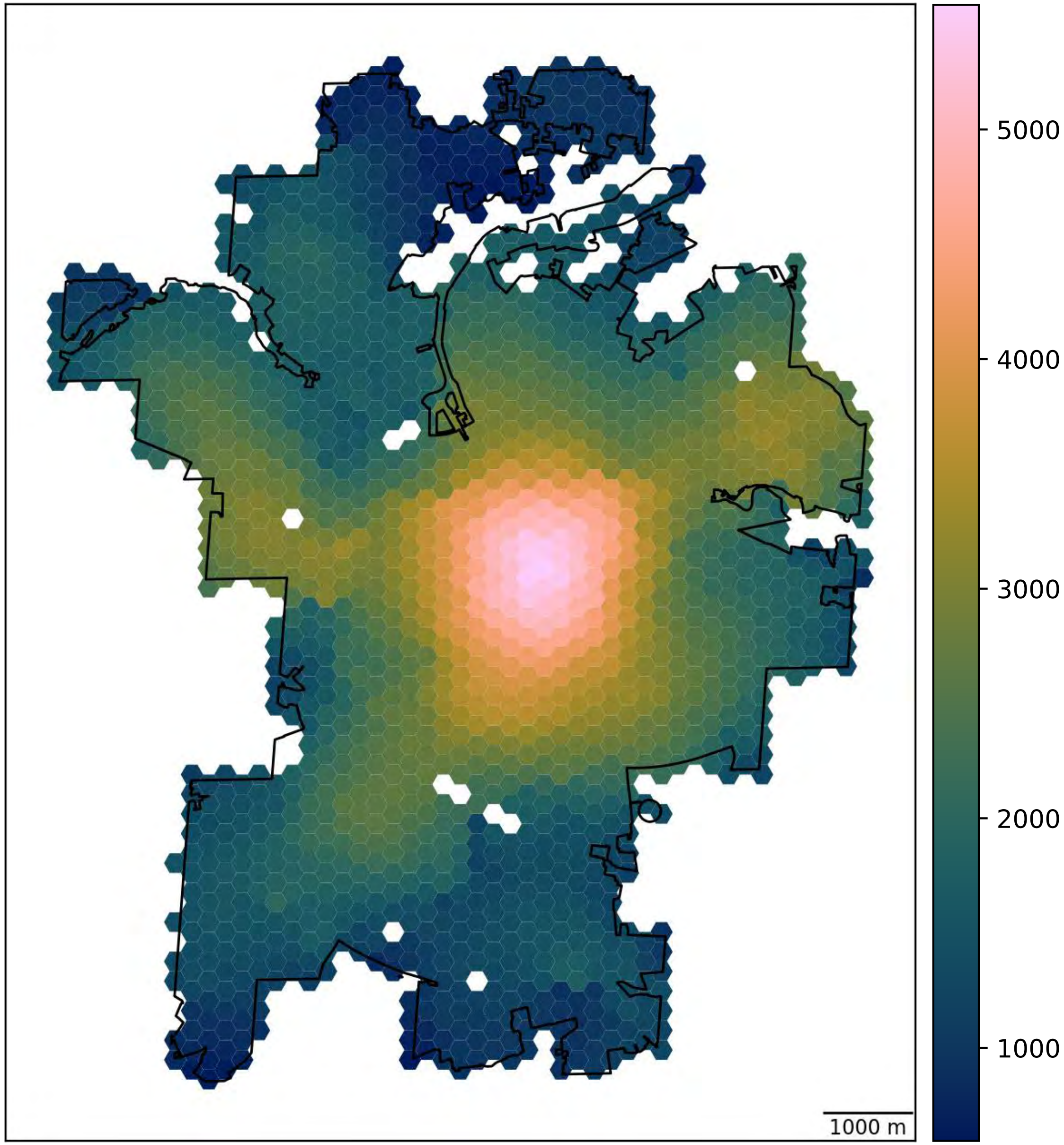

A: Estimated Mean 1000 m neighbourhood population per km² requirement for ≥80% probability of engaging in walking for transport

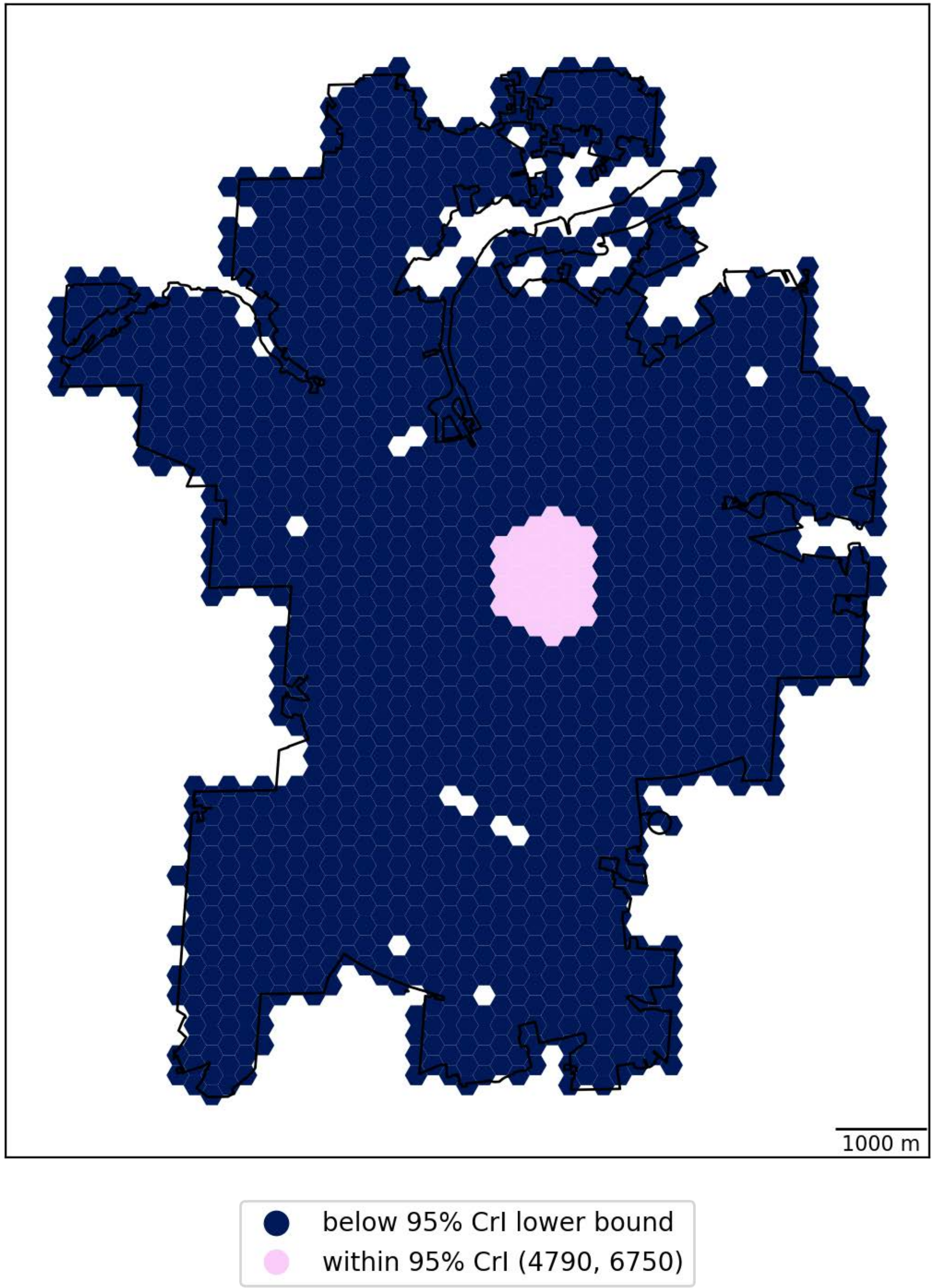

B: Estimated Mean 1000 m neighbourhood population per km² requirement for reaching the WHO's target of a ≥15% relative reduction in insufficient physical activity through walking

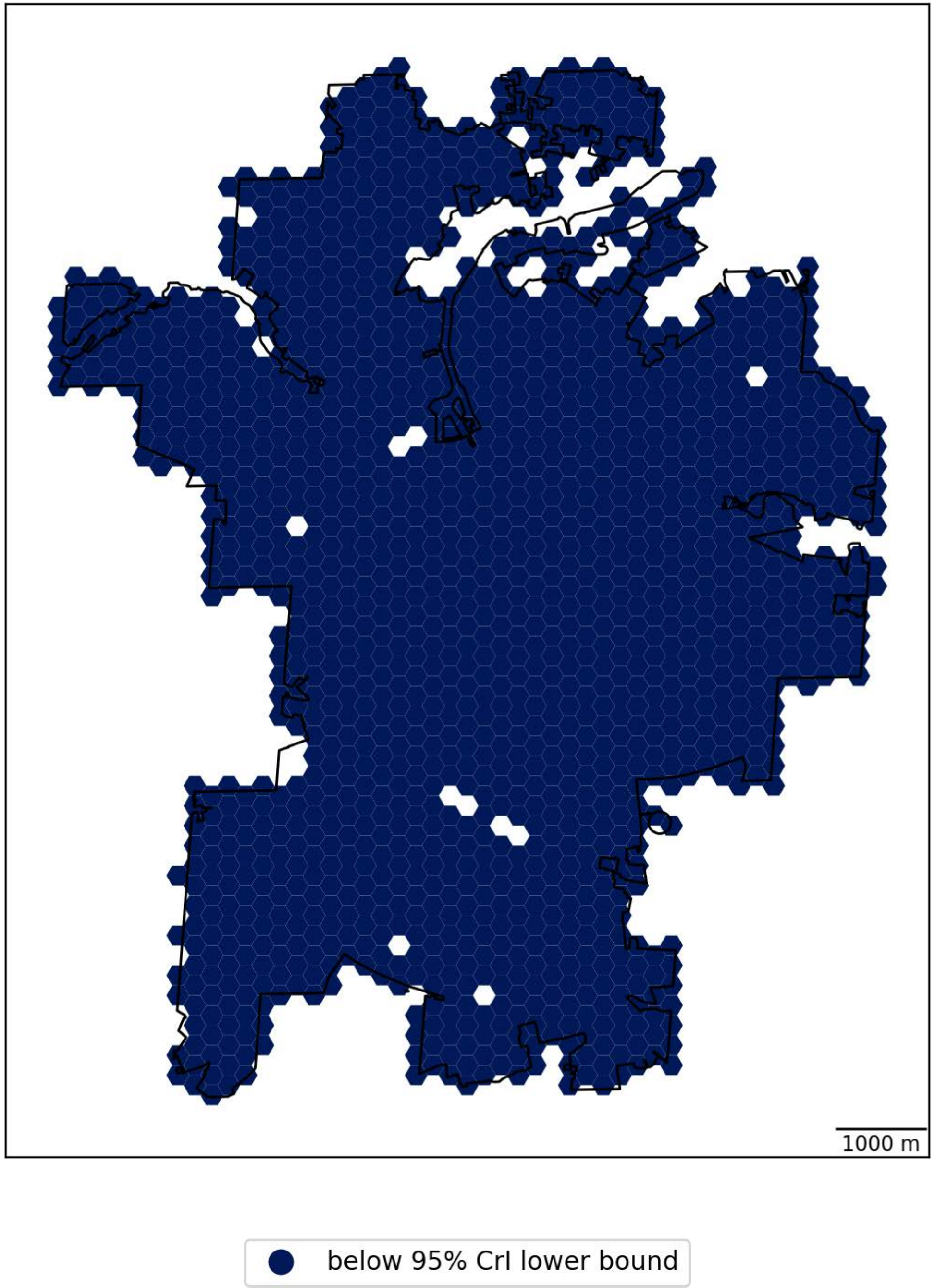

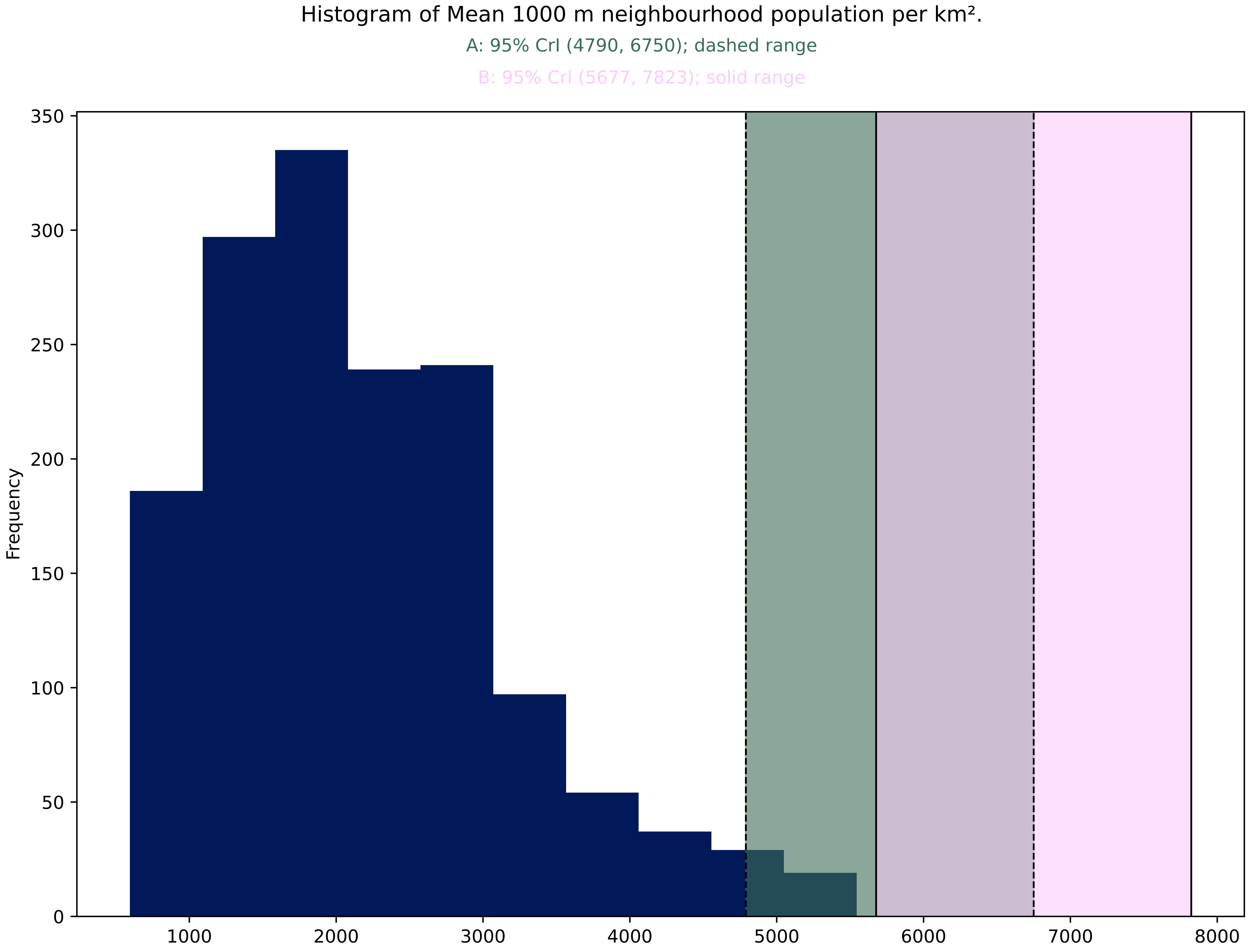

Histogram of Mean 1000 m neighbourhood population per km².
A: 95% CrI (4790, 6750); dashed range
B: 95% CrI (5677, 7823); solid range
Frequency
0
50
100
150
200
250
300
350
1000
2000
3000
4000
5000
6000
7000
8000

Mean 1000 m neighbourhood street intersections per km²

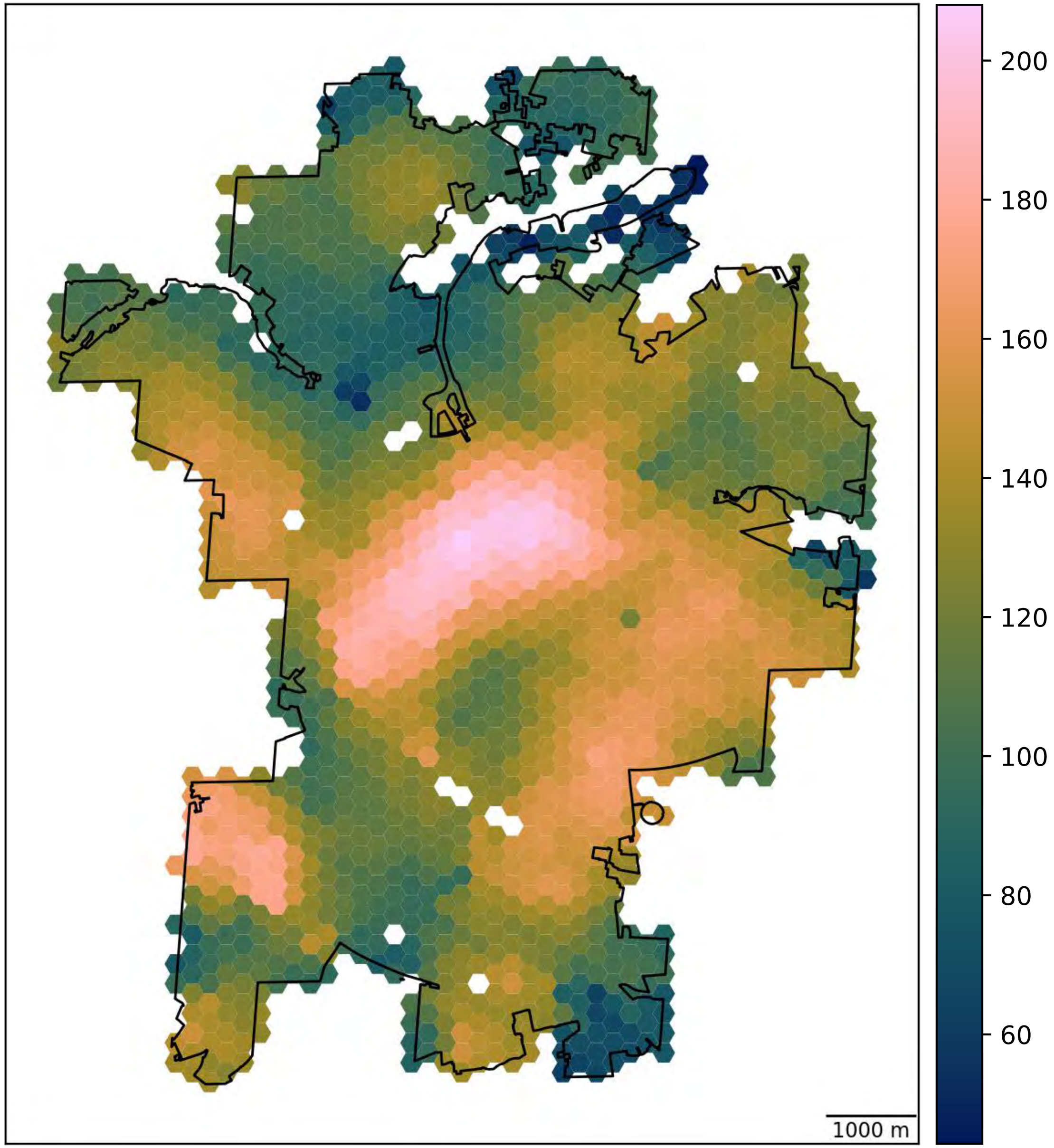

A: Estimated Mean 1000 m neighbourhood street intersections per km² requirement for ≥80% probability of engaging in walking for transport

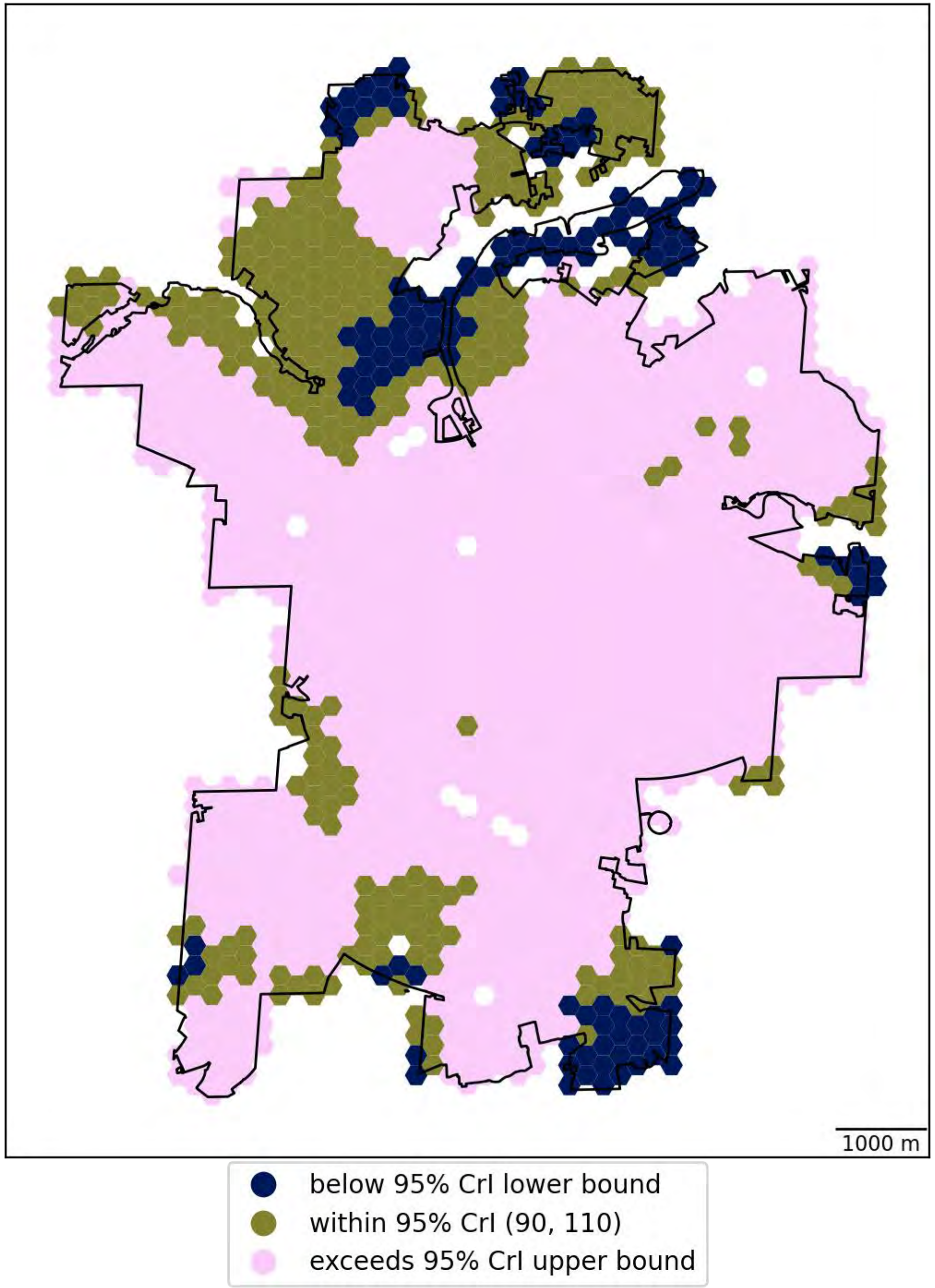

B: Estimated Mean 1000 m neighbourhood street intersections per km² requirement for reaching the WHO's target of a ≥15% relative reduction in insufficient physical activity through walking

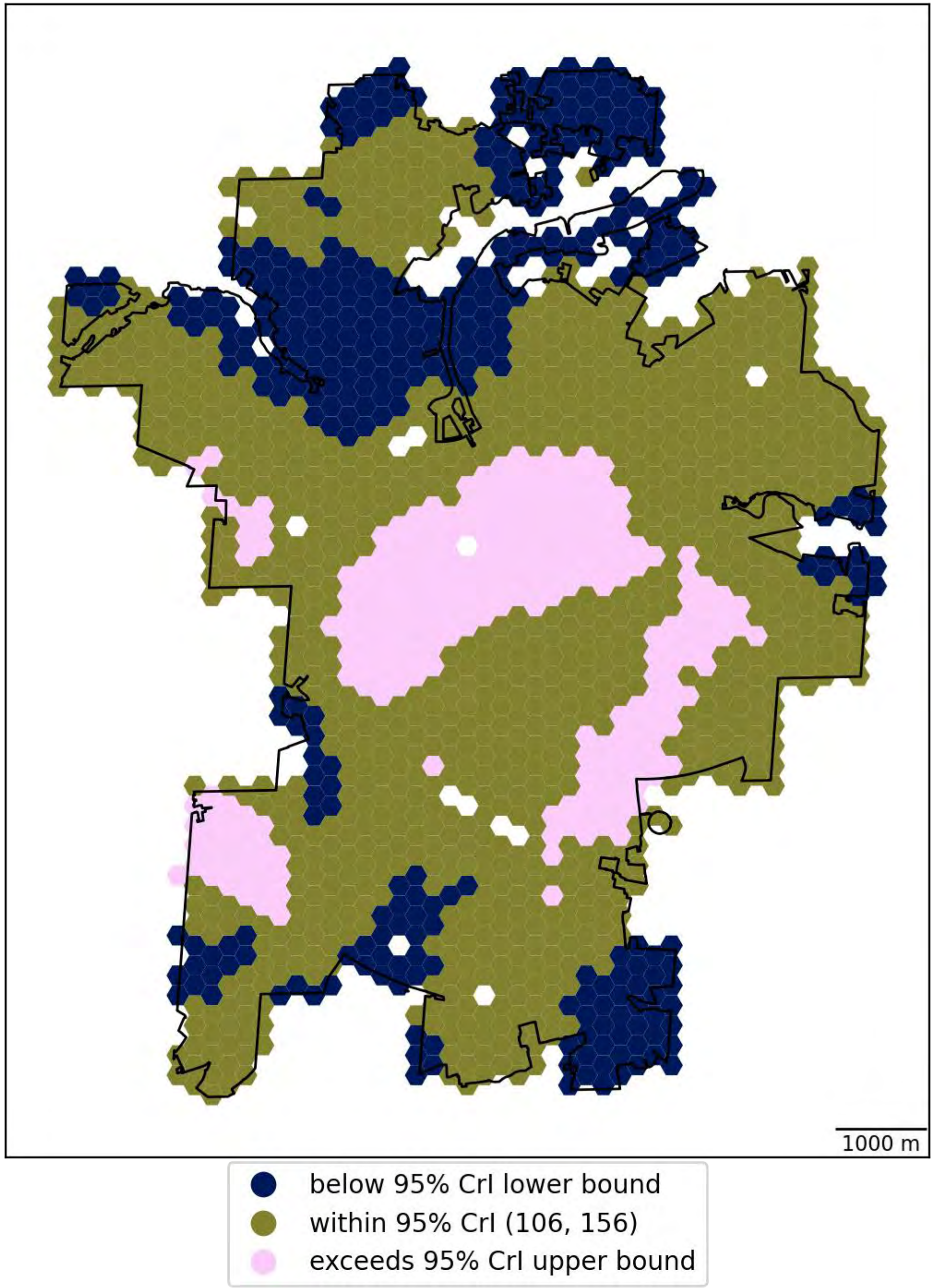

Histogram of Mean 1000 m neighbourhood street intersections per km².

A: 95% CrI (90, 110); dashed range

B: 95% CrI (106, 156); solid range

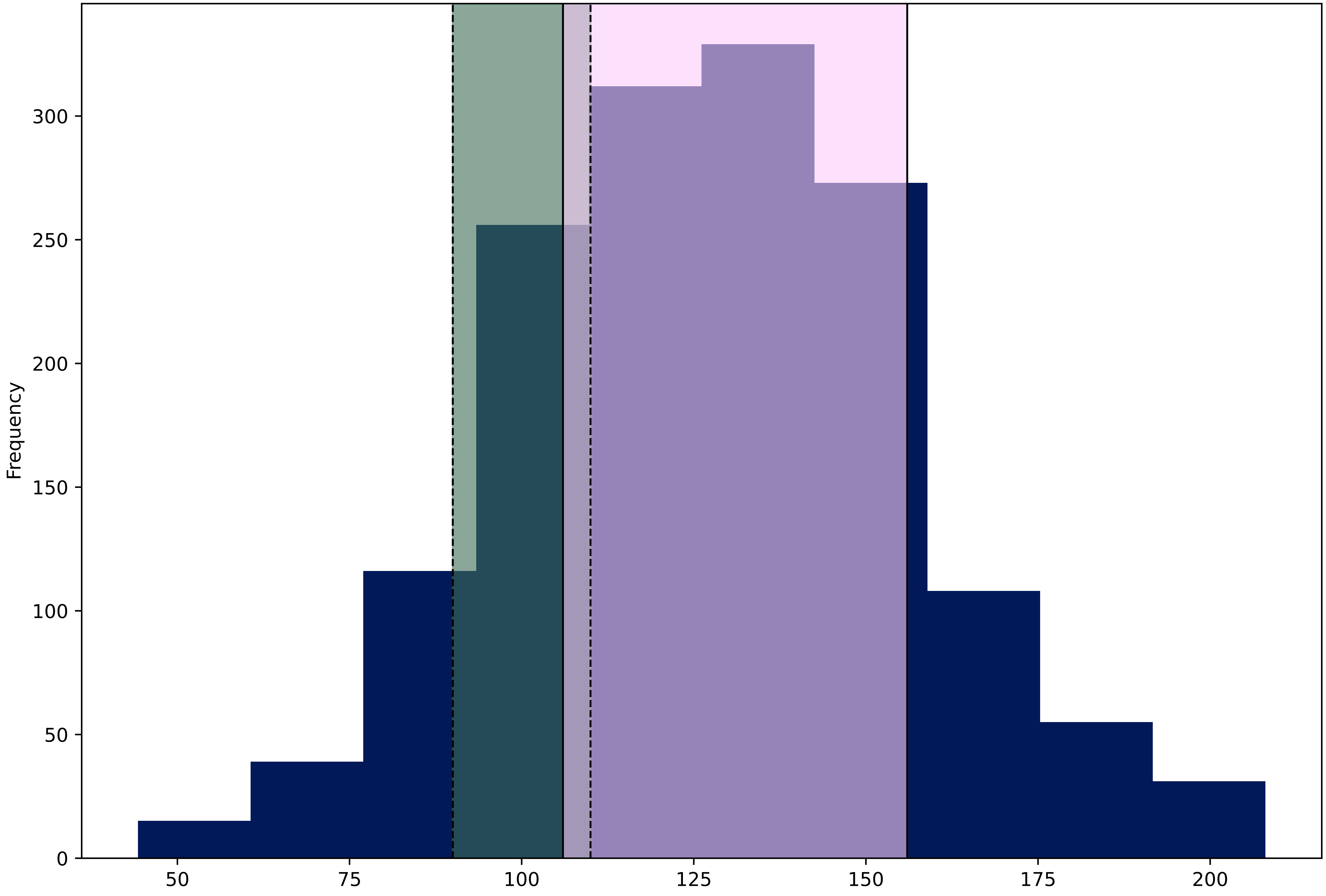

Distance to nearest public transport stops (m; up to 500m)

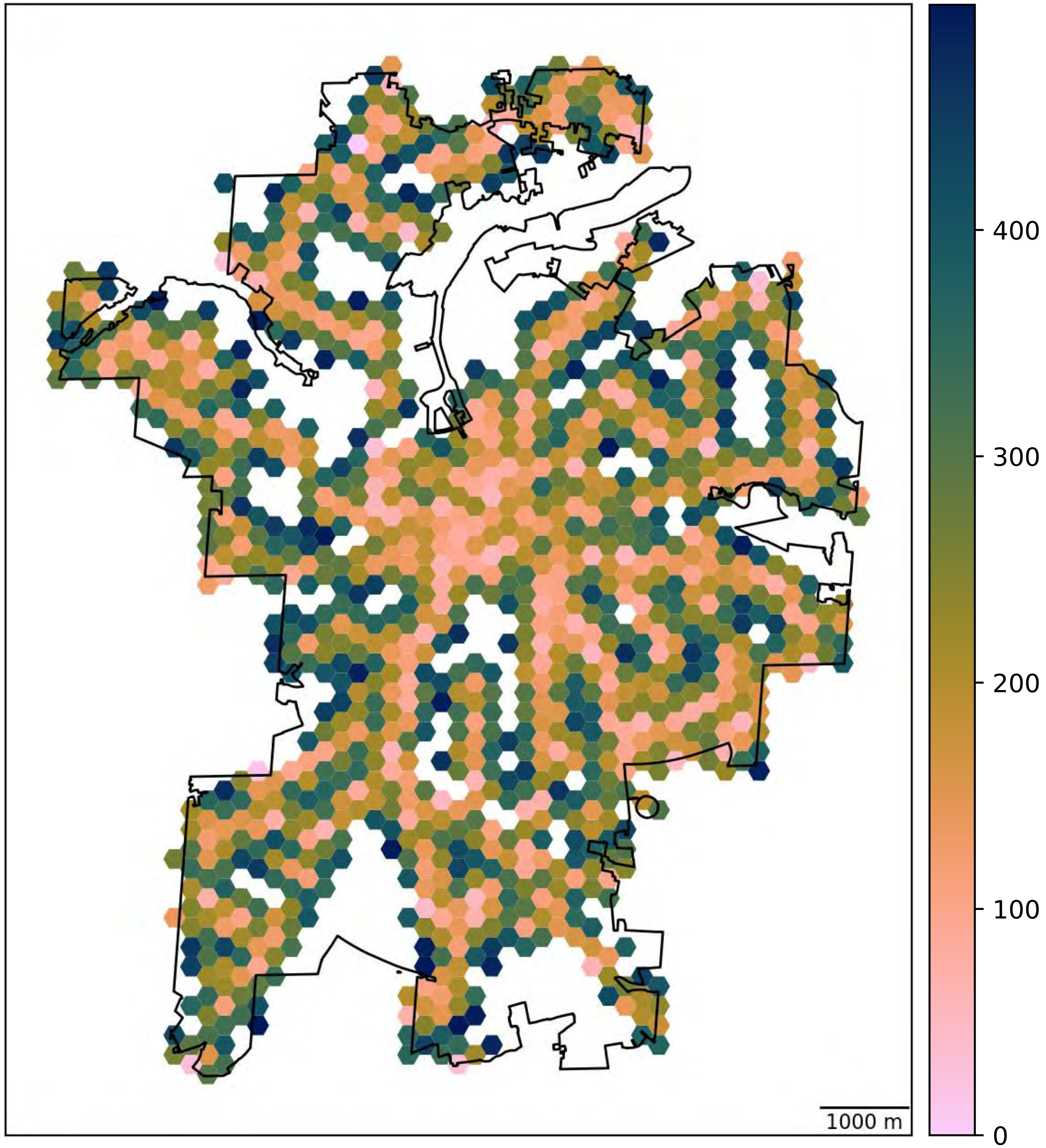

distances: Estimated Distance to nearest public transport stops (m; up to 500m) requirement for distances to destinations, measured up to a maximum distance target threshold of 500 metres

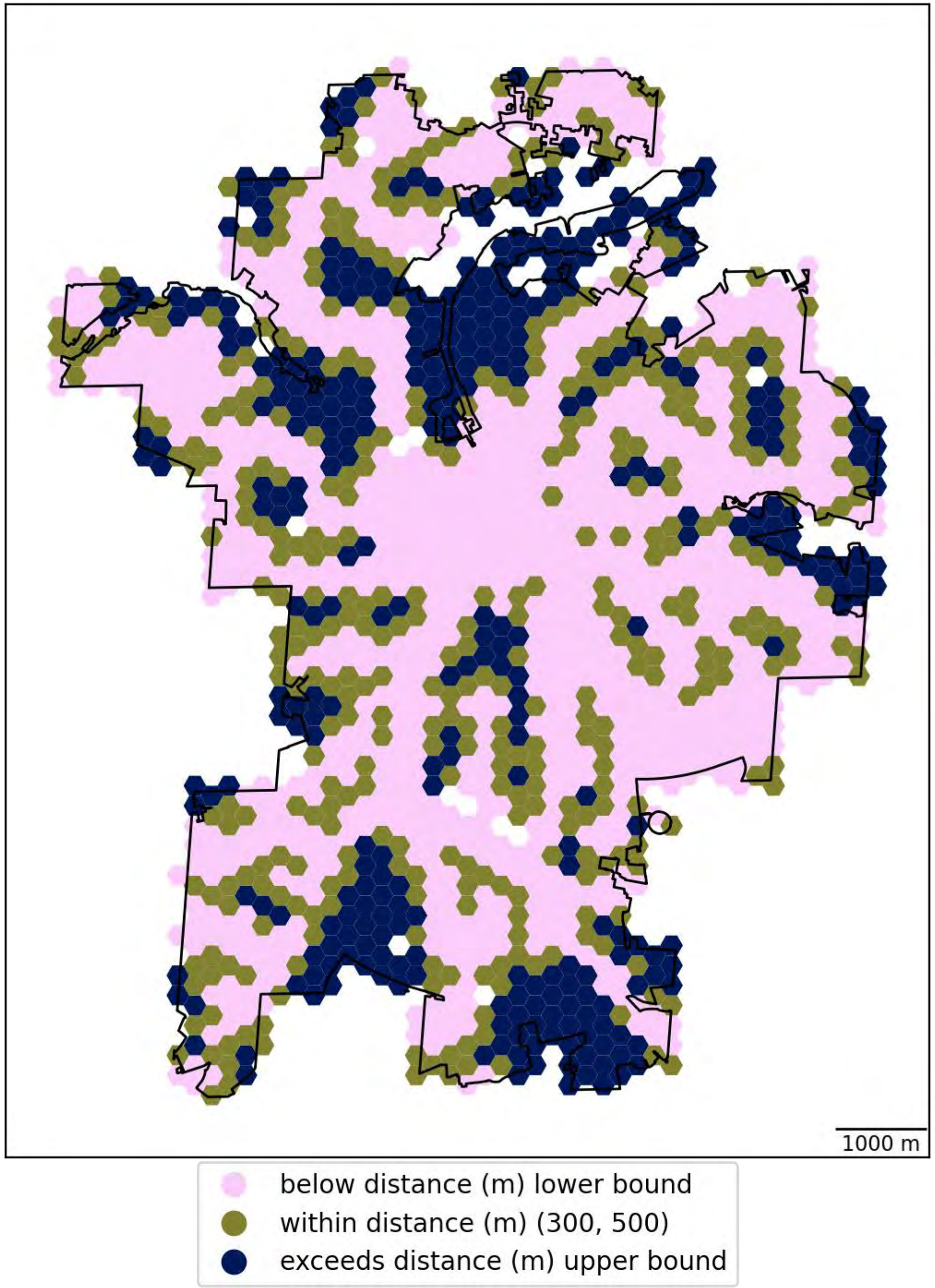

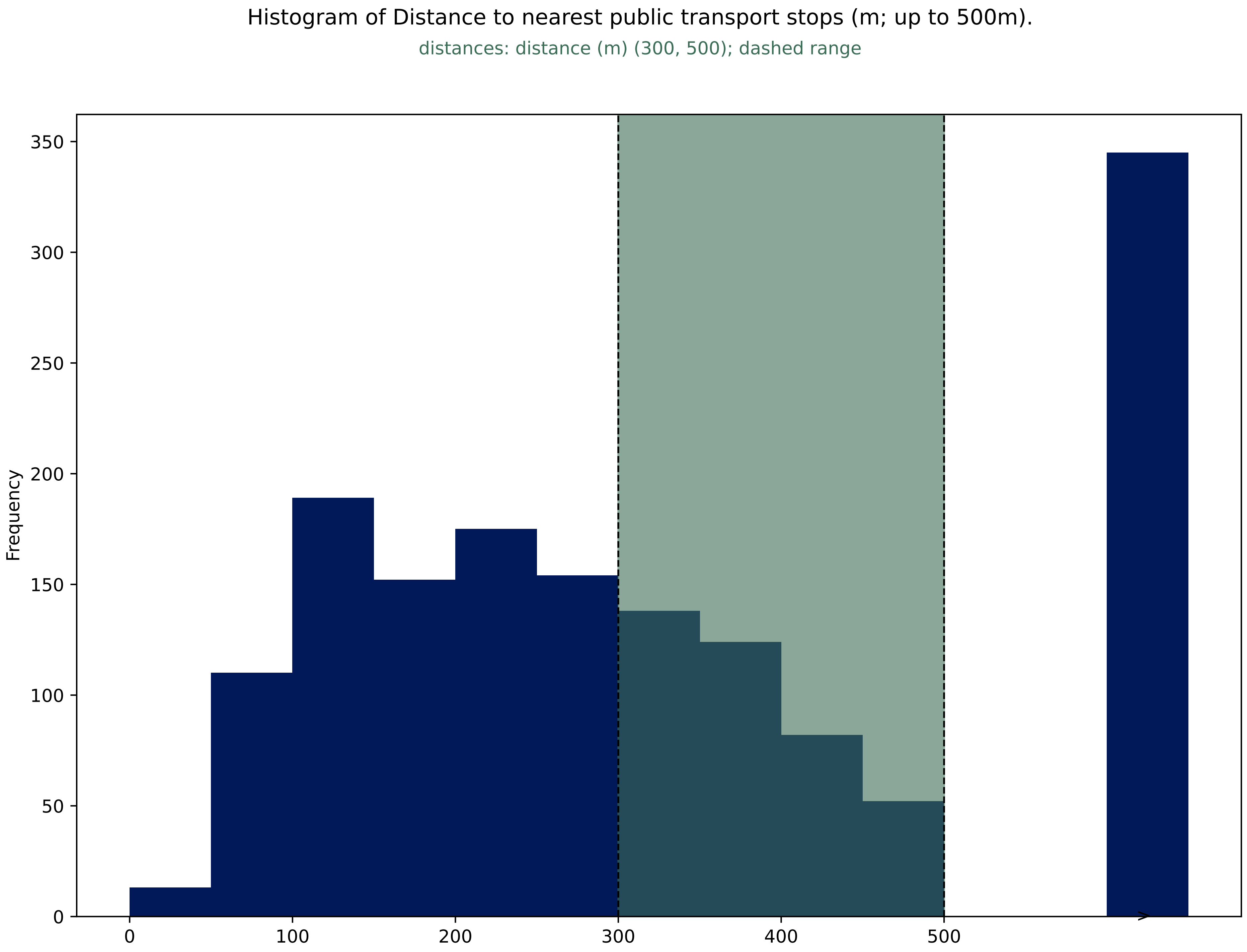

Histogram of Distance to nearest public transport stops (m; up to 500m).
distances: distance (m) (300, 500); dashed range
Frequency
0
50
100
150
200
250
300
350
0
100
200
300
400
500
>

Distance to nearest park (m; up to 500m)

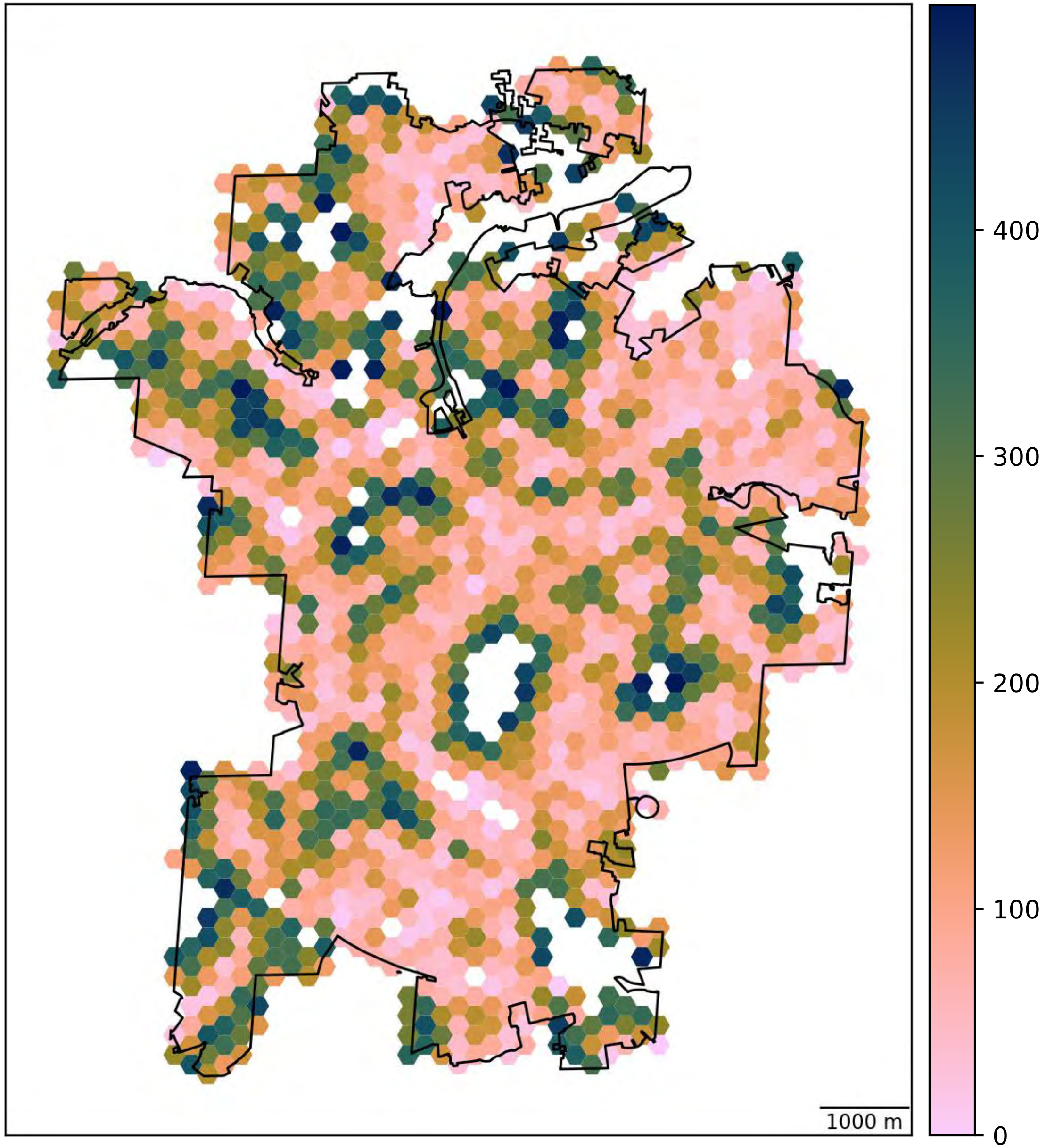

distances: Estimated Distance to nearest park (m; up to 500m) requirement for distances to destinations, measured up to a maximum distance target threshold of 500 metres

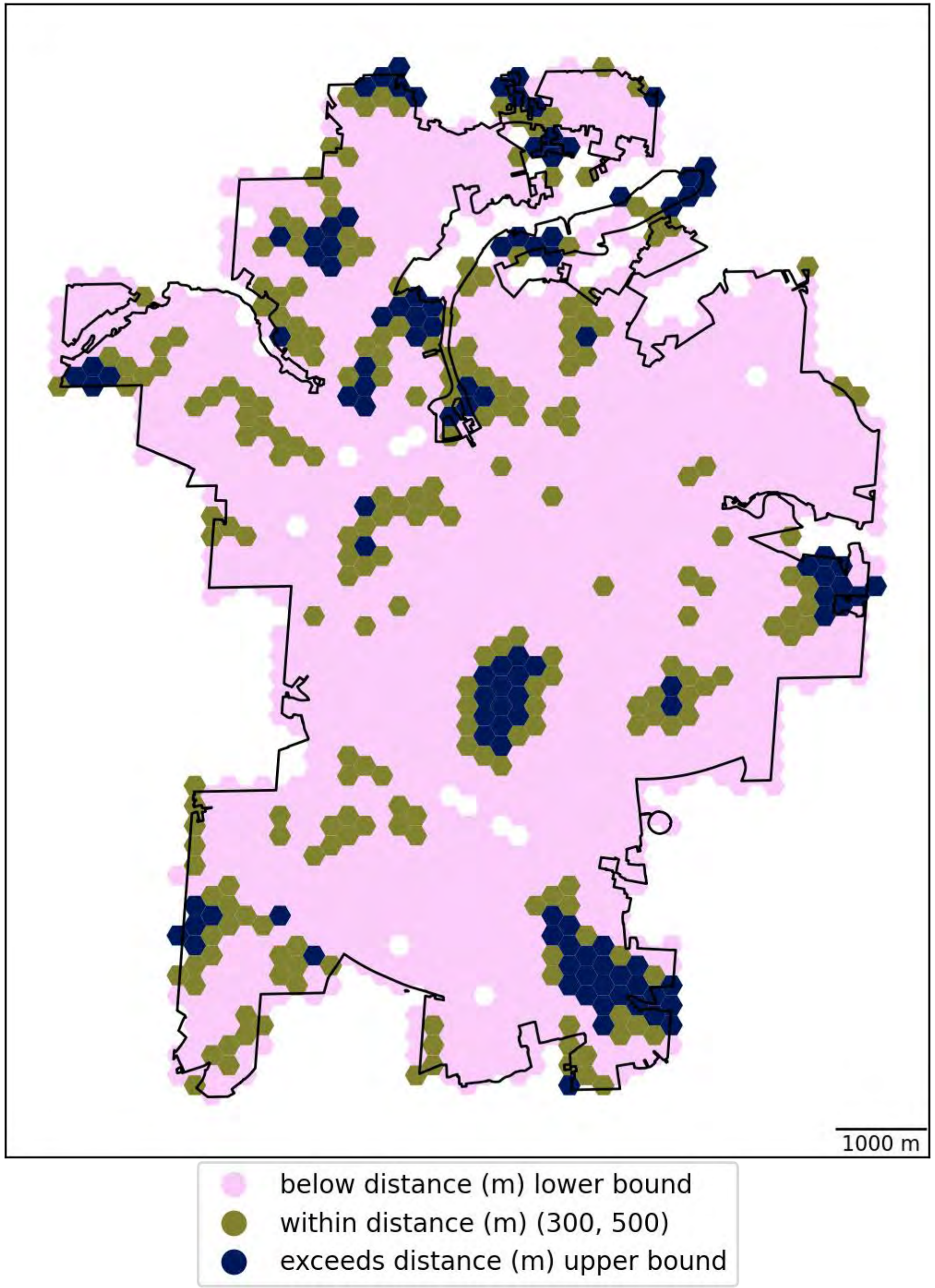

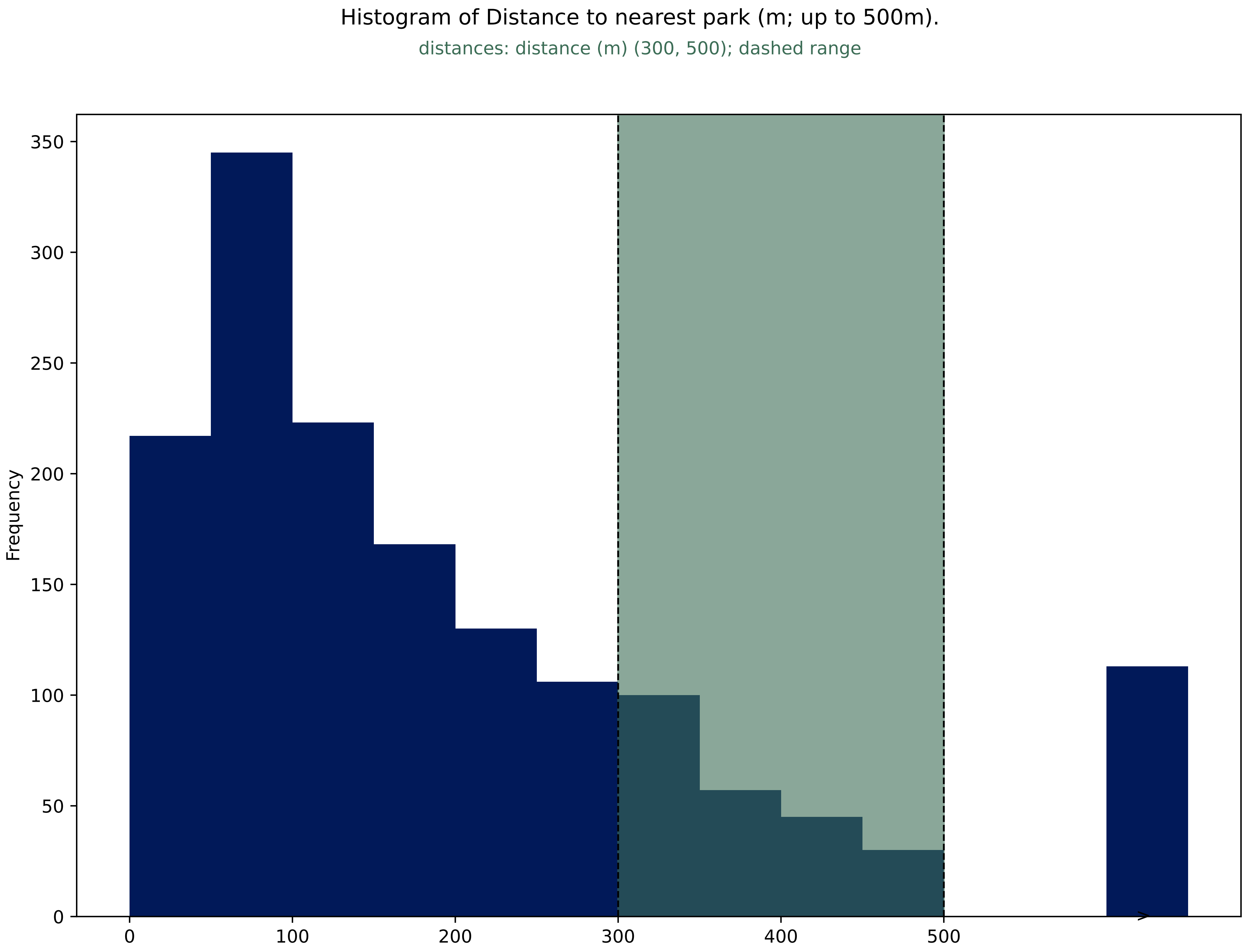

Histogram of Distance to nearest park (m; up to 500m).
distances: distance (m) (300, 500); dashed range
Frequency
0
50
100
150
200
250
300
350
0
100
200
300
400
500
>

Europe, Germany, Cologne

Satellite imagery of urban study region (Bing)

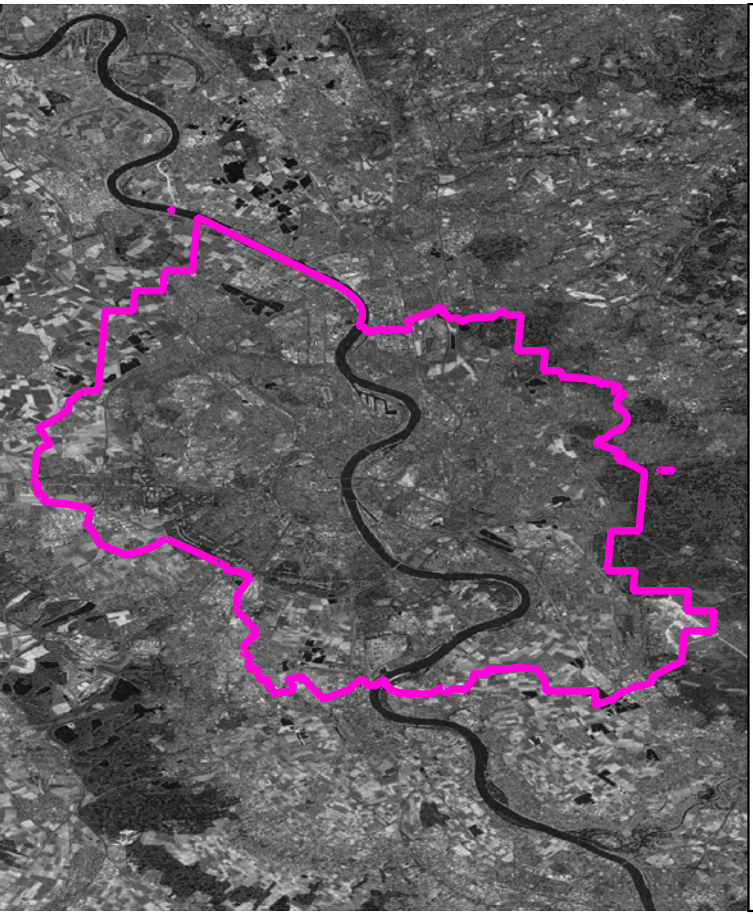

Walkability, relative to city

Walkability, relative to 25 global cities

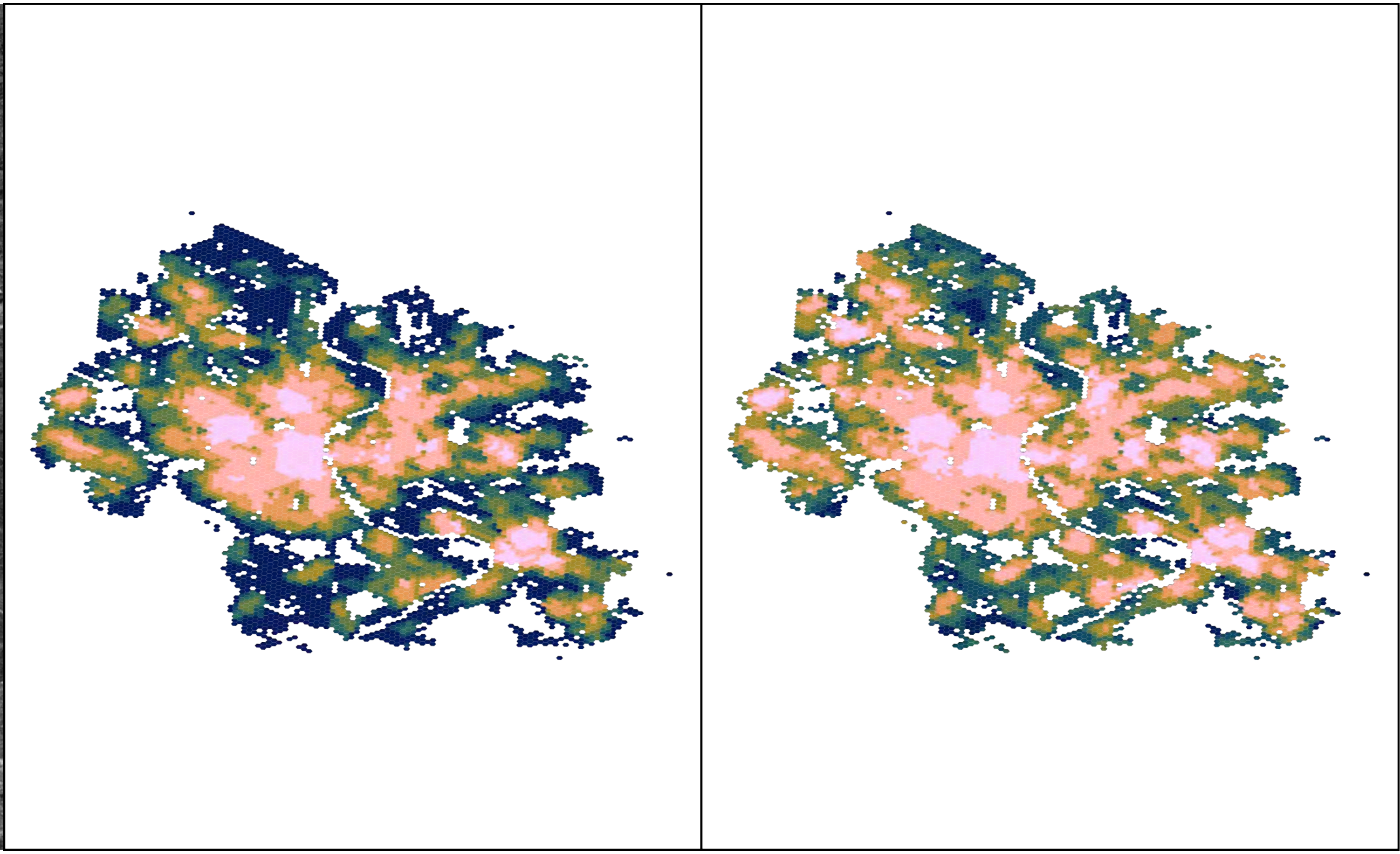

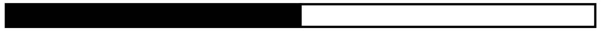

Walkability score

- <-3
- -3 to -2
- -2 to -1
- -1 to 0
- 0 to 1
- 1 to 2
- 2 to 3
- ≥3

Walkability relative to all cities by component variables (2D histograms), and overall (histogram)

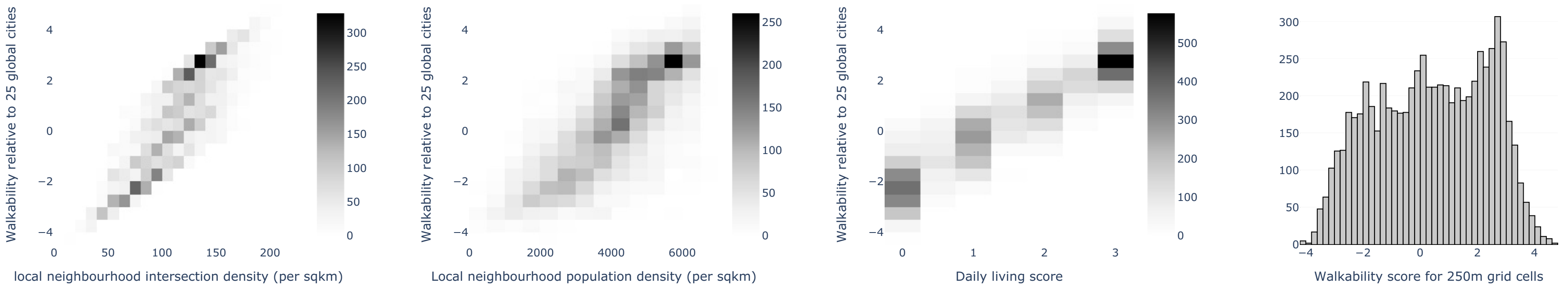

Mean 1000 m neighbourhood population per km²

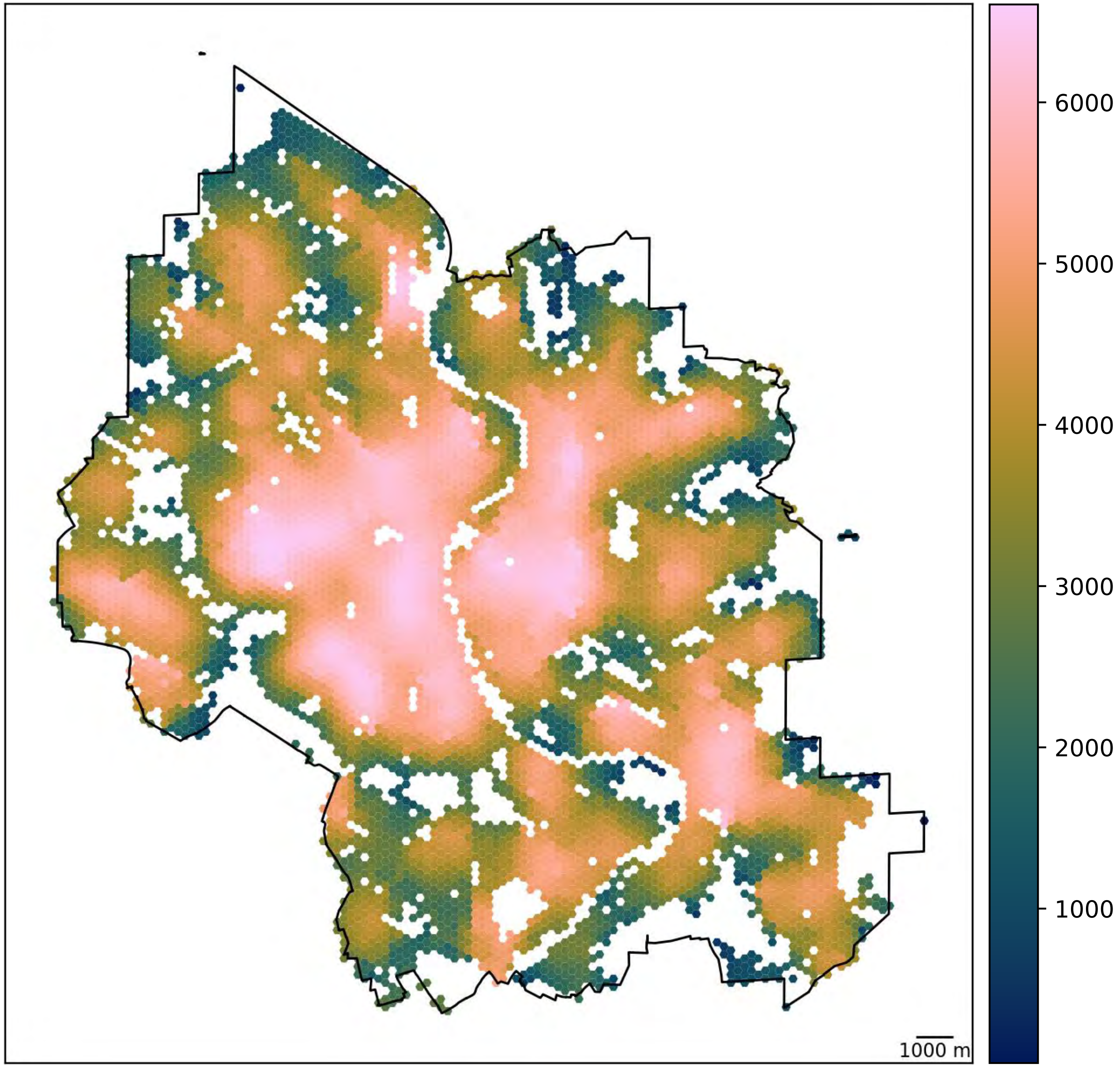

A: Estimated Mean 1000 m neighbourhood population per km² requirement for ≥80% probability of engaging in walking for transport

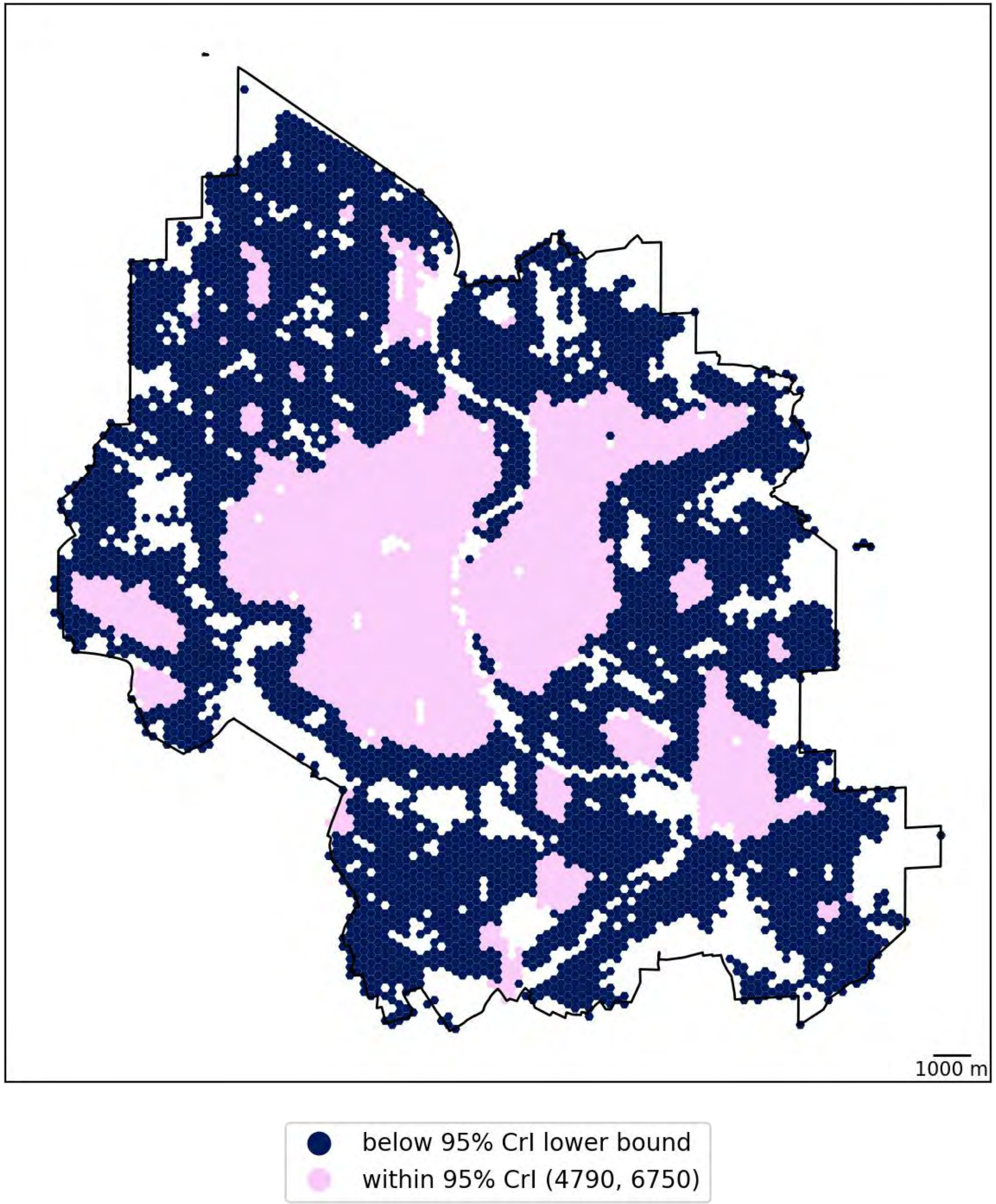

B: Estimated Mean 1000 m neighbourhood population per km² requirement for reaching the WHO's target of a ≥15% relative reduction in insufficient physical activity through walking

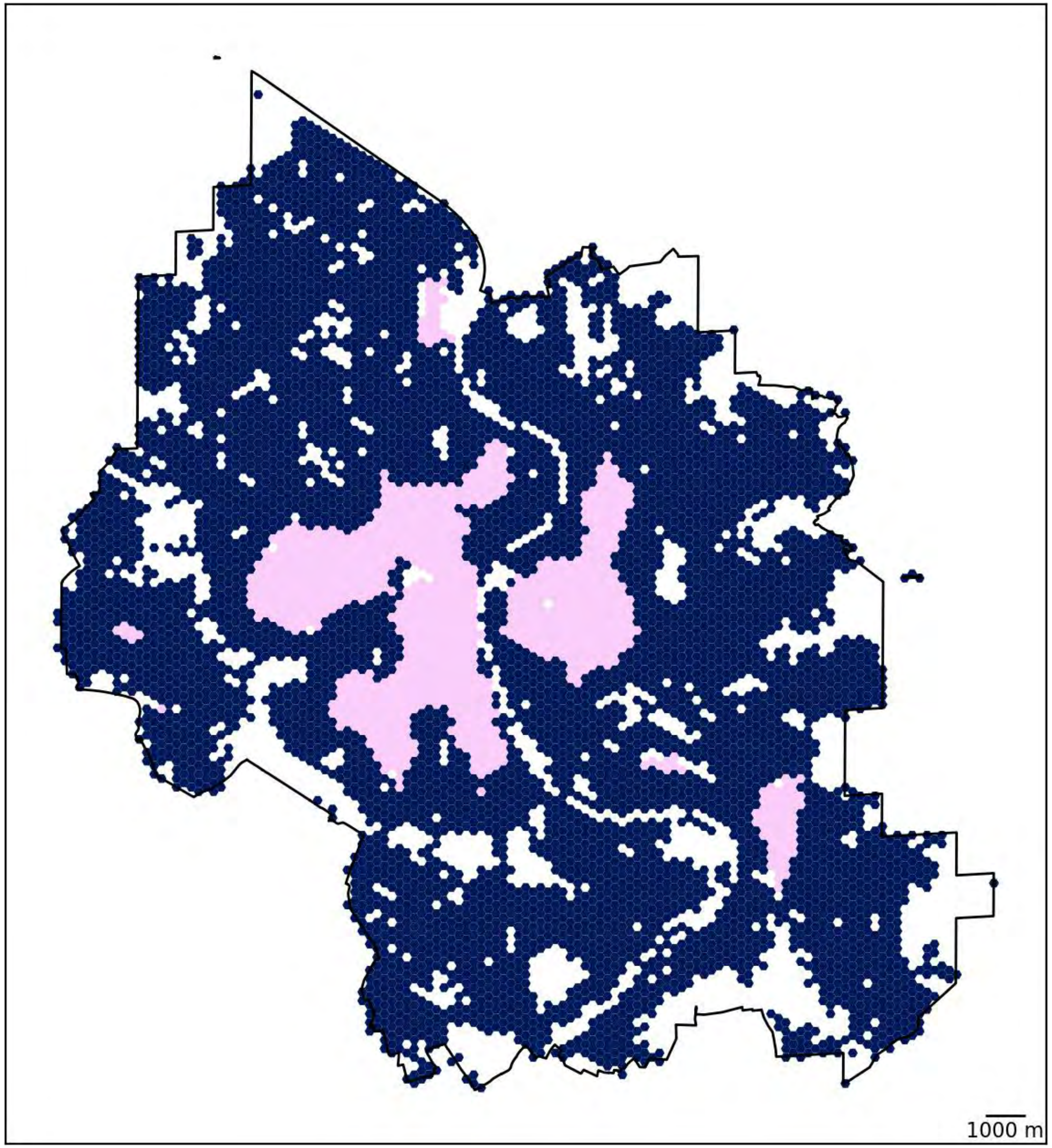

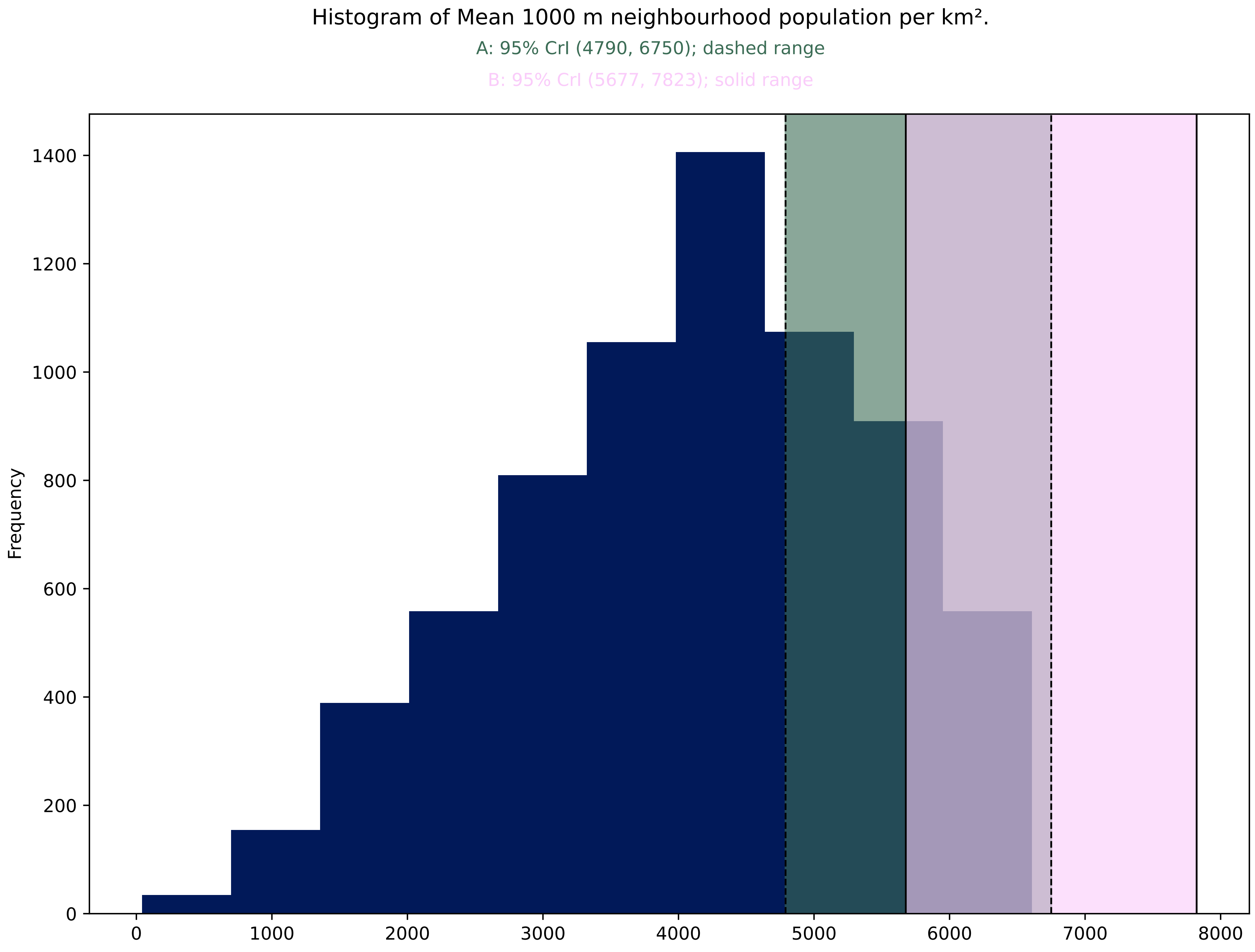

Histogram of Mean 1000 m neighbourhood population per km².
A: 95% CrI (4790, 6750); dashed range
B: 95% CrI (5677, 7823); solid range
Frequency
0
200
400
600
800
1000
1200
1400
0
1000
2000
3000
4000
5000
6000
7000
8000

Mean 1000 m neighbourhood street intersections per km²

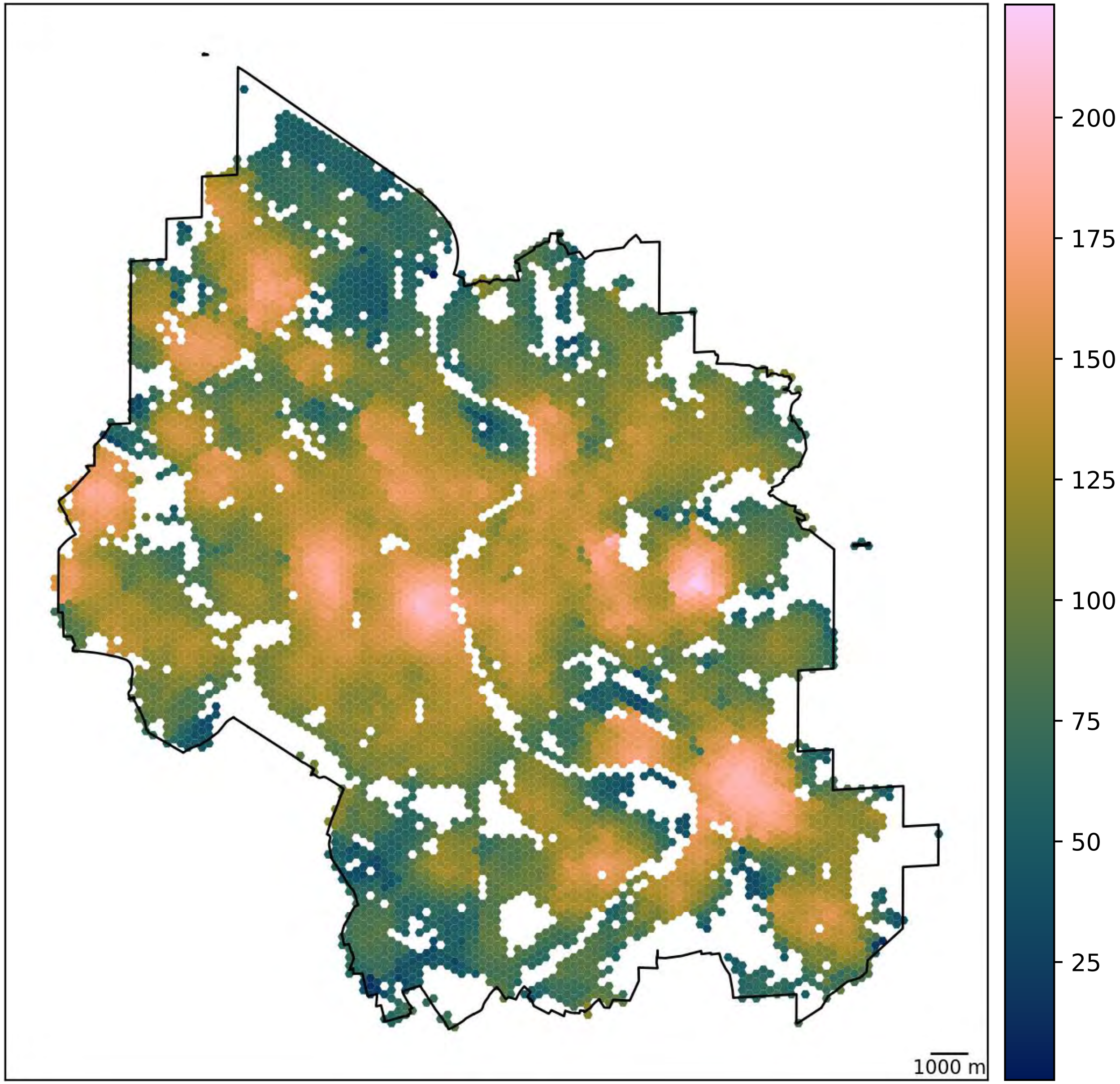

A: Estimated Mean 1000 m neighbourhood street intersections per km² requirement for ≥80% probability of engaging in walking for transport

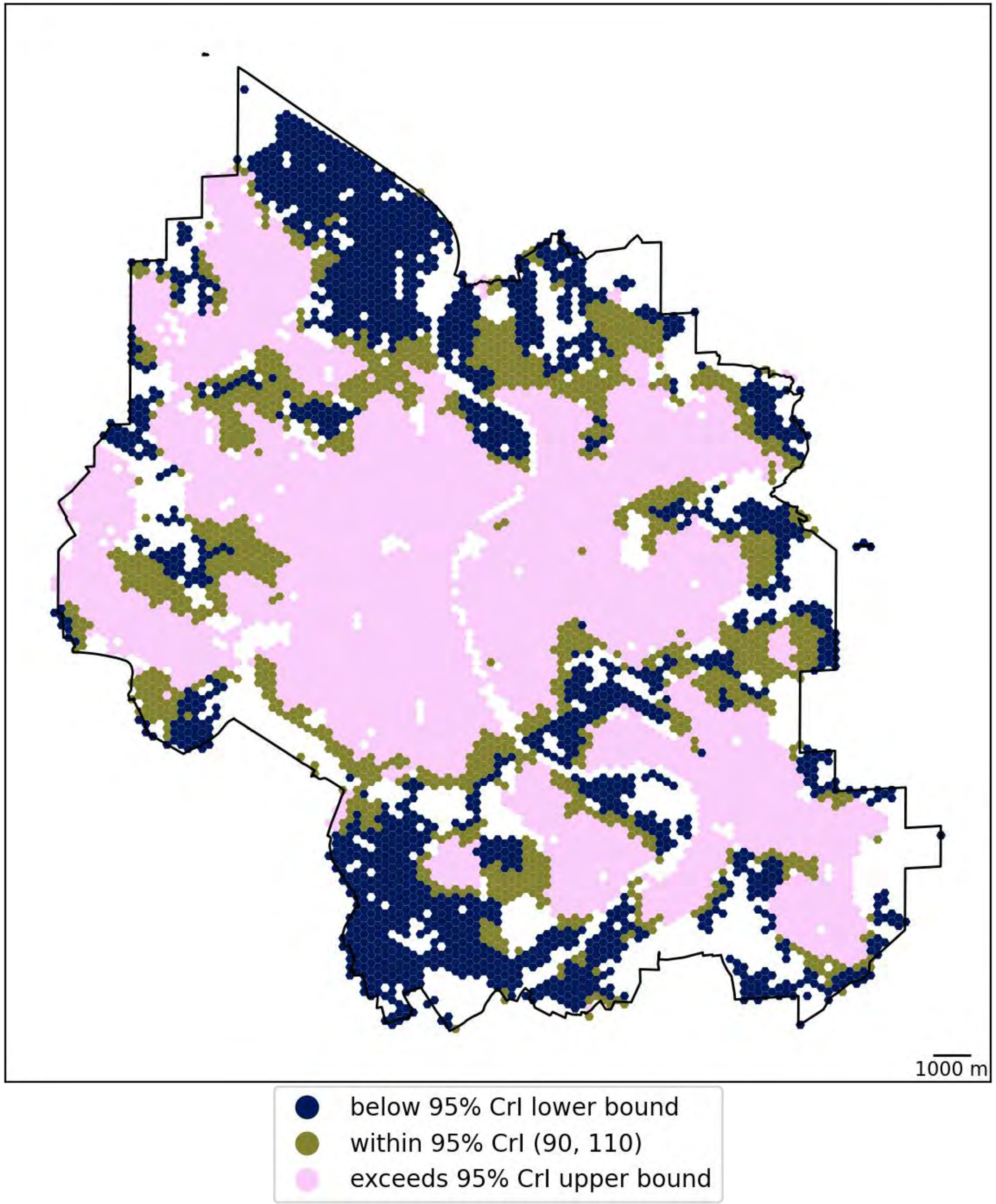

B: Estimated Mean 1000 m neighbourhood street intersections per km² requirement for reaching the WHO's target of a ≥15% relative reduction in insufficient physical activity through walking

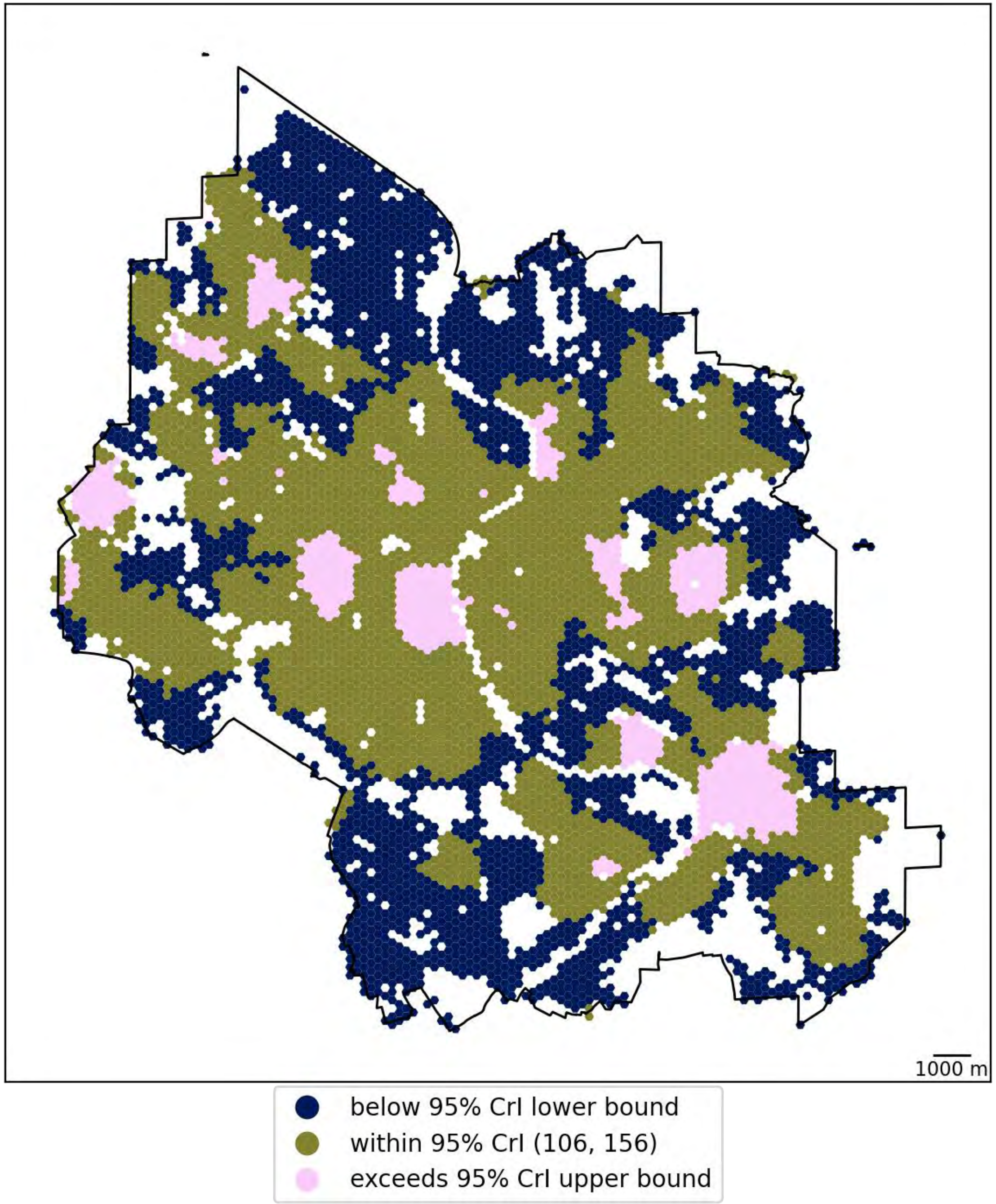

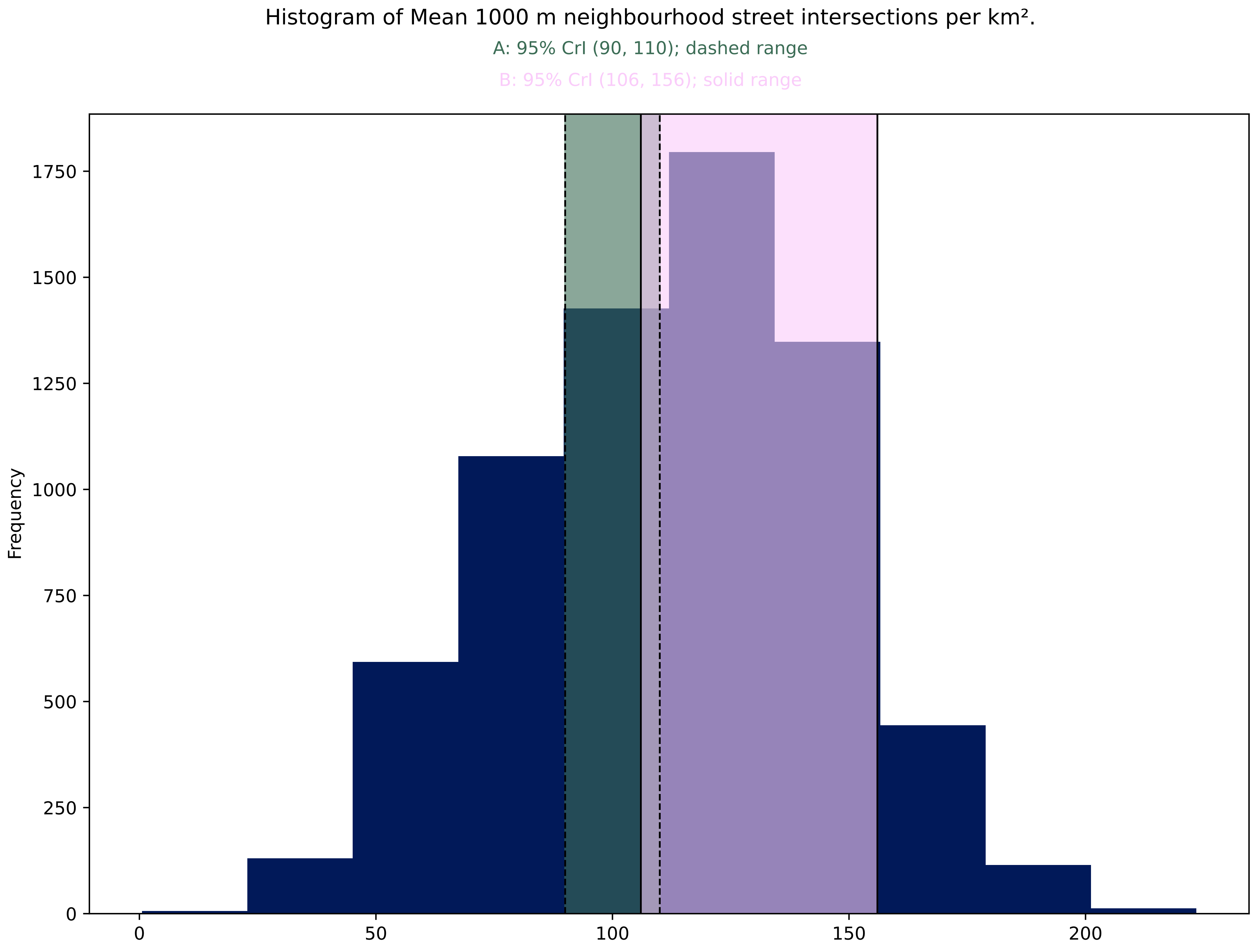

Histogram of Mean 1000 m neighbourhood street intersections per km².
A: 95% CrI (90, 110); dashed range
B: 95% CrI (106, 156); solid range
Frequency
0
250
500
750
1000
1250
1500
1750
0
50
100
150
200

Distance to nearest public transport stops (m; up to 500m)

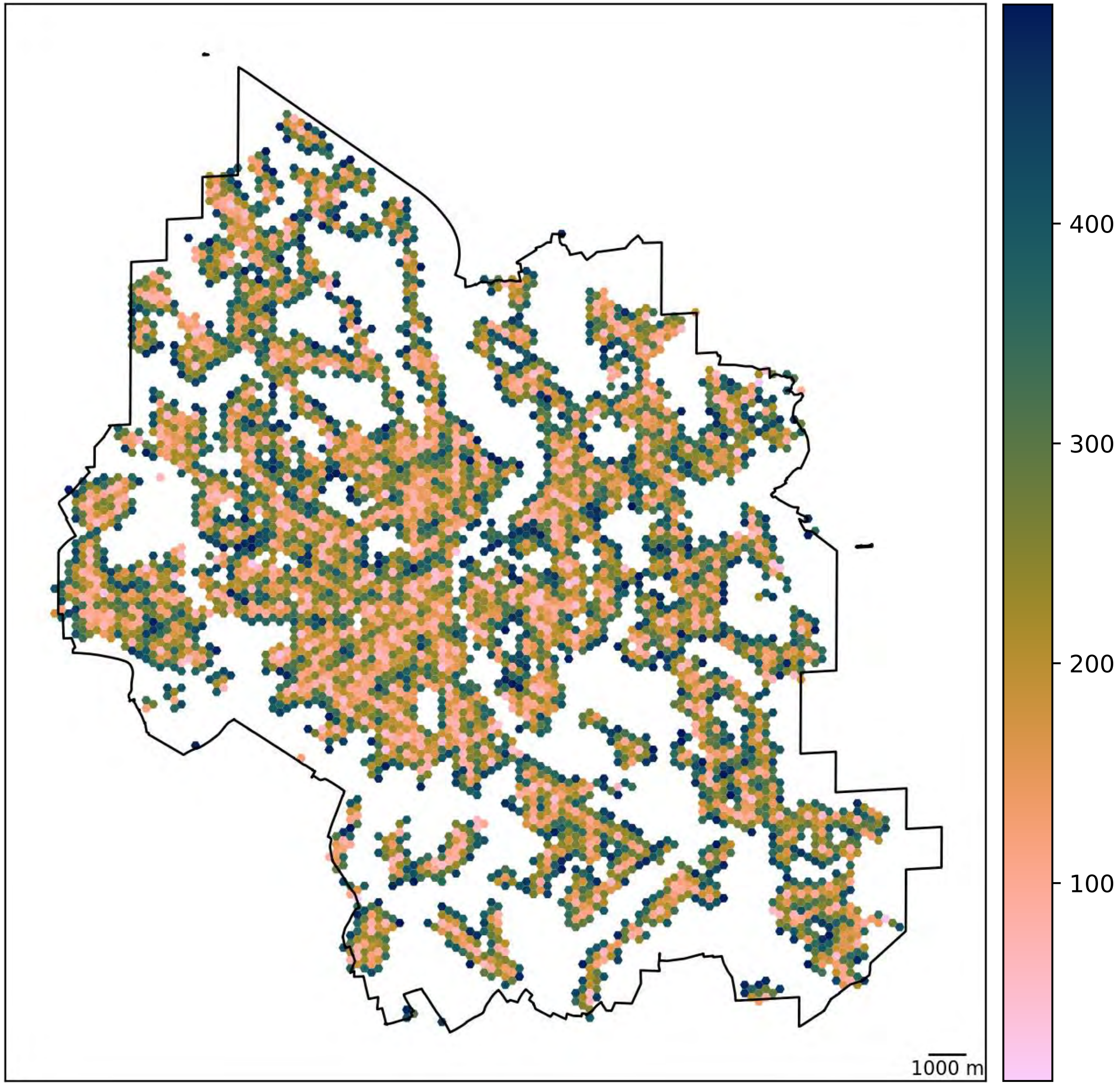

distances: Estimated Distance to nearest public transport stops (m; up to 500m) requirement for distances to destinations, measured up to a maximum distance target threshold of 500 metres

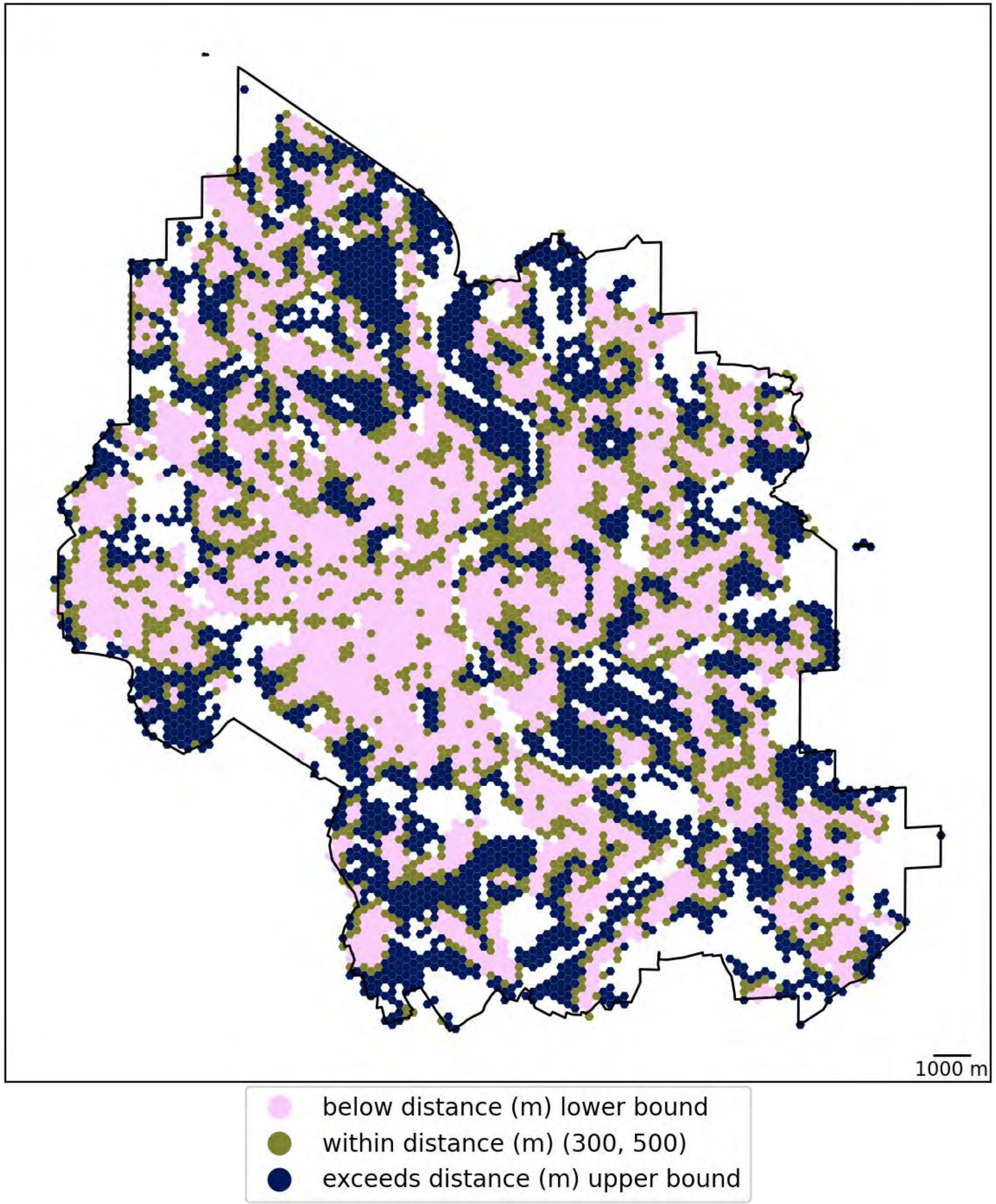

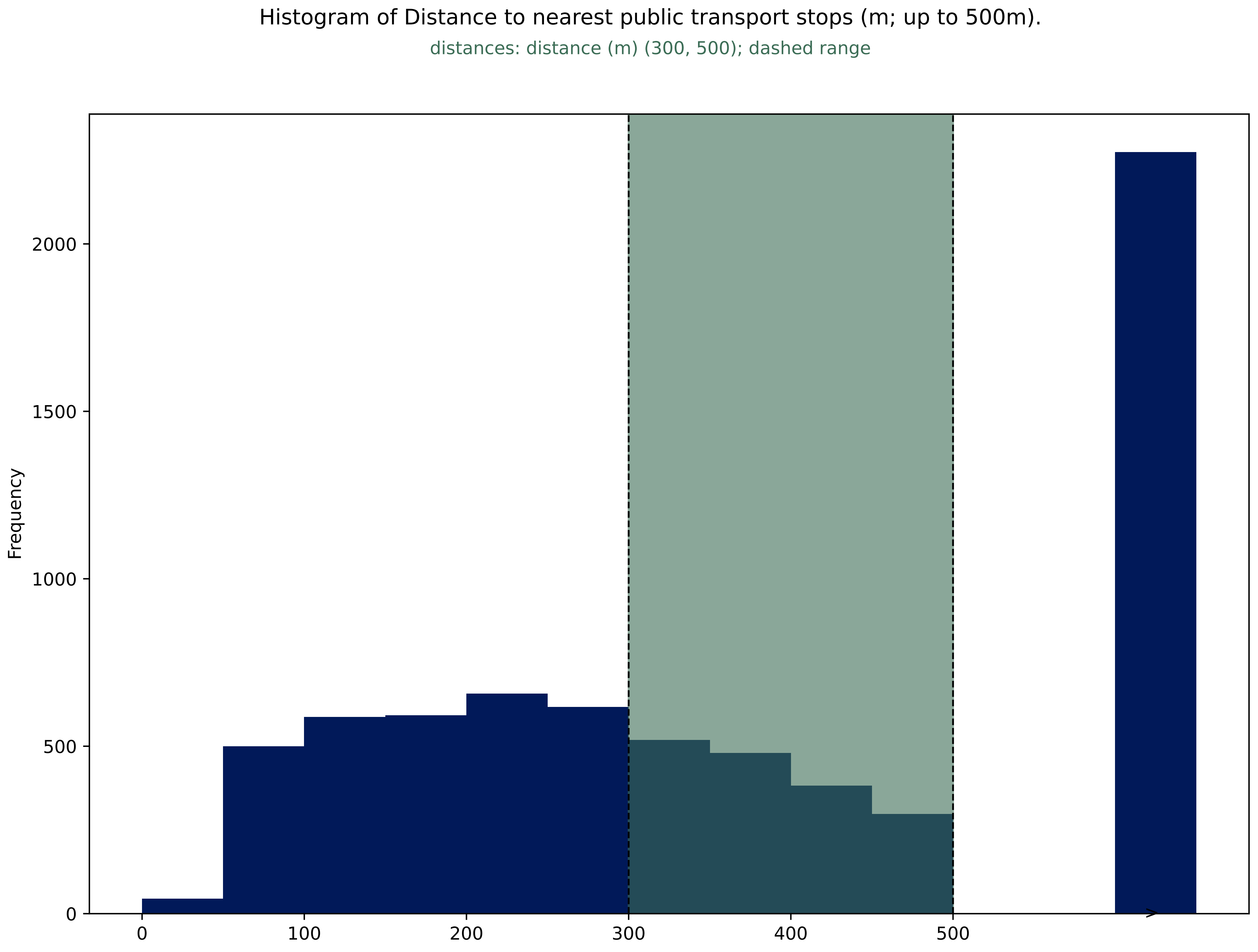
Histogram of Distance to nearest public transport stops (m; up to 500m).
distances: distance (m) (300, 500); dashed range
Frequency
0
500
1000
1500
2000
0
100
200
300
400
500
>

Distance to nearest park (m; up to 500m)

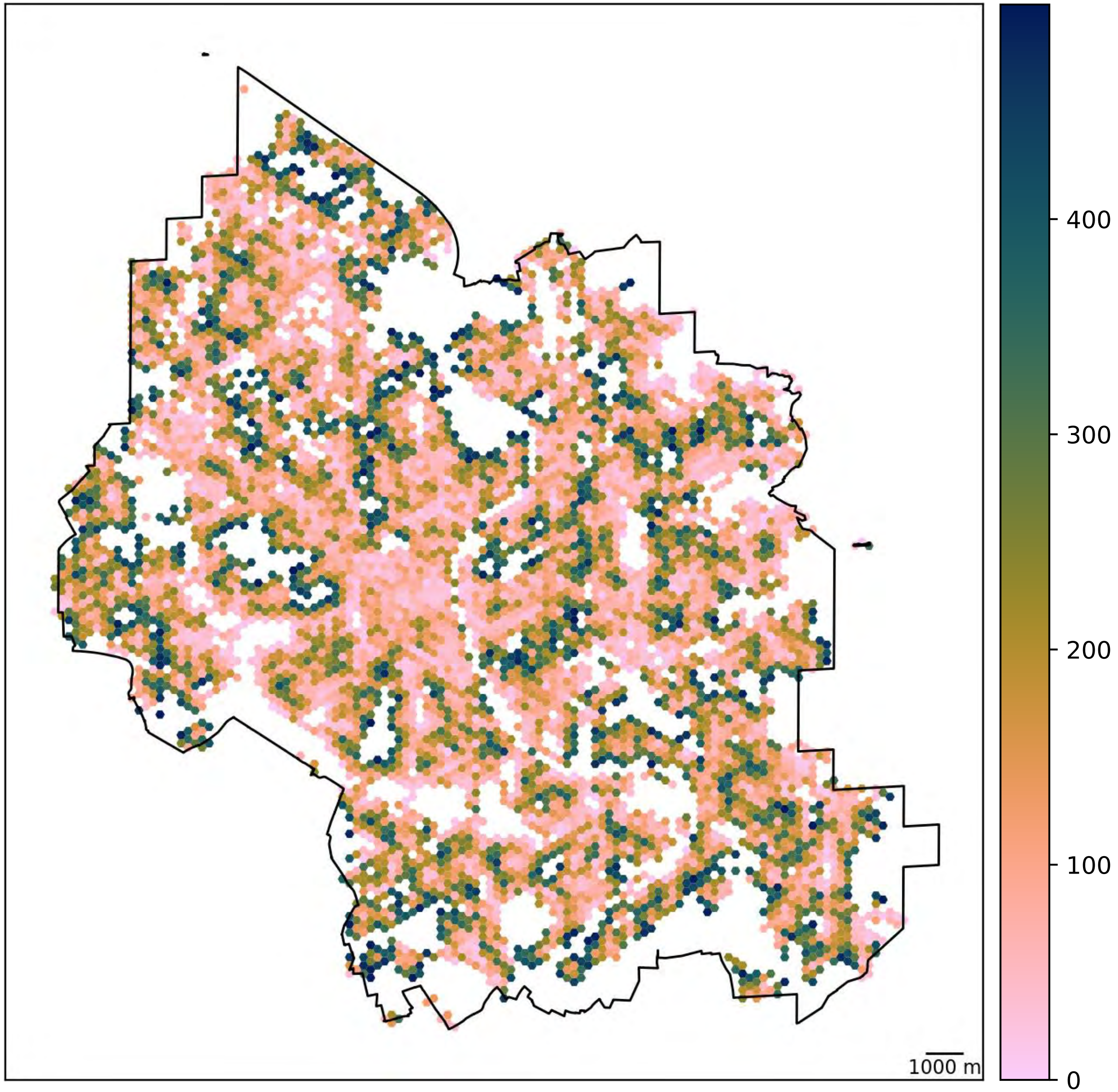

distances: Estimated Distance to nearest park (m; up to 500m) requirement for distances to destinations, measured up to a maximum distance target threshold of 500 metres

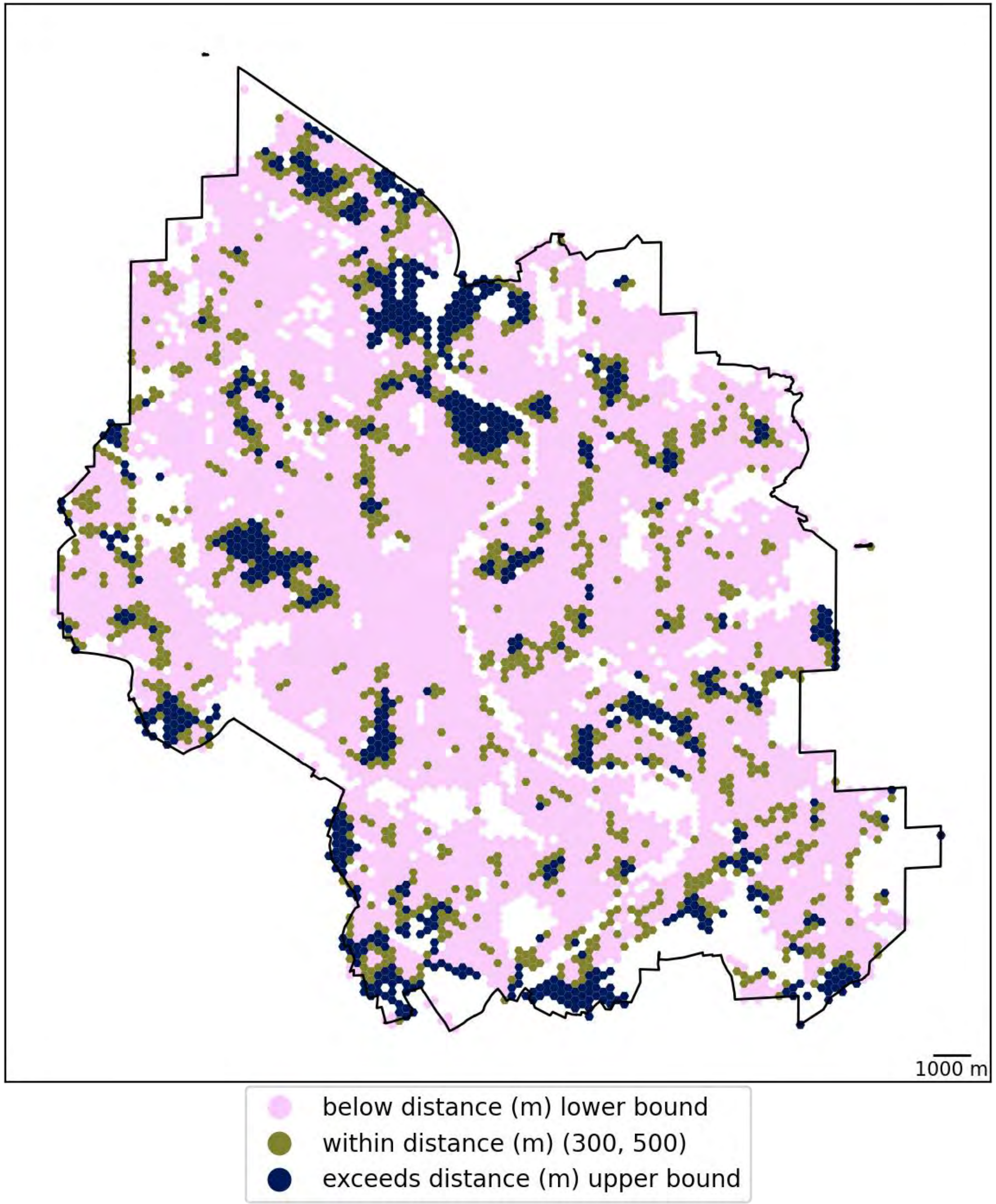

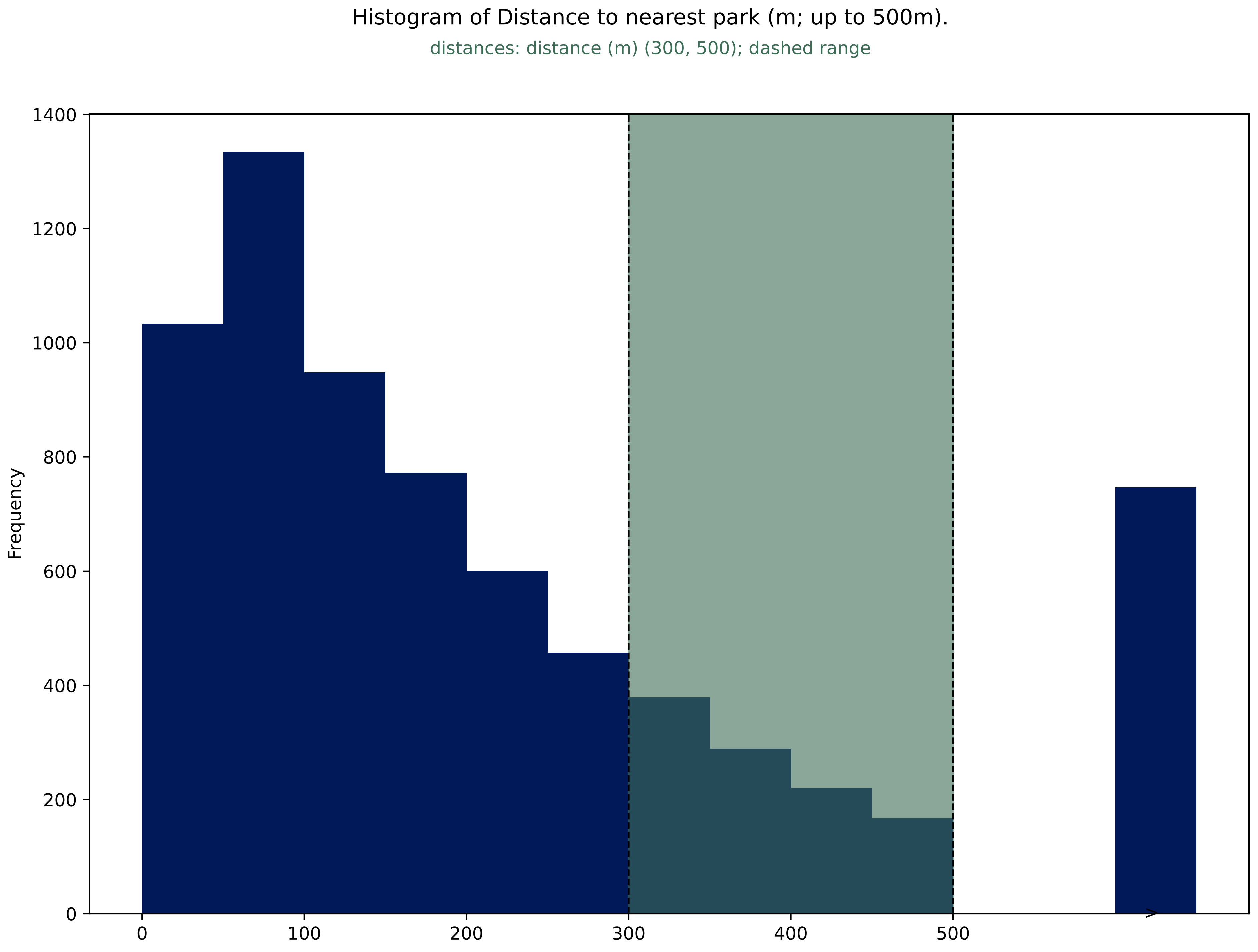
Histogram of Distance to nearest park (m; up to 500m).
distances: distance (m) (300, 500); dashed range
Frequency
0
200
400
600
800
1000
1200
1400
0
100
200
300
400
500

Europe, Portugal, Lisbon

Satellite imagery of urban study region (Bing)

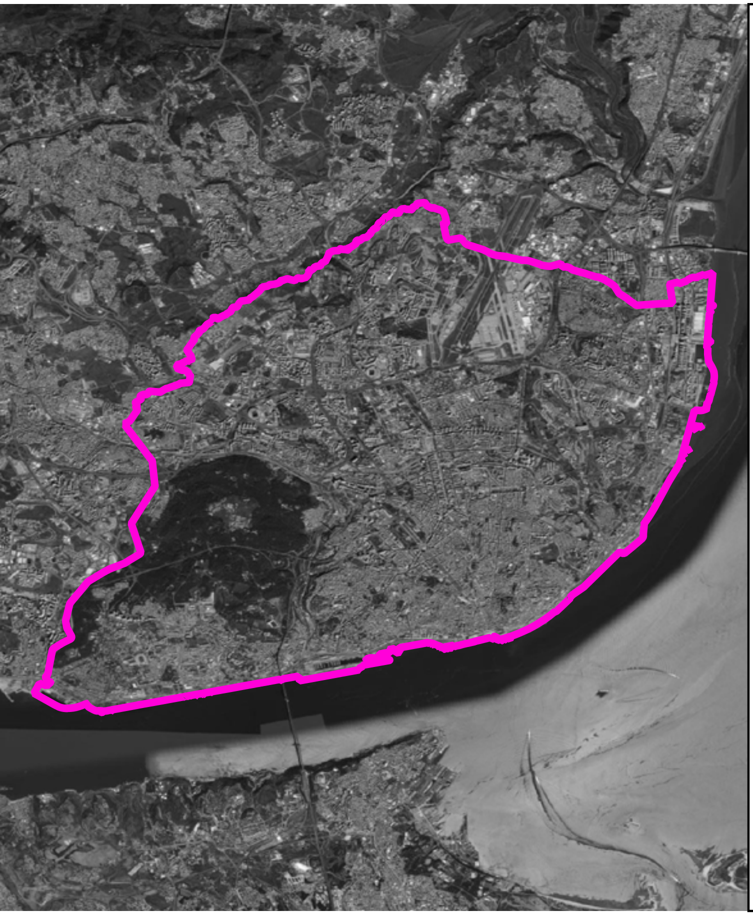

Walkability, relative to city

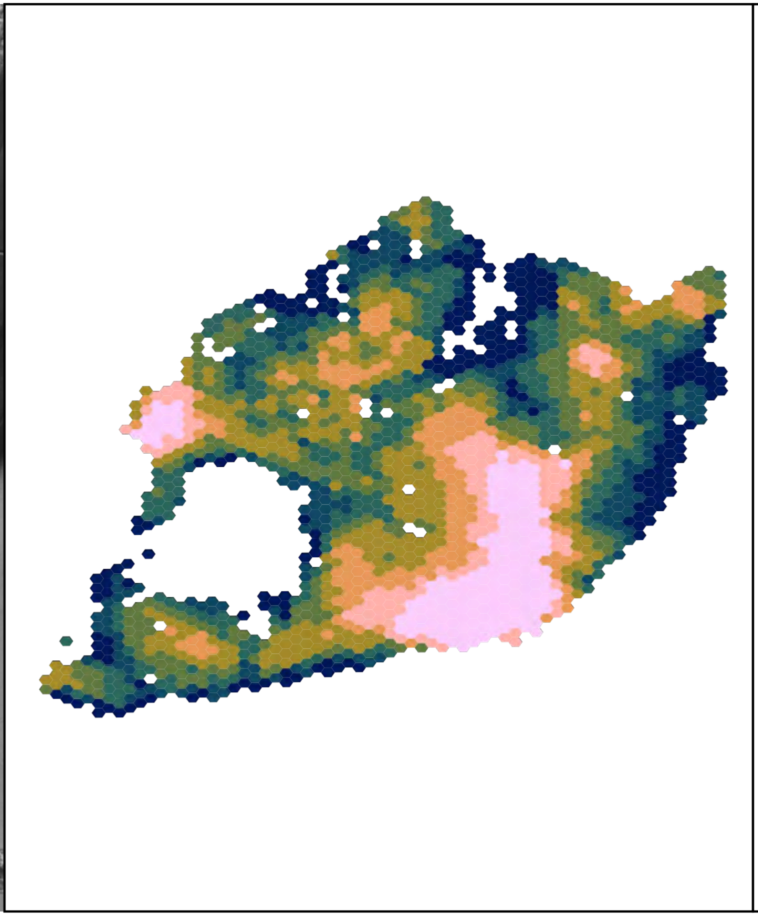

Walkability, relative to 25 global cities

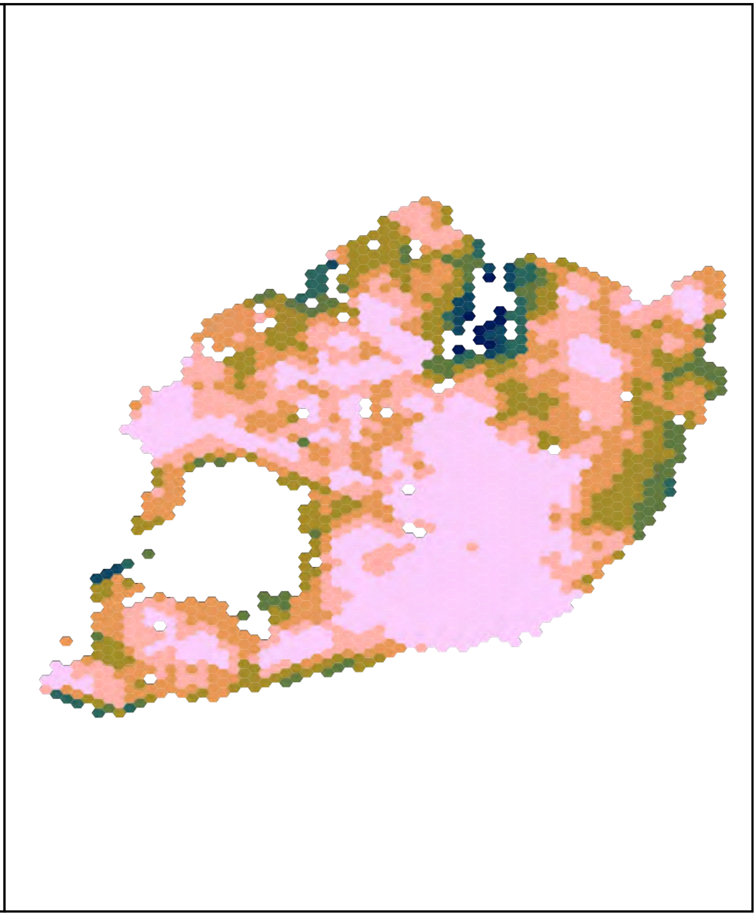

Urban boundary

0 6 12 km

Walkability score

- <-3
- -3 to -2
- -2 to -1
- -1 to 0
- 0 to 1
- 1 to 2
- 2 to 3
- ≥3

Walkability relative to all cities by component variables (2D histograms), and overall (histogram)

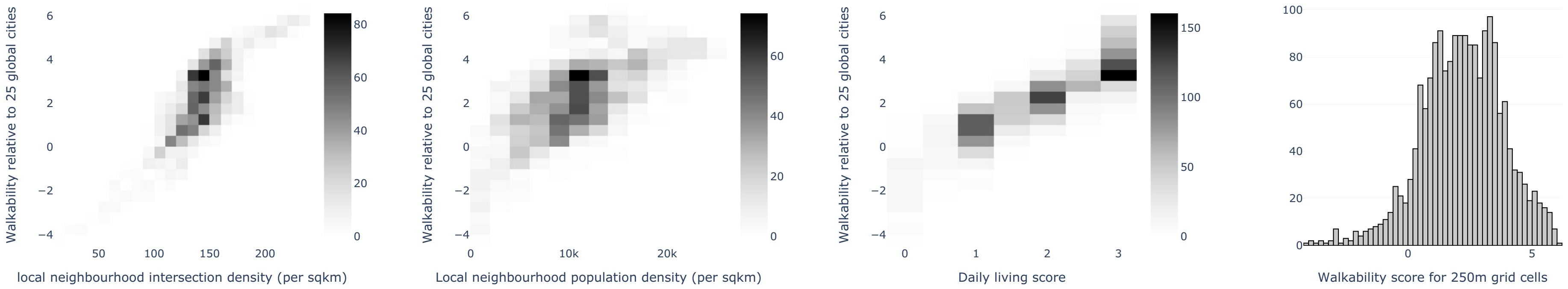

Mean 1000 m neighbourhood population per km²

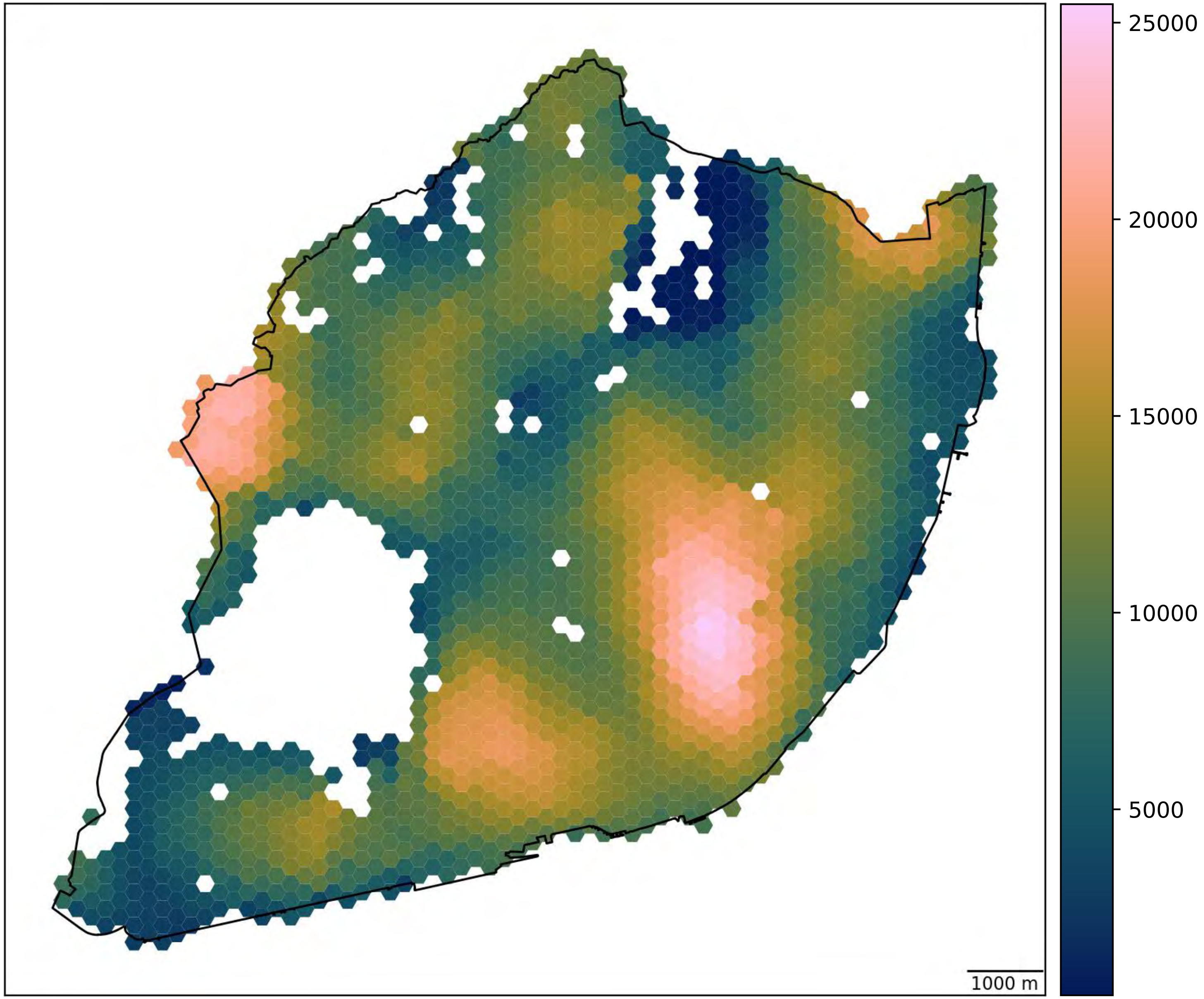

A: Estimated Mean 1000 m neighbourhood population per km² requirement for ≥80% probability of engaging in walking for transport

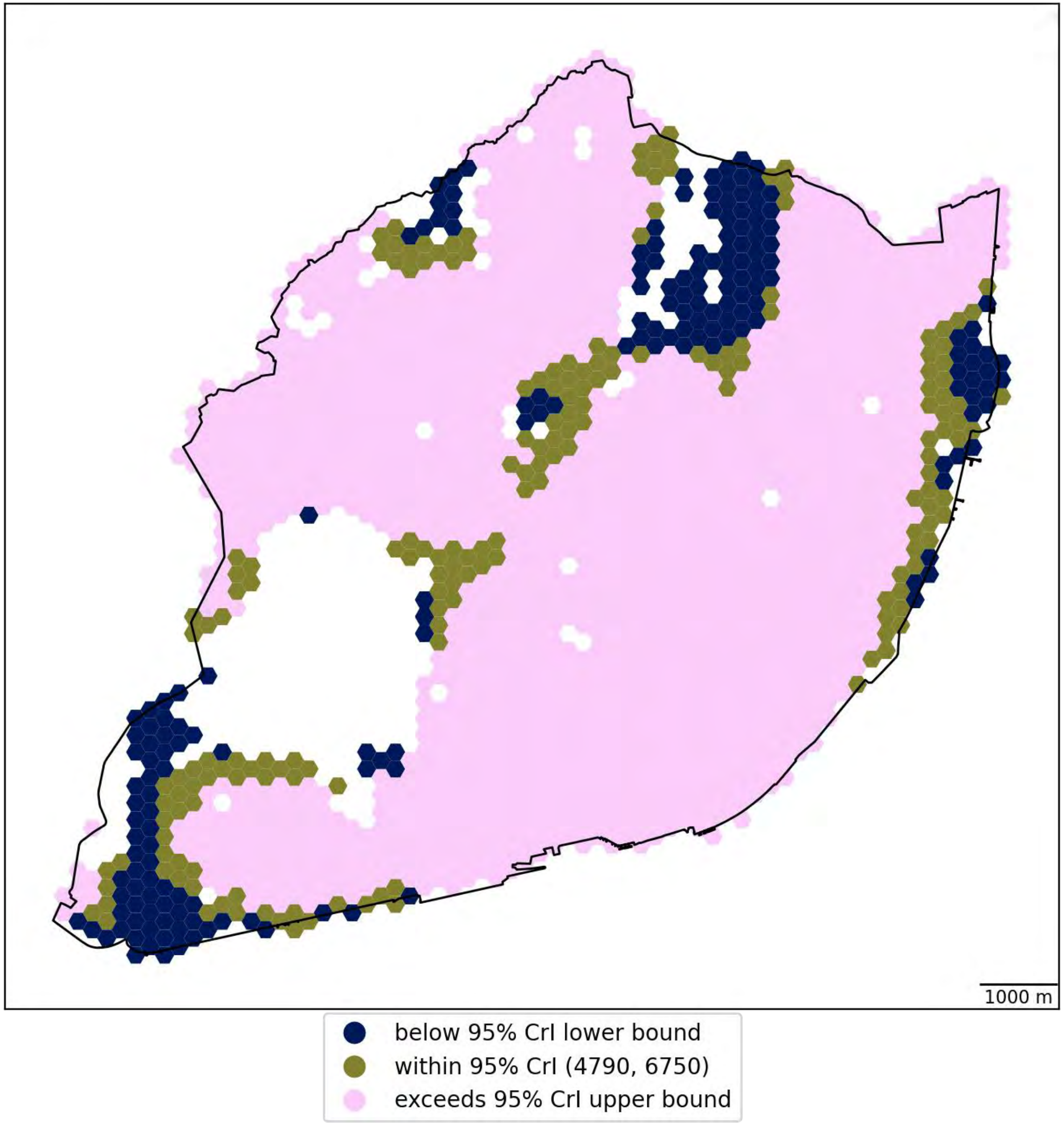

B: Estimated Mean 1000 m neighbourhood population per km² requirement for reaching the WHO's target of a ≥15% relative reduction in insufficient physical activity through walking

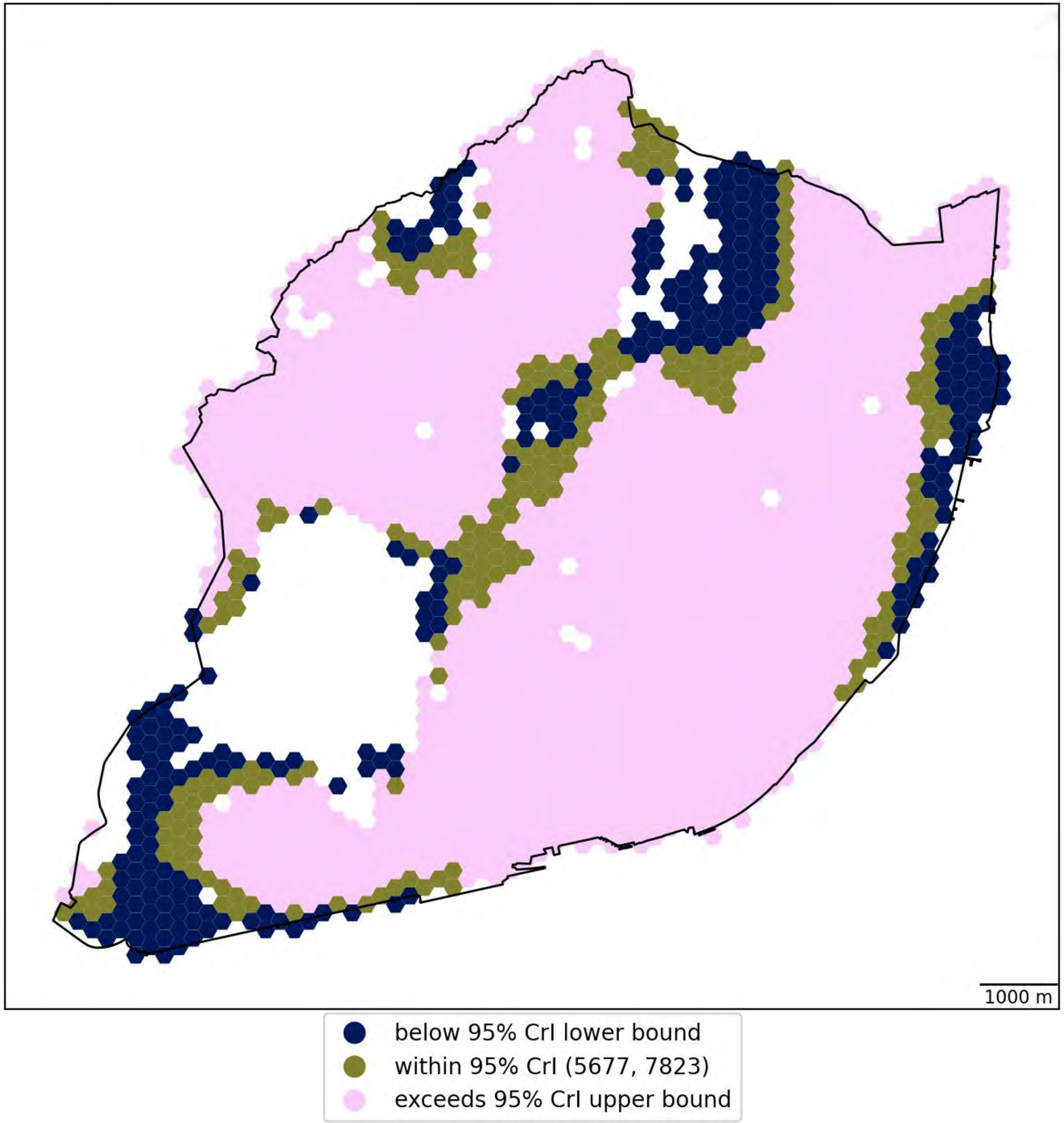

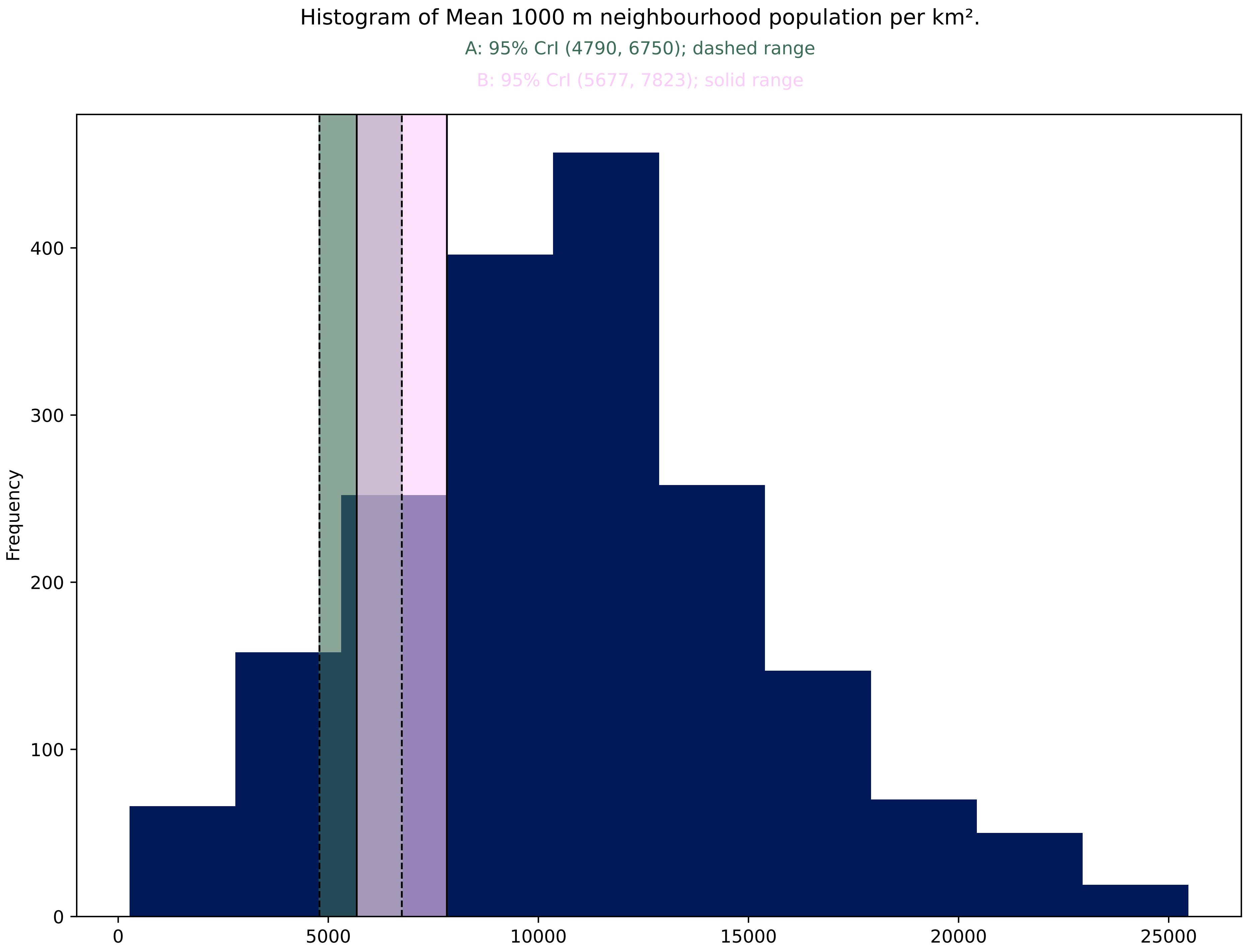
Histogram of Mean 1000 m neighbourhood population per km².
A: 95% CrI (4790, 6750); dashed range
B: 95% CrI (5677, 7823); solid range
Frequency
0
100
200
300
400
0
5000
10000
15000
20000
25000

Mean 1000 m neighbourhood street intersections per km²

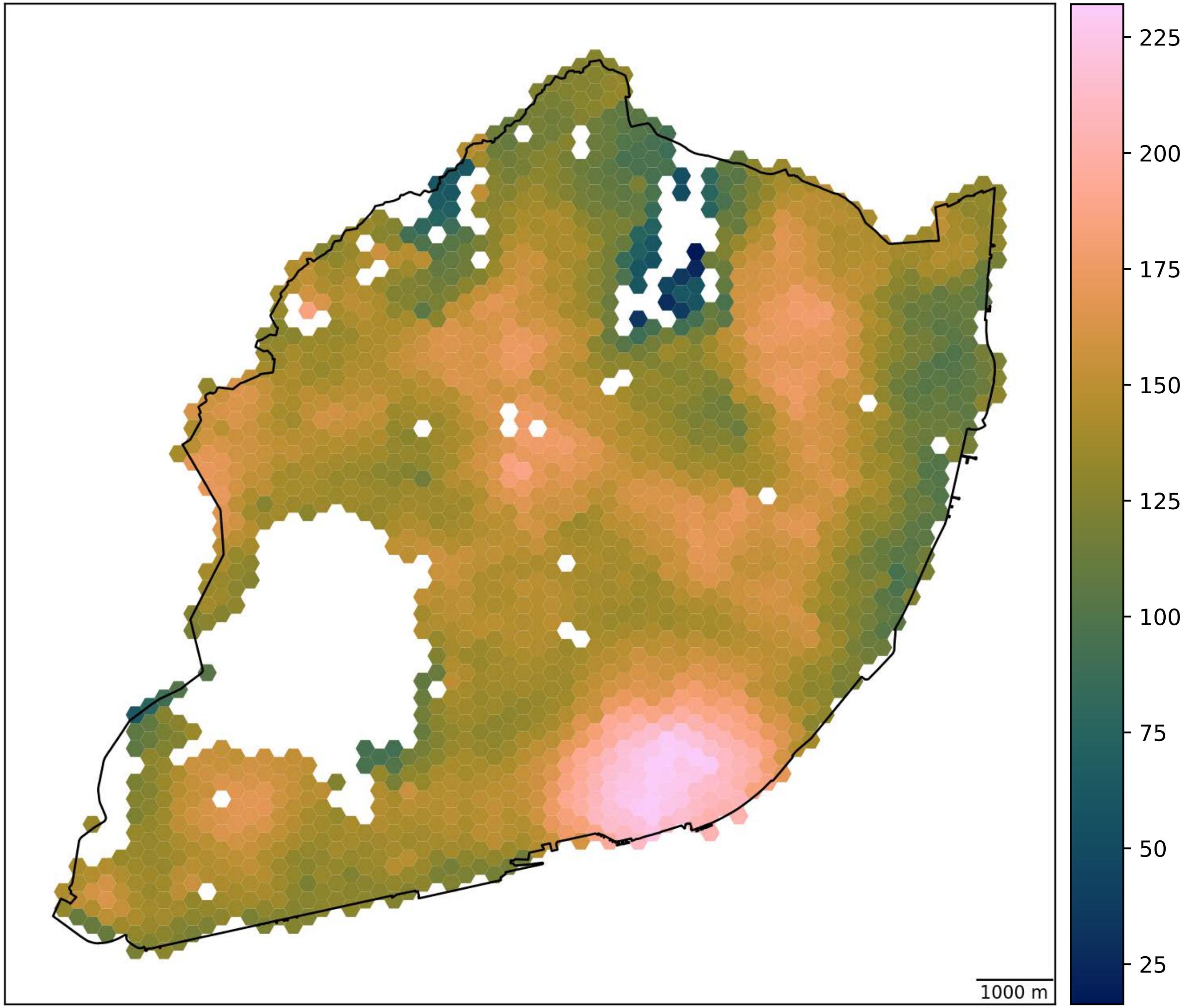

A: Estimated Mean 1000 m neighbourhood street intersections per km² requirement for ≥80% probability of engaging in walking for transport

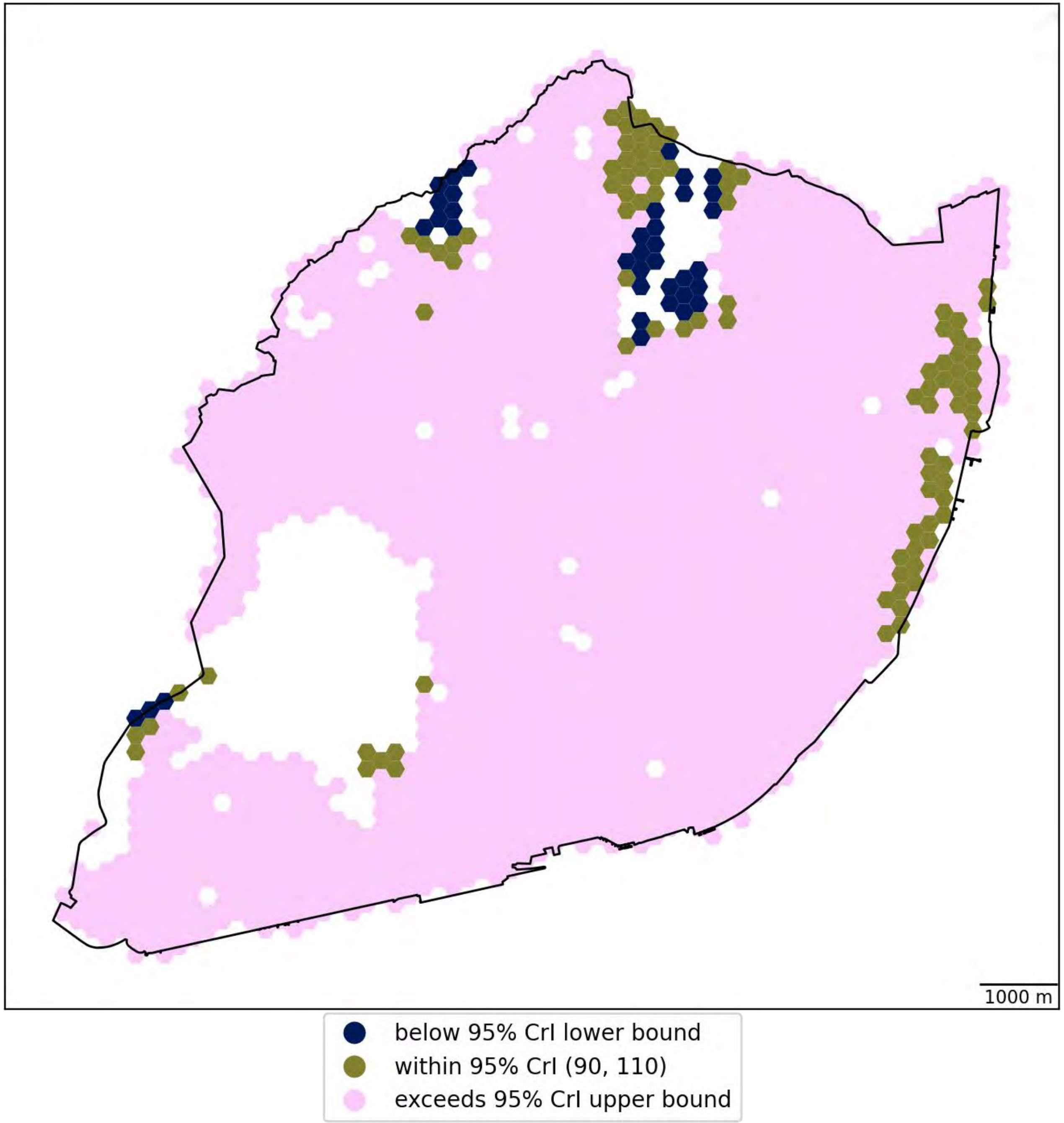

B: Estimated Mean 1000 m neighbourhood street intersections per km² requirement for reaching the WHO's target of a ≥15% relative reduction in insufficient physical activity through walking

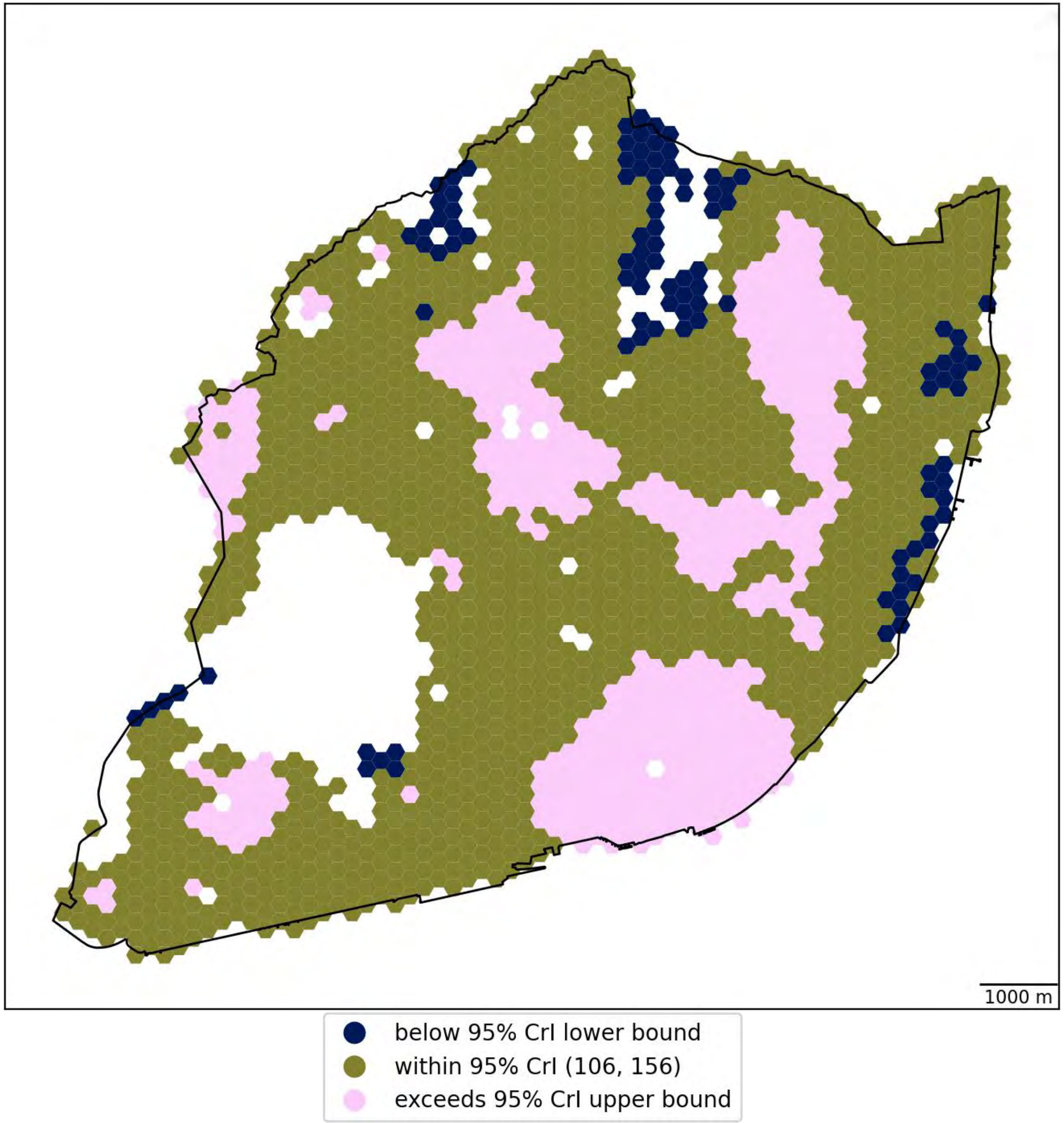

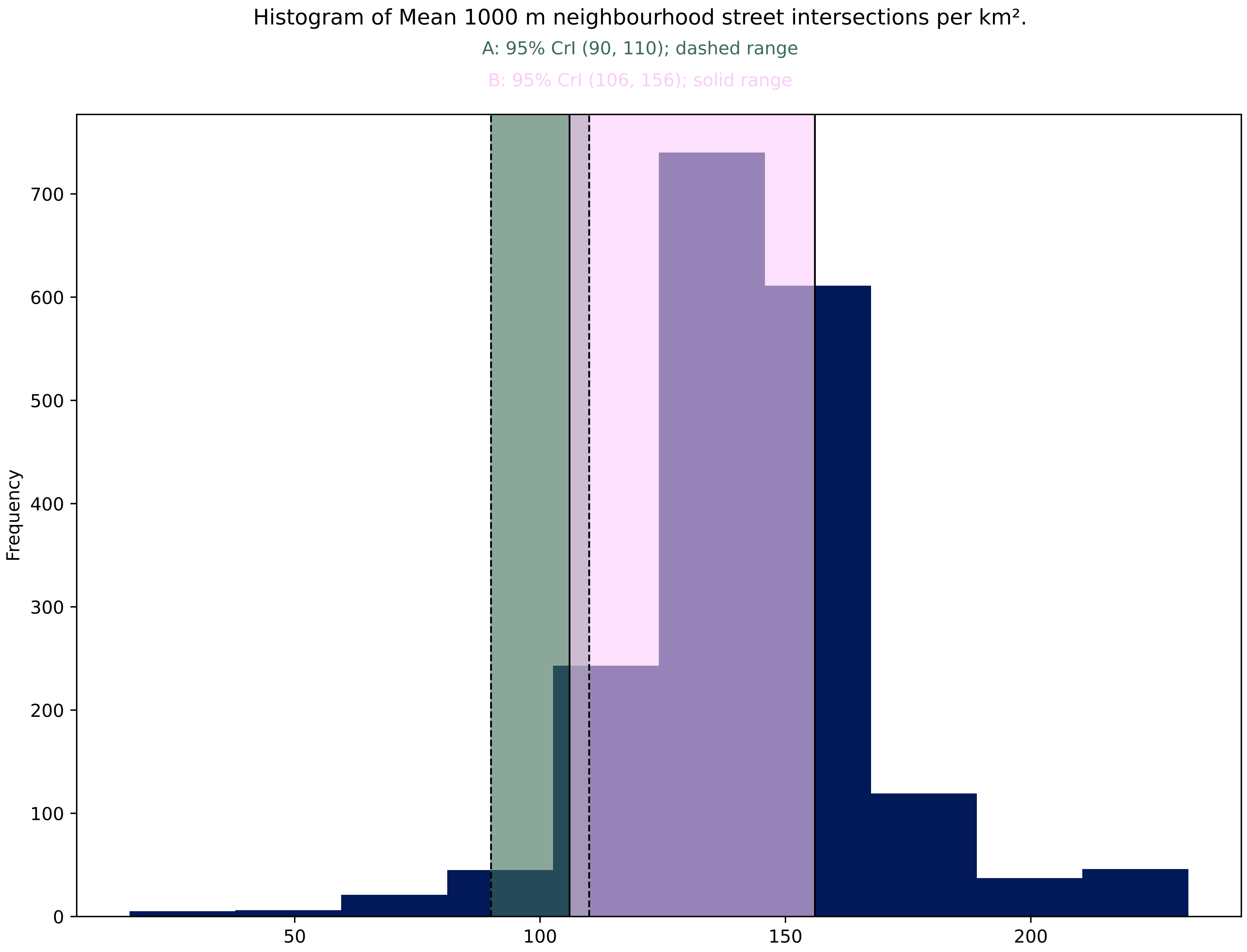
Histogram of Mean 1000 m neighbourhood street intersections per km².
A: 95% CrI (90, 110); dashed range
B: 95% CrI (106, 156); solid range
Frequency
0
100
200
300
400
500
600
700
50
100
150
200

Distance to nearest public transport stops (m; up to 500m)

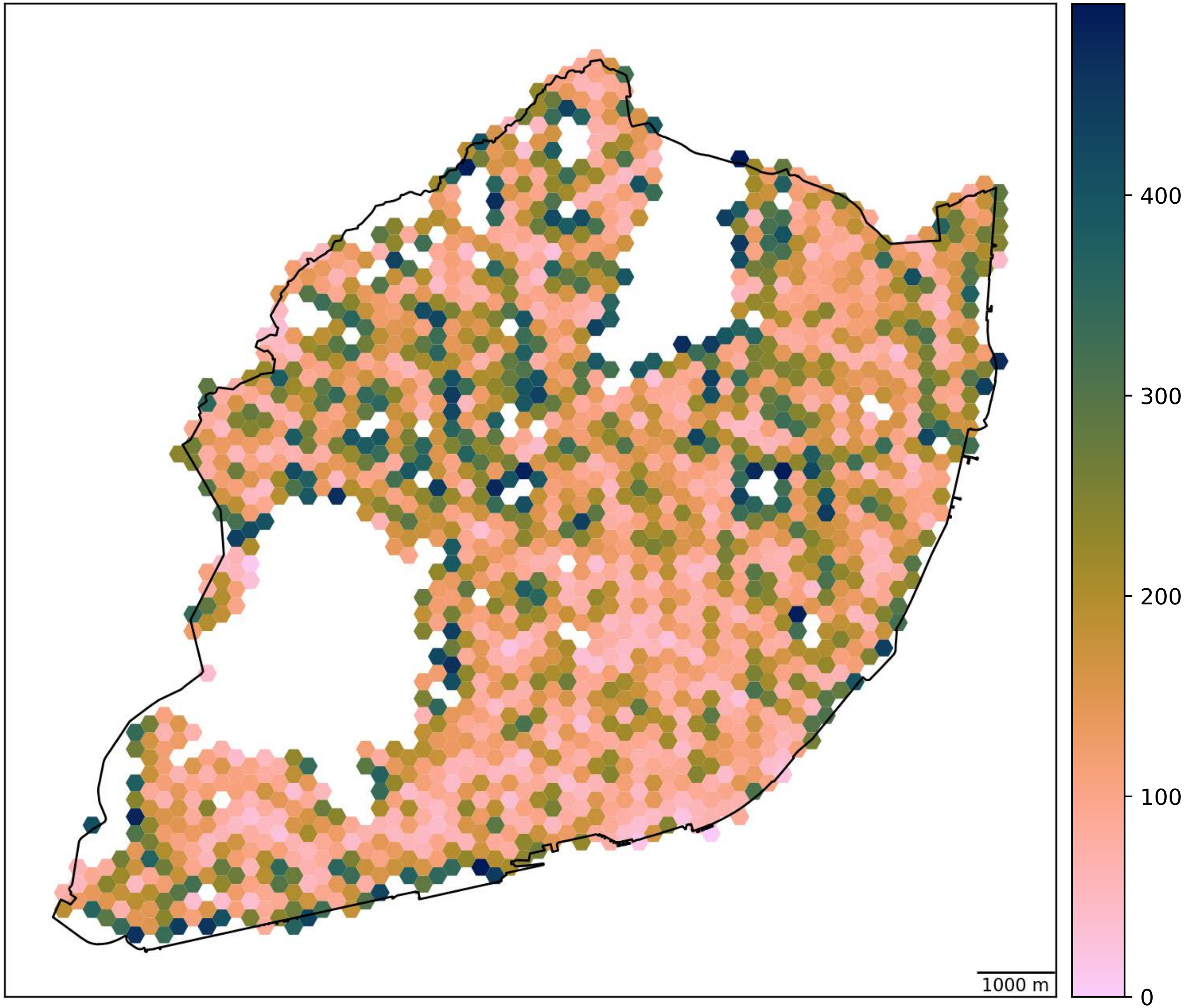

distances: Estimated Distance to nearest public transport stops (m; up to 500m) requirement for distances to destinations, measured up to a maximum distance target threshold of 500 metres

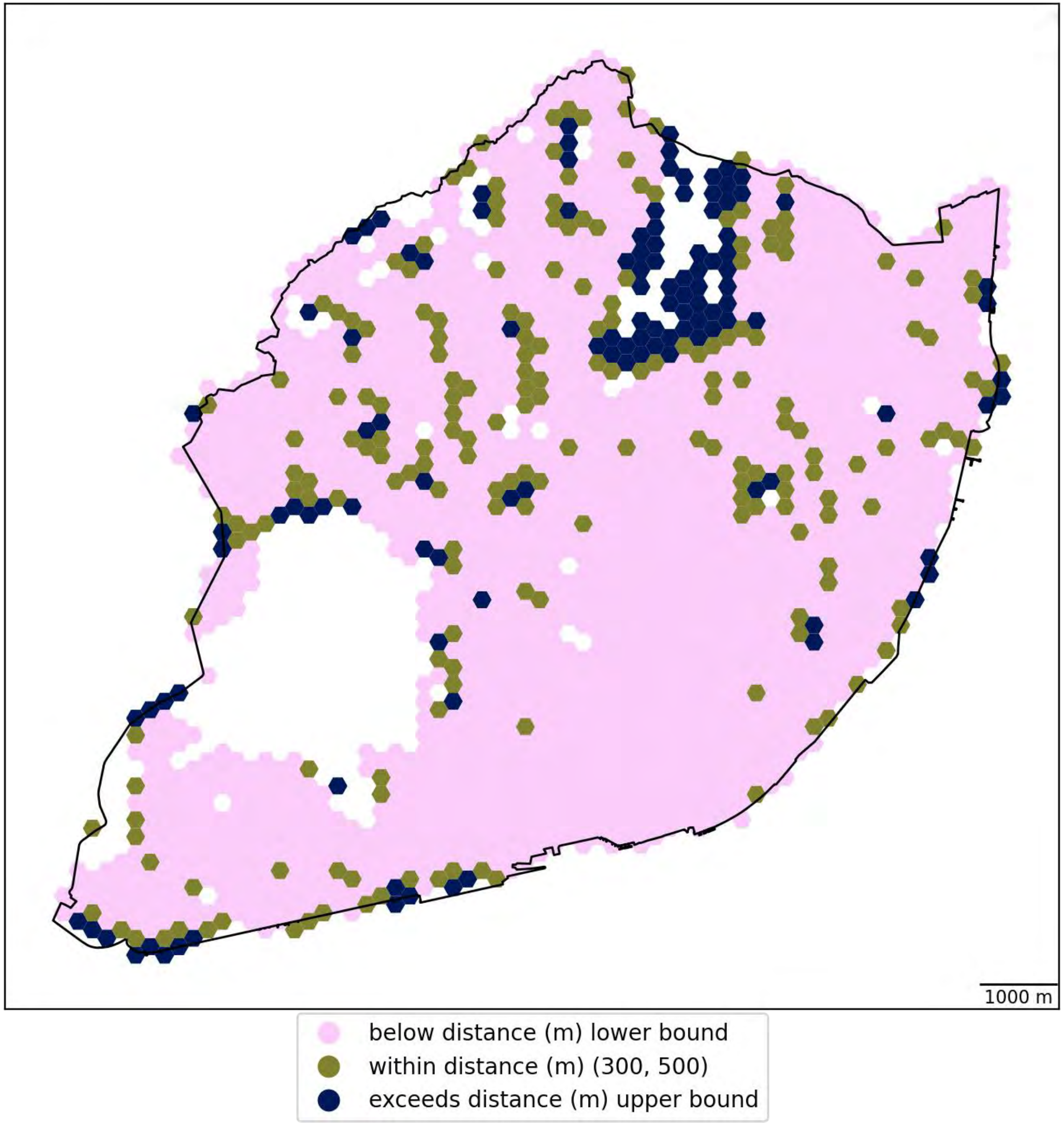

Histogram of Distance to nearest public transport stops (m; up to 500m).

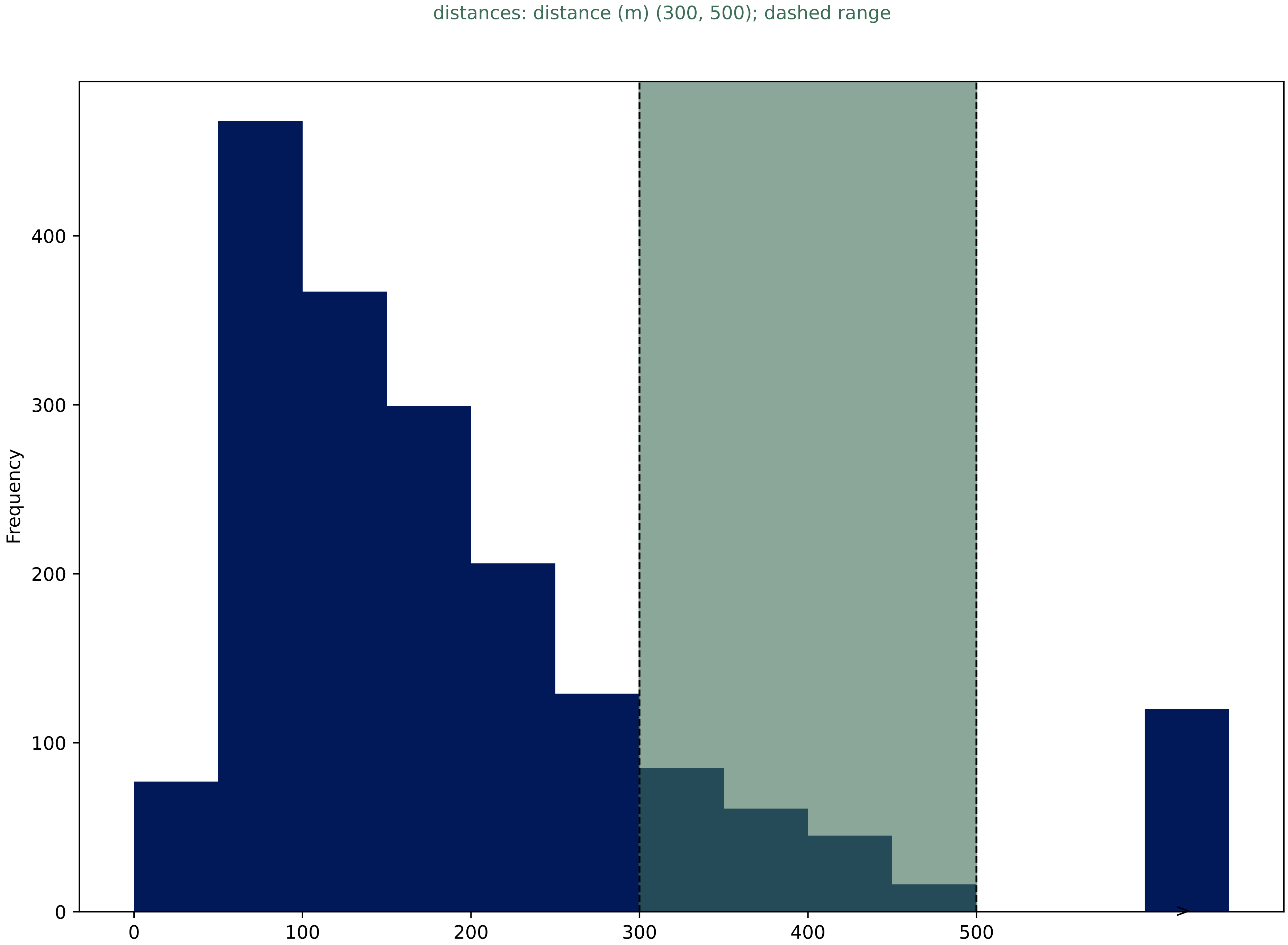

Distance to nearest park (m; up to 500m)

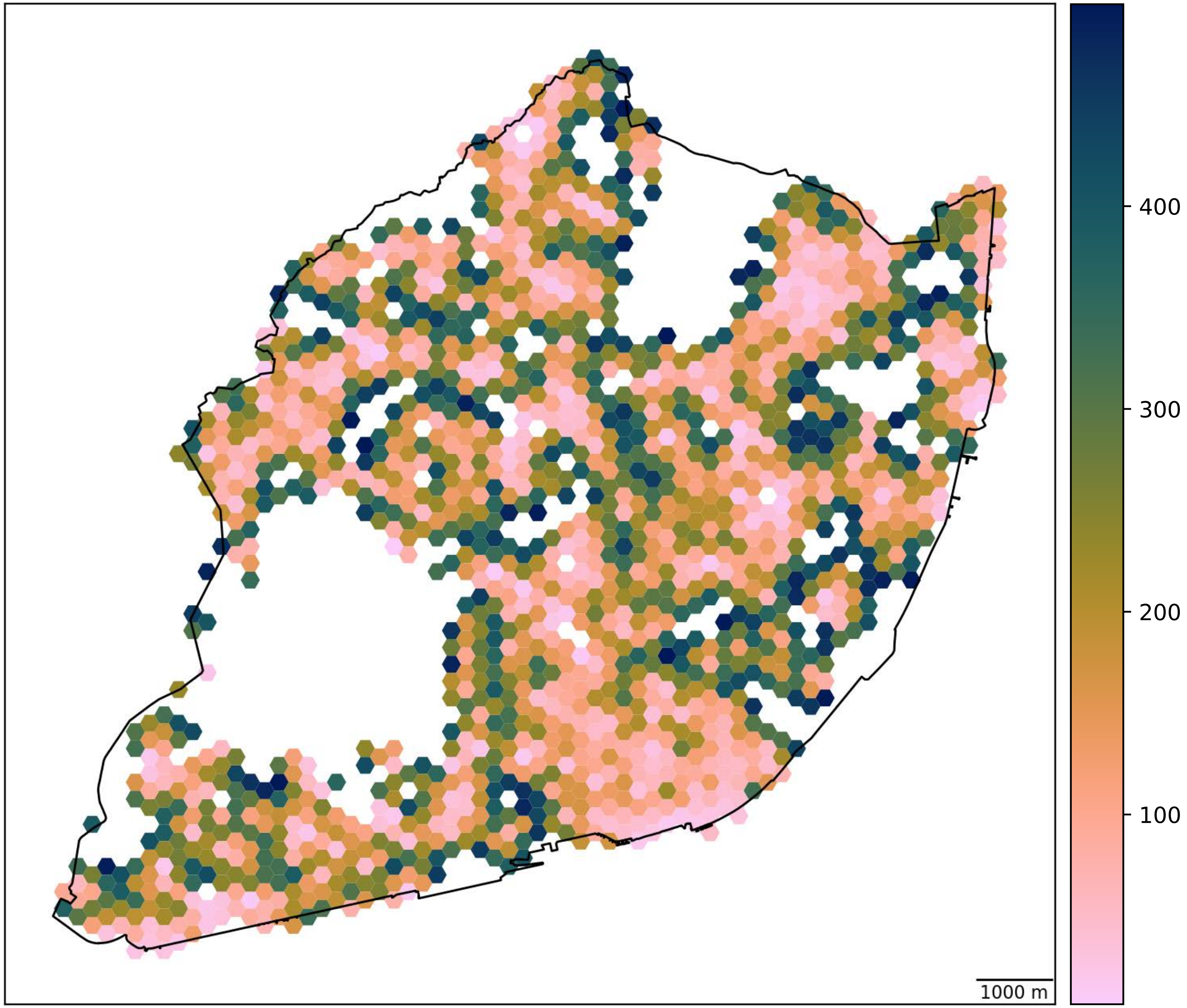

distances: Estimated Distance to nearest park (m; up to 500m) requirement for distances to destinations, measured up to a maximum distance target threshold of 500 metres

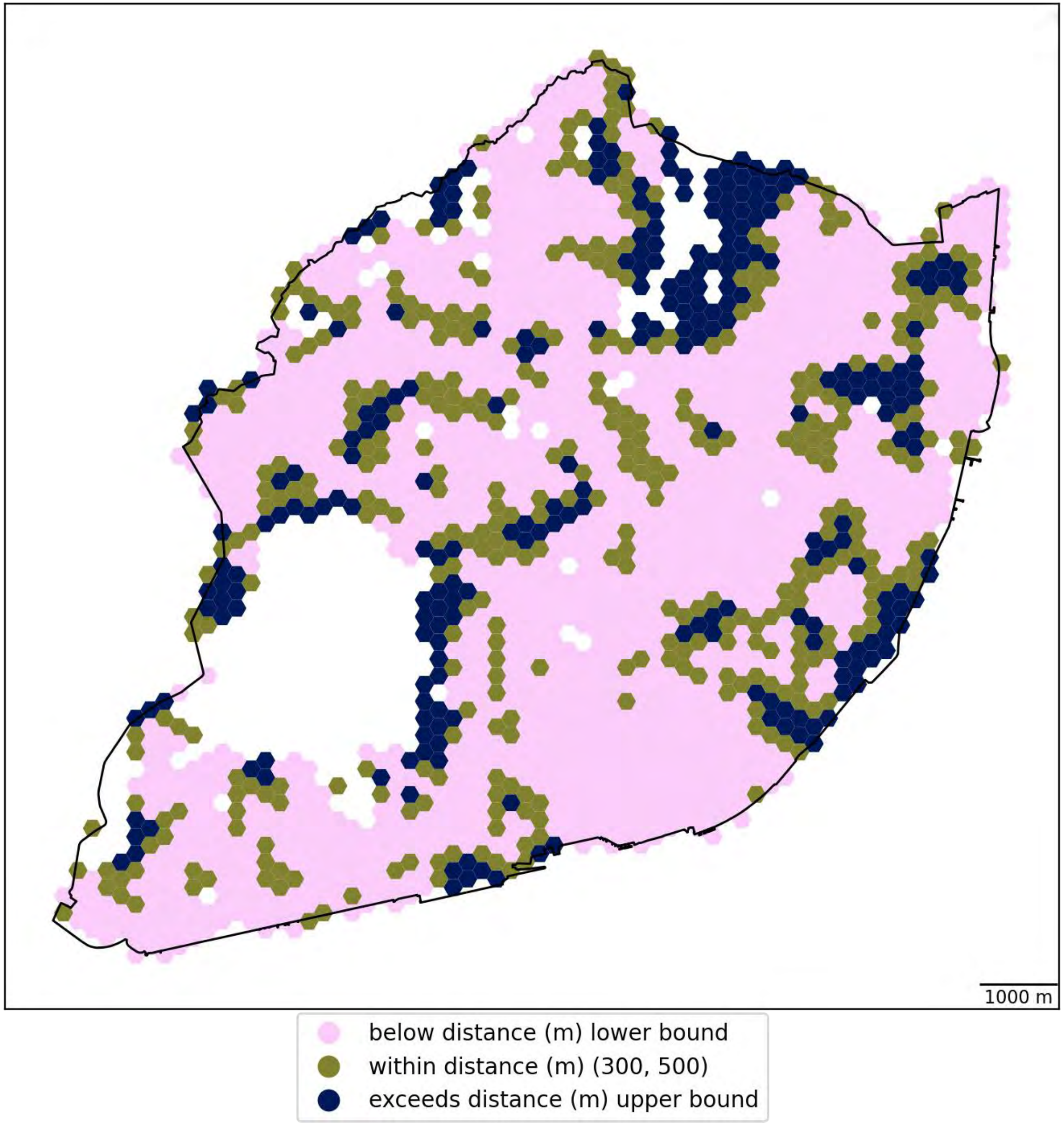

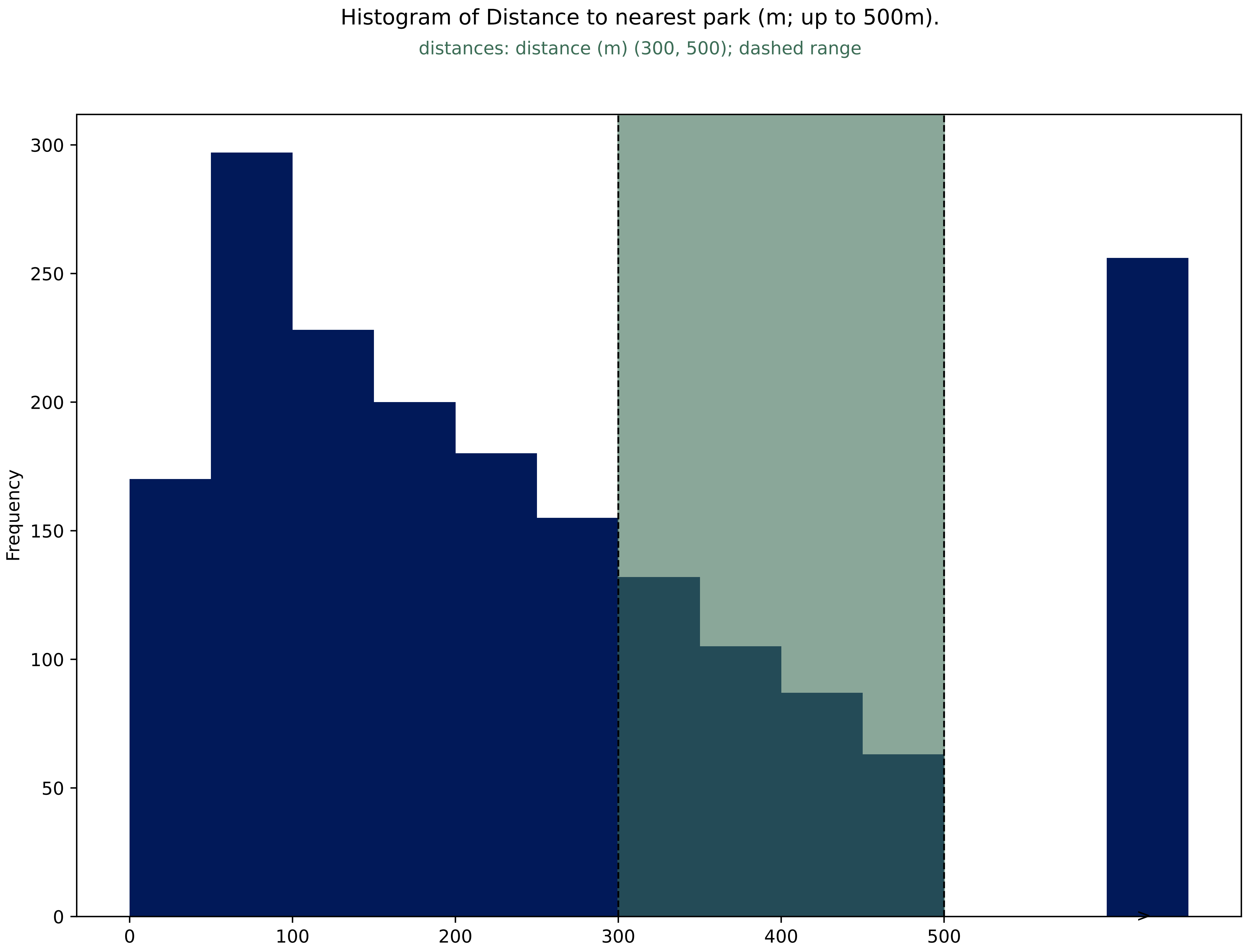

Histogram of Distance to nearest park (m; up to 500m).
distances: distance (m) (300, 500); dashed range
Frequency
0
50
100
150
200
250
300
0
100
200
300
400
500
>

Europe, Spain, Barcelona

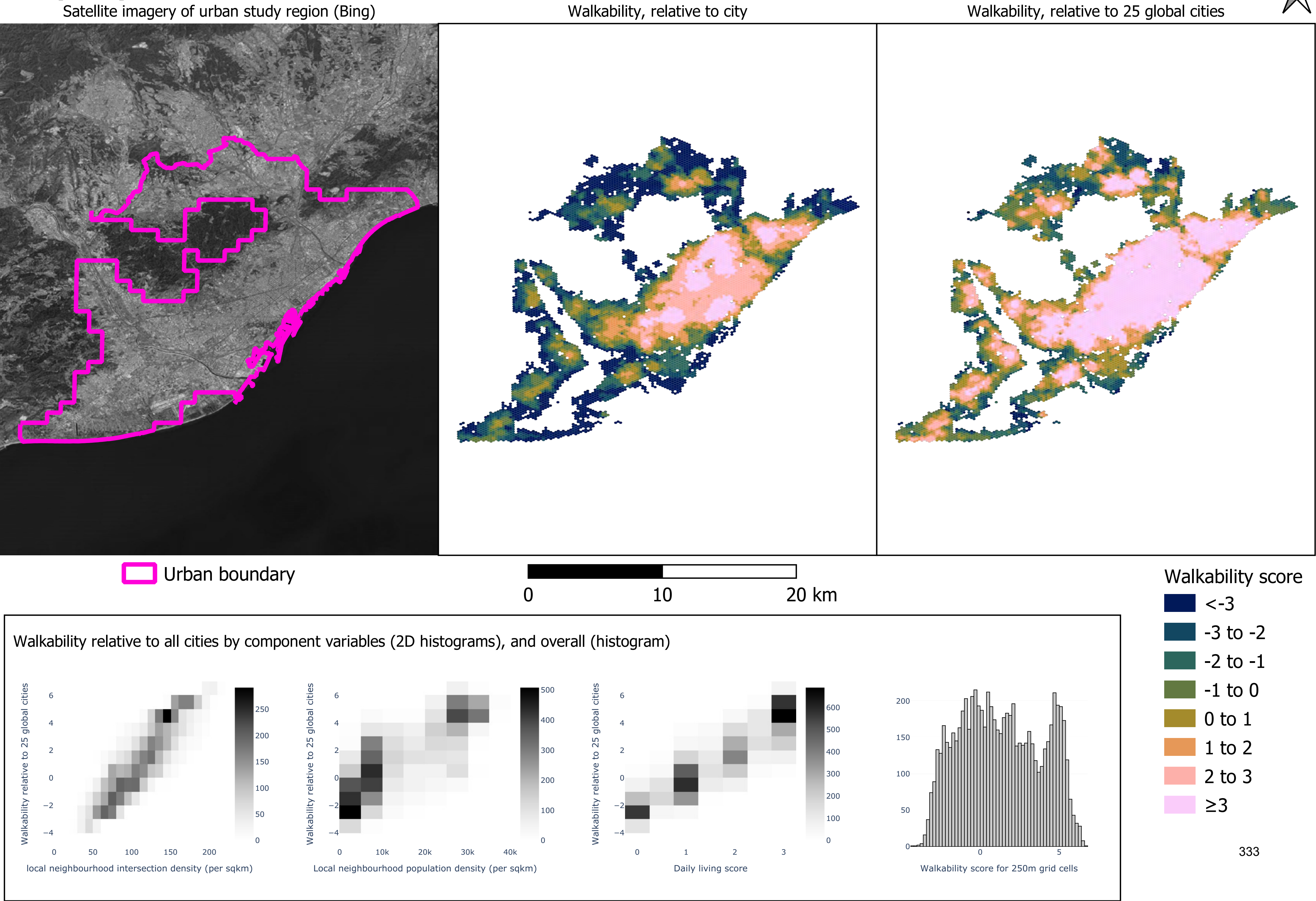

Mean 1000 m neighbourhood population per km²

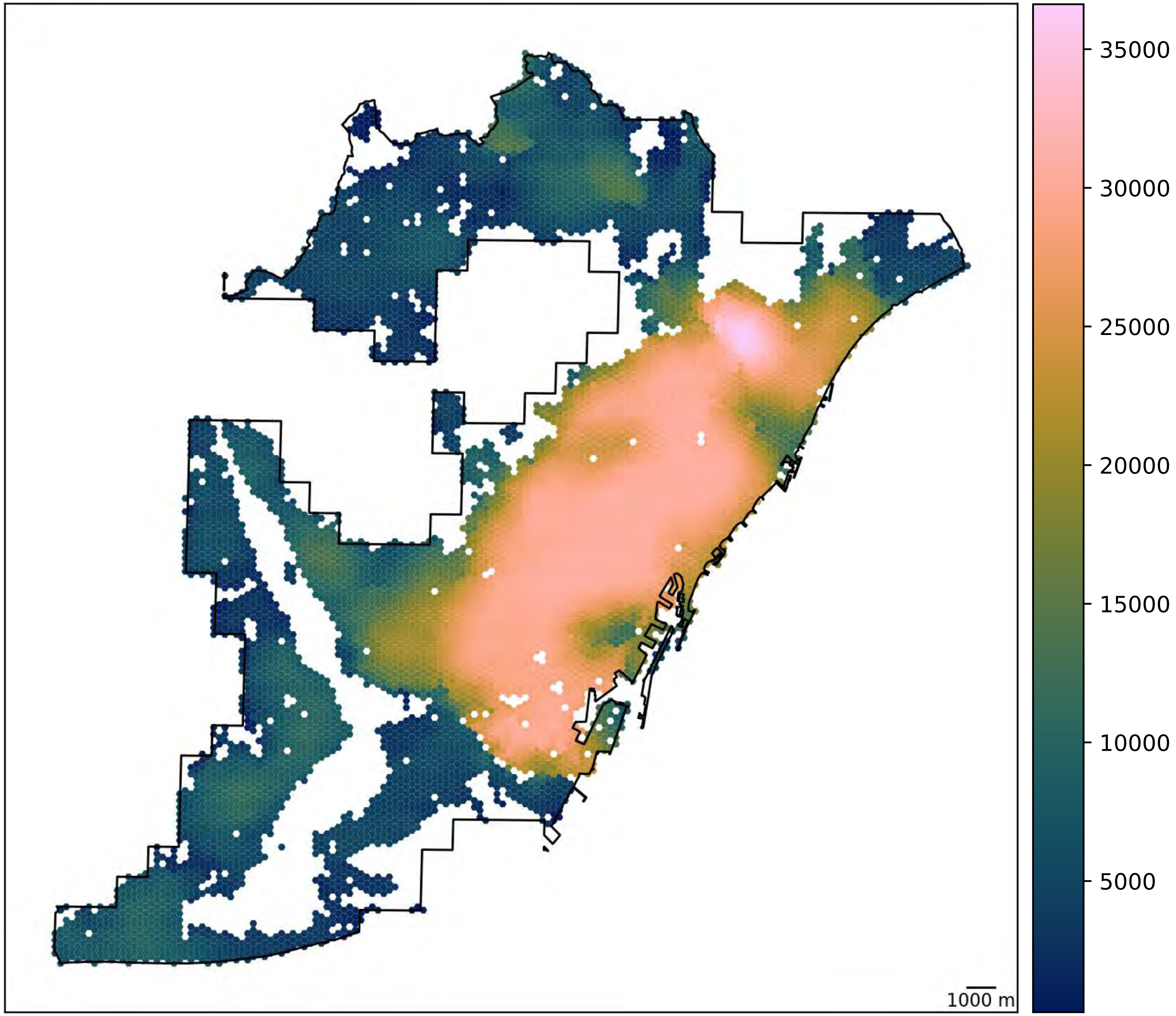

A: Estimated Mean 1000 m neighbourhood population per km² requirement for ≥80% probability of engaging in walking for transport

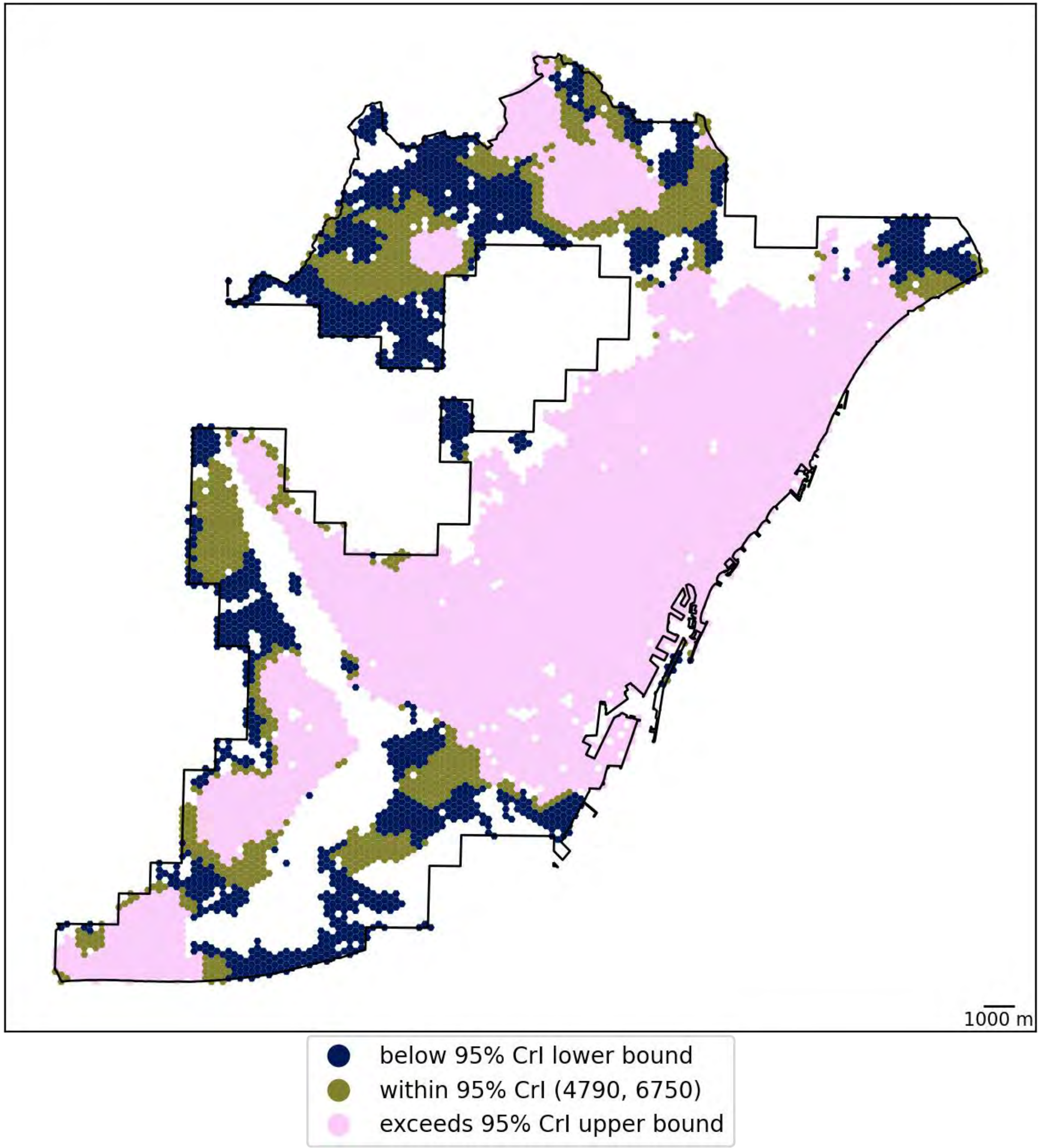

B: Estimated Mean 1000 m neighbourhood population per km² requirement for reaching the WHO's target of a ≥15% relative reduction in insufficient physical activity through walking

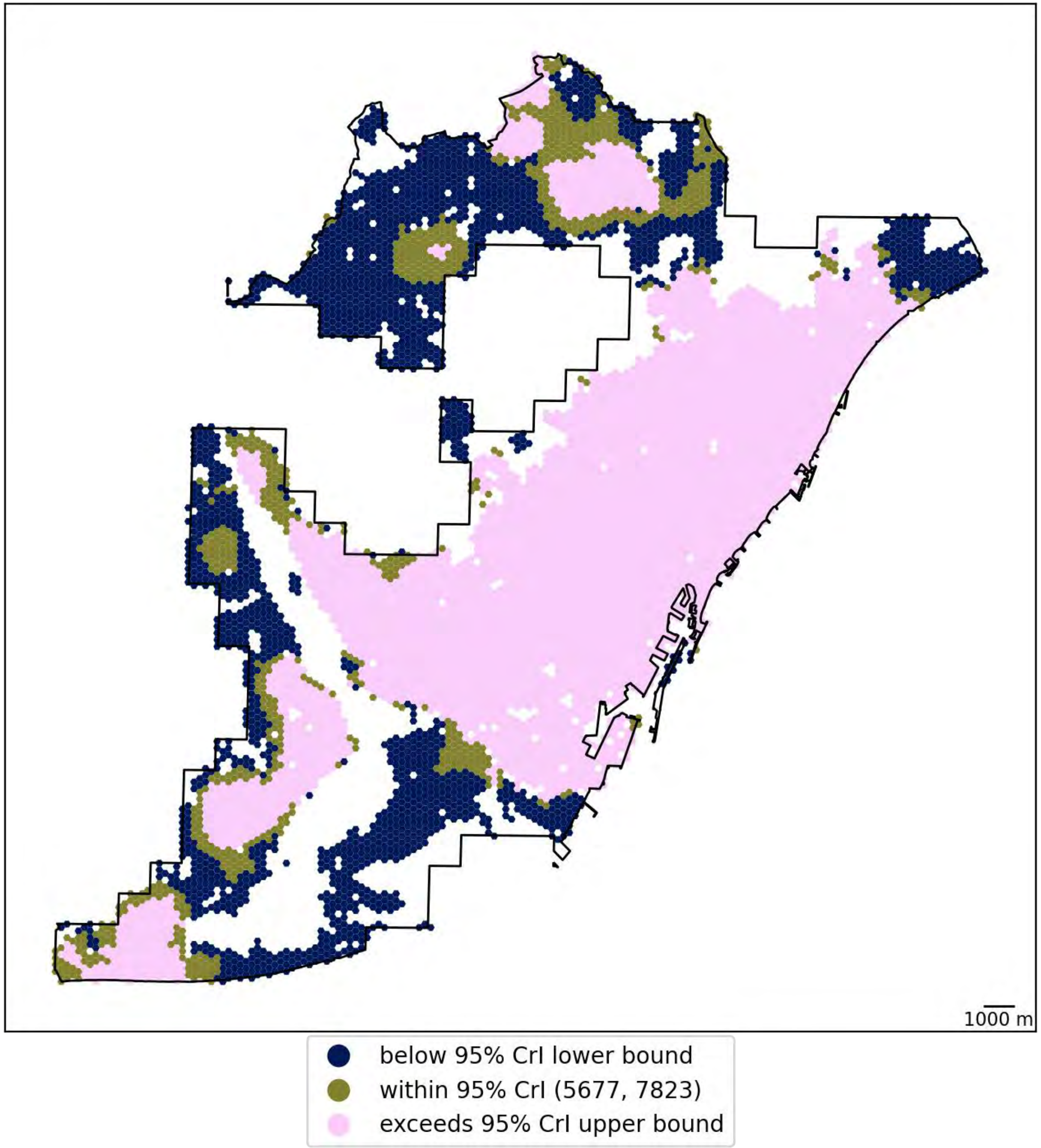

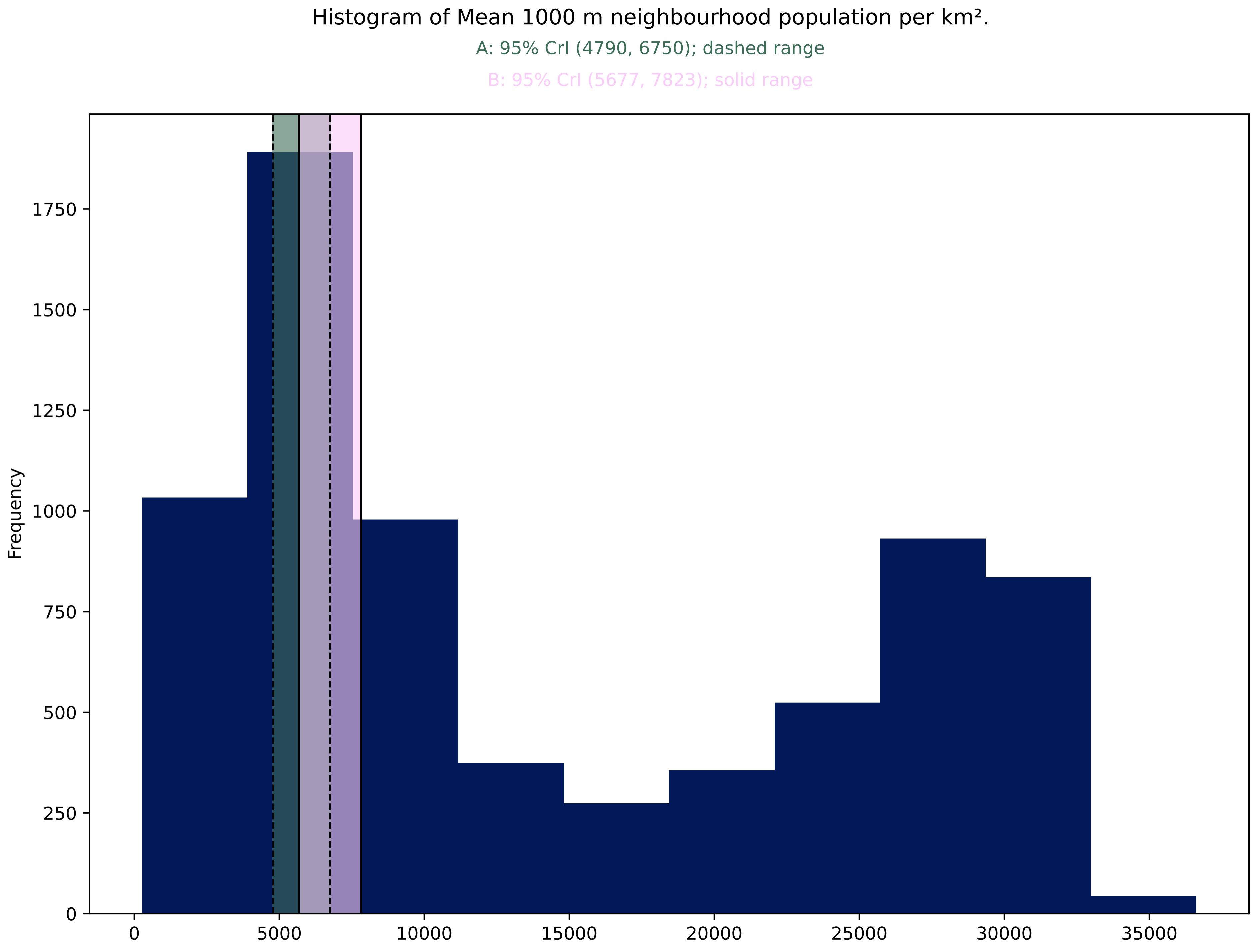

Histogram of Mean 1000 m neighbourhood population per km².
A: 95% CrI (4790, 6750); dashed range
B: 95% CrI (5677, 7823); solid range
Frequency
0
250
500
750
1000
1250
1500
1750
0
5000
10000
15000
20000
25000
30000
35000

Mean 1000 m neighbourhood street intersections per km²

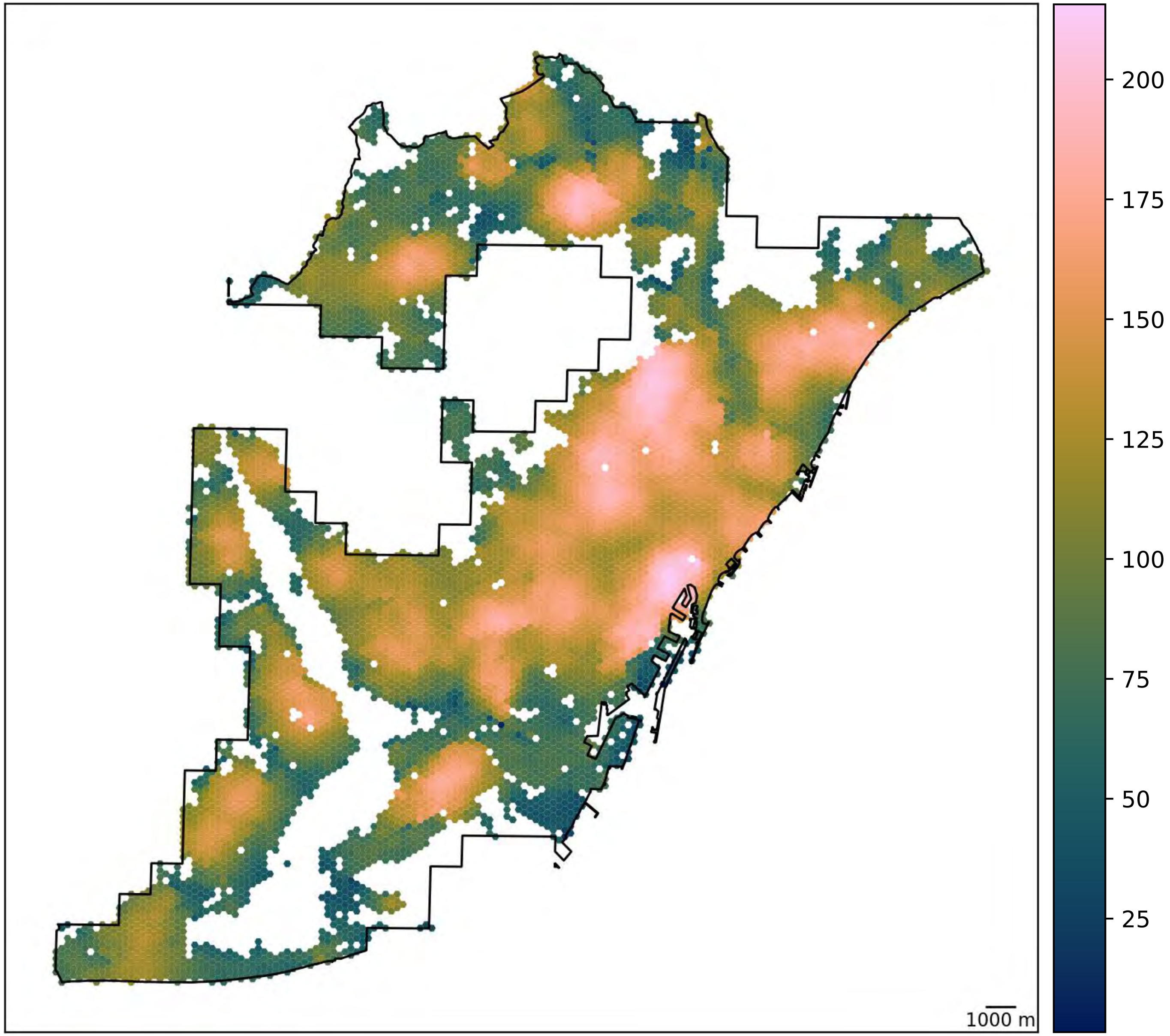

A: Estimated Mean 1000 m neighbourhood street intersections per km² requirement for ≥80% probability of engaging in walking for transport

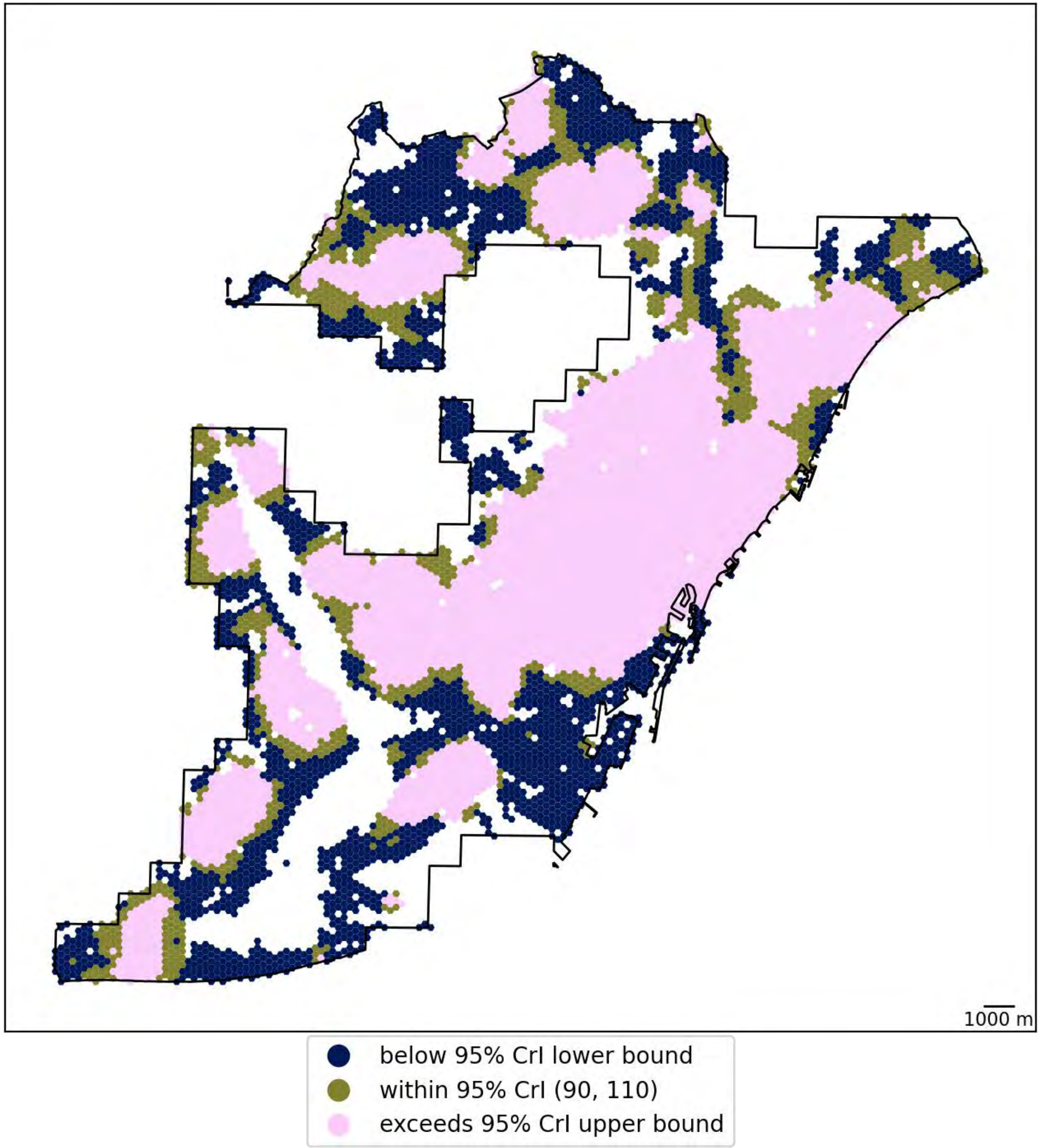

B: Estimated Mean 1000 m neighbourhood street intersections per km² requirement for reaching the WHO's target of a ≥15% relative reduction in insufficient physical activity through walking

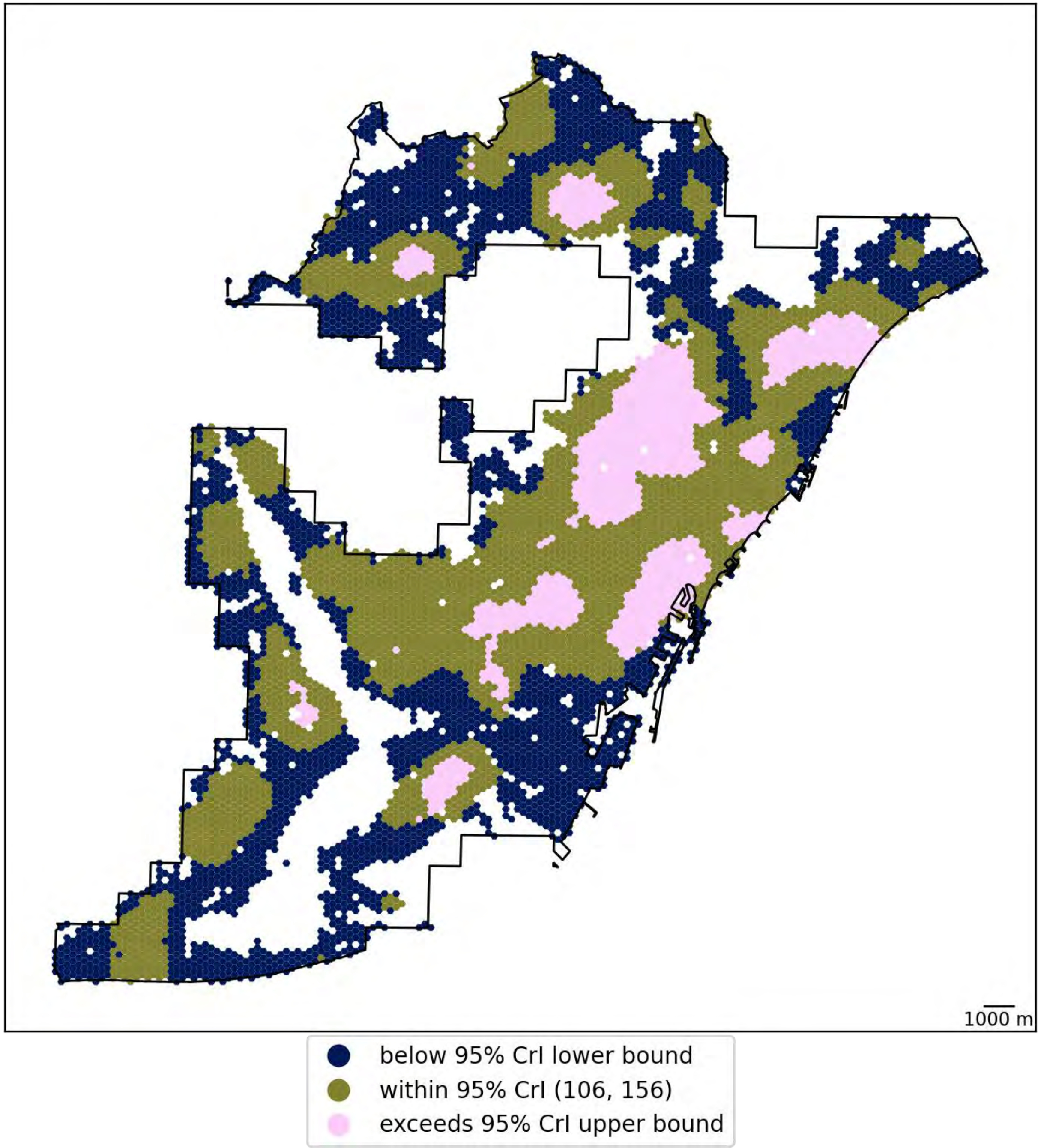

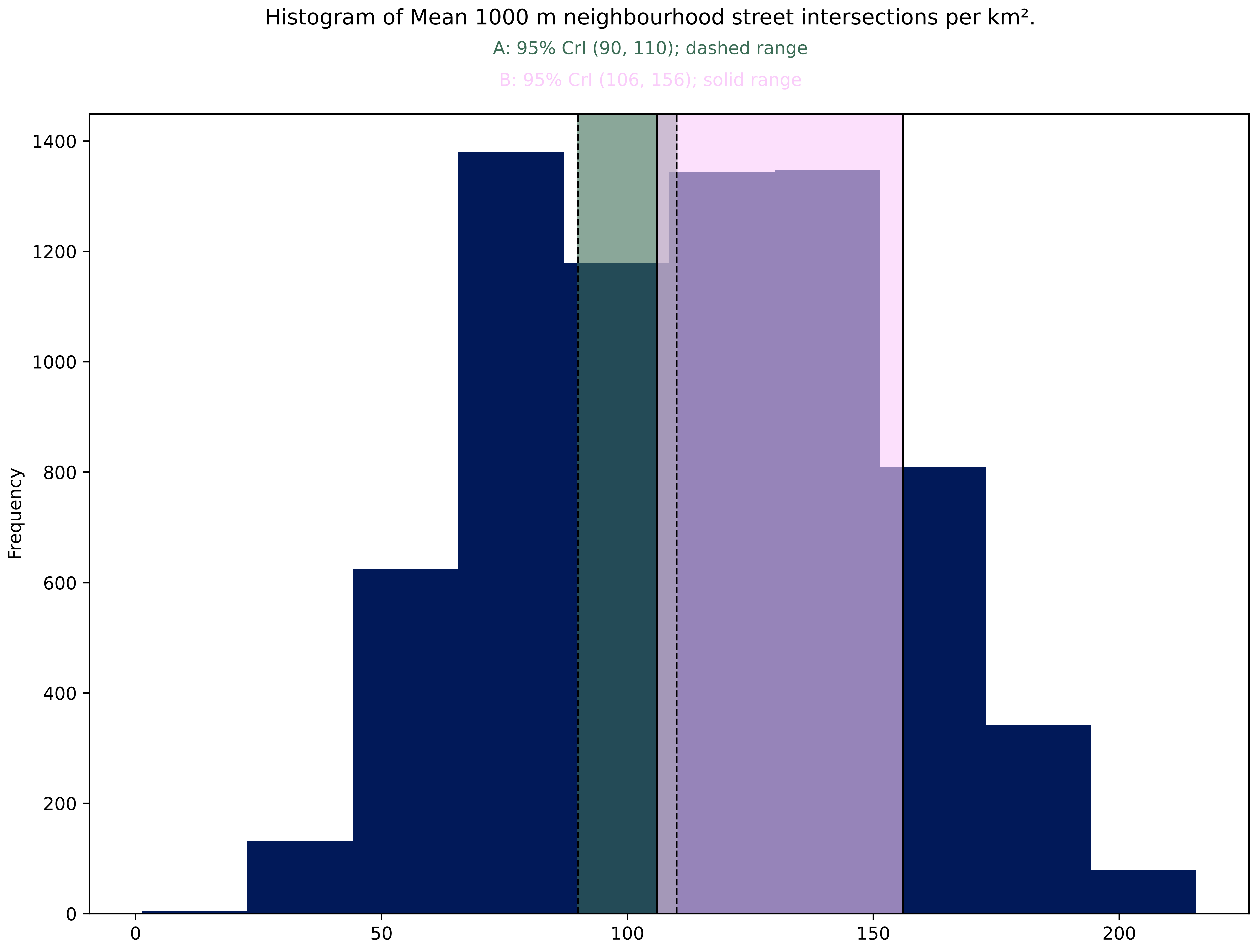
Histogram of Mean 1000 m neighbourhood street intersections per km².
A: 95% CrI (90, 110); dashed range
B: 95% CrI (106, 156); solid range
Frequency
0
200
400
600
800
1000
1200
1400
0
50
100
150
200

Distance to nearest public transport stops (m; up to 500m)

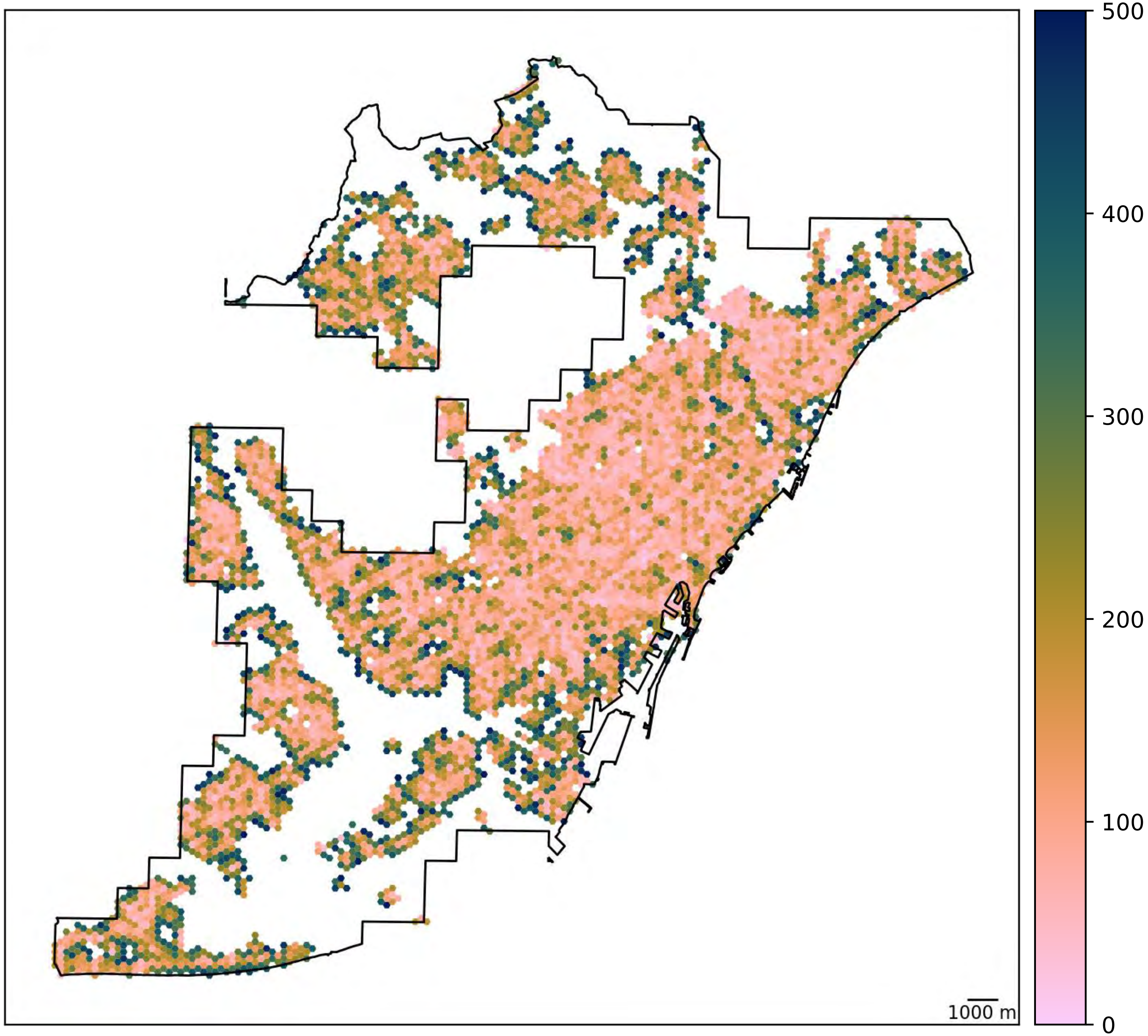

distances: Estimated Distance to nearest public transport stops (m; up to 500m) requirement for distances to destinations, measured up to a maximum distance target threshold of 500 metres

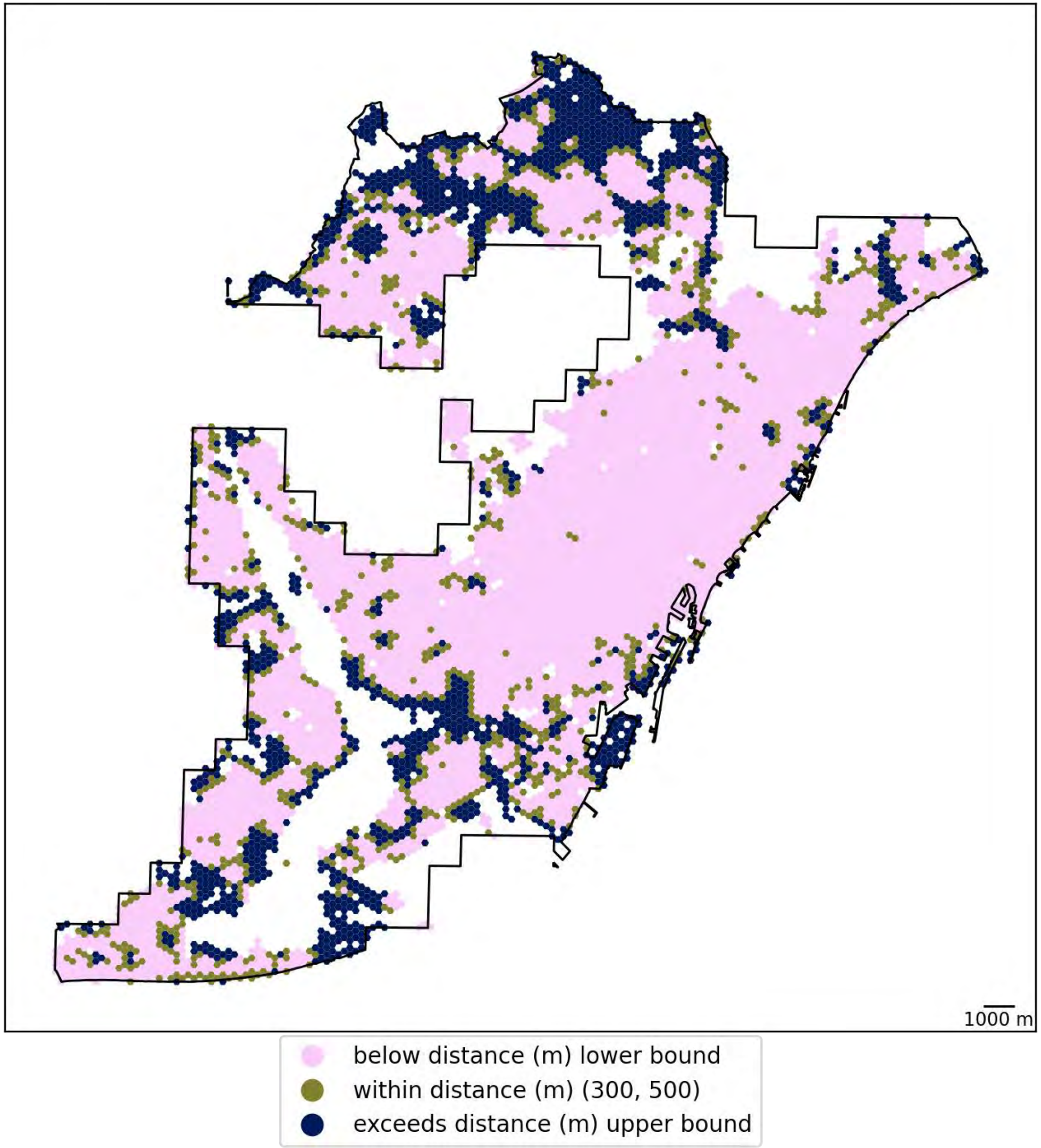

Histogram of Distance to nearest public transport stops (m; up to 500m).

distances: distance (m) (300, 500); dashed range

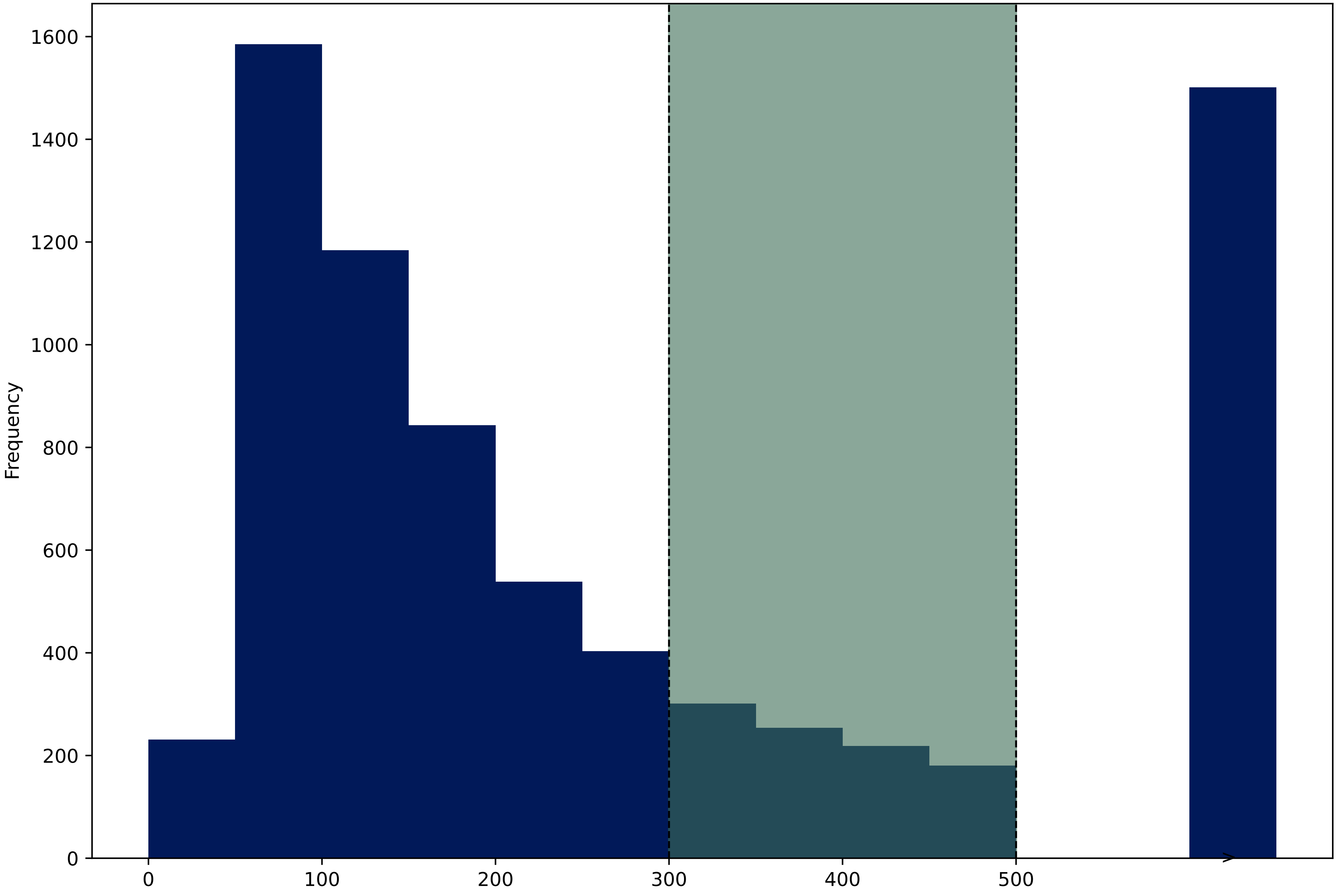

Distance to nearest park (m; up to 500m)

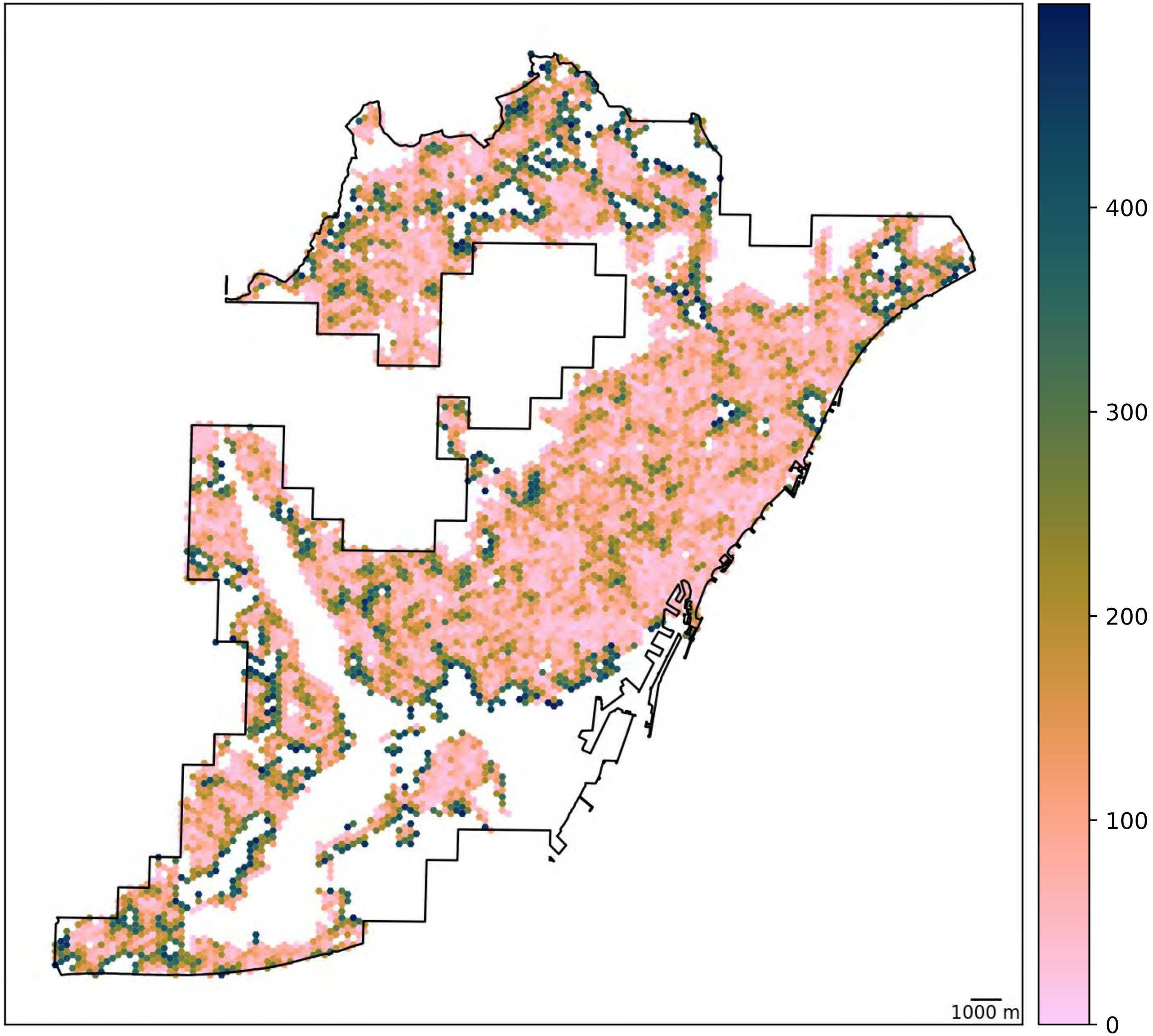

distances: Estimated Distance to nearest park (m; up to 500m) requirement for distances to destinations, measured up to a maximum distance target threshold of 500 metres

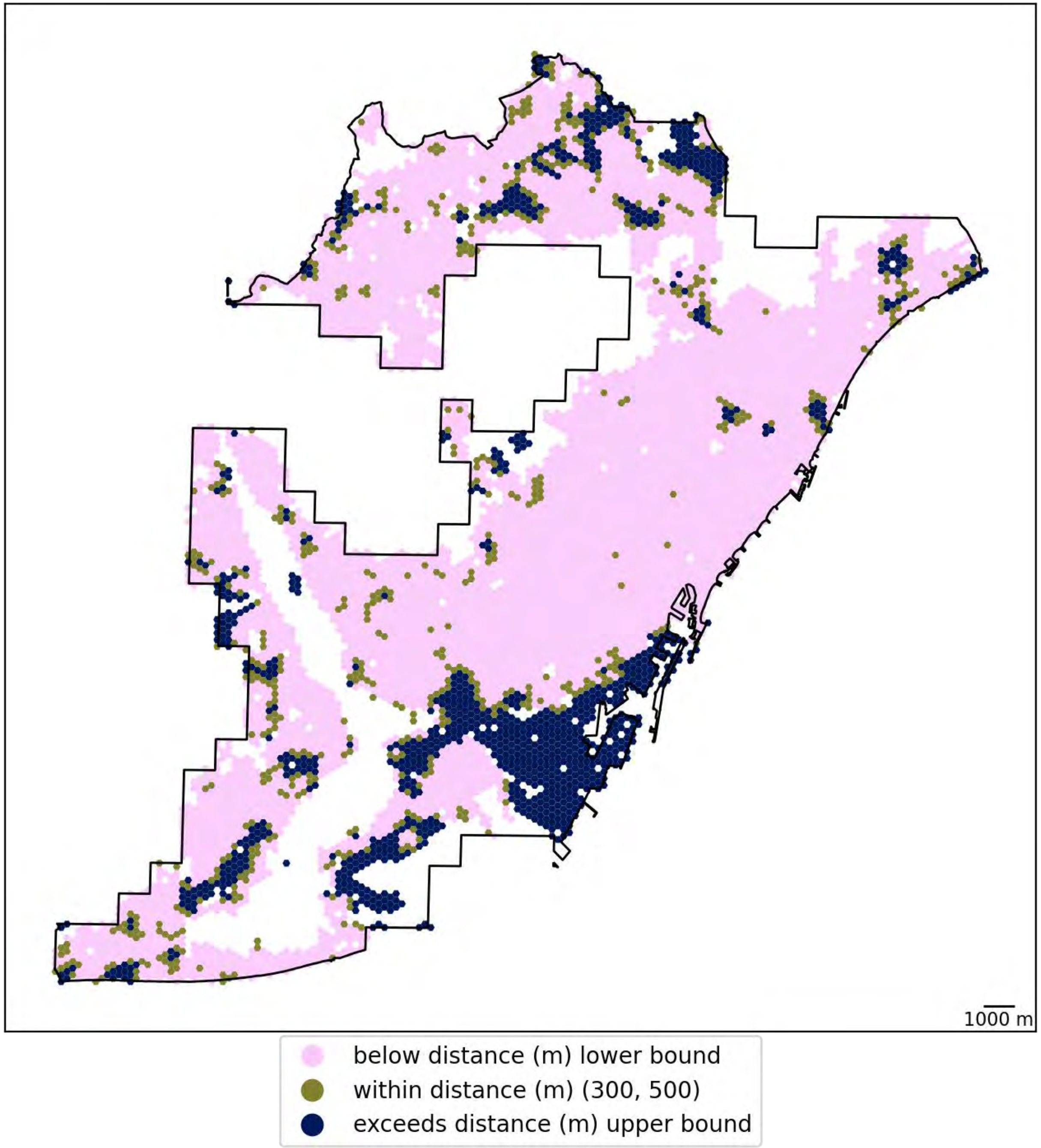

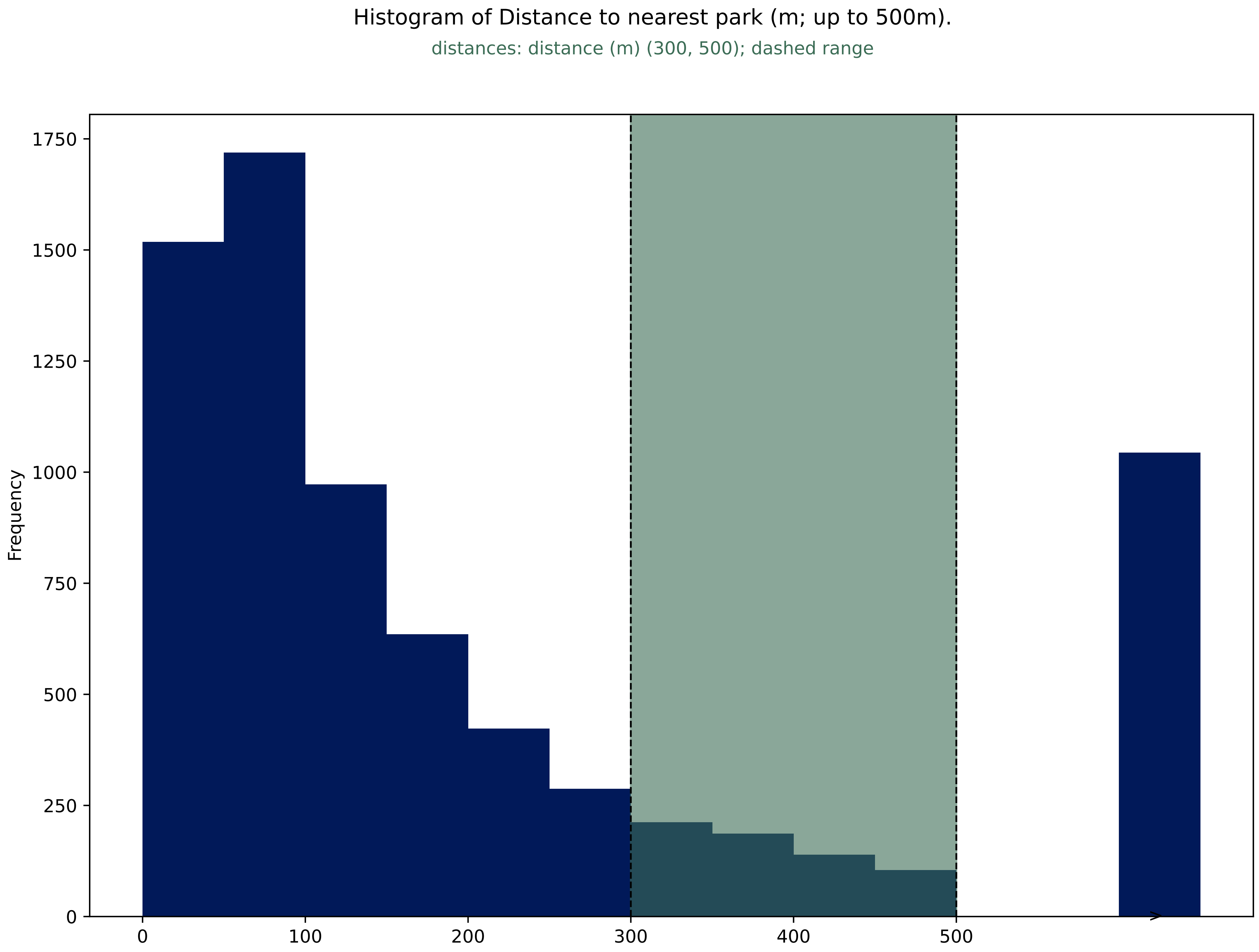

Histogram of Distance to nearest park (m; up to 500m).
distances: distance (m) (300, 500); dashed range
Frequency
1750
1500
1250
1000
750
500
250
0
0
100
200
300
400
500

Europe, Spain, Valencia

Satellite imagery of urban study region (Bing)

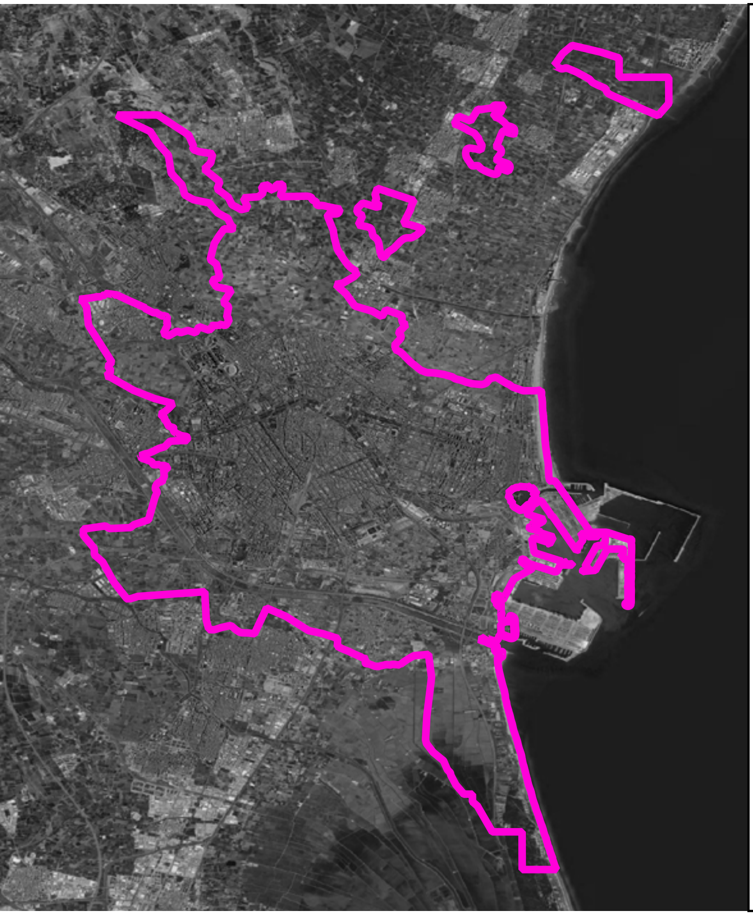

Walkability, relative to city

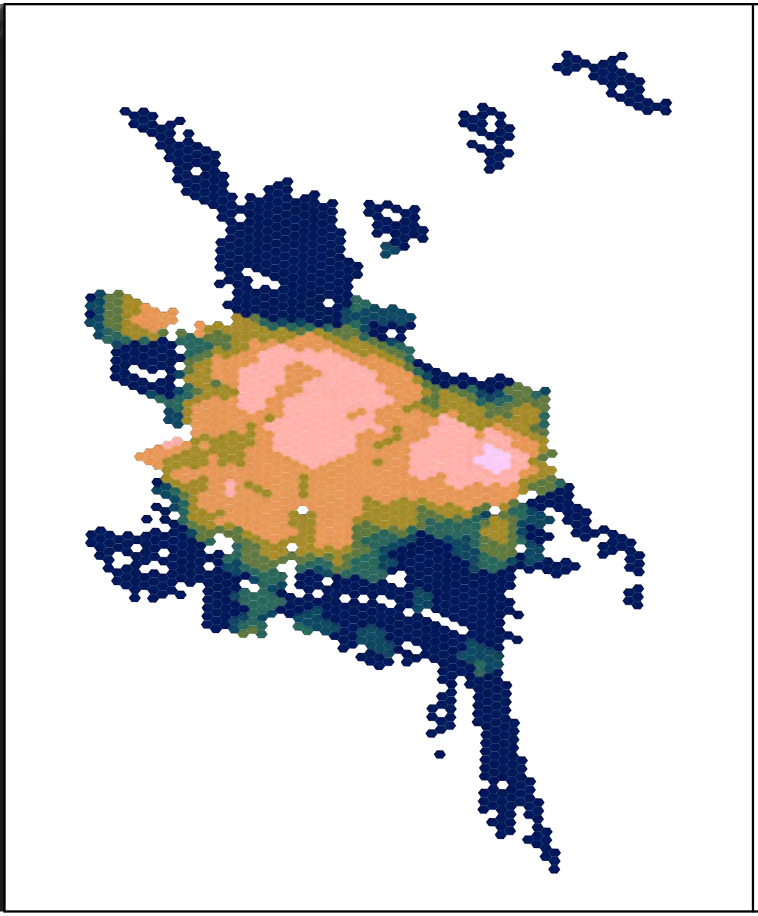

Walkability, relative to 25 global cities

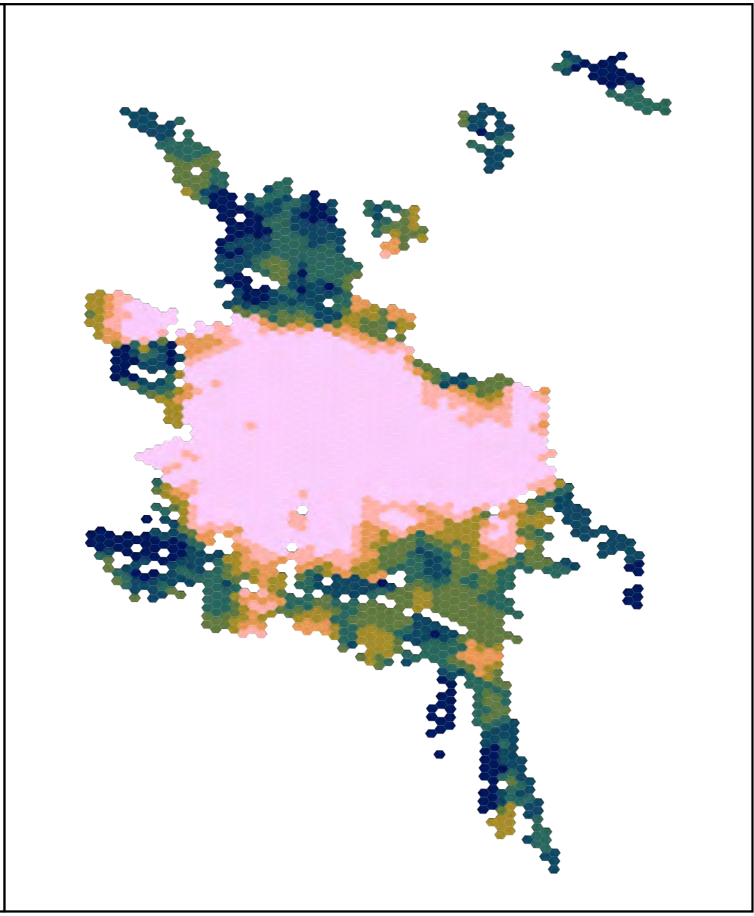

Urban boundary

0 6 12 km

Walkability score

- <-3
- -3 to -2
- -2 to -1
- -1 to 0
- 0 to 1
- 1 to 2
- 2 to 3
- ≥3

Walkability relative to all cities by component variables (2D histograms), and overall (histogram)

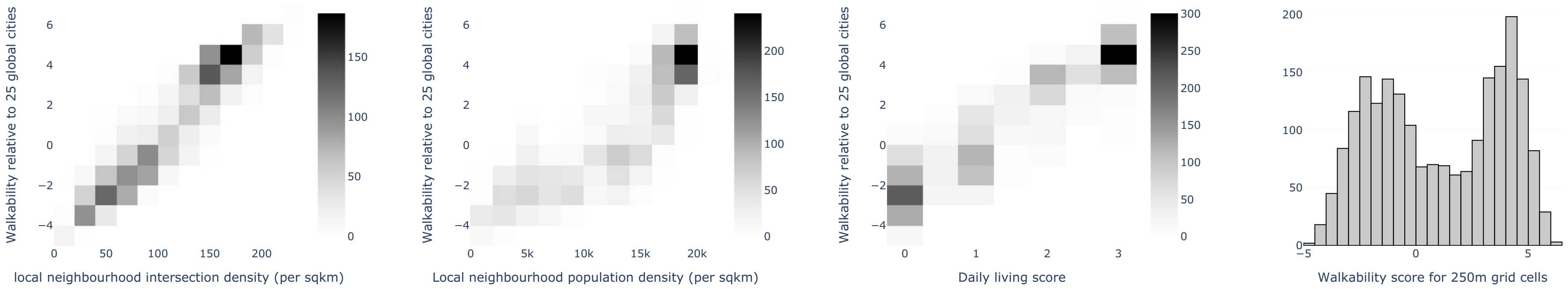

Mean 1000 m neighbourhood population per km²

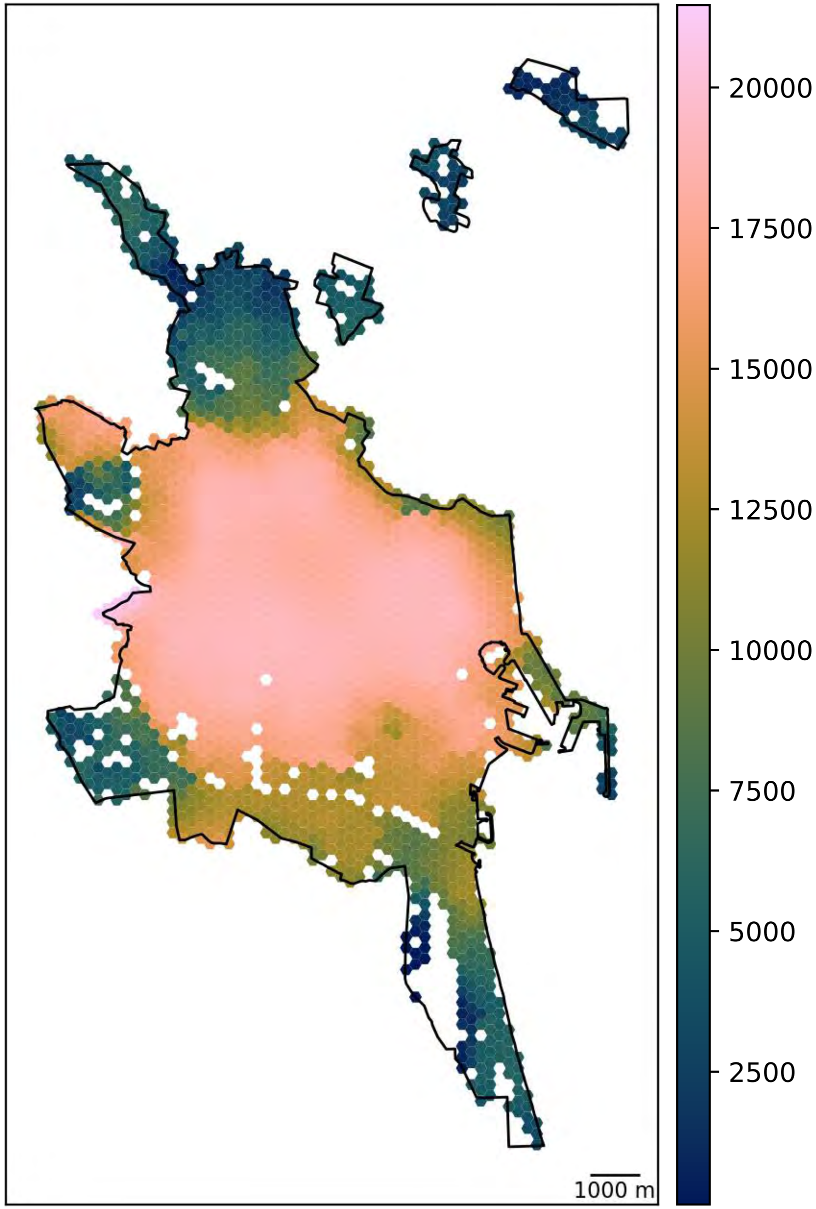

A: Estimated Mean 1000 m neighbourhood population per km² requirement for ≥80% probability of engaging in walking for transport

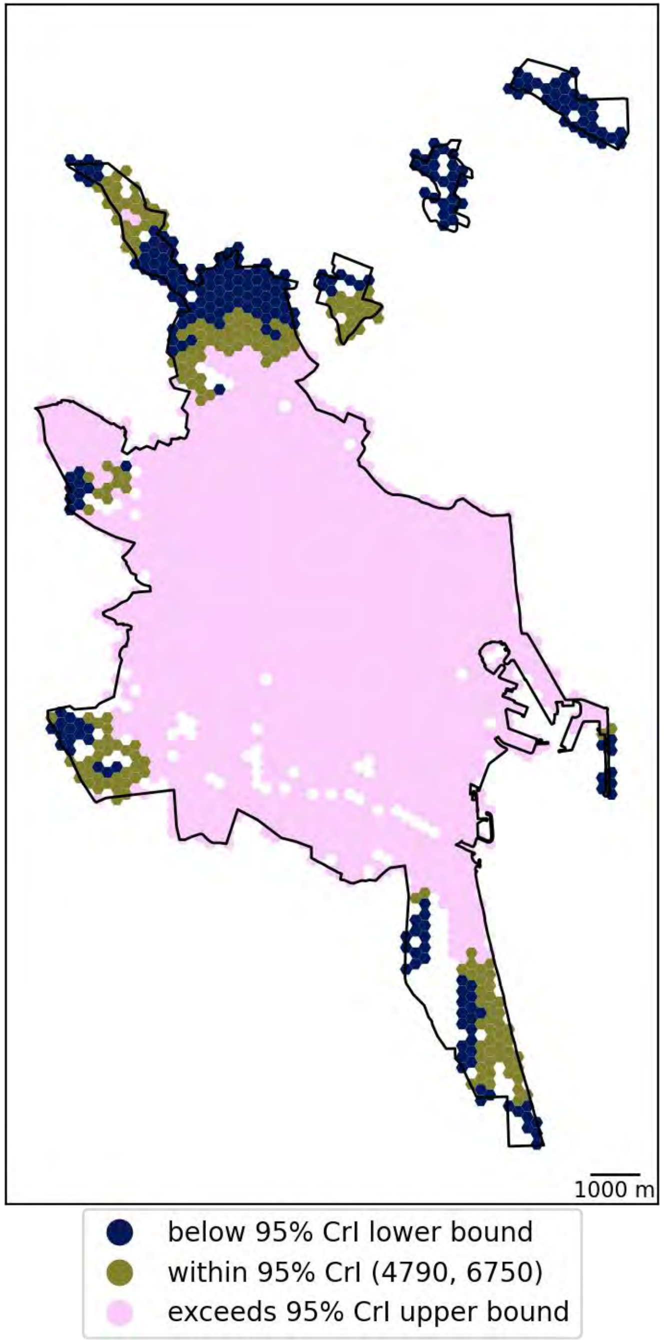

B: Estimated Mean 1000 m neighbourhood population per km² requirement for reaching the WHO's target of a ≥15% relative reduction in insufficient physical activity through walking

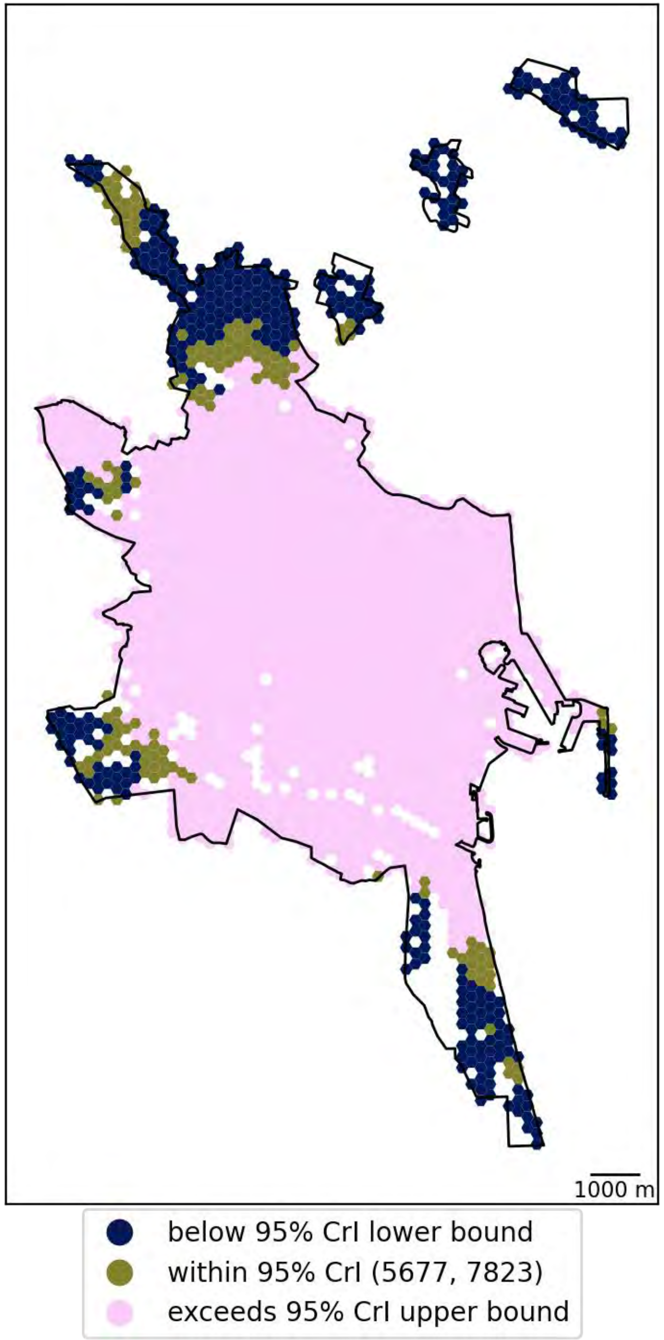

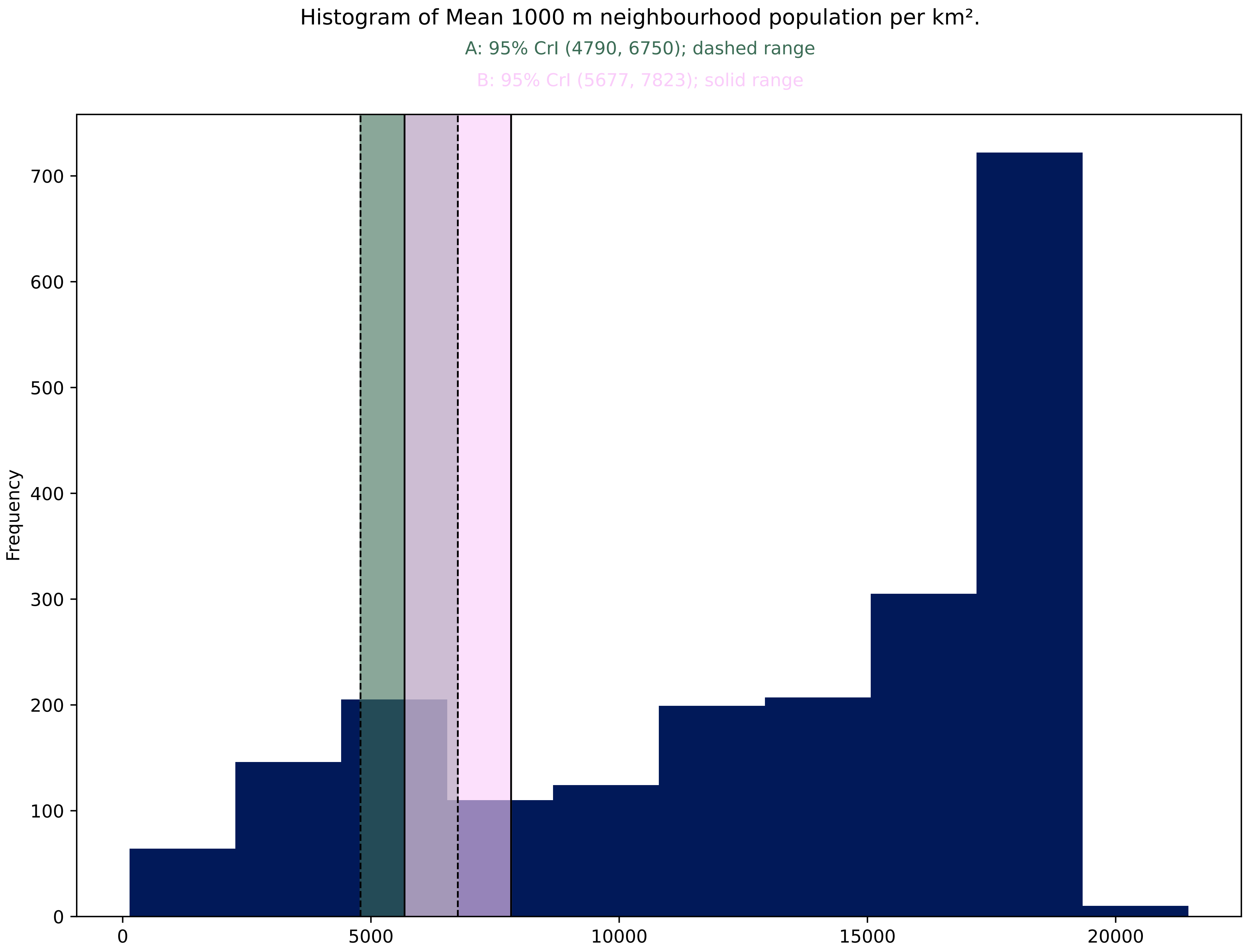

Histogram of Mean 1000 m neighbourhood population per km².
A: 95% CrI (4790, 6750); dashed range
B: 95% CrI (5677, 7823); solid range
Frequency
0
100
200
300
400
500
600
700
0
5000
10000
15000
20000

Mean 1000 m neighbourhood street intersections per km²

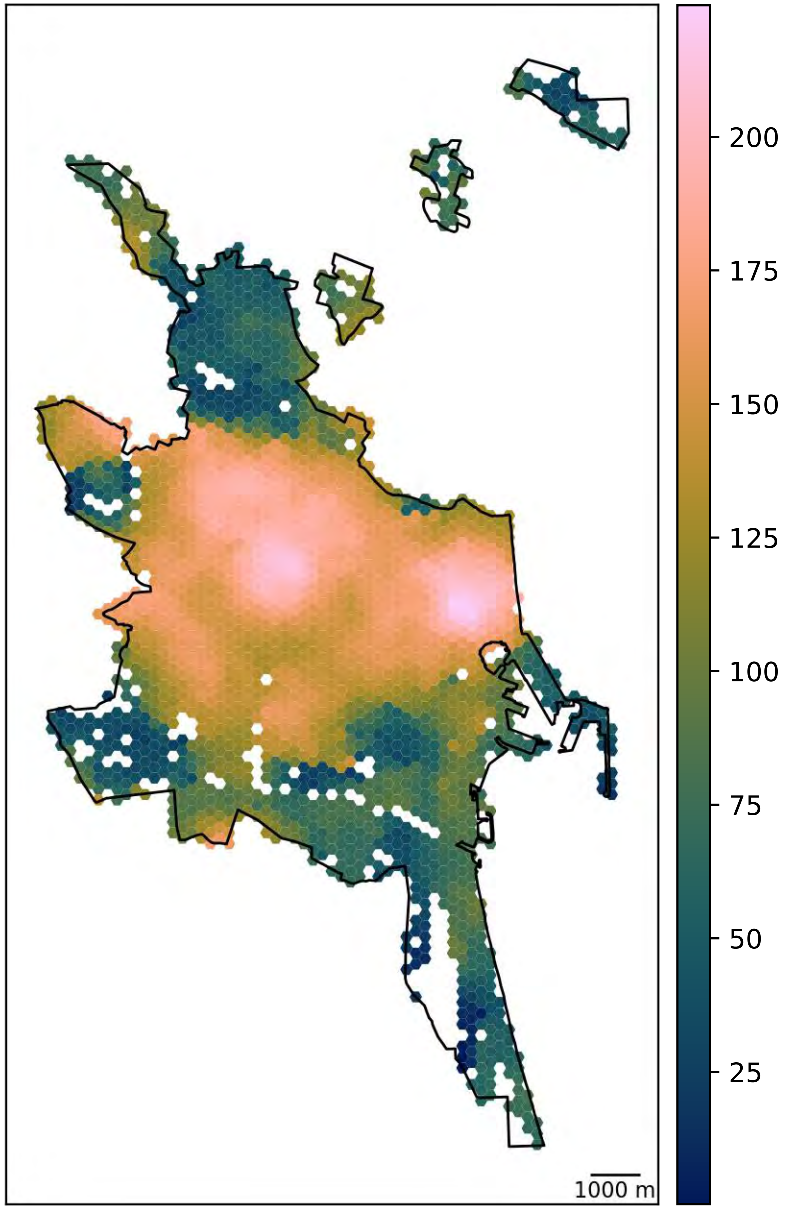

A: Estimated Mean 1000 m neighbourhood street intersections per km² requirement for ≥80% probability of engaging in walking for transport

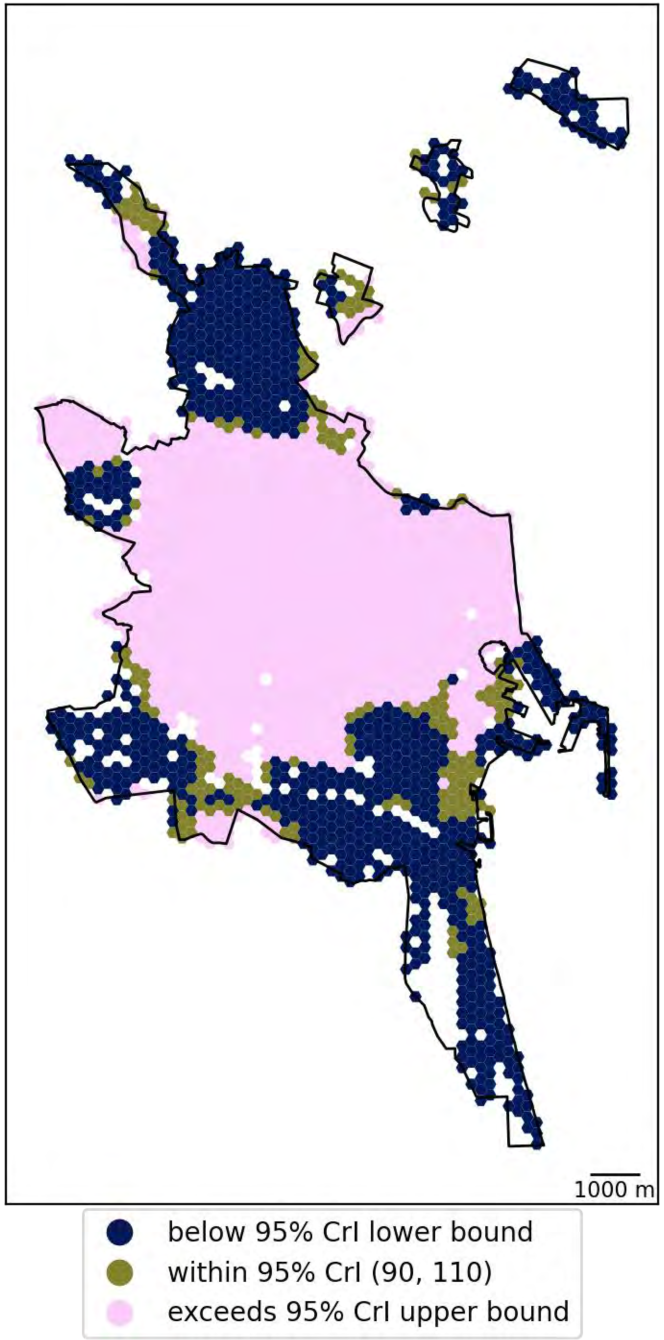

B: Estimated Mean 1000 m neighbourhood street intersections per km² requirement for reaching the WHO's target of a ≥15% relative reduction in insufficient physical activity through walking

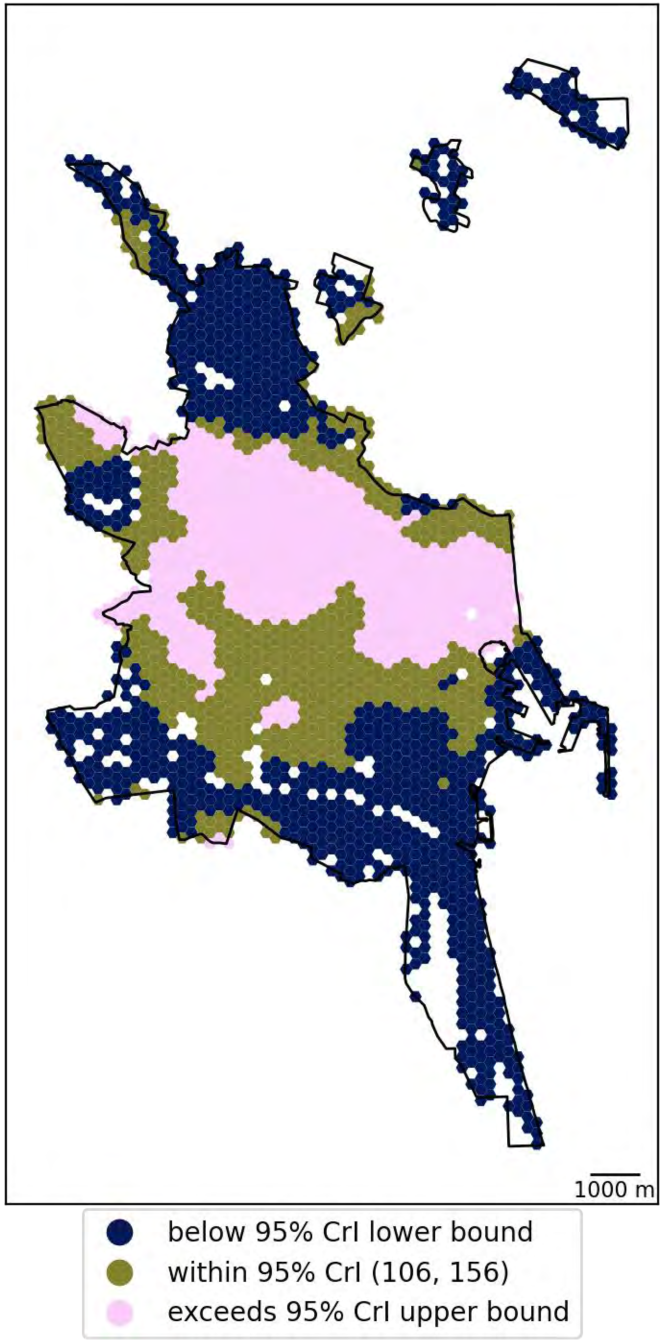

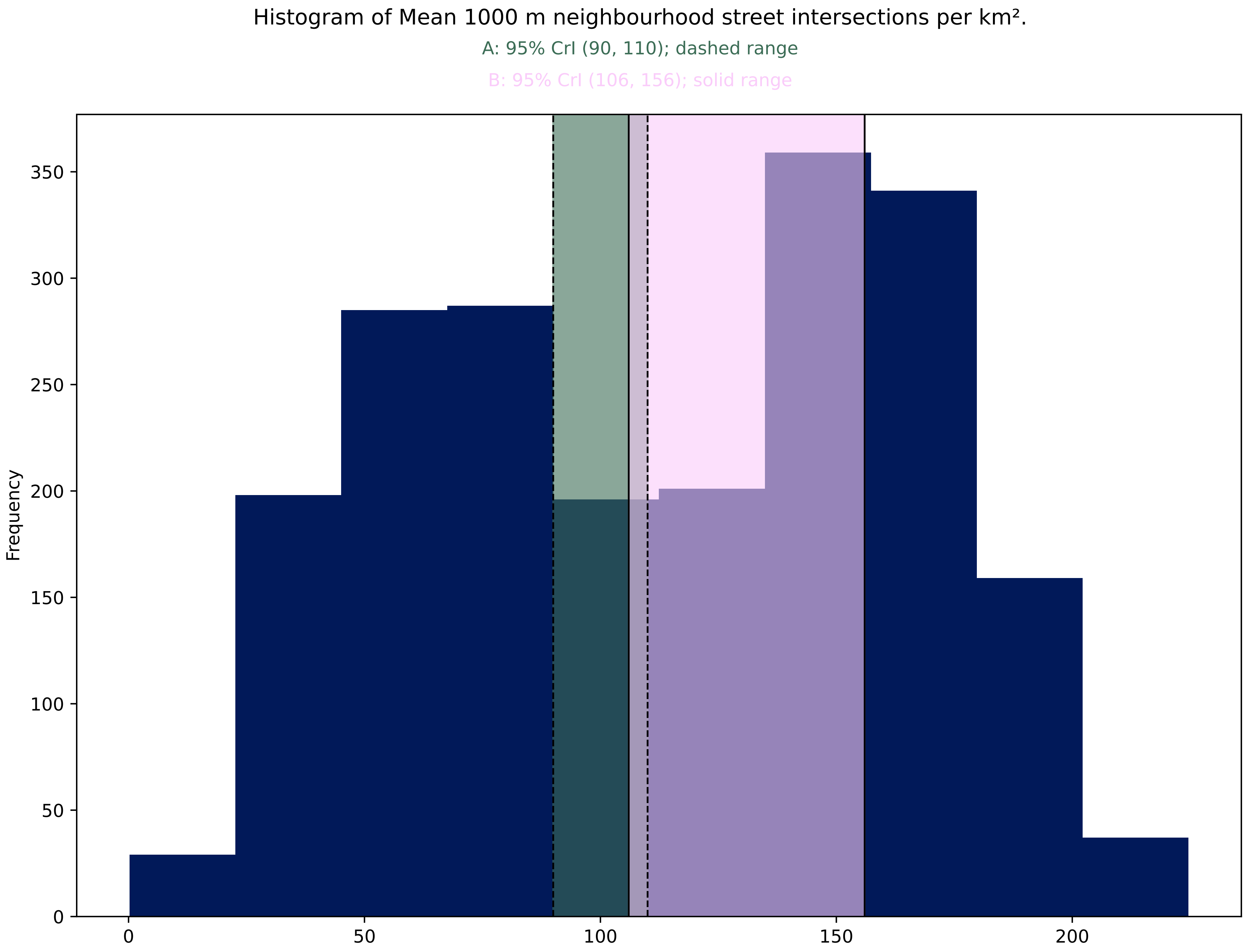
Histogram of Mean 1000 m neighbourhood street intersections per km².
A: 95% CrI (90, 110); dashed range
B: 95% CrI (106, 156); solid range
Frequency
0
50
100
150
200
250
300
350
0
50
100
150
200

Distance to nearest public transport stops (m; up to 500m)

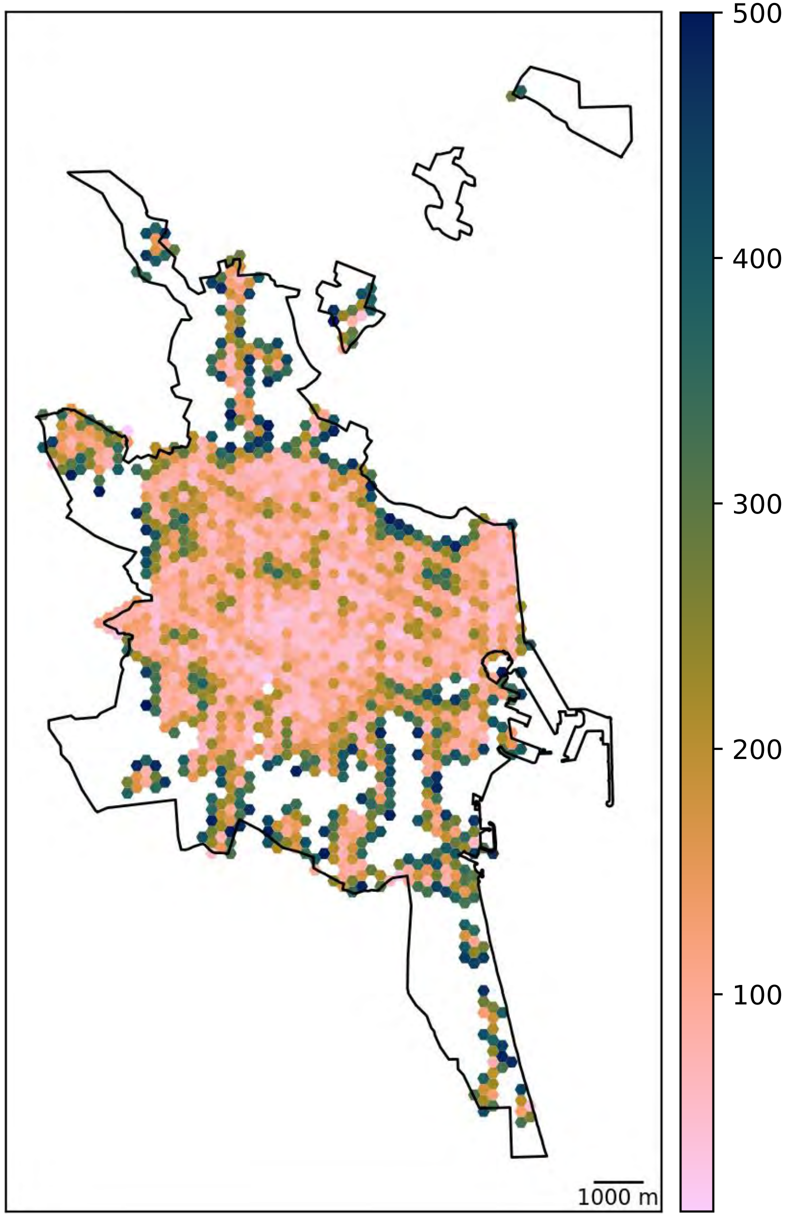

distances: Estimated Distance to nearest public transport stops (m; up to 500m) requirement for distances to destinations, measured up to a maximum distance target threshold of 500 metres

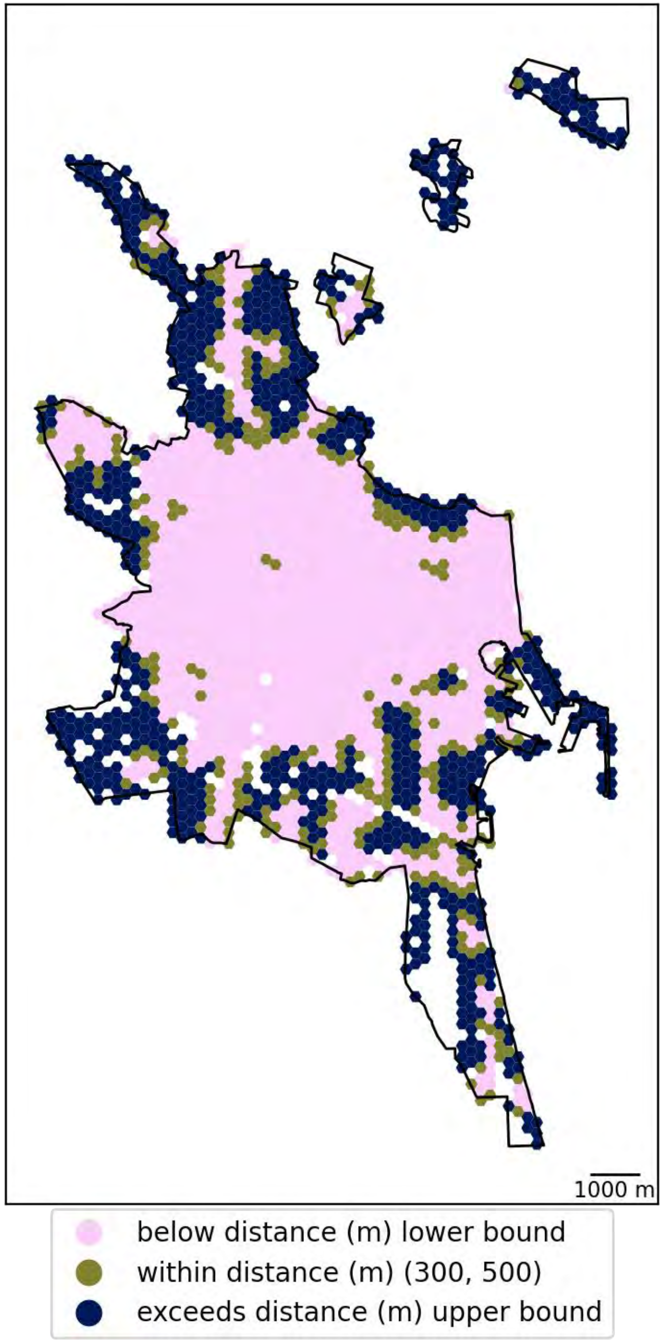

Histogram of Distance to nearest public transport stops (m; up to 500m).

distances: distance (m) (300, 500); dashed range

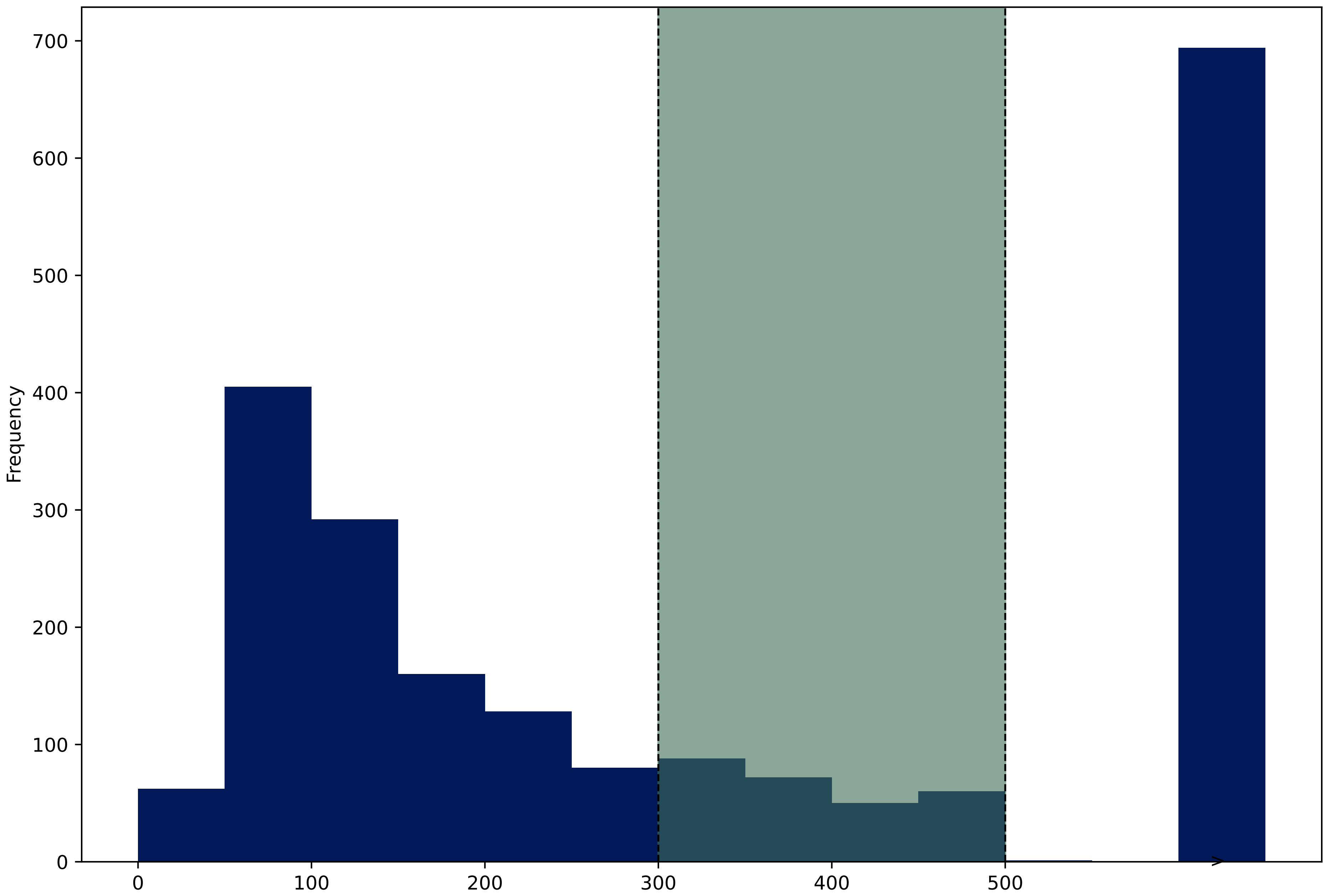

Distance to nearest park (m; up to 500m)

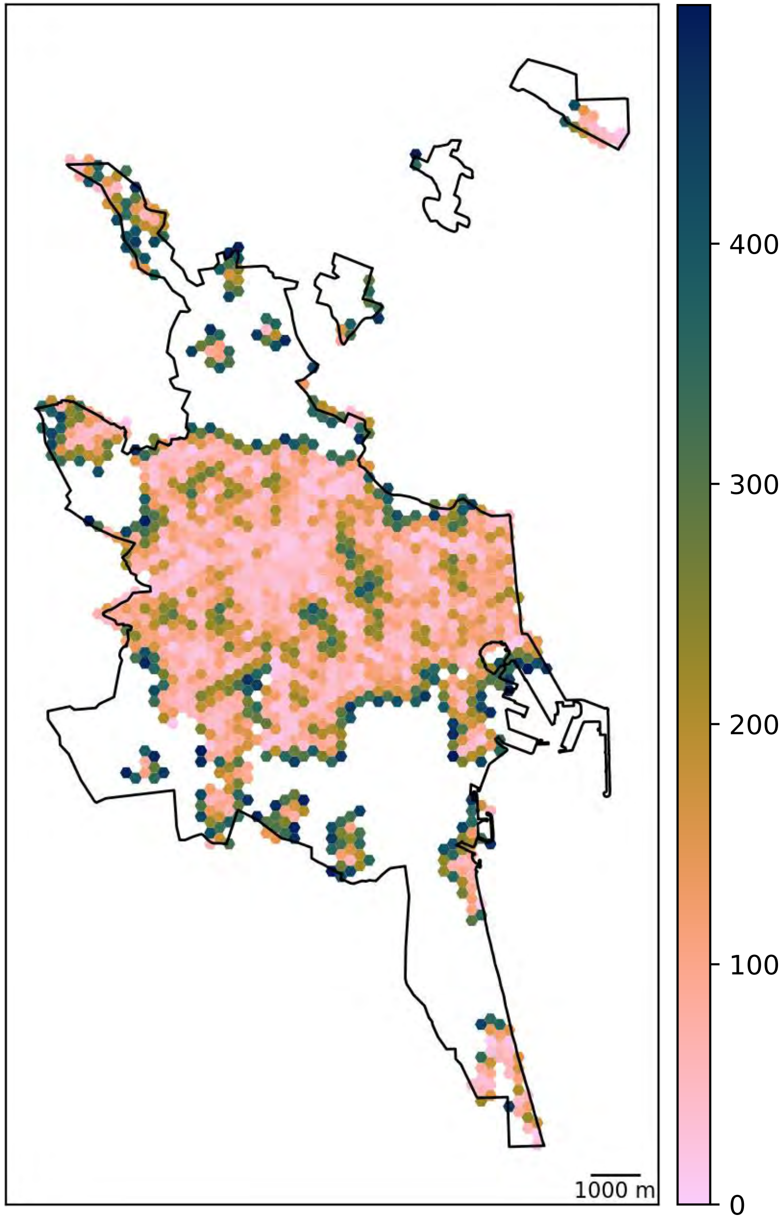

distances: Estimated Distance to nearest park (m; up to 500m) requirement for distances to destinations, measured up to a maximum distance target threshold of 500 metres

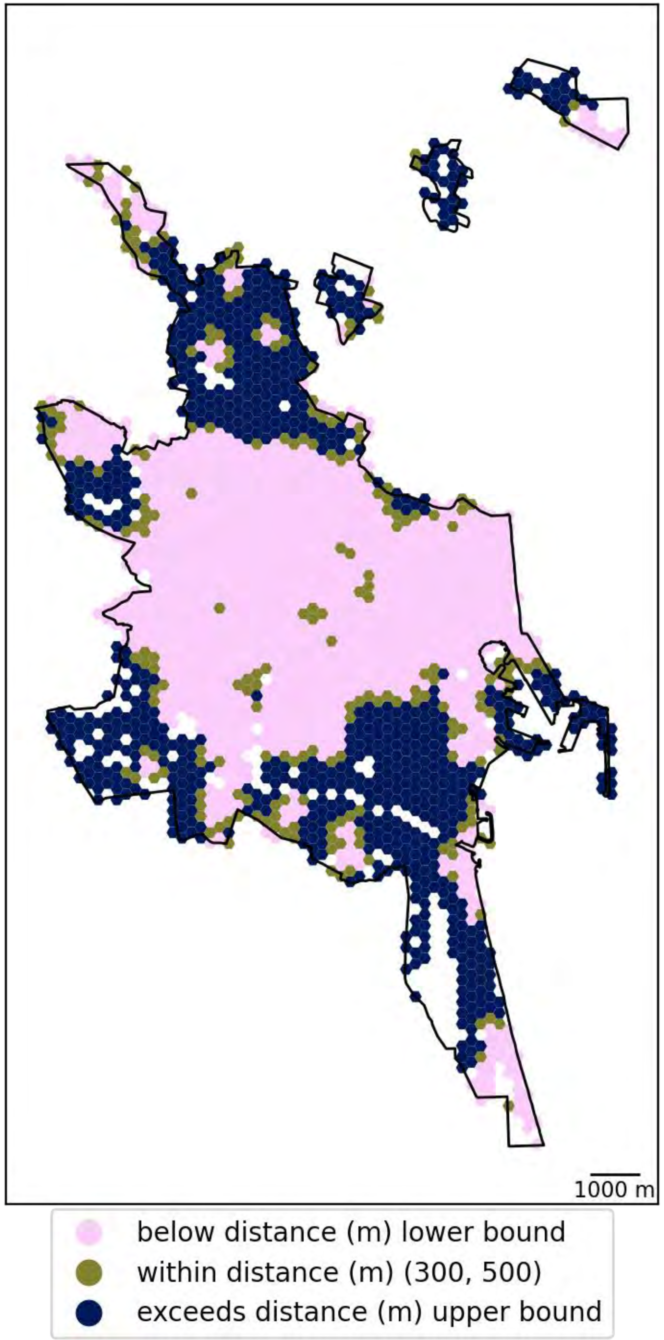

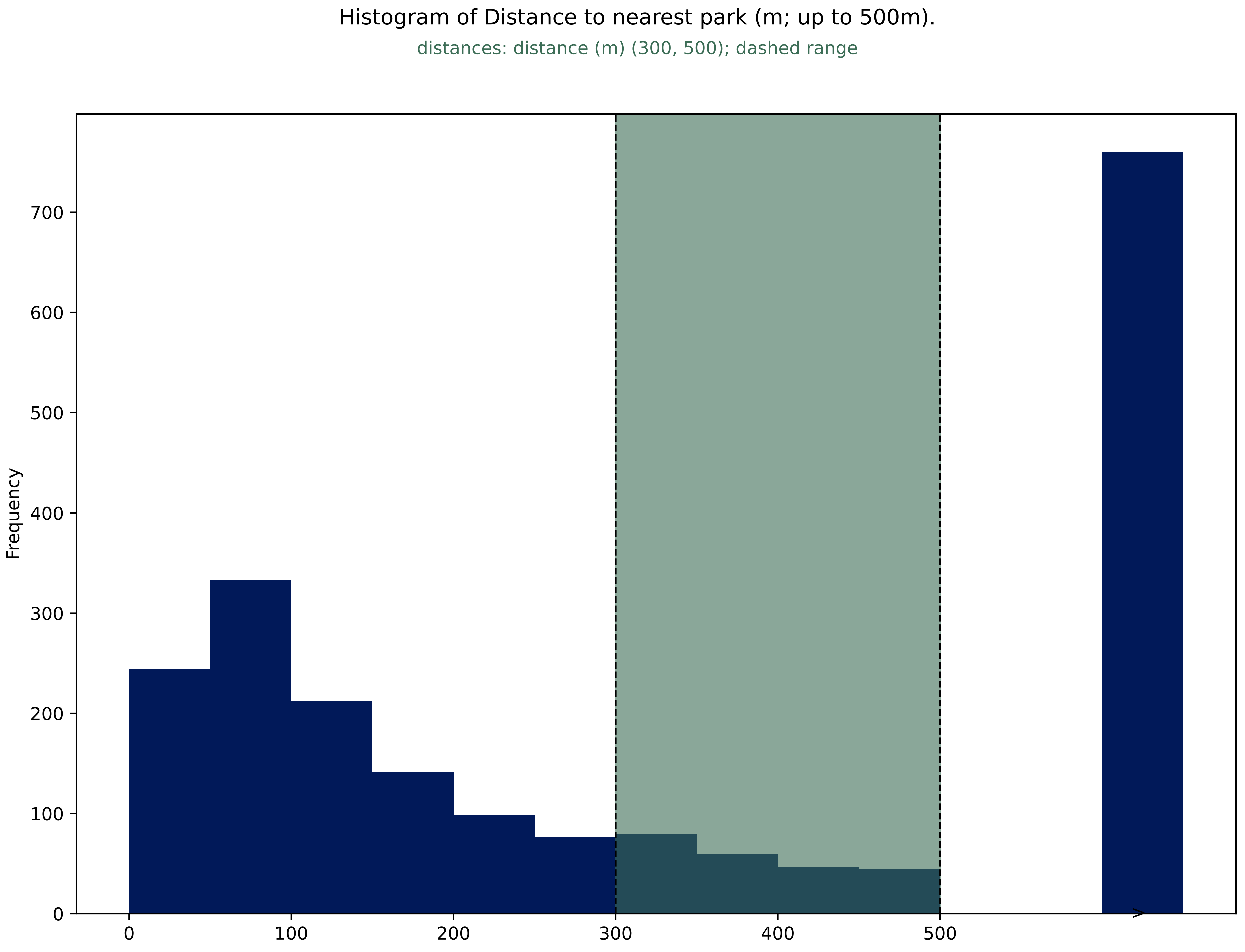
Histogram of Distance to nearest park (m; up to 500m).
distances: distance (m) (300, 500); dashed range
Frequency
0
100
200
300
400
500
600
700
0
100
200
300
400
500
>

Europe, Spain, Vic

Satellite imagery of urban study region (Bing)

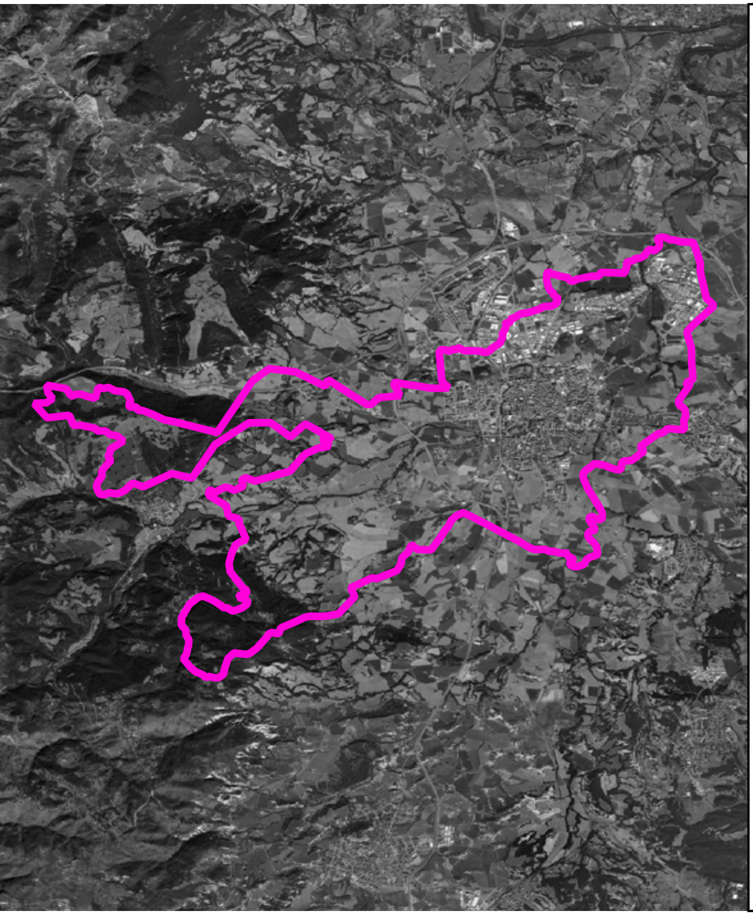

Walkability, relative to city

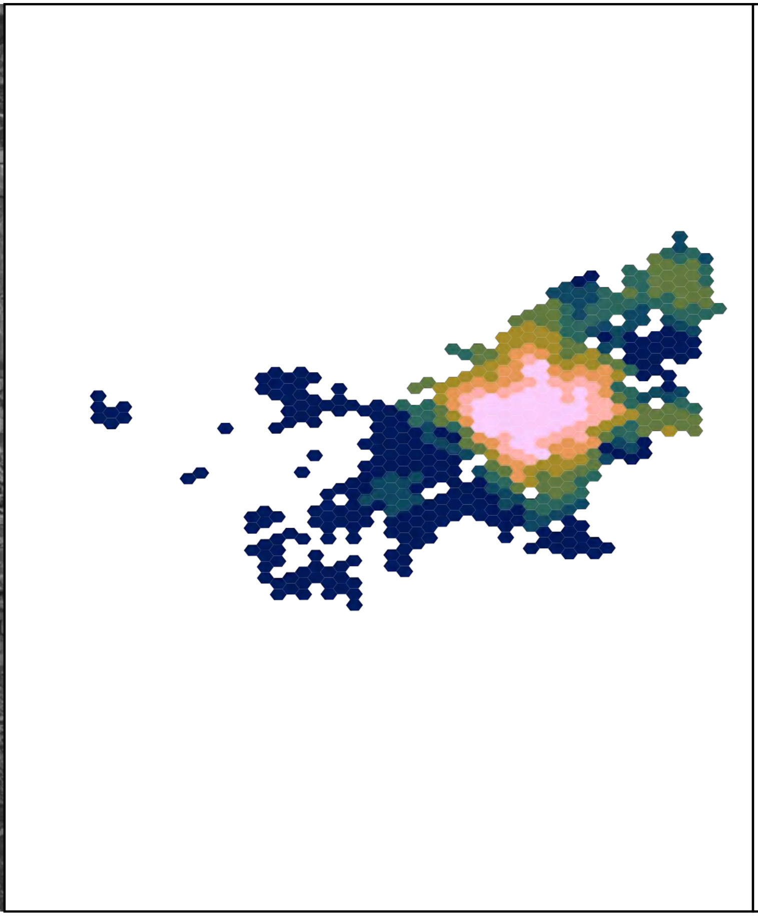

Walkability, relative to 25 global cities

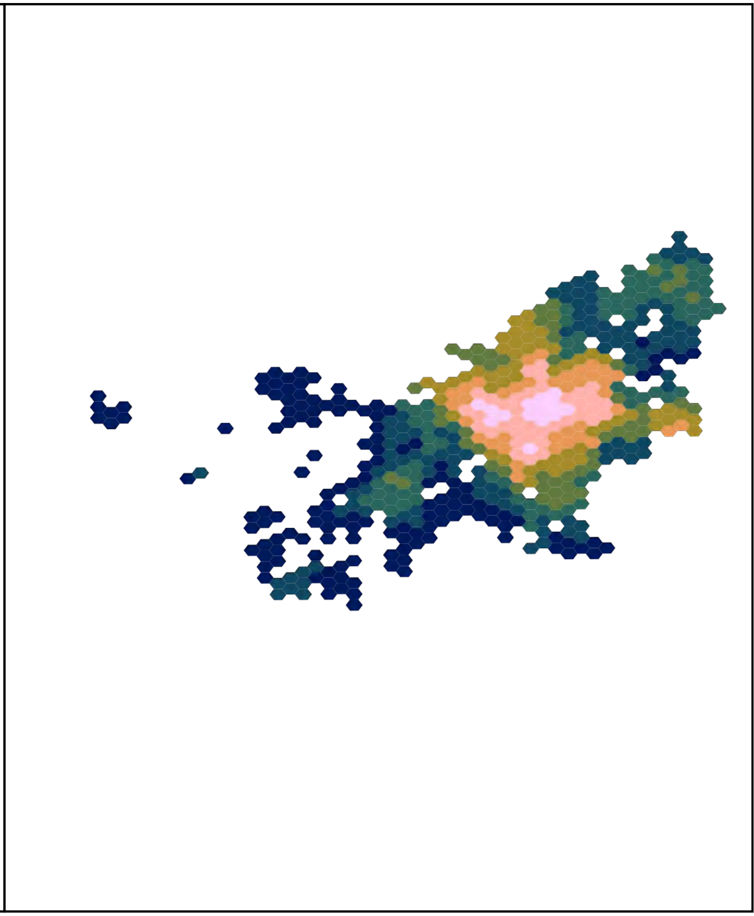

Urban boundary

0 4 8 km

Walkability score

- <-3
- -3 to -2
- -2 to -1
- -1 to 0
- 0 to 1
- 1 to 2
- 2 to 3
- ≥3

Walkability relative to all cities by component variables (2D histograms), and overall (histogram)

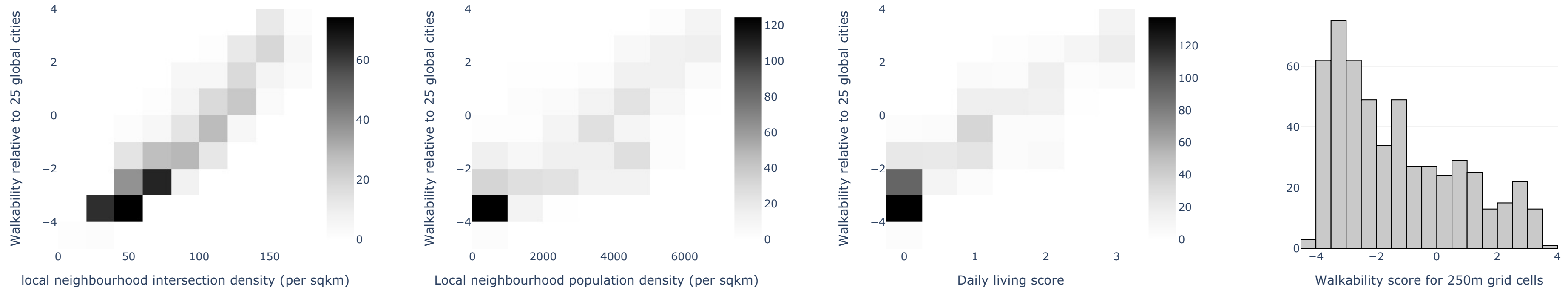

Mean 1000 m neighbourhood population per km²

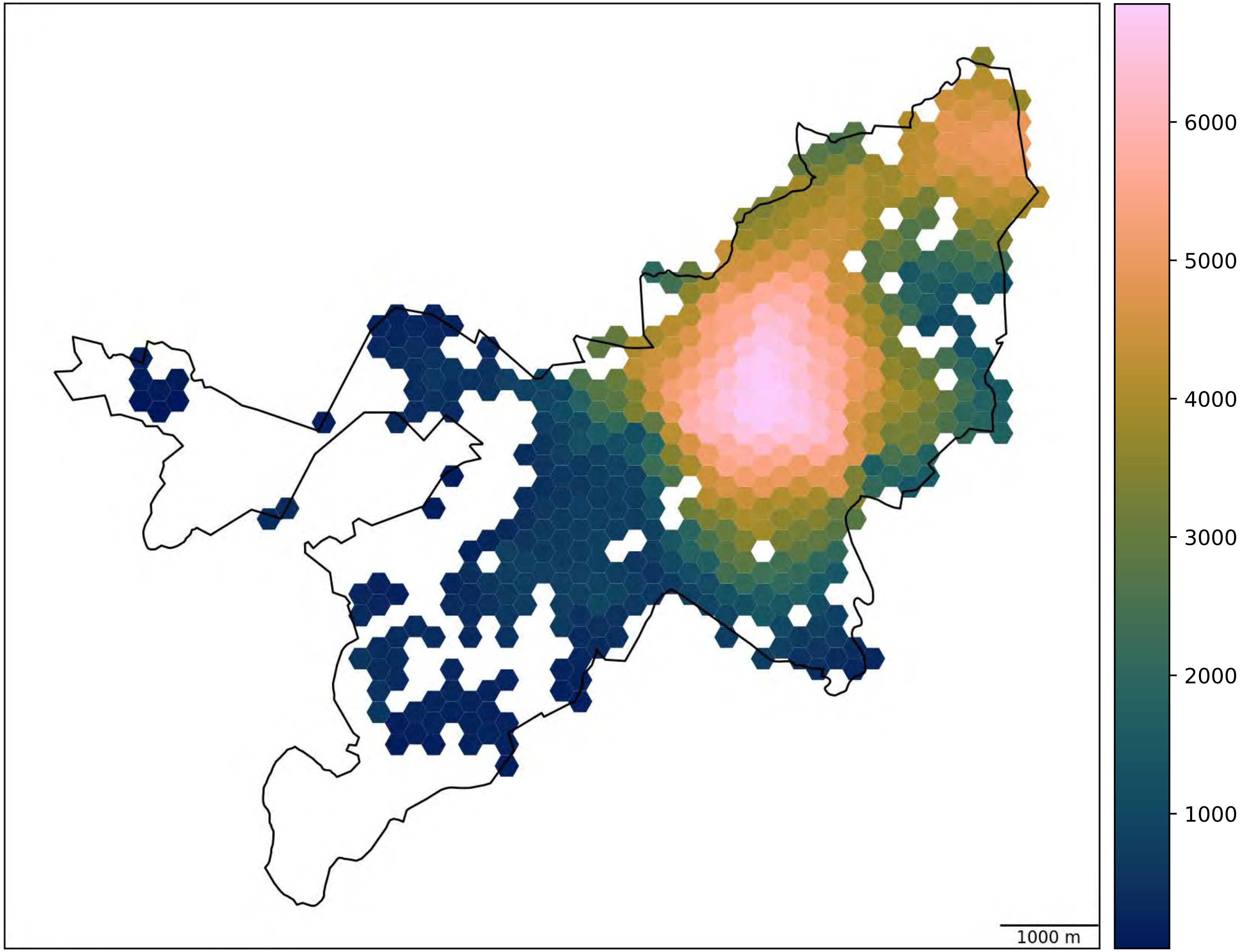

A: Estimated Mean 1000 m neighbourhood population per km² requirement for ≥80% probability of engaging in walking for transport

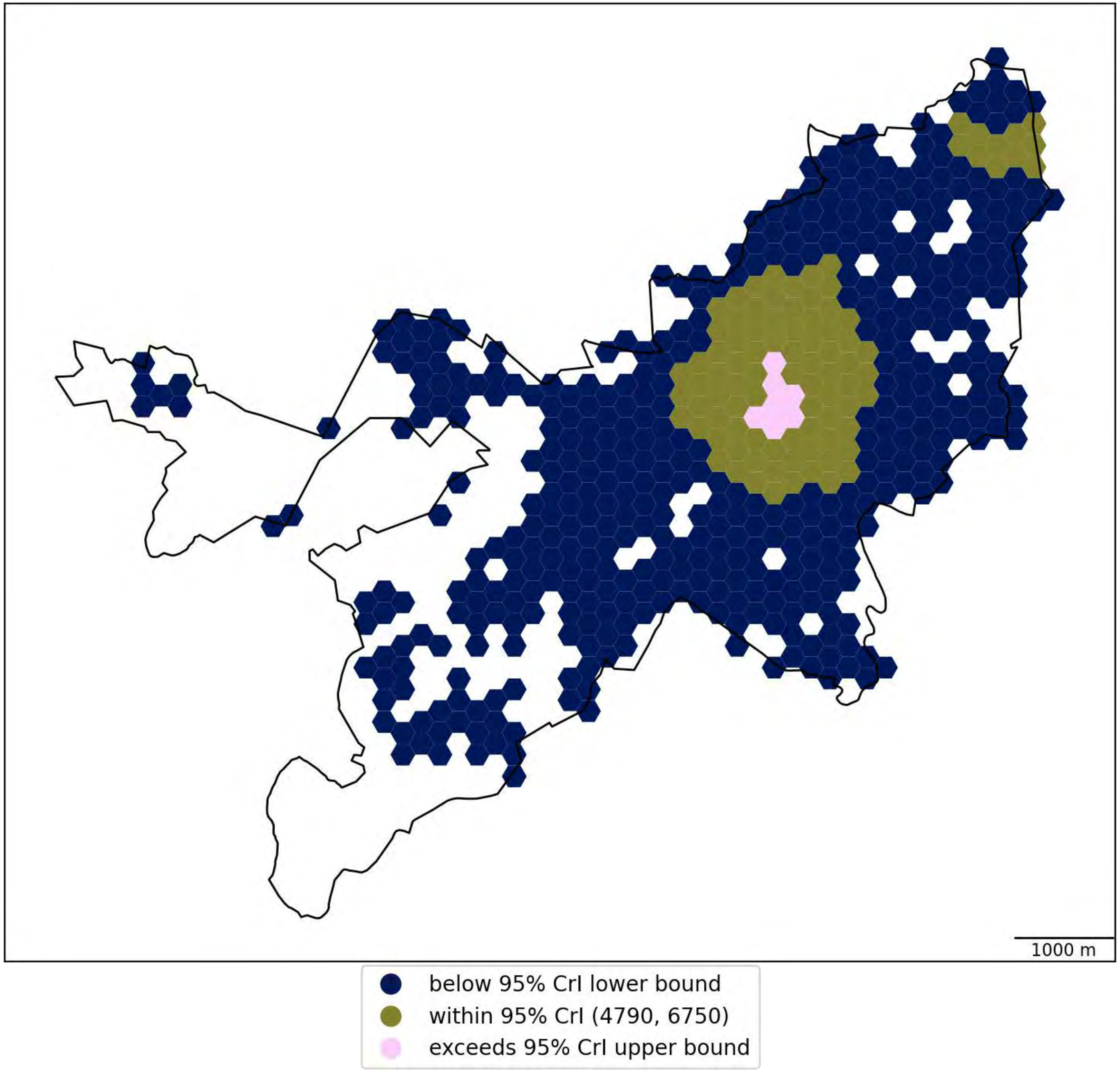

B: Estimated Mean 1000 m neighbourhood population per km² requirement for reaching the WHO's target of a ≥15% relative reduction in insufficient physical activity through walking

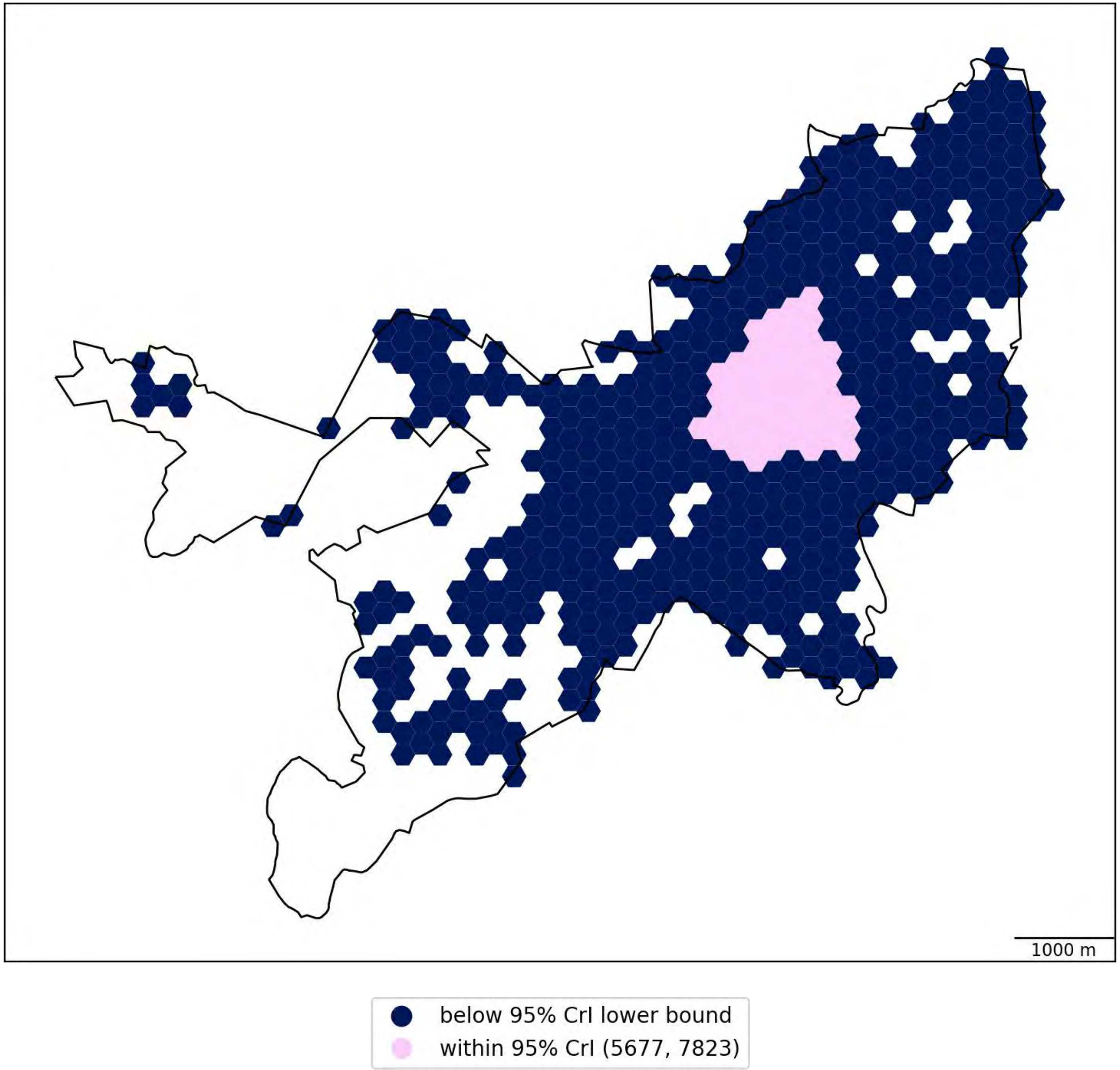

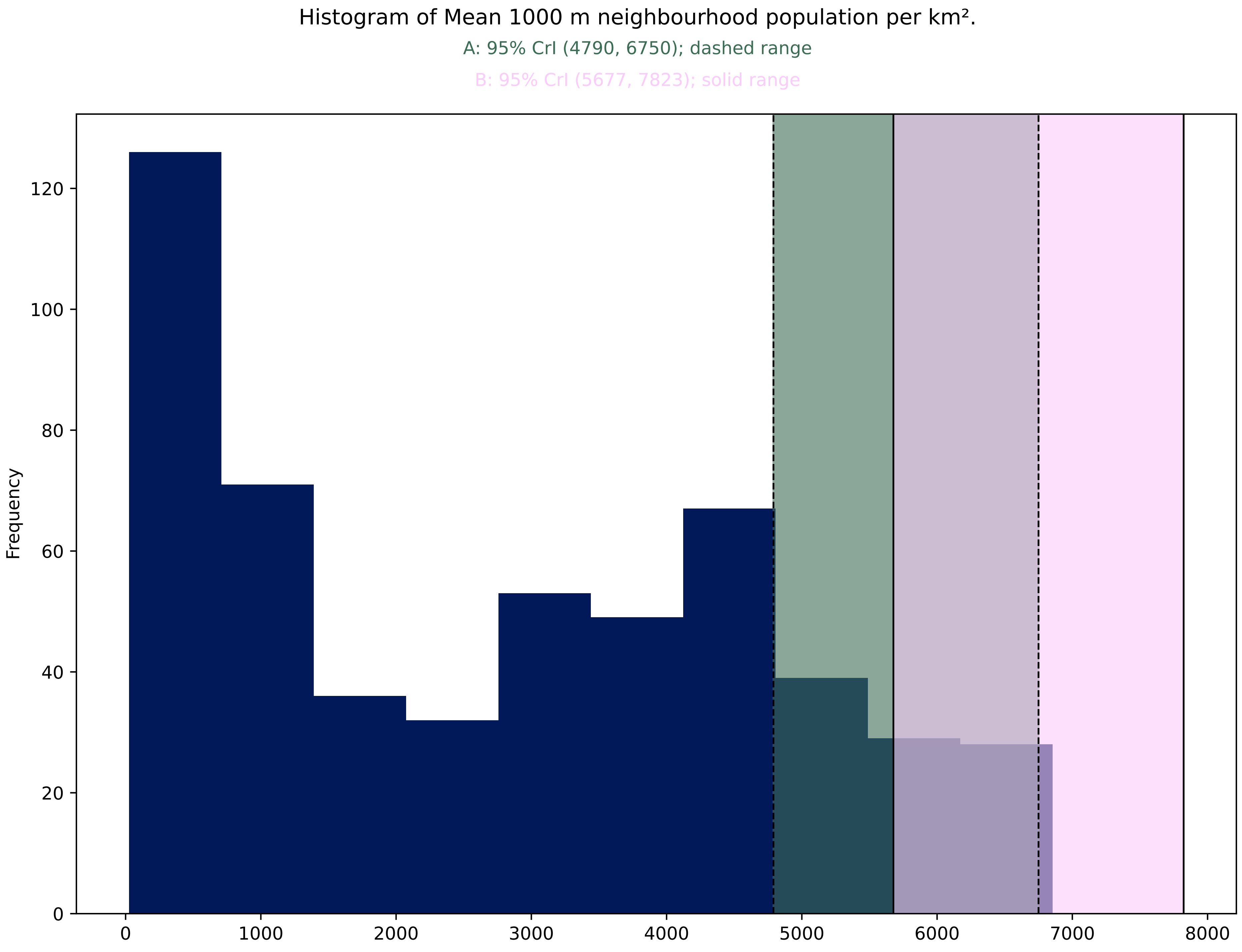

Histogram of Mean 1000 m neighbourhood population per km².
A: 95% CrI (4790, 6750); dashed range
B: 95% CrI (5677, 7823); solid range
Frequency
0
20
40
60
80
100
120
0
1000
2000
3000
4000
5000
6000
7000
8000

Mean 1000 m neighbourhood street intersections per km²

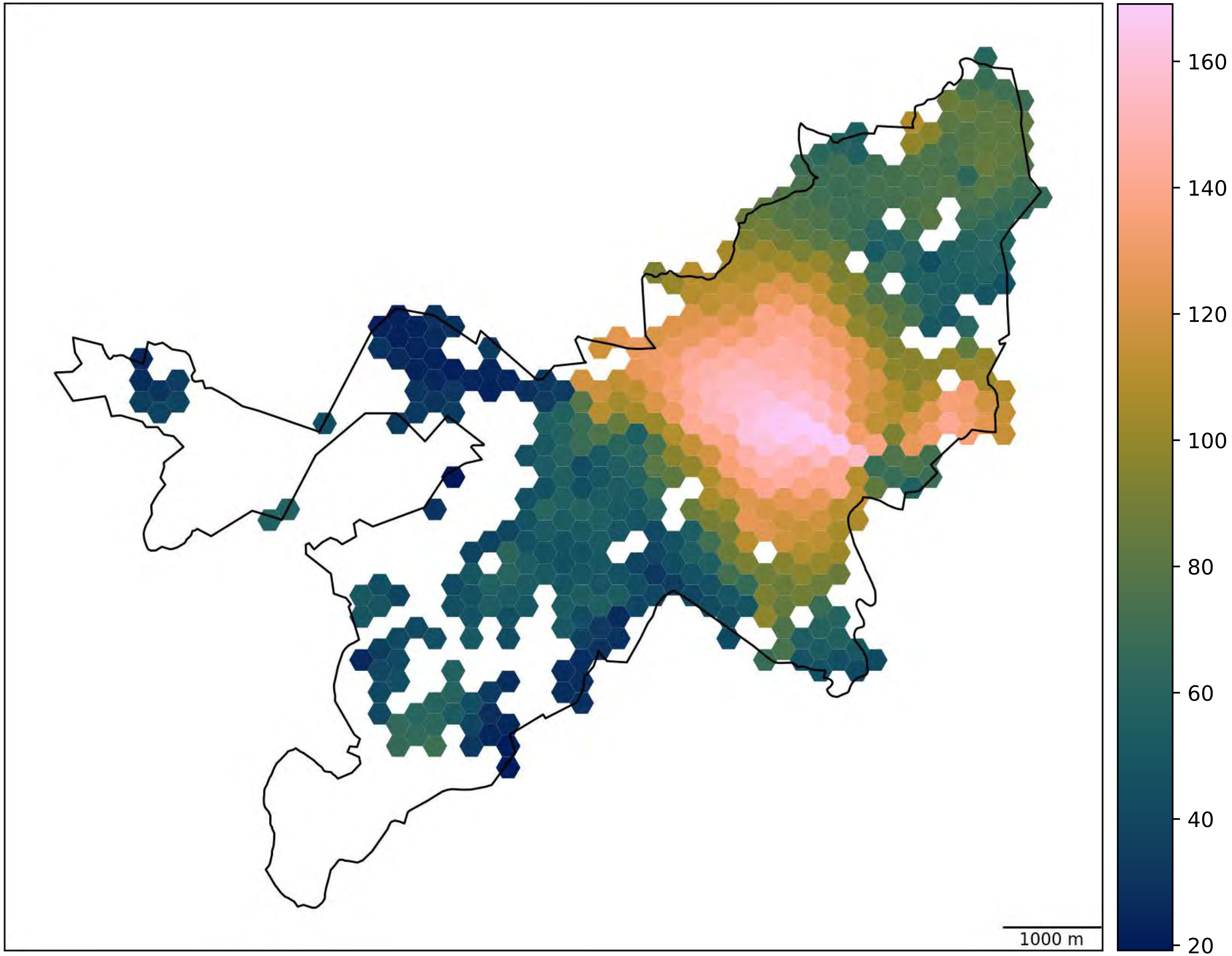

A: Estimated Mean 1000 m neighbourhood street intersections per km² requirement for ≥80% probability of engaging in walking for transport

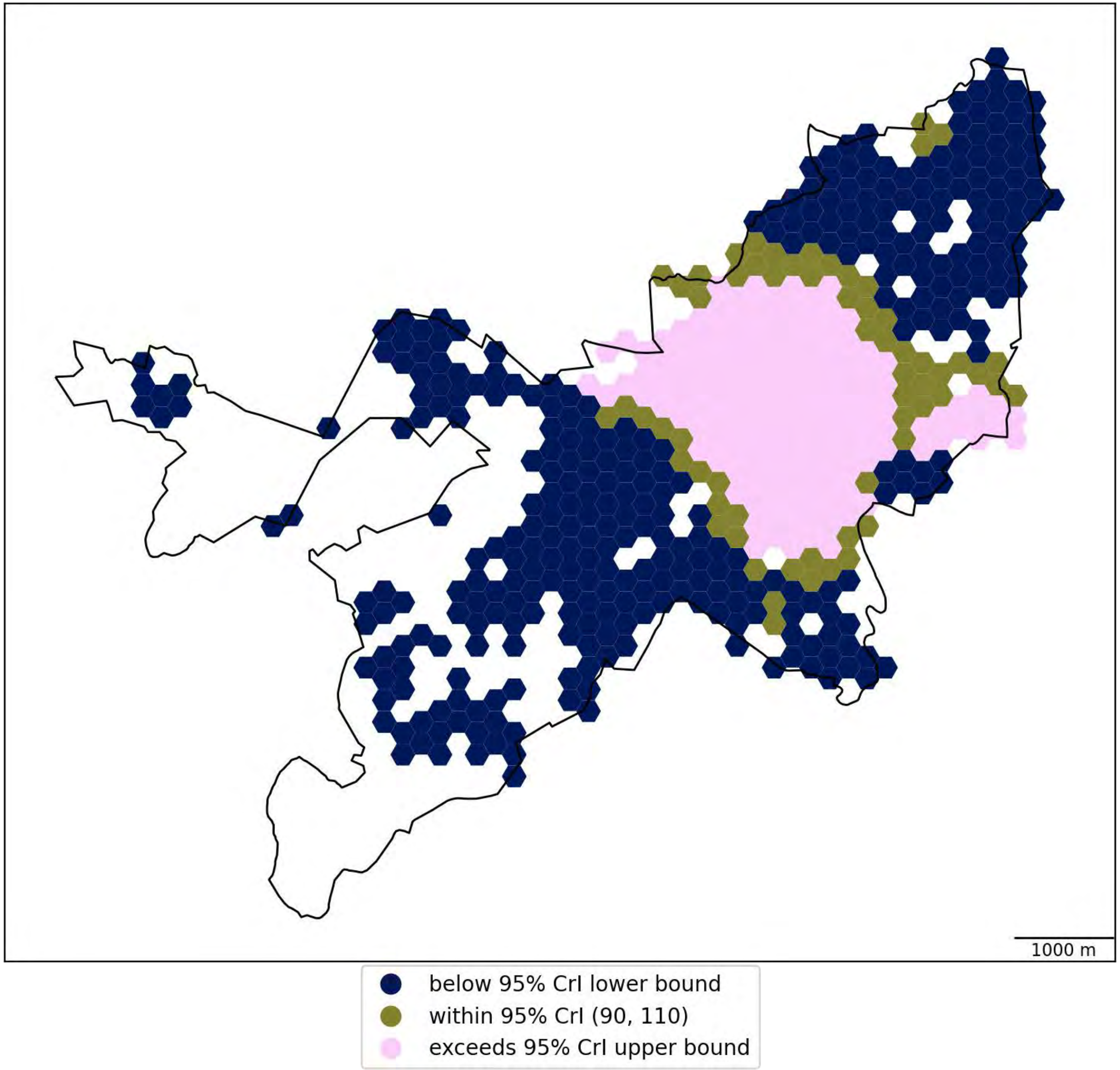

B: Estimated Mean 1000 m neighbourhood street intersections per km² requirement for reaching the WHO's target of a ≥15% relative reduction in insufficient physical activity through walking

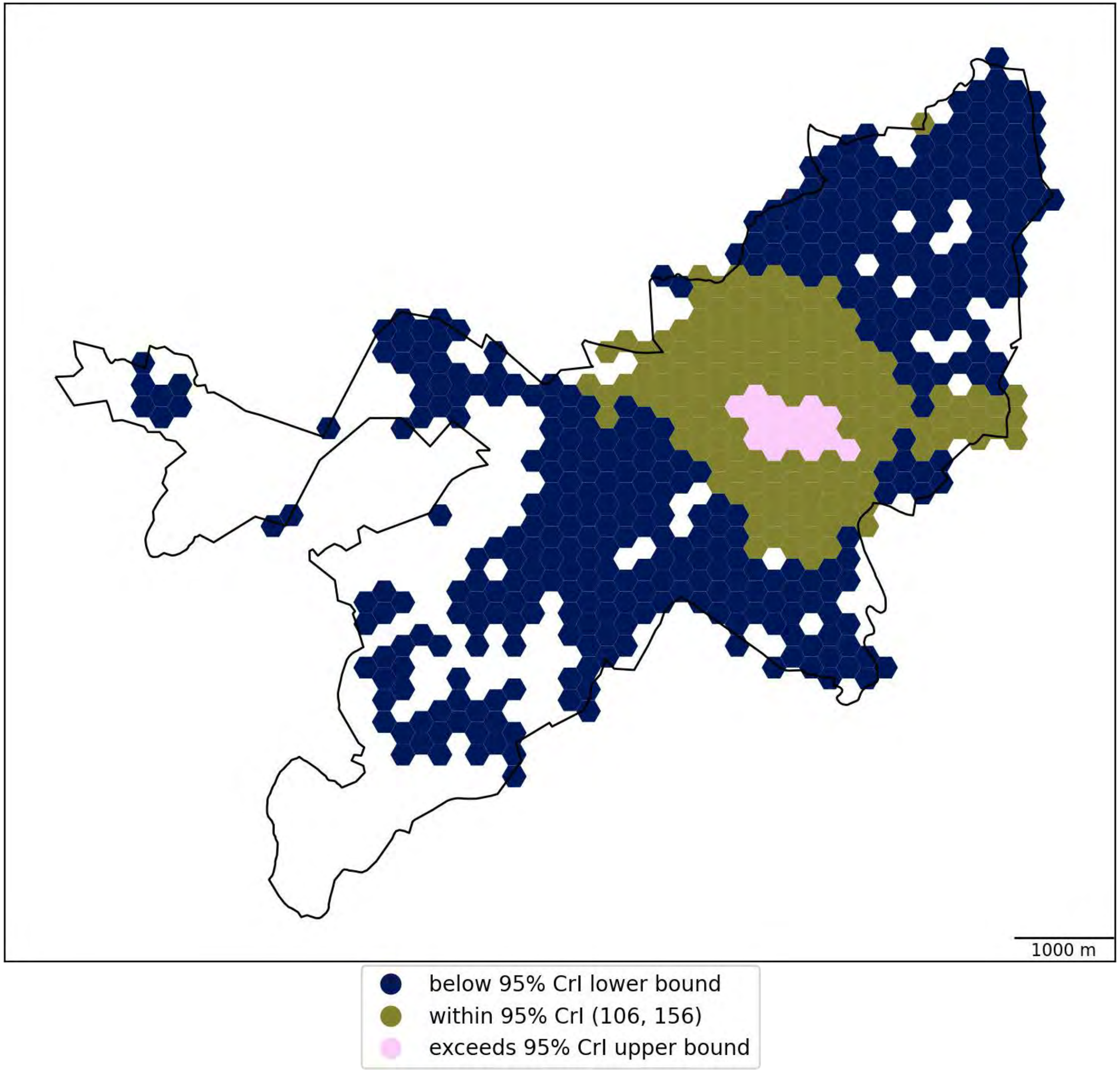

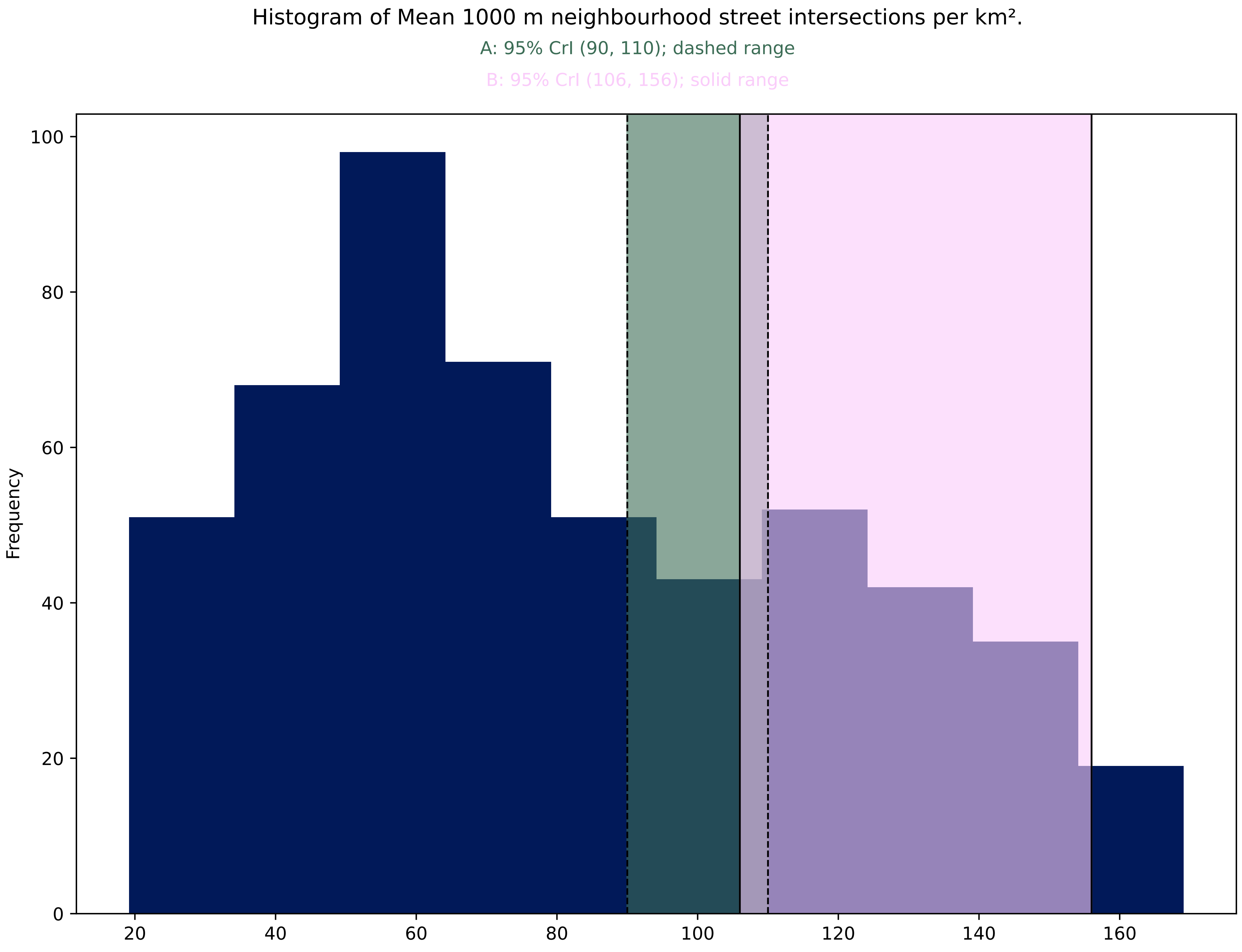

Histogram of Mean 1000 m neighbourhood street intersections per km².
A: 95% CrI (90, 110); dashed range
B: 95% CrI (106, 156); solid range
Frequency
0
20
40
60
80
100
20
40
60
80
100
120
140
160

Distance to nearest public transport stops (m; up to 500m)

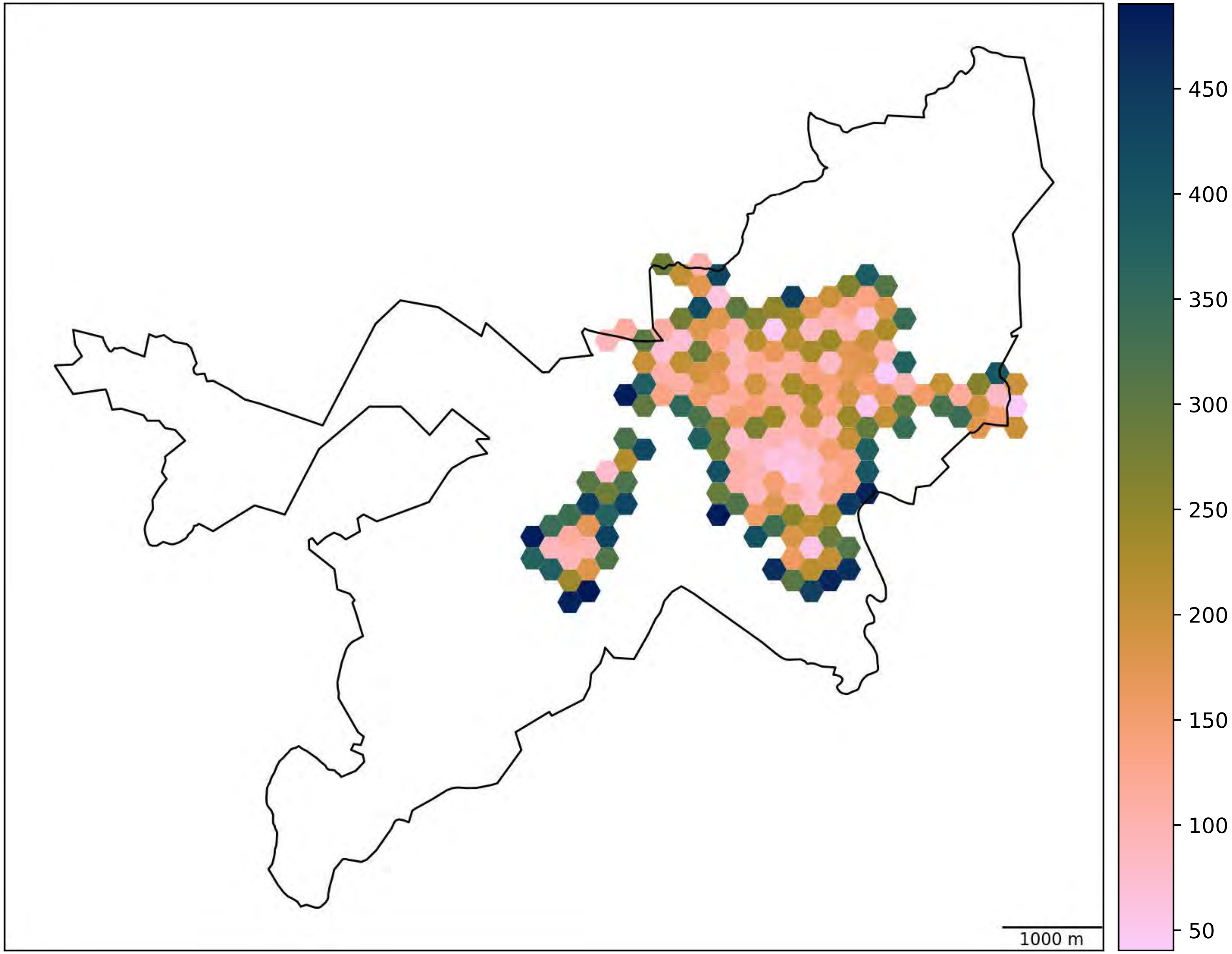

distances: Estimated Distance to nearest public transport stops (m; up to 500m) requirement for distances to destinations, measured up to a maximum distance target threshold of 500 metres

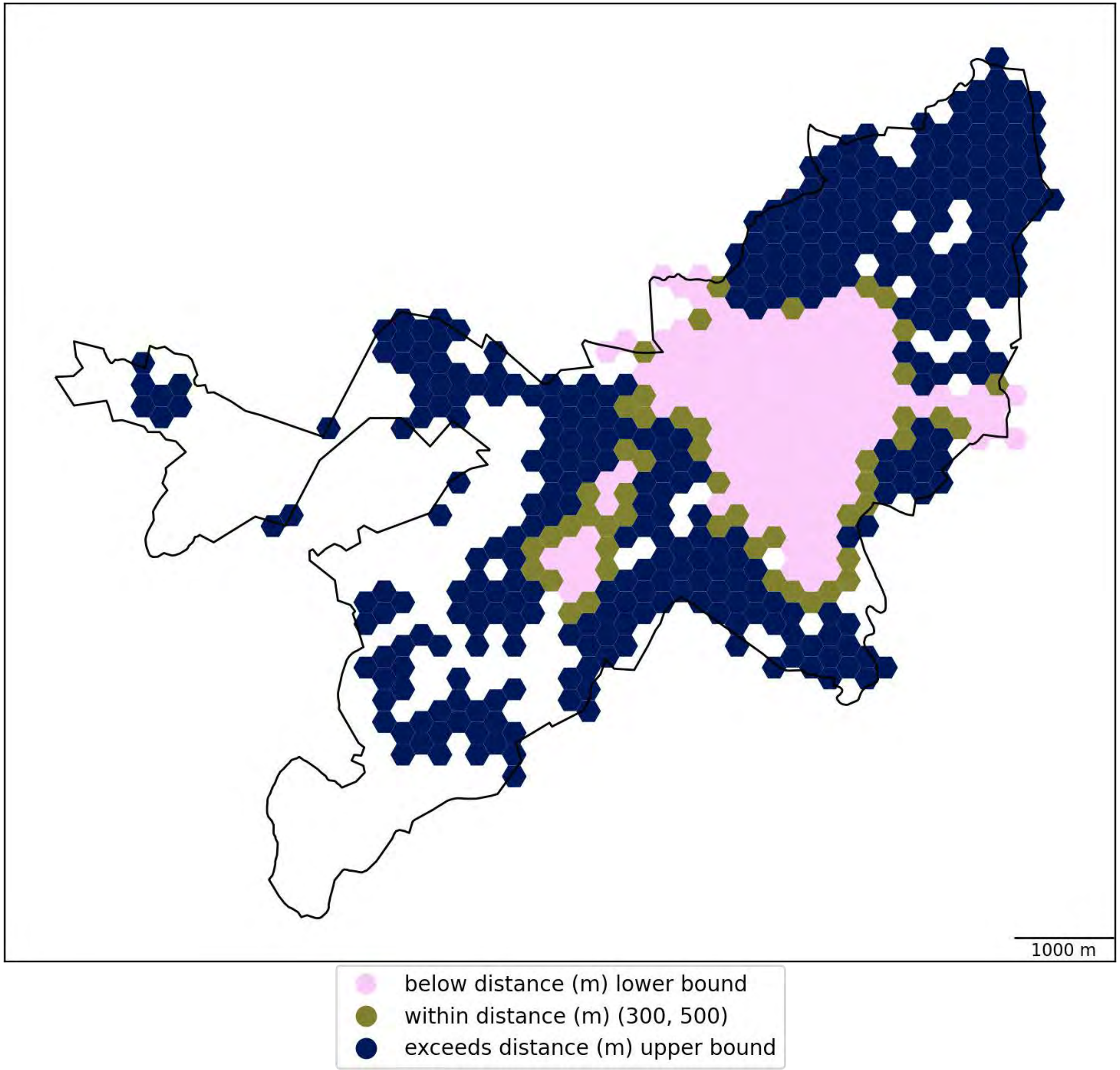

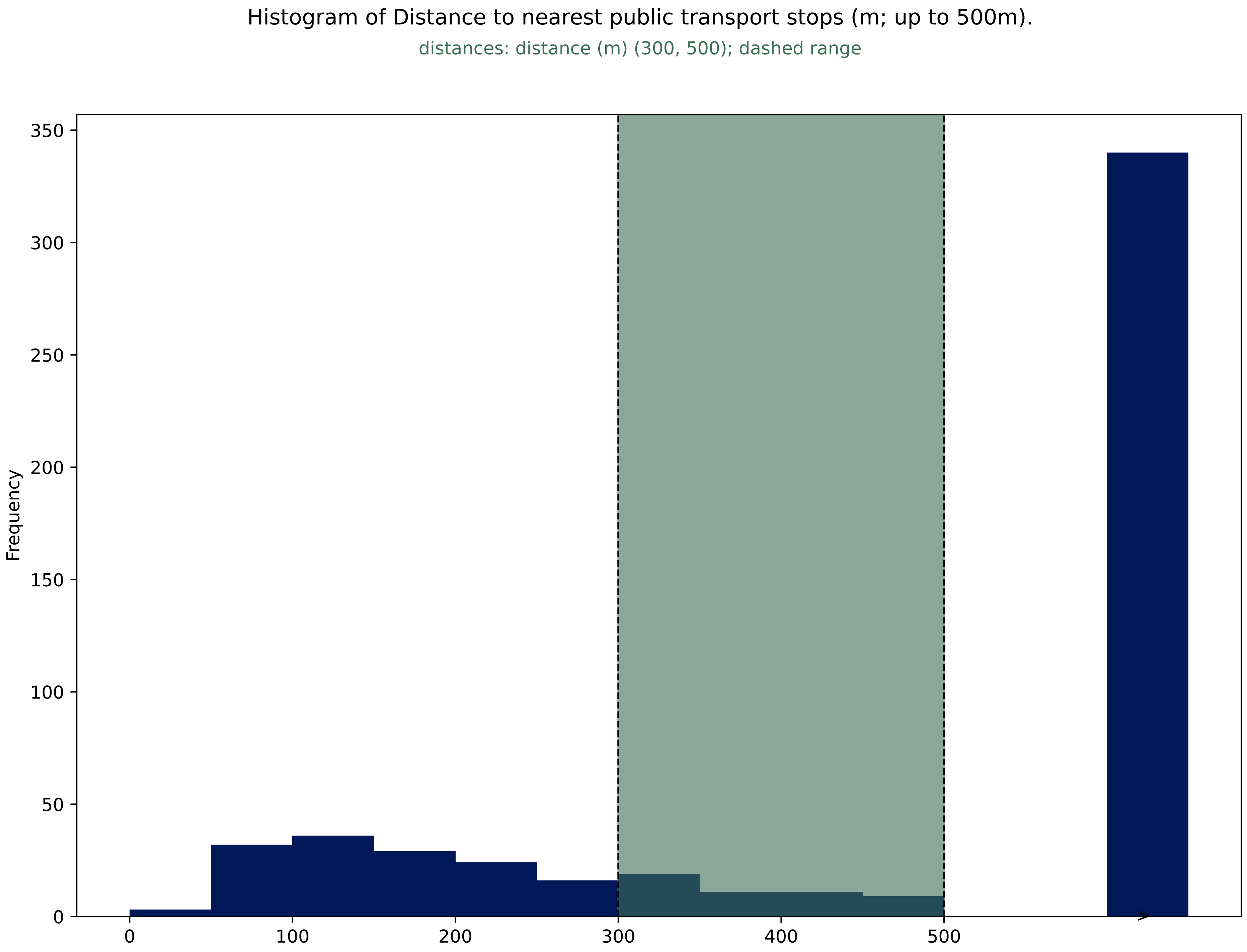

Histogram of Distance to nearest public transport stops (m; up to 500m).
distances: distance (m) (300, 500); dashed range
Frequency
0
50
100
150
200
250
300
350
0
100
200
300
400
500
>

Distance to nearest park (m; up to 500m)

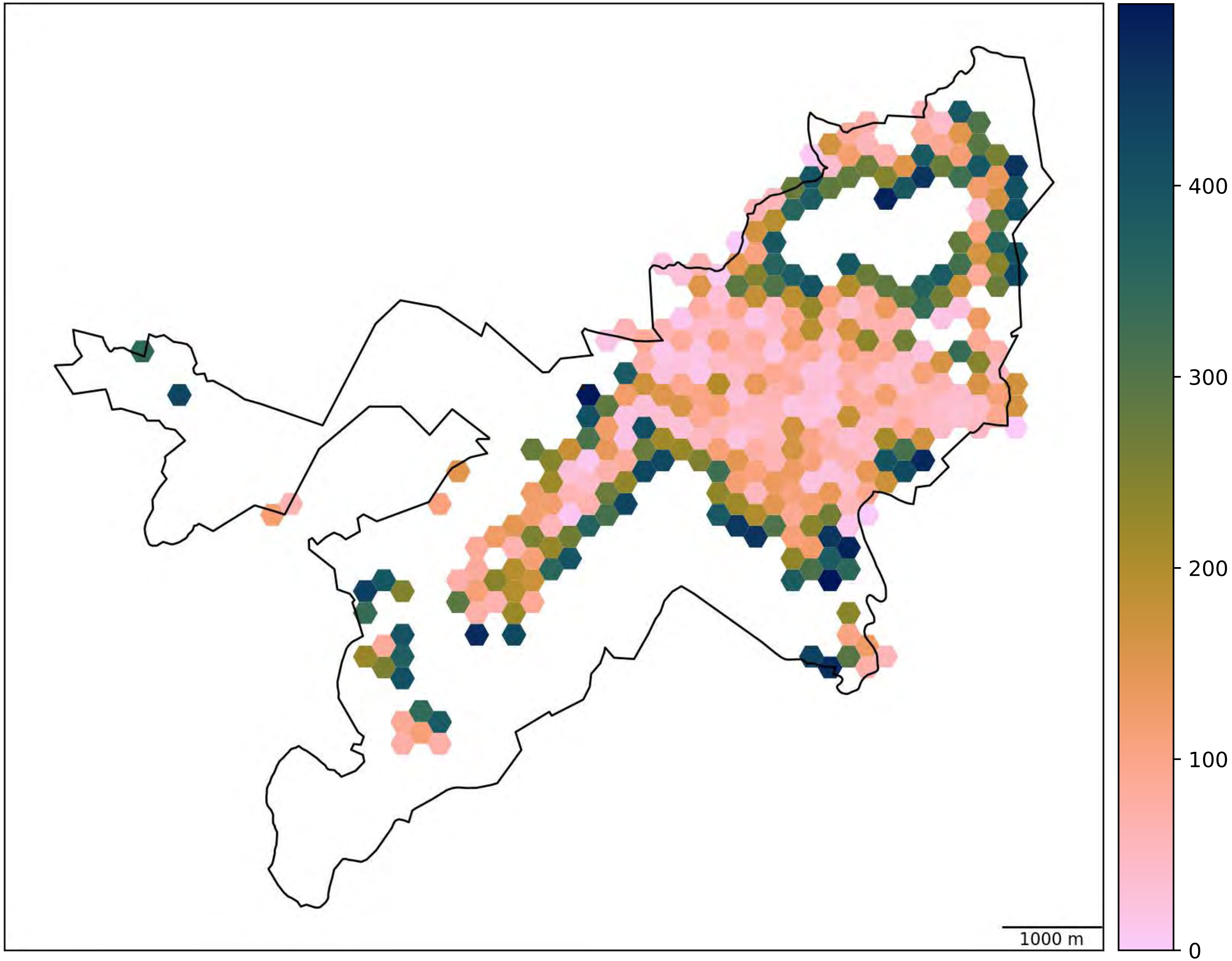

distances: Estimated Distance to nearest park (m; up to 500m) requirement for distances to destinations, measured up to a maximum distance target threshold of 500 metres

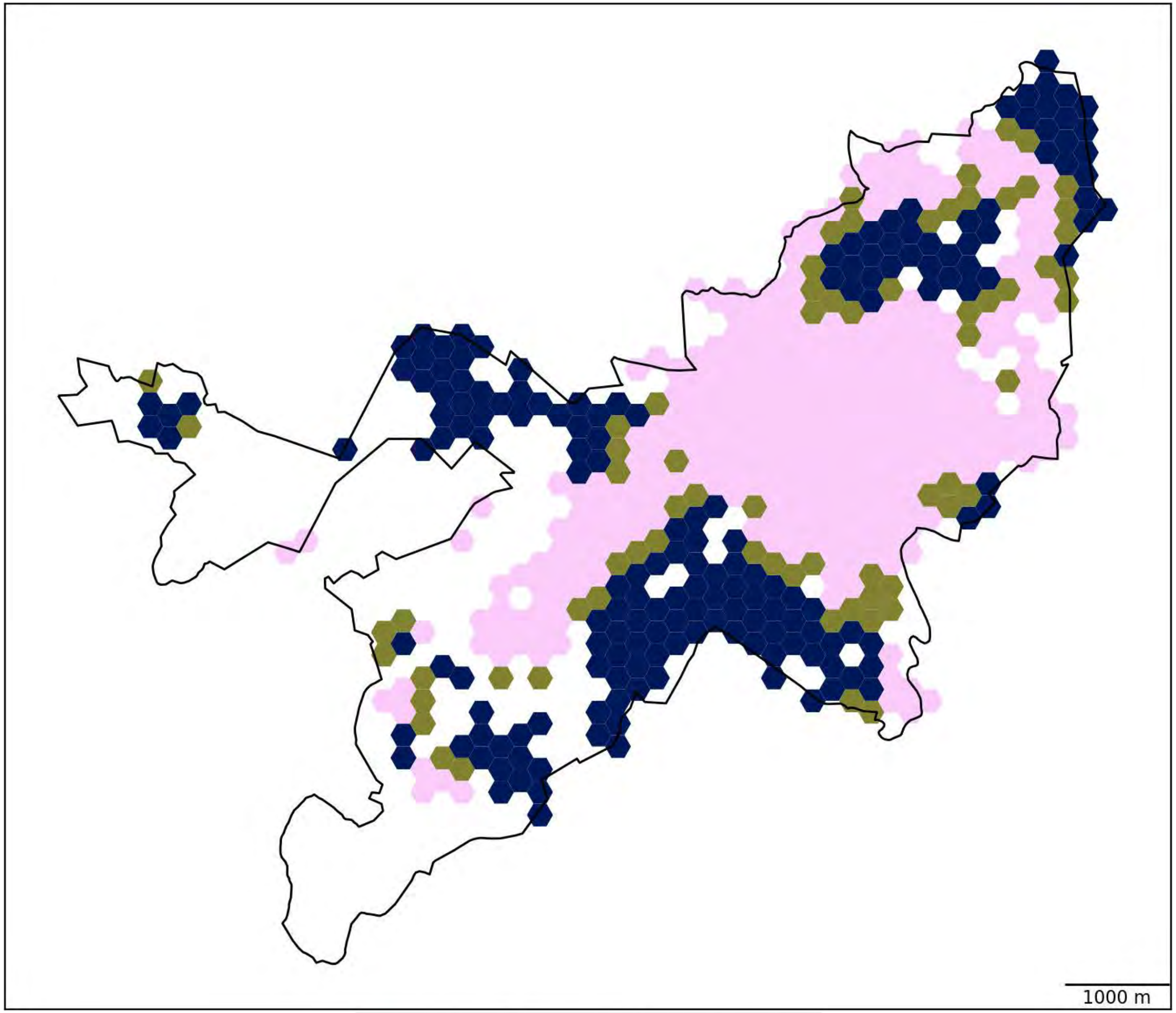

below distance (m) lower bound
within distance (m) (300, 500)
exceeds distance (m) upper bound

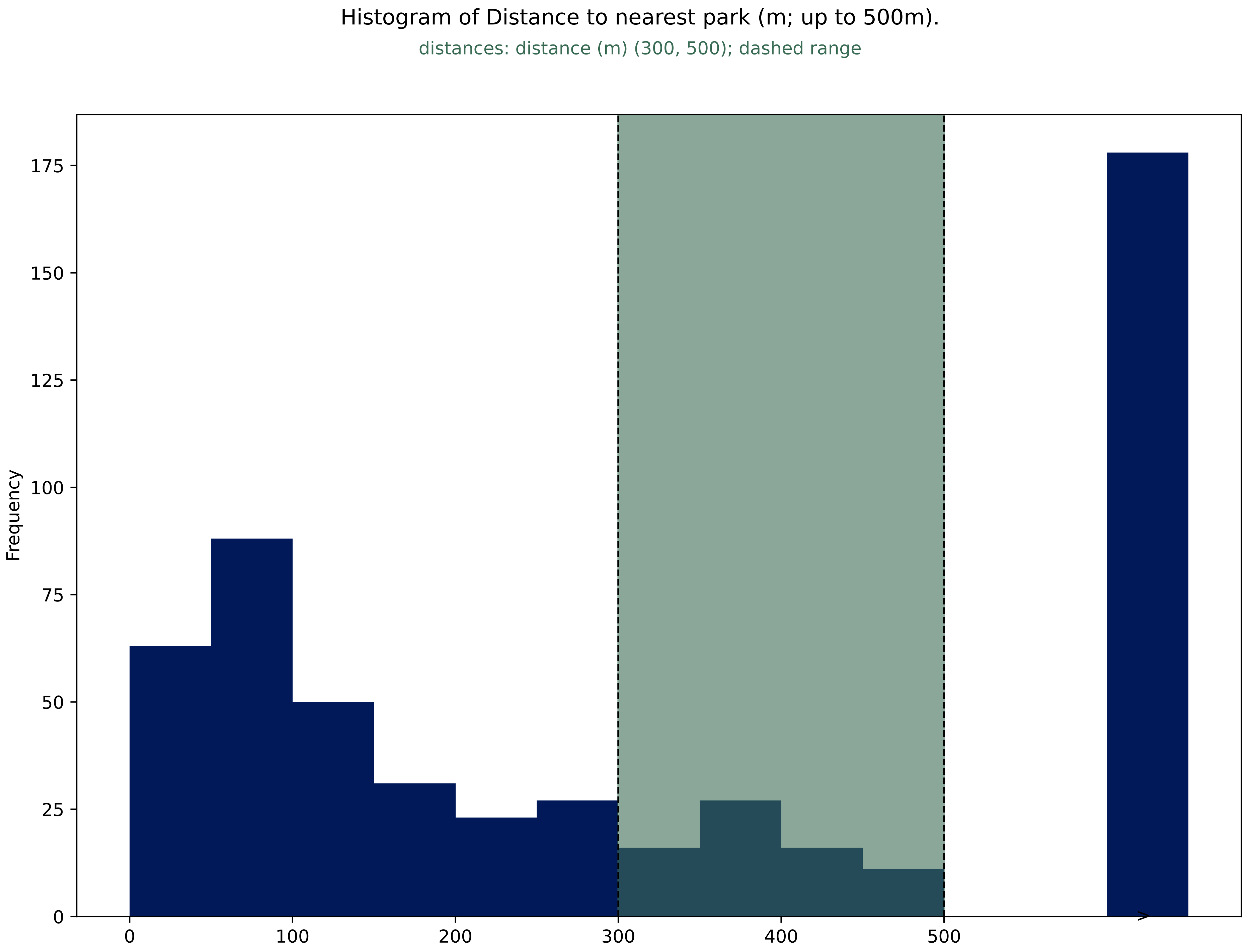
Histogram of Distance to nearest park (m; up to 500m).
distances: distance (m) (300, 500); dashed range
Frequency
0
25
50
75
100
125
150
175
0
100
200
300
400
500
>

Europe, Switzerland, Bern

Satellite imagery of urban study region (Bing)

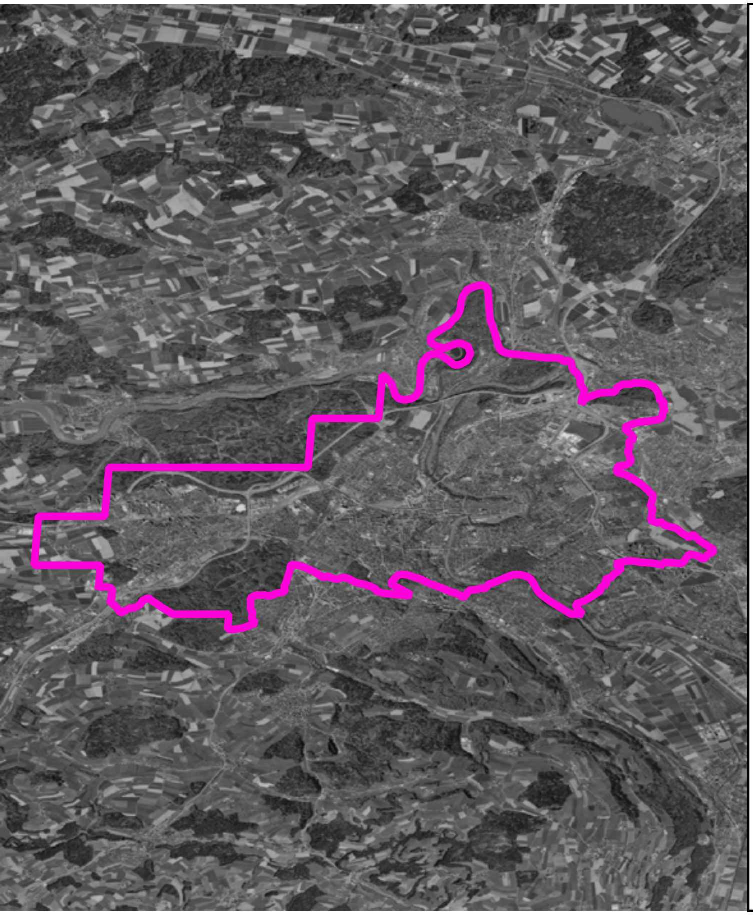

Walkability, relative to city

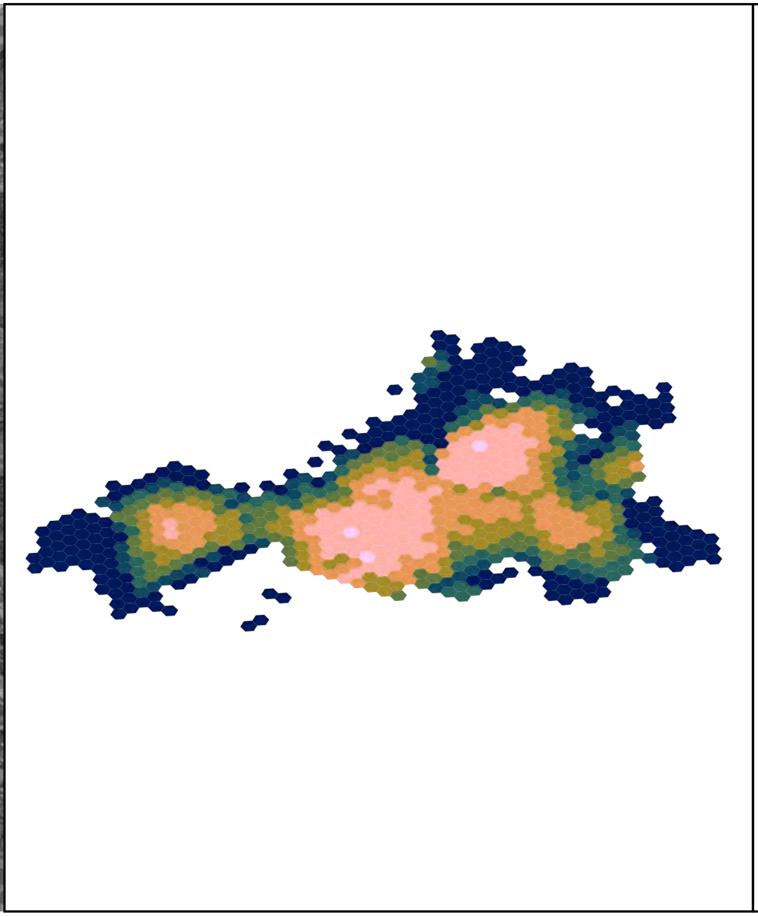

Walkability, relative to 25 global cities

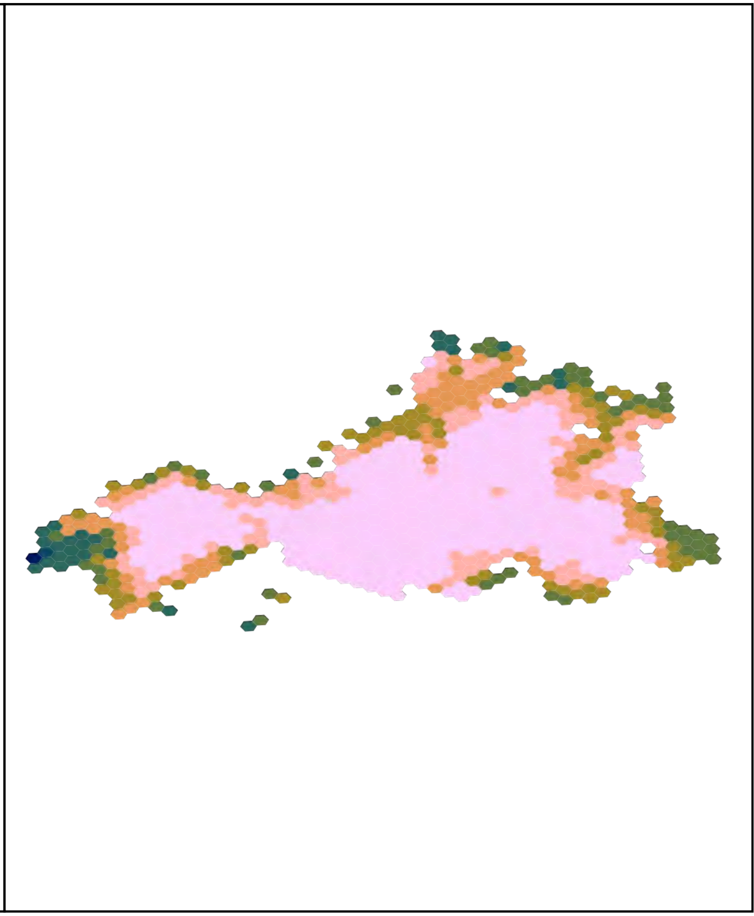

Urban boundary

0 4 8 km

Walkability score

- <-3
- -3 to -2
- -2 to -1
- -1 to 0
- 0 to 1
- 1 to 2
- 2 to 3
- ≥3

Walkability relative to all cities by component variables (2D histograms), and overall (histogram)

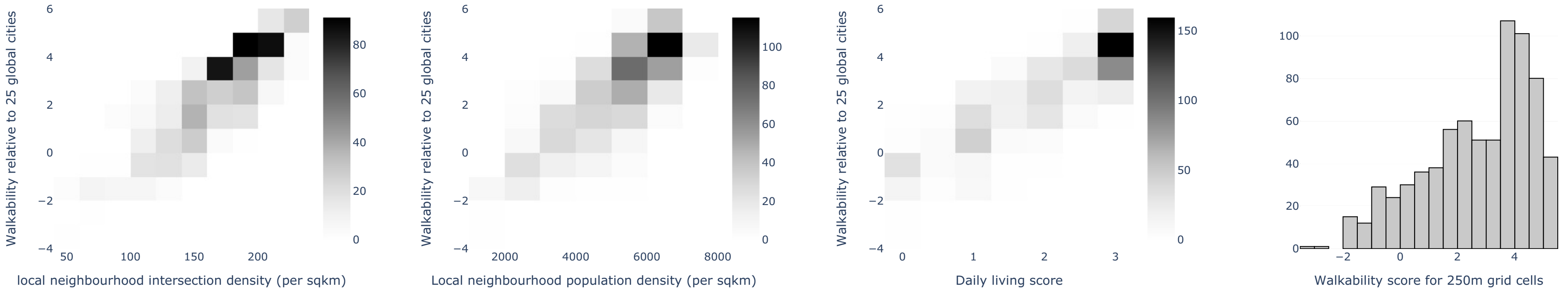

Mean 1000 m neighbourhood population per km²

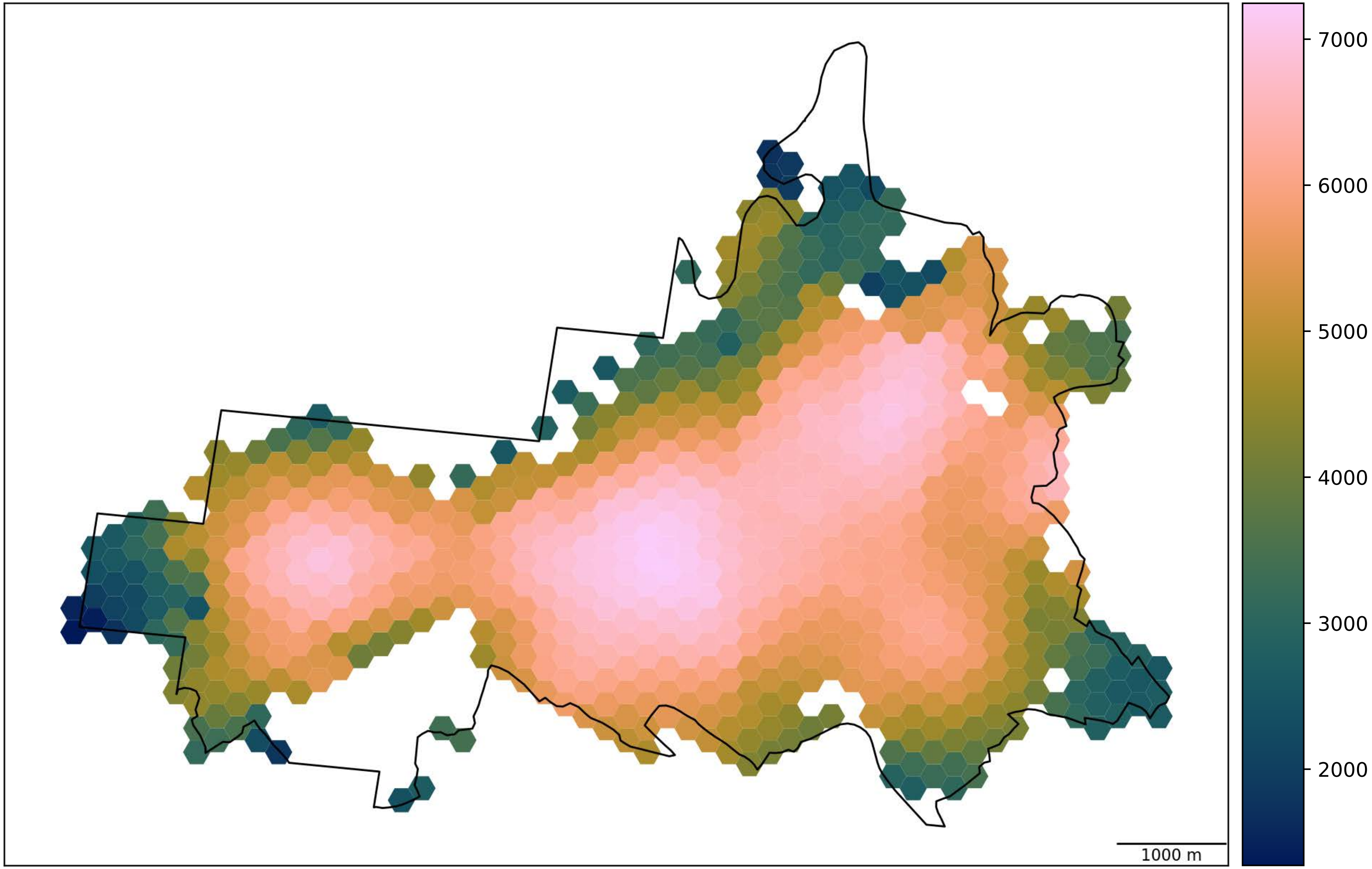

A: Estimated Mean 1000 m neighbourhood population per km² requirement for ≥80% probability of engaging in walking for transport

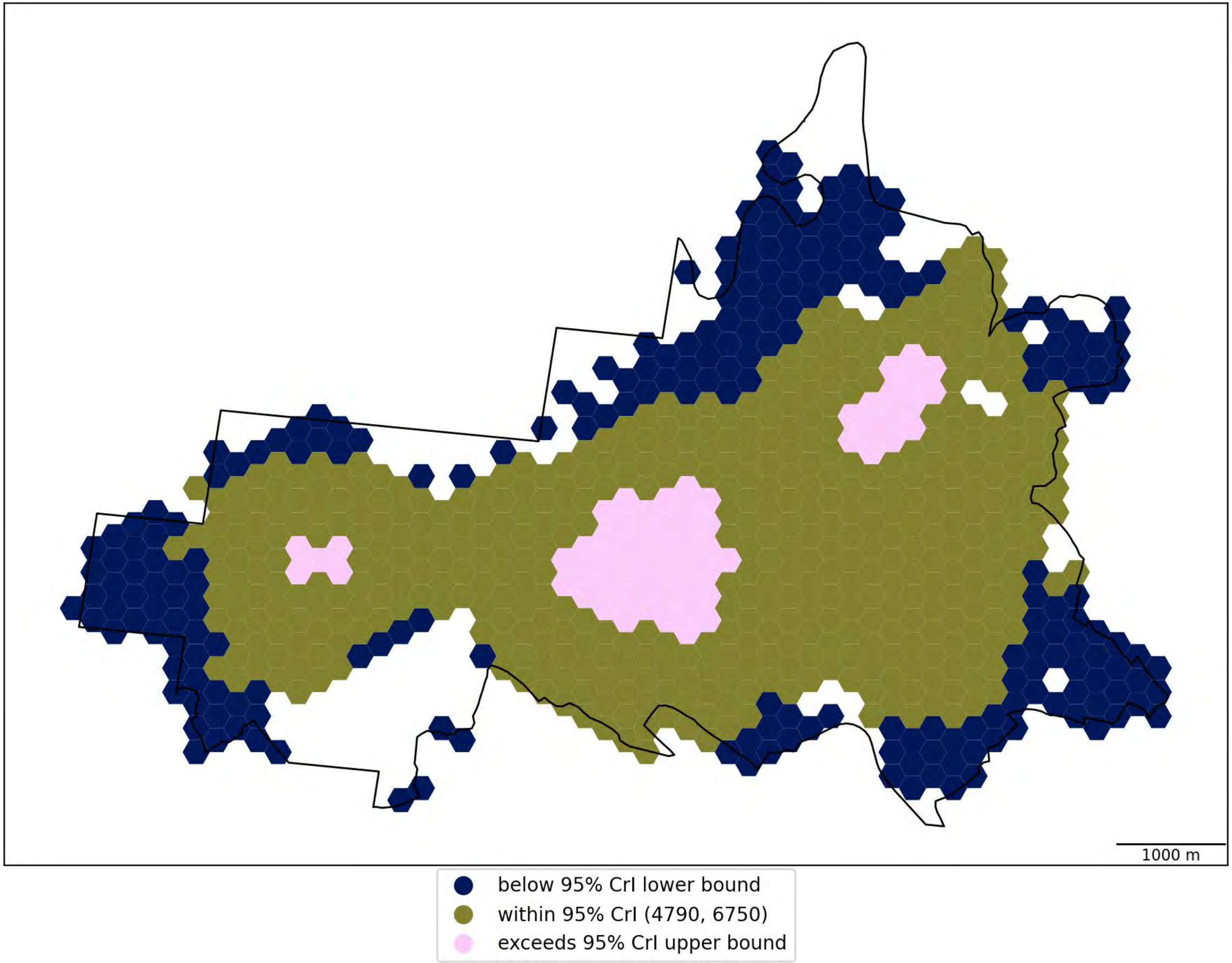

B: Estimated Mean 1000 m neighbourhood population per km² requirement for reaching the WHO’s target of a ≥15% relative reduction in insufficient physical activity through walking

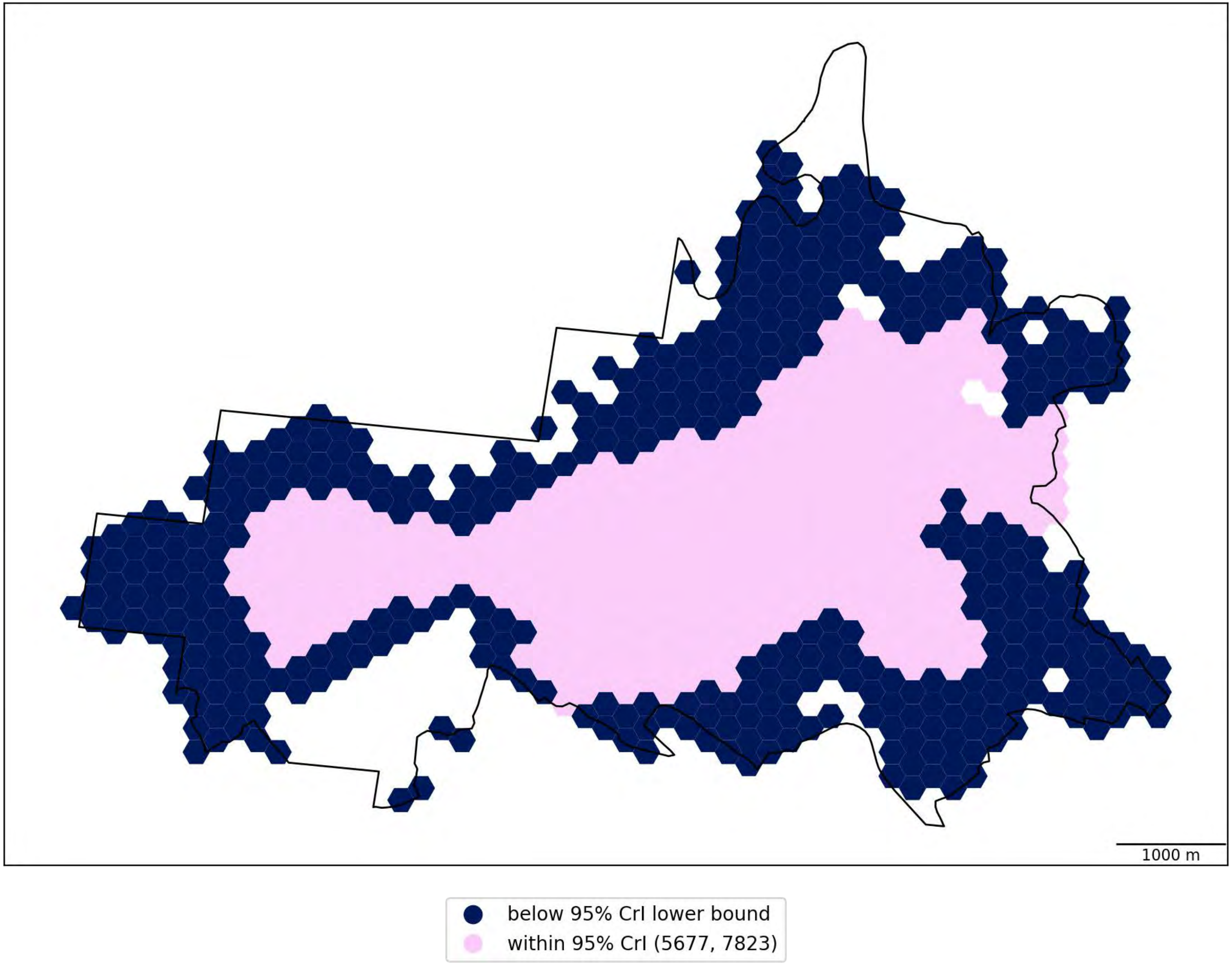

Histogram of Mean 1000 m neighbourhood population per km².

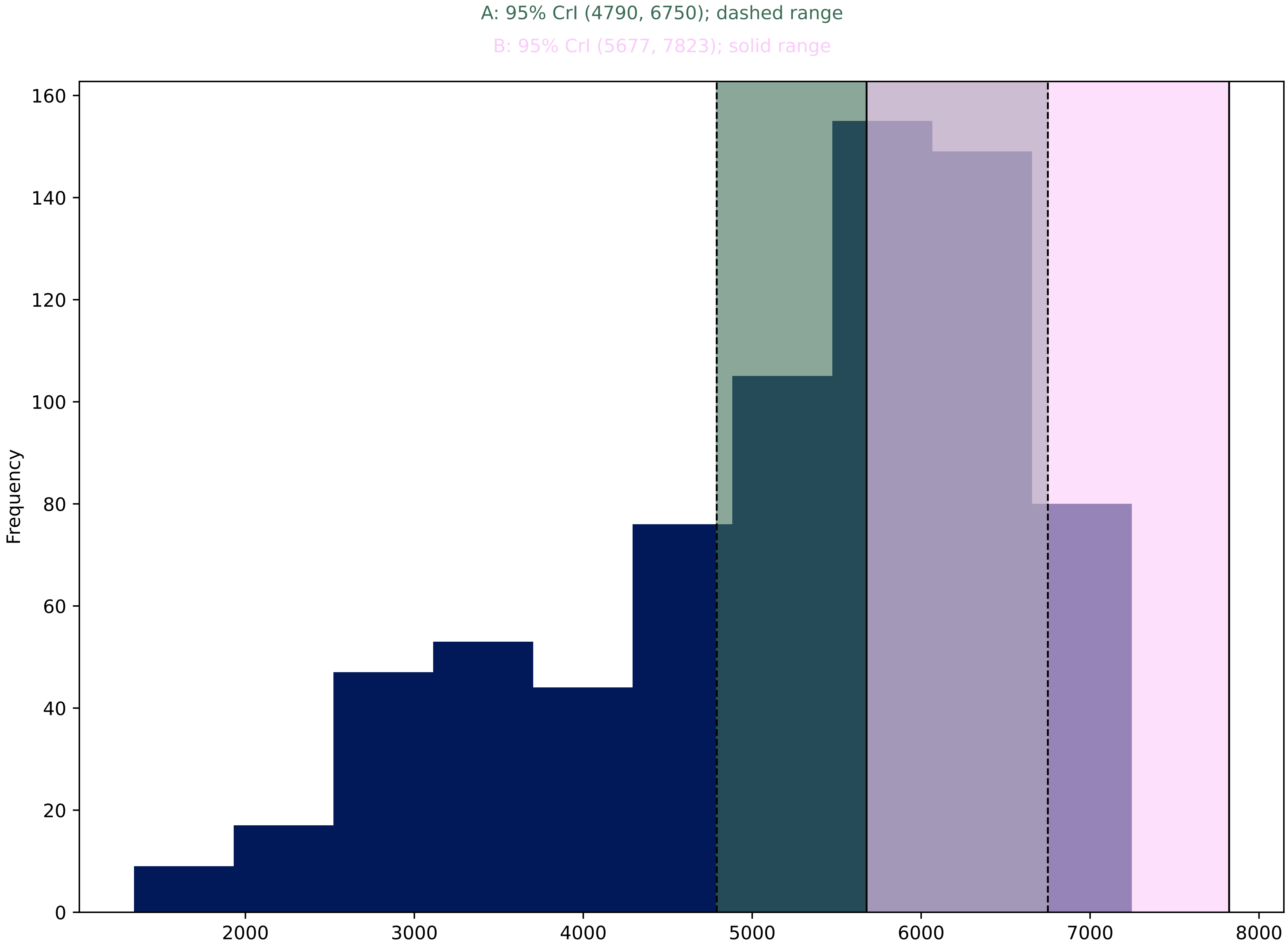

Mean 1000 m neighbourhood street intersections per km²

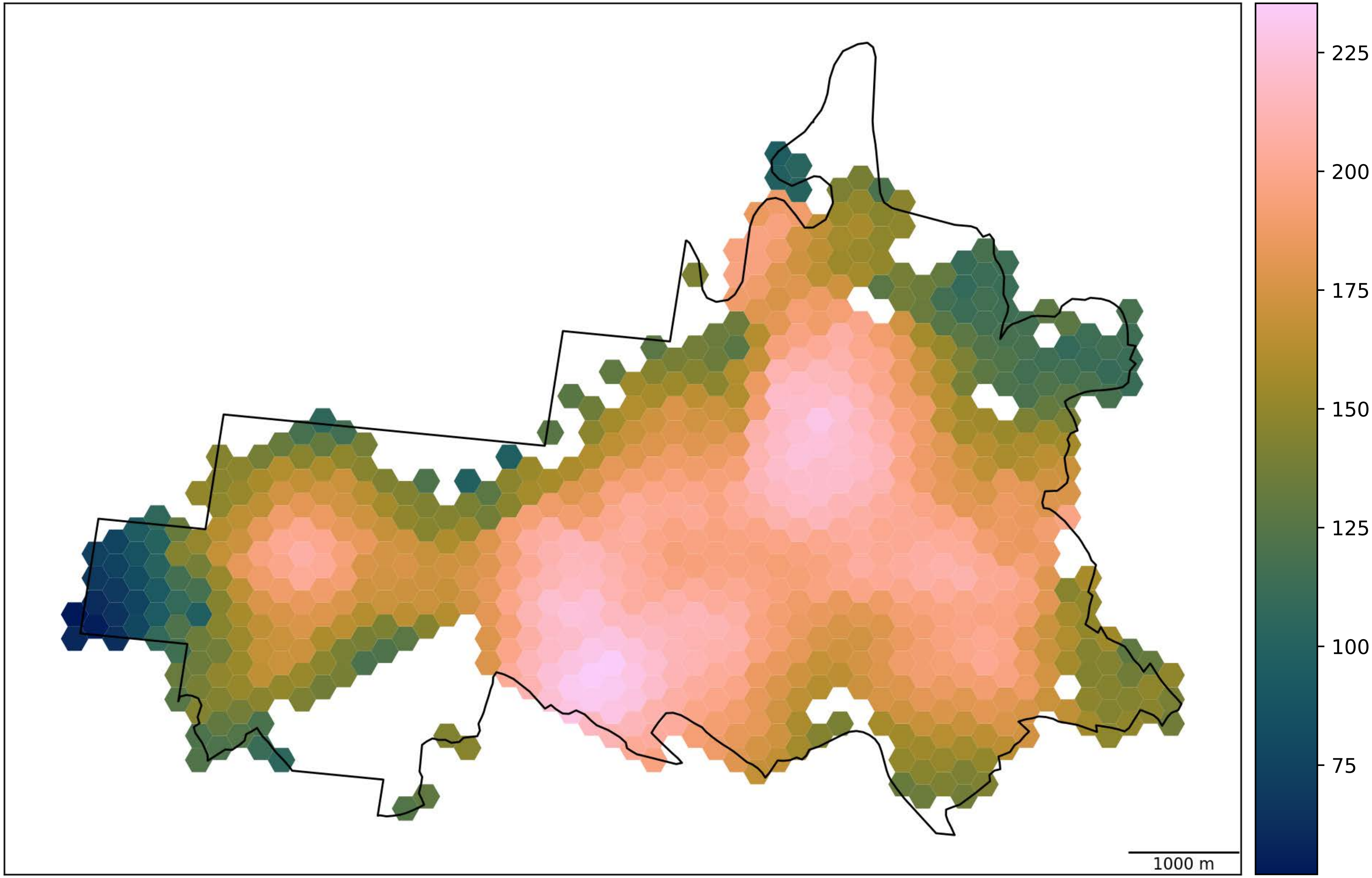

A: Estimated Mean 1000 m neighbourhood street intersections per km² requirement for ≥80% probability of engaging in walking for transport

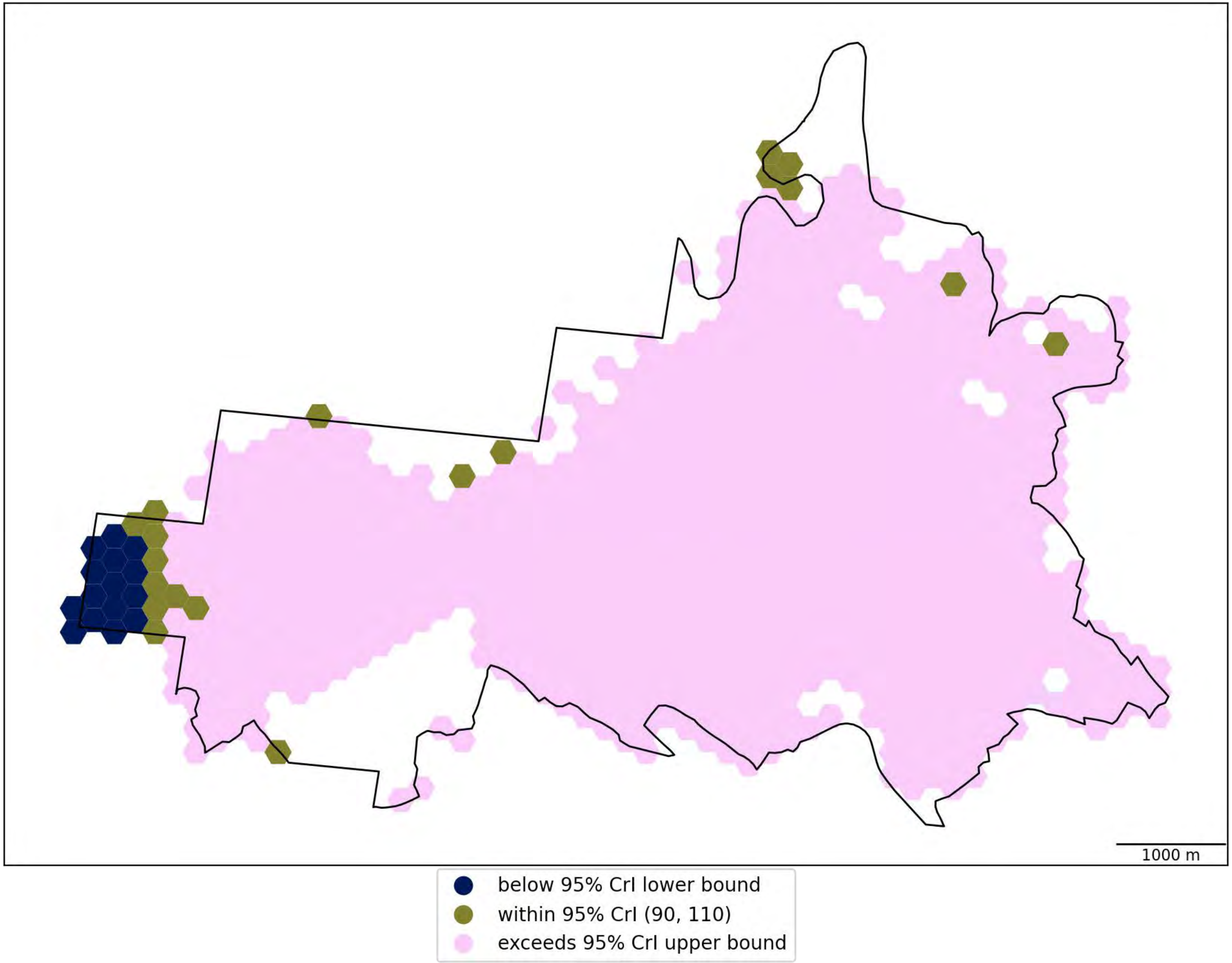

B: Estimated Mean 1000 m neighbourhood street intersections per km² requirement for reaching the WHO's target of a ≥15% relative reduction in insufficient physical activity through walking

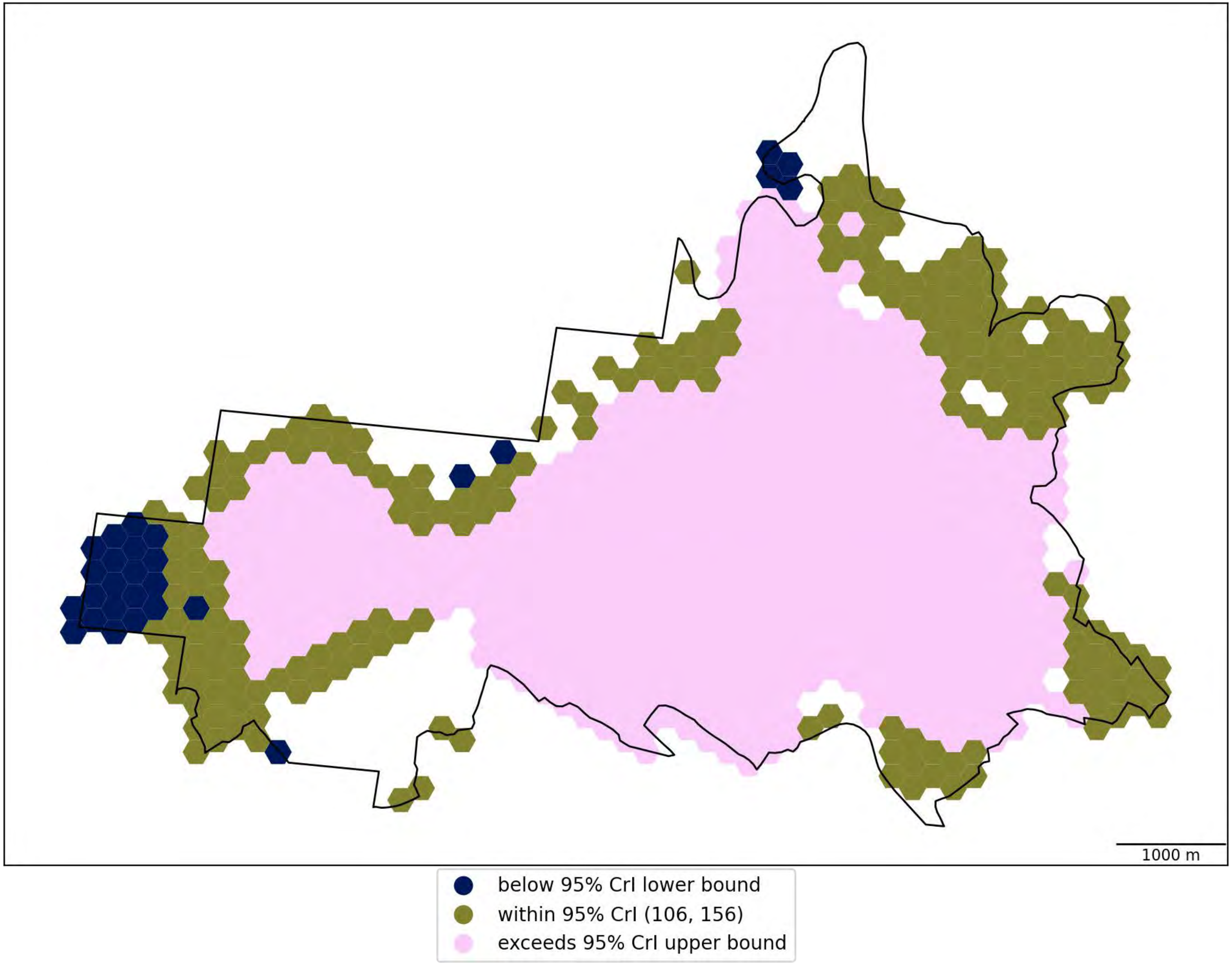

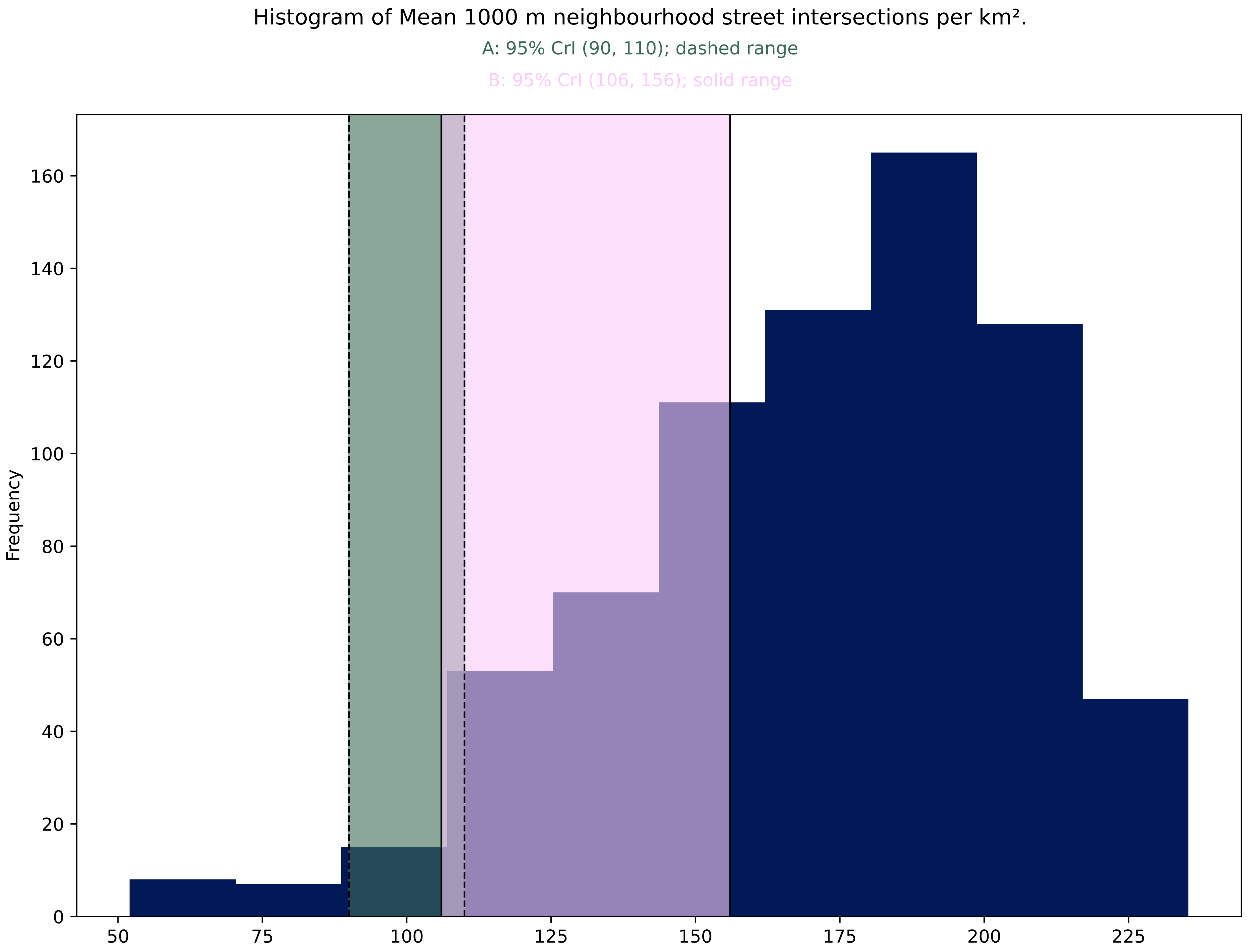
Histogram of Mean 1000 m neighbourhood street intersections per km².
A: 95% CrI (90, 110); dashed range
B: 95% CrI (106, 156); solid range
Frequency
0
20
40
60
80
100
120
140
160
50
75
100
125
150
175
200
225

Distance to nearest public transport stops (m; up to 500m)

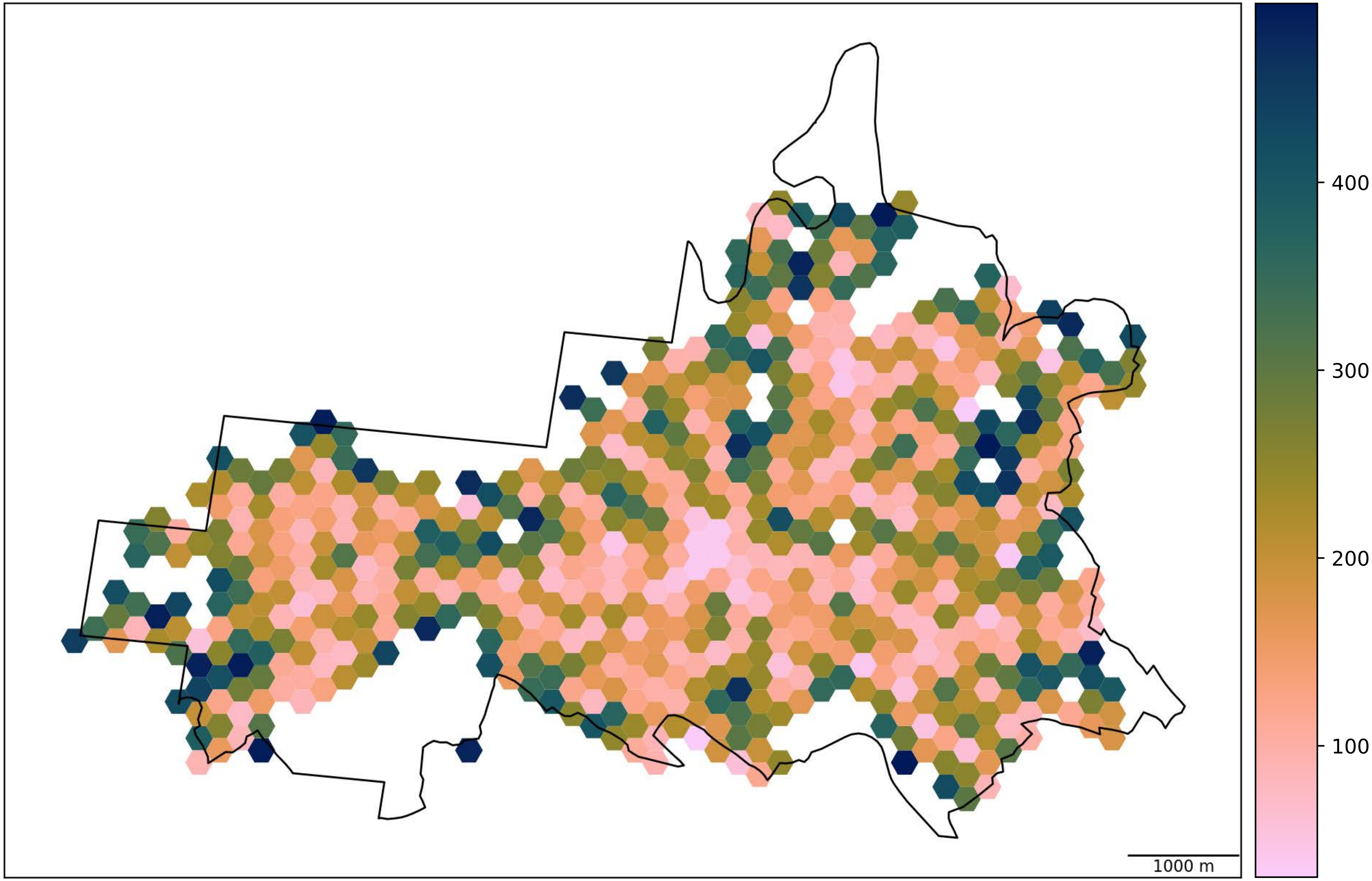

distances: Estimated Distance to nearest public transport stops (m; up to 500m) requirement for distances to destinations, measured up to a maximum distance target threshold of 500 metres

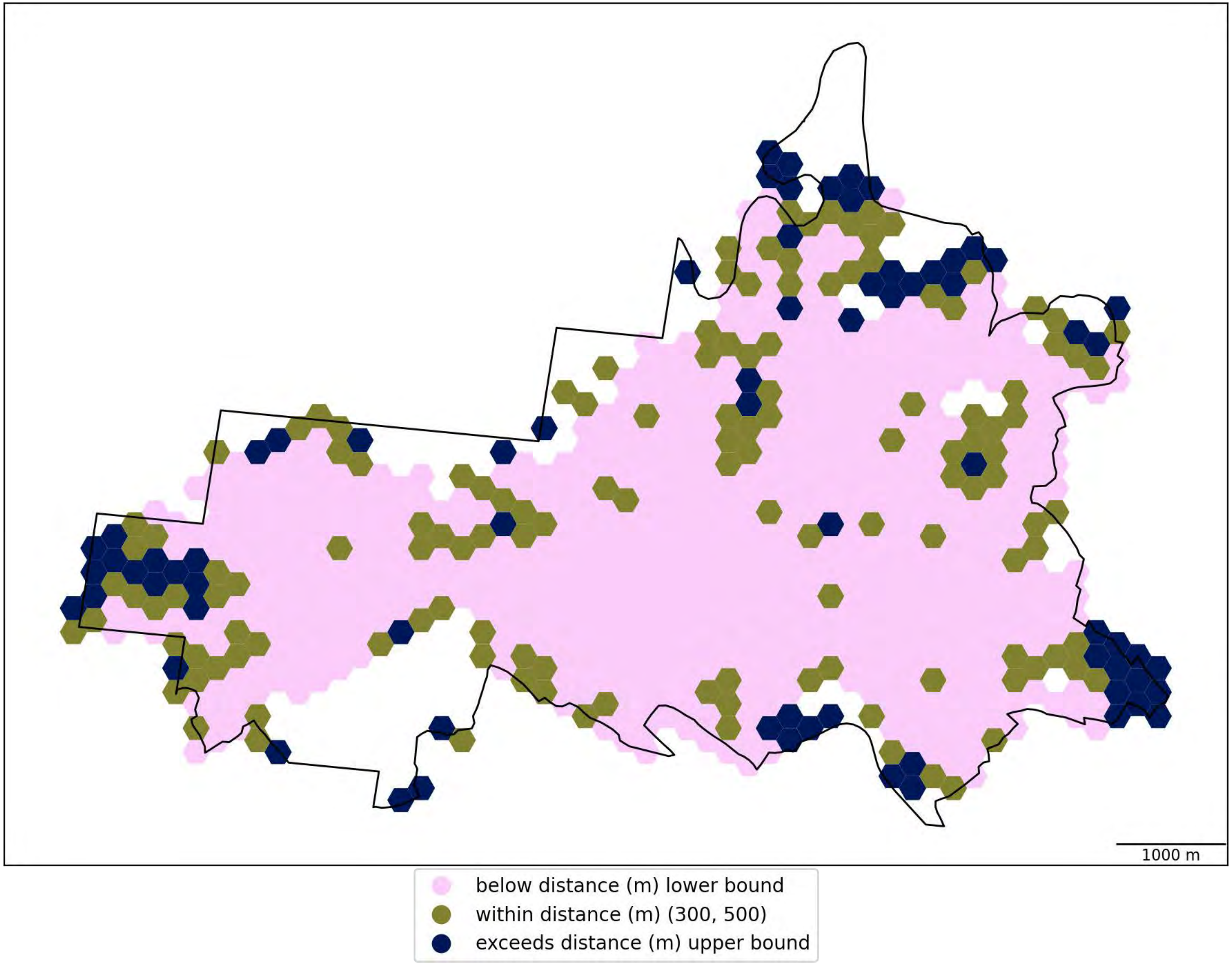

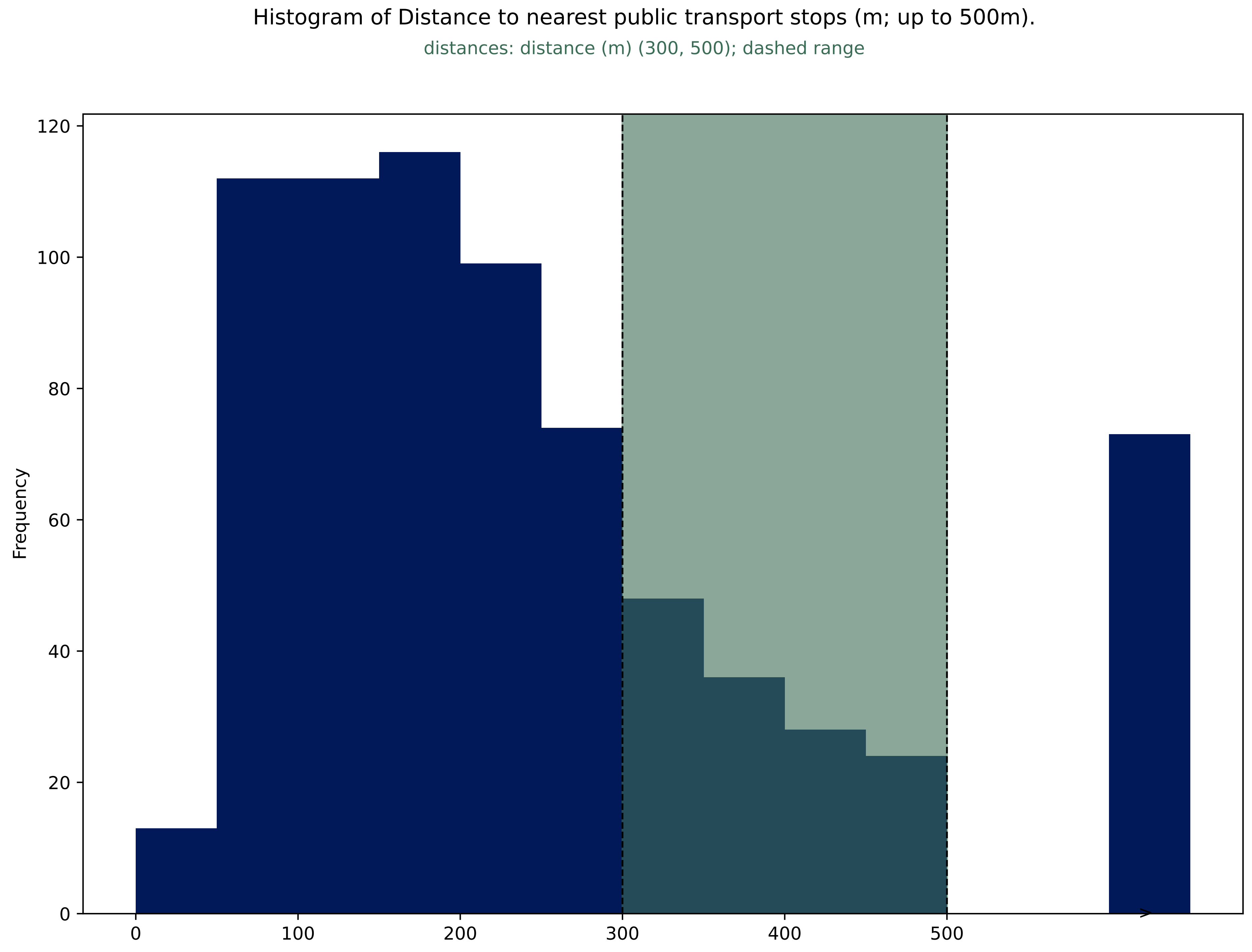

Histogram of Distance to nearest public transport stops (m; up to 500m).
distances: distance (m) (300, 500); dashed range
Frequency
0
20
40
60
80
100
120
0
100
200
300
400
500
>

Distance to nearest park (m; up to 500m)

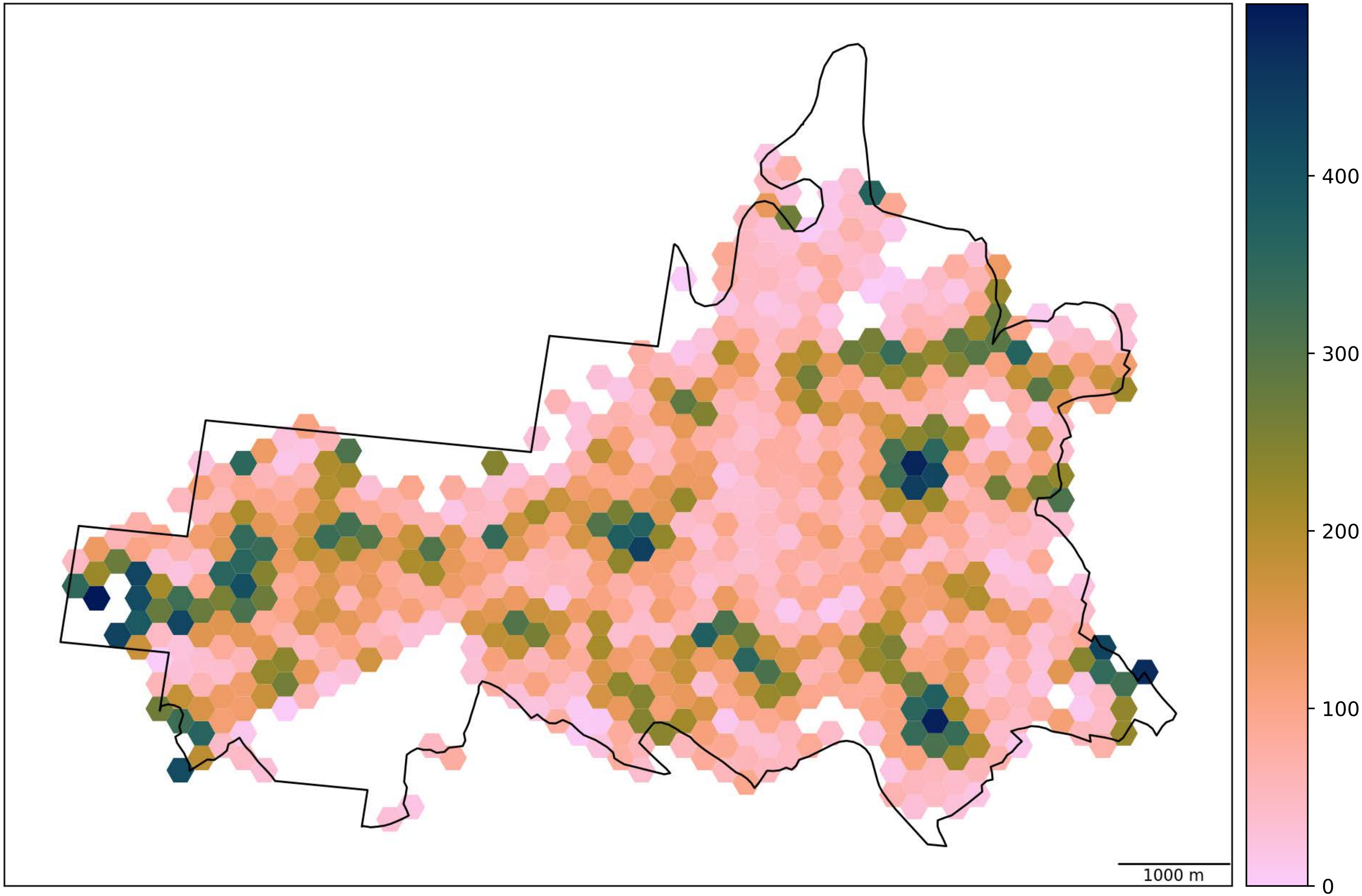

distances: Estimated Distance to nearest park (m; up to 500m) requirement for distances to destinations, measured up to a maximum distance target threshold of 500 metres

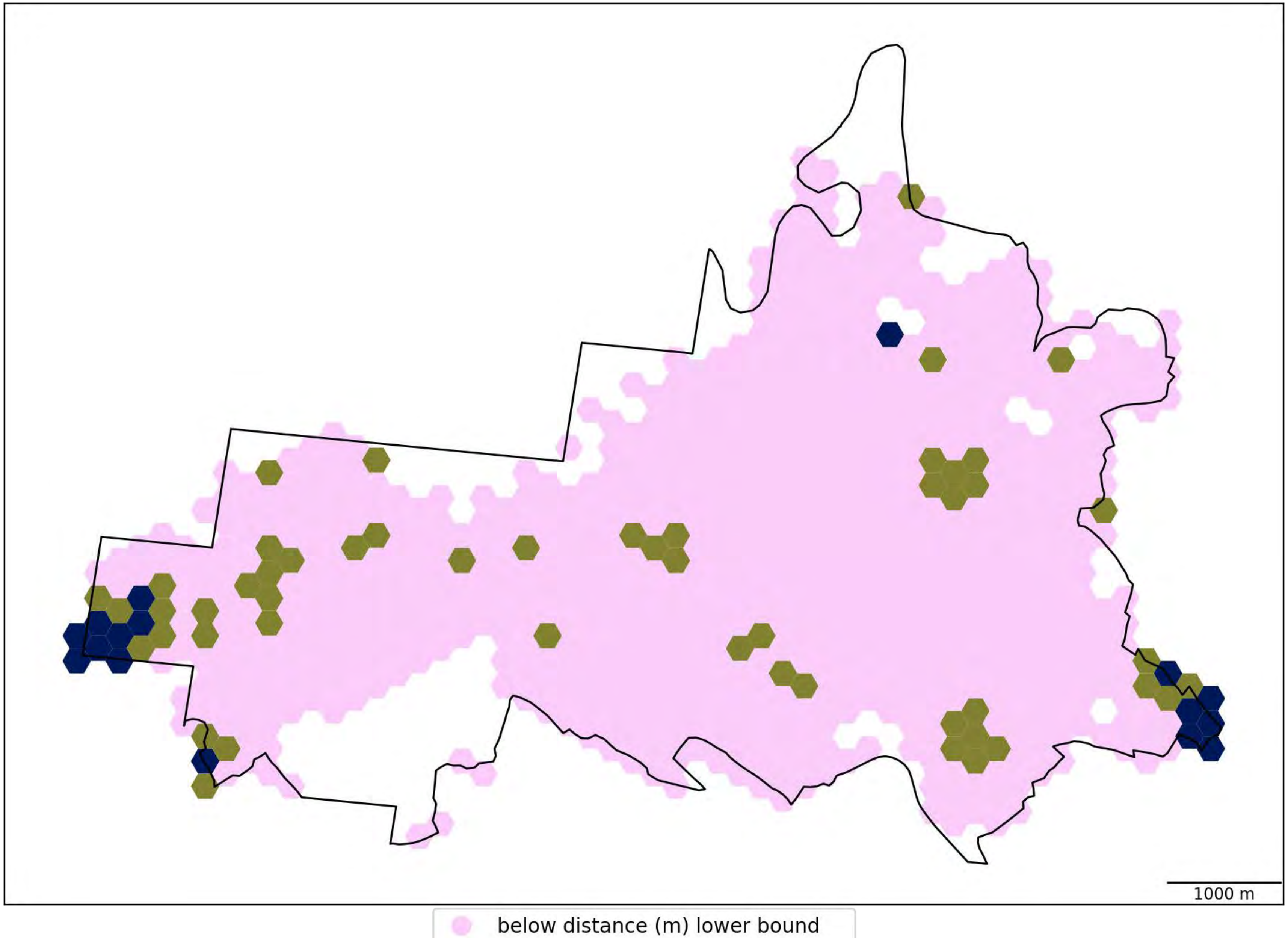

Histogram of Distance to nearest park (m; up to 500m).

distances: distance (m) (300, 500); dashed range

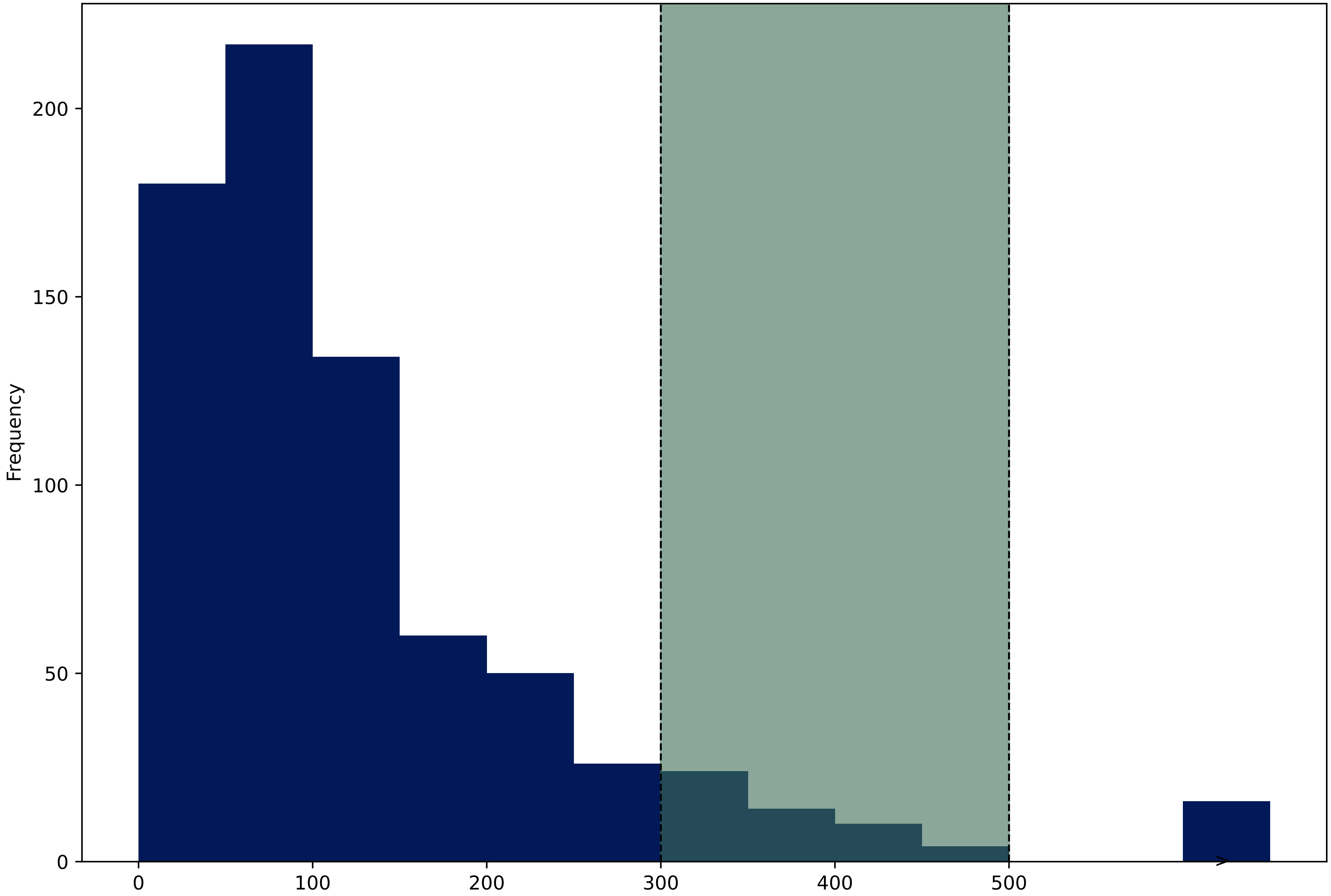

Europe, United Kingdom, Belfast

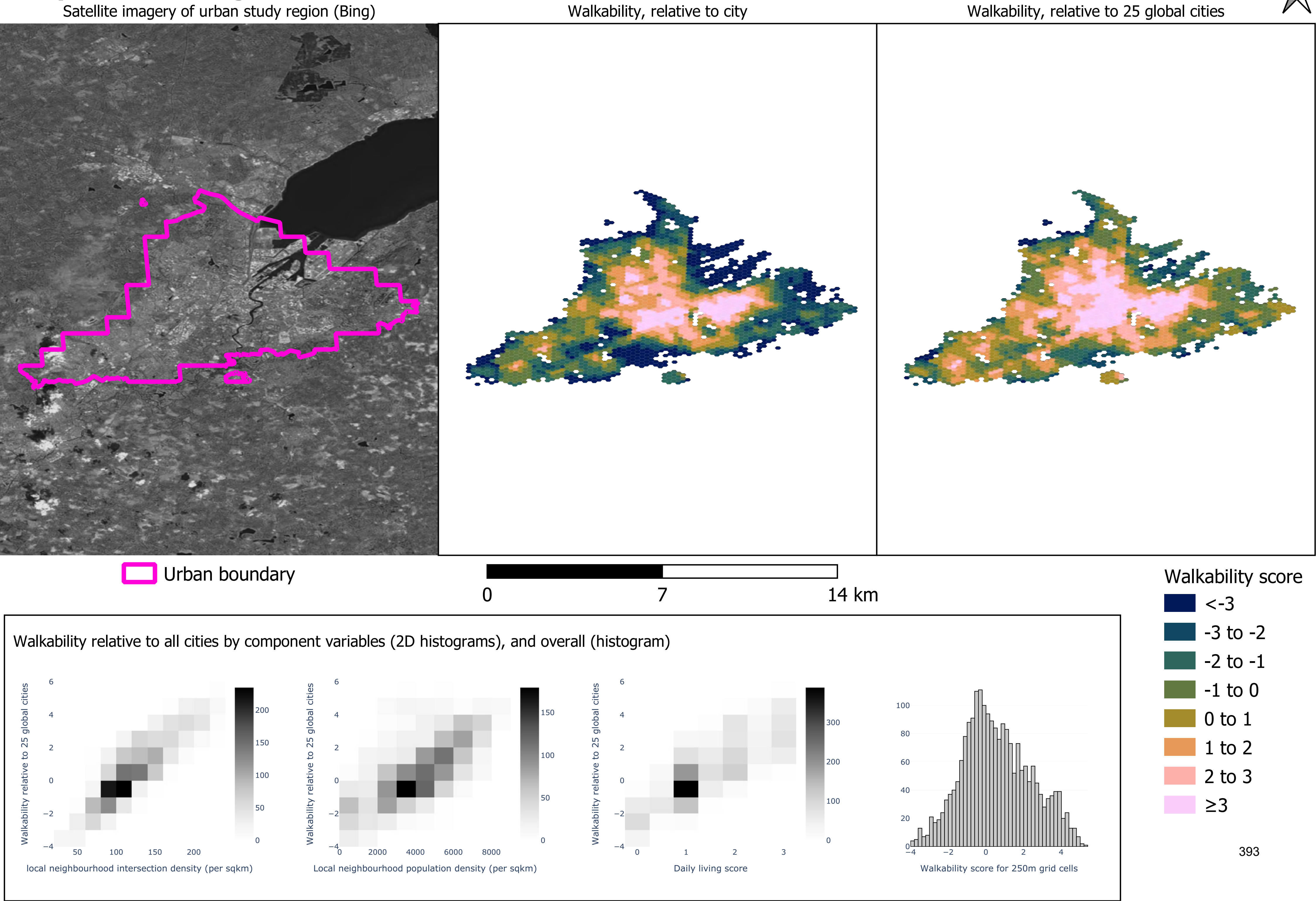

Mean 1000 m neighbourhood population per km²

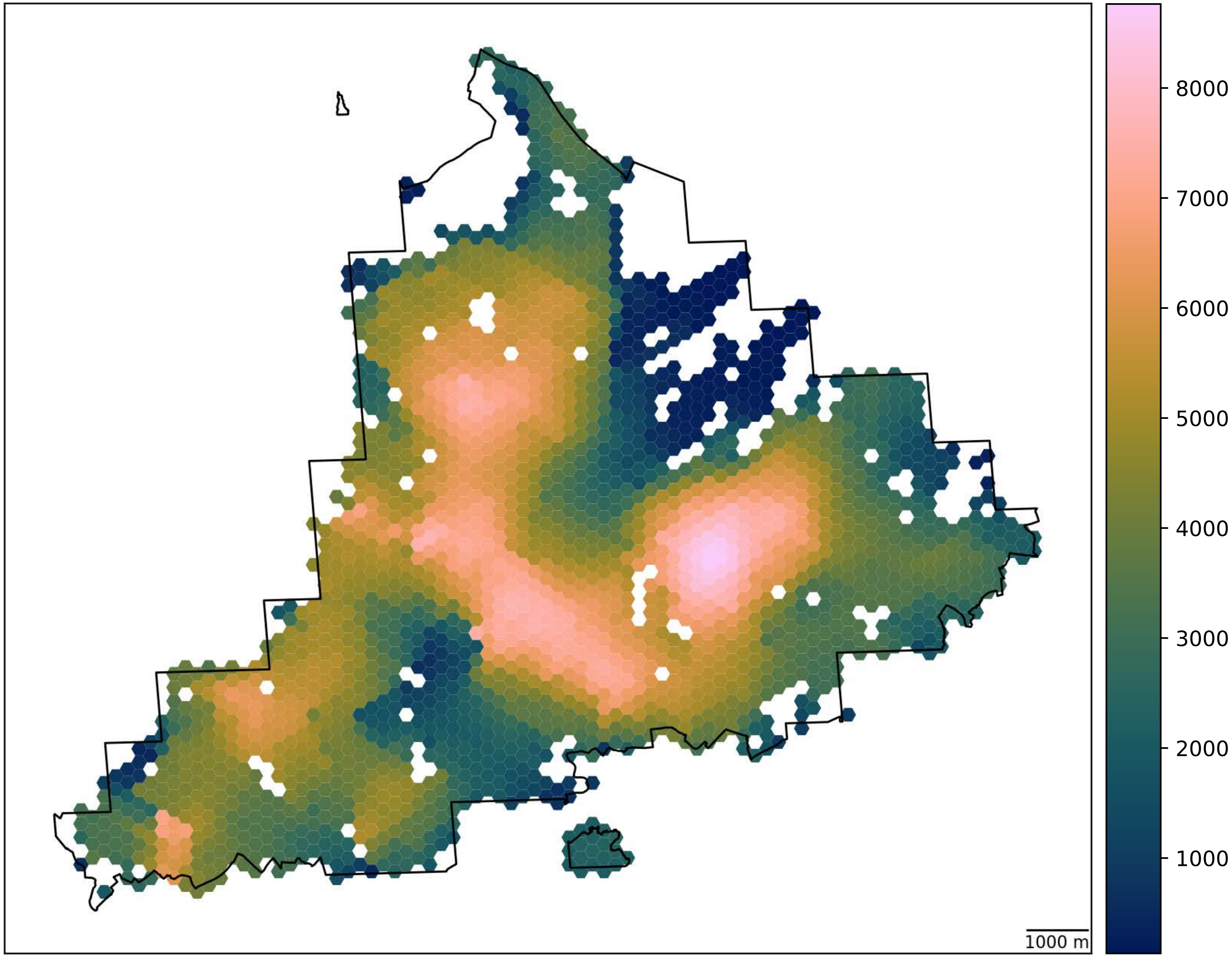

A: Estimated Mean 1000 m neighbourhood population per km² requirement for ≥80% probability of engaging in walking for transport

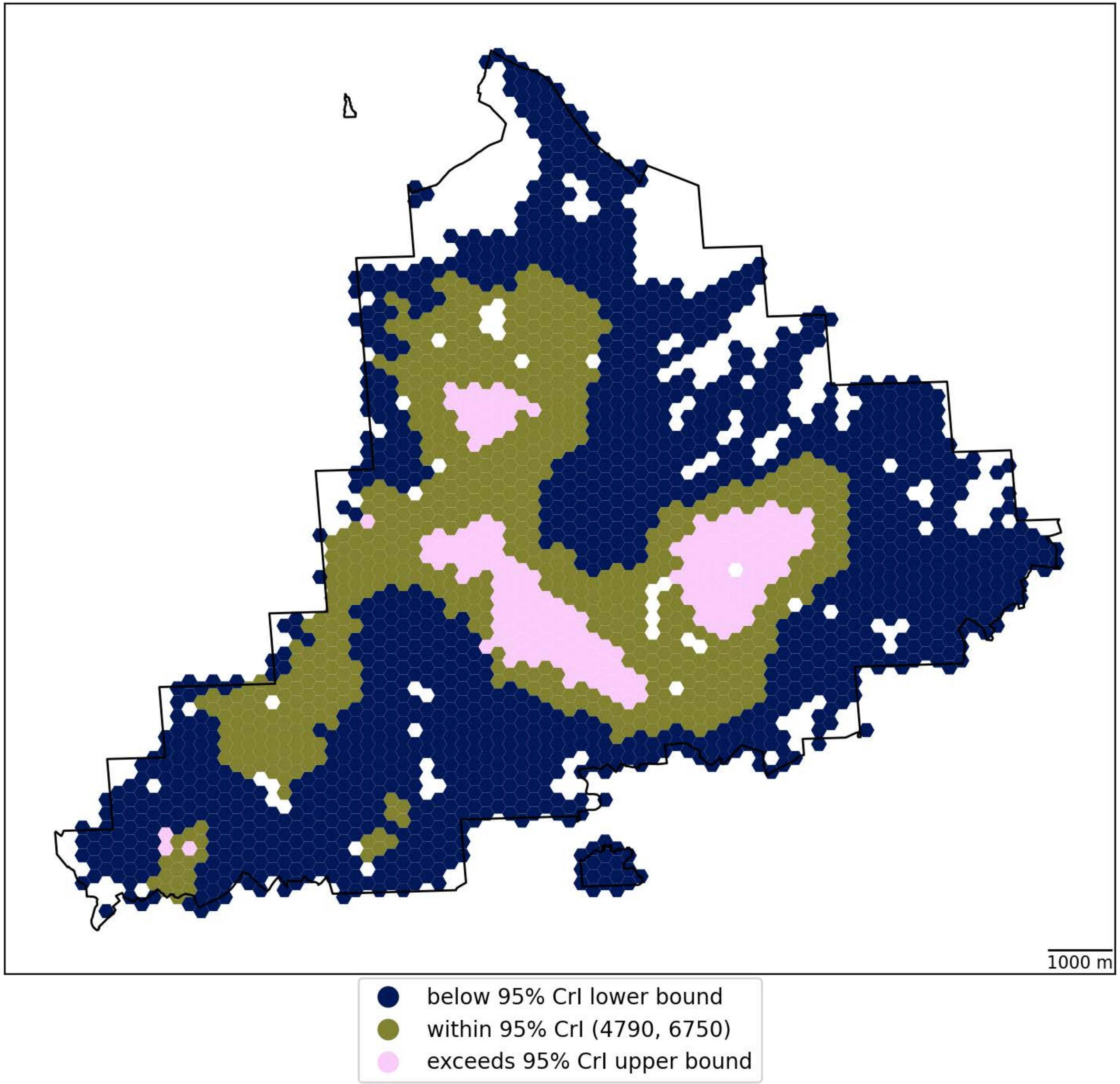

B: Estimated Mean 1000 m neighbourhood population per km² requirement for reaching the WHO's target of a ≥15% relative reduction in insufficient physical activity through walking

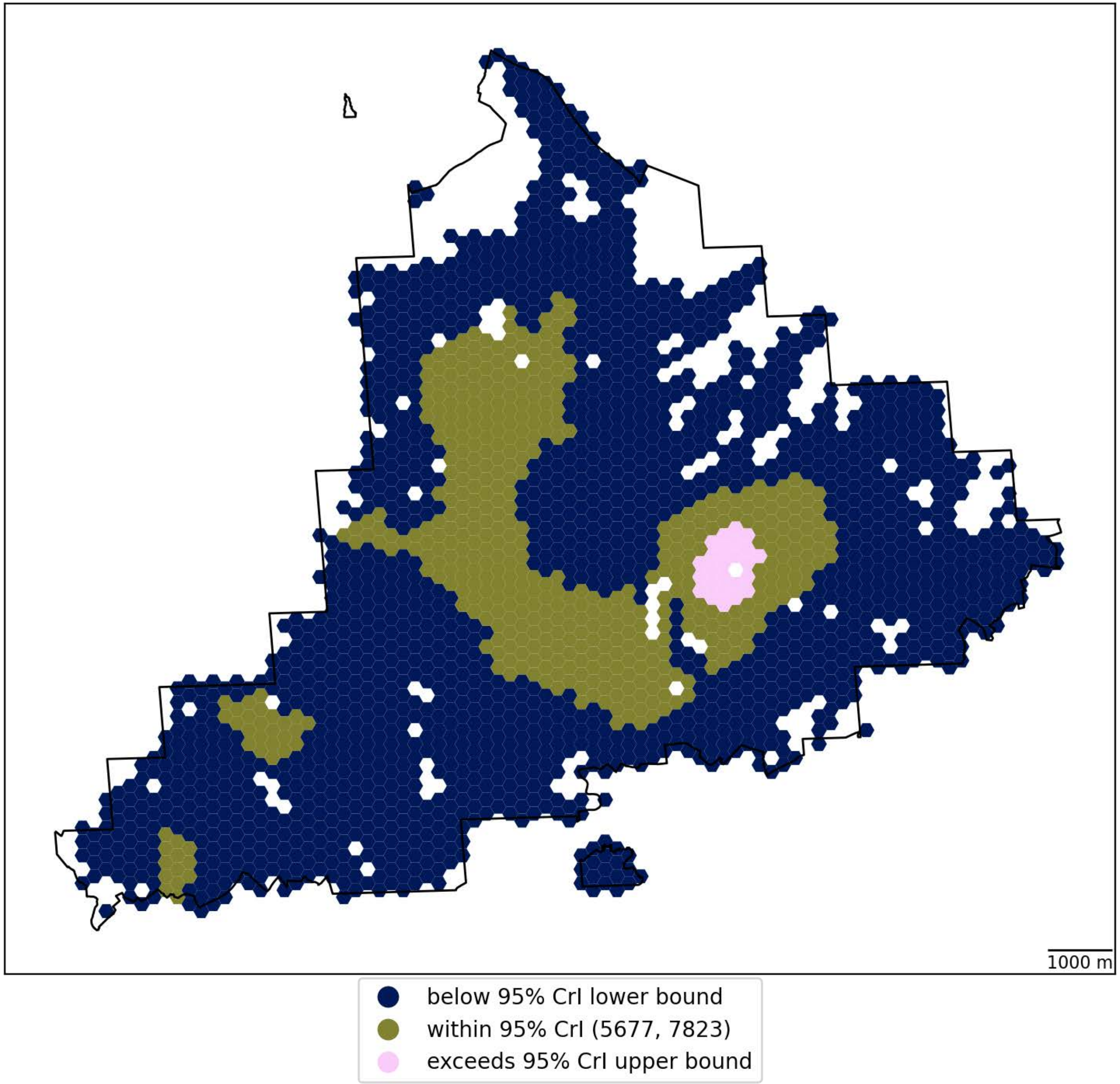

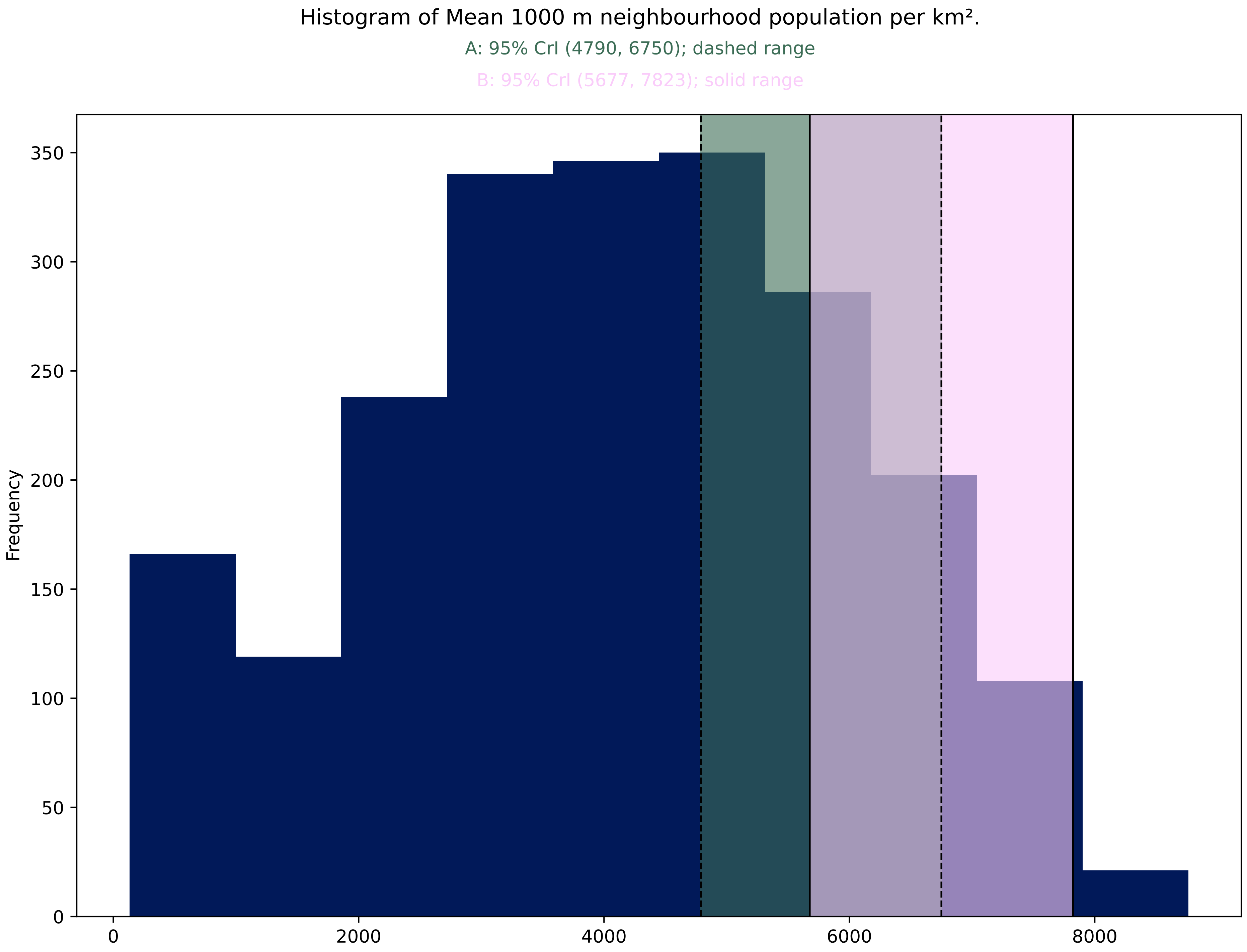

Histogram of Mean 1000 m neighbourhood population per km².
A: 95% CrI (4790, 6750); dashed range
B: 95% CrI (5677, 7823); solid range
Frequency
0
50
100
150
200
250
300
350
0
2000
4000
6000
8000

Mean 1000 m neighbourhood street intersections per km²

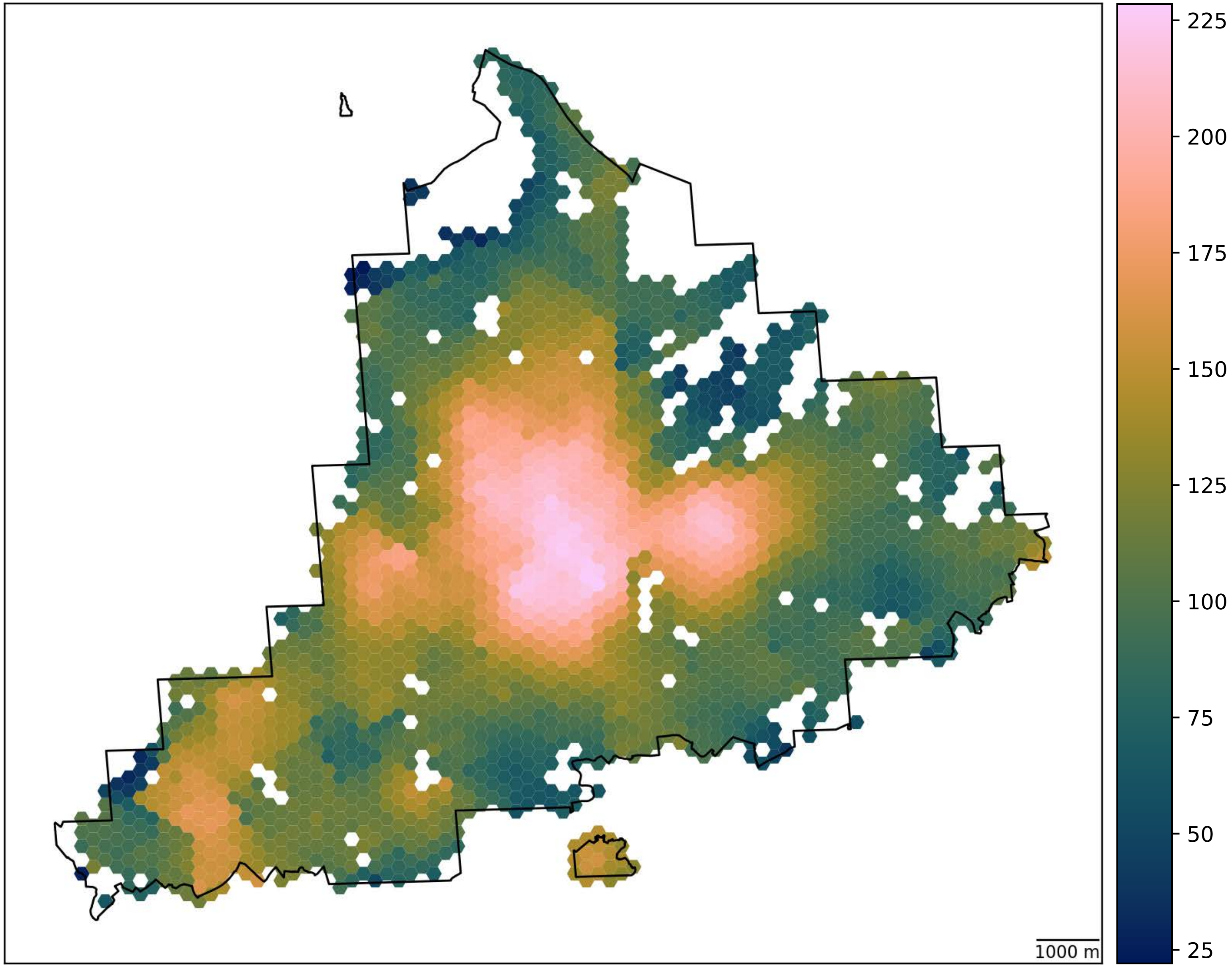

A: Estimated Mean 1000 m neighbourhood street intersections per km² requirement for ≥80% probability of engaging in walking for transport

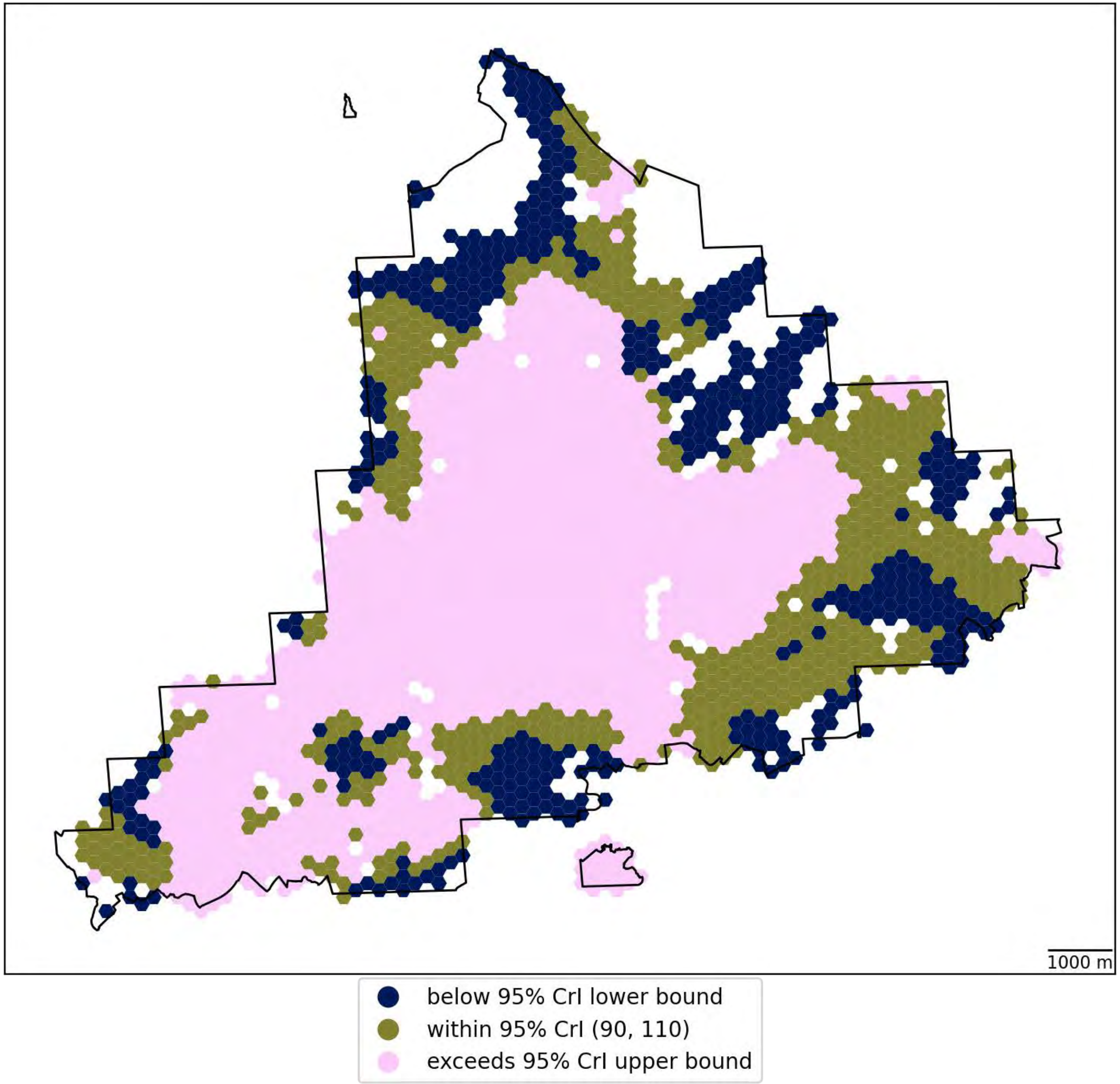

B: Estimated Mean 1000 m neighbourhood street intersections per km² requirement for reaching the WHO’s target of a ≥15% relative reduction in insufficient physical activity through walking

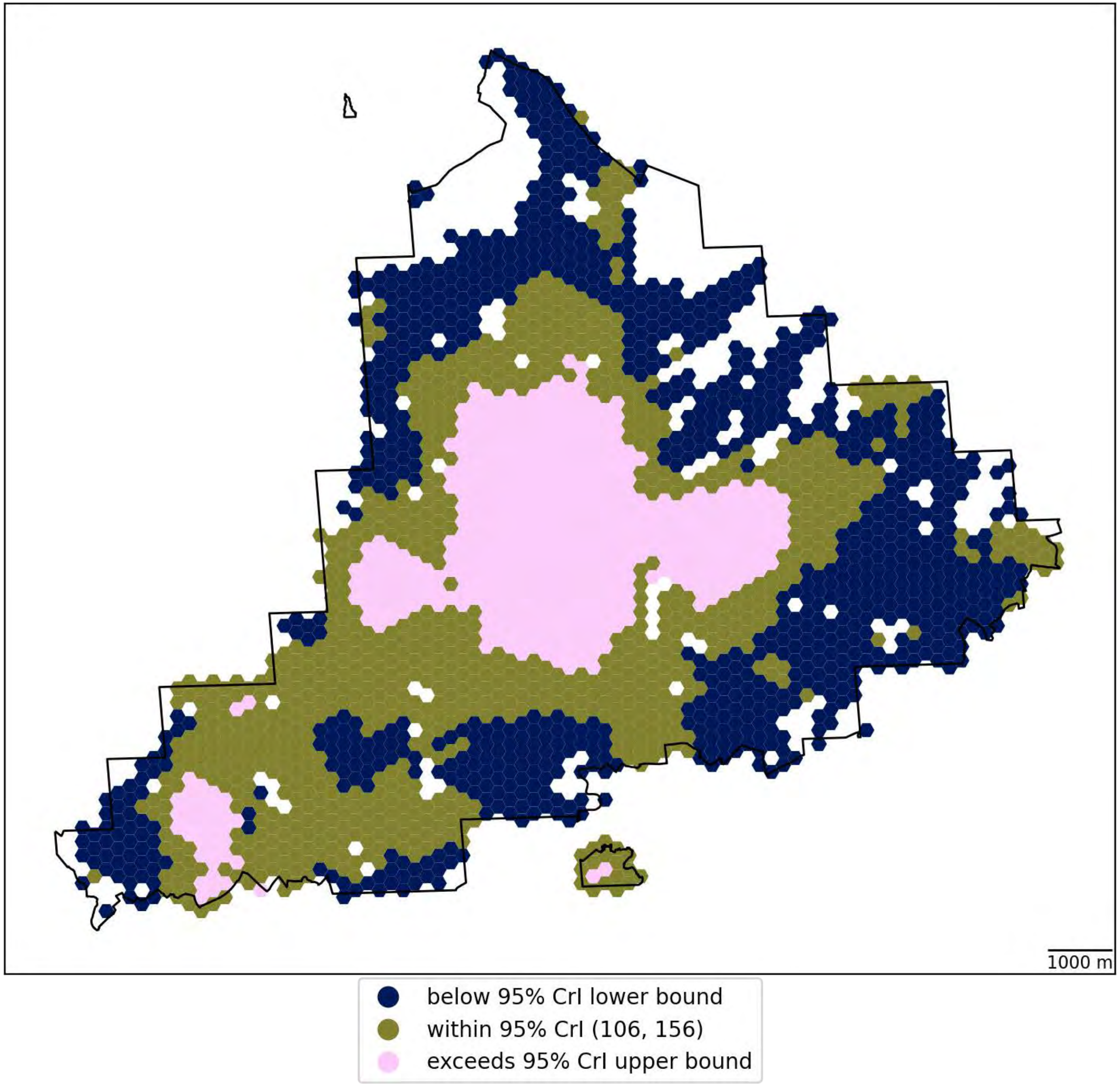

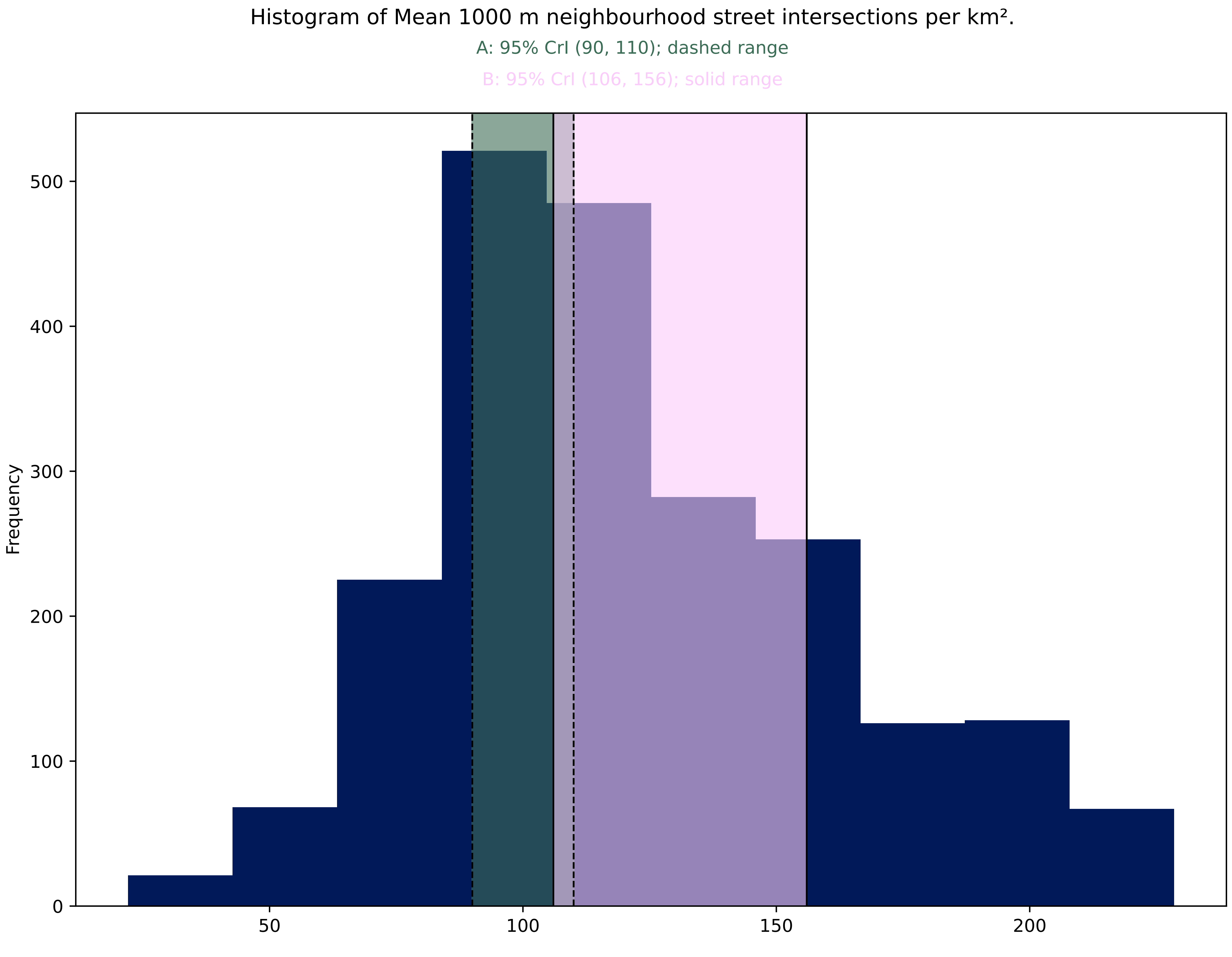

Histogram of Mean 1000 m neighbourhood street intersections per km².
A: 95% CrI (90, 110); dashed range
B: 95% CrI (106, 156); solid range
Frequency
0
100
200
300
400
500
50
100
150
200

Distance to nearest public transport stops (m; up to 500m)

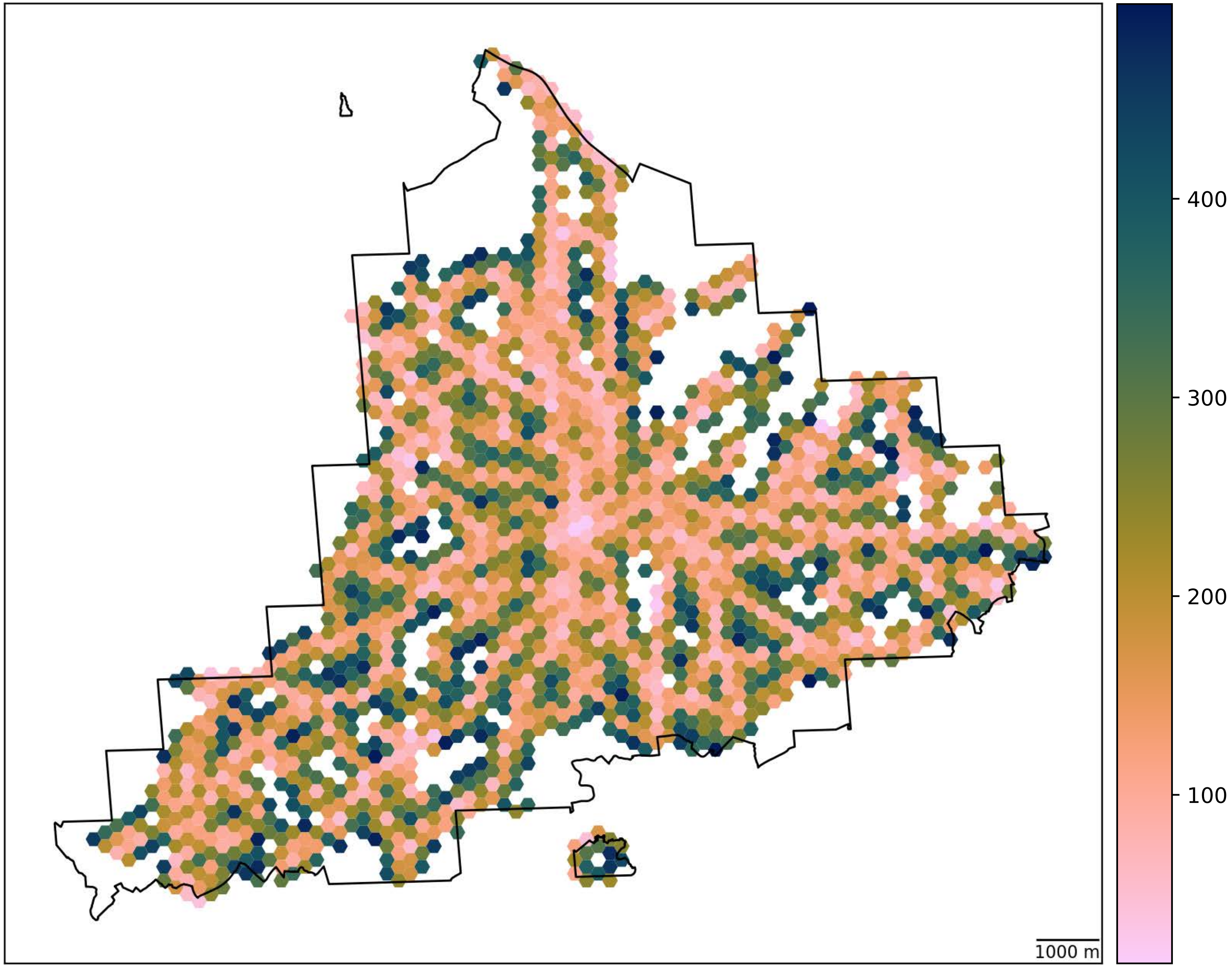

distances: Estimated Distance to nearest public transport stops (m; up to 500m) requirement for distances to destinations, measured up to a maximum distance target threshold of 500 metres

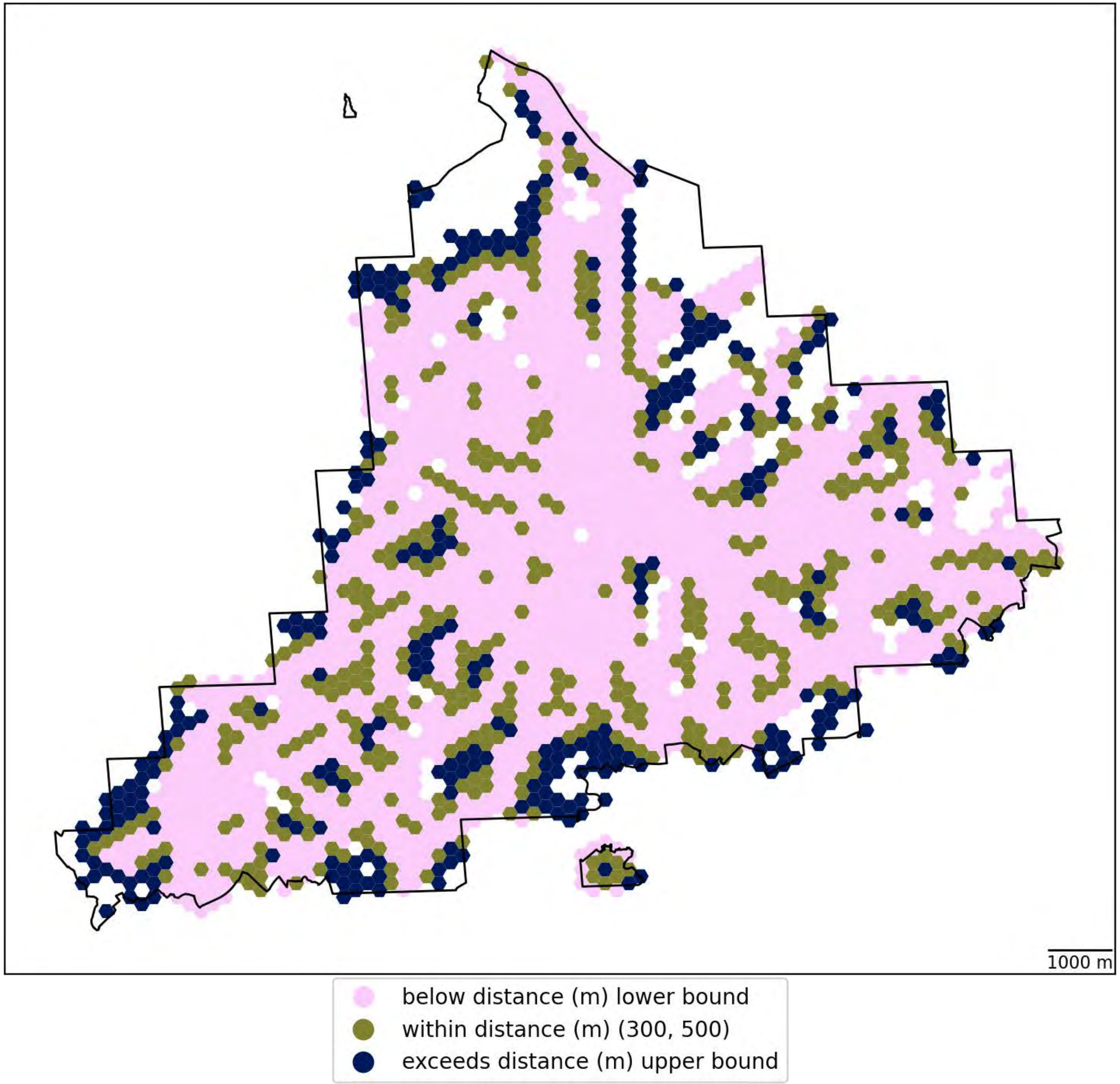

Histogram of Distance to nearest public transport stops (m; up to 500m).

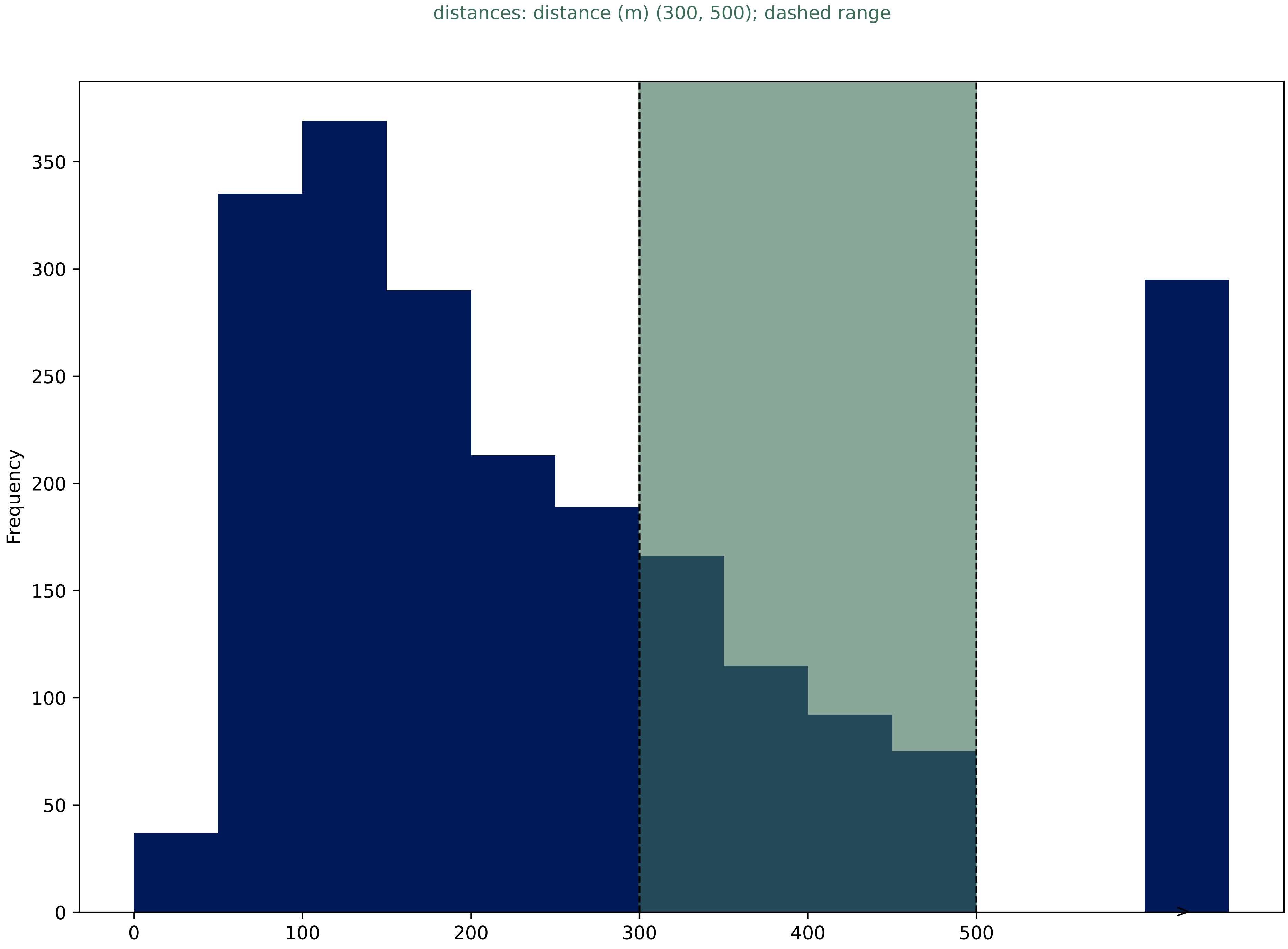

Distance to nearest park (m; up to 500m)

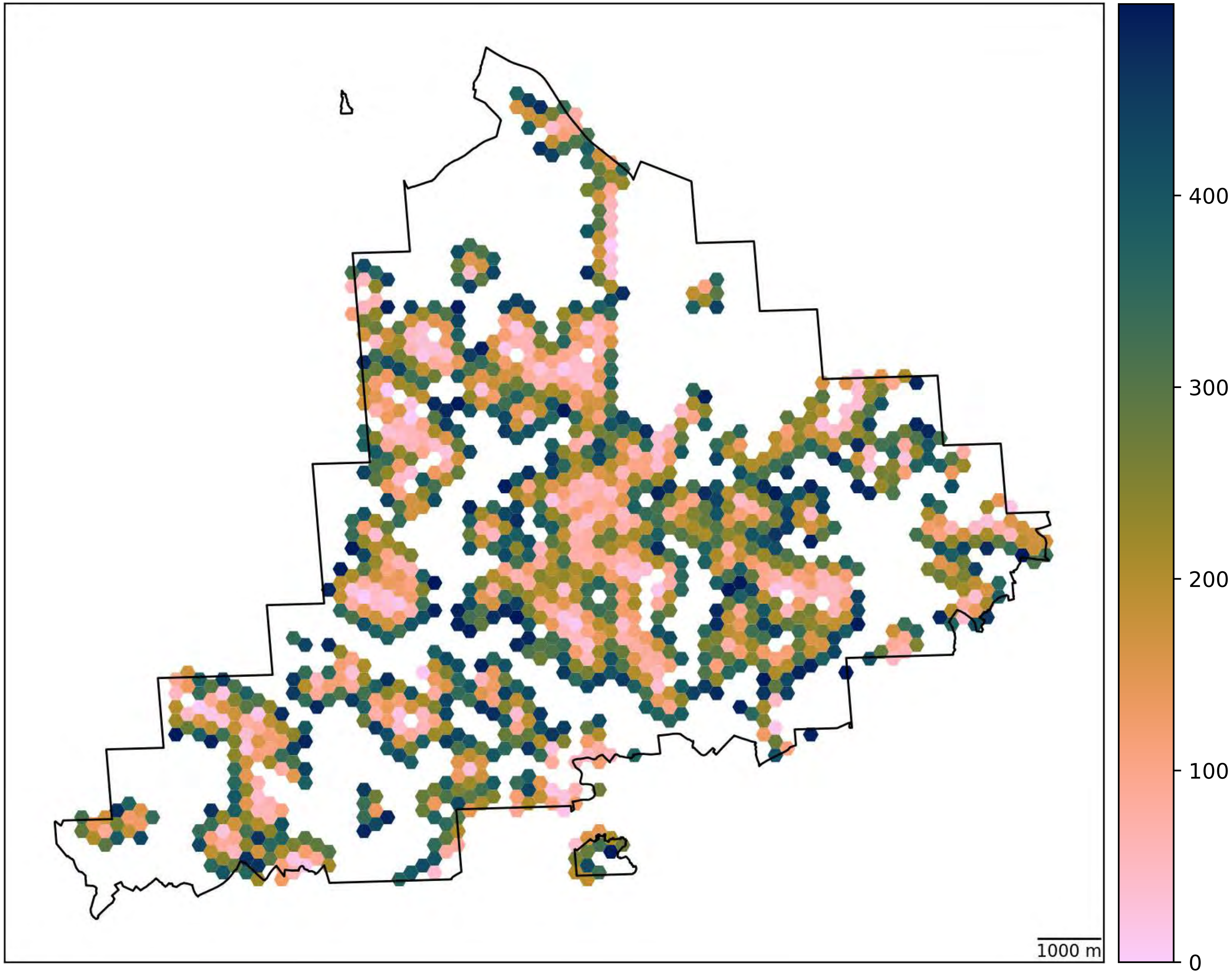

distances: Estimated Distance to nearest park (m; up to 500m) requirement for distances to destinations, measured up to a maximum distance target threshold of 500 metres

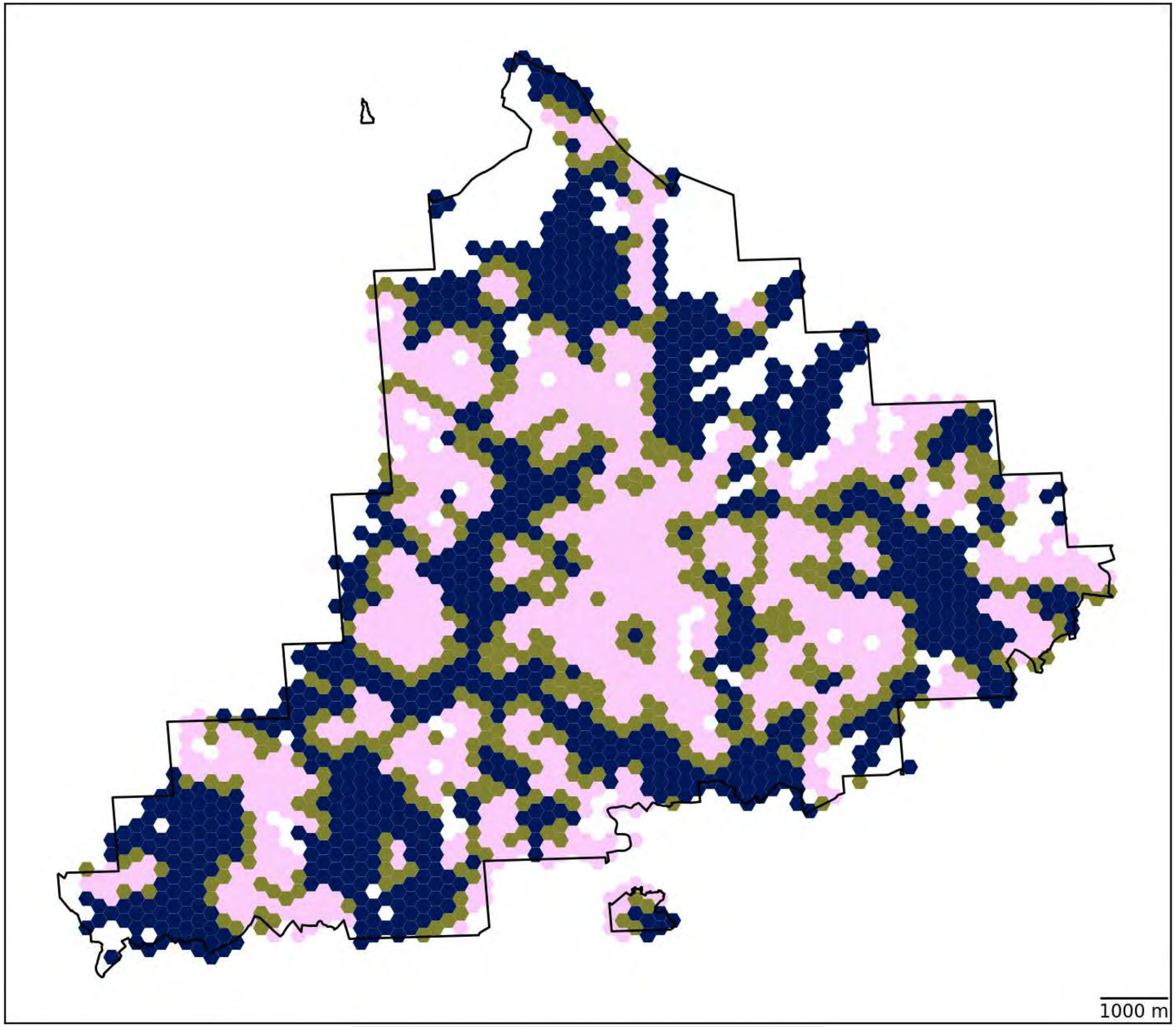

below distance (m) lower bound
within distance (m) (300, 500)
exceeds distance (m) upper bound

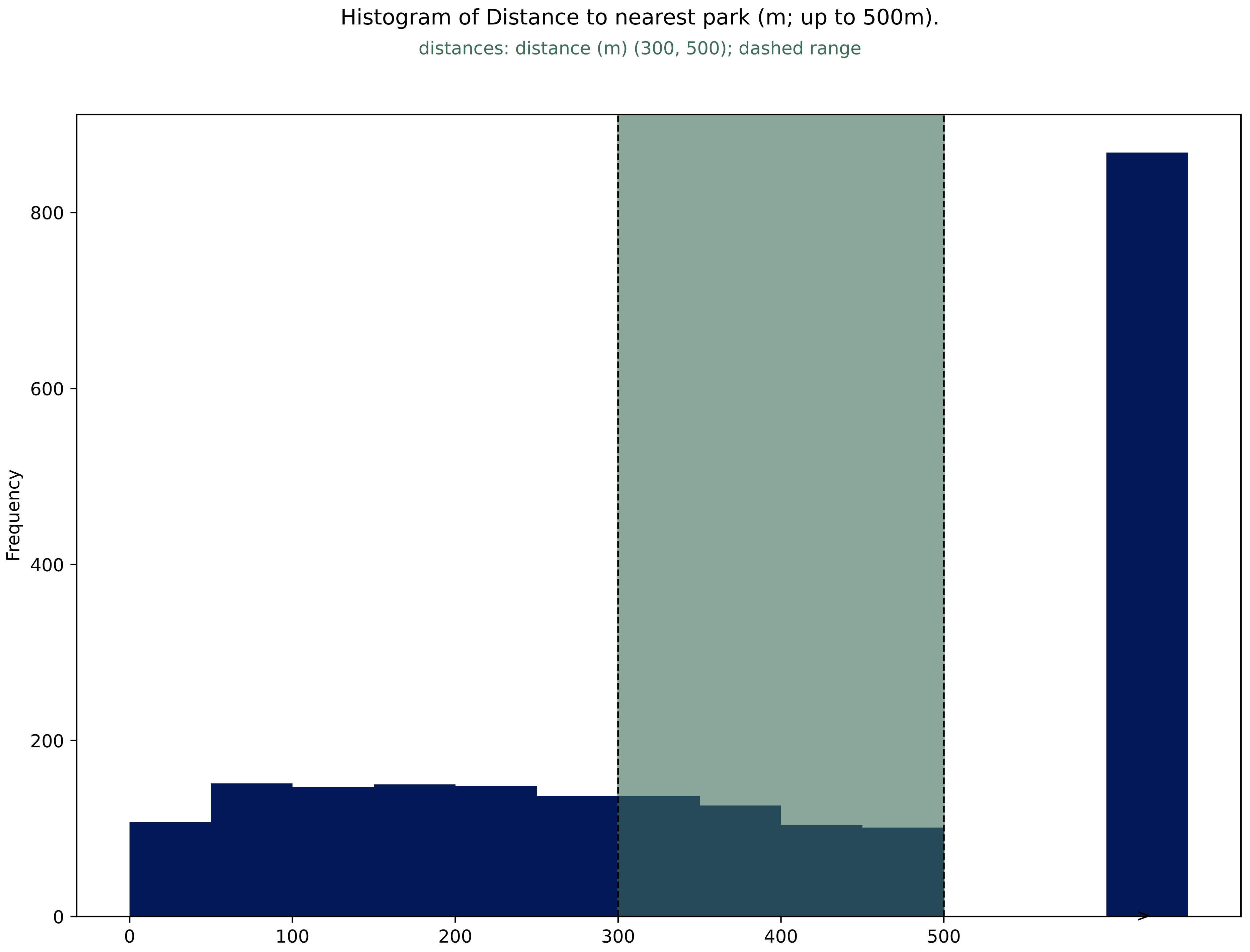
Histogram of Distance to nearest park (m; up to 500m).
distances: distance (m) (300, 500); dashed range
Frequency
0
200
400
600
800
0
100
200
300
400
500
>
